# Synthesis of azulene fluorophores reveals structure–property relationships

**DOI:** 10.1039/d6qo01099g

**Published:** 2026-10-12

**Authors:** Chiara Faverio, João Gabriel Ribeiro, Mehmet Kaplan, Gabriele Kociok-Köhn, Tony D. James, G. Dan Pantoş, Simon E. Lewis

**Affiliations:** a Department of Chemistry, University of Bath Bath BA2 7AY UK S.E.Lewis@bath.ac.uk; b Ankara University, Faculty of Science, Department of Chemistry 06100 Ankara Turkiye mehmetkaplan@ankara.edu.tr; c Physical Structure Characterization Facility, University of Bath Bath BA2 7AY UK; d School of Chemistry and Chemical Engineering, Henan Normal University Xinxiang 453007 China

## Abstract

We report the synthesis, photophysical characterisation, and crystallographic analysis of a series of substituted azulenes. The compounds are analogues of the known fluorophore diethyl 2-amino-6-hydroxyazulene-1,3-dicarboxylate, in which the electron-withdrawing groups at C1 and C3 have been retained, but the electron-donating groups at C2 and C6 have been systematically varied. Correlations were observed between the nature of the C2 and C6 substituents and the fluorescence quantum yields and emission maxima; on this basis trends in the structure–property relationship have been elucidated. A novel azulene exhibiting a five-fold increase in quantum yield over the previously known fluorophore has been identified.

## Abstract 2

Systematic variation of substituents at the 2- and 6-positions of azulene-1,3-diesters has shown that the nature of the 6-position substituent profoundly affects the fluorescence quantum yield when a 2-amino group is present.

## Graphical abstract

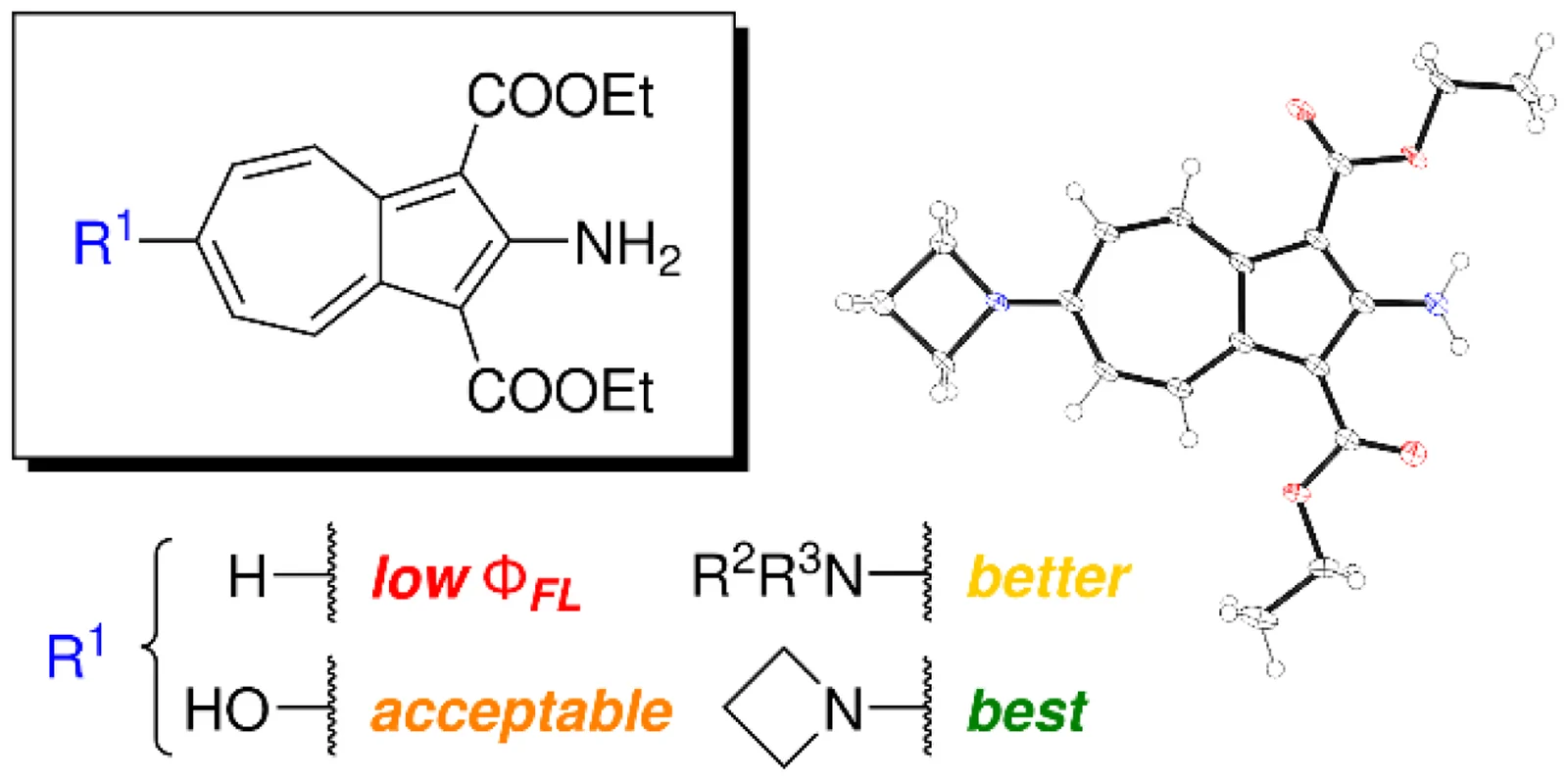

## Introduction

Azulene is a non-benzenoid isomer of naphthalene, which is known for its blue colour and large dipole (1.08 D).^1^ The latter has classically been explained in terms of a resonance structure consisting of fused tropylium and cyclopentadienide rings (Fig. 1a), although recent work highlights that this model cannot explain all observed characteristics of this ring system.^2^ Azulene is also a famous example of a molecule with anomalous fluorescence, which violates Kasha's rule,^3^ emitting from the S_2_ state. This phenomenon has been explained in terms of a relatively large S_1_–S_2_ energy gap,^4^ as well as rapid S_1_ decay *via* a conical intersection,^2,5^ and more recently in terms of the antiaromaticity of the S_1_ state.^2,6^ Accordingly, the azulene motif has been employed in chemical probes having both colorimetric and/or fluorescent readouts. For example, colorimetric sensing of analytes including Hg^2+^,^7^ Ag^+^,^8^ fluoride,^9^ nitrite,^10^ phosphate^11^ and cyanide^12^ has been achieved with azulene-containing probes. The relationship between the electronic characteristics (and position) of substituents on the azulene core and the changes in absorption maxima that result has been studied. Predictive rules have been formulated that may be used for the rational design of azulene-containing colorimetric sensors.^13^

**Fig. 1:**
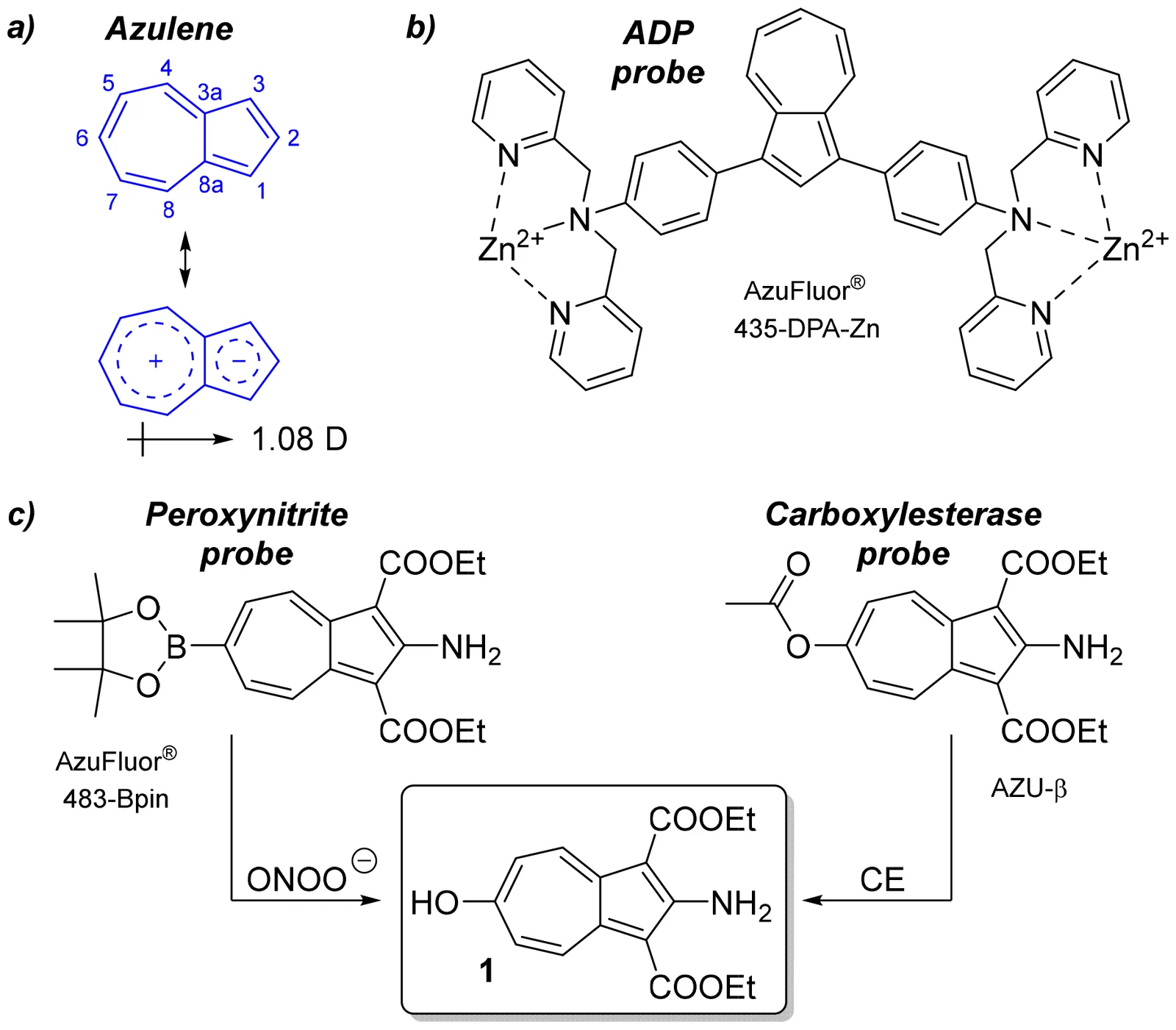
Structures of azulene and azulene-containing fluorescent probes. CE = carboxylesterase.

Fluorescent sensors that employ an azulene fluorophore are scarce.^14^ The first was reported in 2004 for the detection of fluoride,^15^ and various azulenes exhibit fluorescence changes in response to changes in pH (halofluorochromism).^16^ In the field of bioimaging, analyte-responsive azulene-containing probes have been reported by us for the detection of ADP (Fig. 1b),^17^ peroxynitrite^18^ and carboxylesterase^19^ (Fig. 1c) by two-photon fluorescence microscopy. Whereas the first of these is a chemosensor, showing fluorescence enhancement upon (reversible) binding of ADP, the latter two are both chemodosimeters (activity-based probes), undergoing irreversible reactions in the presence of their analytes leading to a fluorescence turn-on response. Specifically, peroxynitrite-mediated oxidative cleavage of the C–B bond in AzuFluor® 483-Bpin leads to formation of fluorescent hydroxyaminoazulene 1. Carboxylesterase-catalysed acetate cleavage in AZU-β leads to formation of the same product.

In contrast to substituted azulenes’ absorption maxima, the structure–property relationship between substituent pattern and electronic nature for fluorescence emission maxima and quantum yields is not as well understood. Spacing of energy levels is calculable and hence emission maxima may be predicted. However, many azulenes exhibit no fluorescence. For example, both AzuFluor® 483-Bpin and AZU-β show negligible fluorescence prior to undergoing reaction to form 1. Prediction of quantum yield is challenging, as this parameter encompasses the relative rates of the radiative and non-radiative processes occurring in a system. We therefore undertook the present study with the aim of systematically varying the structure of 1 through functional group exchange and/or deletion, in order to increase understanding of the structural requirements for azulene fluorophores.

As shown in Fig. 2, fluorophore 1 corresponds to an azulene bearing electron-withdrawing groups at C1 and C3, and electron-donating groups at C2 and C6. We surveyed the literature to identify relevant reports of structurally related azulenes with reported fluorescence (or lack thereof). An analogue of 1 lacking the C6-EDG is the well-known azulene 2, first synthesised by Nozoe^20^ and commonly employed as an entry to the azulene ring system. Fluorescence of 2 has been reported only in a solid matrix and with low quantum yield.^21^ An analogue of 1 lacking both the C2 and C6-EDGs is the azulene diester 3, which is essentially non-fluorescent.^22^ Recently Merkhatuly *et al.* have reported azulenes 4–10 bearing one or more EDG at C2, C6 or elsewhere, which all exhibit fluorescence in solution.^23–27^ Several of these also show intense absorbance in the visible region, which may be explicable through molecular orbital inversion.^28^ Emission maxima for 4–10 are shown in Fig. 2 and it can be seen that extension of π-conjugation leads to a bathochromic shift (9 and 10). Trends in quantum yield cannot be discerned as these are only reported for 7 and 8. Finally, Shoji *et al.* have reported azulenes of type 11, with a pyrrolidinyl C6-EDG, C1,3-EWG ester groups and a C2 aryl substituent.^29^ These all show solid-state fluorescence (for both electron-rich and electron-poor aryl substituents), but an analogue of 11 lacking the C2 substituent does not. In solution, compounds of type 11 show negligible fluorescence.

**Fig. 2:**
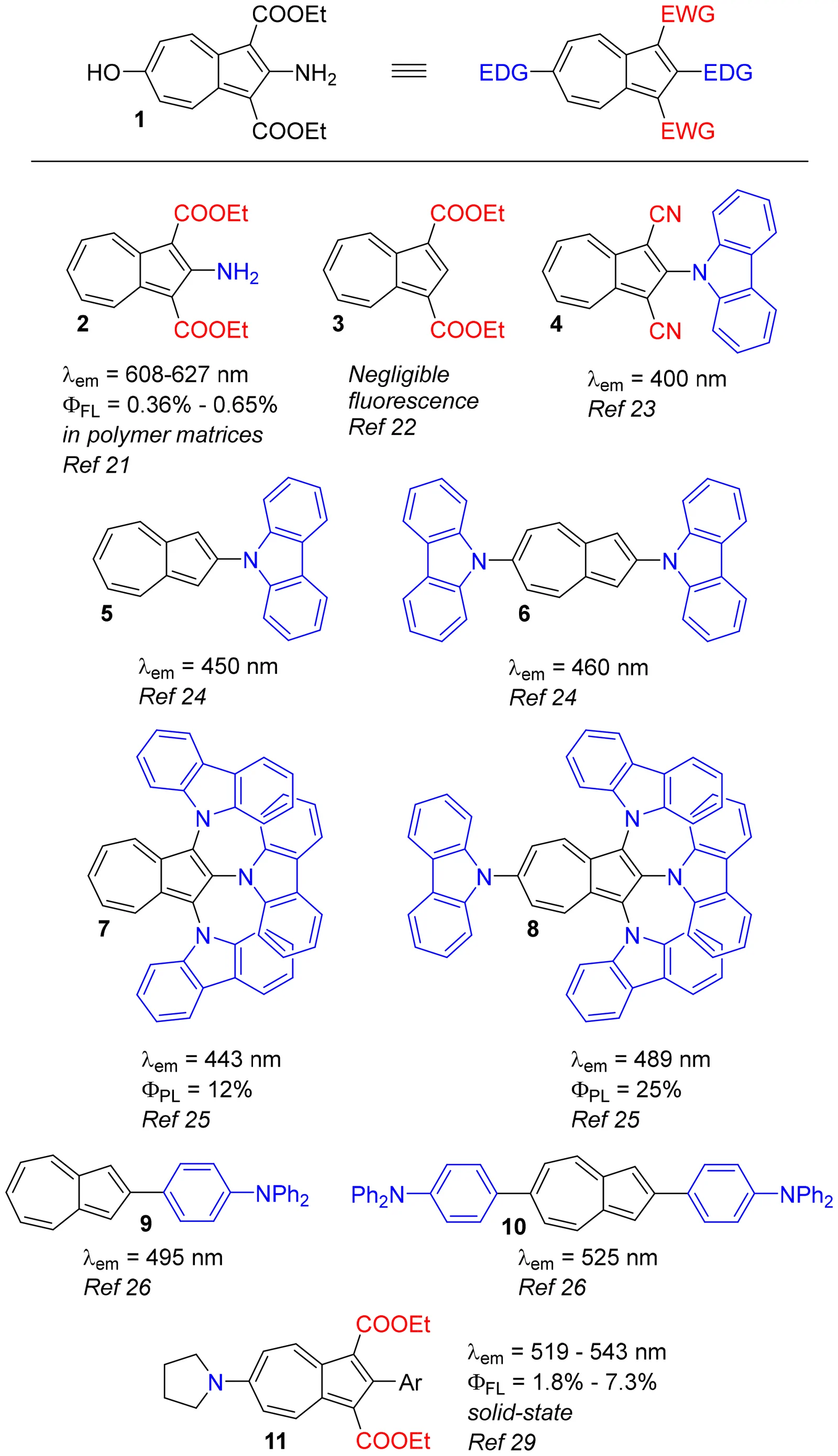
Fluorescence of certain reported azulenes. *Φ*_FL_ denotes reported fluorescence quantum yield; *Φ*_PL_ denotes reported photoluminescence quantum yield. EDG = electron-donating group. EWG = electron-withdrawing group.

## Results and discussion

### Synthesis

We aimed to prepare analogues of 1 for the purpose of elucidating structure–property relationships in (potential) fluorophores of this type. Specifically, we targeted analogues of 1 in which the C1 and C3 electron-withdrawing ester substituents were retained, but the C2 and C6 substituents were replaced with a range of *O*- or *N*-substituents (or removed entirely). Some of the targets are novel and some are reported in the historical literature (only) and hence are incompletely characterised; for these we report comprehensive characterisation data for the first time. The starting point in these syntheses was 12, prepared through straightforward bromination of Nozoe azulene 2.^30^ S_N_Ar reaction of 12 with sodium azide gave novel azidoazulene 13 cleanly (Scheme 1), followed by reduction with nickel boride^31^ to give 2,6-diaminoazulene 14 (*i.e.* the 6-amino analogue of 1). 14 has previously been characterised by UV/vis and IR spectroscopy only^32–34^ (its fluorescence properties were never studied).

**Scheme 1:**
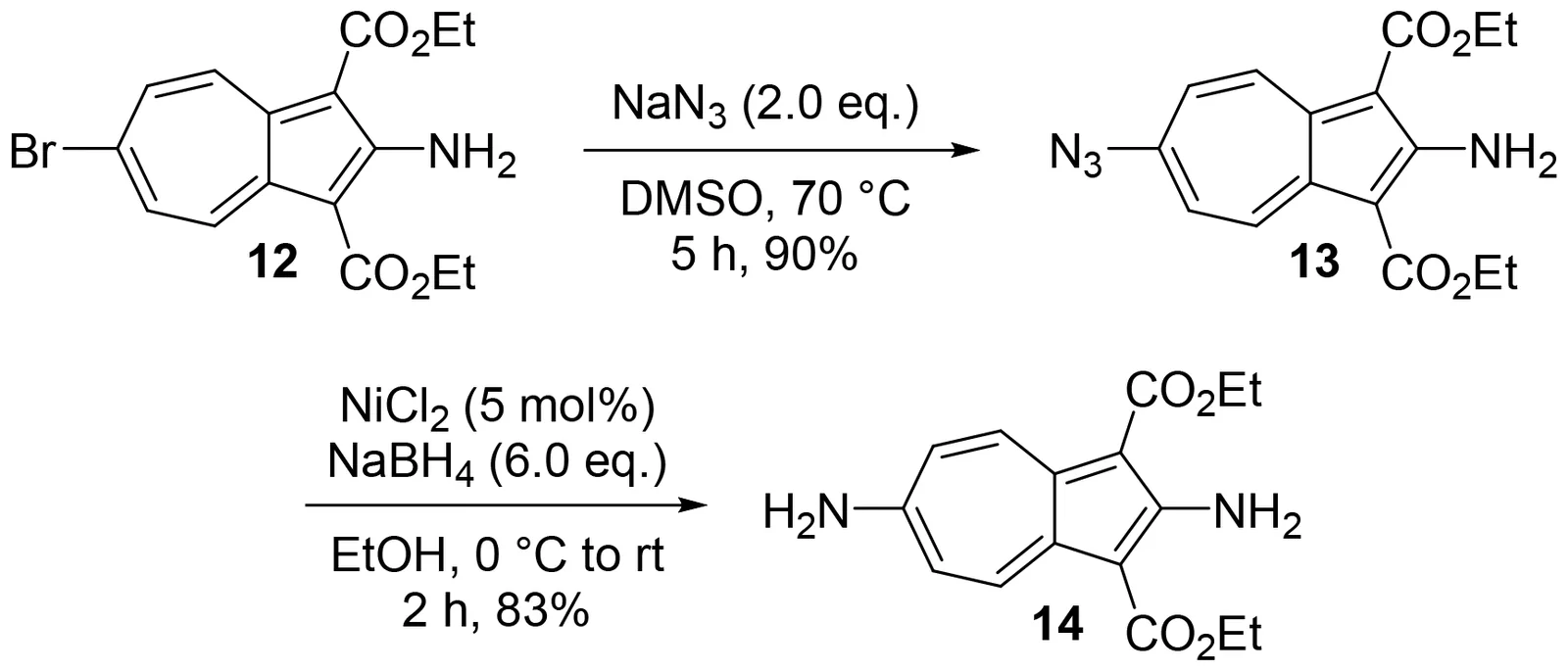
Two-step synthesis of 2,6-diaminoazulene 14.

Preliminary examination showed 14 exhibited fluorescence in solution, so we explored the introduction of other *N*-substituents at C6. Shoji *et al.* have previously reported the S_N_Ar reaction of 12 with cyclic secondary amines, employing solvent-free conditions (neat) in a sealed tube at 130 °C.^35^ We adapted this approach by employing solvent, as shown in Table 1. An initial attempt employed α-methylbenzylamine as nucleophile in DMF with microwave acceleration (entry 1), but side product 15a was the major product. We ascribe this result to the formation of dimethylamine from decomposition of DMF under microwave irradiation.^36^ DMSO proved to be a much more satisfactory solvent, giving 6-(1-piperidinyl)azulene 15b in good yield at a temperature 50 °C lower than the previously reported procedure^35^ (entry 2). Comparable yields were obtained for 6-(4-morpholinyl)azulene 15c and 6-(1-pyrrolidinyl)azulene 15d under the same conditions (entries 3 and 4). The low boiling point of azetidine meant that for the synthesis of novel 6-(1-azetidinyl)azulene 15e the reaction temperature needed to be lowered further, but even at 50 °C reaction with this nucleophile proceeded in only slightly attenuated yield (entry 5). However, primary amine nucleophiles bearing α-branching proved less reactive. Treatment of 12 with (*R*)-α-methylbenzylamine in DMSO at 80 °C resulted in only minimal conversion, and an increase to 130 °C was required to provide product (*R*)-15f in good yield (entry 6); enantiomeric (*S*)-15f was also synthesised (entry 7). Another nucleophile comprising a stereocentre was used in the preparation of (*S*)-15g (entry 8). Finally, sodium cyanate was employed in an attempt to prepare 6-(iso)cyanato product(s) (entry 9), but only 14 was isolated, presumably due to formation of the desired 6-isocyanatoazulene followed by addition of water and decarboxylation.

**Table 1:** Reaction of 6-bromoazulene 12 with various nucleophiles

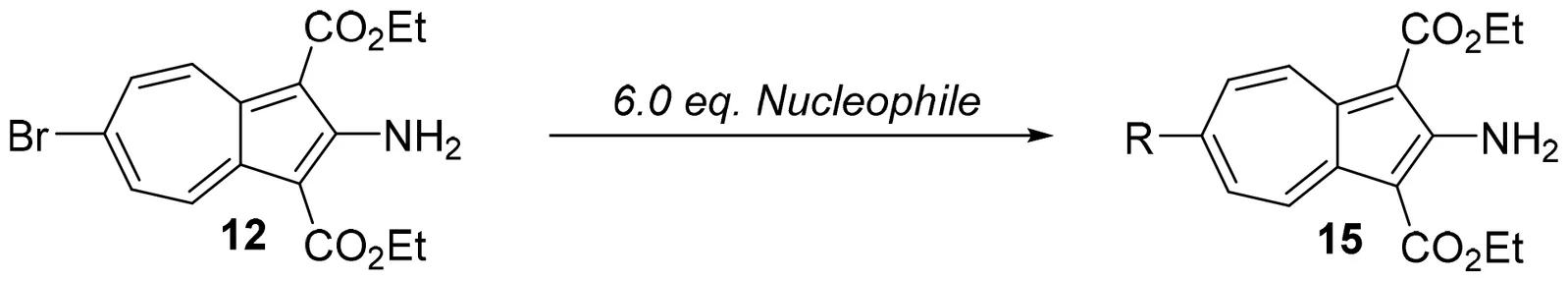				
Product	Solvent	Temperature, time	*R*	Yield
15a^a^	DMF	130 °C, µw	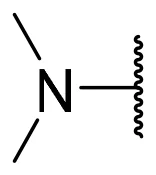	67%
		200 W, 3 h		
15b	DMSO	80 °C, 17 h	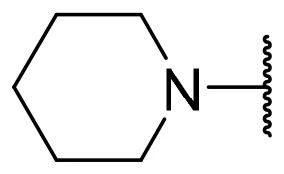	75%
15c	DMSO	80 °C, 16 h	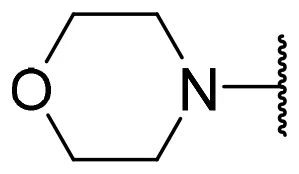	70%
15d	DMSO	80 °C, 16 h	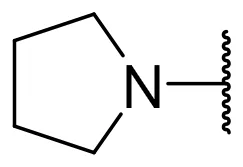	67%
15e	DMSO	50 °C, 17 h	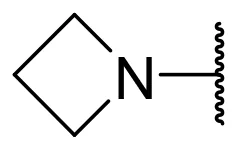	54%
(*R*)-15f	DMSO	80 °C, 40 h, then 130 °C, 21 h	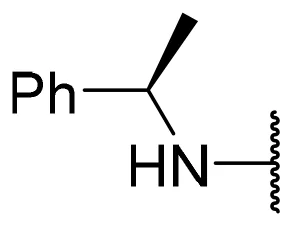	63%
(*S*)-15f	DMSO	130 °C, 63 h	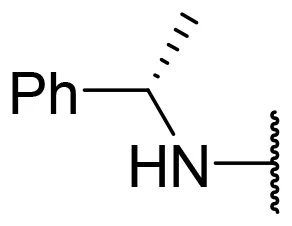	59%
(*S*)-15g	DMSO	130 °C, 16 h	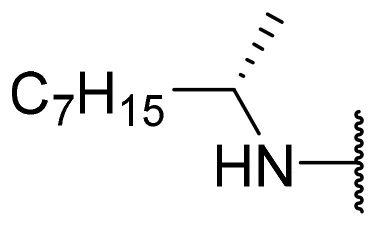	50%
14^b^	DMSO	130 °C, 47 h	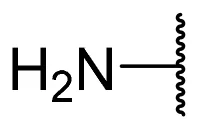	20%

a Microwave reaction; (*S*)-α-methylbenzylamine was used as the nucleophile; 9% of (*S*)-15f was also isolated.

b NaOCN was used as the nucleophile.

Products 14 and 15a–e gave crystals suitable for X-ray diffraction and the structures so obtained are shown in Fig. 3. The structure of 15e is notable for the seemingly perfect coplanarity of the azetidine and azulene rings and trigonal planar geometry at nitrogen (torsion angles of 0°; sum of bond angles at nitrogen ∑*θ* = 360°). 15a and 15d are also nearly coplanar (torsion angles <6°; ∑*θ* = 360°). In contrast, 15b exhibits torsion angles of 10.7(2)° and 43.9(2)°, with slight pyramidalization at nitrogen (∑*θ* = 353.8°). For 15c, disorder in the morpholine ring precludes a precise analysis.

**Fig. 3:**
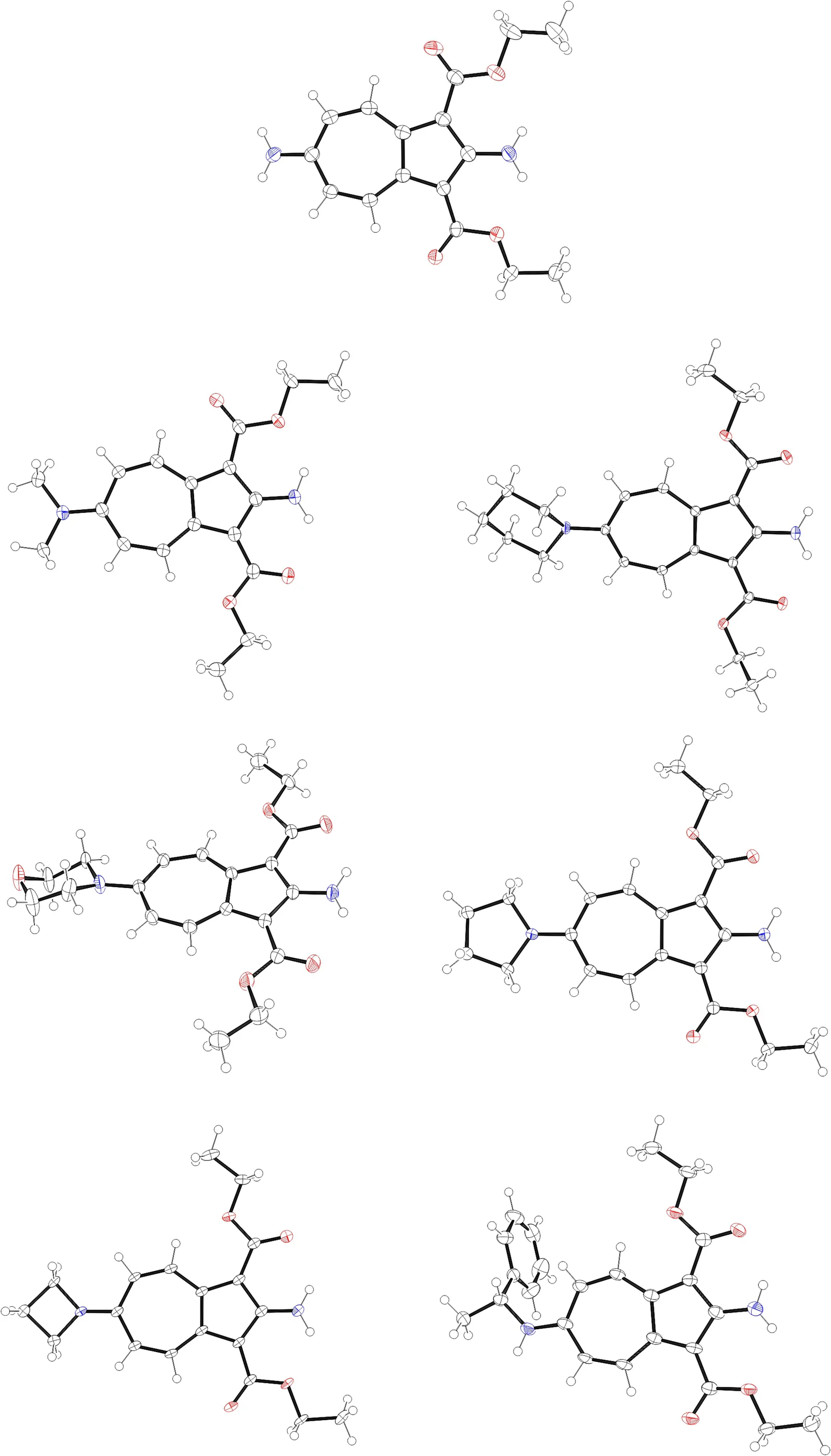
ORTEP images of 14 (CCDC 2566798), 15a (CCDC 2566799), 15b (CCDC 2566800), 15c (CCDC 2566801), 15d (CCDC 2566802), 15e (CCDC 2566803) and (*R*)-15f (CCDC 2566804). Ellipsoids are shown at 50% probability. Hydrogens are shown as spheres of arbitrary radius. Disorder in the morpholine ring of 15c and in one ethyl group of 15e has been omitted for clarity.

In structures 15a–g there is no steric impediment to conjugation of the C2 amine lone pair with the azulene ring. In contrast, the azulenes of type 11 having an aryl substituent at C2 cannot achieve coplanarity due to steric clash between the C2 aryl group and the ester groups at C1 and C3. Rather, they adopt a near-perpendicular arrangement of the two aryl rings in the solid state (biaryl angles >70°).^29^ Given the reported fluorescence of azulenes of type 11, we next aimed to synthesise variants of 14 in which conjugation of the C2 amine lone pair with the azulene ring is modulated by substitution on the C2 nitrogen (and distortion from planarity). The C2 amino group in both 2 and 12 is only poorly nucleophilic. Hence, as shown in Scheme 2, prolonged reflux in acetic anhydride was required to effect acetylation of 12, giving both amide 16 and imide 17.^34^ Treatment of 16 with sodium azide gave novel azidoazulene 18, analogously with 13. However, an initial attempt at effecting the same substitution on imide 17 unexpectedly gave 18, as a result of monodeacetylation during purification by manual column on basic alumina. A subsequent attempt featuring a purification by rapid filtration through silica with crude material dry-loaded gave novel 19 as expected. Finally, reduction of 18 gave 6-amino-2-amidoazulene 20,^34^ and reduction of 19 gave novel 6-amino-2-imidoazulene 21.

**Scheme 2:**
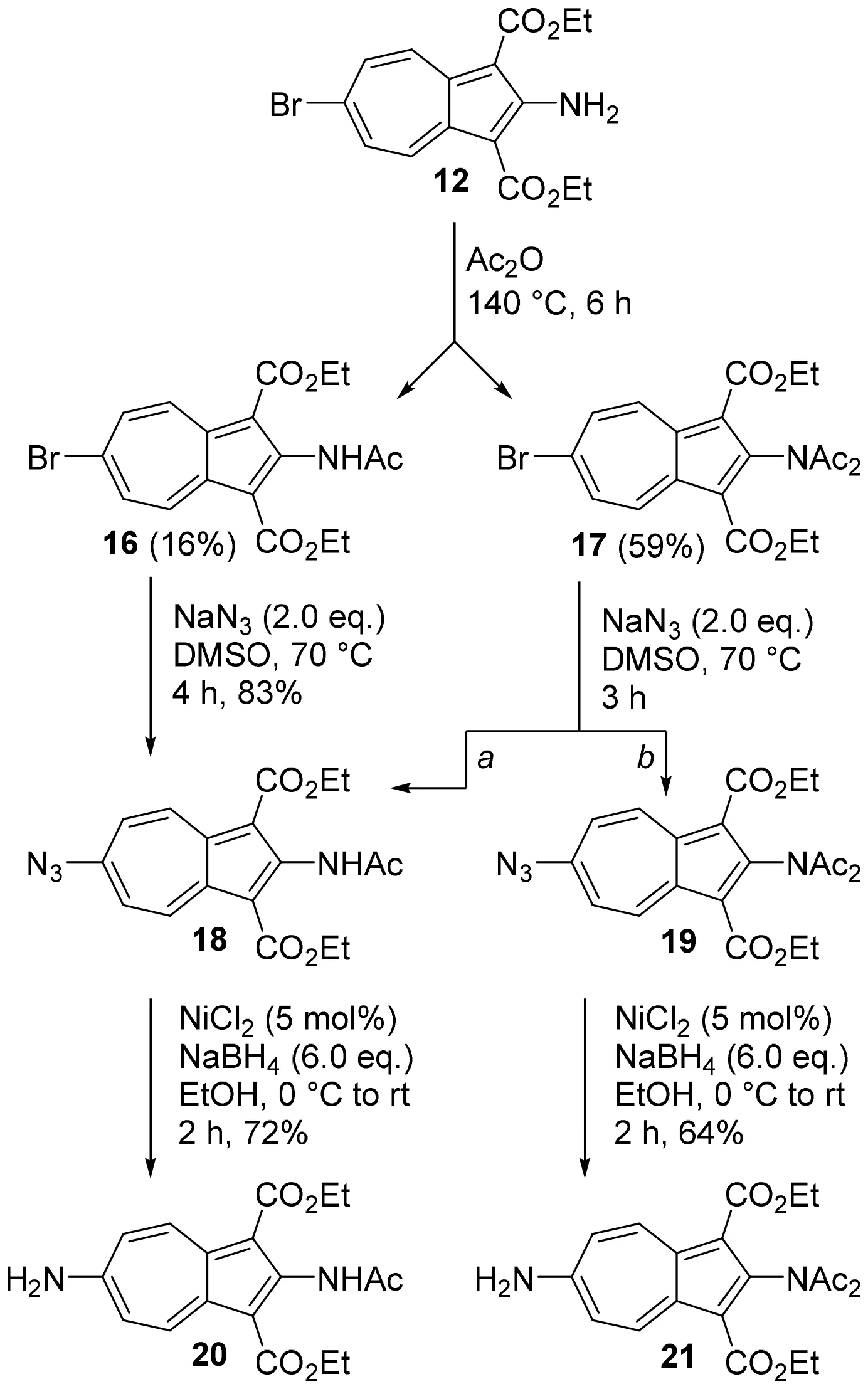
Synthesis of singly and doubly acetylated analogues of 14. ^*a*^ Alumina chromatography; 62%. ^*b*^ Silica chromatography; crude dry-loaded on Celite; 85%.

Compounds 16, 17 and 18 gave crystals suitable for X-ray diffraction and the structures so obtained are shown in Fig. 4. In singly *N*-substituted 16, the amide is partially distorted away from coplanarity with the azulene ring, with the C–C–N–C torsion angle = 33.6(6)°. This value is appreciably lower than the biaryl torsion angles above 70° reported for 11. Instead, in 16 part of the steric clash between the C2 *N*-substituent and the adjacent groups is accommodated by one of the esters also distorting away from planarity. Thus, the ester adjacent to the amide oxygen has a C–C–C=O torsion angle = 45.7(6)°, whereas the other ester remains coplanar with the azulene ring. In doubly *N*-substituted 17, the conformation is different, with the C2 imide substituent perpendicular to the azulene ring, having C–C–N–C torsion angles = 87.8(5)° and 89.2(5)°. Both ester groups are near coplanar with the azulene ring. The situation with 18 is very similar to 16, with the C–C–N–C torsion angle = 33.7(4)°, the adjacent ester C–C–C=O torsion angle = 36.9(4)° and the other ester coplanar with the azulene ring. The conformational preferences of *N*-azulenylamides have been extensively studied by Okamoto *et al.*^37^ both in the solid state and in solution. Trends were reported relating the nature of the *N*-azulenyl substituent and the other *N*-substituent (if any) to the torsion angle between the azulene substituent and the amide oxygen (synperiplanar or antiperiplanar). However, the Okamoto group did not study any *N*-azulenylimides and in this context it is particularly interesting to note that in 17 one imide carbonyl is synperiplanar with the azulene, but the other is antiperiplanar.

**Fig. 4:**
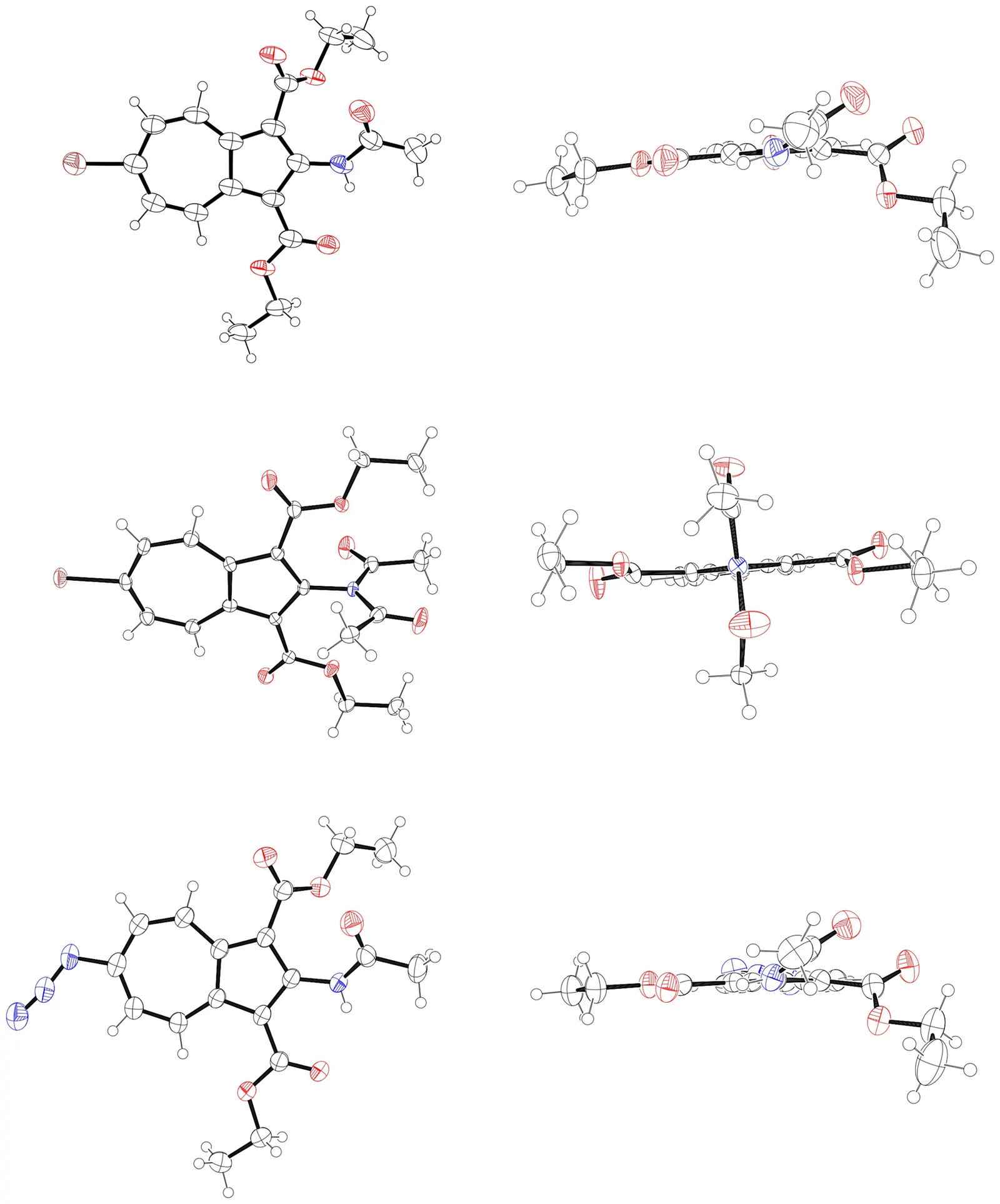
ORTEP images of 16 (top, CCDC 2566805), 17 (middle, CCDC 2566806) and 18 (bottom, CCDC 2566807). Ellipsoids are shown at 50% probability. Hydrogens are shown as spheres of arbitrary radius. Only one of two independent molecules in the unit cell of 16 is shown.

We next sought to prepare an analogue of 14 with substituted amino groups at both C2 and C6. As per the procedure of Barybin *et al.*, 12 underwent C2-diazotization/chloride substitution as well as C6 halide exchange by S_N_Ar to give 22 in 1 step (Scheme 3).^38^ Next, attempted double S_N_Ar reaction with excess pyrrolidine did not give desired 24, but instead furnished monosubstitution product 23 only. The analogue of 23 which lacks the C6 pyrrolidine substituent is reported to undergo S_N_Ar reactions at C2.^39^ Hence, we ascribe the lack of formation of 24 to initial attack on 22 occurring at the more electrophilic site (C6), but then the newly introduced electron-donating C6-pyrrolidino substituent in 23 rendering C2 less electrophilic, such that the second substitution does not occur under these conditions. We confirmed that the S_N_Ar reaction on 22 occurred at C6 (and not at C2) through the observation of a NOESY interaction between azulene H5/7 and pyrrolidine Cα-H in 23 (see SI). The failure to form 24 from 23 is in contrast to the reported successful formation of 24 from the 2-bromo analogue of 23, albeit under more forcing conditions in the latter case.^40^

**Scheme 3:**
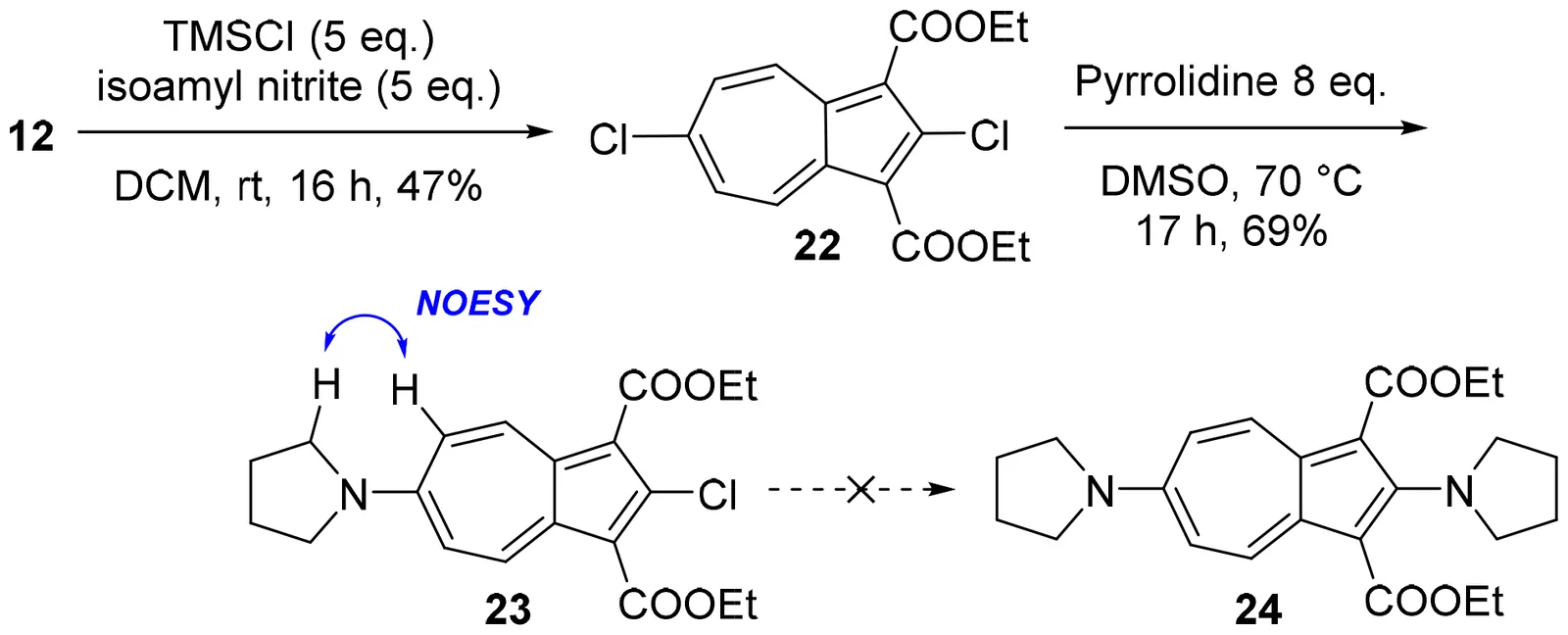
Attempted synthesis of 2,6-bis(pyrrolidino)azulene 24.

A different substitution pattern, not previously explored in the context of azulene fluorescence is that in which the C2-EDG in 1 is removed, leaving a C6-EDG and the C1,3 ester substituents. To access such a substitution pattern, 12 was diazotised and reduced with *p*-hydroquinone to give 25 (Scheme 4).^41^ S_N_Ar reaction of 25 with potassium acetate in DMSO (followed by hydrolysis) proceeded in good yield, thereby establishing a new route to 6-hydroxyazulene 26 (which had previously been synthesised by vicarious nucleophilic substitution of 3 with *tert*-butyl hydroperoxide in liquid ammonia^42^). To access the amino variant, 25 was treated with sodium azide to give 27,^16*a*^ which was reduced to 28^43^ (this had also previously been synthesised by vicarious nucleophilic substitution of 3 ^44^). Finally, we sought to access 32,^45^ the isomer of 1 where the amino and hydroxyl groups are transposed. For this we employed 6-bromo-2-hydroxyazulene 29,^46^ which underwent S_N_Ar to afford C6-azidoazulene 30. An alternative two-step route involving acetylation of 29 to give 31, followed by azide introduction with concomitant acetate cleavage to give 30, did not give an improved overall yield. As 30 proved to be moderately unstable, the crude material was immediately subjected to reduction to give 32.

**Scheme 4:**
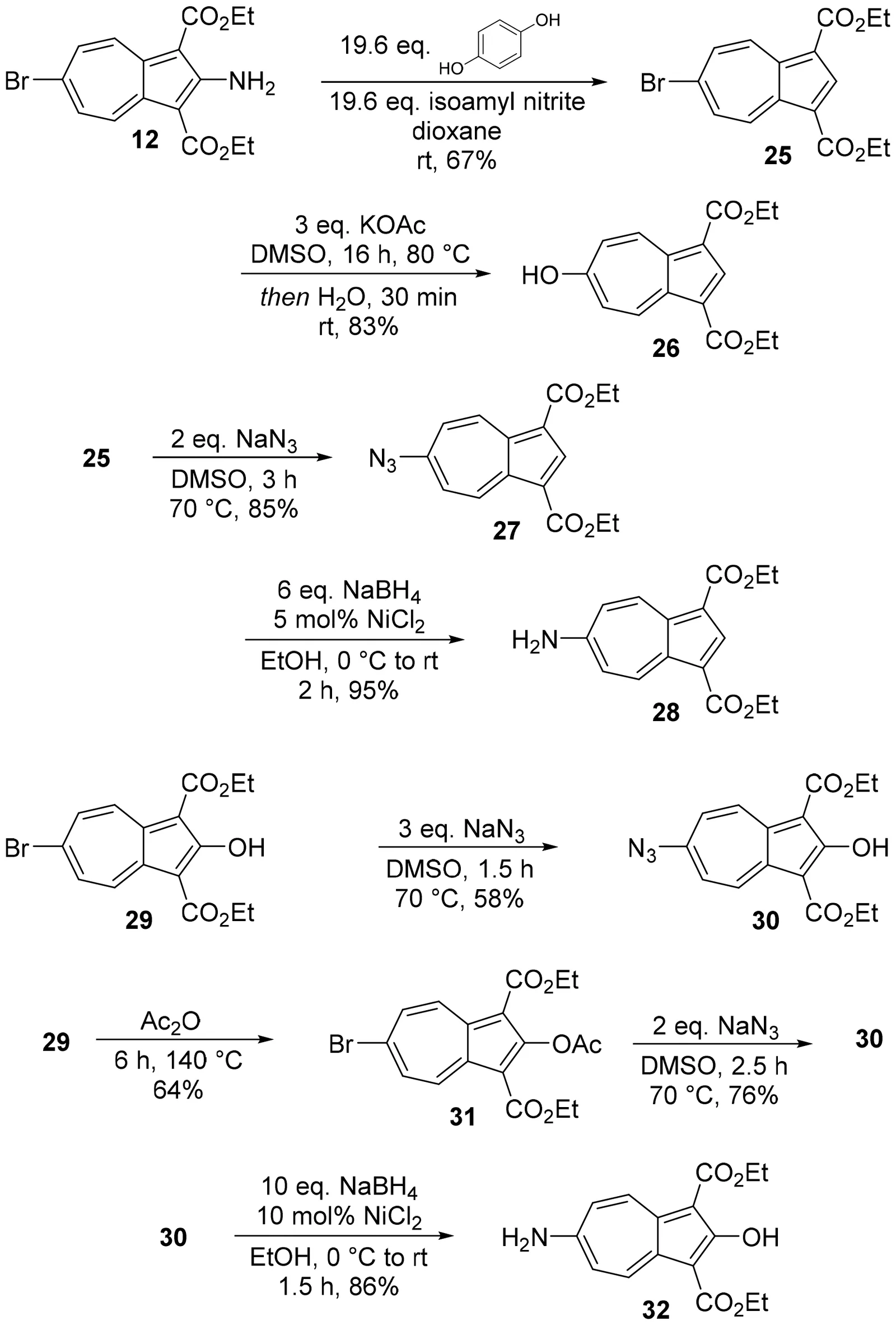
Synthesis of azulenes with a C2-*O*-substituent or no C2 substituent.

Compounds 28 and 31 gave crystals suitable for X-ray diffraction and the structures so obtained are shown in Fig. 5. The conformation adopted by ester 31 in the solid state merits comparison with its amido analogue 16 (Fig. 4). Whereas in the case of 16 the steric clash is mitigated by both the amido and an ester group reorienting away from planarity, in the case of 31 it is the acetoxy group alone which is oriented out of plane, with C–C–O–C torsion angles of 93.7(4)° and 94.2(4)°. The ethyl esters remain coplanar with the azulene ring (largest torsion angle of 8.7(5)°). As per the analyses of Okamoto, the azulene and the acetate ester carbonyl oxygen are synperiplanar.

**Fig. 5:**
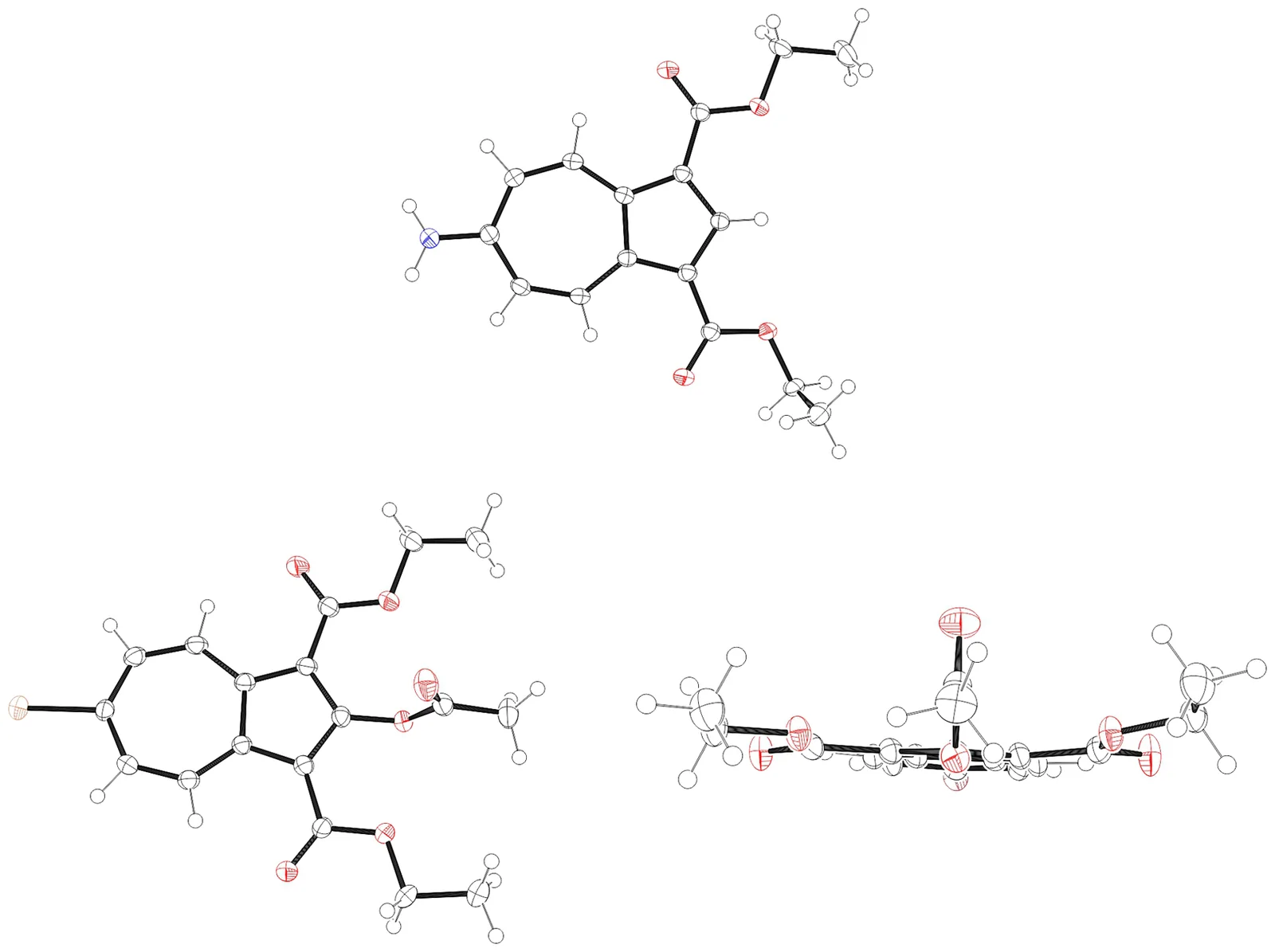
ORTEP images of 28 (top, CCDC 2566808) and 31 (bottom, CCDC 2566809). Ellipsoids are shown at 50% probability. Hydrogens are shown as spheres of arbitrary radius.

Of the compounds reported here, most were stable to chromatographic purification on silica or alumina. The main exception was 30 (which could not be chromatographed on any stationary phase). Compounds 18 and 19 required specific chromatographic conditions to carry out their purification (as shown in Scheme 2), and compounds 13 and 27 were purified by rapid filtration through a plug of alumina; 13 was unstable in solution. These compounds all possess a 6-azido group; we note that other reported 6-azidoazulenes are purified by methods that avoid chromatography (*e.g.* recrystallisation).^45,47^

### Photophysical characterisation

Fluorescence emission spectra were acquired for selected compounds (1, 14, 15a–g, 20, 21, 23, 26, 28 and 32) and the normalised emission spectra are shown in Fig. 6. These were acquired at a common excitation wavelength (350 nm) to facilitate comparison between them. Fluorescence quantum yields for these compounds were measured using an integrating sphere and the values are presented in Table 2, along with selected UV/vis absorption maxima and calculated Stokes shifts (For UV/vis spectra see SI).

**Fig. 6:**
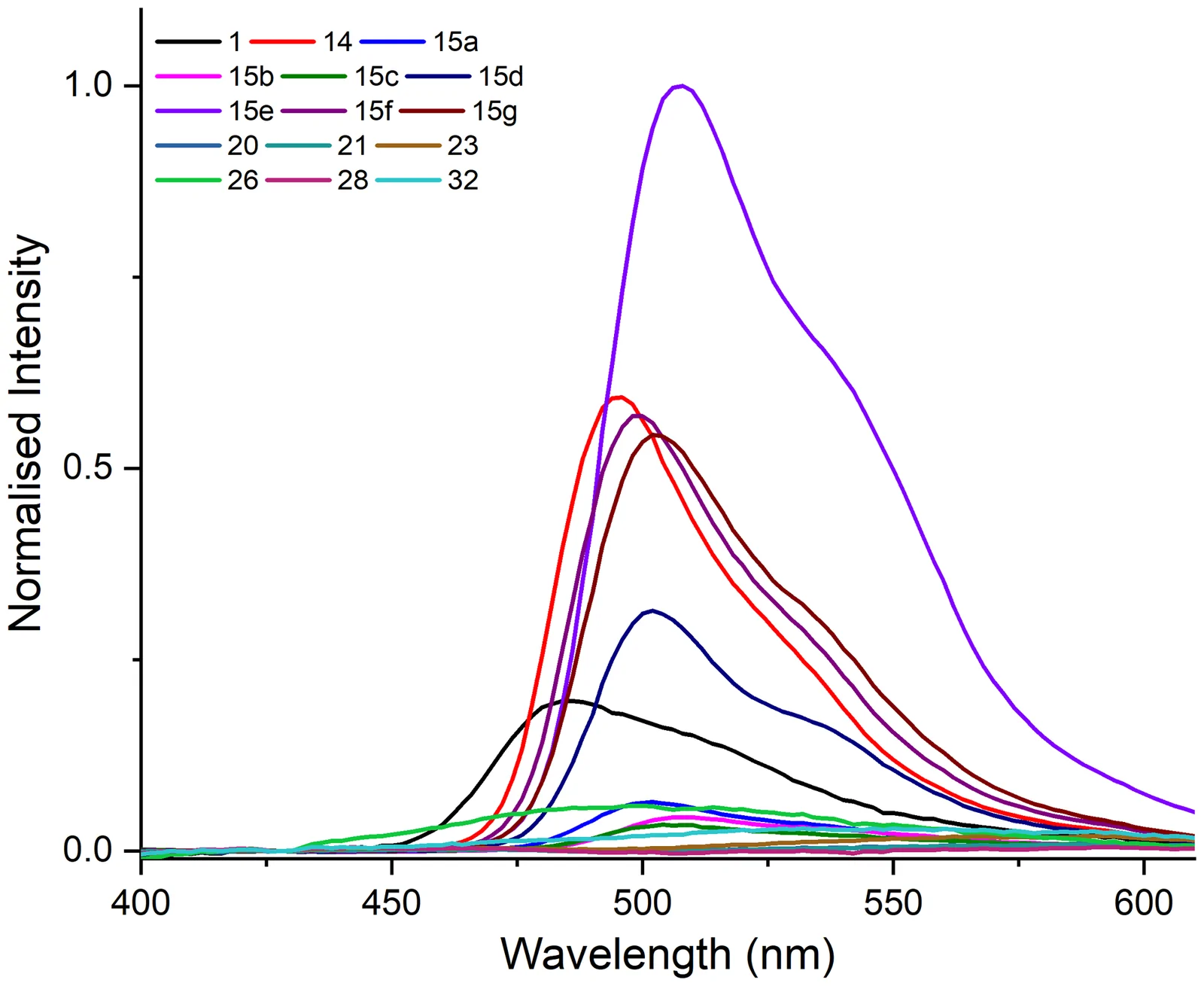
Normalised fluorescence emission spectra. Spectra were acquired as 5 µM solutions in 99.5% CH_3_CN/0.5% DI water, v/v, with *λ*_ex_ = 350 nm.

**Table 2:** Selected absorption maxima, fluorescence quantum yields, emission maxima and Stokes shifts

Compound	*λ*_abs _ a (nm)	*Φ*_FL _ b (%)	*λ*_em_ (nm)	Stokes shift^c^ (nm)
14	344, 477	12.1	496	19
15a	351, 477	1.3	506	29
15b	353, 479	0.9	508	(29)
15c	348, 479	0.7	508	(29)
15d	353, 479	6.4	502	23
15e	353, 479	20.4	508	29
15f	350, 477	11.6	500	23
15g	350, 479	11.1	502	23
20	336, 396	0.2	508	(112)
21	332, 410	0.2	508	(96)
23	340, 423	0.4	508	(85)
26	317, 373	1.2	508	135
28	334, 423	0.1	508	(85)
32	337, 421	0.6	508	(87)

a Absorption maxima given are the most intense and the longest wavelength.

b Quantum yields were calculated by integrating the emission peaks 450–610 nm.

c Figures in parentheses for compounds with *Φ*_FL_ < 1%.

The fluorescence of the compound with the highest quantum yield (15e) was then evaluated in a variety of solvents. As shown in Fig. 7 and Table 3, *Φ*_FL_ varies significantly between solvents, although no correlation between quantum yield and solvent dielectric constant is discernible. Compound 15e does not exhibit significant solvatofluorochromism, with *λ*_em_ only varying between 508 and 502 nm.

**Fig. 7:**
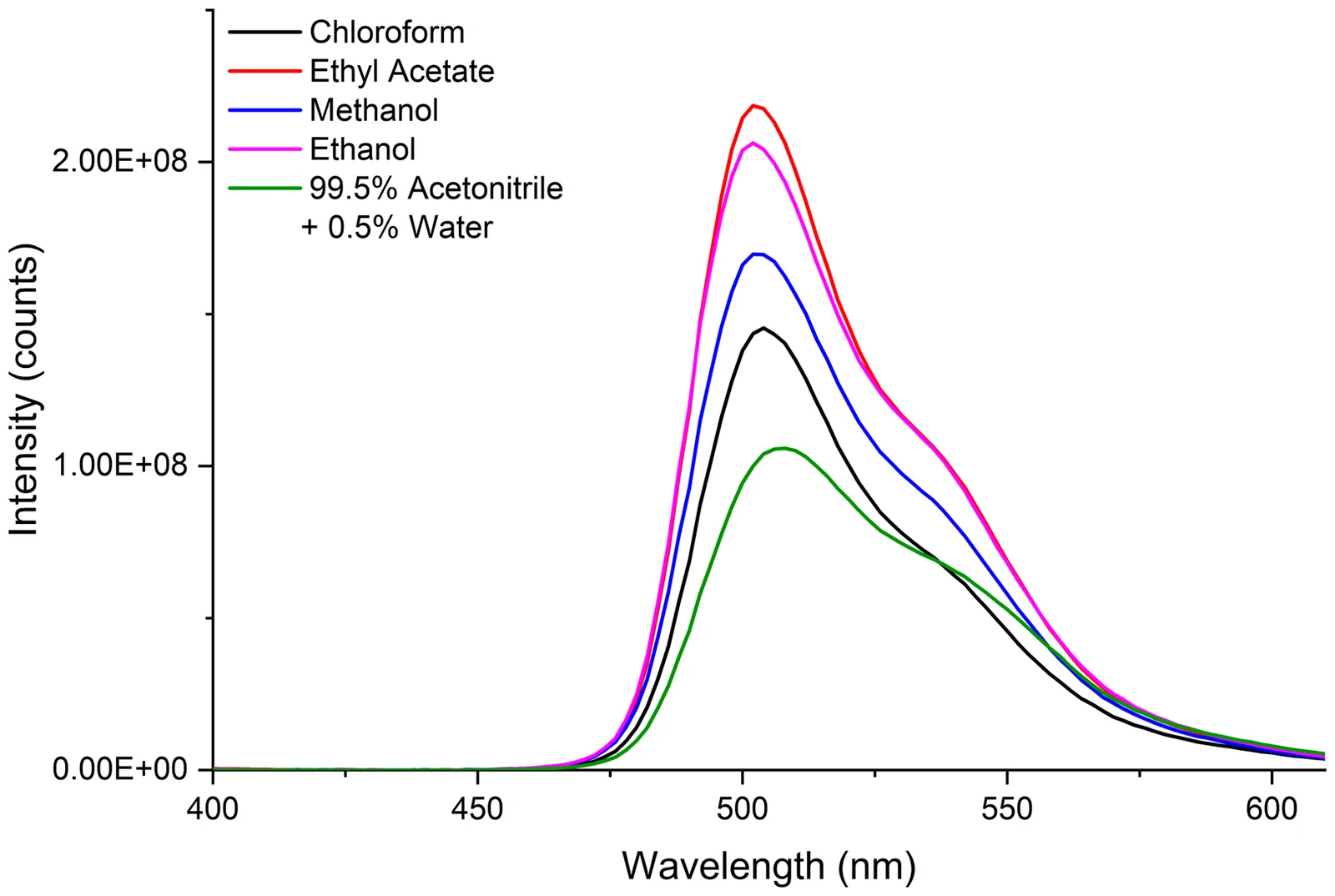
Normalised fluorescence emission spectra for 15e. Spectra were acquired as 5 µM solutions in various solvents, with *λ*_ex_ = 350 nm.

**Table 3:** Fluorescence quantum yields for 15e in various solvents

Solvent	*Φ*_FL _ a (%)
Ethyl acetate	41.7
Ethanol	37.3
Methanol	32.6
Chloroform	26.2
99.5% CH_3_CN/0.5% water	20.4

a Quantum yields were calculated by integrating the emission peaks 450–610 nm.

We next studied whether selected compounds displayed changes in fluorescence in response to changes in pH (*i.e.* halofluorochromism). Our previously reported fluorophore 1 contains functional groups that could be protonated (2-amino) or deprotonated (6-hydroxy). Treatment of 1 with *N*,*N*-diisopropylethylamine (DIPEA) led to a 2.6-fold increase in fluorescence intensity in the presence of 10 equivalents of base (Fig. 8).

**Fig. 8:**
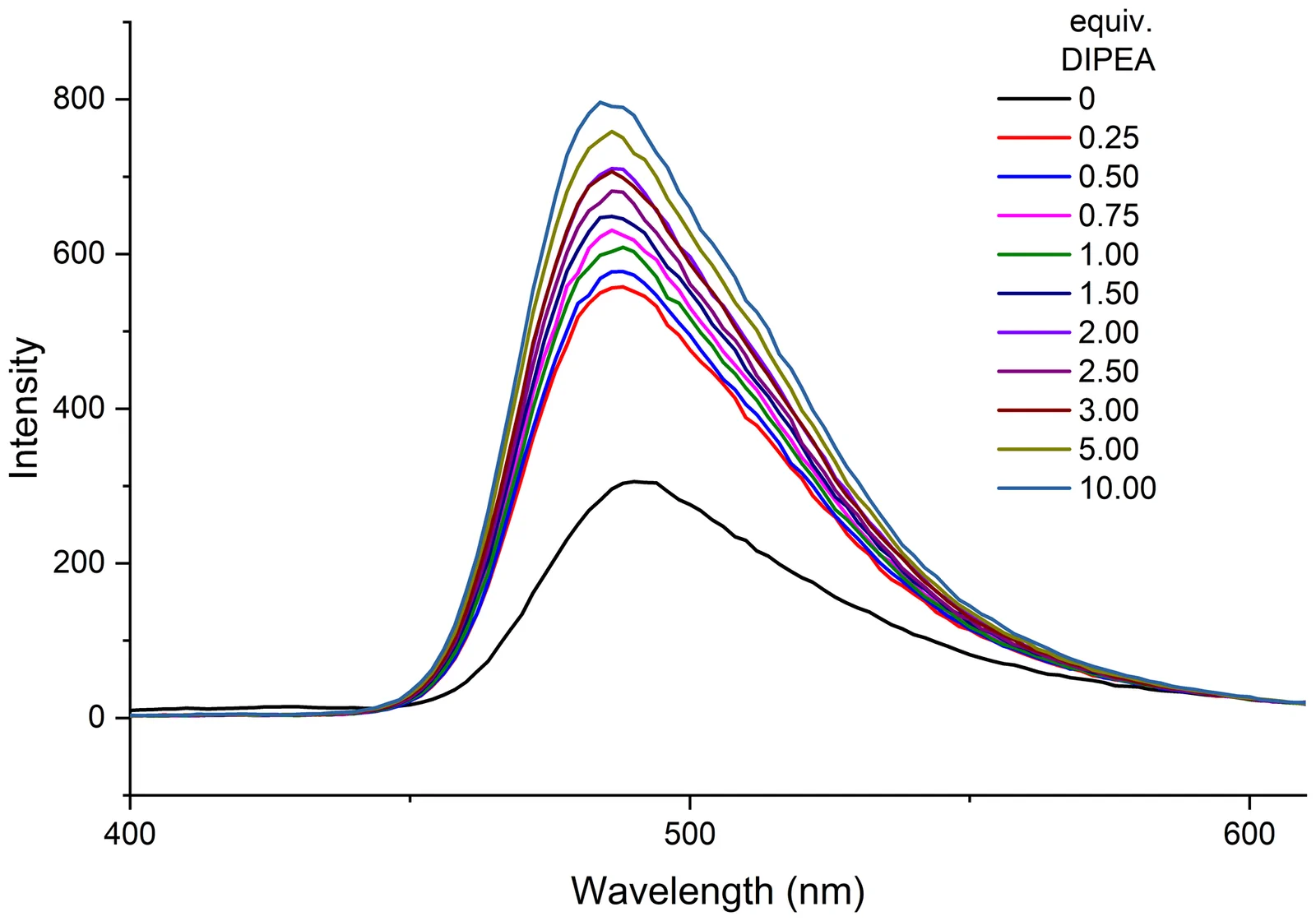
Fluorescence spectra of 1 (10 µM) in neutral conditions and with increasing quantities of DIPEA. Experimental conditions: 99.5% acetonitrile – 0.5% water at room temperature (25 °C). Fluorescence intensities were measured with *λ*_ex_ = 335 nm (ex. slit: 5 nm, em. slit: 10 nm).

In contrast, treatment of 1 with formic acid led to a maximum 1.7-fold increase in fluorescence intensity with 1 equivalent of acid; further equivalents of acid led to a reduction in fluorescence intensity, such that after addition of 10 equivalents the intensity had returned to the level of pure 1 alone (see SI). Fluorescence of the 6-hydroxyazulene 26 was also evaluated in the presence of various equivalents of DIPEA and a more modest maximal 1.3-fold increase in intensity was observed (see SI). Finally, the 2,6-diaminoazulene 14 was selected for evaluation as a representative example of the 6-amino series of compounds (14 and 15a–g). As this fluorophore lacks basic hydroxyl groups, only its response to acid was assessed. In the event, only a minimal change in fluorescence intensity (up to a 1.1-fold increase) was observed with formic acid (see SI).

The chiral azulenes 15f and 15g were characterised by circular dichroism spectropolarimetry. As expected, the electronic transitions associated with the azulene chromophore display a Cotton effect commensurate with a directly connected stereogenic centre. The two enantiomers of 15f show mirror image spectra with intense bisignate transitions in the UV region (Fig. 9 and SI).

**Fig. 9:**
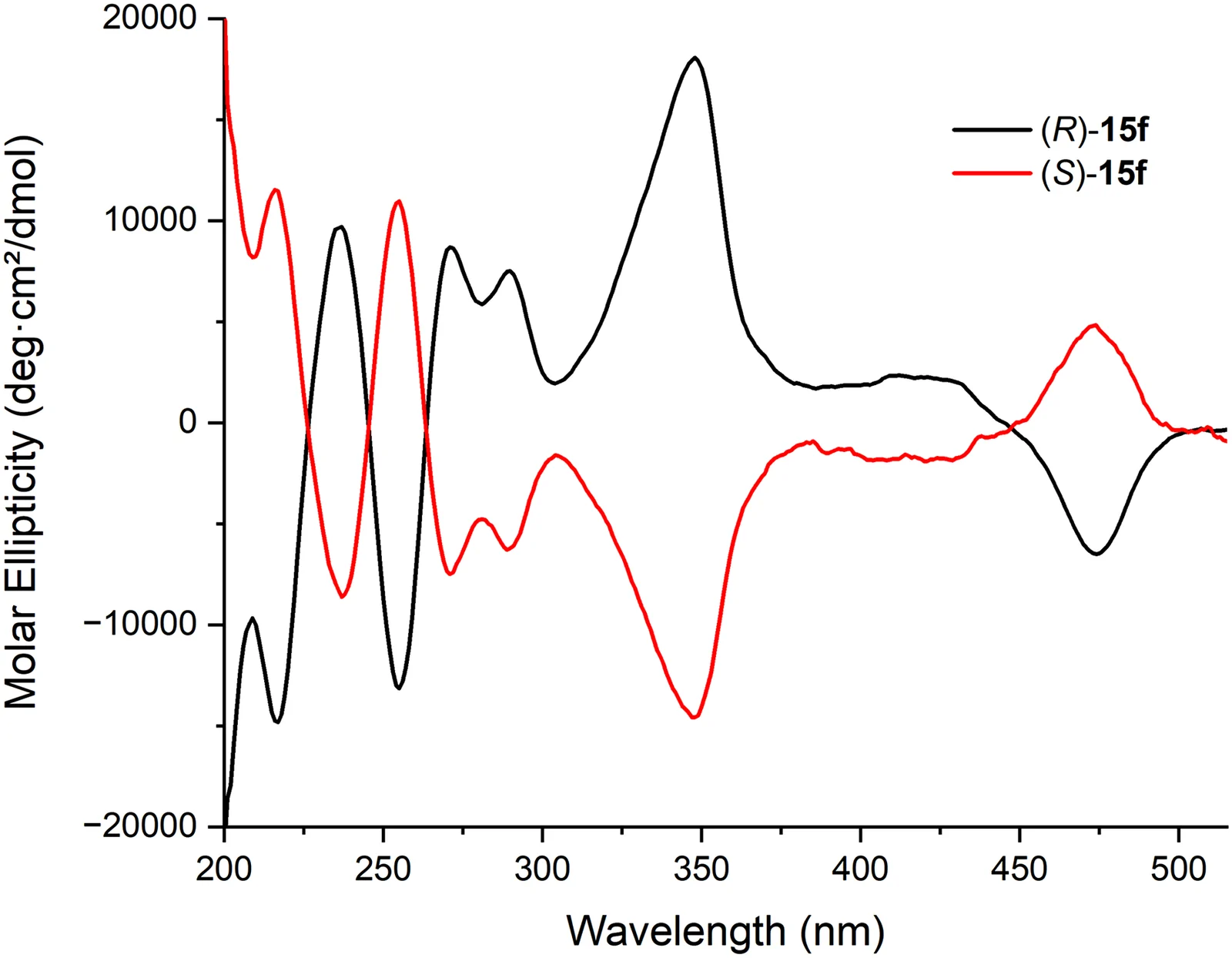
CD spectra of the enantiomers of 15f (CH_3_CN solution).

We previously reported 1 as the fluorophore in our turn-on activity-based probes AzuFluor® 483-Bpin and AZU-β (Fig. 1) and report its fluorescence characteristics here, to enable comparison with the other compounds, for which fluorescence data are being reported for the first time. We previously reported^18^ a value of *Φ*_FL_ = 1.0% for 1, but this value was acquired under different conditions (PBS/MeOH 48 : 52, pH = 8.2; *λ*_ex_ = 340 nm) and by a different method.^48^ Under the conditions in this present work, we measured *Φ*_FL_ = 4.0% for 1, and it is this value that we compare to the those for the new (prospective) fluorophores in this work. When such comparisons are made, several trends are evident.

Firstly, *the presence of a highly electron-donating C2-substituent appears to be essential for achieving a high quantum yield* in this series of compounds. In the original fluorophore 1, this requirement is fulfilled by the C2-amino group, and all the fluorophores that have a greater quantum yield than 1 (*i.e.*14 and 15d–g), also have a C2-amino substituent. On the other hand, the compounds *lacking* a C2-amino substituent (*i.e.*20, 21, 23, 26, 28 and 32) all exhibit lower quantum yields than 1. Thus, the compounds that lack *any* C2-substituent (26 and 28) show only minimal (26) or negligible (28) fluorescence. Those bearing a less electron-donating heteroatom at C2, *i.e.*32 (C2 oxygen; more electronegative) and 23 (C2 chlorine; more electronegative and poor orbital overlap) also show minimal fluorescence. The case of 20 and 21 is particularly interesting as they do possess a (substituted) nitrogen at C2 but show negligible fluorescence. We rationalise this in terms of the carbonyl substituent(s) at nitrogen. The amido (20) or imido (21) substituents are inherently less electron-releasing from nitrogen into the ring. Moreover, the X-ray structures of the precursors to 20 and 21 (*i.e.*16–18, see Fig. 4) clearly show significant distortion away from planarity around the C2–N bond, due to steric clash between the C2 *N*-substituents and the ester groups. By analogy the same effect is expected to be operative in 20 and 21 (both in the solid state and in solution) and will further attenuate the electron-donating ability of the C2 *N*-substituent.

Secondly, *a C6-amino substituent appears to be beneficial for higher quantum yield*. A direct comparison of 1 with its 6-amino analogue 14 shows the introduction of the C6-amine to lead to a 3-fold increase in quantum yield. The initially observed fluorescence of 14 led us to synthesise 15a–g and it can be seen that the choice of substituents on the C6-amine profoundly affects the quantum yield, which ranges from 0.7% to 20.4%. The model previously proposed for the fluorescence of 1 was that the C6-hydroxyl group served as a donor group in a D–π–A fluorophore in which the esters at the 1- and 3-positions served as the acceptors. Under this model, replacement of the C6-hydroxyl with a more effective π–donor group such as an amine would potentially be expected to lead to a larger Stokes shift. Bathochromic shifts up to 22 nm are observed for the emission maxima of 14 and 15a–g, in comparison to 1. Variation of the C6-donor group would also be expected to result in variation in the quantum yield, in the broadest sense.

Thirdly, *a C6-azetidino substituent imparts a clear increase in quantum yield*, with 15e showing more than a 5-fold increase in *Φ*_FL_ compared to 1. For D–π–A fluorophores such as those in this present study, formation of a twisted intramolecular charge-transfer (TICT) state represents a potential mechanism of fluorescence quenching.^49^ Upon excitation to the locally excited (LE) state, the C6–N bond may rotate away from planarity to access the TICT state, that can undergo non-radiative relaxation, thereby providing a pathway for non-emissive return to the ground state, and hence a lower quantum yield. We propose that the wide range of quantum yields observed for 14 and 15a–g is due (in part) to the varying extent to which these species can access the TICT state upon excitation. Various strategies have been developed to suppress TICT, such as rigidification through annulation, modulation of steric hindrance or employing inductive effects.^50^ In fluorophores where the donor motif is an amine, use of an azetidine to suppress TICT is a precedented strategy.^51^ The trends described above are represented pictorially in Fig. 10.

**Fig. 10:**
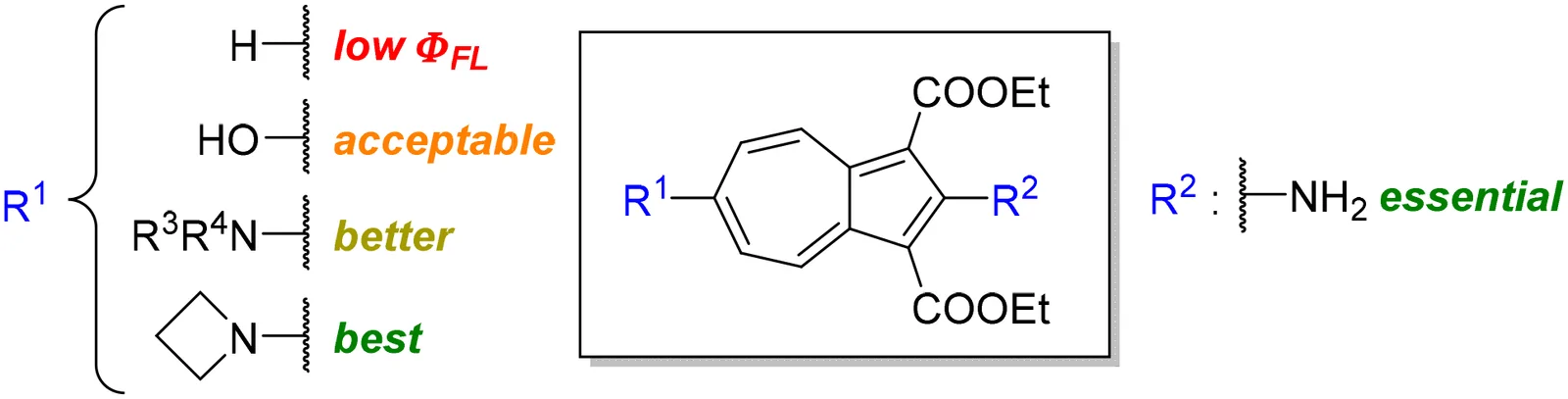
*Φ*_FL_ structure–property relationship in azulene-1,3-diester fluorophores.

## Conclusions

Starting from the structure of our previously reported azulene fluorophore 1, we have designed and synthesized an array of analogues incorporating variation at the 2- and 6-positions. This has been achieved using classical Nozoe azulene syntheses as well as diazotization and acylation at the 2-position, in conjunction with halogenation and nucleophilic aromatic substitution at the 6-position. Photophysical evaluation of these analogues has revealed correlations between the electronic nature of the C2 & C6 substituents and the fluorescence parameters *Φ*_FL_ and *λ*_em_. The three trends in the structure–property relationship elucidated by this work all have implications for the use of azulene fluorophores in bioimaging and other fluorescence sensing applications. The requirement for the electron-donating C2-amino group is a design constraint in one sense but also provides a potential handle for the design of activity-based “turn-on” fluorescent probes. Thus, an azulene in this series bearing a C2 *N*-acylated substituent would be minimally fluorescent (*cf.*20 and 21) but could undergo activation by means of a deacylation reaction to produce an emissive C2-amino derivative. Regarding the substitution requirements at C6, the enhancements in fluorescence quantum yield afforded by various amino substituents (and by an azetidino substituent in particular) pave the way for new azulene-based fluorescent probes having enhanced optical brightness compared to 1. Further modifications at C6 may merit examination, such as rigidification through annulation (with the aim of further enhancing *Φ*_FL_) or introduction of a π-linker between the C6 carbon and the amine EDG (with the aim of achieving larger Stokes shifts and/or two-photon absorption cross-section^52^). Studies in these areas are ongoing in our laboratories and will be reported in due course.

## Author contributions

S. E. L., G. D. P. and T. D. J. conceived and supervised the project and secured funding. C. F. carried out all synthesis and spectroscopy, except fluorescence and UV/vis spectroscopy, which was carried out by J. G. R., G. D. P. and M. K. X-ray crystallography was carried out by G. K. K. The manuscript was written by S. E. L. with input from all authors. All authors have approved the final draft of the manuscript.

## Conflicts of interest

The authors are employees and students of the University of Bath, which owns the trademark “AzuFluor®”.

## Supplementary Material

QO-OLF-D6QO01099G-s001

QO-OLF-D6QO01099G-s002

## Data Availability

All data supporting this study are available in the supplementary information (SI) accompanying this article. Supplementary information: experimental procedures, NMR spectra, UV/vis absorbance spectra, fluorescence spectra, X-ray data. See DOI: https://doi.org/10.1039/d6qo01099g. CCDC 2566798–2566809 contain the supplementary crystallographic data for this paper.^53*a*–*l*^
