# Supplementary material for: Synthesis of azulene fluorophores reveals structure–property relationships
Source: Org Chem Front. 2026 Oct 12. Online ahead of print. doi: 10.1039/d6qo01099g (PMC13655715; doi:10.1039/d6qo01099g)
Supplement: Supplementary file 1 [file QO-OLF-D6QO01099G-s001.pdf]

Supplementary Information (SI) for Organic Chemistry Frontiers.
This journal is © the Partner Organisations

Structure-Property Relationships in Azulene Fluorophores.

Chiara Faverio,[a] João Gabriel Ribeiro,[a] Mehmet Kaplan,[a,b] Gabriele Kociok-Köhn,[c] Tony D. James,*[a,d] G. Dan Pantoş*[a] and Simon E. Lewis*[a]

a. Department of Chemistry, University of Bath, Bath, BA2 7AY, U.K. E mail: S.E.Lewis@bath.ac.uk
b. Ankara University, Faculty of Science, Department of Chemistry, 06100, Ankara, Turkiye. E-mail: mehmetkaplan@ankara.edu.tr
c. Physical Structure Characterization Facility, University of Bath, Bath, BA2 7AY, U.K.
d. School of Chemistry and Chemical Engineering, Henan Normal University, Xinxiang 453007, China

ELECTRONIC SUPPORTING INFORMATION

General Experimental Details — Page S2
Synthetic procedures — Page S3
UV/Vis spectra for selected compounds — Page S28
Fluorescence spectra (halochromism studies) — Page S33
NMR, IR and mass spectra — Page S35
Crystallographic Data — Page S116
References — Page S140

General Experimental Details

All reactions were carried out under an atmosphere of N_2, through the use of a Schlenk line, unless otherwise stated. Anhydrous solvents were either purchased from Fisher Scientific or Sigma-Aldrich or purified through anhydrous alumina columns using an Innovative Technology Inc. PS-400-7 solvent purification system. Solvents were deoxygenated by channelling a stream of N_2 through the liquid (sparging). Thin layer chromatography (TLC) was carried out on aluminium plates coated with silica gel (Alugram®SIL G/$UV_{254\ nm}$), and visualisation was achieved with UV light or $KMnO_4$, ceric ammonium molybdate and iodine dips, followed by gentle heating. Solvents were removed using Büchi rotary evaporators and with high vacuum on a Schlenk line. Flash column chromatography either carried out manually using Davisil® LC 60 Å silica gel (35-70 micron) purchased from Sigma-Aldrich or using a Teledyne ISCO CombiFlash® NextGen300+ system equipped with UV/vis detection. In the latter case, Teledyne RediSep® Bronze pre-packed silica columns were used.

NMR spectra were run in $CDCl_3$ unless otherwise stated, on Bruker Avance 400, Bruker Avance 500 II+ or Agilent A500a instruments. IR spectra were recorded on a Perkin-Elmer 1600 FT-IR instrument. Capillary melting points were recorded on Stanford Research Systems OptiMelt MPA100 melting point apparatus. UV/Vis absorption spectra were acquired using a Jasco J-810 spectrometer or an Agilent Technologies Cary 60 spectrometer. The quantum yields were determined using a Horiba Fluorolog-QM spectrofluorometer equipped with QuantaPhi-2 integrating sphere using the QuantaPhi-2 cuvette holder. Circular dichroism (CD) experiments were performed on an Applied Photophysics Chirascan spectrophotometer equipped with a Peltier temperature controller - the experiments were conducted in quartz cuvettes of 10 mm pathlength in CH_3CN at 20 °C using a 1 nm step acquisition, with a time per point of 0.5 s and a bandwidth of 3 nm. High resolution mass spectrometry (HRMS) was carried out using a micrOTOF ESI-TOF spectrometer coupled to an Agilent 1200 LC system for auto-sampling. For SCXRD the selected crystal was mounted onto a goniometer head and cooled to 150K with an Oxford Cryosystem. Intensity data for compound **17** were collected on a Rigaku New Xcalibur, EosS2 using graphite monochromated Mo-Ka radiation (λ = 0.71073 Å) while intensity data for structures **14, 15a-g, 16-18, 28** and **31** were collected on a SuperNova, Dual, Cu at

zero, EosS2 using a Cu microfocus source (λ = 1.54184) Å. Unit cell determination, data collection, data reduction and a symmetry-related (multi-scan) absorption correction were performed using the CrysAlisPro software.[1] The structures were solved with SHELXT and refined by a full-matrix least-squares procedure based on F^2 (Shelxl-2019/2).[2] All non-hydrogen atoms were refined anisotropically. Hydrogen atoms were placed onto calculated positions and refined using a riding model. Additional programmes used for analysing data and their graphical manipulation included: SHELXle[3] and Mercury[4]

Diethyl 2-amino-6-azidoazulene-1,3-dicarboxylate 13

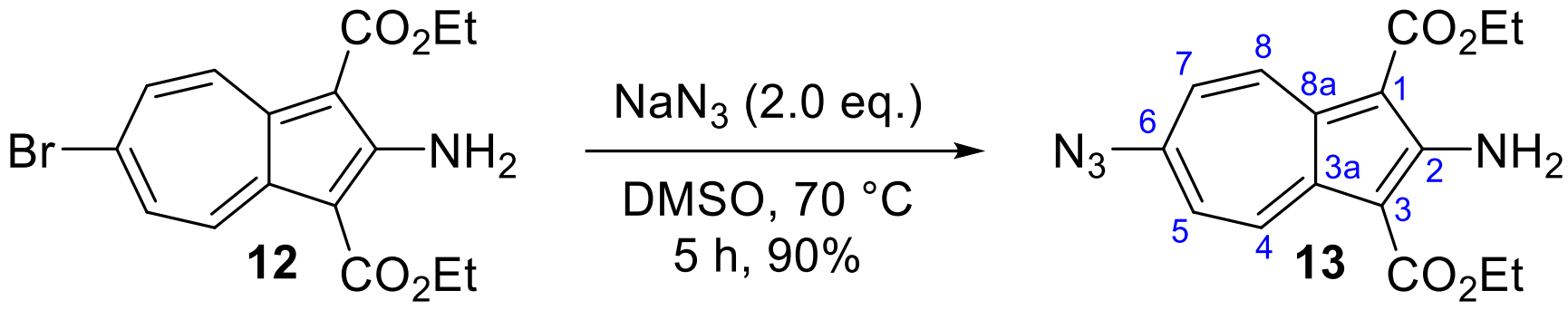

In a 50 mL round-bottom flask, diethyl 2-amino-6-bromoazulene-1,3-dicarboxylate **12**[5] (150 mg, 0.41 mmol, 1.0 eq.) and sodium azide (53 mg, 0.82 mmol, 2.0 eq.) were weighed. The flask was equipped with a condenser, and the system was purged with N_2. Anhydrous DMSO (13.6 mL) was added, and the resulting mixture was heated at 70 °C for 4 hours. The reaction was quenched by addition of H_2O (40 mL), and the mixture was extracted with ethyl acetate (40 mL × 3). The combined organic layers were washed with brine (40 mL), dried over $MgSO_4$, filtered and concentrated under reduced pressure. The crude was re-dissolved in 10 mL of 1:1 mixture of petrol ether 40-60/ethyl acetate and filtered through a pad of basic aluminium oxide (Brockmann I for chromatography, 40-300 µm, 60 Å). The solids were then washed with 100 mL of a 1:1 mixture of petrol ether 40-60/ethyl acetate. The filtrate was concentrated under reduced pressure to give **13** (121 mg, 90%) as a brown solid. ^{1}H-NMR (400 MHz, $CDCl_3$) δ 9.08 (2H, d, J = 11.2 Hz, H4,8), 7.71 (2H, s, $-NH_2$), 7.21 (2H, d, J = 11.2 Hz, H5,7), 4.47 (4H, q, J = 7.1 Hz, -OC<u>*H*</u>$_2$CH$_3$), 1.48 (6H, t, J = 7.1 Hz, -OCH$_2$C<u>*H*</u>$_3$) ppm; HRMS (ESI+) *m/z* calc for $[C_{16}H_{16}N_4O_4 + H]^+$, 329.1244; found, 329.1247. **13** was unstable in solution, so further purification or characterisation was not possible. Accordingly, **13** was used immediately in the next step.

Diethyl 2,6-diaminoazulene-1,3-dicarboxylate 14

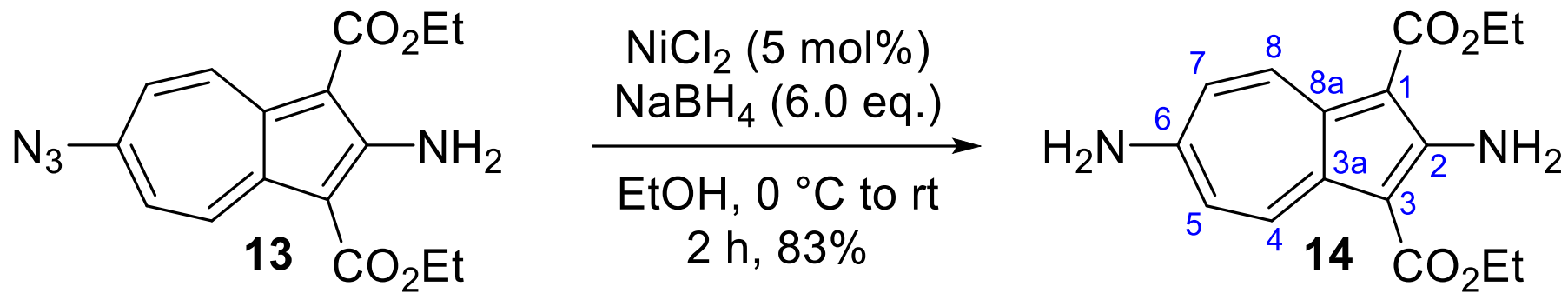

Diethyl 2-amino-6-azidoazulene-1,3-dicarboxylate **13** (76 mg, 0.23 mmol, 1.0 eq.) and nickel (II) chloride (1.5 mg, 0.012 mmol, 0.05 eq.) were dissolved in *abs*. ethanol (4.6 mL) under inert atmosphere. The solution was cooled to 0 °C with an ice bath and sodium borohydride (53 mg, 1.39 mmol, 6 eq.) was added in one portion. The reaction mixture was stirred at 0 °C for 30 minutes and at room temperature for 1.5 hours. After this time, the reaction mixture was cooled to 0 °C with an ice bath, quenched with saturated aqueous NH_4Cl (12 mL) and then diluted with H_2O (5 mL). The reaction mixture was extracted with ethyl acetate (30 mL × 3); the combined organic layers were washed with brine (30 mL), dried over $MgSO_4$, filtered and concentrated under reduced pressure. The crude was purified by re-crystallization with a solvent/antisolvent system, using chloroform (5 mL) and slowly adding *n*-hexane (15 mL), to give **14** (58 mg, 83%) as a yellow solid; m.pt. 208 °C (Lit.[6] 207-208 °C); R_f 0.31 (EtOAc/PE 1:1); ^{1}H-NMR (400 MHz, $CDCl_3$) δ 9.00 (2H, d, *J* = 11.5 Hz, H4,8), 7.28 (2H, br s, C2-NH_2), 6.87 (2H, app dt, *J* = 11.5, 1.3 Hz, H5,7), 4.51 (2H, s, C6-NH_2), 4.43 (4H, q, *J* = 7.1 Hz, -C$\underline{H}_2$$CH_3$), 1.45 (6H, t, *J* = 7.1 Hz, CH_3) ppm; ^{13}C-NMR (101 MHz, $CDCl_3$) δ 166.88 (C=O), 159.08 (C2), 153.39 (C6), 139.65 (C3a,8a), 133.66 (C4,8), 119.03 (C5,7), 99.72 (C1,3), 59.56 (-$\underline{C}H_2CH_3$), 14.88 (CH_3) ppm; IR ν_{max} (film) 3501, 3424, 3393, 3341, 3230, 2978, 1639, 1572, 1493, 1435, 1196, 1094, 1029, 955, 845, 783, 726 cm^{-1}; HRMS (ESI+) *m*/*z* calc for $[C_{16}H_{18}N_2O_4 + Na]^+$, 325.1159; found, 325.1157.

Diethyl 2-amino-6-(dimethylamino)azulene-1,3-dicarboxylate 15a

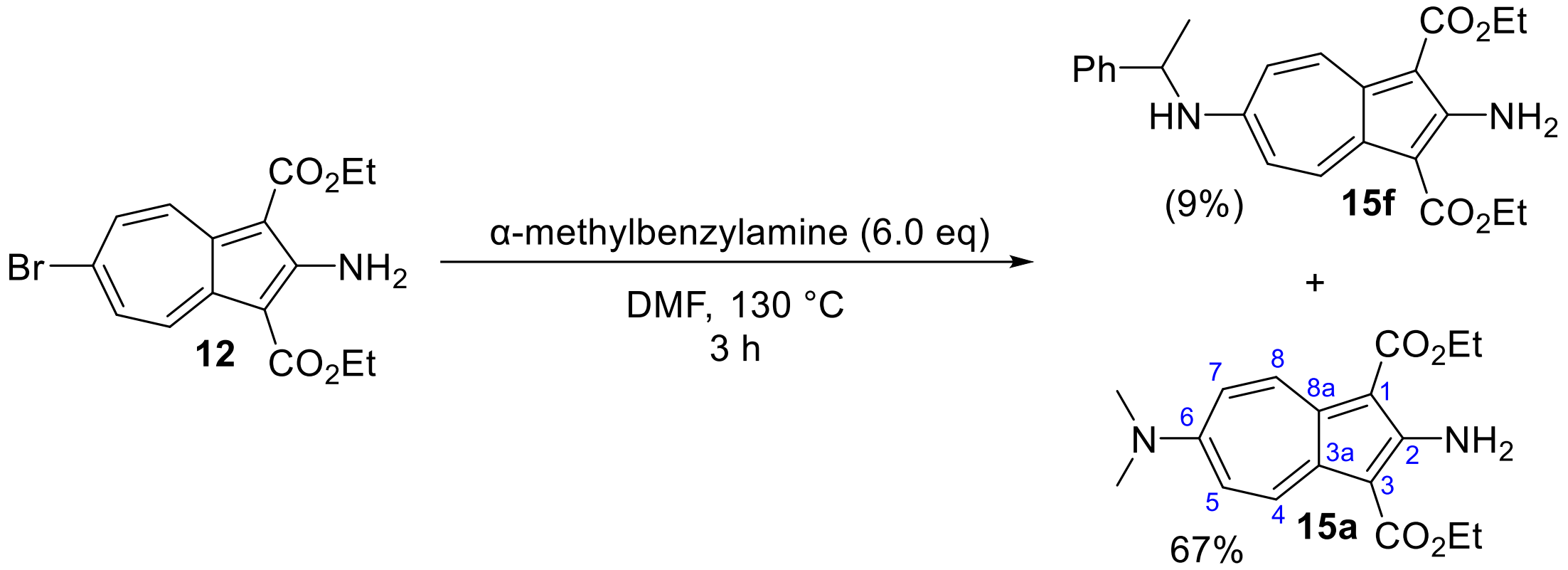

To diethyl 2-amino-6-bromoazulene-1,3-dicarboxylate **12** (100 mg, 0.27 mmol, 1.0 eq.) in a 10 mL CEM vial, anhydrous DMF (1.8 mL) was added under inert atmosphere. (*S*)-methylbenzylamine (0.21 mL, 1.6 mmol, 6.0 eq.) was added and the resulting suspension was irradiated in a MW apparatus (130 °C, 200 W) for 3 hours. The reaction mixture was diluted with H_2O (10 mL) and extracted with ethyl acetate (10 mL × 3). The combined organic layers were washed with brine (10 mL), dried over $MgSO_4$, filtered and concentrated under reduced pressure. The crude was purified by chromatography (12 g silica cartridge; hexane/EtOAc 100:0 → 0:100) to give desired product **15f** (10 mg, 9%) as a yellow solid; R_f 0.26 (PE/EtOAc 7:3) and byproduct **15a** (60 mg, 67%) as a yellow/orange solid; R_f 0.18 (PE/EtOAc 7:3). Spectroscopic data for **15f** are given on page S11.

15a: m.pt 177.7-183.9 °C; ^{1}H-NMR (400 MHz, $CDCl_3$) δ 8.98 (2H, d, *J* = 12.1 Hz, H4,8), 7.24 (2H, s, NH_2), 6.90 (2H, d, *J* = 12.1 Hz, H5,7), 4.42 (4H, q, *J* = 7.1 Hz, -C<u>*H*</u>$_2$CH$_3$), 3.21 (6H, s, $N(CH_3)_2$) 1.40 (6H, t, *J* = 7.1 Hz, CH_3) ppm; ^{13}C-NMR (101 MHz, $CDCl_3$) 166.99 (C=O), 158.87 (C2), 155.65 (C6), 138.00 (C3a,8a), 132.94 (C4,8), 116.64 (C5,7), 98.93 (C1,3), 59.36 (-<u>*C*</u>H$_2$CH$_3$), 41.90 ($N(CH_3)_2$), 14.93 (CH_3) ppm; IR ν_{max} (film) 3498, 3442, 3354, 3324, 2975, 2921, 1657, 1577, 1562, 1424, 1385, 1341, 1279, 1229, 1197, 1094, 1029, 908, 884, 818, 787, 693, 657 cm^{-1}; HRMS (ESI+) *m*/*z* calc for $[C_{18}H_{22}N_2O_4 + Na]^+$, 353.1472; found, 353.1472. Crystals suitable for x-ray diffraction were grown by slow diffusion of hexane into a $CHCl_3$ solution of **15a**.

General procedure for synthesis of 15b-15g

In a 25 mL round-bottom flask, diethyl 2-amino-6-bromoazulene-1,3-dicarboxylate **12** (100 mg; 0.27 mmol; 1.0 eq.) was weighted. The flask was equipped with a condenser, and the system was purged with N_2. Anhydrous DMSO (1.8 mL) and the nucleophile (6.0 eq.) were added in this order, the resulting orange/brown suspension was heated at 80 °C (70 °C for **15d**; 50 °C for **15e**; 130 °C **15f** and **15g**) overnight. The mixture was cooled at room temperature and diluted with H_2O (20 mL) and the mixture was extracted with ethyl acetate (25 mL × 3). The combined organic layers were washed with brine (20 mL), dried over $MgSO_4$, filtered and concentrated under reduced pressure. Purification specified for each compound.

Diethyl 2-amino-6-(piperidin-1-yl)azulene-1,3-dicarboxylate 15b

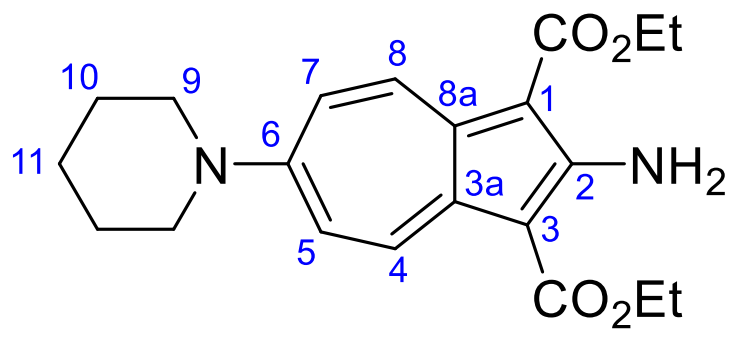

15b

15b: purified by chromatography (12 g silica cartridge; hexane/EtOAc 100:0 → 0:40); 76 mg (75%), bright yellow solid; m.pt 96.8-98.9 °C; R_f 0.64 (PE/EtOAc 6:4); ^{1}H-NMR (400 MHz, $CDCl_3$) δ 8.97 (2H, d, *J* = 12.0 Hz, H4,8), 7.32 (2H, br s, NH_2), 7.13 (2H, d, *J* = 12.0 Hz, H5,7), 4.42 (4H, q, *J* = 7.1 Hz, -C$\underline{H}_2$CH$_3$), 3.52-3.46 (4H, m, H9) 1.77-1.66 (6H, m, H10,11), 1.45 (6H, t, *J* = 7.1 Hz, CH_3) ppm; ^{13}C-NMR (101 MHz, $CDCl_3$) 166.92 (C=O), 159.58 (C2), 157.25 (C6), 139.58 (C3a,8a), 132.51 (C4,8), 121.07 (C5,7), 99.19 (C1,3), 59.45 (-$\underline{C}$H$_2$CH$_3$), 51.09 (C9), 26.00 (C10), 24.39 (C11), 14.90 (CH_3) ppm; IR ν_{max} (film) 3451, 3329, 2937, 1660, 1630, 1565, 1512, 1427, 1369, 1334, 1215, 1092, 1019, 882, 832, 792, 665 cm^{-1}; HRMS (ESI+) *m/z* calc for $[C_{21}H_{26}N_2O_4 + Na]^+$, 393.1785; found, 393.1787. Crystals suitable for x-ray diffraction were grown by slow diffusion of hexane into a $CHCl_3$ solution of **15b**. ^{1}H-NMR data are in agreement with those previously reported[7] (other data have not previously been reported).

Diethyl 2-amino-6-morpholinoazulene-1,3-dicarboxylate 15c

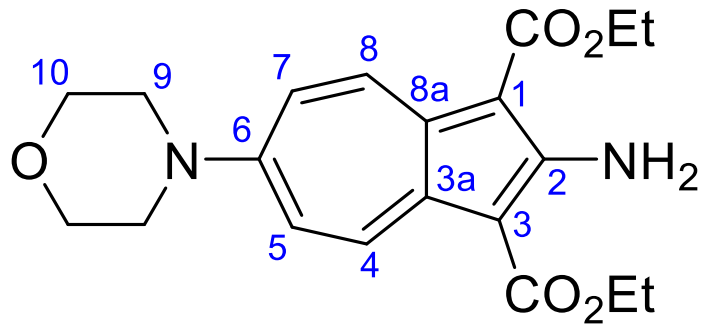

15c

15c: purified by solvent/antisolvent crystallization from 1.5 mL of chloroform and 15 mL of *n*-hexane; 70 mg (70%), bright yellow solid ; m. pt. 148.2-152.1 °C; R_f 0.44 (PE/EtOAc 1:1); ^{1}H-NMR (400 MHz, $CDCl_3$) δ 9.03 (2H, d, *J* = 11.9 Hz, H4,8), 7.41 (2H, br s, NH_2), 7.15 (2H, d, *J* = 11.9 Hz, H5,7), 4.43 (4H, q, *J* = 7.1 Hz, -C$\underline{H}_2$$CH_3$), 3.90-3.86 (4H, m, H10) 3.45-3.40 (4H, m, H9), 1.46 (6H, t, *J* = 7.1 Hz, CH_3) ppm; ^{13}C-NMR (101 MHz, $CDCl_3$) 166.82 (C=O), 160.17 (C2), 156.51 (C6), 140.64 (C3a,8a), 132.06 (C4,8), 121.41 (C5,7), 99.62 (C1,3), 66.92 (C10), 59.63 (-$\underline{C}H_2CH_3$), 50.23 (C9), 14.87 (CH_3) ppm; IR ν_{max} (film) 3427, 3318, 2978, 1671, 1589, 1564, 1478, 1366, 1332, 1183, 1110, 1069, 1027, 885, 824, 792, 679, 586 cm^{-1}; HRMS (ESI+) *m*/*z* calc for $[C_{20}H_{24}N_2O_5 + H]^+$, 373.1758; found, 373.1756. Crystals suitable for x-ray diffraction were grown by slow diffusion of hexane into a $CHCl_3$ solution of **15c**. ^{1}H-NMR data have been previously reported, but in a different solvent[7] (other data have not previously been reported).

Diethyl 2-amino-6-(pyrrolidin-1-yl)azulene-1,3-dicarboxylate 15d

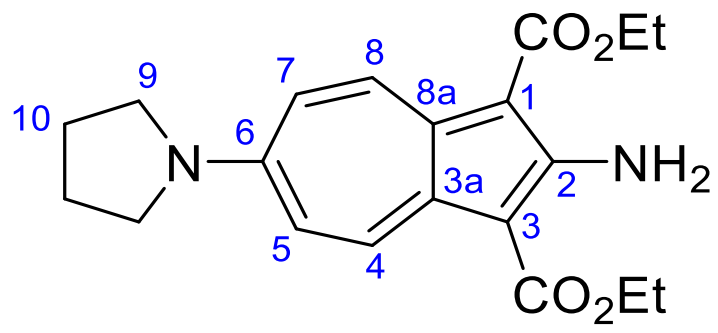

15d

15d: purified by chromatography (12 g silica cartridge; hexane/EtOAc 100:0 → 0:95) and then re-crystallized with solvent/antisolvent technique (dissolved in 3 mL of chloroform and precipitated by adding 9 mL of *n*-hexane). 65 mg (67%), bright yellow solid; m. pt. 221.2-222.5 °C; R_f 0.22 (PE/EtOAc 7:3); ^{1}H-NMR (400 MHz, $CDCl_3$) δ 8.98 (2H, d, *J* = 12.0 Hz, H4,8), 7.19 (2H, br s, NH_2), 6.77 (2H, d, *J* = 12.0 Hz, H5,7), 4.42 (4H, q, *J* = 7.1 Hz, -C$\underline{H}_2$$CH_3$), 3.51-3.46 (4H, m, H9) 2.13-2.05 (4H, m, H10), 1.45 (6H, t, *J* = 7.1 Hz, CH_3) ppm; ^{13}C-NMR (101 MHz, $CDCl_3$) 166.91 (C=O), 158.34 (C2), 153.43 (C6), 137.28 (C3a,8a), 133.28 (C4,8), 116.74 (C5,7), 98.69 (C1,3), 59.18 (-$\underline{C}H_2CH_3$), 48.93 (C9), 25.62 (C10), 14.81 (CH_3) ppm; IR ν_{max} (film) 3498, 3359, 2972, 1650, 1562, 1428, 1371, 1337, 1274, 1247, 1203, 1096, 1033, 971, 905, 883, 816, 788, 695 cm^{-1}; HRMS (ESI+) *m/z* calc for $[C_{20}H_{24}N_2O_4 + Na]^+$, 379.1628; found, 379.1630. Crystals suitable for x-ray diffraction were grown by slow diffusion of hexane into a $CHCl_3$ solution of **15d**. ^{1}H-NMR data are in agreement with those previously reported[7] (other data have not previously been reported).

Diethyl 2-amino-6-(azetidin-1-yl)azulene-1,3-dicarboxylate 15e

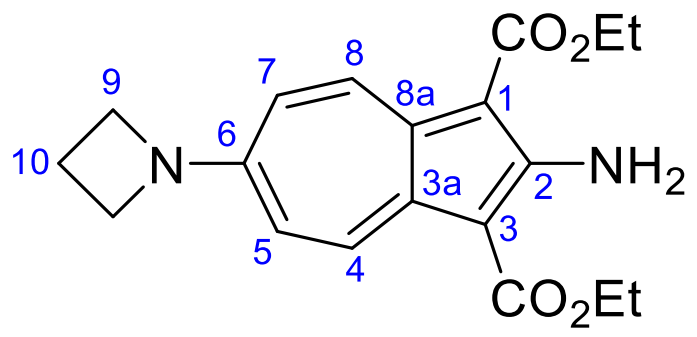

15e

15e: purified by chromatography (12 g silica cartridge; hexane/EtOAc 100:0 → 0:60) 55 mg (54%), yellow solid; m. pt. 222.0-225.9 °C; R_f 0.29 (PE/EtOAc 6:4); ^{1}H-NMR (400 MHz, $CDCl_3$) δ 8.95 (2H, d, *J* = 11.7 Hz, H4,8), 7.21 (2H, br s, NH_2), 6.38 (2H, d, *J* = 11.7 Hz, H5,7), 4.41 (4H, q, *J* = 7.1 Hz, -C$\underline{H}_2$$CH_3$), 4.10 (4H, t, *J* = 7.4 Hz, H9) 2.42 (2H, p, *J* = 7.4 Hz, H10), 1.44 (6H, t, *J* = 7.1 Hz, CH_3) ppm; ^{13}C-NMR (101 MHz, $CDCl_3$) 167.00 (C=O), 158.44 (C2), 155.14 (C6), 137.5 (C3a,8a), 133.48 (C4,8), 114.08 (C5,7), 99.16 (C1,3), 59.36 (-$\underline{C}H_2CH_3$), 51.47 (C9), 15.25 (C10), 14.92 (CH_3) ppm; IR ν_{max} (film) 3517, 3392, 2922, 1667, 1647, 1573, 1562, 1455, 1431, 1375, 1345, 1308, 1268, 1239, 1205, 1149, 1088, 1037, 92, 913, 889, 830, 819, 787, 693 cm^{-1}; HRMS (ESI+) *m/z* calc for $[C_{19}H_{22}N_2O_4 + H]^+$, 343.1652; found, 343.1645. Crystals suitable for x-ray diffraction were grown by slow diffusion of hexane into a $CHCl_3$ solution of **15e**.

Diethyl (*R*)-2-amino-6-((1-phenylethyl)amino)azulene-1,3-dicarboxylate (*R*)-15f

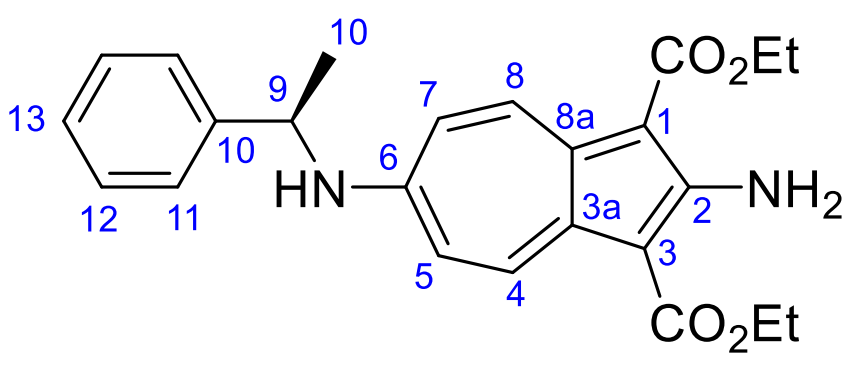

(*R*)-**15f**

(*R*)-**15f**: reaction run for 40 hours; purified by chromatography (12 g silica cartridge; hexane/EtOAc 100:0 → 0:100); 69 mg (63%), yellow solid; m. pt. 174.7-181.2 °C; R_f 0.36 (PE/EtOAc 6:4); ^{1}H-NMR (400 MHz, $CDCl_3$) δ 8.92 (2H, d, *J* = 11.7 Hz, H4,8), 7.37-7.35 (5H, m, Ph), 7.21 (2H, br s, NH_2), 6.73 (2H, d, *J* = 11.7 Hz, H5,7), 5.02 (1H, d, *J* = 5.9 Hz, C6-NH), 4.67 (app p, *J* = 6.5 Hz, H9), 4.46-4.34 (4H, m, diastereotopic -C$\underline{H}_2$$CH_3$), 1.61 (3H, d, *J* = 6.7 Hz H10), 1.42 (6H, t, *J* = 7.1 Hz, -CH_2C$\underline{H}_3$) ppm; ^{13}C-NMR (101 MHz, $CDCl_3$) 166.86 (C=O), 158.77 (C2), 153.21 (C6), 143.11 (C10), 138.72 (C3a,8a), 133.49 (C4,8), 129.15 (C12), 127.70 (C13), 125.79 (C11), 117.88 (C5,7), 99.34 (C1,3), 59.43 (-$\underline{C}H_2CH_3$), 53.58 (C9), 24.84 (C10), 14.90 (CH_3) ppm; IR ν_{max} (film) 3502, 3362, 3323, 2969, 2925, 1644, 1574, 1529, 1484, 1428, 1373, 1320, 1300, 1267, 1238, 1202, 1105, 1035, 961, 930, 893, 847, 829, 789, 748, 697 cm^{-1}; HRMS (ESI+) *m*/*z* calc for [$C_{24}H_{27}N_2O_4$ + Na]$^+$, 429.1785; found, 429.1786. Molar ellipticity (474 nm): -5500 deg×cm^2/dmol Crystals suitable for x-ray diffraction were grown by slow diffusion of hexane into a *tert*-butyl methyl ether solution of (*R*)-**15f**.

(*S*)-**15f**: Molar ellipticity (474 nm): 5500 deg×cm^2/dmol

Diethyl (*S*)-2-amino-6-(nonan-2-ylamino)azulene-1,3-dicarboxylate (*S*)-15g

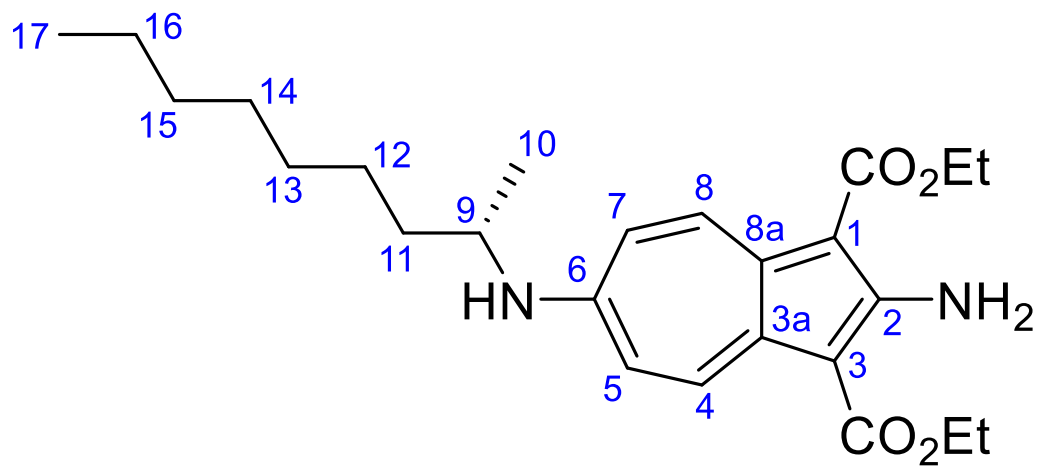

(*S*)-**15g**

(*S*)-**15g**: purified by chromatography (12 g silica cartridge; hexane/EtOAc 100:0 → 0:50); 58 mg (50%), yellow solid; m. pt. 100.4-106.5 °C; R_f 0.66 (PE/EtOAc 7:3); ^{1}H-NMR (400 MHz, $CDCl_3$) δ 8.98 (2H, d, *J* = 11.5 Hz, H4,8), 7.19 (2H, br s, NH_2), 6.74 (2H, d, *J* = 11.5 Hz, H5,7), 4.59 (1H, d, *J* = 7.9 Hz, C6-NH), 4.42 (4H, app q, *J* = 7.1 Hz, -OC$\underline{H}_2$CH$_3$), 3.64 (1H, app hept, J = 6.5 Hz, H9), 1.64-1.48 (2H, m, H11), 1.44 (6H, t, *J* = 7.1 Hz, -OCH$_2$C$\underline{H}_3$), 1.41-1.23 (10H, m, H12-16), 1.25 (3H, d, *J* = 6.2 Hz, H10), 0.87 (3H, t, *J* = 6.7 Hz, H17) ppm; ^{13}C-NMR (101 MHz, $CDCl_3$) δ 166.93 (C=O), 158.54 (C2), 154.11 (C6), 138.13 (C3a,8a), 133.78 (C4,8), 117.29 (C5,7), 99.22 (C1,3), 59.38 (-O$\underline{C}$H$_2$CH$_3$), 48.88 (C9), 36.92 (C11), 31.90 (C12-16), 29.61 (C12-16), 29.35 (C12-16), 26.16 (C12-16), 22.75 (C12-16), 20.49 (C10), 14.89 (-OCH$_2\underline{C}$H$_3$), 14.20 (C17) ppm; IR ν_{max} (film) 3502, 3337, 2928, 2851, 1647, 1573, 1531, 1483, 1470, 1431, 1374, 1328, 1268, 1208, 1113, 1037, 942, 894, 842, 830, 754, 720, 691 cm^{-1}; HRMS (ESI+) *m*/*z* calc for $[C_{25}H_{36}N_2O_4 + Na]^+$, 451.2567; found, 451.2571. Molar ellipticity (474 nm): 3000 deg×cm^2/dmol

Diethyl 2-acetamido-6-bromoazulene-1,3-dicarboxylate 16 and diethyl 2-(*N*-acetylacetamido)-6-bromoazulene-1,3-dicarboxylate 17

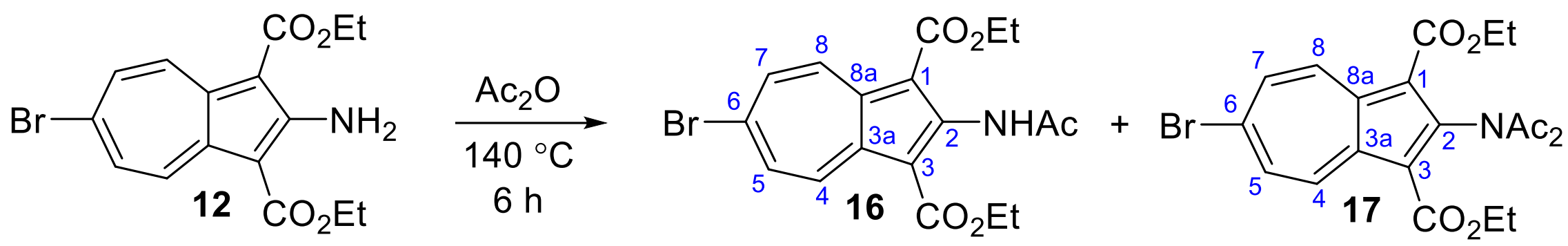

In a 25 mL round bottom flask 700 mg (1.91 mmol) of diethyl 2-amino-6-bromoazulene-1,3-dicarboxylate **12**[5] were weighted, the system was equipped with a condenser and purged with N_2. Acetic anhydride (1.9 mL) was added and the resulting red suspension was heated at 140 °C, upon heating the suspension becomes a red solution. After 6 hours, the reaction mixture is cooled to room temperature and excess acetic anhydride is removed under reduced pressure. The crude was purified by chromatography (24 g silica cartridge; hexane/EtOAc 100:0 → 0:80) to give **16** (126 mg, 16%) and **17** (508 mg, 59%).

16: pink solid, m.pt. 208-211 °C (lit.[8] 212-213 °C); R_f 0.19 (PE/EtOAc 7:3); ^{1}H-NMR (500 MHz, $CDCl_3$) δ 10.53 (1H, s, NH), 8.94 (2H, d, *J* = 11.4 Hz, H4,8), 7.93 (2H, d, *J* = 11.4 Hz, H5,7), 4.45 (4H, q, *J* = 7.1 Hz, -C$\underline{H}_2$CH$_3$), 2.30 (3H, s, NCOCH$_3$), 1.44 (6H, t, *J* = 7.1 Hz, -CH$_2$C$\underline{H}_3$) ppm; ^{13}C-NMR (126 MHz, $CDCl_3$) δ 168.20 (N$\underline{C}$OCH$_3$), 165.92 ($\underline{C}$OOEt), 147.74 (C2), 139.91 (C3a,8a), 134.85 (C6), 134.15 (C4,8), 133.89 (C5,7), 109.42 (C1,3), 60.96 ($\underline{C}$H$_2$CH$_3$), 24.70 (NCO$\underline{C}$H$_3$), 14.60 (CH$_2\underline{C}$H$_3$) ppm; IR ν_{max} (film) 3242, 2983, 1703, 1659, 1542, 1487, 1436, 1422, 1363, 1260, 1223, 1176, 1125, 1024, 999, 831, 782, 729, 671, 629 cm^{-1}; HRMS (ESI+) *m/z* calc for $[C_{18}H_{18}{}^{79}BrNO_5 + H]^+$, 408.0441; found, 408.0456. Crystals suitable for x-ray diffraction were grown by slow diffusion of pet. ether into an ethyl acetate solution of **16**. Melting point and infrared data are in agreement with those previously reported[8]; other data have not been reported previously.

17: bright red solid, m.pt. 146-148 °C (lit.[8] 141-142 °C); R_f 0.19 (PE/EtOAc 7:3); ^{1}H-NMR (500 MHz, $CDCl_3$) δ 9.58 (2H, d, *J* = 11.1 Hz, H4,8), 8.12 (2H, d, *J* = 11.1 Hz, H5,7), 4.38 (4H, q, *J* = 7.1 Hz, -C*H*$_2$CH$_3$), 2.29 (6H, s, NCOCH$_3$), 1.37 (6H, t, *J* = 7.1 Hz, -CH$_2$C*H*$_3$) ppm; ^{13}C-NMR (126 MHz, $CDCl_3$) δ 172.25 (N*C*OCH$_3$), 163.55 (*C*OOEt), 149.30 (C2), 140.86 (C3a,8a), 140.33 (C6), 138.70 (C4,8), 134.99 (C5,7), 114.94 (C1,3), 60.91 (*C*H$_2$CH$_3$), 26.75 (NCO*C*H$_3$), 14.45 (CH$_2$*C*H$_3$) ppm; IR ν_{max} (film) 2986, 1702, 1684, 1571, 1421, 1366, 1286, 1253, 1190, 1139, 1074, 1023, 842, 690 cm^{-1}; HRMS (ESI+) *m/z* calc for $[C_{20}H_{20}{}^{79}BrNO_6 + H]^+$, 450.0547; found, 450.0543. Crystals suitable for x-ray diffraction were grown by slow diffusion of pet. ether into an ethyl acetate solution of **17**. Melting point and infrared data are in agreement with those previously reported[8]; other data have not been reported previously.

Diethyl 2-acetamido-6-azidoazulene-1,3-dicarboxylate 18

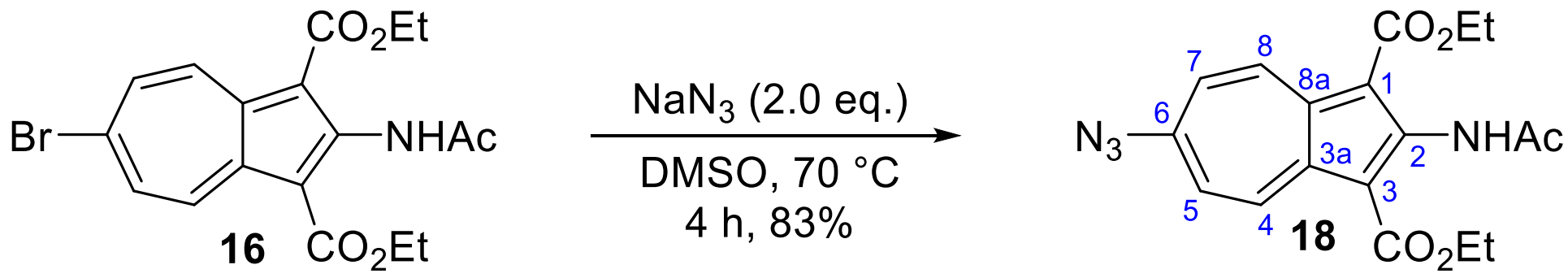

2-acetamido-6-bromoazulene-1,3-dicarboxylate **16** (109 mg, 0.27 mmol, 1.0 eq.) and sodium azide (35 mg, 0.53 mmol, 2.0 eq.) were weighted in a 25 mL round-bottom flask. The flask was equipped with a condenser, and the system was purged with N_2. Anhydrous DMSO (8.9 mL) was added and the reaction mixture was heated at 70 °C for 4 hours. The mixture was cooled at room temperature and diluted with H_2O (50 mL) and the mixture was extracted with ethyl acetate (50 mL × 3). The combined organic layers were washed with brine (50 mL × 2), dried over $MgSO_4$, filtered and concentrated under reduced pressure. The crude was solid loaded onto celite and purified by chromatography (12 g silica cartridge; 3CV of hexane then hexane/EtOAc 70:30 → 50:50) to give **18** as an orange solid (82 mg, 83%). m.pt. 140.2 °C (dec.); R_f 0.34 (PE/EtOAc 1:1); ^{1}H-NMR (400 MHz, $CDCl_3$) δ 10.42 (1H, br s, NH), 9.11 (2H, d, *J* = 11.4 Hz, H4,8), 7.23 (2H, d, *J* = 11.4 Hz, H5,7), 4.44 (4H, q, *J* = 7.1 Hz, -C<u>*H*</u>$_2$CH$_3$), 2.29 (3H, s, NCOCH$_3$), 1.43 (6H, t, *J* = 7.1 Hz, -CH$_2$C<u>*H*</u>$_3$) ppm; ^{13}C-NMR (101 MHz, $CDCl_3$) δ 168.21 (N<u>*C*</u>OCH$_3$), 166.02 (<u>*C*</u>OOEt), 149.29 (C6), 146.14 (C2), 138.40 (C3a,8a), 135.27 (C4,8), 121.22 (C5,7), 109.34 (C1,3), 60.81 (<u>*C*</u>H$_2$CH$_3$), 24.64 (NCO<u>*C*</u>H$_3$), 14.58 (CH$_2$<u>*C*</u>H$_3$) ppm; IR ν_{max} (film) 3265, 2979, 2904, 2117, 1736, 1709, 1650, 1581, 1543, 1488, 1432, 1371, 1279, 1237, 1180, 1112, 1027, 839, 756, 589 cm^{-1}; HRMS (ESI+) *m/z* calc for $[C_{18}H_{18}N_4O_5 + Na]^+$, 393.1169; found, 393.1162.

Diethyl 2-(*N*-acetylacetamido)-6-azidoazulene-1,3-dicarboxylate 19

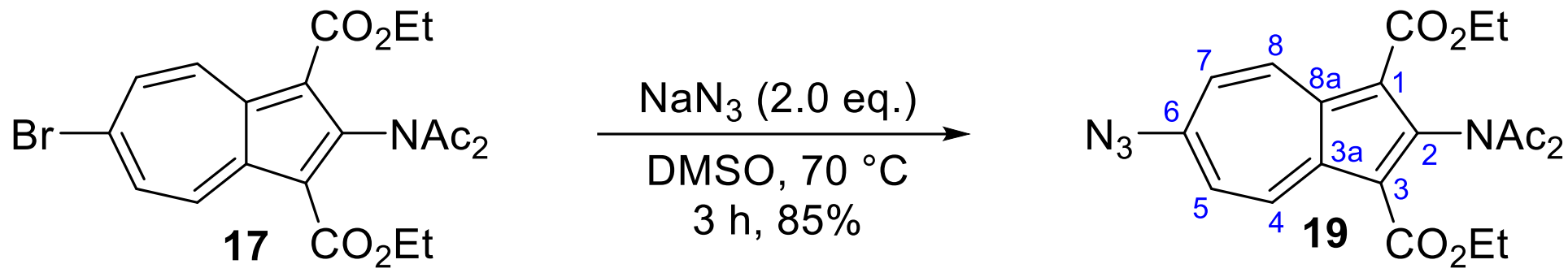

2-(*N*-acetylacetamido)-6-bromoazulene-1,3-dicarboxylate **17** (130 mg, 0.29 mmol, 1.0 eq.) and sodium azide (38 mg, 0.58 mmol, 2.0 eq.) were weighted in a 25 mL round-bottom flask. The flask was equipped with a condenser, and the system was purged with N_2. Anhydrous DMSO (9.6 mL) was added and the reaction mixture was heated at 70 °C for 3 hours. The mixture was cooled at room temperature and diluted with H_2O (50 mL) and the mixture was extracted with ethyl acetate (50 mL × 3). The combined organic layers were washed with brine (50 mL), dried over $MgSO_4$, filtered and concentrated under reduced pressure. The crude was solid loaded onto celite and purified by chromatography (12 g silica cartridge; hexane/EtOAc 100:0 → 60:40) to give **19** as a red/pink solid (101 mg, 85%). m.pt. 133.6 °C (dec.); R_f 0.31 (PE/EtOAc 7:3); ^{1}H-NMR (400 MHz, $CDCl_3$) δ 9.73 (2H, d, *J* = 11.4 Hz, H4,8), 7.40 (2H, d, *J* = 11.4 Hz, H5,7), 4.37 (4H, q, *J* = 7.1 Hz, -C<u>*H*</u>$_2$CH$_3$), 2.30 (6H, s, NCOCH$_3$), 1.37 (6H, t, *J* = 7.1 Hz, -CH$_2$C<u>*H*</u>$_3$) ppm; ^{13}C-NMR (101 MHz, $CDCl_3$) δ 172.51 (N<u>*C*</u>OCH$_3$), 163.81 (<u>*C*</u>OOEt), 154.01 (C6), 147.39 (C2), 139.84 (C4,8), 139.34 (C3a,8a), 122.01 (C5,7), 114.59 (C1,3), 60.76 (<u>*C*</u>H$_2$CH$_3$), 26.77 (NCO<u>*C*</u>H$_3$), 14.47 (CH$_2$<u>*C*</u>H$_3$) ppm; IR ν_{max} (film) 2984, 2113, 1723, 1682, 1584, 1425, 1366, 1291, 1235, 1218, 1197, 1137, 1093, 1025, 866, 689, 593 cm^{-1}; HRMS (ESI+) *m/z* calc for $[C_{20}H_{20}N_4O_6 + Na]^+$,435.1275; found, 435.1268.

Diethyl 2-acetamido-6-aminoazulene-1,3-dicarboxylate 20

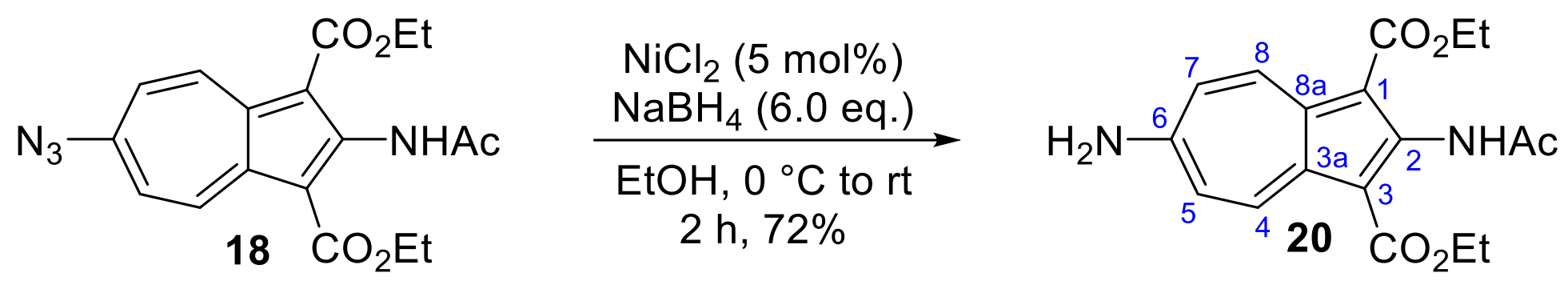

Diethyl 2-acetamido-6-azidoazulene-1,3-dicarboxylate **18** (82 mg, 0.22 mmol, 1.0 eq.) and nickel (II) chloride (1.4 mg, 0.011 mmol, 0.05 eq.) were suspended in *abs*. ethanol (4.4 mL) in a 25 mL round-bottom flask under N_2 atmosphere. The orange suspension was cooled to 0 °C in an ice-bath and sodium borohydride (50 mg, 1.3 mmol, 6.0 eq.) was added. The reaction mixture was stirred for 10 minutes at 0 °C and then allowed to warm to room temperature; overtime the reaction mixture becomes a yellow suspension. After 2 hours, the reaction mixture is cooled to 0 °C and quenched with saturated aqueous NH_4Cl (10 mL). The mixture is diluted with H_2O (10 mL) and extracted with ethyl acetate (25 mL × 3). The combined organic layers were washed with brine (25 mL), dried over $MgSO_4$, filtered and concentrated under reduced pressure. The crude was solid loaded onto celite and purified by chromatography (12 g silica cartridge; CH_2Cl_2/ EtOAc 100:0 → 0:100) to give **20** as an yellow solid (55 mg, 72%). m.pt. 228.2-230.3 °C (lit.[8] 225-225.5 °C); R_f 0.17 (CH_2Cl_2/ EtOAc 1:2); ^{1}H-NMR (400 MHz, $CDCl_3$) δ 9.97 (1H, br s, C2-NH), 8.85 (2H, d, *J* = 11.5 Hz, H4,8), 6.70 (2H, dt, *J* = 11.5, 1.3 Hz, H5,7), 5.08 (2H, br s, C6-NH_2), 4.39 (4H, q, *J* = 7.1 Hz, -C<u>*H*</u>$_2$$CH_3$), 2.25 (3H, s, NHCOC<u>*H*</u>$_3$), 1.41 (6H, t, *J* = 7.1 Hz, -CH_2C<u>*H*</u>$_3$) ppm; ^{13}C-NMR (101 MHz, $CDCl_3$) δ 168.45 (N<u>*C*</u>OCH_3), 166.58 (<u>*C*</u>OOEt), 158.22 (C6), 141.16 (C2)f, 137.68 (C4,8), 134.44 (C3a,8a), 117.11 (C5,7), 108.18 (C1,3), 60.24 (<u>*C*</u>$H_2$$CH_3$), 24.62 (NCO<u>*C*</u>$H_3$), 14.66 ($CH_2$<u>*C*</u>$H_3$) ppm; the N<u>*C*</u>O$CH_3$ signal was visible in the HMBC spectrum only; IR ν_{max} (film) 3406, 3340, 3236, 2972, 2930, 2901, 2864, 1645, 1600, 1577, 1541, 1432, 1378, 1340, 1281, 1208, 1122, 1110, 1072, 1032, 838, 780, 588 cm^{-1}; HRMS (ESI+) *m*/*z* calc for $[C_{18}H_{20}N_2O_5 + Na]^+$, 367.1264; found, 367.1261.

Diethyl 2-(*N*-acetylacetamido)-6-aminoazulene-1,3-dicarboxylate 21

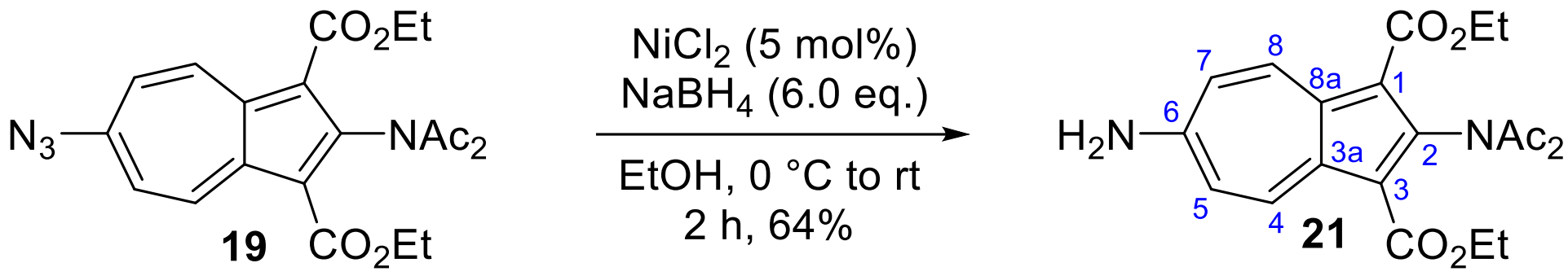

Diethyl 2-(*N*-acetylacetamido)-6-azidoazulene-1,3-dicarboxylate **19** (101 mg, 0.25 mmol, 1.0 eq.) and nickel (II) chloride (1.6 mg, 0.012 mmol, 0.05 eq.) were suspended in *abs*. ethanol (4.9 mL) in a 25 mL round-bottom flask under N_2 atmosphere. The red suspension was cooled to 0 °C in an ice-bath and sodium borohydride (56 mg, 1.5 mmol, 6.0 eq.) was added. The reaction mixture was stirred for 10 minutes at 0 °C and then allowed to warm to room temperature; overtime the reaction mixture becomes a yellow suspension. After 2 hours, the reaction mixture is cooled to 0 °C and quenched with saturated aqueous NH_4Cl (10 mL). The mixture is diluted with H_2O (10 mL) and extracted with ethyl acetate (25 mL × 3). The combined organic layers were washed with brine (25 mL), dried over $MgSO_4$, filtered and concentrated under reduced pressure. The crude was solid loaded onto celite and purified by chromatography (12 g silica cartridge; CH_2Cl_2/ EtOAc 100:0 → 30:70) to give **21** as a yellow solid (61 mg, 64%). m.pt. 208.6-210.1 °C; R_f 0.23 (CH_2Cl_2/ EtOAc 2:1); ^{1}H-NMR (400 MHz, $CDCl_3$) δ 9.12 (2H, d, *J* = 11.6 Hz, H4,8), 6.62 (2H, dt, *J* = 11.7, 1.4 Hz, H5,7), 5.71 (2H, br s, C6-NH_2), 4.29 (4H, q, *J* = 7.1 Hz, -C<u>*H*</u>$_2$CH$_3$), 2.34 (6H, s, N($COCH_3$)$_2$), 1.37 (6H, t, *J* = 7.2 Hz, -CH$_2$C<u>*H*</u>$_3$) ppm; ^{13}C-NMR (101 MHz, $CDCl_3$) δ 173.94 (N<u>*C*</u>OCH$_3$), 164.69 (<u>*C*</u>OOEt), 162.60 (C6), 140.27 (C4,8), 140.14 (br, C2), 134.54 (C3a,8a), 118.11 (C5,7), 111.94 (C1,3), 60.16 (<u>*C*</u>H$_2$CH$_3$), 26.84 (NCO<u>*C*</u>H$_3$), 14.57 (CH$_2$<u>*C*</u>H$_3$) ppm; IR ν_{max} (film) 3437, 3349, 3239, 2978, 2933, 2901, 1720, 1689, 1660, 1625, 1602, 1574, 1464, 1445, 1425, 1382, 1348, 1290, 1248, 1206, 1169, 1125, 1038, 959, 942, 902, 854, 823, 775, 691, 649, 612 cm^{-1}; HRMS (ESI+) *m*/*z* calc for $[C_{20}H_{22}N_2O_6 + Na]^+$, 409.1370; found, 409.1370.

Diethyl 2-chloro-6-(pyrrolidin-1-yl)azulene-1,3-dicarboxylate 23

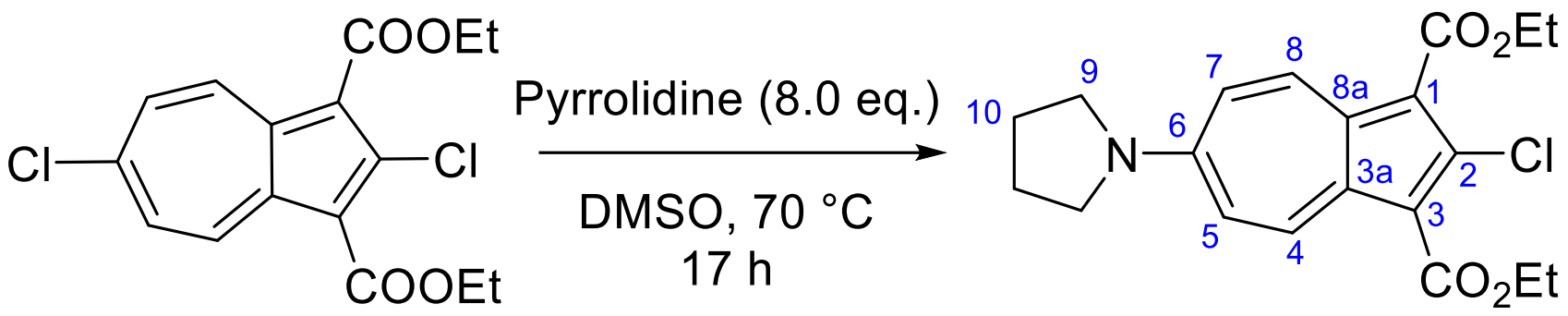

22 **23**

To diethyl 2,6-dichloroazulene-1,3-dicarboxylate **22**[9] (40 mg, 0.12 mmol, 1.0 eq.) in a 10 mL round-bottom flask, anhydrous DMSO (0.8 mL) was added under N_2 atmosphere to obtain a reddish/pinkish suspension. Pyrrolidine (78 µL, 0.94 mmol, 8.0 eq.) was added and the reaction mixture became a yellow solution, which was heated at 70 °C for 17 hours. The mixture was cooled at room temperature and diluted with H_2O (5 mL) and the mixture was extracted with ethyl acetate (7 mL × 3). The combined organic layers were washed with brine (10 mL), dried over $MgSO_4$, filtered and concentrated under reduced pressure. The crude was purified by chromatography (12 g silica cartridge; hexane/EtOAc 100:0 → 0:80) to give **23** as a yellow solid (31 mg, 69%). m.pt. degradation above 123.4 °C; R_f 0.36 (PE/EtOAc 1:1); ^{1}H-NMR (400 MHz, $CDCl_3$) δ 8.88 (2H, d, *J* = 12.0 Hz, H4,8), 6.53 (2H, d, *J* = 12.0 Hz, H5,7), 4.42 (4H, q, *J* = 7.1 Hz, -C<u>*H*</u>$_2$CH$_3$), 3.46-3.43 (2H, m, H9), 2.10-2.07 (2H, m, H10), 1.44 (6H, t, *J* = 7.1 Hz, -CH$_2$C<u>*H*</u>$_3$) ppm; ^{13}C-NMR (101 MHz, $CDCl_3$) δ 165.14 (<u>*C*</u>OOEt), 157.08 (C6), 137.31 (C4,8), 132.96 (C3a,8a), 132.74 (C2, 115.28 (C5,7), 113.71 (C1,3), 59.95 (<u>*C*</u>H$_2$CH$_3$), 49.41 (C9), 25.35 (C10), 14.55 (CH$_2$<u>*C*</u>H$_3$) ppm; IR ν_{max} (film) 2980, 2924, 2869, 1668, 1594, 1566, 1445, 1428, 1366, 1342, 1200, 1149, 1106, 1047, 1028, 889, 818, 777, 621 cm^{-1}; HRMS (ESI+) *m*/*z* calc for $[C_{20}H_{22}{}^{35}ClNO_4 + H]^+$, 376.1310; found, 376.1308.

Diethyl 6-bromoazulene-1,3-dicarboxylate 25

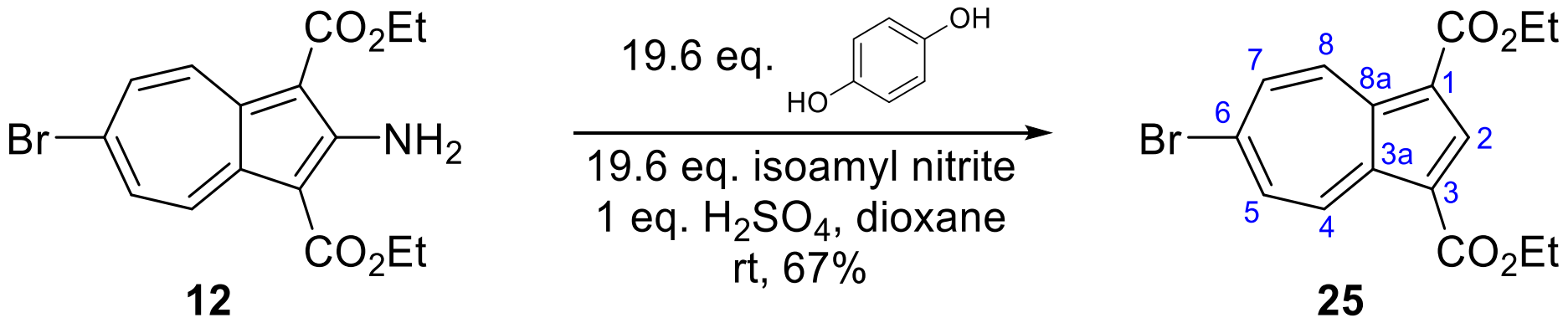

In a 3-necked 250 mL round-bottom flask, diethyl 2-amino-6-bromoazulene-1,3-dicarboxylate **12**[5] (732 mg, 2.0 mmol, 1.0 eq.) was dissolved in anhydrous THF (4 mL) under inert atmosphere. Sulfuric acid (0.1 mL, 2.0 mmol, 1.0 eq.) and *p*-hydroquinone (220 mg, 2.0 mmol, 1.0 eq.) were added in the flask and the mixture was diluted with anhydrous 1,4-dioxane (62 mL). A solution of *p*-hydroquinone (4.1 g, 37 mmol, 18.6 eq.) in 1,4-dioxane (39 mL) and a solution of isoamyl nitrite (5.3 mL, 39 mmol, 19.6 eq.) in 1,4-dioxane (39 mL) were added simultaneously via two dropping funnel over 20 minutes. The deep red solution was stirred at room temperature overnight; after this time, the reaction was quenched with 1 M Na_2SO_3 (100 mL) and stirred at room temperature for 30 minutes. The mixture was extracted with *n*-hexane (200 mL × 4), the combined organic layers were dried over $MgSO_4$, filtered and concentrated under reduced pressure. The crude was purified by chromatography on aluminium oxide basic (Brockmann I, 40-300 µm, 60 Å) eluted with toluene to give **25** as a red/pink solid (472 mg, 67%).; ^{1}H-NMR (400 MHz, $CDCl_3$) δ 9.52 (2H, d, *J* = 11.3 Hz, H4,8), 8.84 (1H, s, H2), 8.05 (2H, d, *J* = 11.3 Hz, H5,7), 4.44 (4H, q, *J* = 7.1 Hz, -C<u>*H*</u>$_2$CH$_3$), 1.45 (6H, t, *J* = 7.1 Hz, -CH$_2$C<u>*H*</u>$_3$) ppm; ^{13}C-NMR (101 MHz, $CDCl_3$) δ 164.85 (<u>*C*</u>OOEt), 143.80 (C2), 142.43 (C3a,8a), 138.71 (C6), 137.40 (C4,8), 133.93 (C5,7), 117.94 (C1,3), 60.45 (<u>*C*</u>H$_2$CH$_3$), 14.68 (CH$_2$<u>*C*</u>H$_3$) ppm; IR ν_{max} (film) 3067, 2958, 2924, 2853, 1682, 1569, 1529, 1480, 1428, 1372, 1347, 1183, 1102, 1079, 1036, 993, 838, 758, 654 cm^{-1}; HRMS (ESI+) *m/z* calc for $[C_{16}H_{15}{}^{79}BrO_4 + H]^+$, 351.0226; found, 351.0224. ^{1}H-NMR data in agreement with those previously reported.[10] However, ^{13}C-NMR data have not been reported previously.

Diethyl 6-hydroxyazulene-1,3-dicarboxylate 26

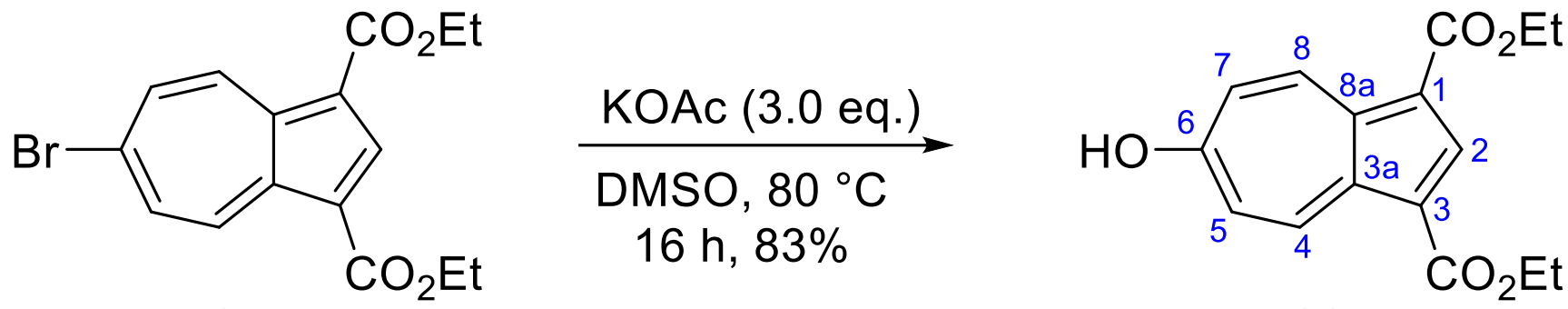

Diethyl 6-bromoazulene-1,3-dicarboxylate **25** (100 mg, 0.28 mmol, 1.0 eq.) and potassium acetate (82 mg, 0.84 mmol, 3.0 eq.) were weighted in a 50 mL round-bottom flask. The flask was equipped with a condenser, and the system was purged with N_2. Anhydrous DMSO (2 mL) was added and the reaction mixture was heated at 80 °C for 16 hours. The mixture was cooled at room temperature and diluted with H_2O (6 mL); the resulting yellow suspension was stirred at room temperature for 30 minutes. The mixture was extracted with ethyl acetate (10 mL × 3), and the combined organic layers were washed with brine (10 mL), dried over $MgSO_4$, filtered and concentrated under reduced pressure. The crude was purified by chromatography (12 g silica cartridge; hexane/EtOAc 100:0 → 0:100 with 2% of toluene) to give **26** as an orange solid (67 mg, 83%). m.pt.159.5-165.6 °C (acetonitrile) (lit.[11] 201-201.5 °C; EtOH/H_2O); R_f 0.17 (PE/EtOAc 1:1); ^{1}H-NMR (400 MHz, acetone-d_6) δ 9.59 (2H, app dt, J = 11.4, 1.4 Hz, H4,8), 8.39 (1H, s, H2), 7.45 (2H, app dt, J = 11.4, 1.3 Hz, H5,7), 4.36 (4H, q, J = 7.1 Hz, -C<u>*H*</u>$_2$CH$_3$), 1.40 (6H, t, J = 7.1 Hz, -CH$_2$C<u>*H*</u>$_3$) ppm; ^{13}C-NMR (126 MHz, acetone-d_6) δ 170.82 (C6), 165.41 (<u>*C*</u>OOEt), 140.19 (C4,8), 139.50 (C3a,8a), 138.30 (C2), 120.20 (C5,7), 116.97 (C1,3), 60.21 (<u>*C*</u>H$_2$CH$_3$), 14.87 (CH$_2$<u>*C*</u>H$_3$) ppm. IR ν_{max} (film) 3152 (br), 2989, 1697, 1650, 1582, 1523, 1462, 1386, 1278, 1217, 1200, 1103, 1094, 1031, 845, 775 cm^{-1}; HRMS (ESI+) m/z calc for $[C_{16}H_{16}O_5 + H]^+$, 289.1071; found, 289.1069. ^{1}H-NMR are in partial agreement with those reported previously;[12] we speculate that the chemical shift of certain protons may be concentration-dependent. ^{13}C-NMR data have not been reported previously.

Diethyl 6-azidoazulene-1,3-dicarboxylate 27

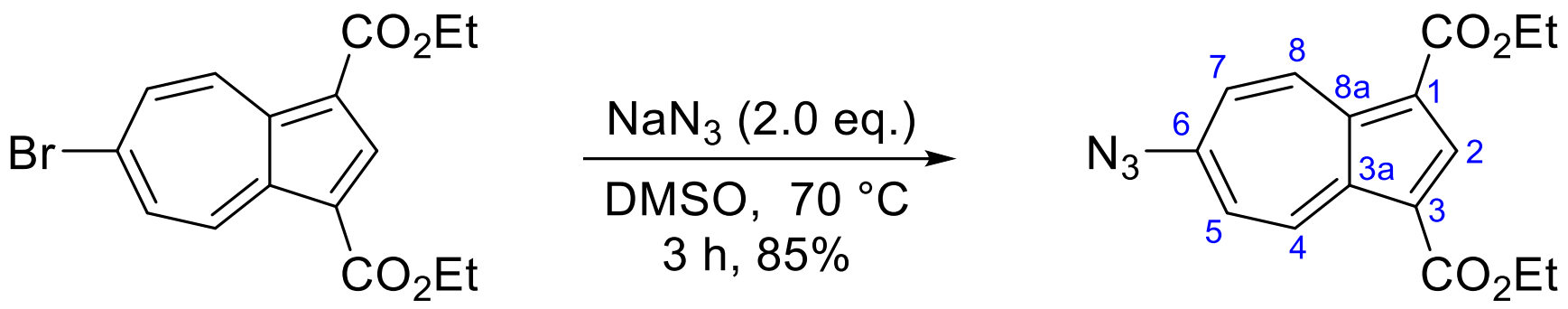

25 **27**

As per the reported procedure,[13] to diethyl 6-bromoazulene-1,3-dicarboxylate **25** (70 mg, 0.2 mmol, 1.0 eq.) and sodium azide (26 mg, 0.40 mmol, 2.0 eq.) were weighted in a 25 mL round-bottom flask, which was equipped with a condenser and the system was purged with N_2. Anhydrous DMSO (6.6 mL) was added and the reaction mixture was heated at 70 °C for 3 hours. The reaction mixture was cooled to room temperature and quenched with H_2O (10 mL). The mixture was extracted with ethyl acetate (15 mL × 3), and the combined organic layers were washed with brine (10 mL), dried over $MgSO_4$, filtered and concentrated under reduced pressure. The crude was re-dissolved in PE/EtOAc 1:1 (20 mL) and filtrated over a plug of aluminium oxide basic (Brockmann I, 40-300 μm, 60 Å). The solids were washed with PE/EtOAc 1:1 (100 mL), the first fraction of filtrate (colourless) was discarded, the red fraction of the filtrate was collected and the solvent was evaporated to give **27** as a red solid (53 mg, 85%). R_f 0.52 (PE/EtOAc 8:2); ^{1}H-NMR (400 MHz, $CDCl_3$) δ 9.68 (2H, d, *J* = 11.2 Hz, H4,8), 8.70 (1H, s, H2), 7.35 (2H, app dt, *J* = 11.4, 1.3 Hz, H5,7), 4.43 (4H, q, *J* = 7.1 Hz, -C$\underline{H}_2$CH$_3$), 1.45 (6H, t, *J* = 7.1 Hz, -CH$_2$C$\underline{H}_3$) ppm; ^{1}H-NMR are in partial agreement with those reported previously;[13] we speculate that the chemical shift of the aromatic protons may be concentration-dependent.

Diethyl 6-aminoazulene-1,3-dicarboxylate 28

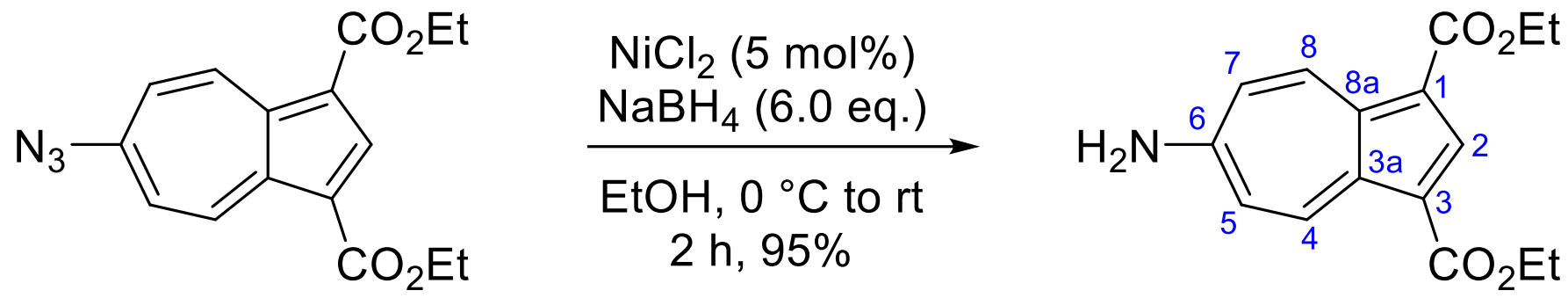

Diethyl 6-azidoazulene-1,3-dicarboxylate **27** (53 mg, 0.17 mmol, 1.0 eq.) and nickel (II) chloride (1 mg, 0.008 mmol, 0.05 eq.) were suspended in *abs*. ethanol (3.4 mL) in a 25 mL round-bottom flask under N_2 atmosphere. The red suspension was cooled to 0 °C in an ice-bath and sodium borohydride (38 mg, 1.0 mmol, 6.0 eq.) was added. The reaction mixture was stirred at 0 °C for 1 hour and then allowed to warm to room temperature. After 2 hours, the reaction mixture is cooled to 0 °C and quenched with saturated aqueous NH_4Cl (5 mL). The mixture is diluted with H_2O (5 mL) and extracted with ethyl acetate (10 mL × 3). The combined organic layers were washed with brine (10 mL), dried over $MgSO_4$, filtered and concentrated under reduced pressure. The crude was purified by chromatography (12 g silica cartridge; PE/ EtOAc 100:0 → 0:100) to give **28** as a yellow solid (46 mg, 95%). m.pt. 221.1-226.4 °C; R_f 0.22 (PE/EtOAc 1:1); ^{1}H-NMR (400 MHz, $CDCl_3$) δ 9.42 (2H, app dt, *J* = 11.4, 1.3 Hz, H4,8), 8.34 (1H, s, H2), 6.88 (2H, app dt, *J* = 11.3, 1.3 Hz, H5,7), 5.11 (2H, br s, $-NH_2$), 4.38 (4H, q, *J* = 7.1 Hz, $-C\underline{H_2}CH_3$), 1.42 (6H, t, *J* = 7.1 Hz, $-CH_2C\underline{H_3}$) ppm; ^{13}C-NMR (101 MHz, $CDCl_3$) δ 165.86 ($\underline{C}$OOEt), 159.46 (C6), 139.96 (C4,8), 137.41 (C3a,8a), 136.35 (C2), 116.61 (C5,7), 116.25 (C1,3), 59.79 ($\underline{C}H_2CH_3$), 14.76 ($CH_2\underline{C}H_3$) ppm. ^{1}H-NMR data have been reported previously, but in a different solvent, along with HRMS data.[14] IR and UV/vis data have been reported previously.[6] However, ^{13}C-NMR data have not been reported previously.

Diethyl 6-azido-2-hydroxyazulene-1,3-dicarboxylate 30 – *from 29*

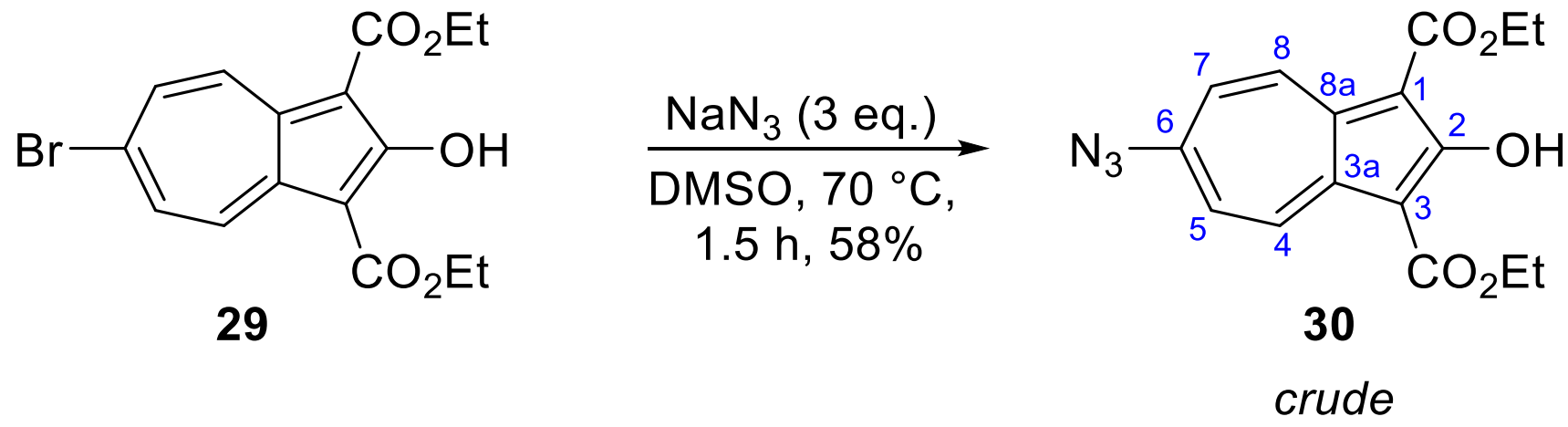

To diethyl 6-bromo-2-hydroxyazulene-1,3-dicarboxylate **29** (100 mg, 0.27 mmol, 1.0 eq.) in a 25 mL round bottom flask, sodium azide (58 mg, 0.82 mmol, 3.0 eq.) was added. The flask was equipped with a condenser, and the system was purged with N_2. Anhydrous DMSO (9 mL) was added, and the resulting red solution was heated at 70 °C for 1.5 hours. The reaction mixture was cooled to room temperature and quenched with H_2O (20 mL). A yellow solid precipitated out and it was filtered over a Büchner funnel. The solid was washed with H_2O (40 mL) and dried in high vacuum to give **30** as a yellow/brown solid (52 mg, 58%). The crude material was used immediately in the next step without purification. ^{1}H-NMR (400 MHz, $CDCl_3$) δ 11.56 (1H, br s, OH), 9.28 (2H, br d, J = 11.2 Hz, H4,8), 7.34 (2H, d, J = 11.2 Hz, H5,7), 4.51 (4H, q, J = 7.1 Hz, -C$\underline{H}_2$CH$_3$), 1.49 (6H, t, J = 7.1 Hz, -CH$_2$C$\underline{H}_3$) ppm; IR ν_{max} (film) 2983, 2109, 1680, 1629, 1587, 1542, 1475, 1432, 1347, 1289, 1273, 1233, 1184, 1130, 1092, 1030, 945, 856, 835, 796, 581 cm^{-1}; HRMS (ESI+) *m*/*z* calc for $[C_{16}H_{15}N_3O_5 + Na]^+$, 352.0904; found, 352.0902. ^{1}H-NMR data agree with those previously reported.[15]

Diethyl 2-acetoxy-6-bromoazulene-1,3-dicarboxylate 31

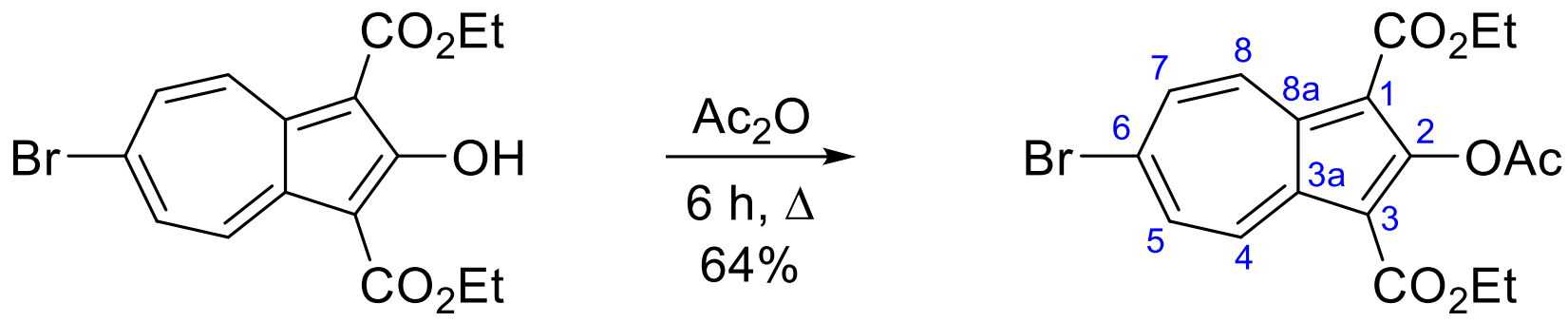

To diethyl 6-bromo-2-hydroxyazulene-1,3-dicarboxylate **29** (115 mg, 0.31 mmol) .) in a 25 mL round bottom flask, acetic anhydride (620 μL, to give a concentration of 0.5 M) was added, giving a yellow suspension. The flask was equipped with a condenser, and the system was purged with N_2, then heated at 140 °C for 6 hours, becoming a red solution over time. Th reaction mixture was concentrated under reduced pressure to remove acetic anhydride, and the crude material was then purified by chromatography (12 g silica cartridge; PE/EtOAc 95:5 → 20:80) to give **31** as an orange/red solid (81 mg, 64%). R_f 0.33 (PE/EtOAc 7:3); ^{1}H-NMR (500 MHz, $CDCl_3$) δ 9.48 (2H, d, *J* = 11.3 Hz, H4,8), 8.07 (2H, d, *J* = 11.3 Hz, H5,7), 4.41 (4H, q, *J* = 7.1 Hz, -C*H*$_2$CH$_3$), 2.43 (3H, s, OAc), 1.43 (6H, t, *J* = 7.1 Hz, -CH$_2$C*H*$_3$) ppm; ^{13}C-NMR (126 MHz, $CDCl_3$) δ 168.27 (O*C*OCH$_3$), 163.64 (*C*OOEt), 159.66 (C2), 140.33 (C3a,8a), 138.06 (C6), 137.13 (C4,8), 134.88 (C5,7), 109.79 (C1,3), 60.60 (*C*H$_2$CH$_3$), 21.15 (OCO*C*H$_3$), 14.57 (CH$_2$*C*H$_3$) ppm. IR ν_{max} (film) 3064, 2979, 1932, 2904, 2867, 1771, 1681, 1577, 1508, 1436, 1377, 1363, 1260, 1222, 1180, 1077, 1035, 998, 934, 882, 847, 780, 707, 630 cm^{-1}; HRMS (ESI+) *m/z* calc for $[C_{18}H_{17}{}^{81}BrO_6 + Na]^+$, 433.0081; found, 433.0082. The only data reported previously for this compound were the melting point and IR spectrum.[16]

Diethyl 6-azido-2-hydroxyazulene-1,3-dicarboxylate 30 – *from 31*

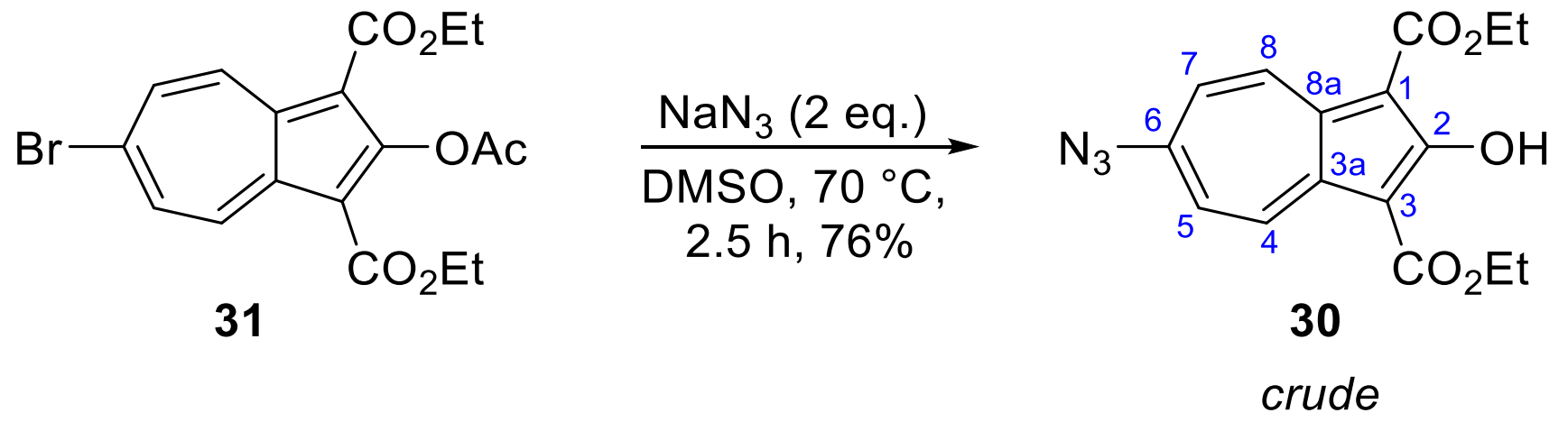

To diethyl 2-acetoxy-6-bromoazulene-1,3-dicarboxylate **31** (80 mg, 0.195 mmol, 1.0 eq.) in a 25 mL round bottom flask, sodium azide (25 mg, 0.391 mmol, 2.0 eq.) was added. The flask was equipped with a condenser, and the system was purged with N_2. Anhydrous DMSO (6.5 mL) was added, and the resulting red solution was heated at 70 °C for 2.5 hours. The reaction mixture was cooled to room temperature and quenched with H_2O (20 mL). The DMSO/water phase was extracted with EtOAc (4 x 25 mL), then the combined organic extracts were washed with brine (2 x 25 mL), dried over $MgSO_4$ and filtered. The filtrate was concentrated under reduced pressure, then dried in high vacuum to give **30** as a yellow/brown solid (49 mg, 76%). The crude material was used immediately in the next step without purification. Data were the same as given on page S24.

Diethyl 6-amino-2-hydroxyazulene-1,3-dicarboxylate 32

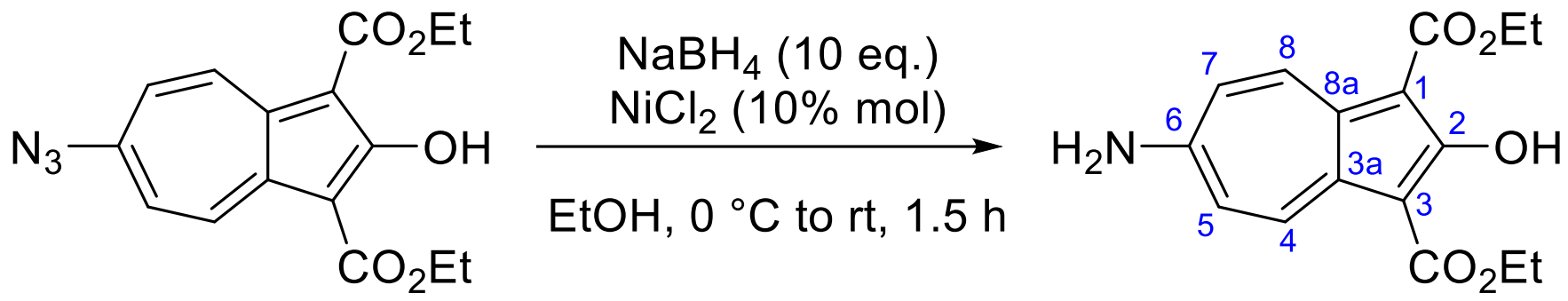

30 32

Diethyl 6-azido-2-hydroxyazulene-1,3-dicarboxylate **30** (52 mg, 0.16 mmol, 1.0 eq.) and nickel (II) chloride (2 mg, 0.016 mmol, 0.1 eq.) were introduced in a 25 mL round-bottom flask. The system was purged with N_2 and *abs*. ethanol (6 mL) was added. The resulting yellow suspension was cooled to 0 °C with an ice bath and sodium borohydride (60 mg, 1.6 mmol, 10.0 eq.) was added in one portion. The resulting red suspension was stirred at 0 °C for 5 minutes and then allowed to warm to room temperature. After 1.5 hours, the reaction mixture was quenched with saturated aqueous NH_4Cl (10 mL). The mixture is diluted with H_2O (5 mL) and extracted with ethyl acetate (20 mL × 3). The combined organic layers were washed with brine (25 mL), dried over $MgSO_4$, filtered and concentrated under reduced pressure. The crude was purified by chromatography (12 g silica cartridge; PE/EtOAc 100:0 → 0:100) to give **32** as an orange solid (41 mg, 86%) m.pt. 172.3 °C (dec.); R_f 0.39 (PE/EtOAc 1:2). ^{1}H-NMR (400 MHz, $CDCl_3$) δ 11.13 (1H, br s, OH), 9.10 (2H, app dt, *J* = 11.4, 1.3 Hz, H4,8), 6.93 (2H, app dt, *J* = 11.5, 1.3 Hz, H5,7), 4.80 (2H, br s, NH_2), 4.47 (4H, q, *J* = 7.1 Hz, -C$\underline{H}_2$CH$_3$), 1.46 (6H, t, *J* = 7.1 Hz, -CH$_2$C$\underline{H}_3$) ppm; ^{13}C-NMR (126 MHz, $CDCl_3$) δ 167.86 (C2), 167.19 ($\underline{C}$OOEt), 156.09 (C3a,8a), 136.41 (C4,8), 118.62 (C5,7), 101.07 (C1,3), 60.21 ($\underline{C}$H$_2$CH$_3$), 14.79 (CH$_2\underline{C}$H$_3$) ppm. The signal for C6 was not observed. IR ν_{max} (film) 3457, 3324, 3211, 2974, 1650, 1624, 1586, 1473, 1425, 1369, 1338, 1271, 1195, 1145, 1027, 955, 891, 796, 651, 616 cm^{-1}; HRMS (ESI–) *m*/*z* calc for $[C_{16}H_{17}NO_5 - H]^-$, 302.1034; found, 302.1032. ^{1}H-NMR data agree with those previously reported;[15] ^{13}C-NMR data were not reported previously.

UV/Vis spectra for selected compounds

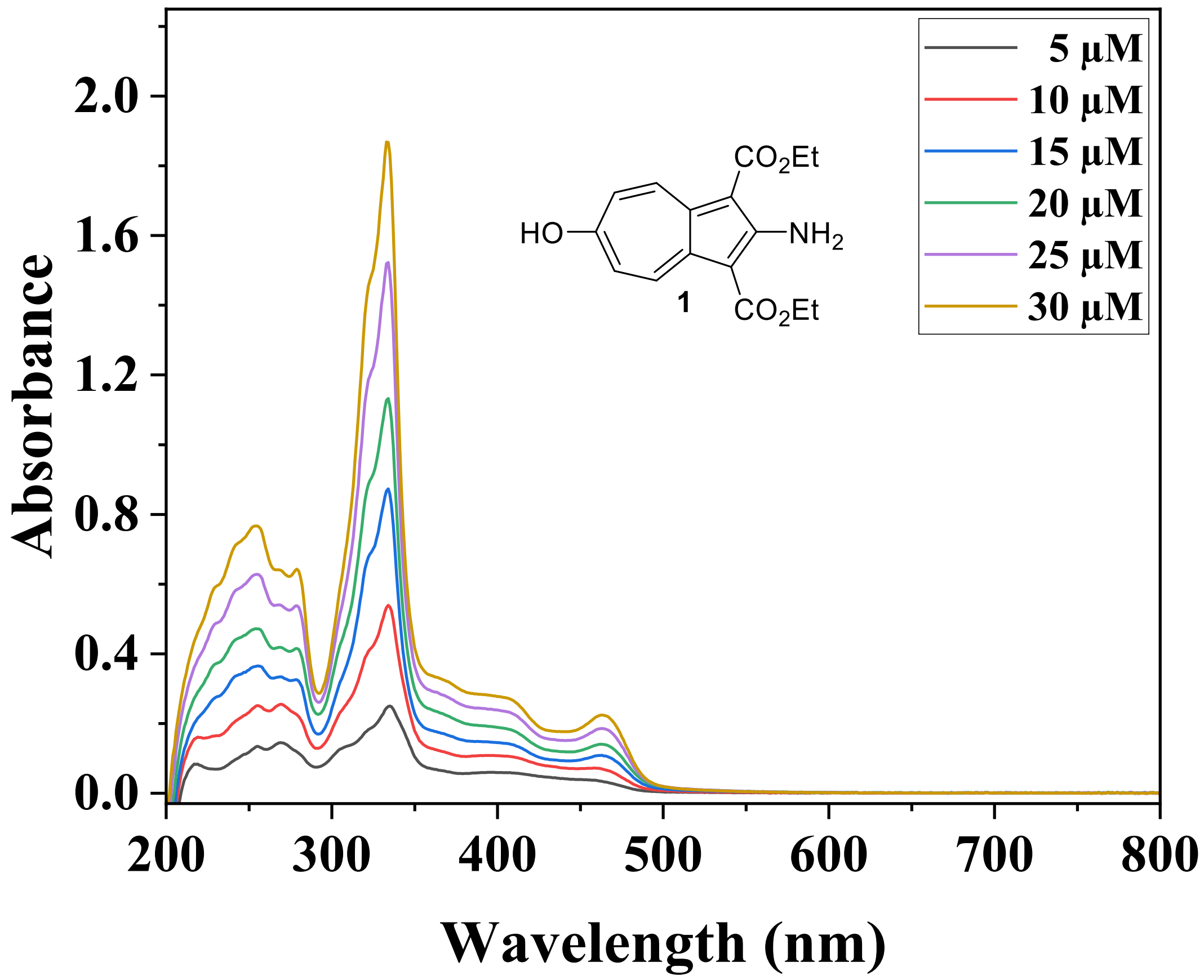

Absorbance spectra in the concentration range of 5 µM to 30 µM for **1**. Experimental conditions: 99.5% acetonitrile - 0.5% deionized water. All measurements were taken at room temperature (25°C) using an Agilent Technologies Cary 60 UV-Vis spectrometer.

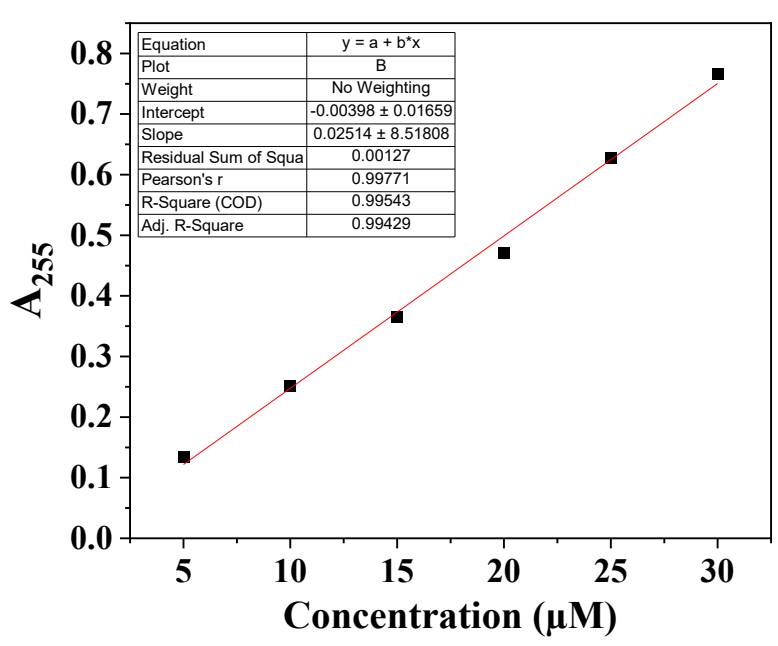

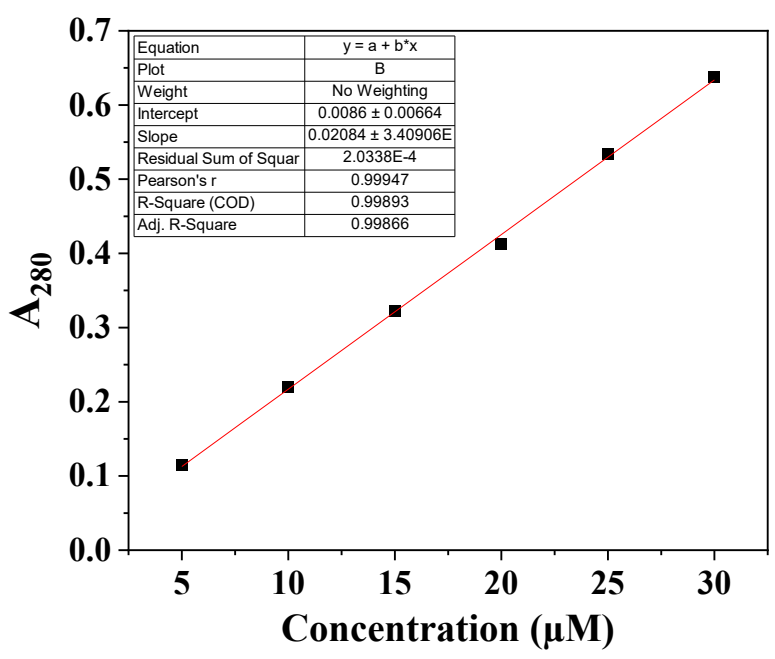

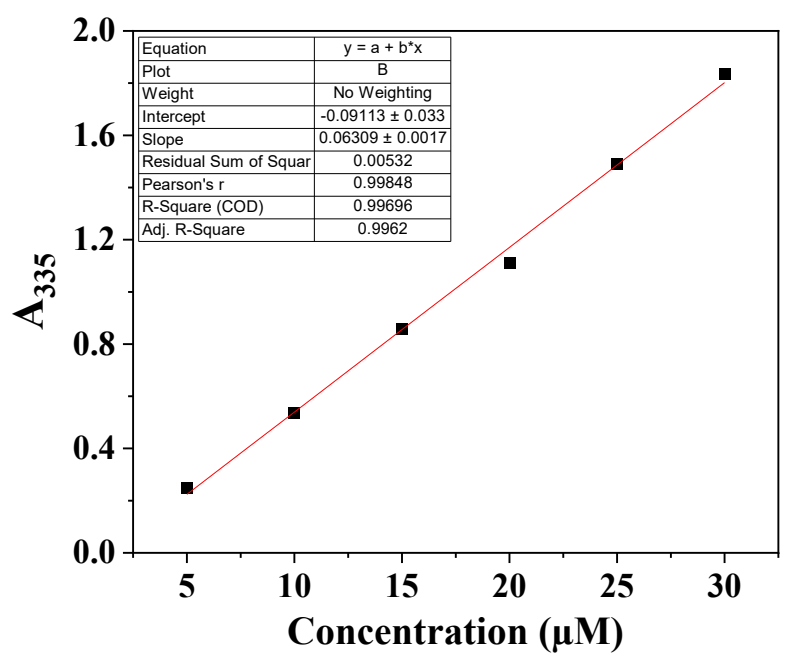

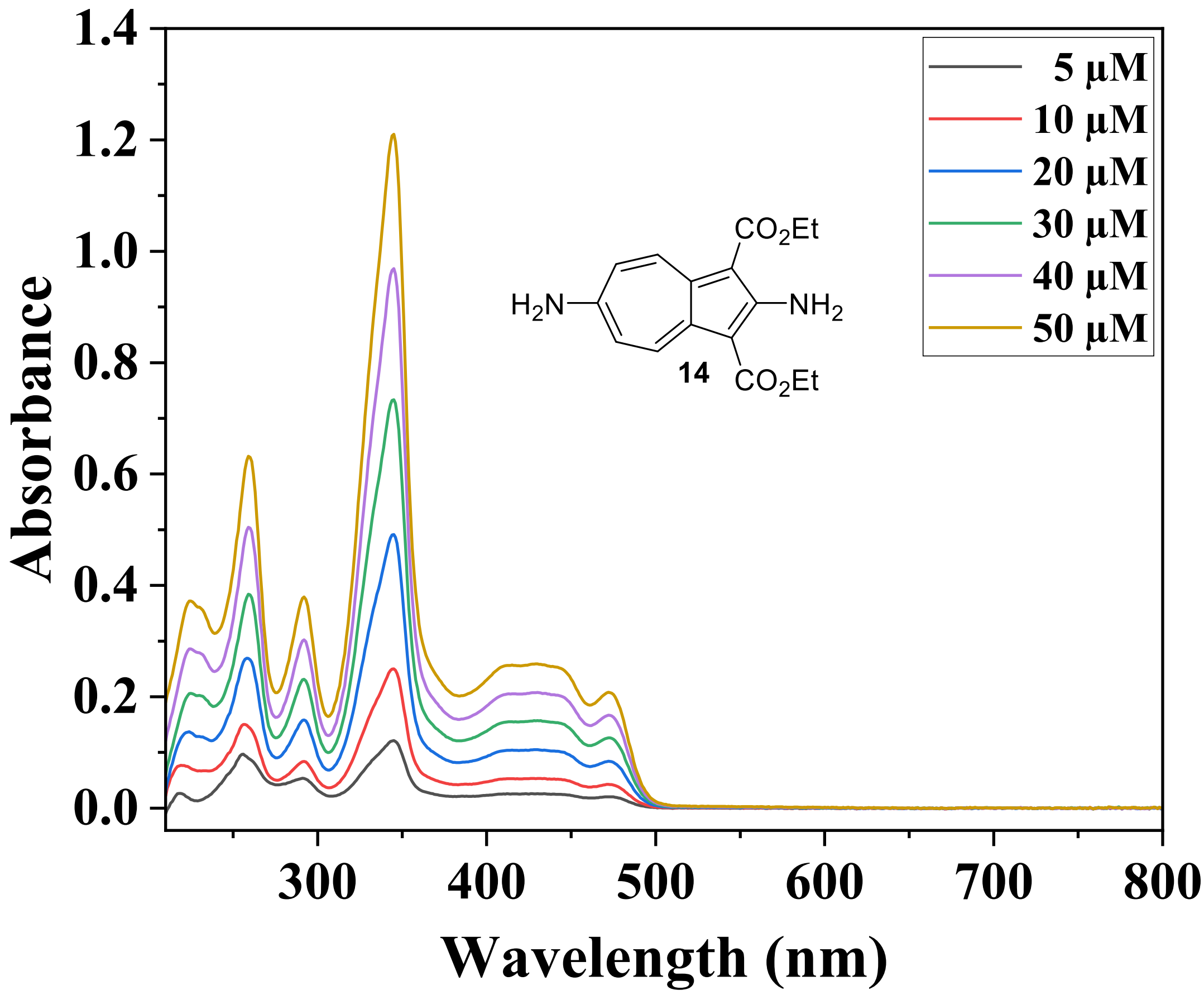

Absorbance spectra in the concentration range of 5 µM to 30 µM for **14**. Experimental conditions: 99.5% acetonitrile - 0.5% deionized water. All measurements were taken at room temperature (25°C) using an Agilent Technologies Cary 60 UV-Vis spectrometer.

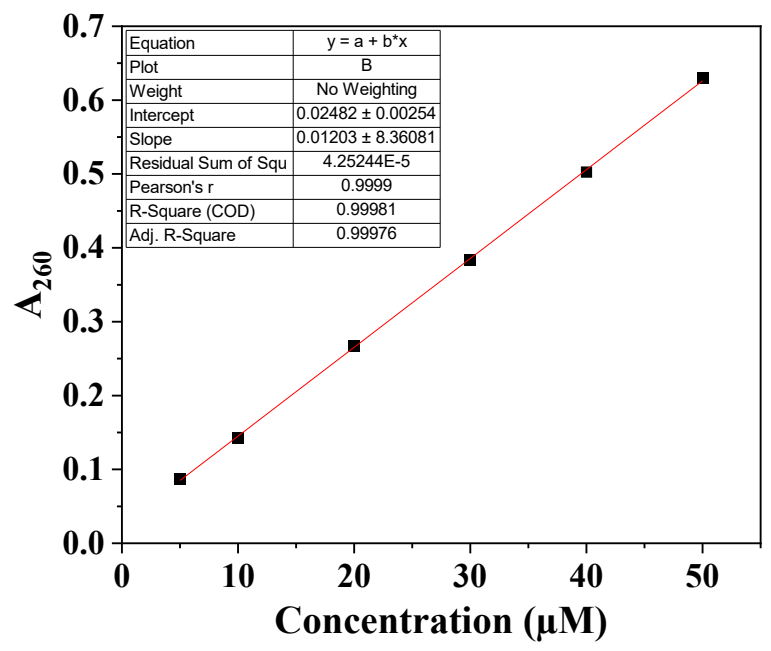

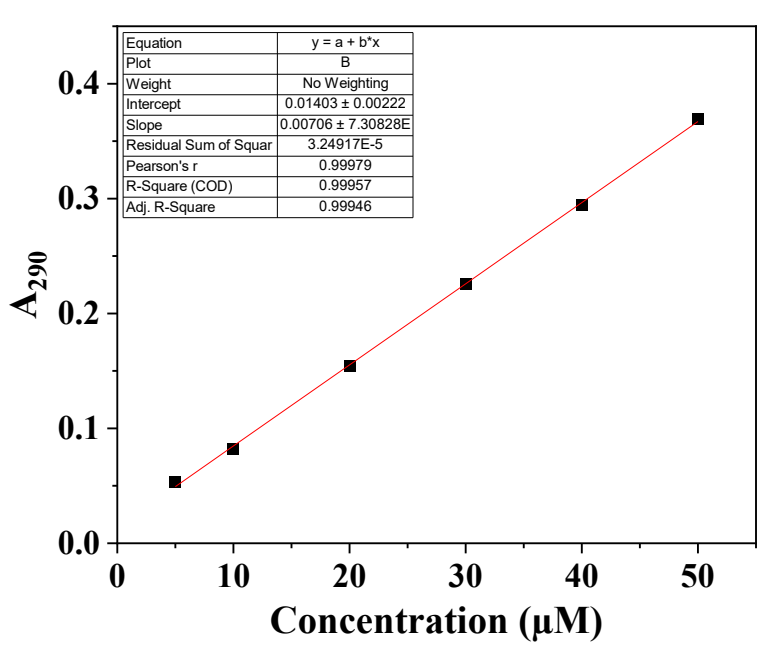

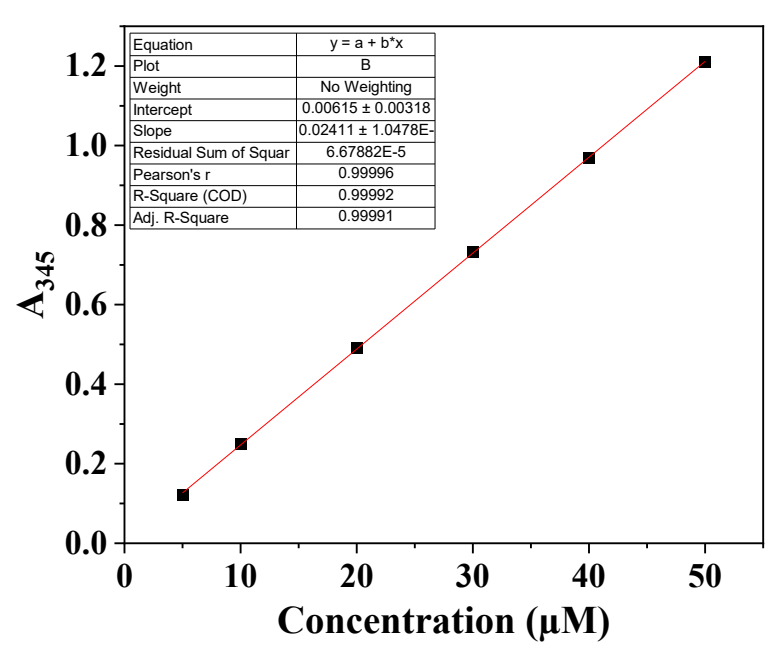

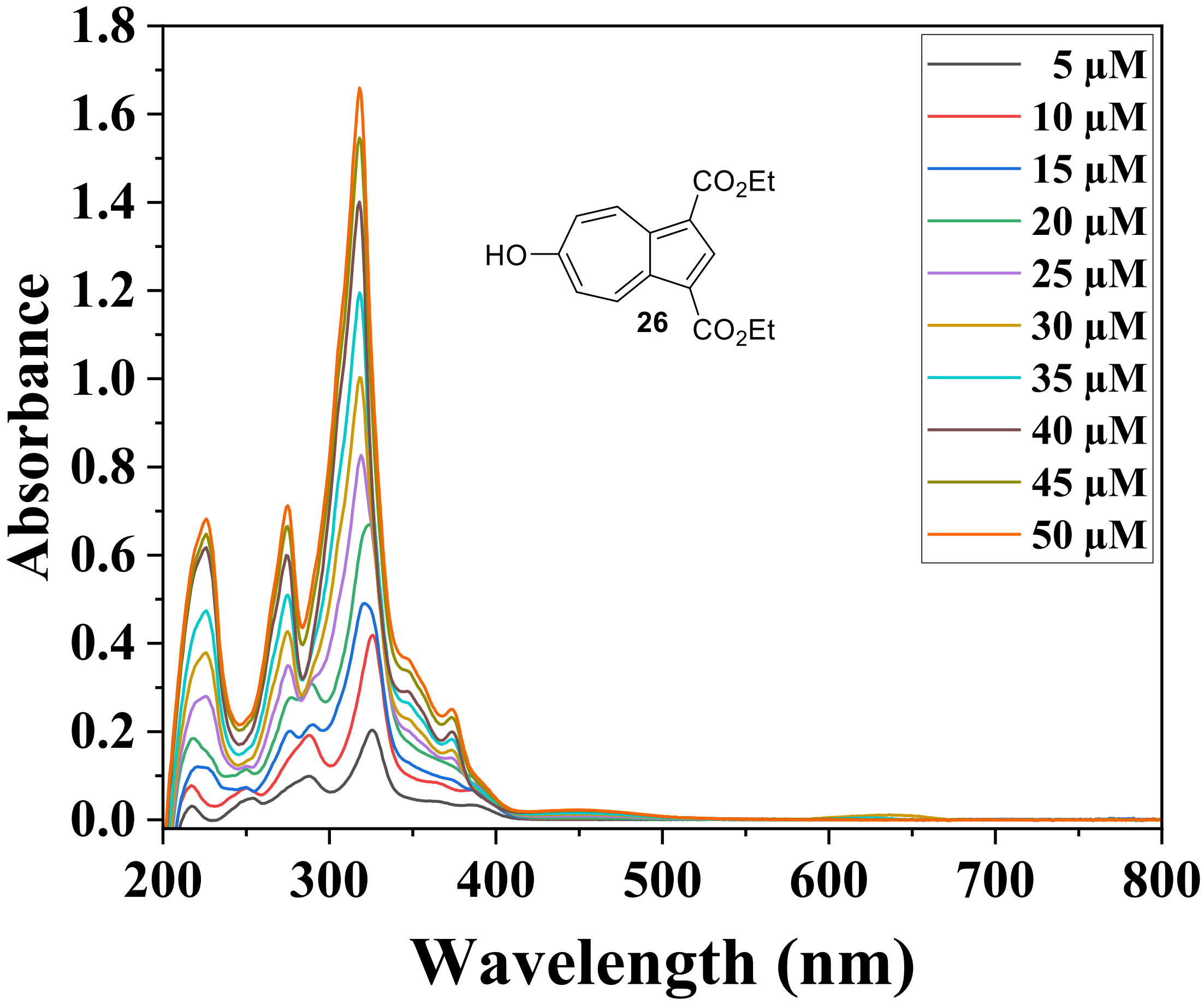

Absorbance spectra in the concentration range of 5 µM to 30 µM for **26**. Experimental conditions: 99.5% acetonitrile - 0.5% deionized water. All measurements were taken at room temperature (25°C) using an Agilent Technologies Cary 60 UV-Vis spectrometer.

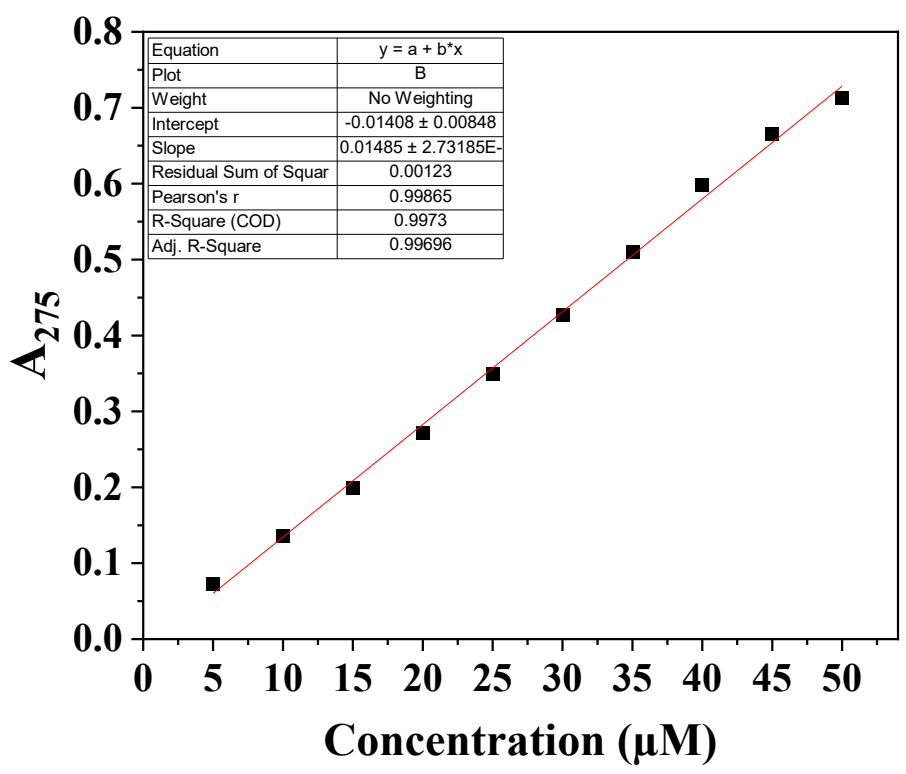

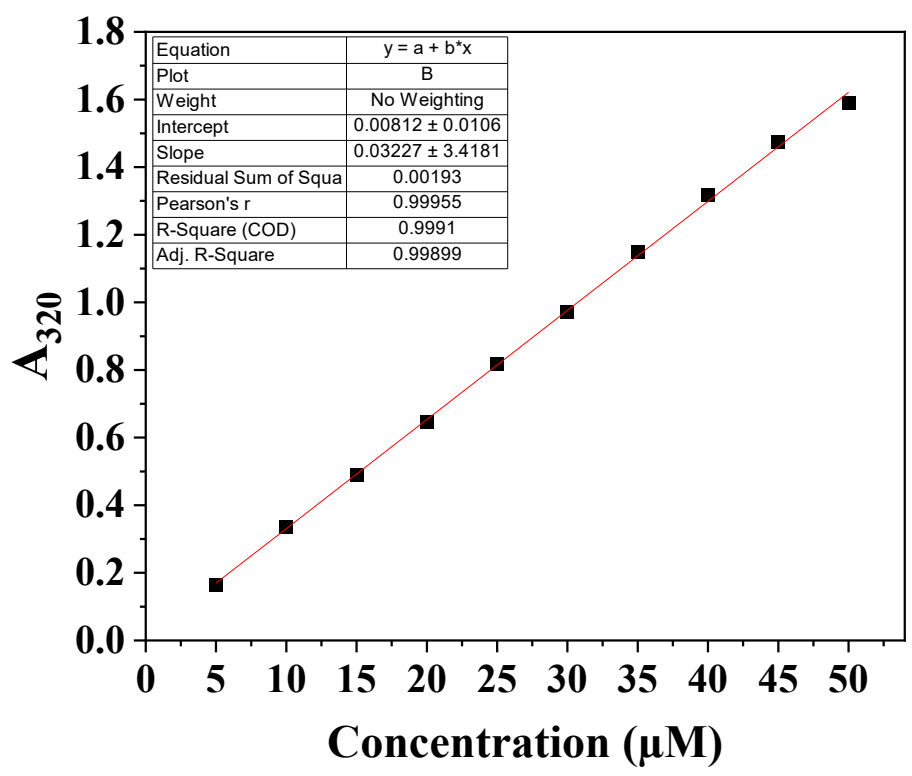

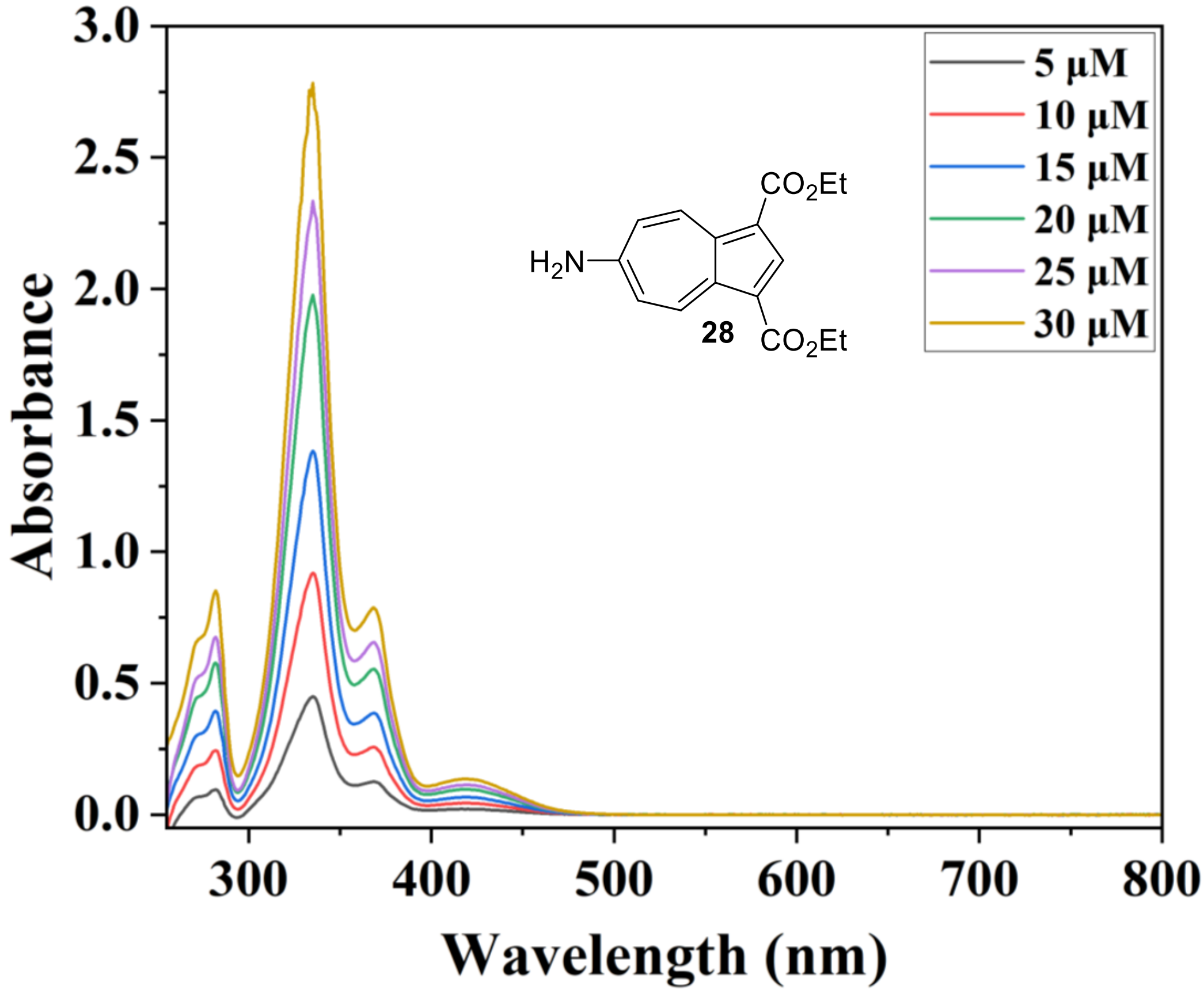

Absorbance spectra in the concentration range of 5 µM to 30 µM for **26**. Experimental conditions: 99.5% acetonitrile - 0.5% deionized water. All measurements were taken at room temperature (25°C) using an Agilent Technologies Cary 60 UV-Vis spectrometer.

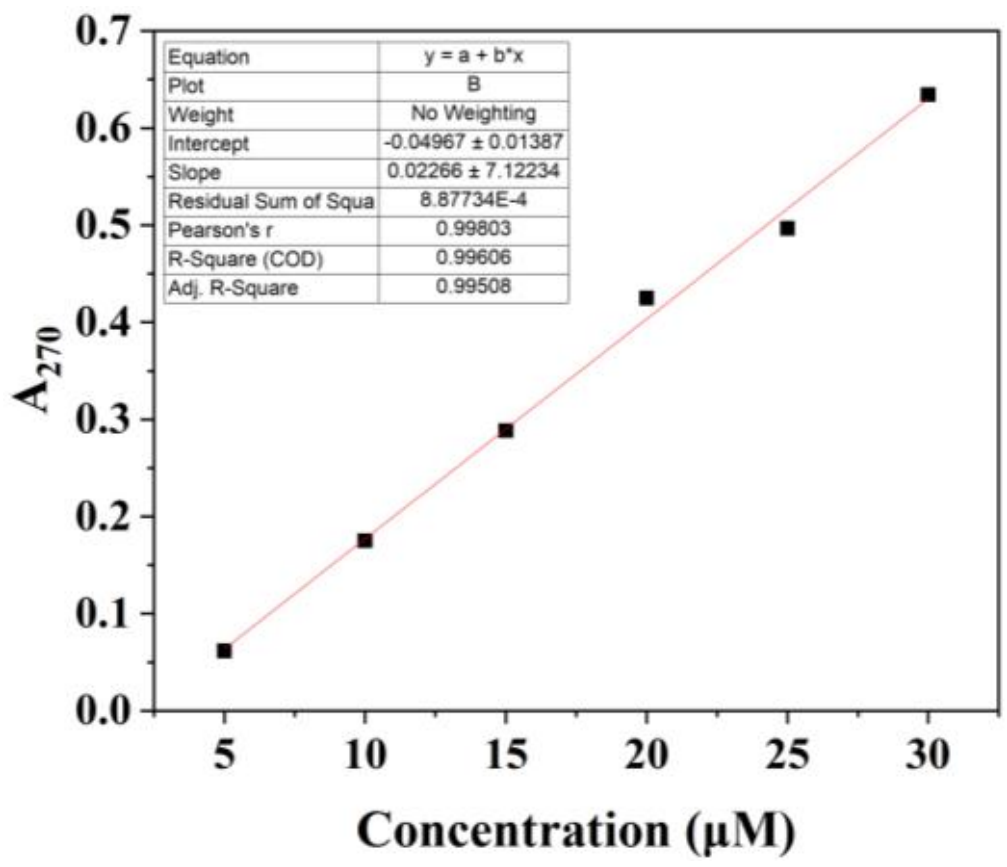

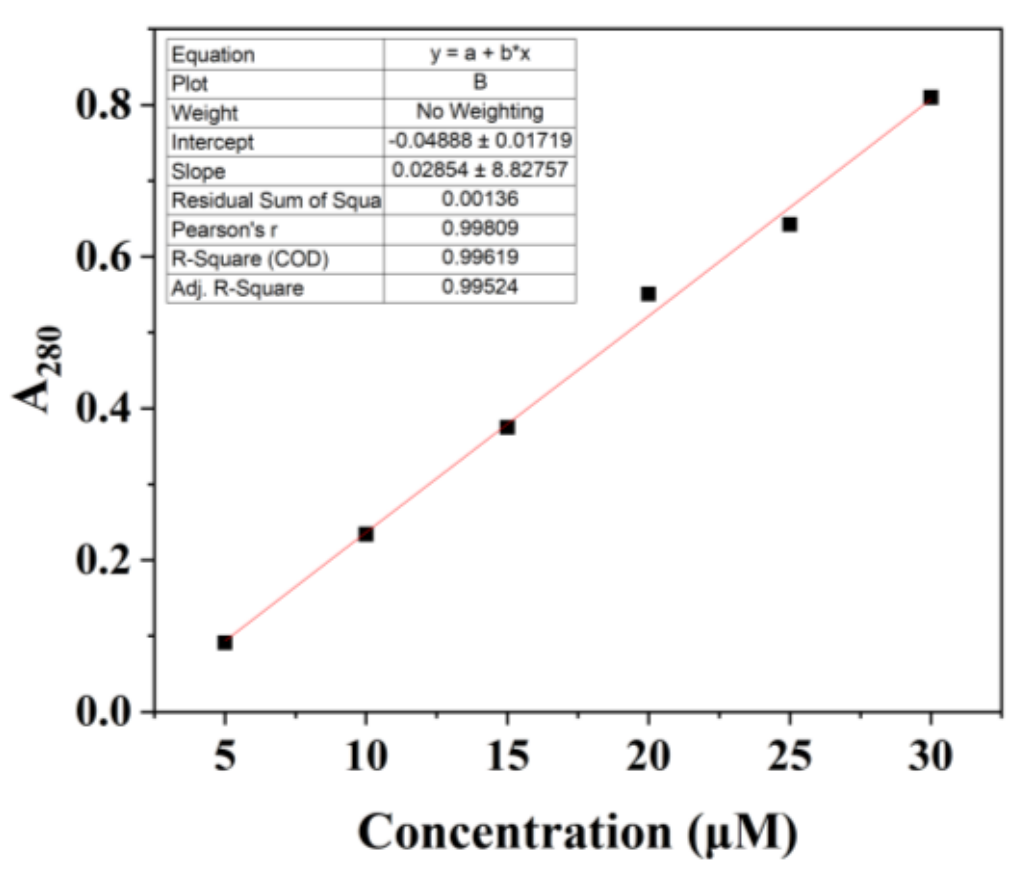

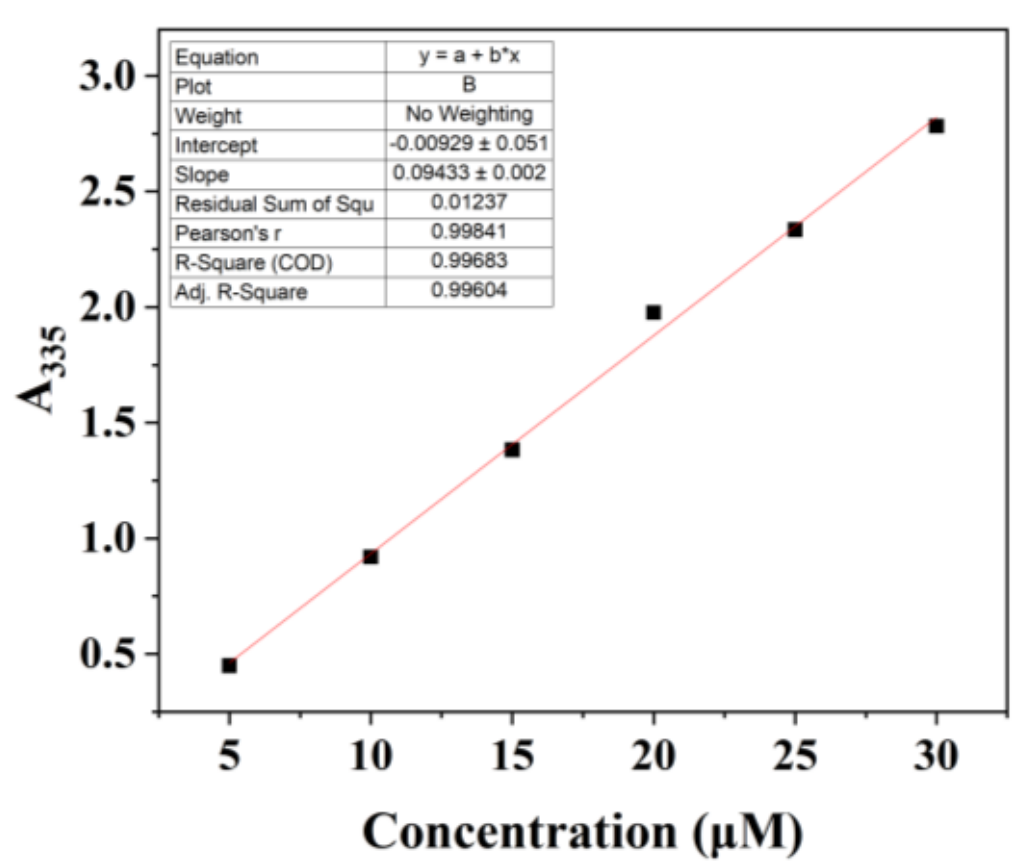

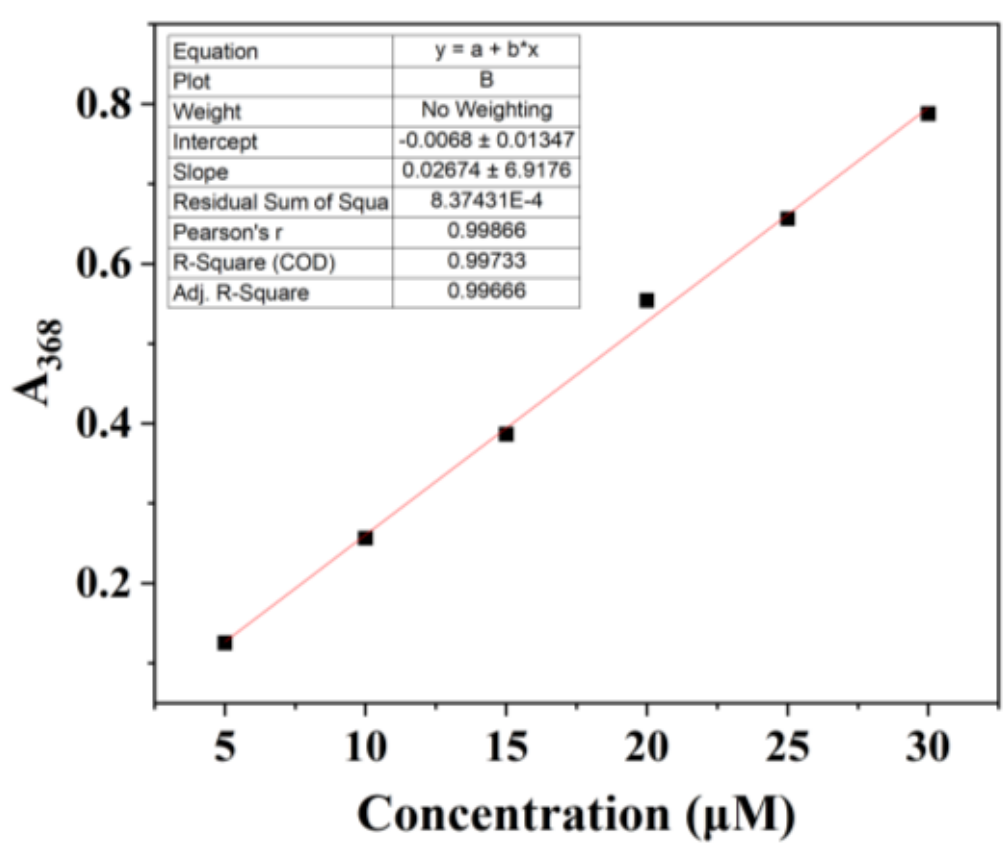

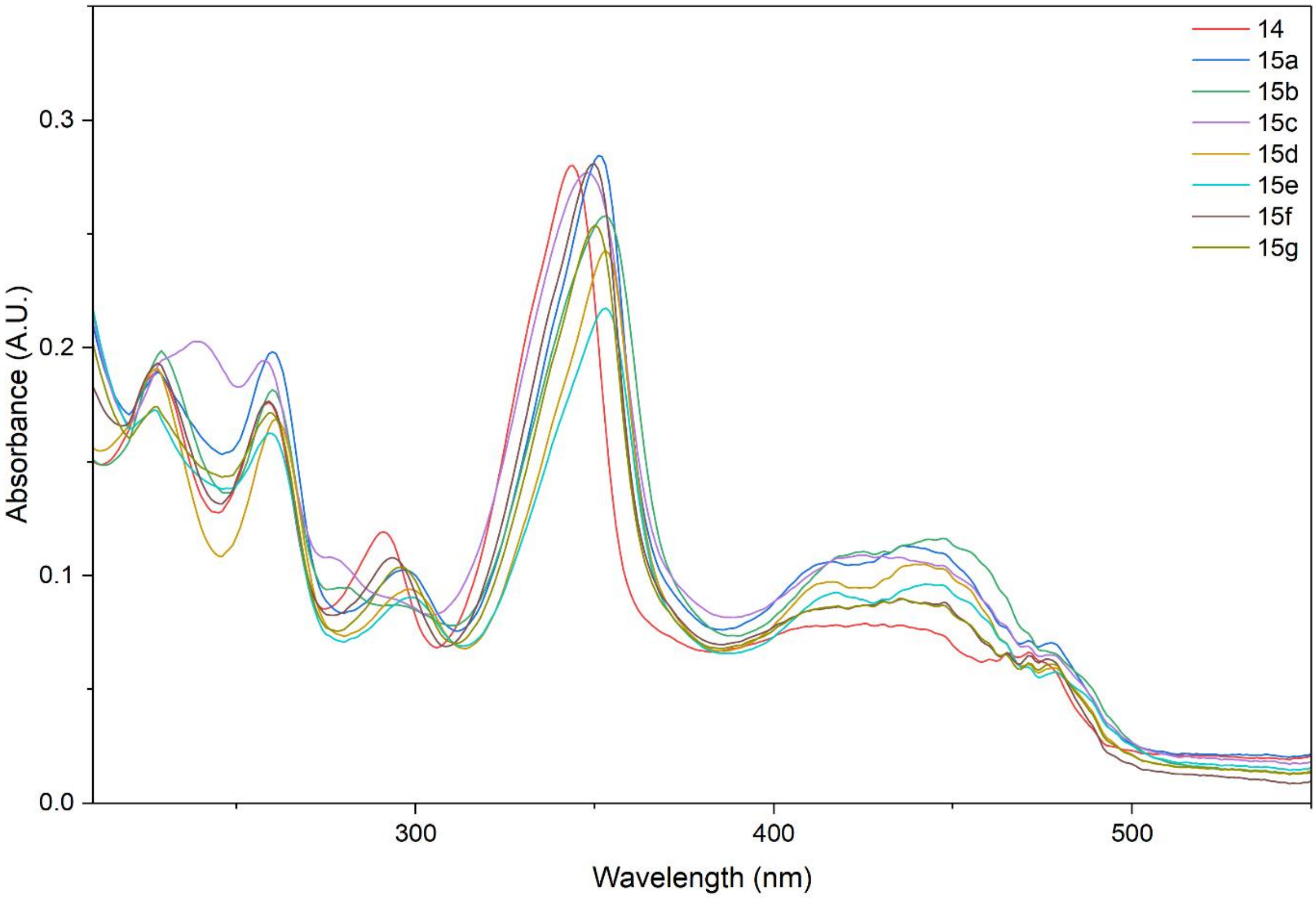

Overlaid absorbance spectra, concentration 5 μM for **14** and **15a-15g**. Experimental conditions: 99.5% acetonitrile - 0.5% deionized water. All measurements were taken at room temperature (25°C) using a Jasco J-810 spectrometer.

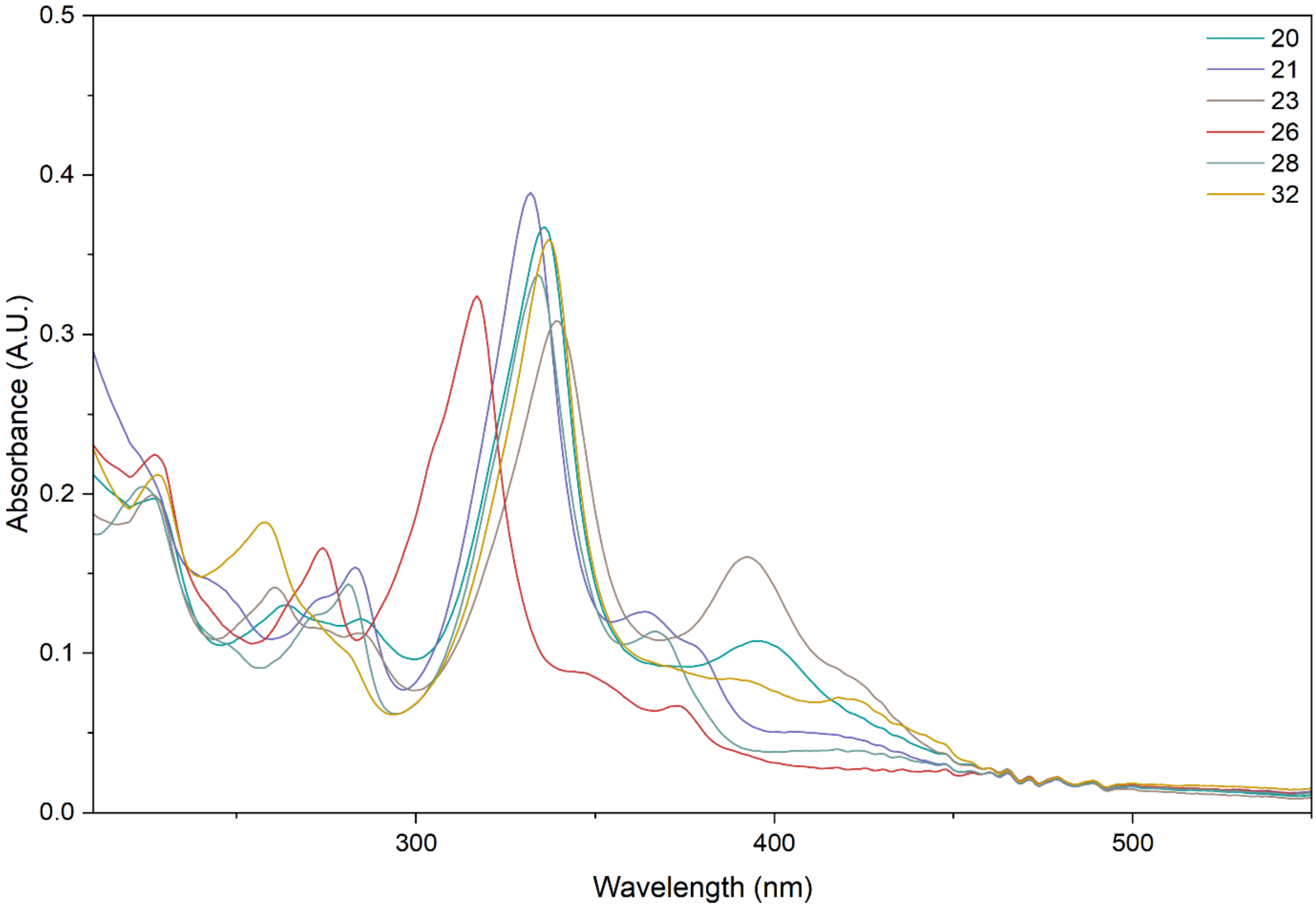

Overlaid absorbance spectra, concentration 5 μM for **20**, **21**, **23**, **26**, **28**, and **32**. Experimental conditions: 99.5% acetonitrile - 0.5% deionized water. All measurements were taken at room temperature (25°C) using a Jasco J-810 spectrometer.

Fluorescence Spectra (Halofluorochromism Studies)

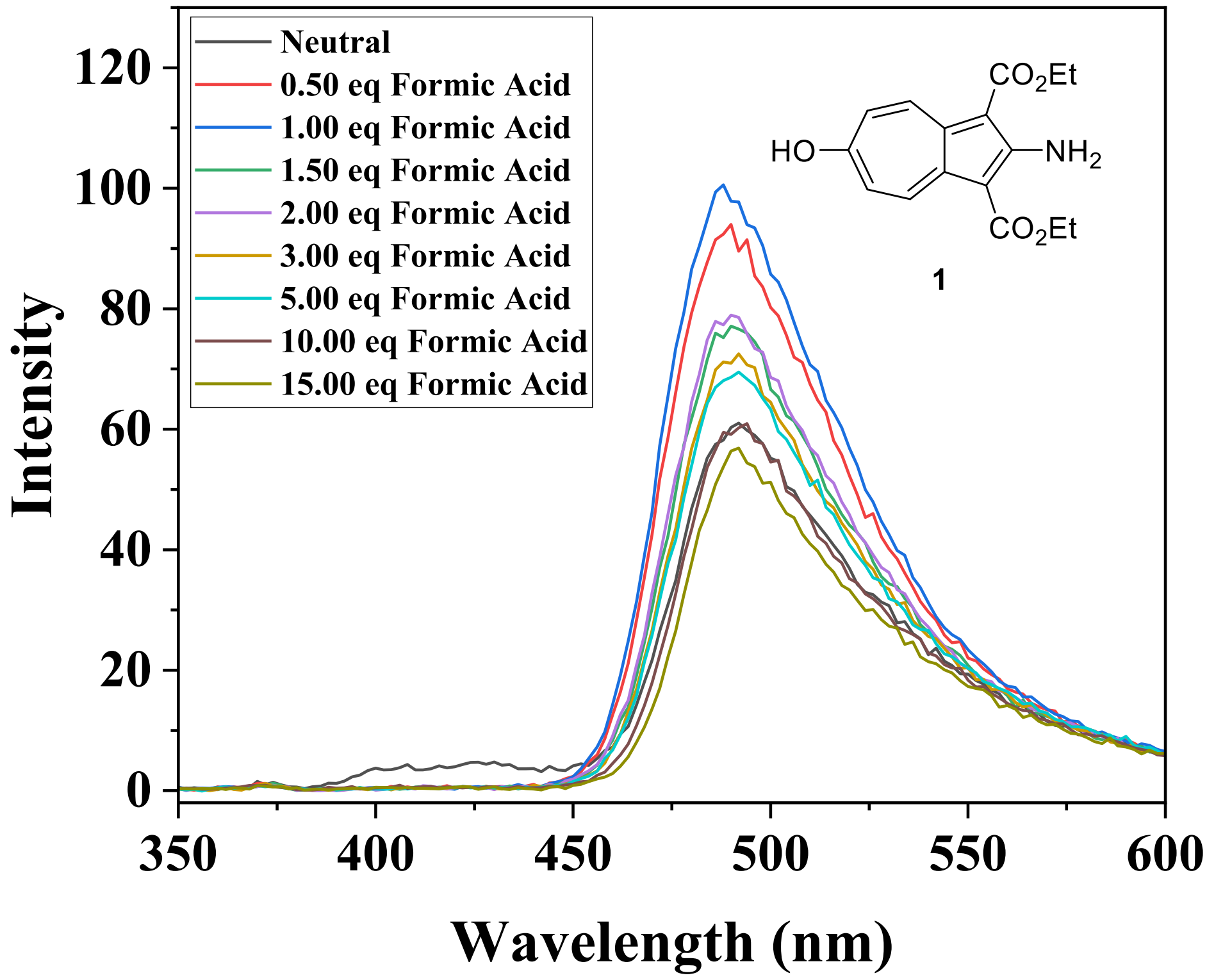

Fluorescence spectra of **1** (10 μM) in neutral conditions and with increasing quantities of formic acid. Experimental conditions: 99.5% acetonitrile - 0.5% water. Measurements were taken at room temperature (25°C) using an Agilent Technologies Cary Eclipse fluorimeter. Fluorescence intensities were measured with λ_{ex} = 335 nm (ex. slit: 5 nm, em. slit: 10 nm).

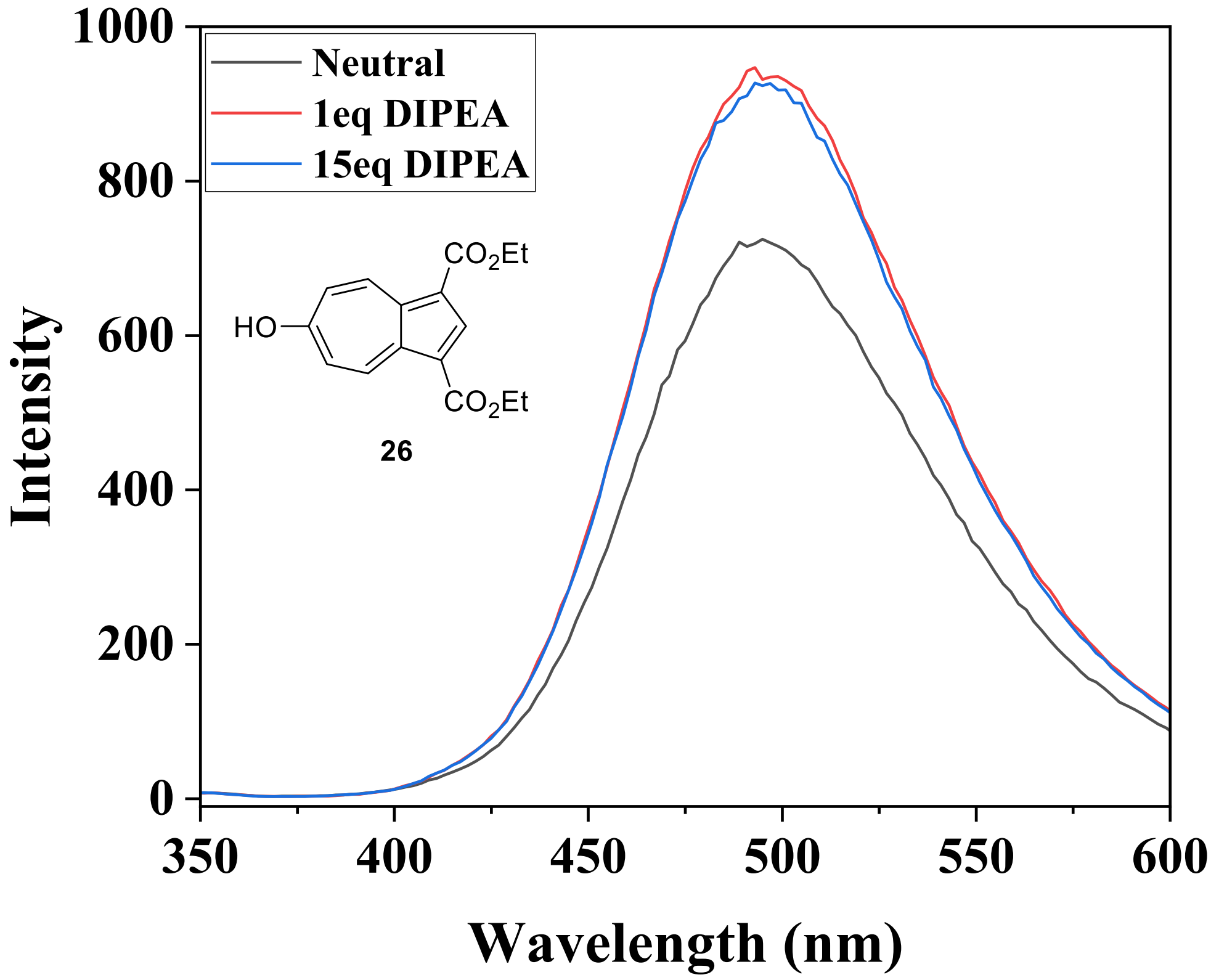

Fluorescence spectra of **26** (10 μM) in neutral conditions and with increasing quantities of DIPEA. Experimental conditions: 99.5% acetonitrile - 0.5% water. Measurements were taken at room temperature (25°C) using an Agilent Technologies Cary Eclipse fluorimeter. Fluorescence intensities were measured with λ_{ex} = 335 nm (ex. slit: 5 nm, em. slit: 10 nm).

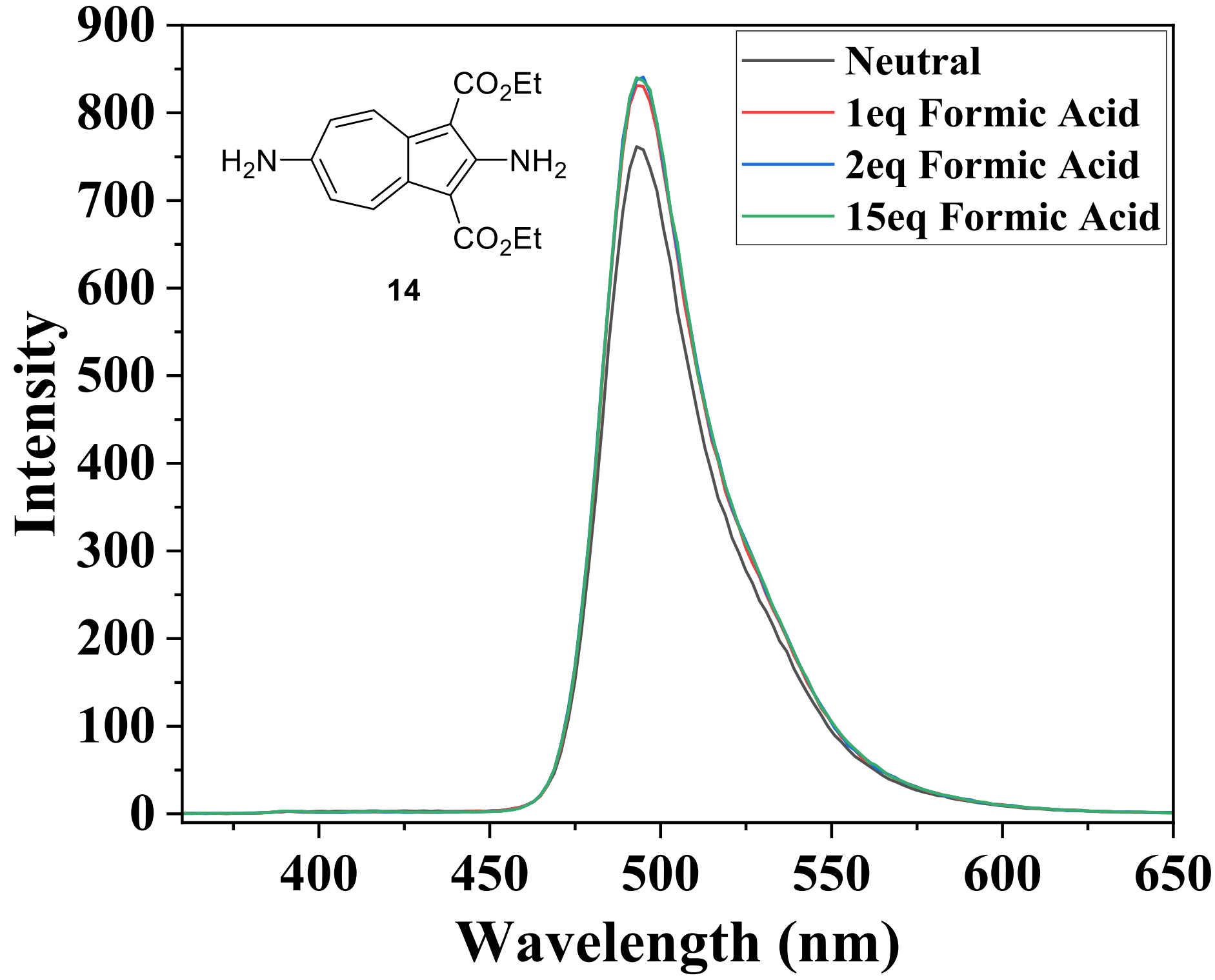

Fluorescence spectra of **14** (10 μM) in neutral conditions and with increasing quantities of formic acid. Experimental conditions: 99.5% acetonitrile - 0.5% water. Measurements were taken at room temperature (25°C) using an Agilent Technologies Cary Eclipse fluorimeter. Fluorescence intensities were measured with λ_{ex} = 335 nm (ex. slit: 5 nm, em. slit: 10 nm).

NMR/IR/Mass Spectra

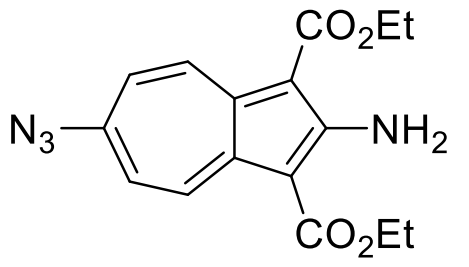

13

^{1}H-NMR, 400 MHz, $CDCl_3$

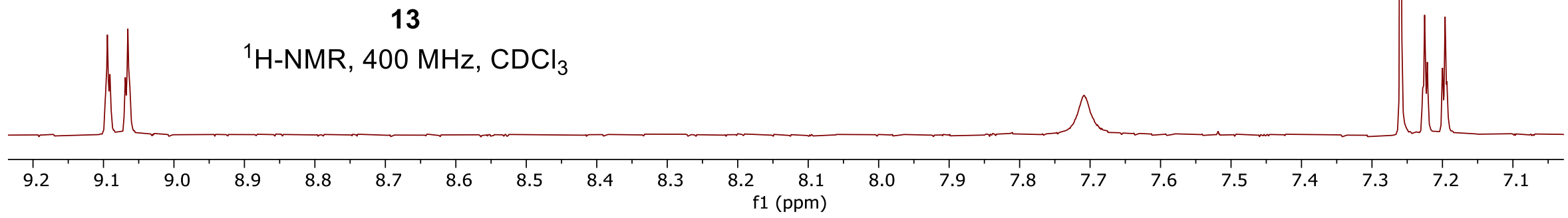

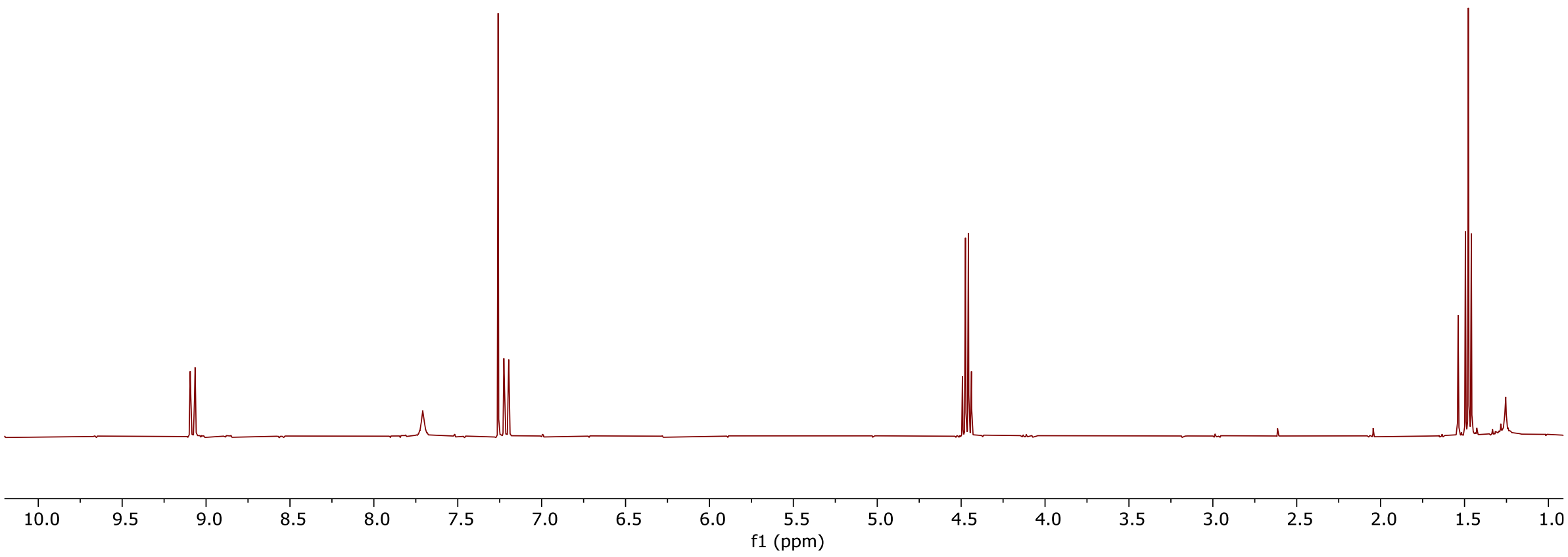

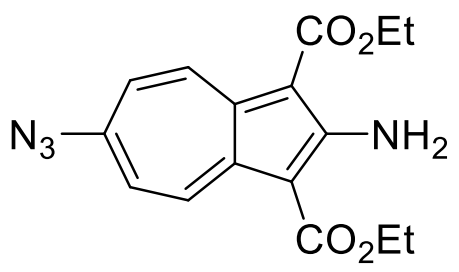

13

HRMS-ESI$^+$

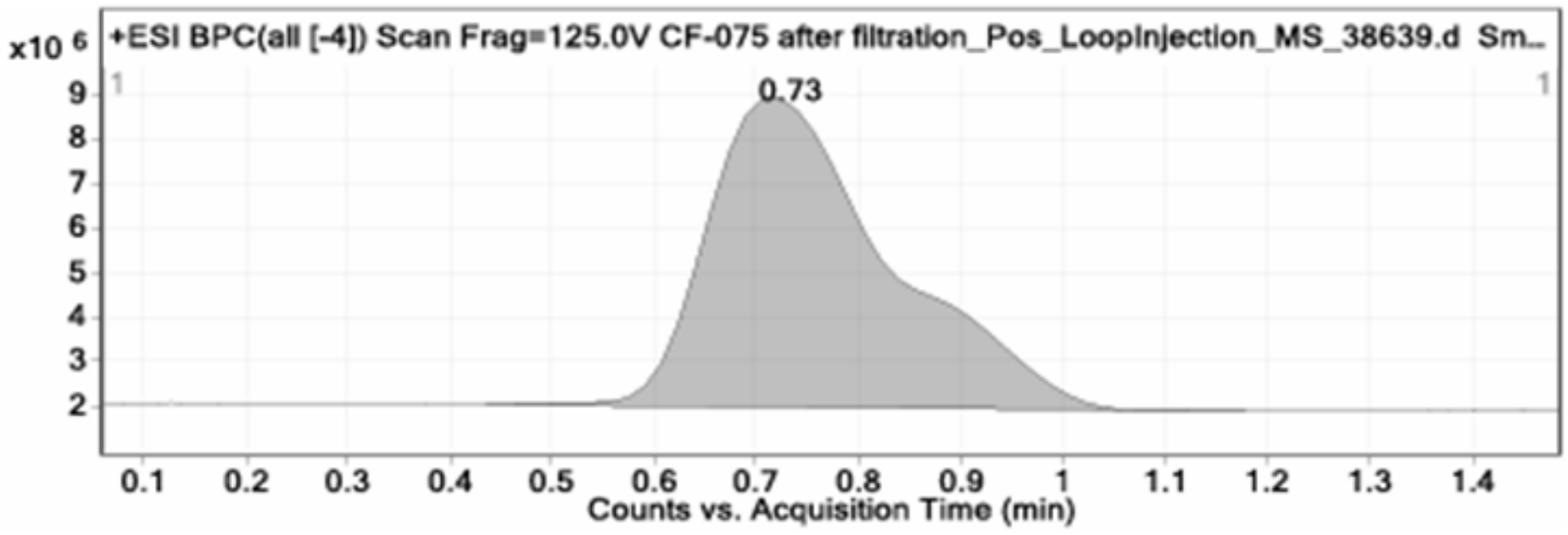

Figure 1: Base peak chromatogram

User Chromatogram Peak List

RT (min)	Area	Area %	Area Sum (%)	Base Peak (m/z)	Width (min)
0.73	86092029	100.00	100.00	355.0912	0.220

Compound Table

Compound Label	RT (min)	Observed mass (m/z)	Neutral observed mass (Da)	Theoretical mass (Da)	Mass error (ppm)	Isotope match score (%)
Cpd 1: C16 H16 N4 O4	0.83	329.1247	328.1176	328.1172	1.28	97.51

Mass errors of between -5.00 and 5.00 ppm with isotope match scores above 60% are considered confirmation of molecular formulae

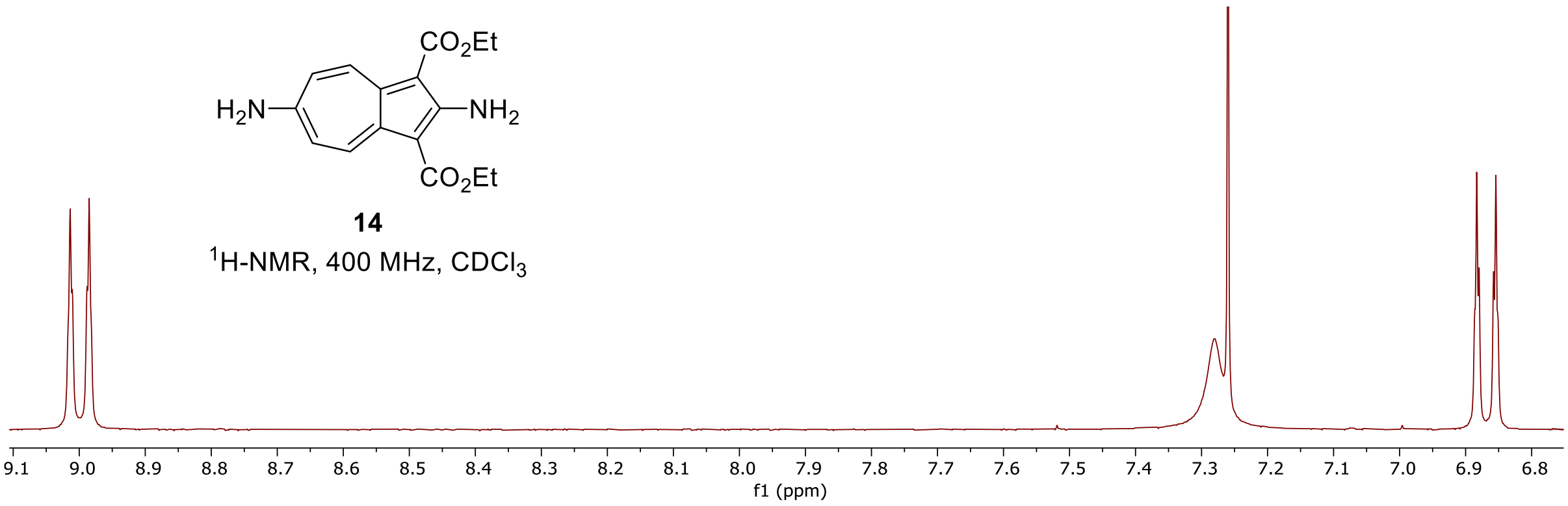
CO2Et
H2N
NH2
CO2Et
14
1H-NMR, 400 MHz, CDCl3
f1 (ppm)

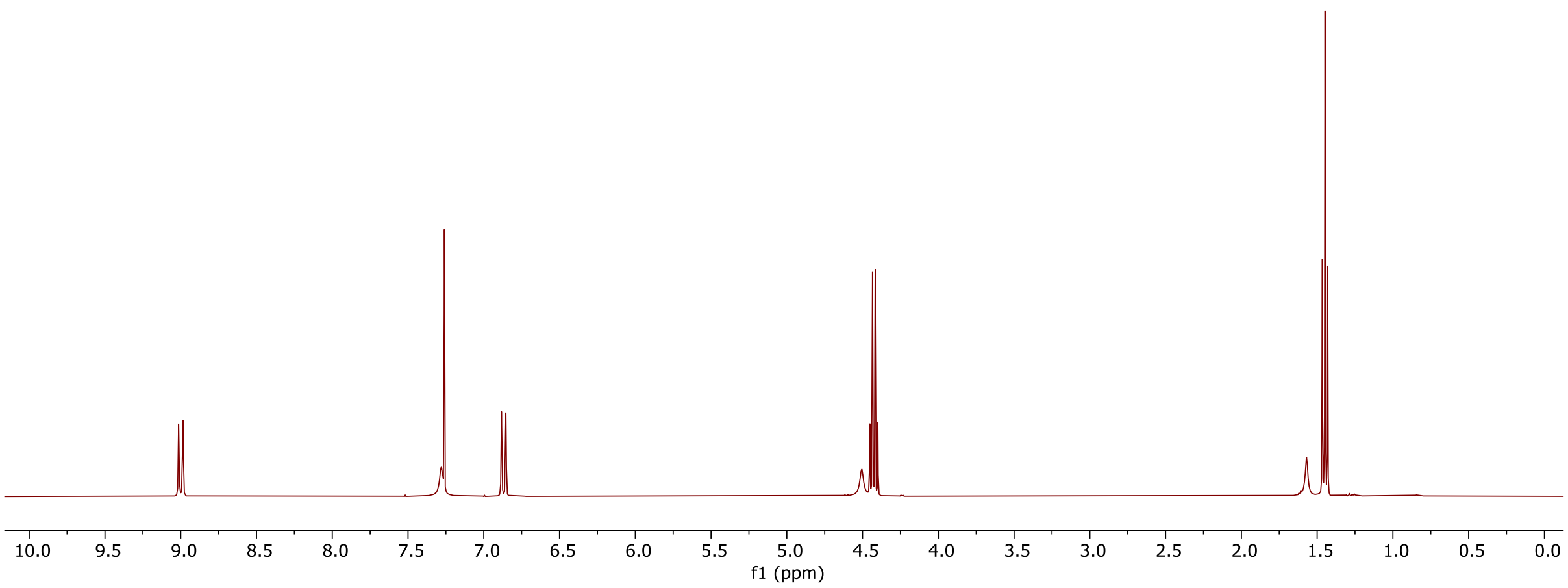
f1 (ppm)

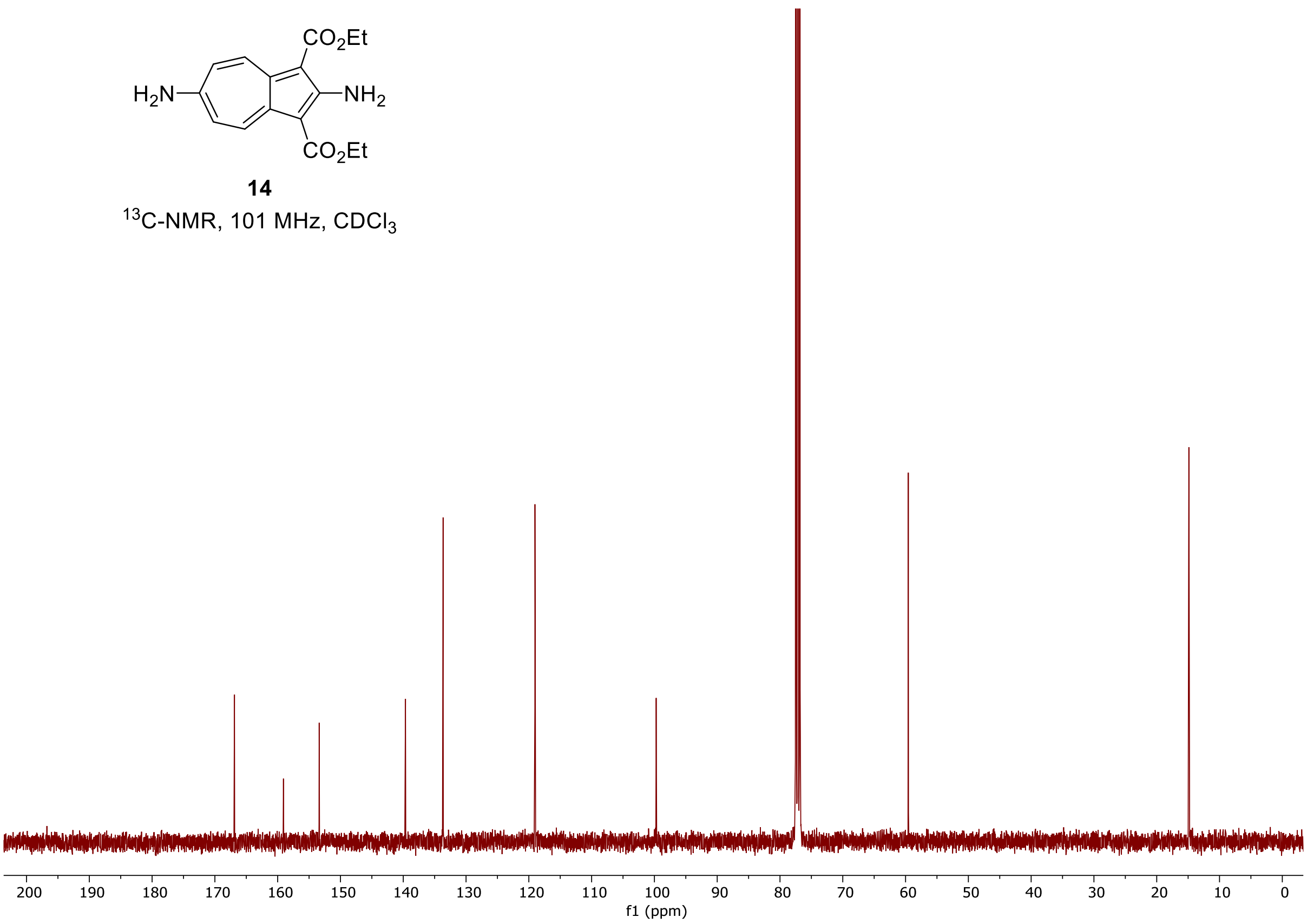
CO2Et
H2N
NH2
CO2Et
14
13C-NMR, 101 MHz, CDCl3
f1 (ppm)

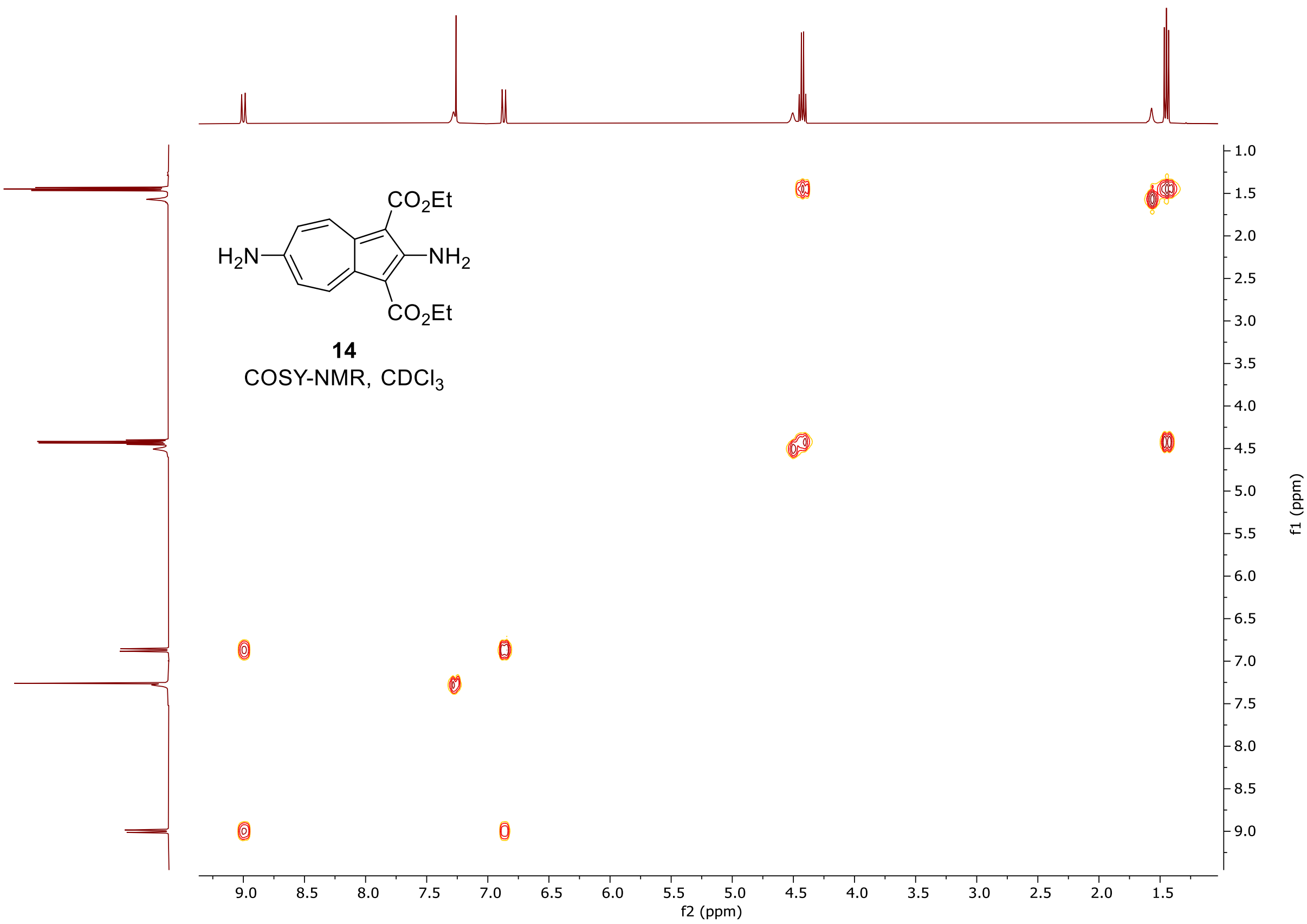
CO2Et
H2N
NH2
CO2Et
14
COSY-NMR, CDCl3
f2 (ppm)
f1 (ppm)

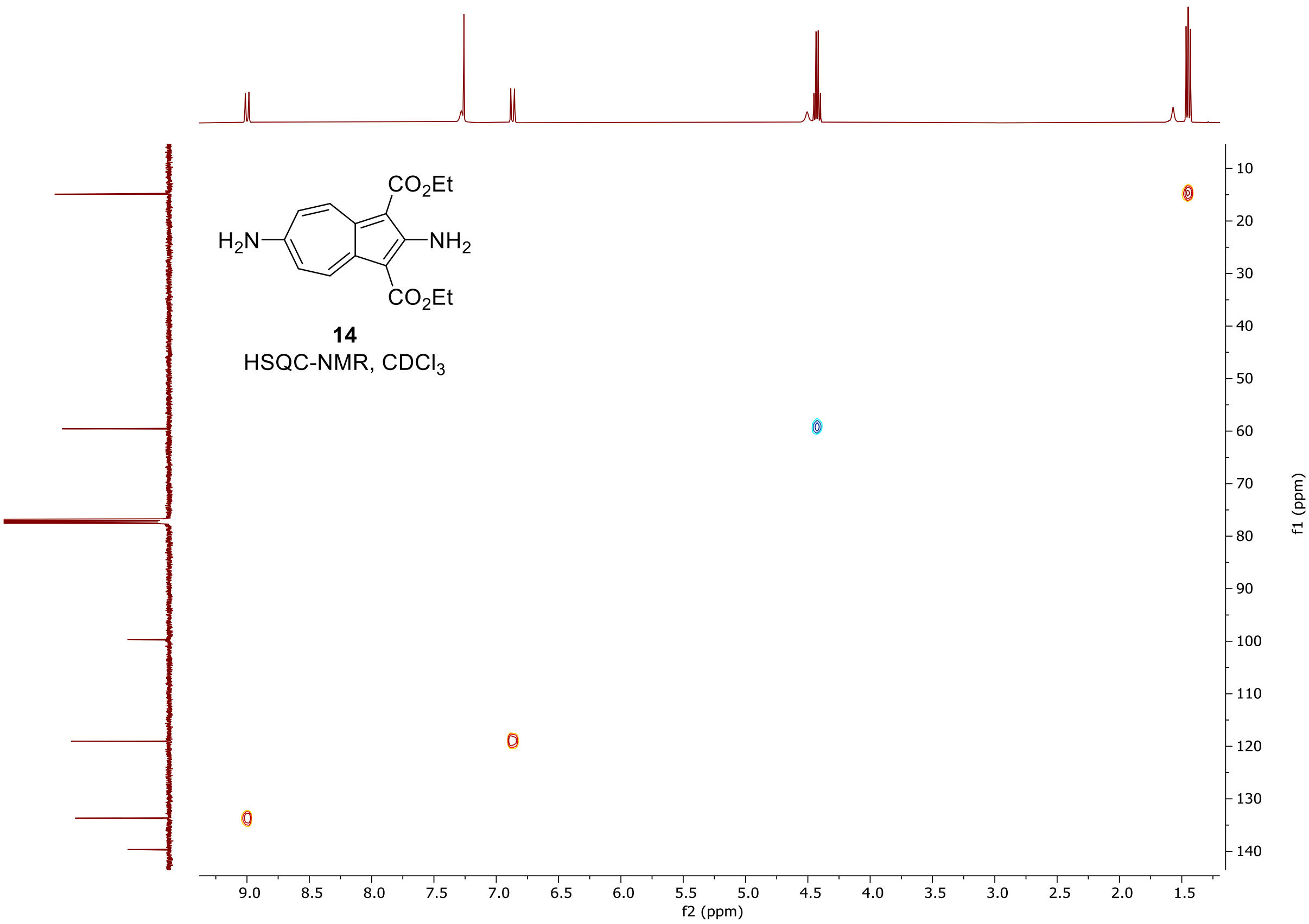
CO2Et
H2N
NH2
CO2Et
14
HSQC-NMR, CDCl3
f2 (ppm)
f1 (ppm)

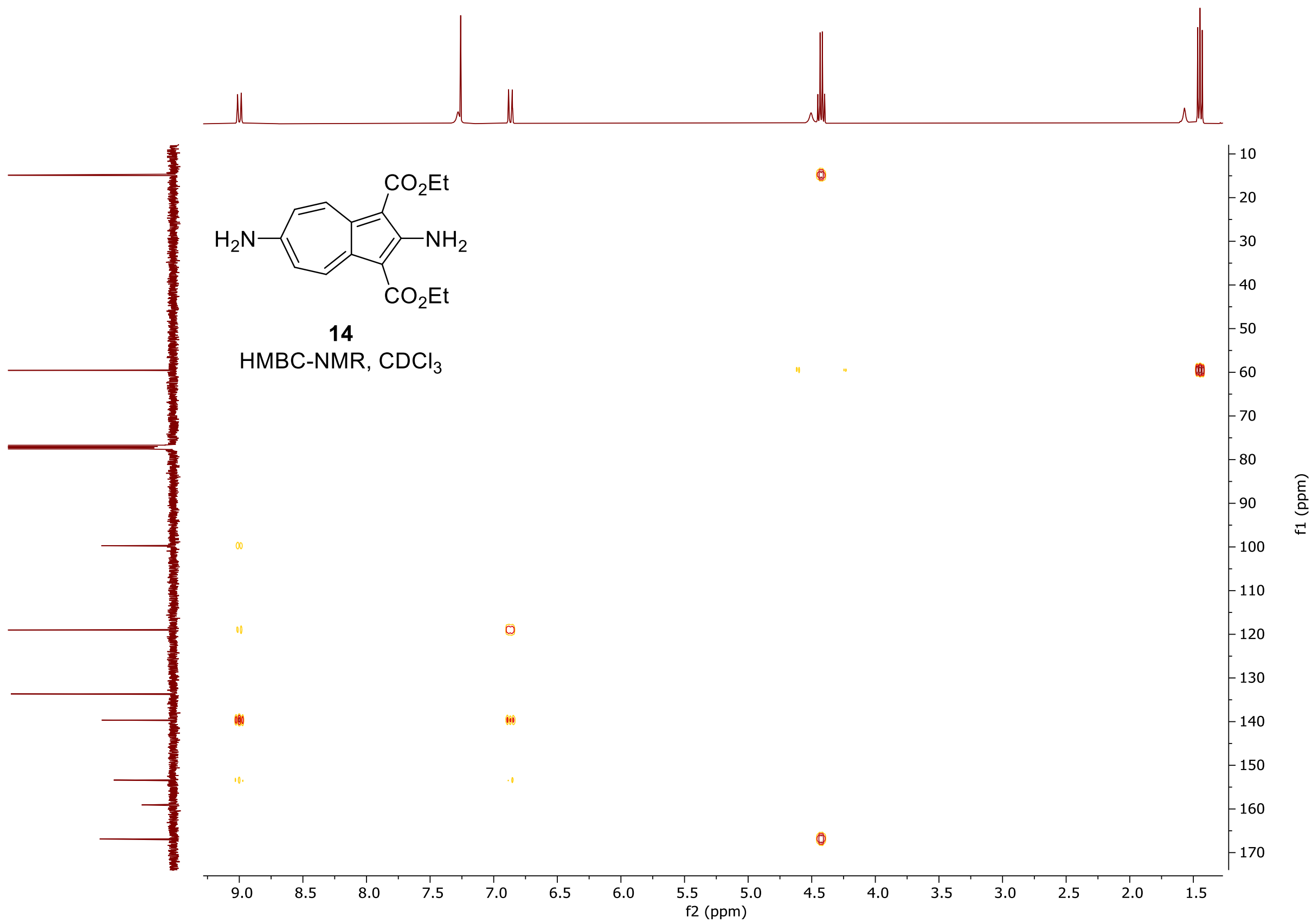

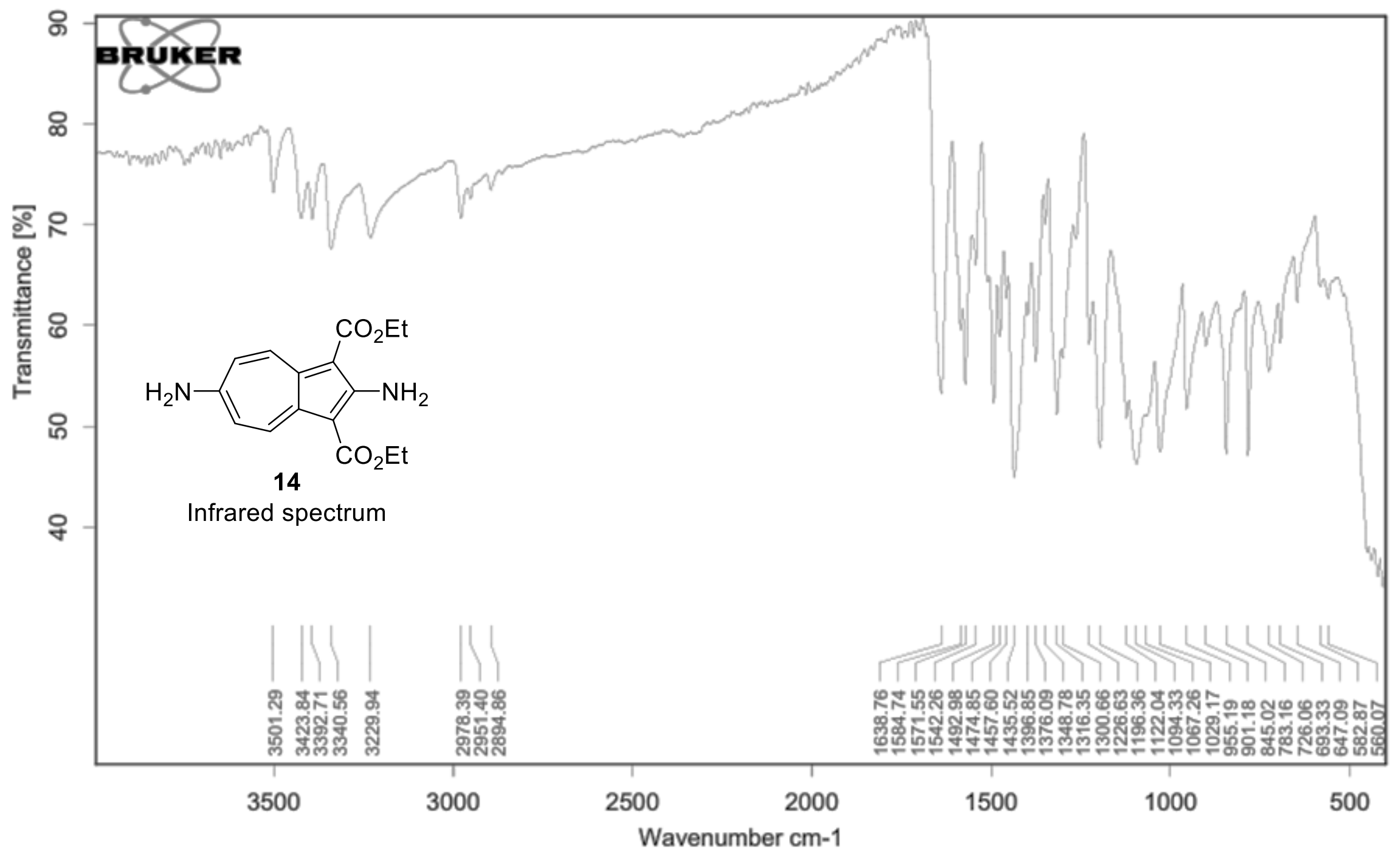

C:\Users\Lab0.25\Documents\IR\220627_1.204 sample sample form	20/09/2023

Page 1 of 1

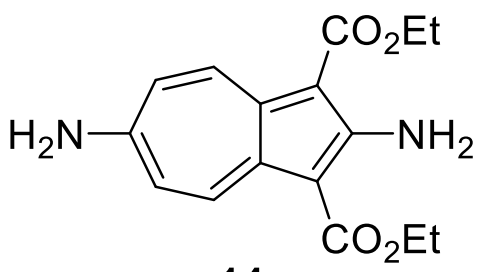

14

HRMS-ESI$^+$

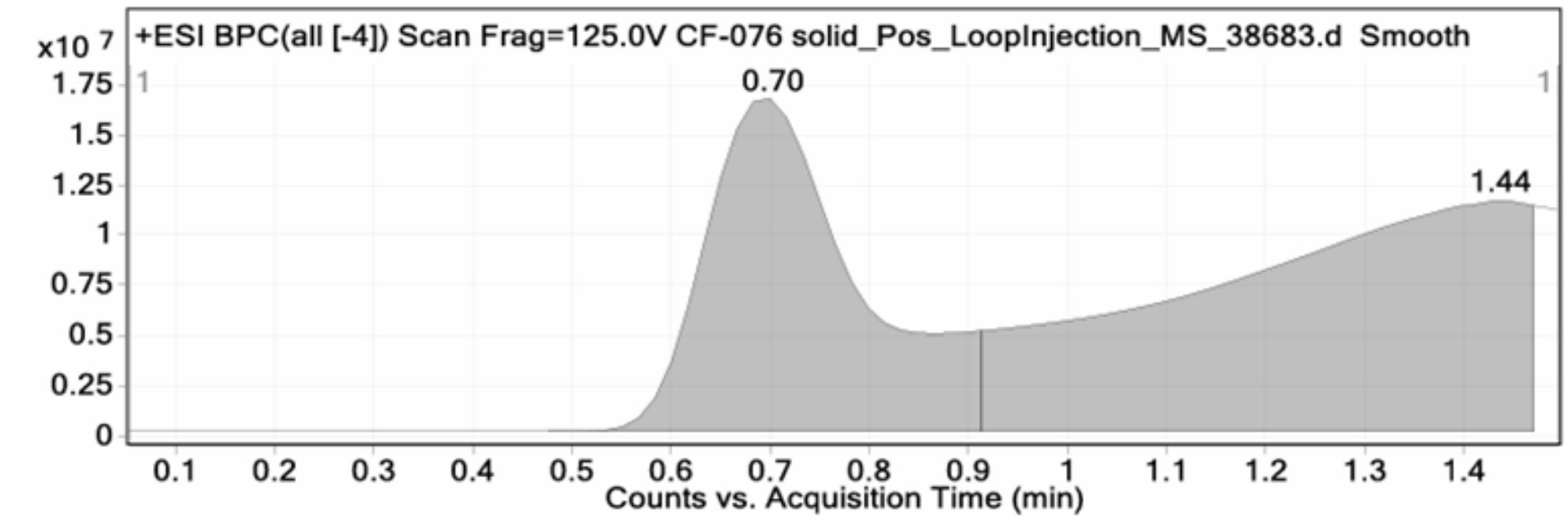

Figure 1: Base peak chromatogram

User Chromatogram Peak List

RT (min)	Area	Area %	Area Sum (%)	Base Peak (m/z)	Width (min)
0.70	176725915	65.17	39.46	303.1302	0.190
1.44	271177269	100.00	60.54	303.1363	0.400

Compound Table

Compound Label	RT (min)	Observed mass (m/z)	Neutral observed mass (Da)	Theoretical mass (Da)	Mass error (ppm)	Isotope match score (%)
Cpd 1: C16 H18 N2 O4	1.43	325.1157	302.1263	302.1267	-1.31	99.56

Mass errors of between -5.00 and 5.00 ppm with isotope match scores above 60% are considered confirmation of molecular formulae

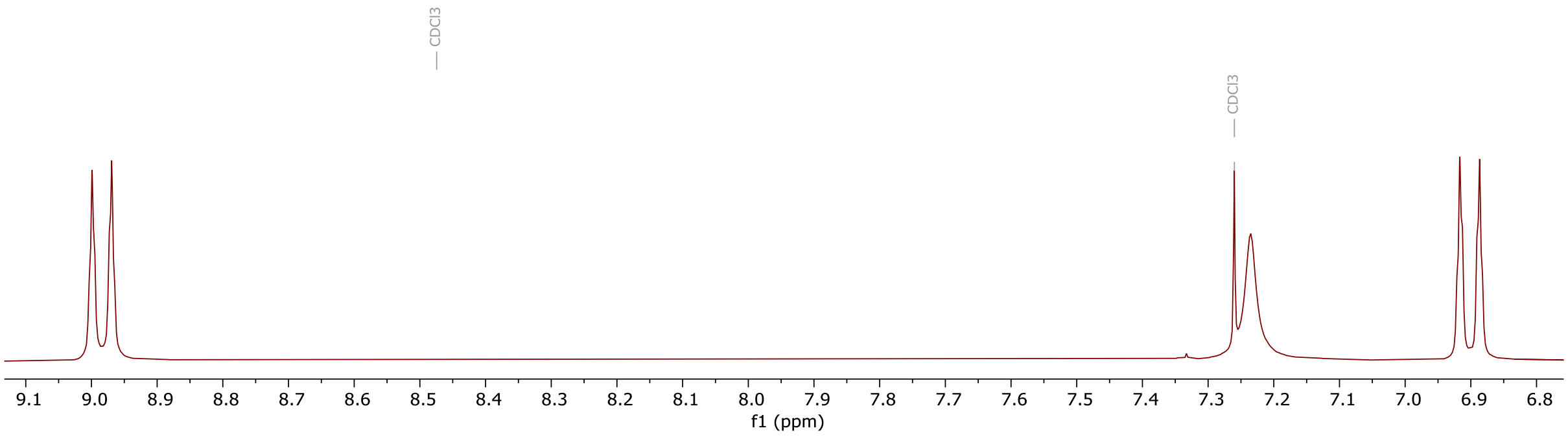

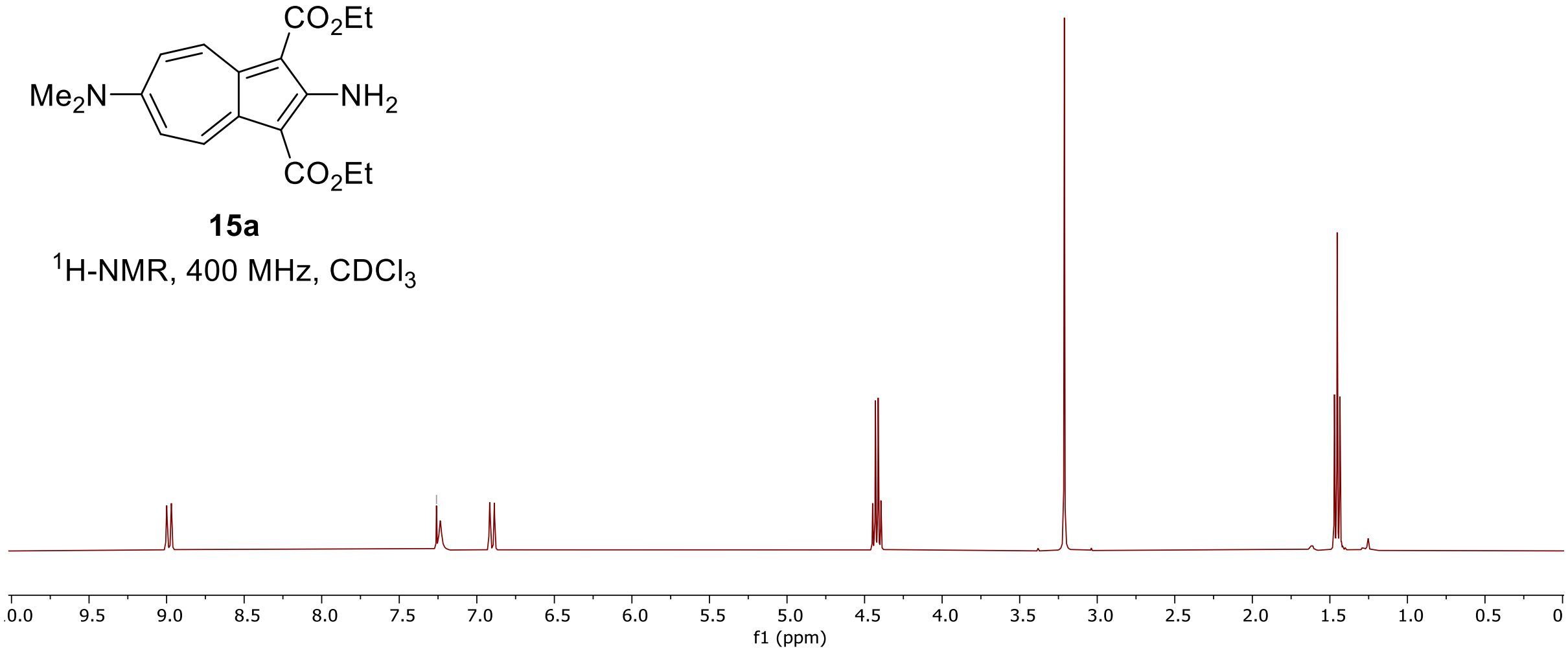

15a

^{1}H-NMR, 400 MHz, $CDCl_3$

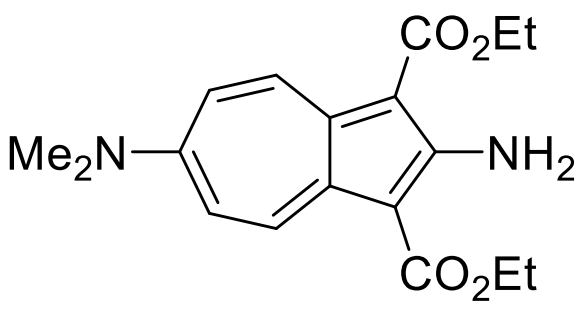

15a

^{13}C-NMR, 101 MHz, $CDCl_3$

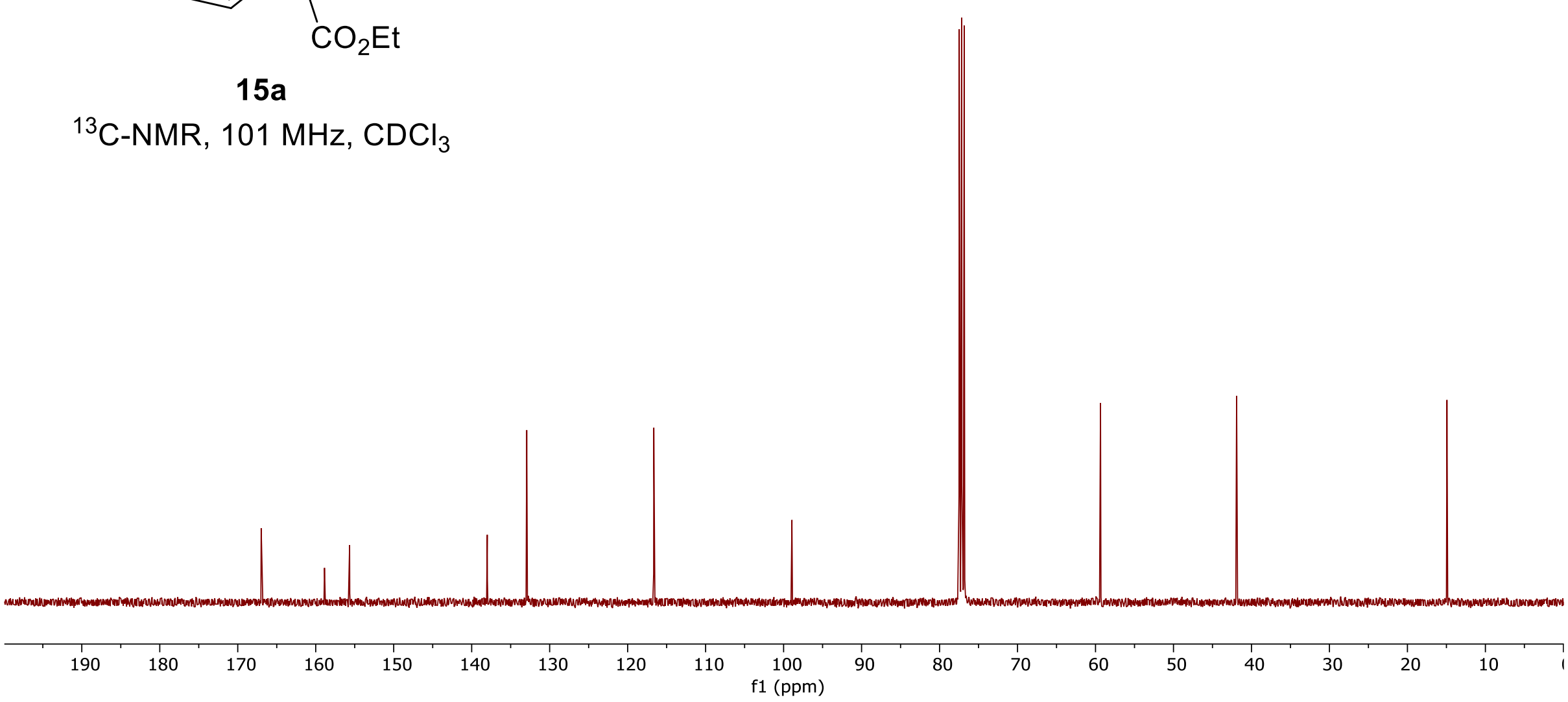

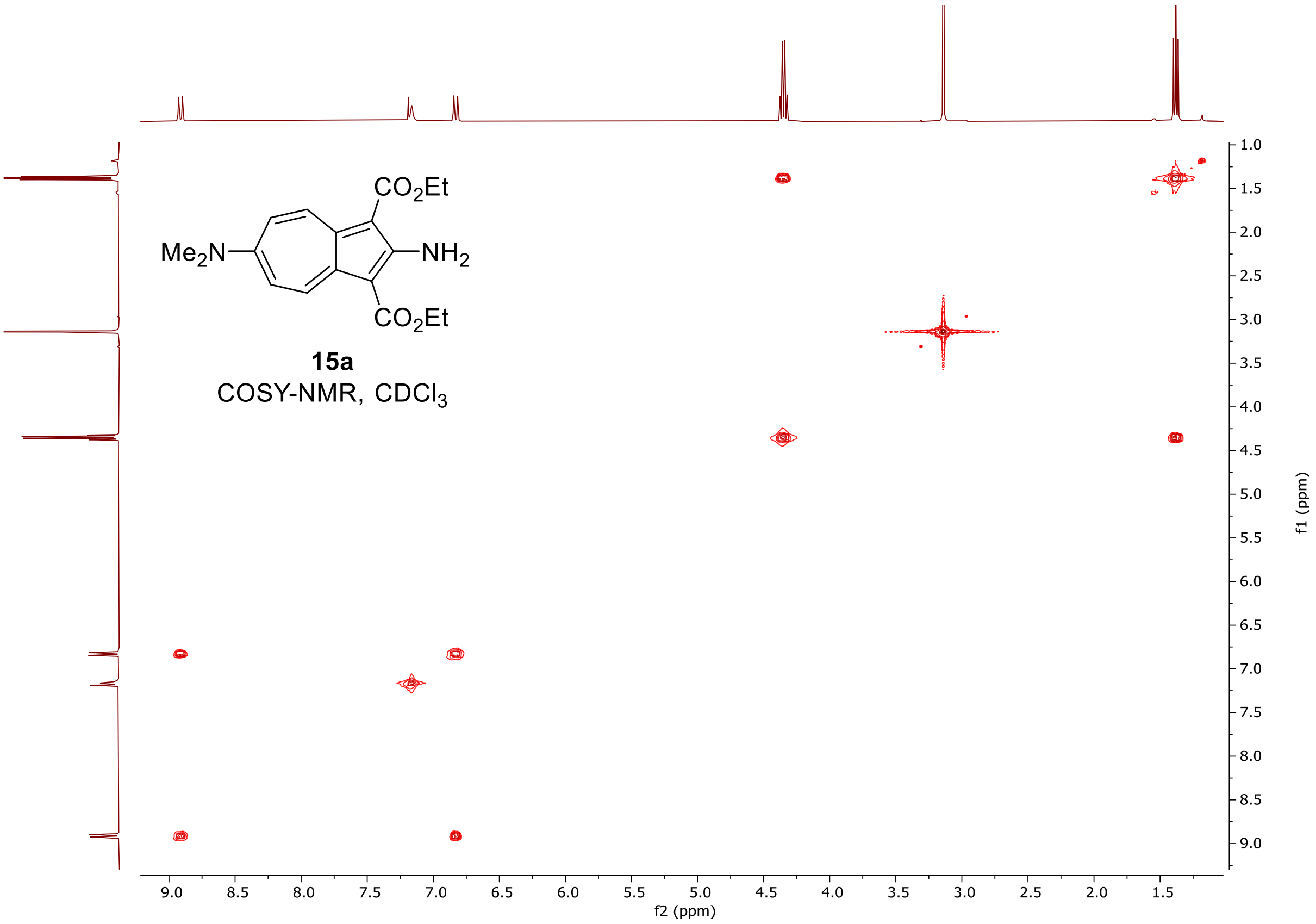
CO2Et
Me2N
NH2
CO2Et
15a
COSY-NMR, CDCl3
f2 (ppm)
f1 (ppm)

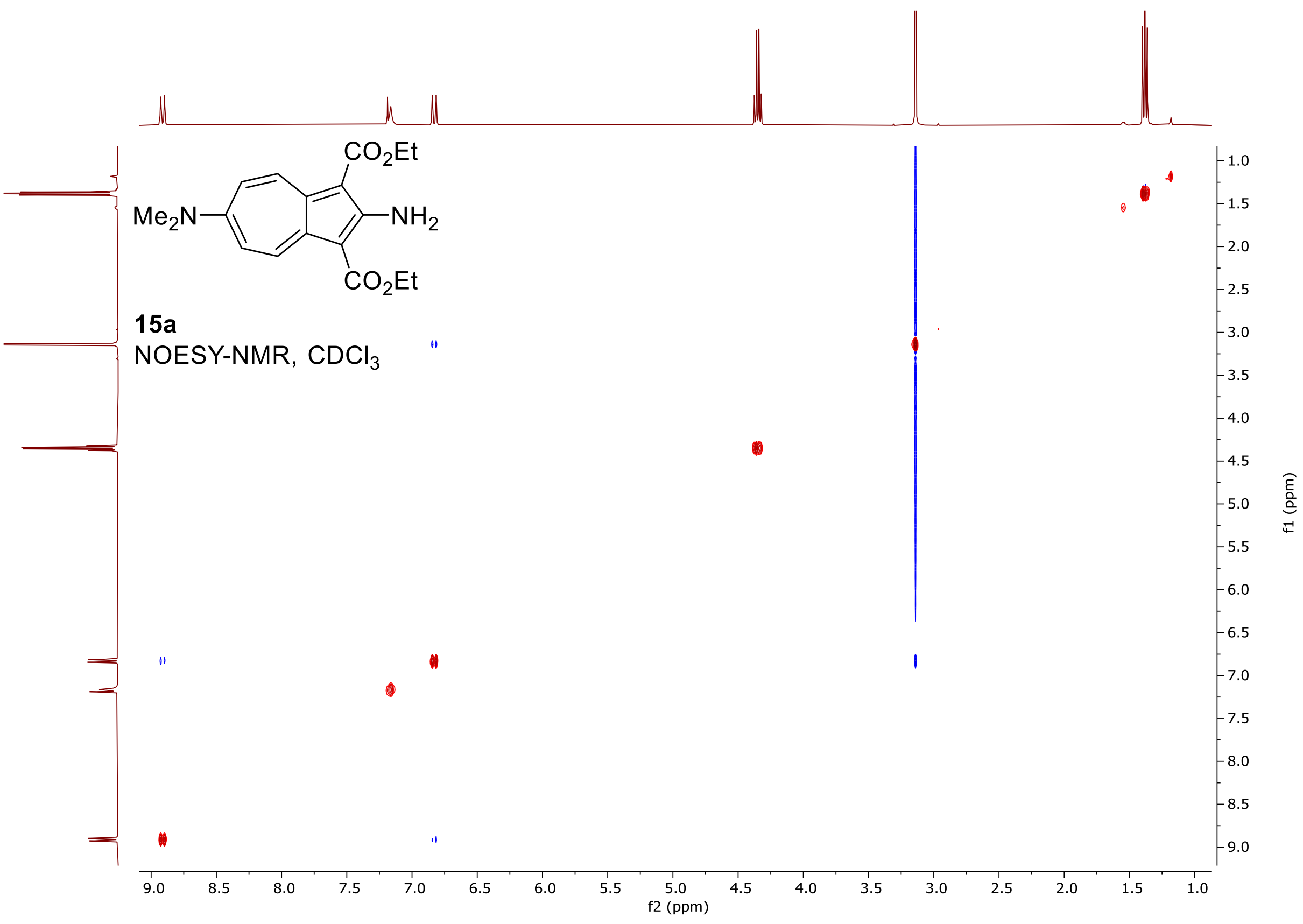
CO2Et
Me2N
NH2
CO2Et
15a
NOESY-NMR, CDCl3
f2 (ppm)
f1 (ppm)

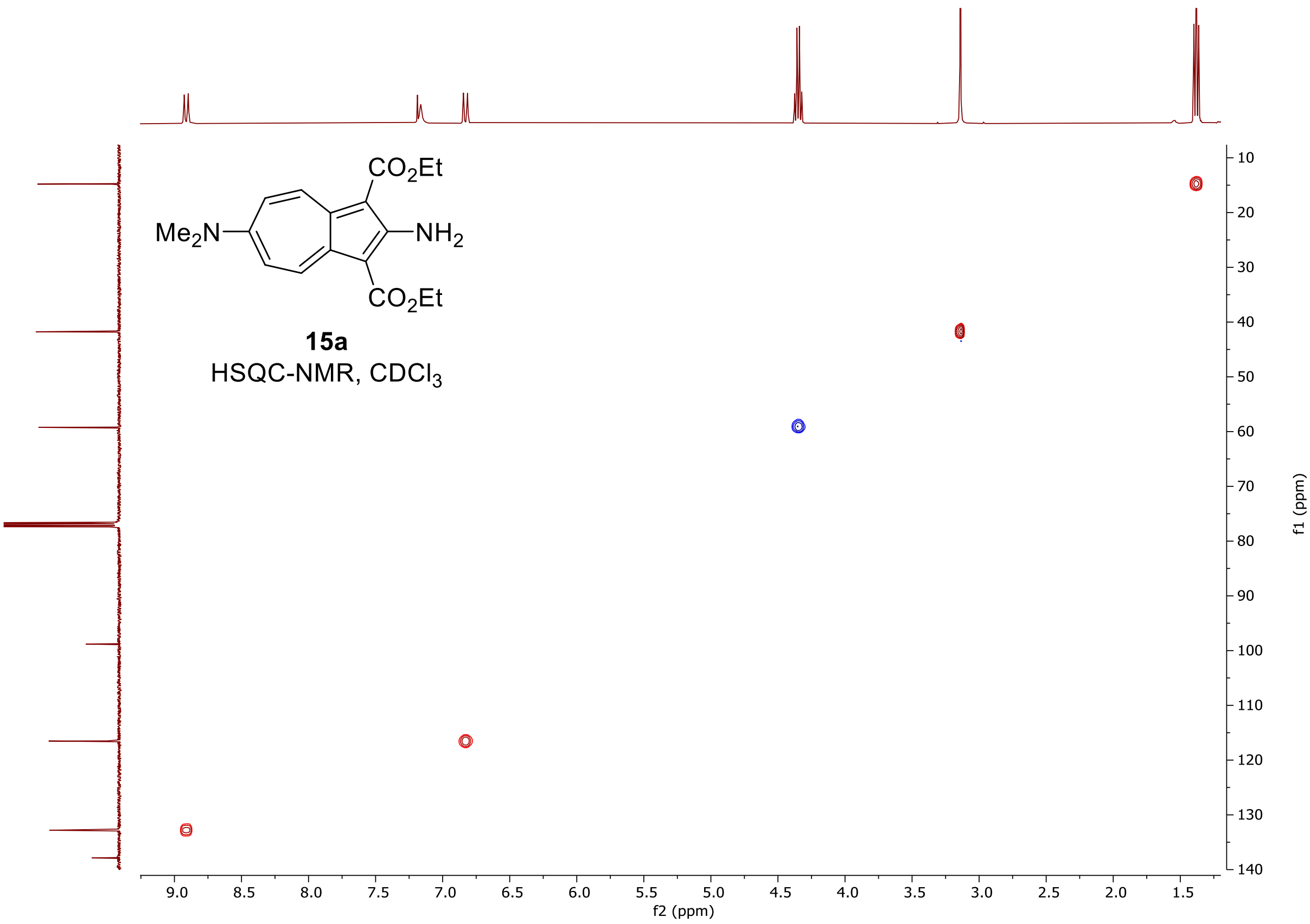
CO2Et
Me2N
NH2
CO2Et
15a
HSQC-NMR, CDCl3
f2 (ppm)
f1 (ppm)

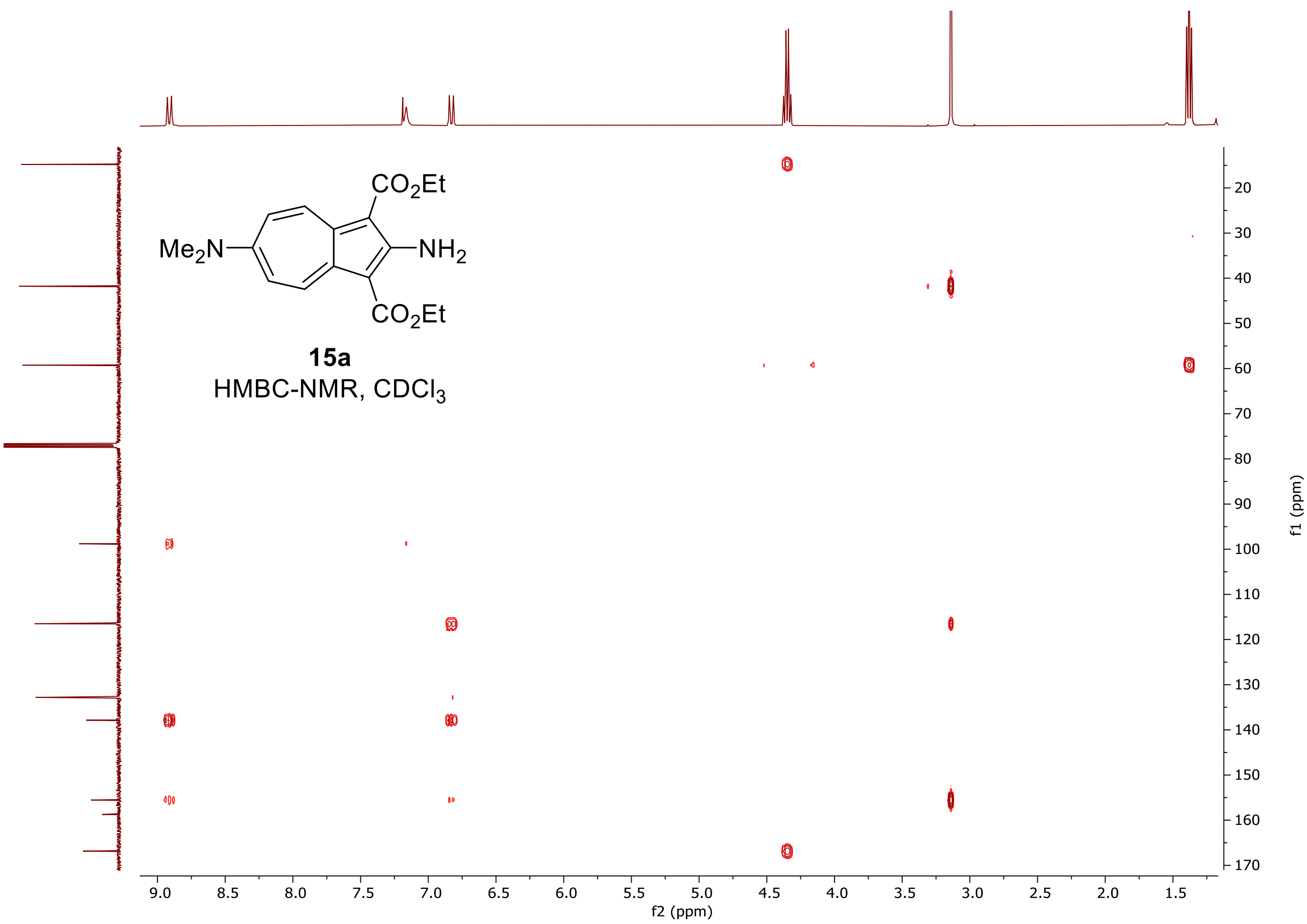
CO2Et
Me2N
NH2
CO2Et
15a
HMBC-NMR, CDCl3
f2 (ppm)
f1 (ppm)

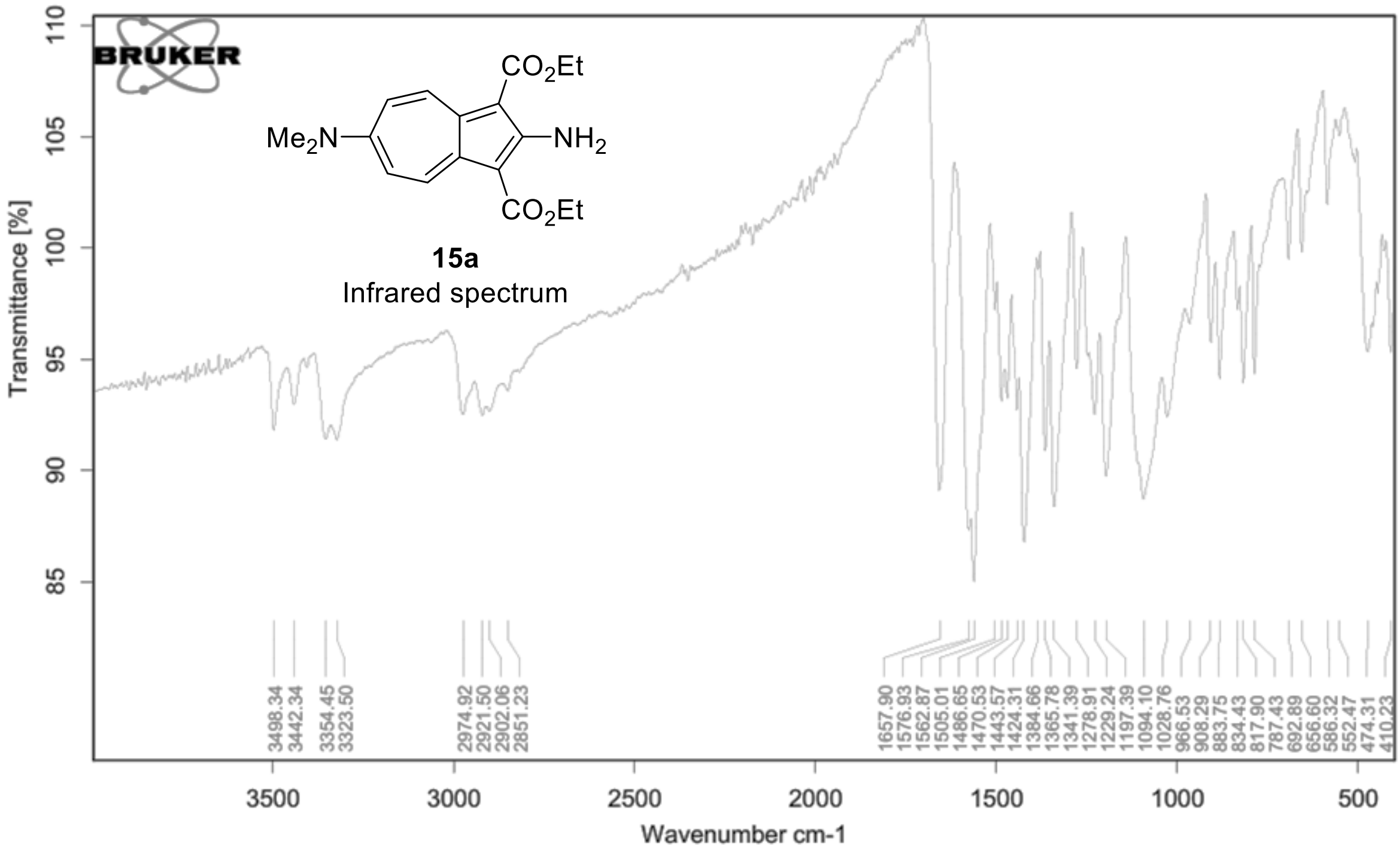

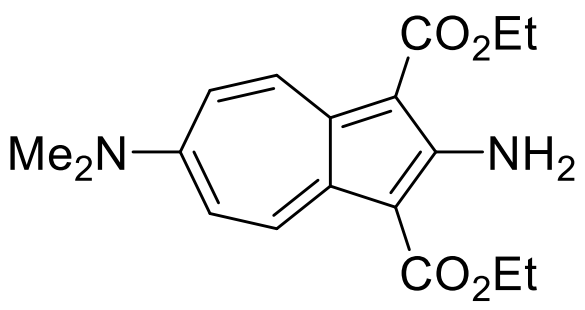

15a
HRMS-ESI$^+$

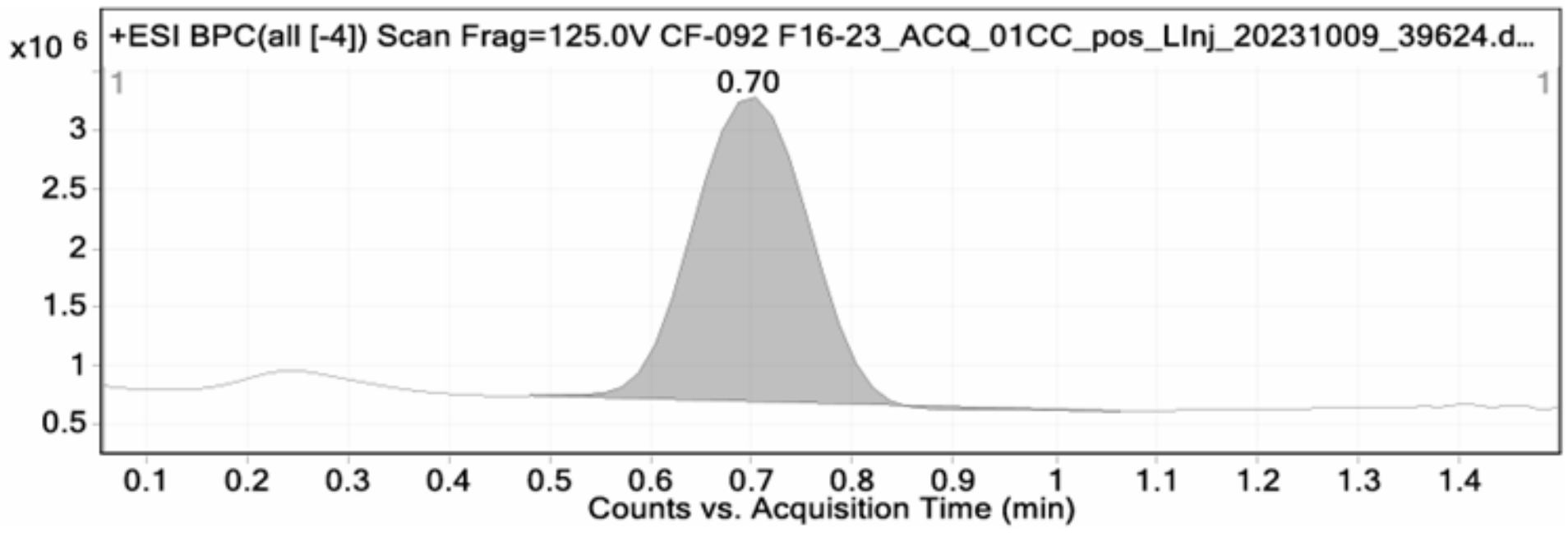

Figure 1: Base peak chromatogram

User Chromatogram Peak List

RT (min)	Area	Area %	Area Sum (%)	Base Peak (m/z)	Width (min)
0.70	20538117	100.00	100.00	330.1608	0.140

Compound Table

Compound Label	RT (min)	Observed mass (m/z)	Neutral observed mass (Da)	Theoretical mass (Da)	Mass error (ppm)	Isotope match score (%)
Cpd 1: C18 H22 N2 O4	0.69	353.1472	330.1574	330.1580	-1.75	99.53

Mass errors of between -5.00 and 5.00 ppm with isotope match scores above 60% are considered confirmation of molecular formulae

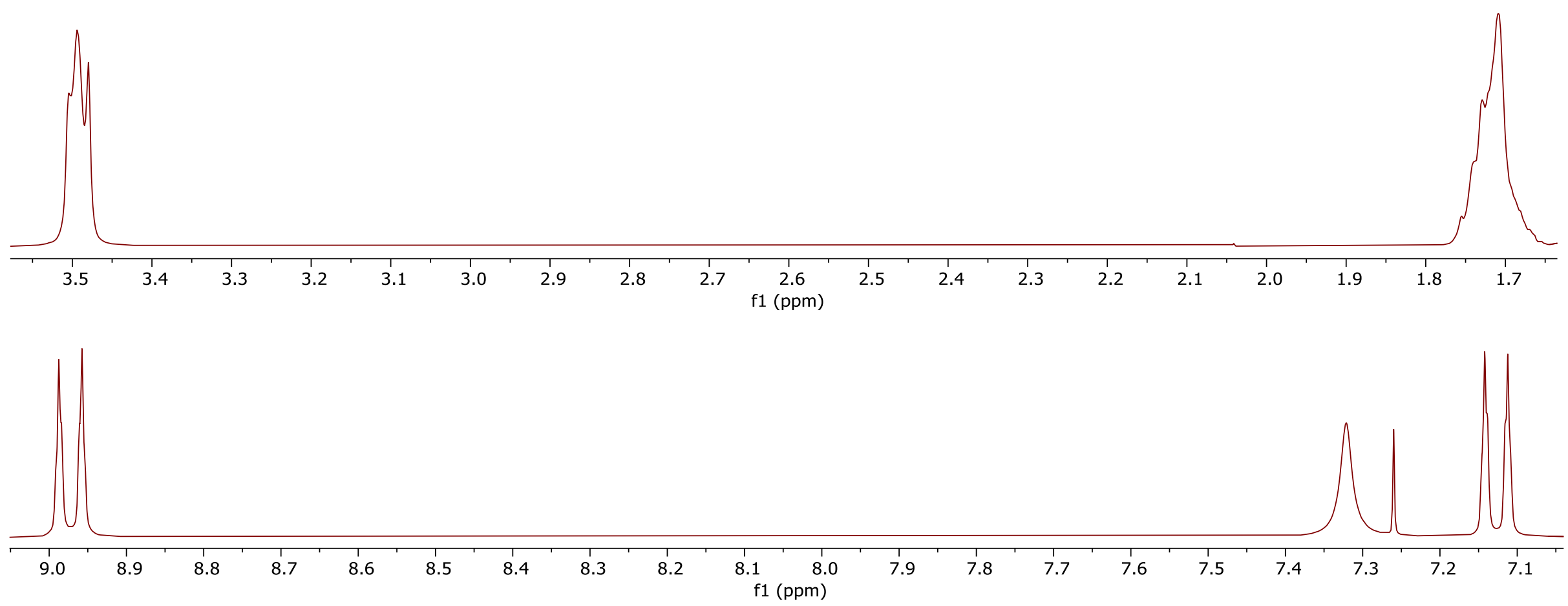

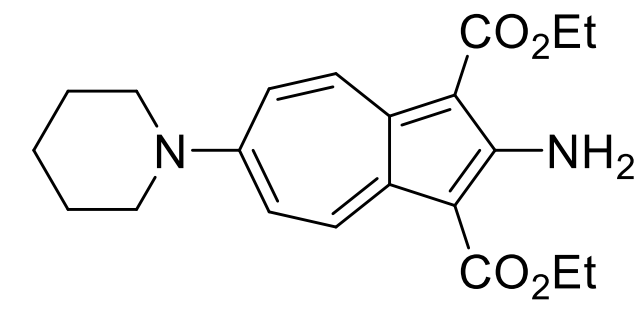

15b

^{1}H-NMR, 400 MHz, $CDCl_3$

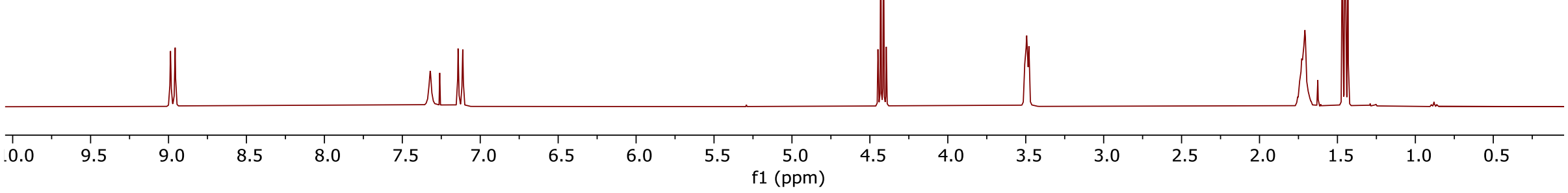

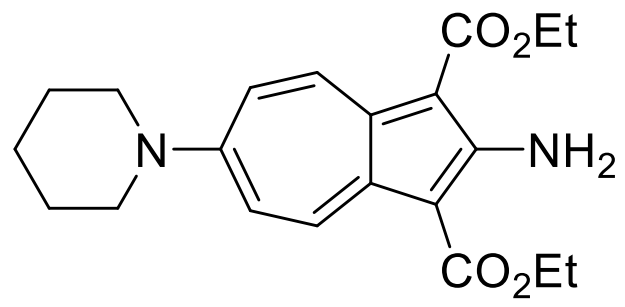

15b

^{13}C-NMR, 101 MHz, $CDCl_3$

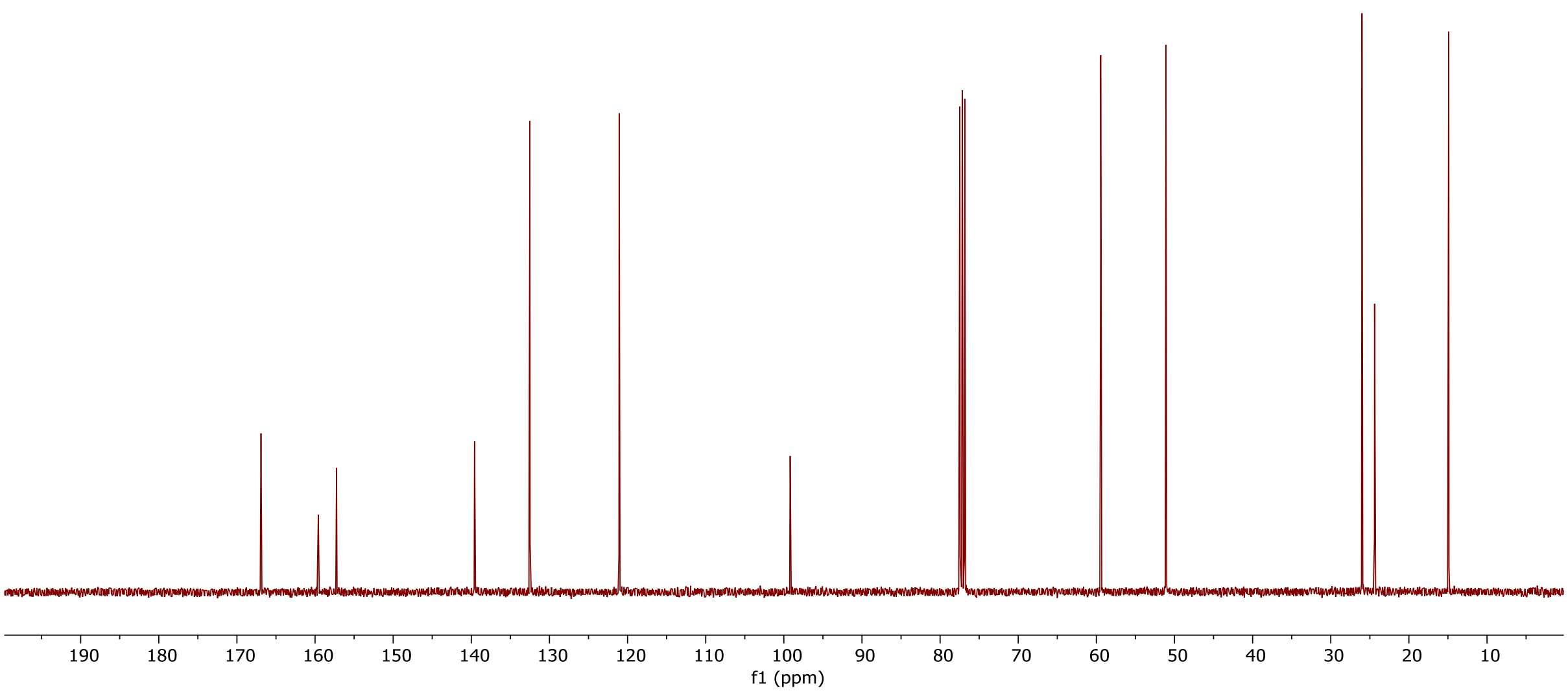

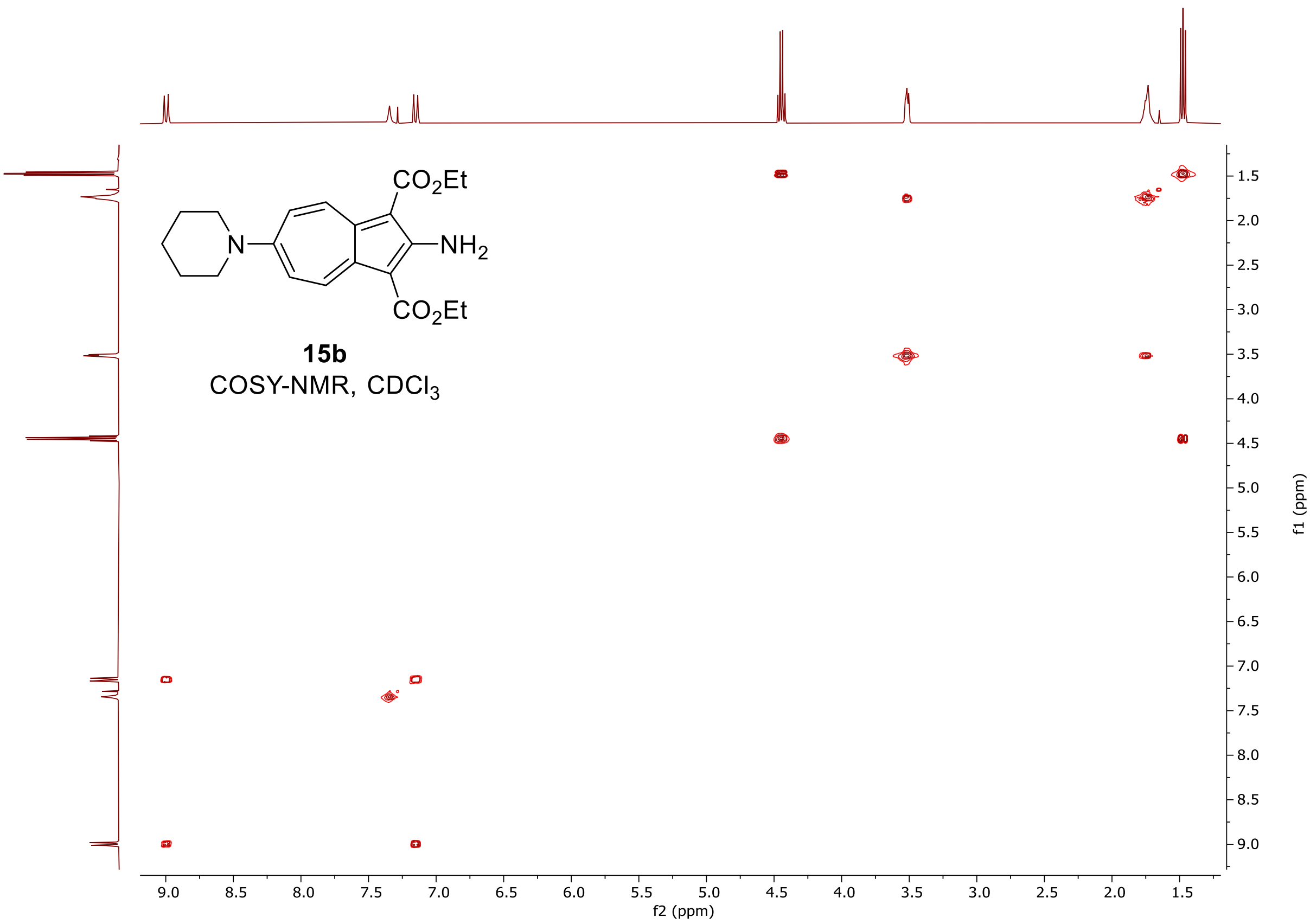

15b
COSY-NMR, $CDCl_3$

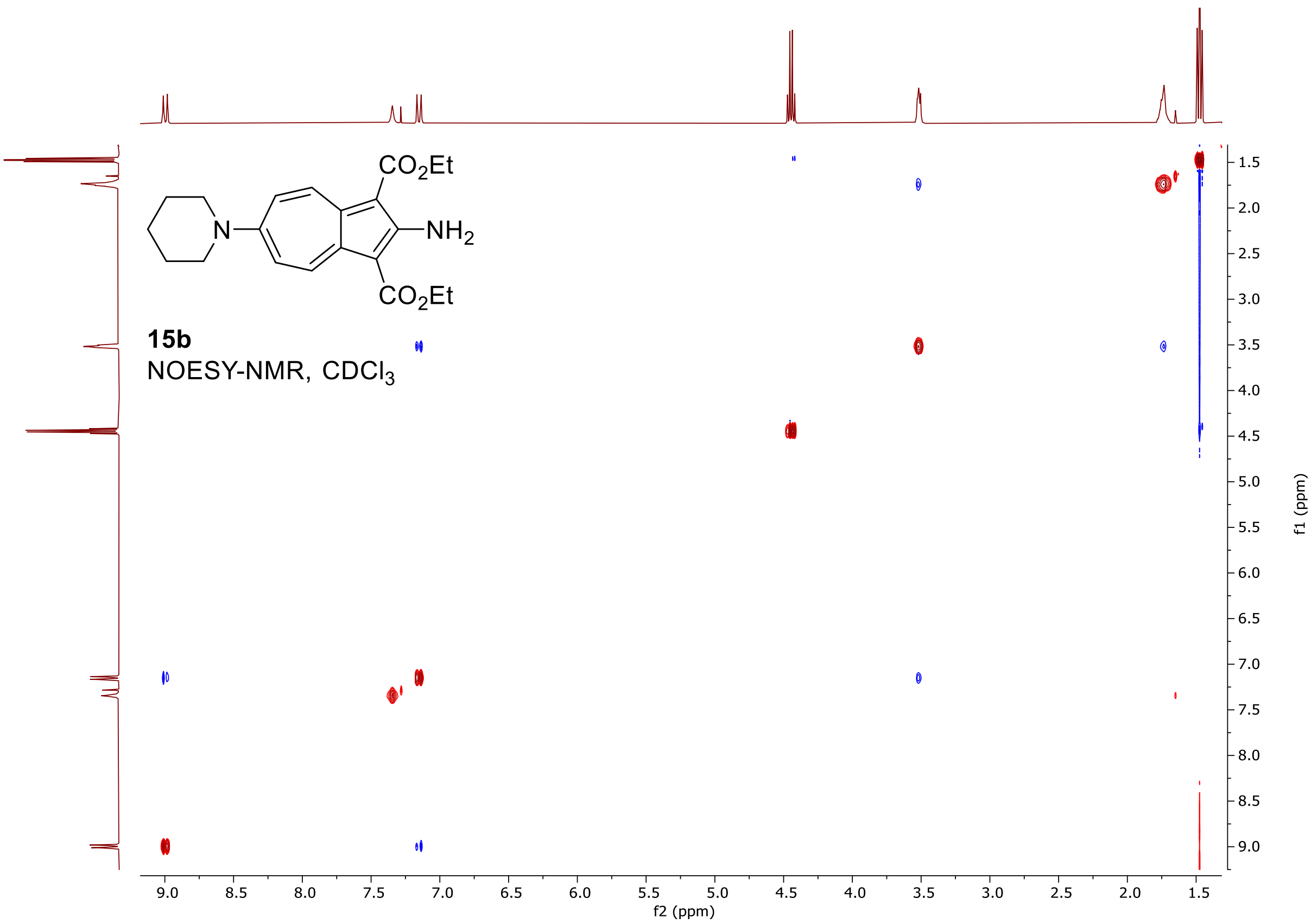

15b
NOESY-NMR, $CDCl_3$

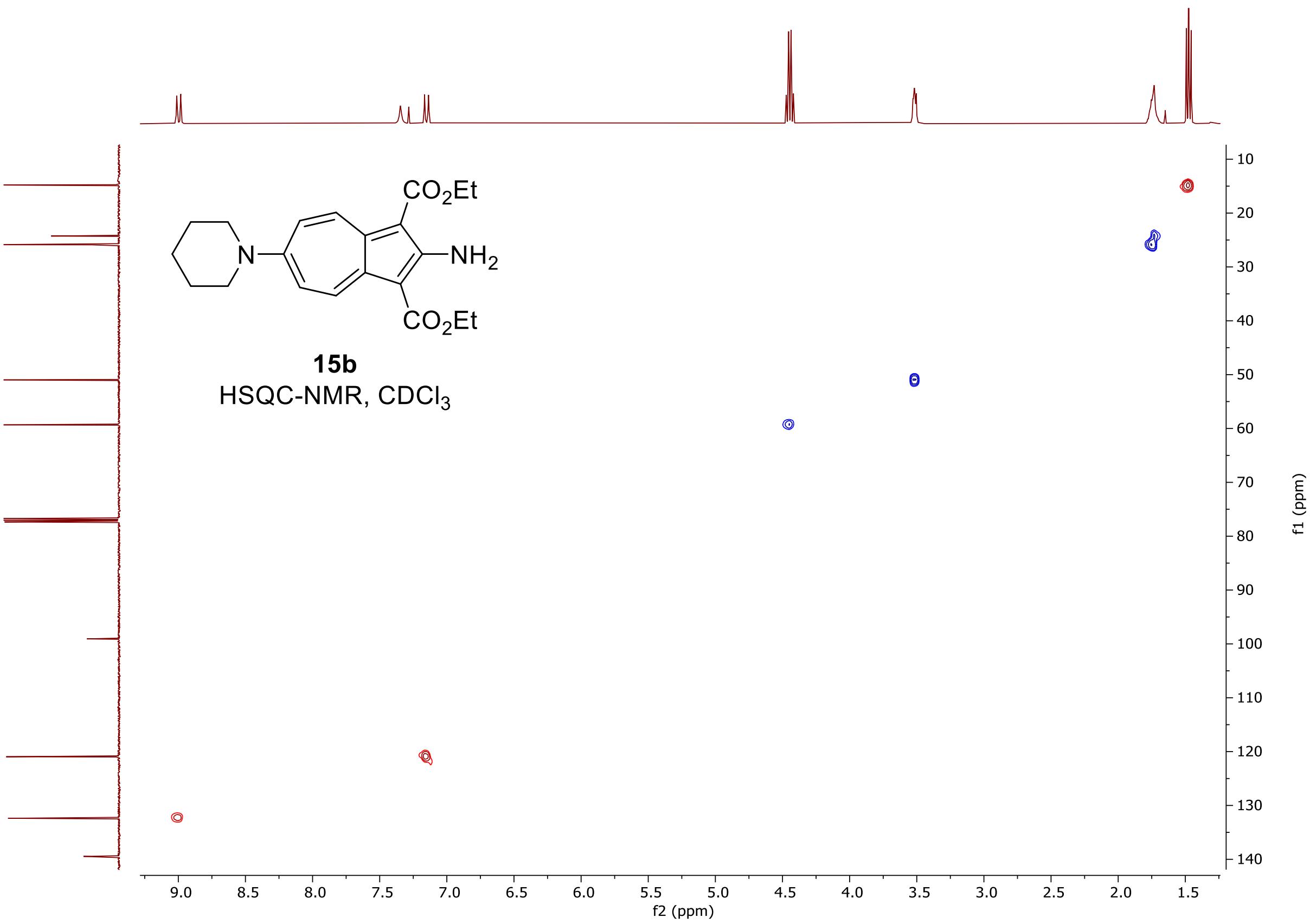
CO2Et
NH2
CO2Et
N
15b
HSQC-NMR, CDCl3
f2 (ppm)
f1 (ppm)

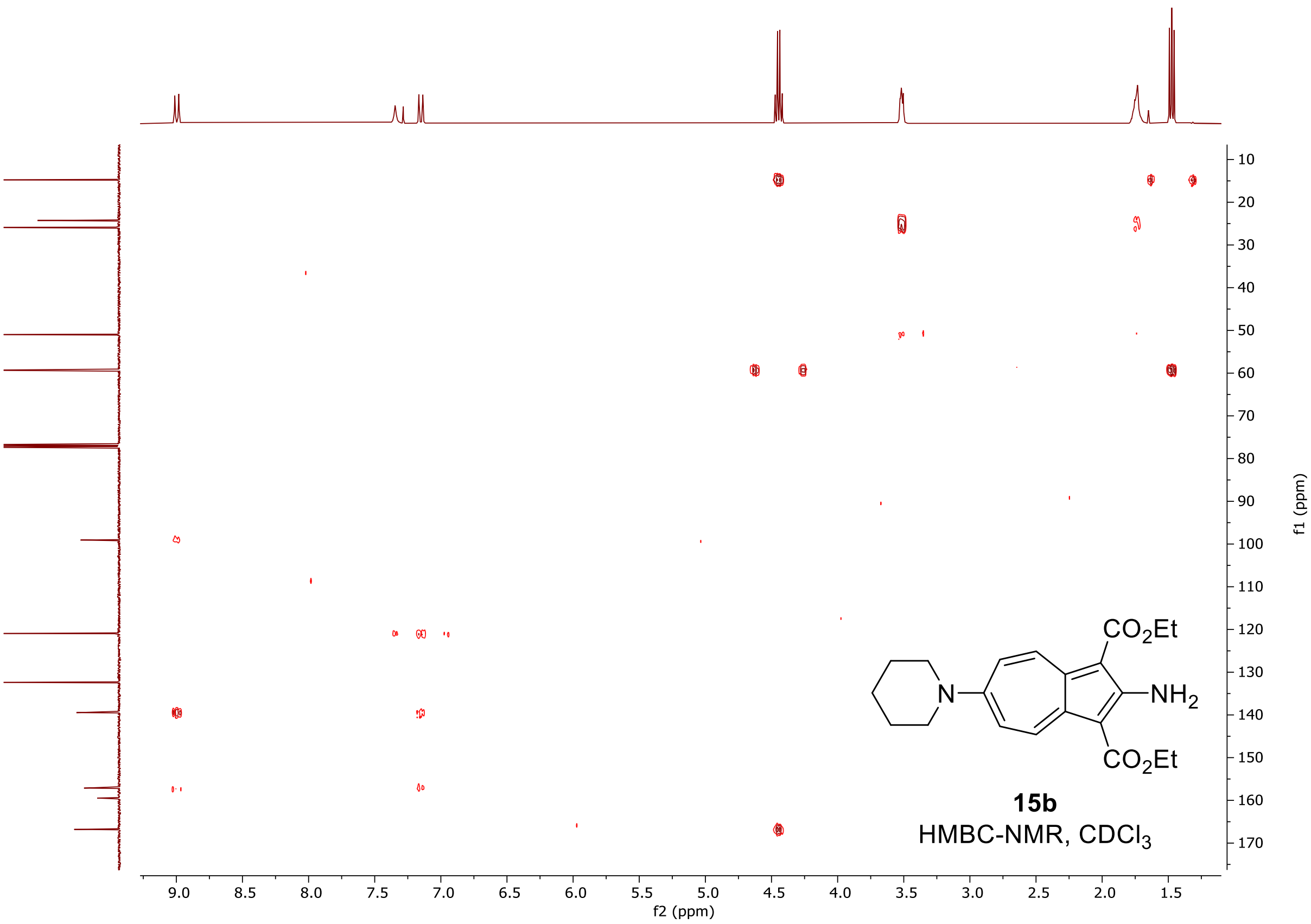
CO2Et
NH2
CO2Et
N
15b
HMBC-NMR, CDCl3
f2 (ppm)
f1 (ppm)

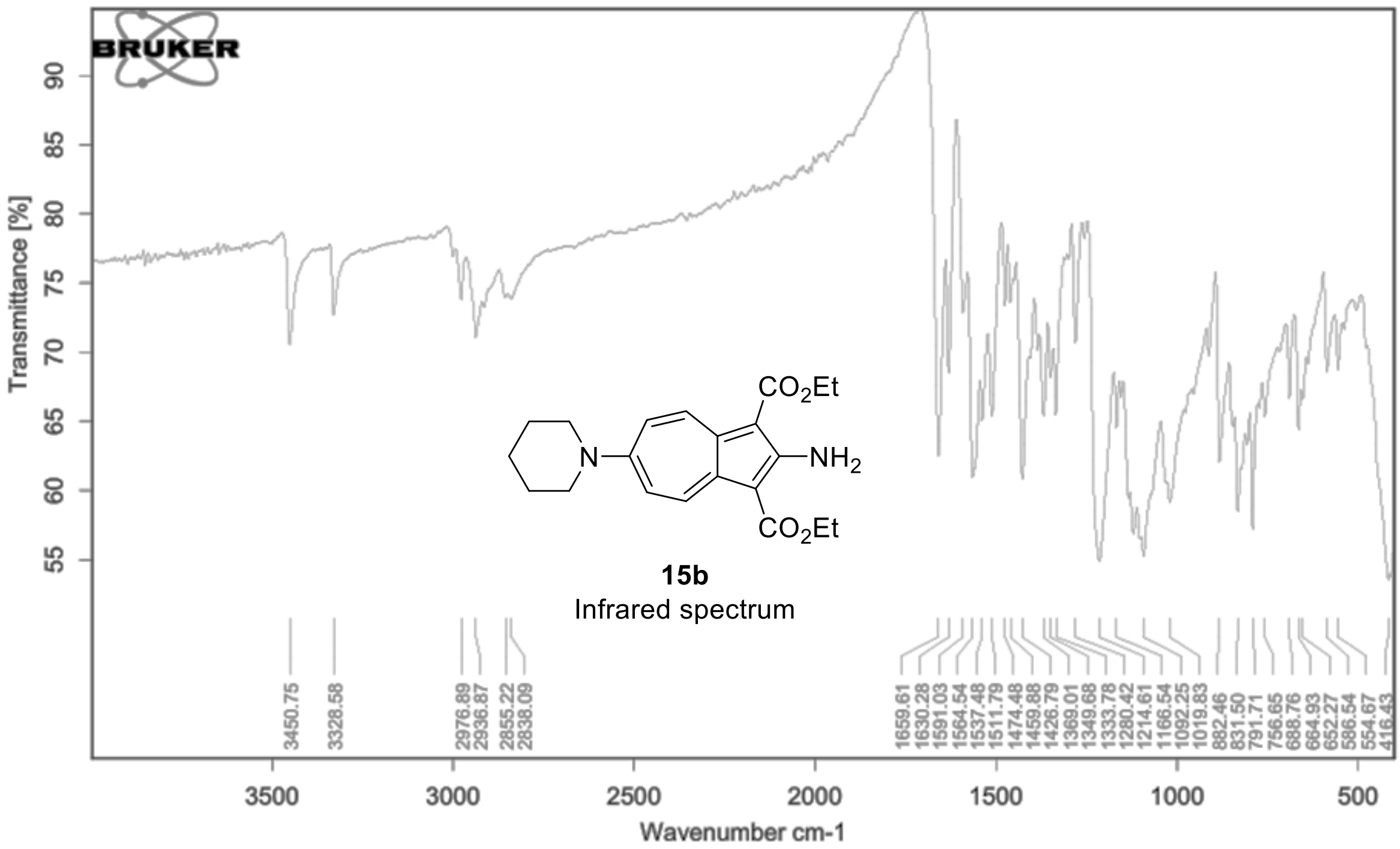

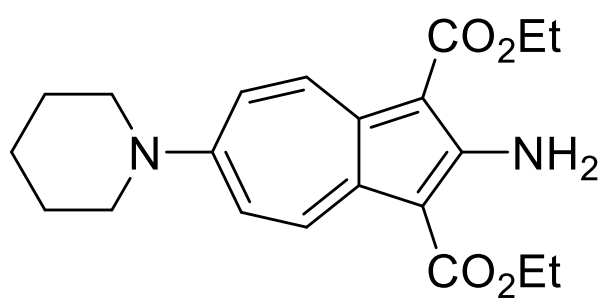

15b
HRMS-ESI$^+$

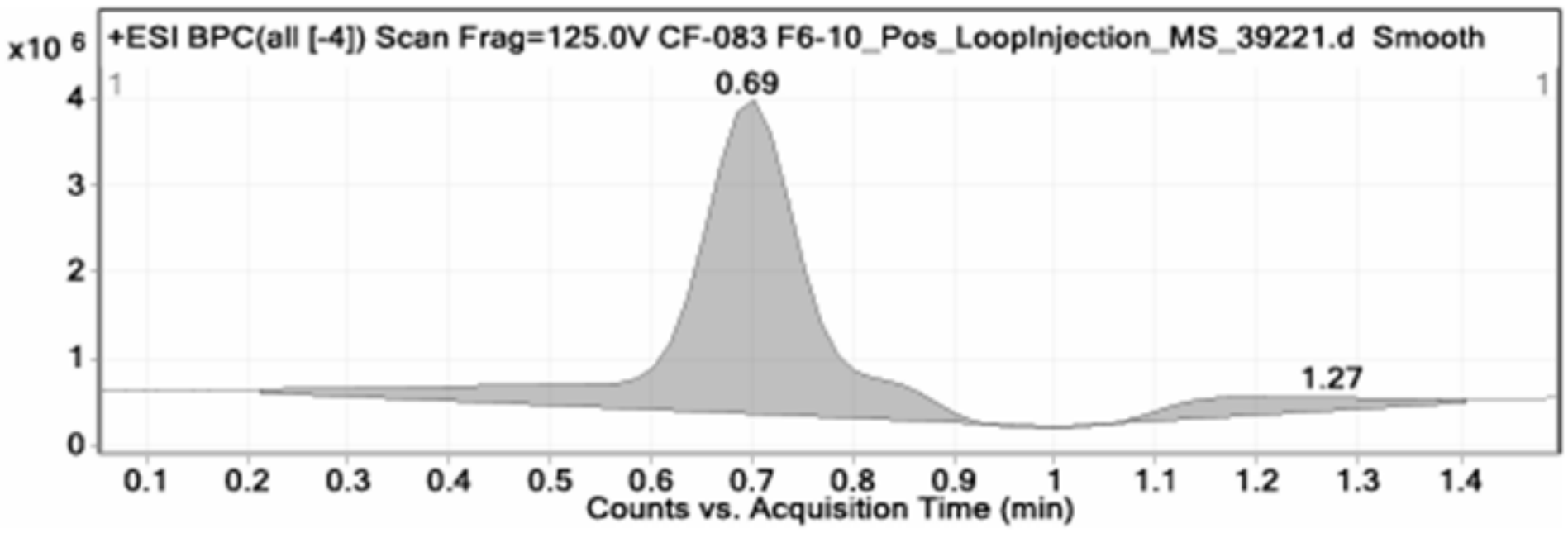

Figure 1: Base peak chromatogram

User Chromatogram Peak List

RT (min)	Area	Area %	Area Sum (%)	Base Peak (m/z)	Width (min)
0.69	29980355	100.00	91.49	371.1952	0.160
1.27	2788803	9.30	8.51	922.0081	0.310

Compound Table

Compound Label	RT (min)	Observed mass (m/z)	Neutral observed mass (Da)	Theoretical mass (Da)	Mass error (ppm)	Isotope match score (%)
Cpd 1: C21 H26 N2 O4	0.69	393.1787	370.1892	370.1893	-0.24	99.54

Mass errors of between -5.00 and 5.00 ppm with isotope match scores above 60% are considered confirmation of molecular formulae

15c

^{1}H-NMR, 400 MHz, $CDCl_3$

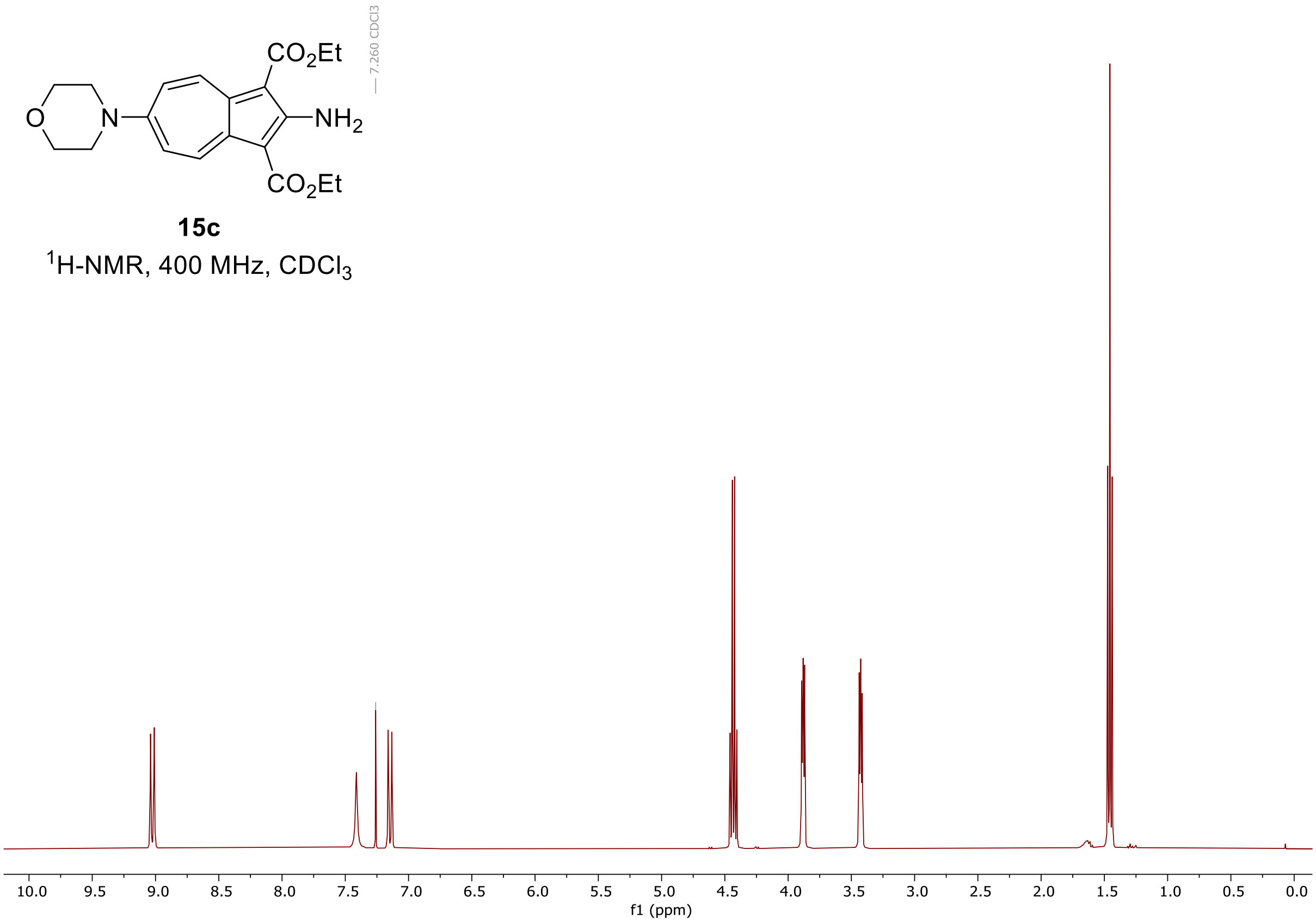

15c

^{13}C-NMR, 101 MHz, $CDCl_3$

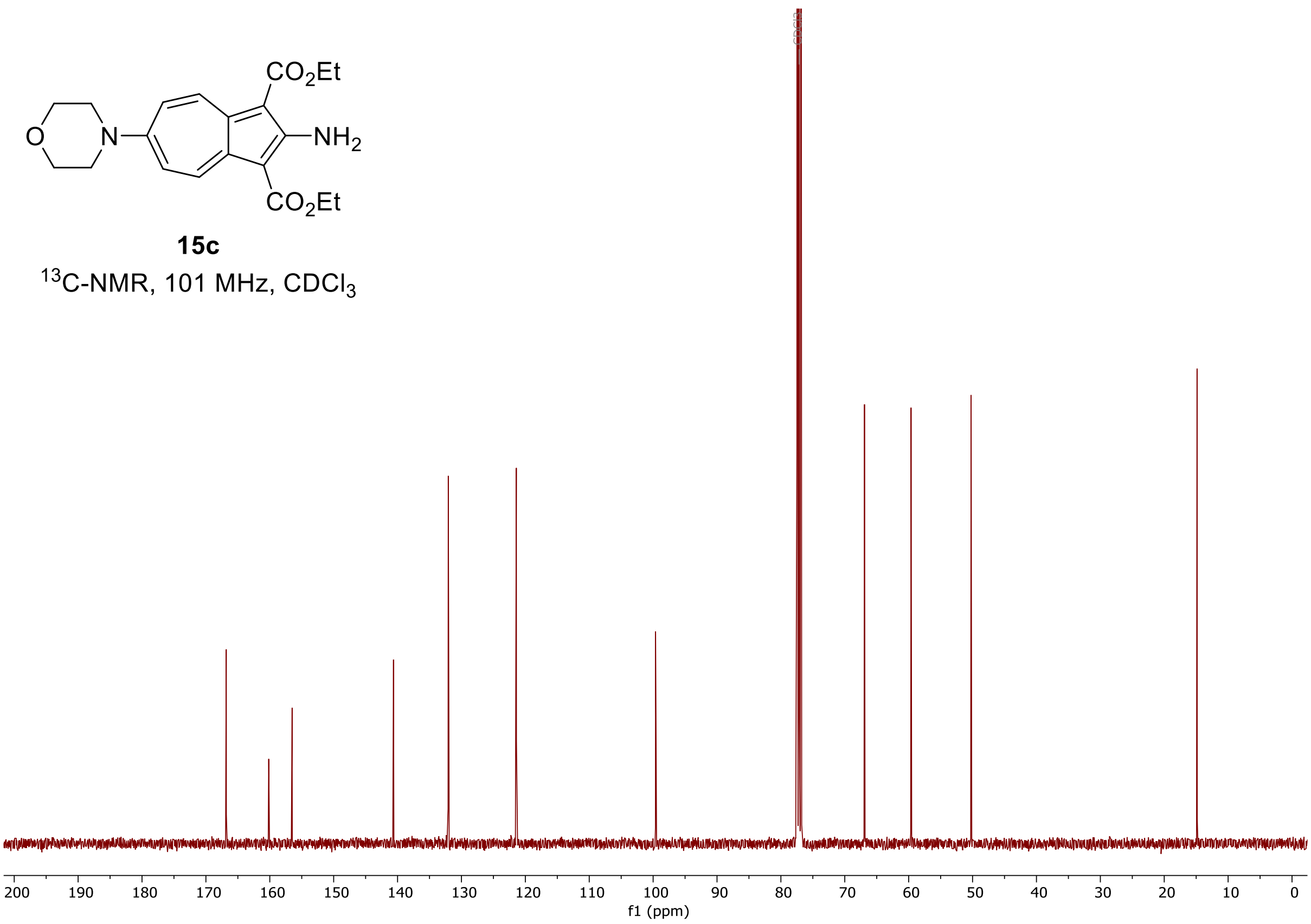

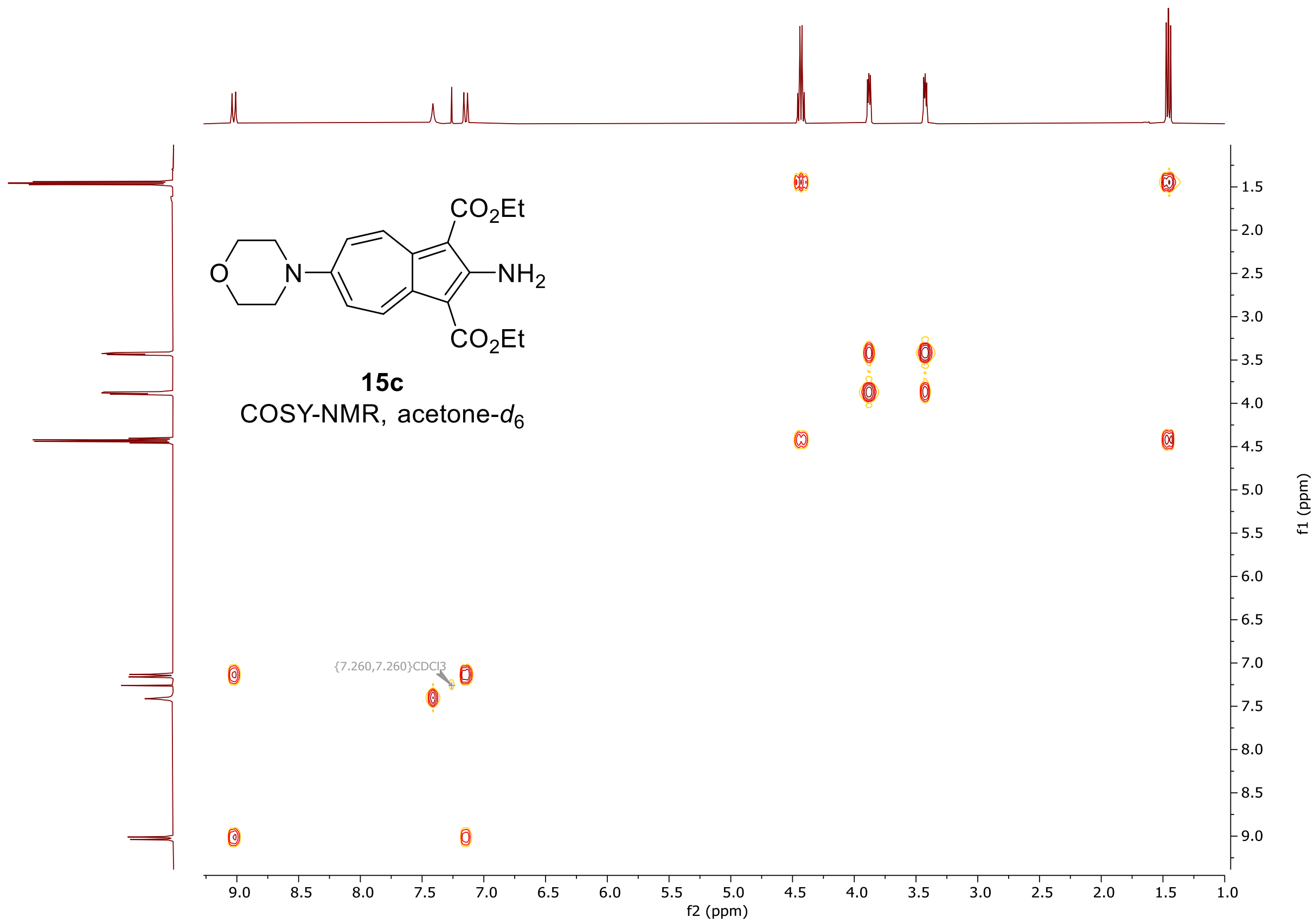

CO2Et
NH2
CO2Et
O
N
15c
COSY-NMR, acetone-d6
{7.260,7.260}CDCl3
f2 (ppm)
f1 (ppm)

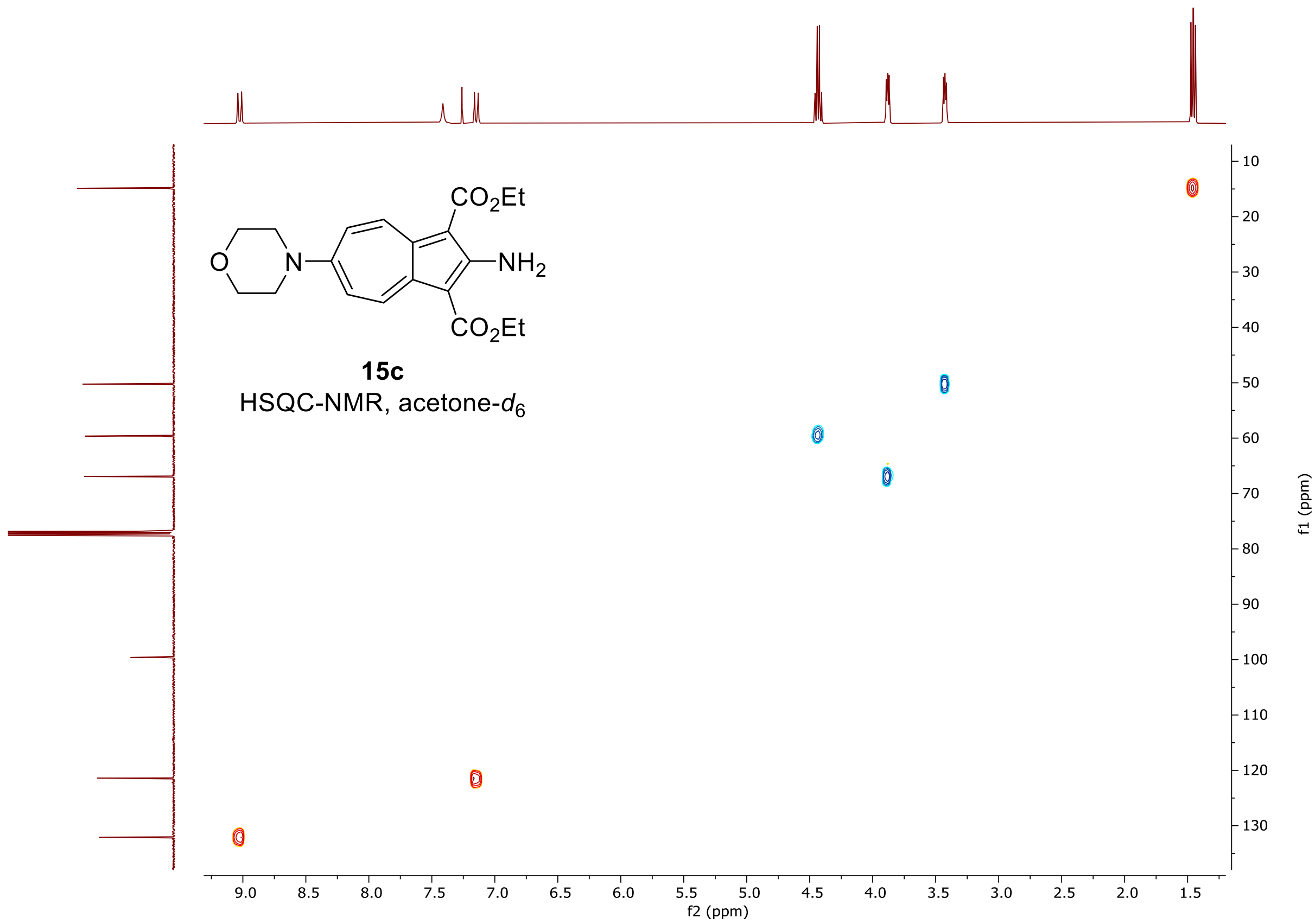

CO2Et
NH2
CO2Et
O
N
15c
HSQC-NMR, acetone-d6
f2 (ppm)
f1 (ppm)

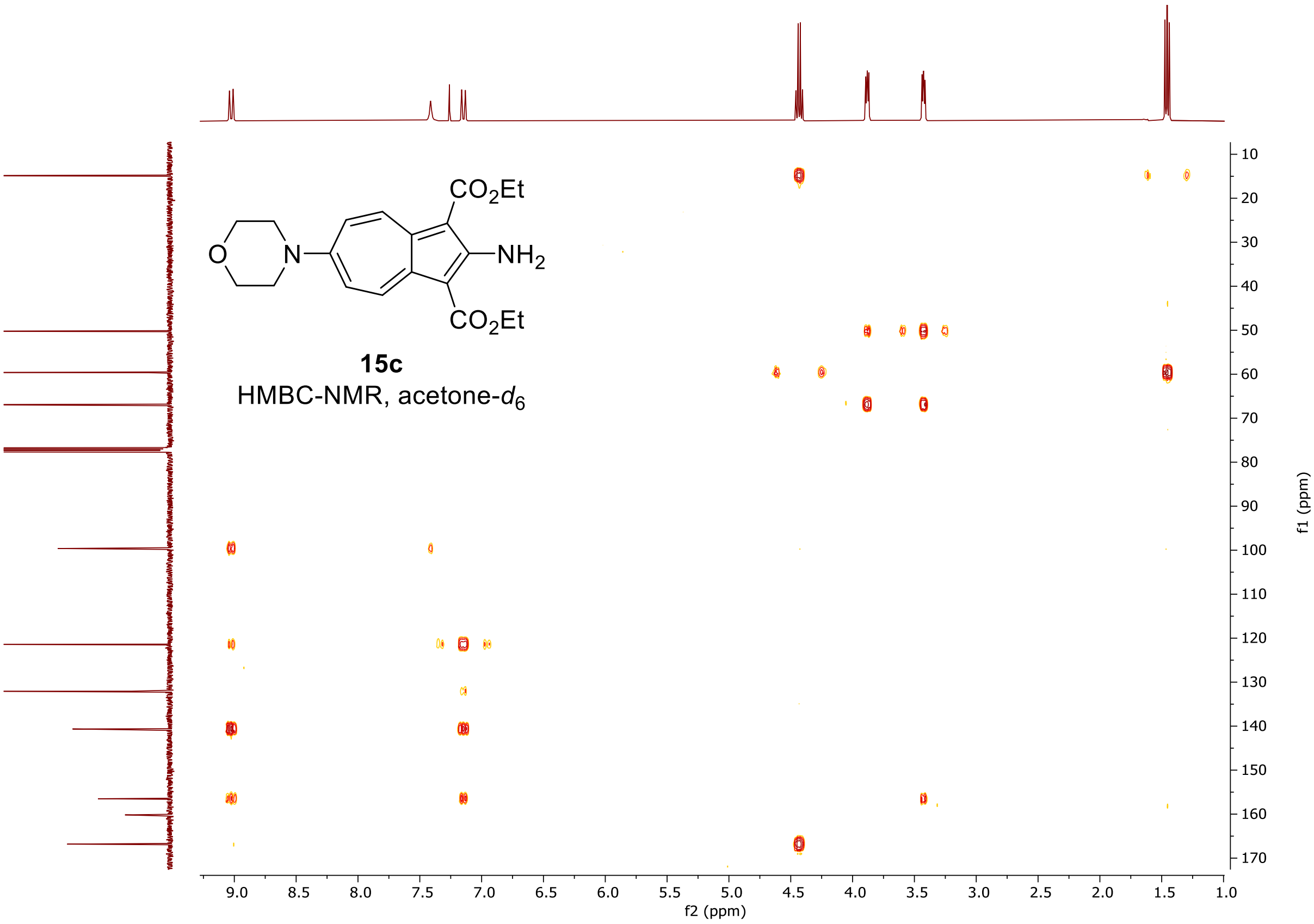
CO2Et
NH2
CO2Et
O
N
15c
HMBC-NMR, acetone-d6
f2 (ppm)
f1 (ppm)

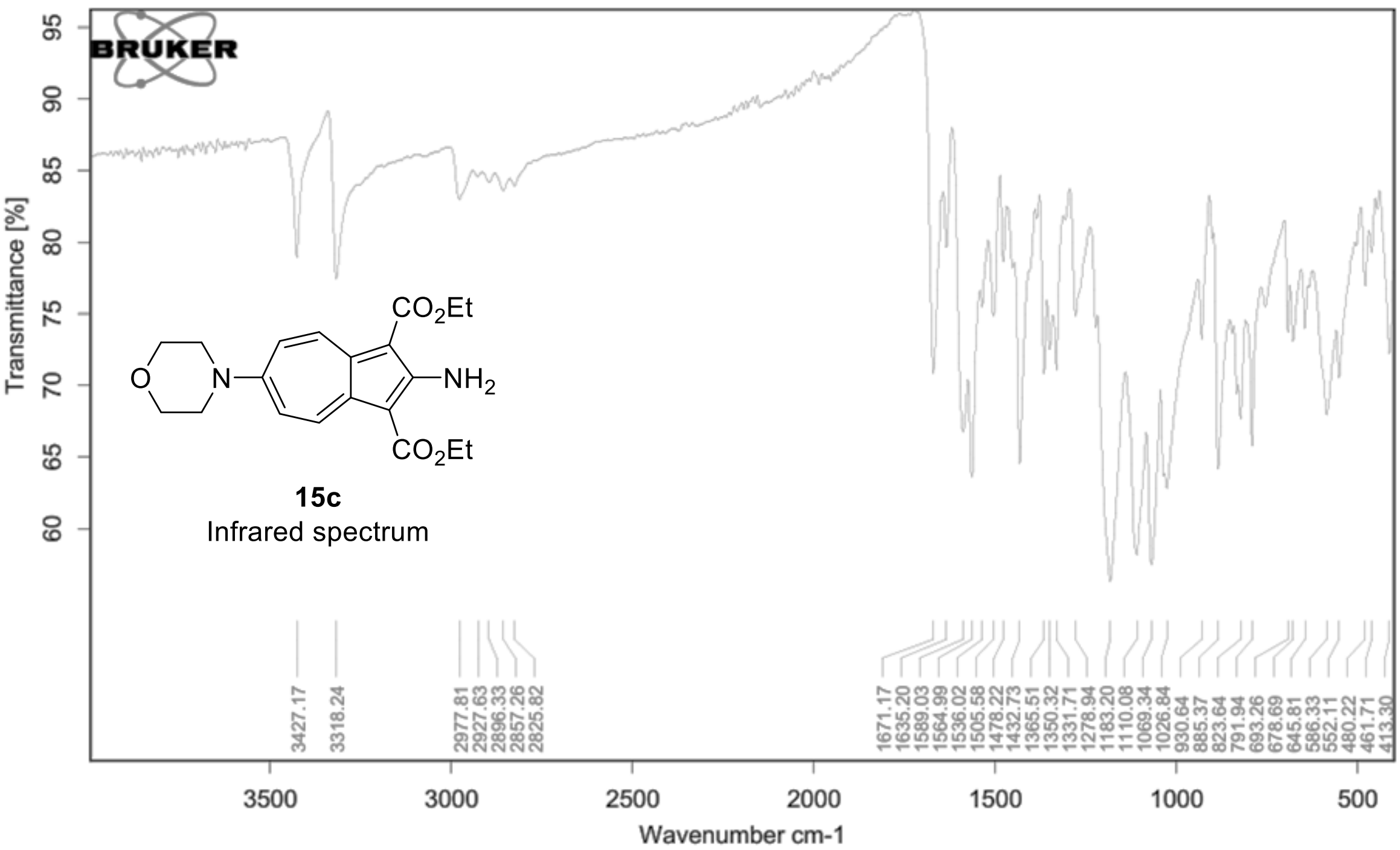
BRUKER
Transmittance [%]
CO2Et
NH2
CO2Et
O
N
15c
Infrared spectrum
3427.17
3318.24
2977.81
2927.63
2896.33
2857.26
2825.82
1671.17
1635.20
1589.03
1564.99
1536.02
1505.58
1478.22
1432.73
1365.51
1350.32
1331.71
1278.94
1183.20
1110.08
1069.34
1026.84
930.64
885.37
823.64
791.94
693.26
678.69
645.81
586.33
552.11
480.22
461.71
413.30
Wavenumber cm-1

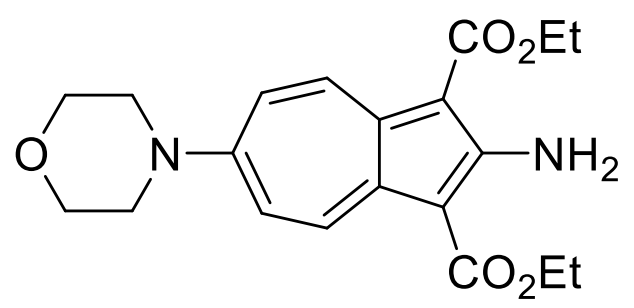

15c
HRMS-ESI+

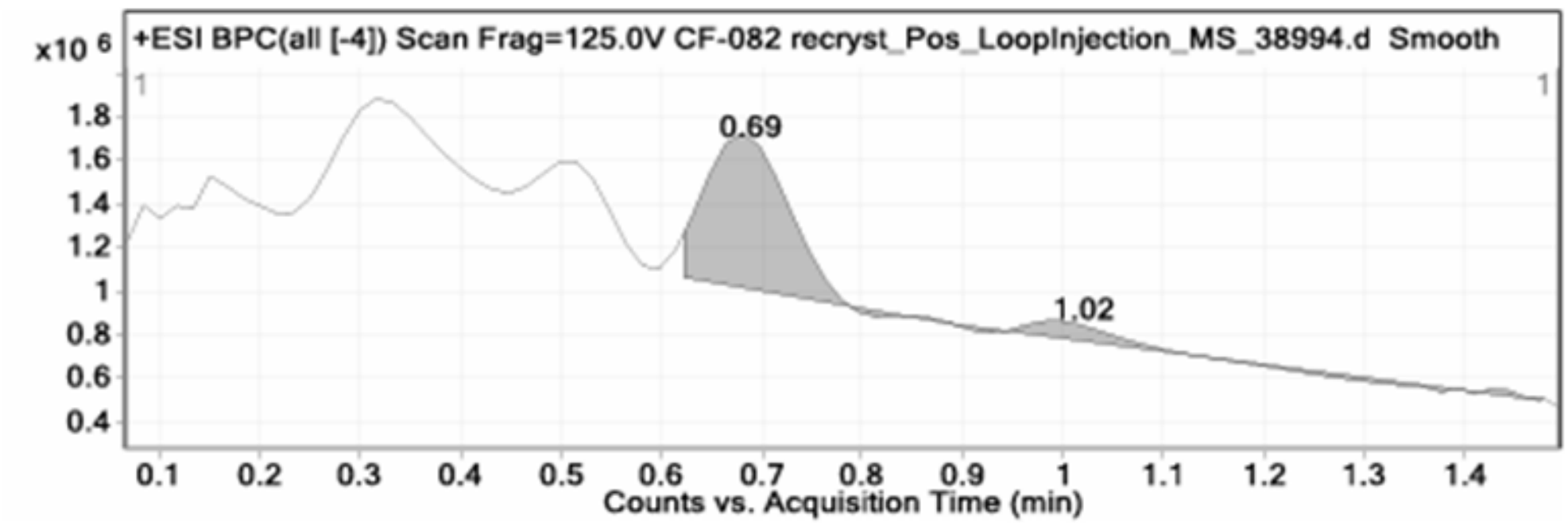

Figure 1: Base peak chromatogram

User Chromatogram Peak List

RT (min)	Area	Area %	Area Sum (%)	Base Peak (m/z)	Width (min)
0.69	3946972	100.00	93.88	185.1385	0.100
1.02	257135	6.51	6.12	185.1385	0.110

Compound Table

Compound Label	RT (min)	Observed mass (m/z)	Neutral observed mass (Da)	Theoretical mass (Da)	Mass error (ppm)	Isotope match score (%)
Cpd 1: C20 H24 N2 O5	0.76	373.1756	372.1682	372.1685	-0.93	99.42

Mass errors of between -5.00 and 5.00 ppm with isotope match scores above 60% are considered confirmation of molecular formulae

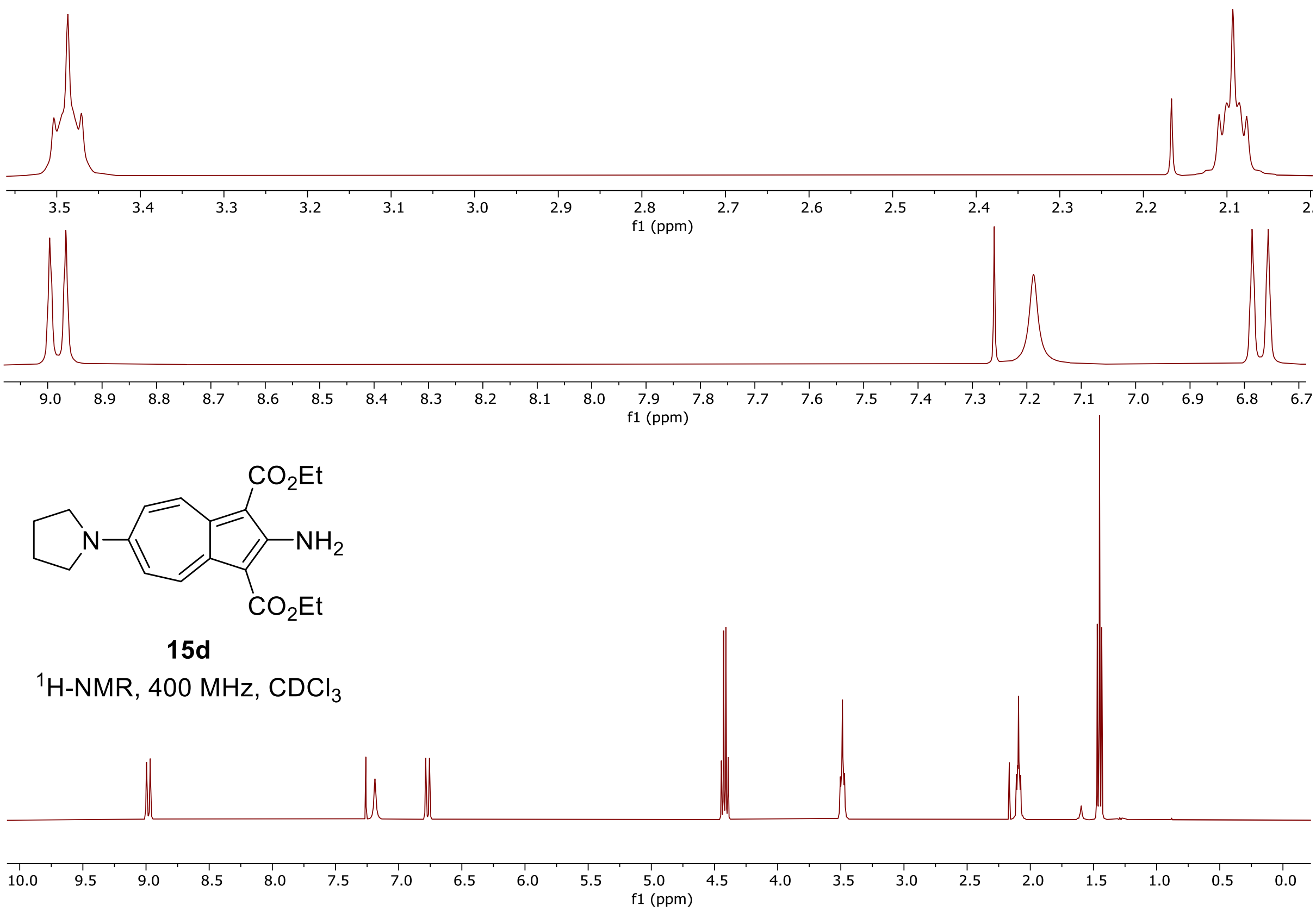

15d
^{1}H-NMR, 400 MHz, $CDCl_3$

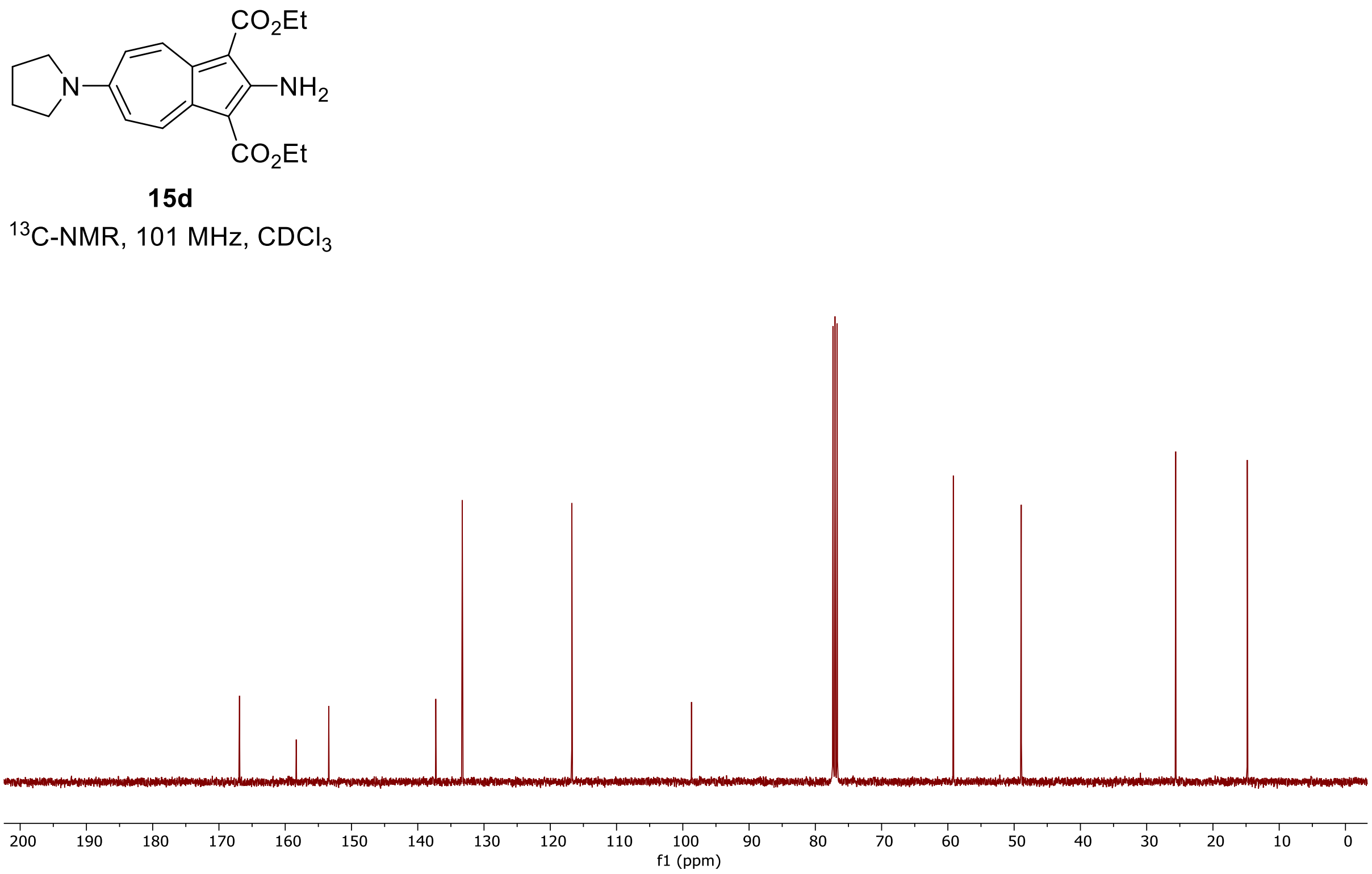

15d
^{13}C-NMR, 101 MHz, $CDCl_3$

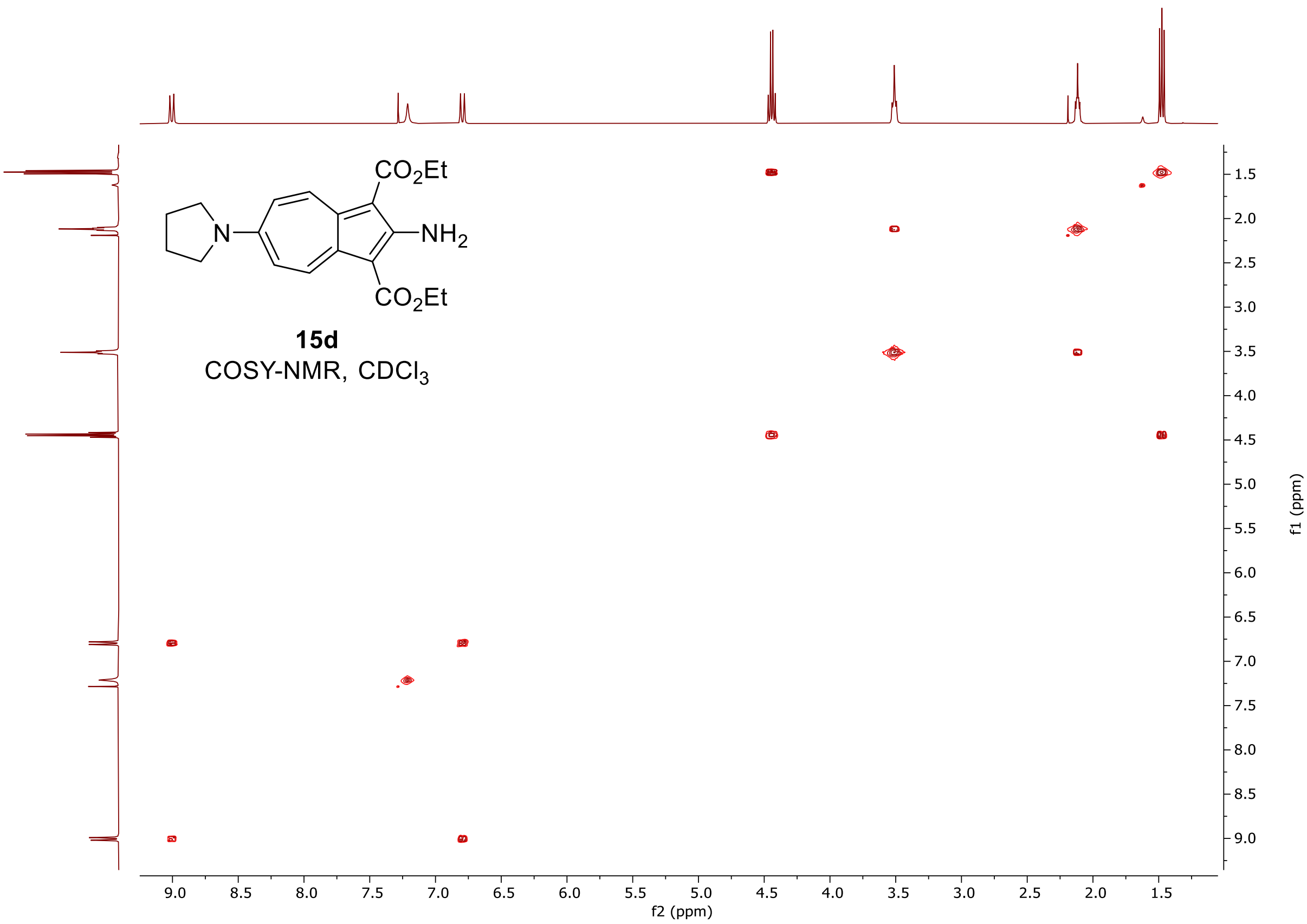
CO2Et
N
NH2
CO2Et
15d
COSY-NMR, CDCl3
f2 (ppm)
f1 (ppm)

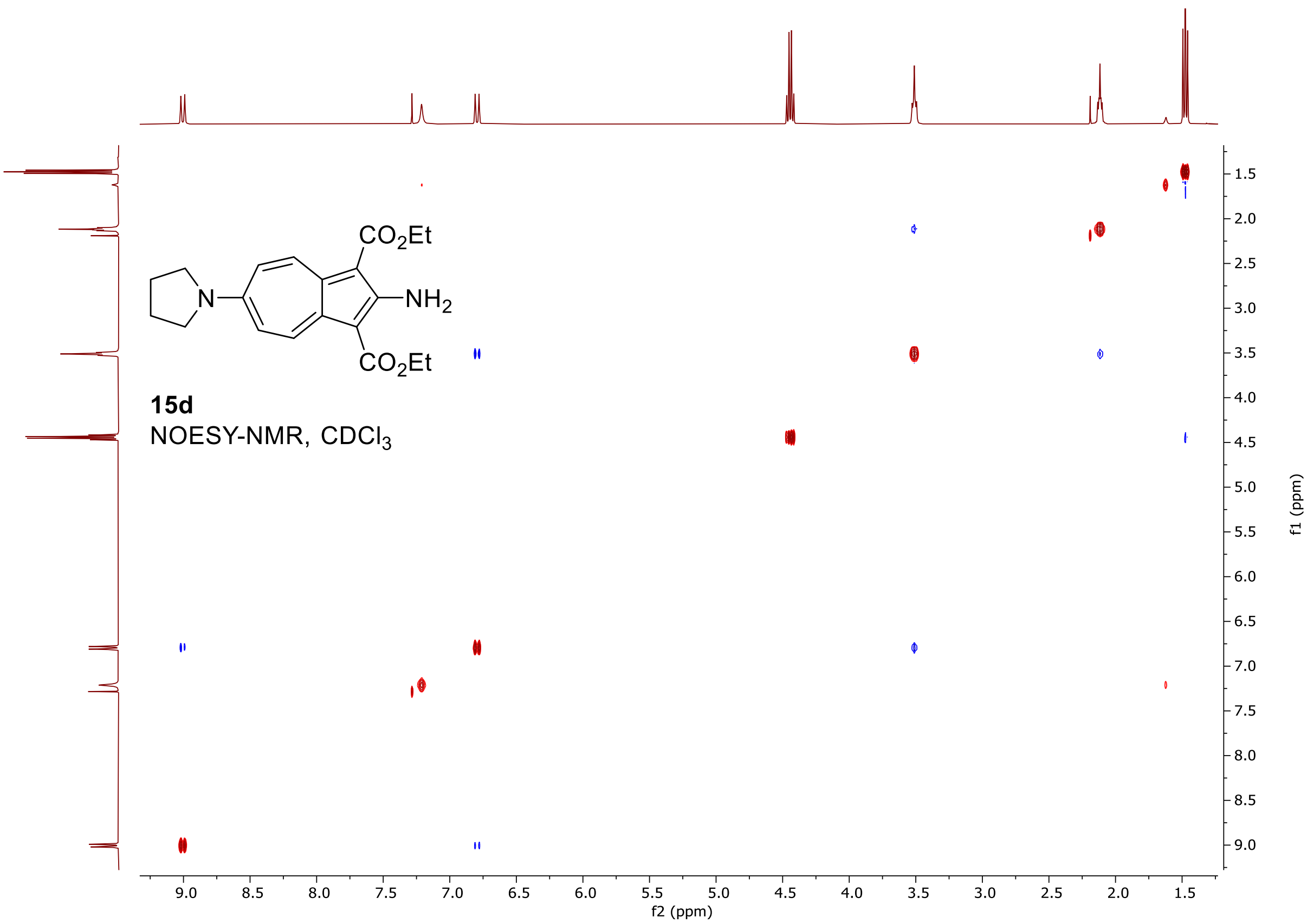
CO2Et
N
NH2
CO2Et
15d
NOESY-NMR, CDCl3
f2 (ppm)
f1 (ppm)

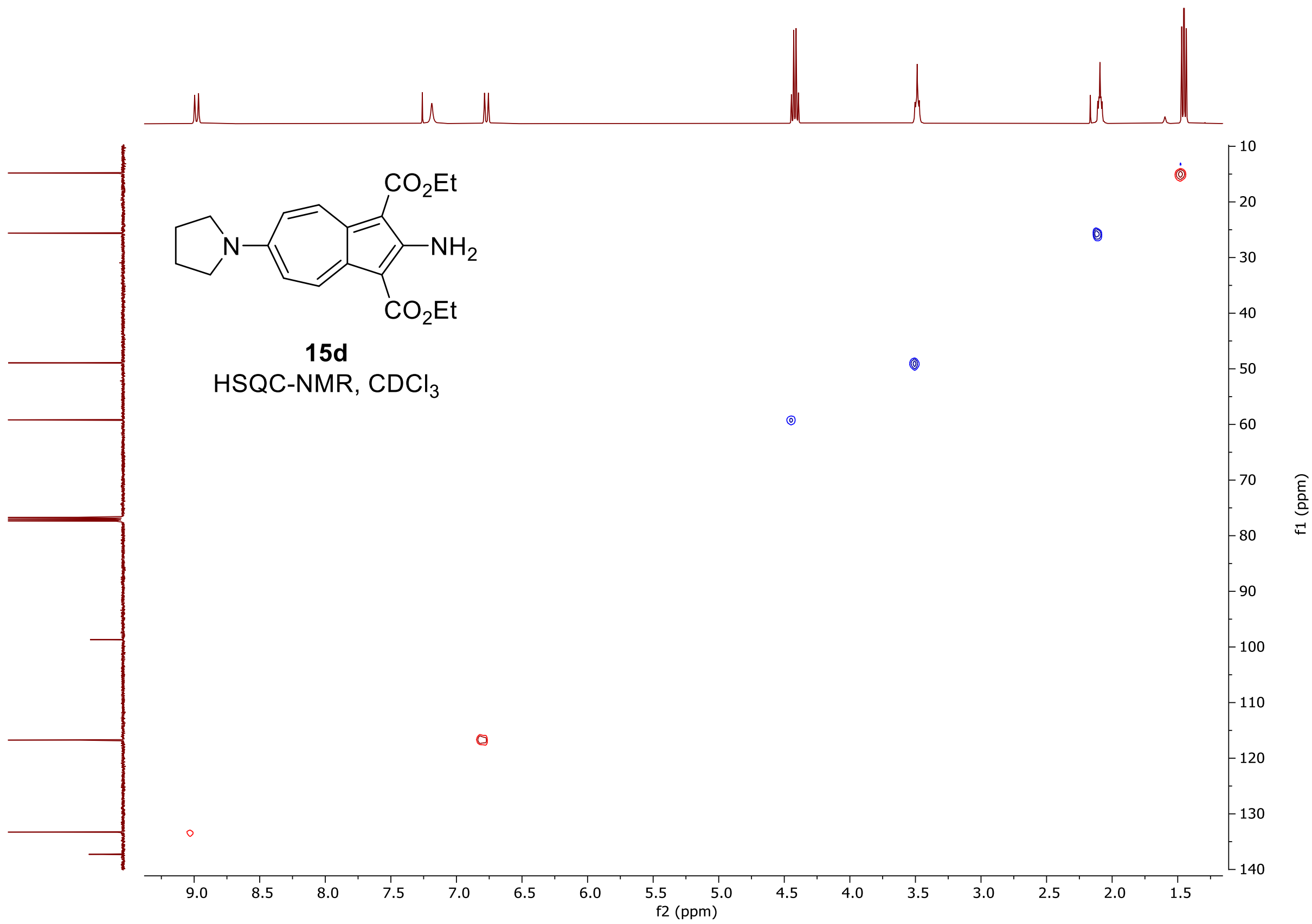

CO2Et
N
NH2
CO2Et
15d
HSQC-NMR, CDCl3
f1 (ppm)
f2 (ppm)

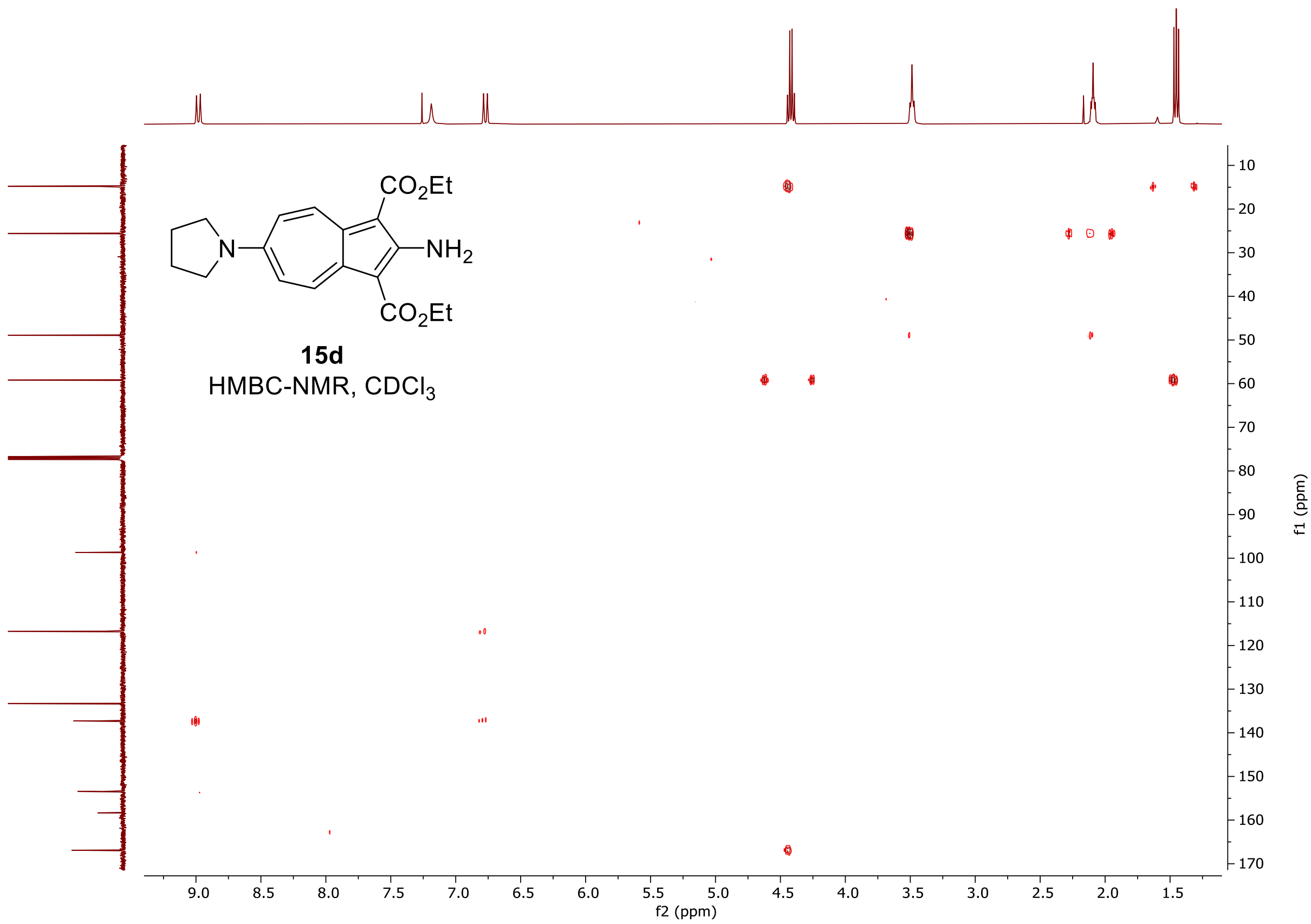

CO2Et
N
NH2
CO2Et
15d
HMBC-NMR, CDCl3
f1 (ppm)
f2 (ppm)

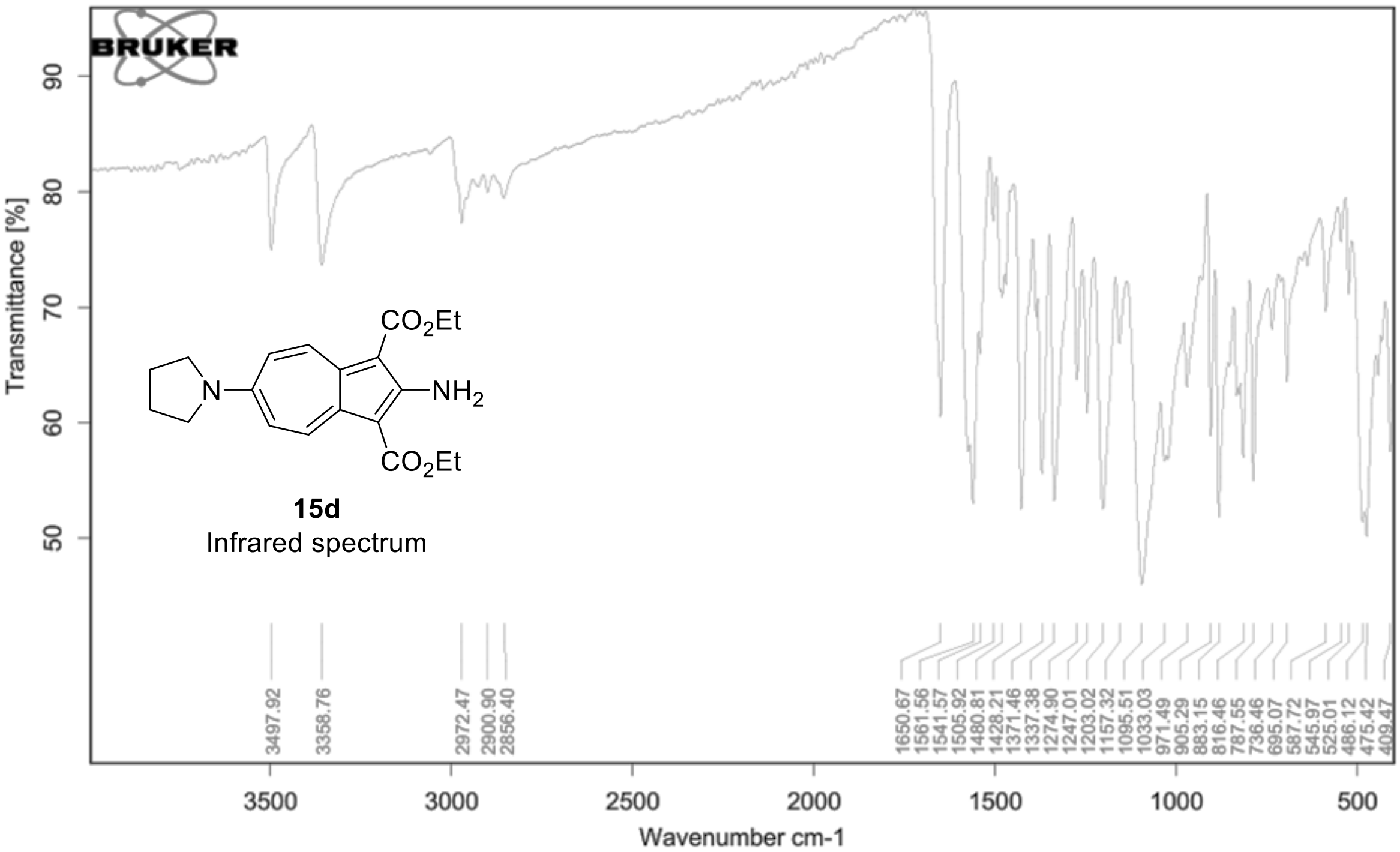

15d
Infrared spectrum

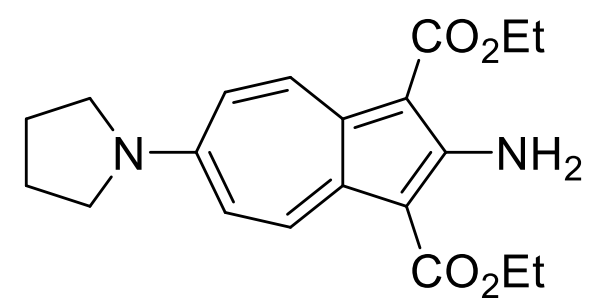

15d
HRMS-ESI$^+$

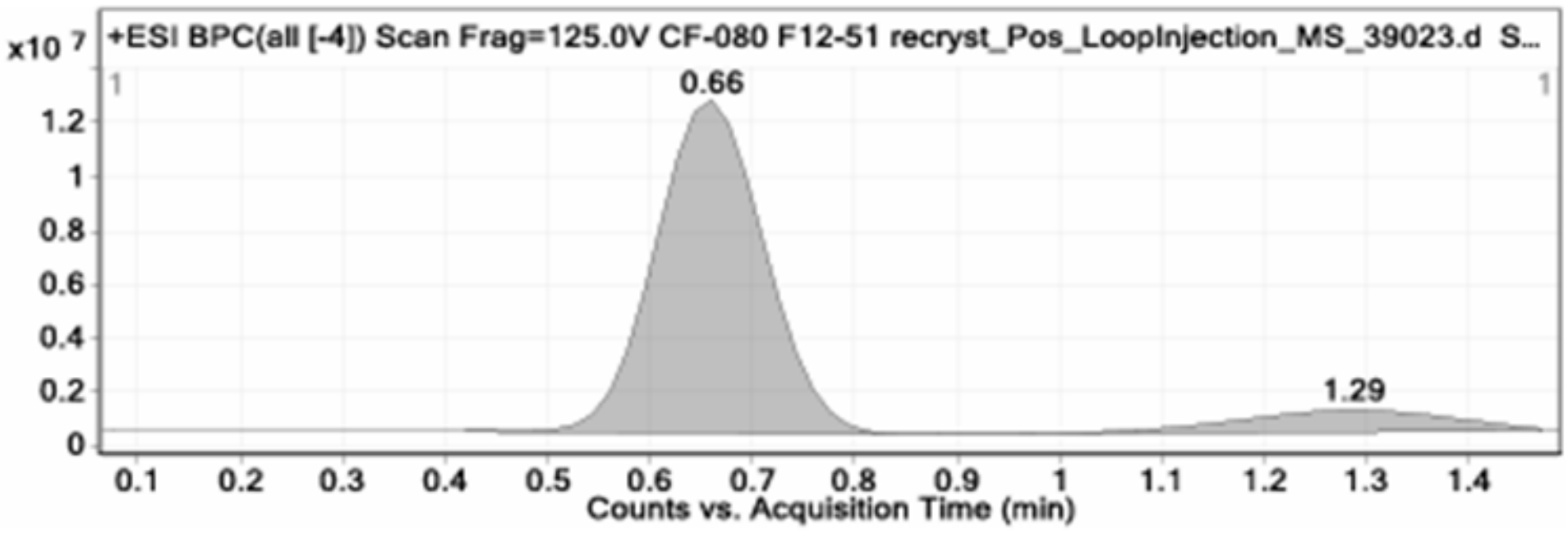

Figure 1: Base peak chromatogram

User Chromatogram Peak List

RT (min)	Area	Area %	Area Sum (%)	Base Peak (m/z)	Width (min)
0.66	92289934	100.00	88.59	357.1803	0.140
1.29	11890847	12.88	11.41	548.5003	0.230

Compound Table

Compound Label	RT (min)	Observed mass (m/z)	Neutral observed mass (Da)	Theoretical mass (Da)	Mass error (ppm)	Isotope match score (%)
Cpd 1: C20 H24 N2 O4	0.66	379.1630	356.1736	356.1736	-0.08	99.25

Mass errors of between -5.00 and 5.00 ppm with isotope match scores above 60% are considered confirmation of molecular formulae

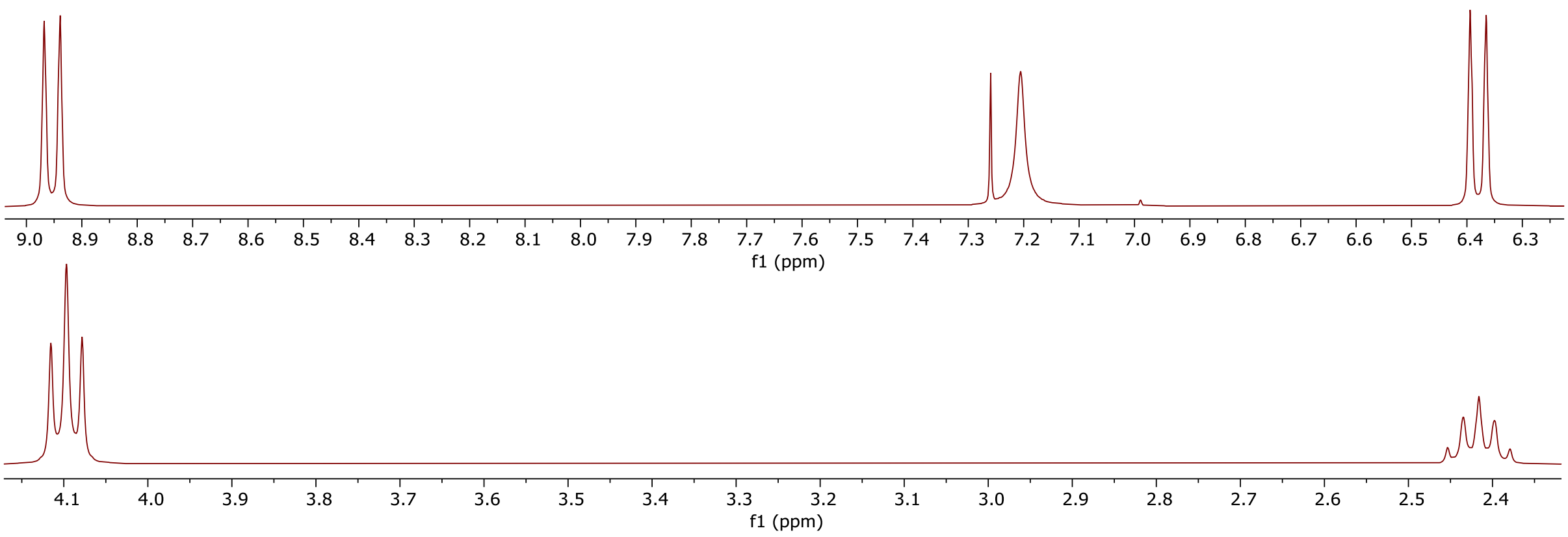

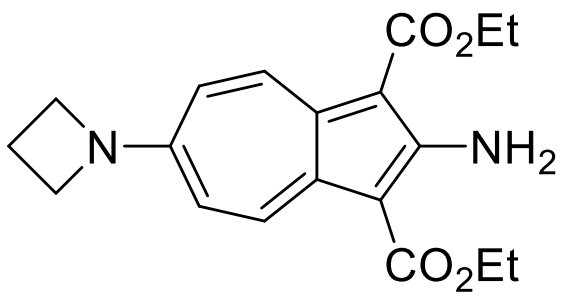

15e

^{1}H-NMR, 400 MHz, $CDCl_3$

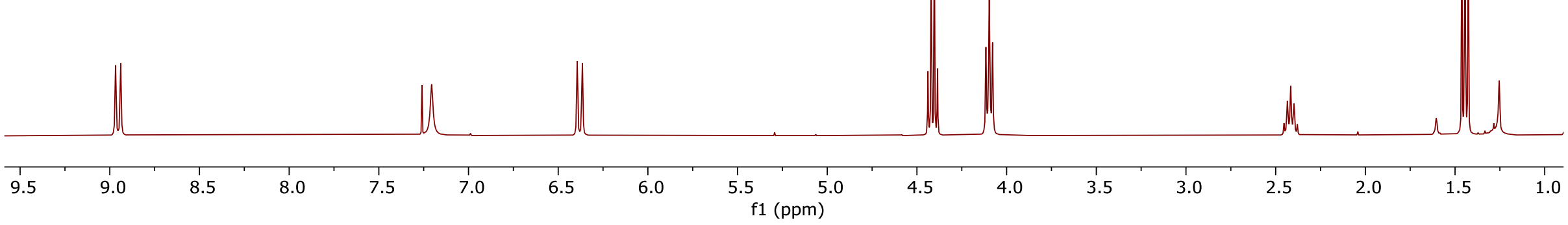

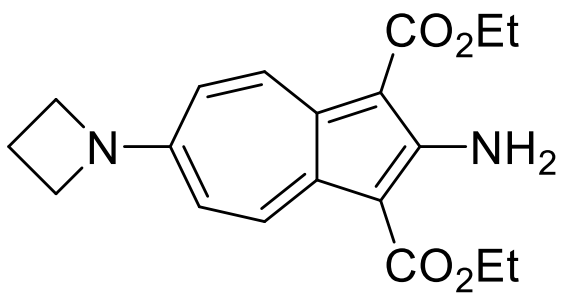

15e

^{13}C-NMR, 101 MHz, $CDCl_3$

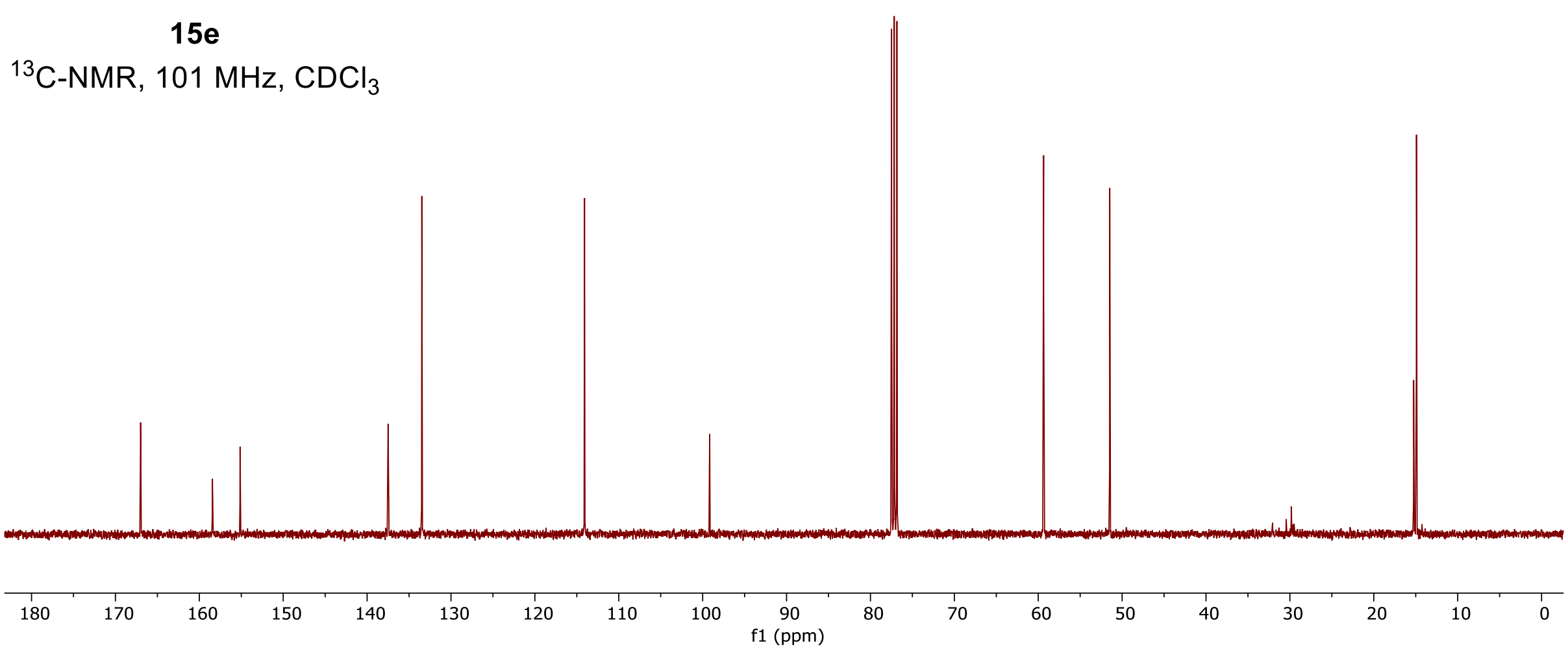

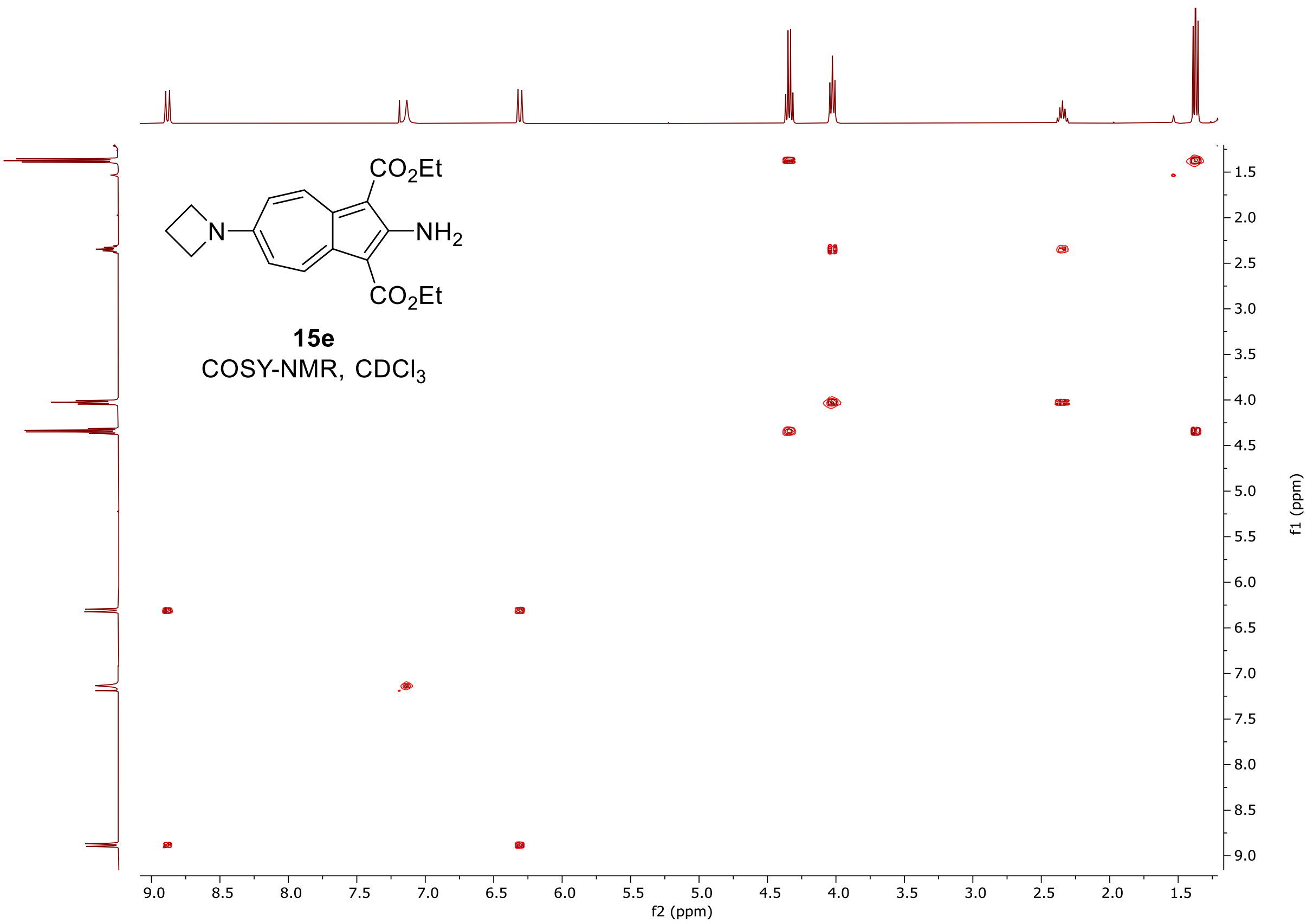
CO2Et
N
NH2
CO2Et
15e
COSY-NMR, CDCl3
f2 (ppm)
f1 (ppm)
9.0
8.5
8.0
7.5
7.0
6.5
6.0
5.5
5.0
4.5
4.0
3.5
3.0
2.5
2.0
1.5

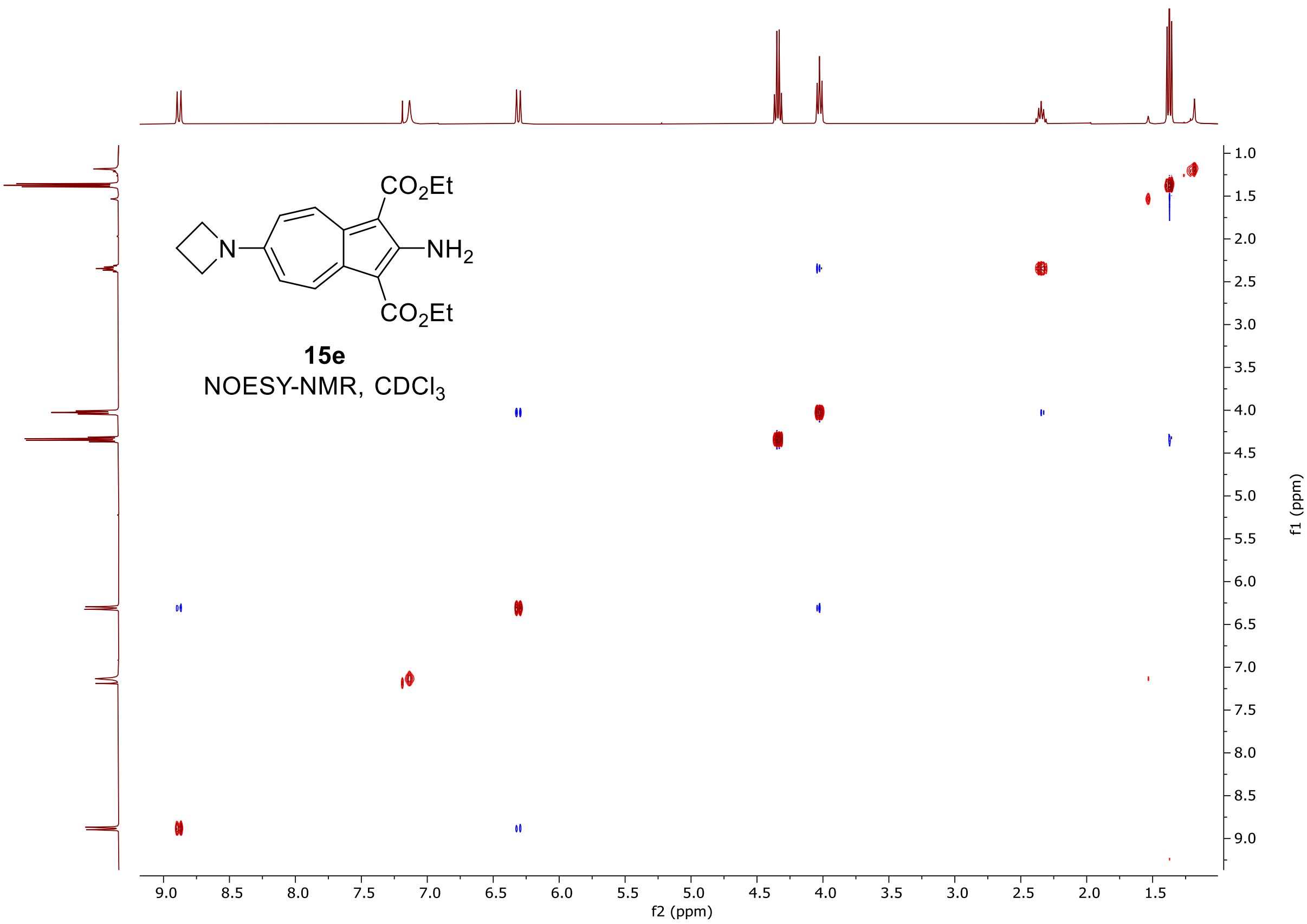
CO2Et
N
NH2
CO2Et
15e
NOESY-NMR, CDCl3
f2 (ppm)
f1 (ppm)
9.0
8.5
8.0
7.5
7.0
6.5
6.0
5.5
5.0
4.5
4.0
3.5
3.0
2.5
2.0
1.5
1.0

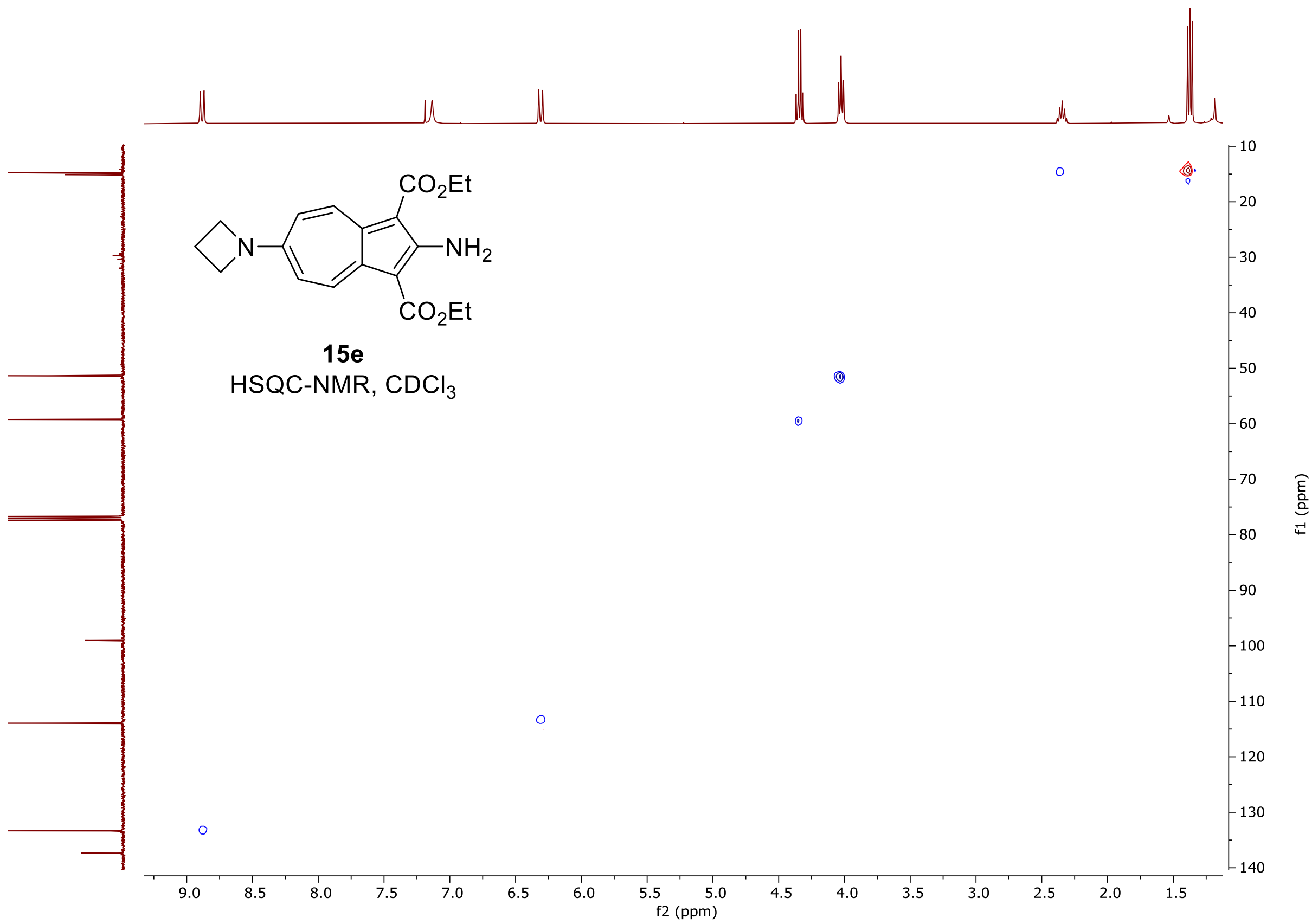
CO2Et
NH2
CO2Et
N
15e
HSQC-NMR, CDCl3
f2 (ppm)
f1 (ppm)

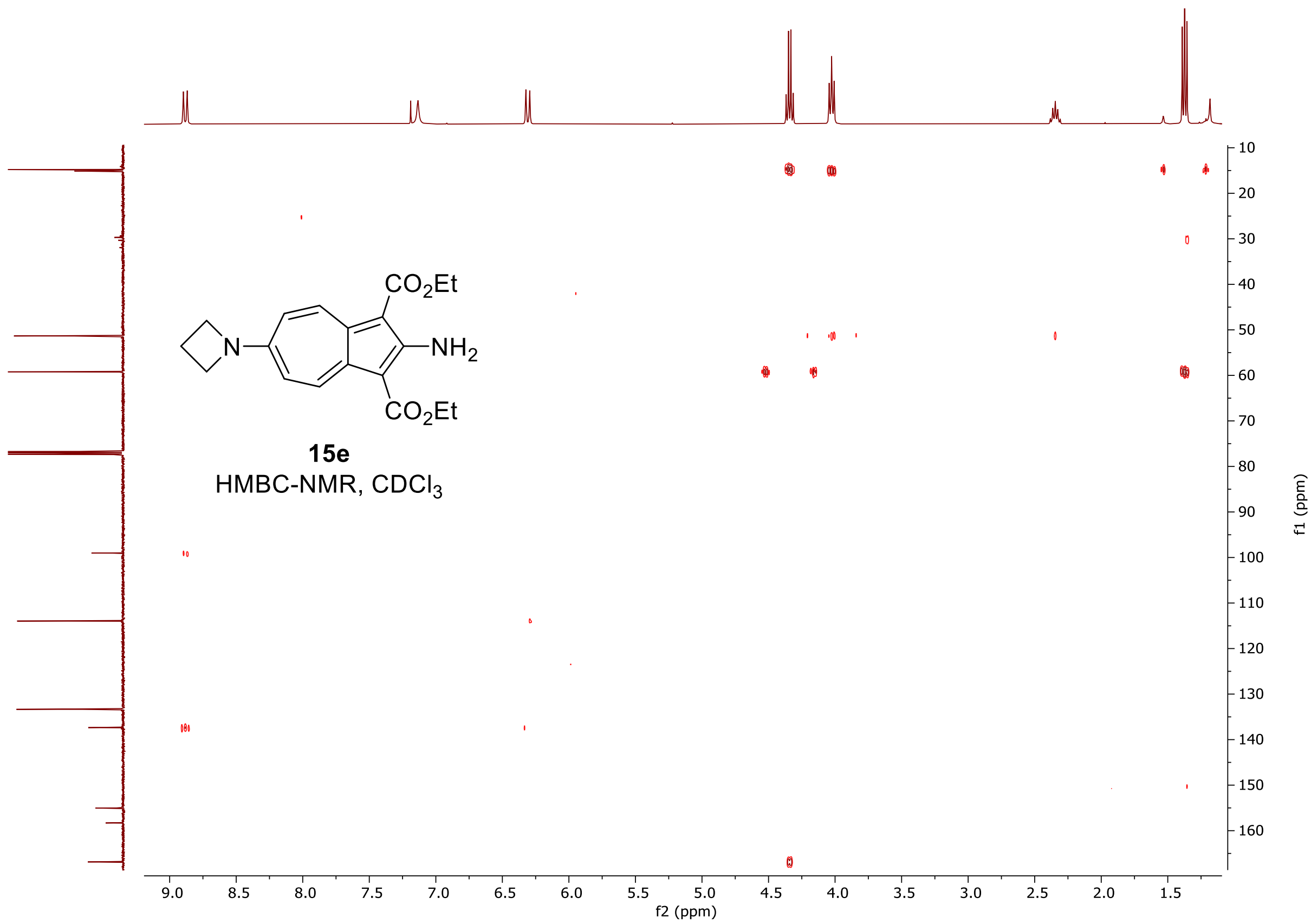
CO2Et
NH2
CO2Et
N
15e
HMBC-NMR, CDCl3
f2 (ppm)
f1 (ppm)

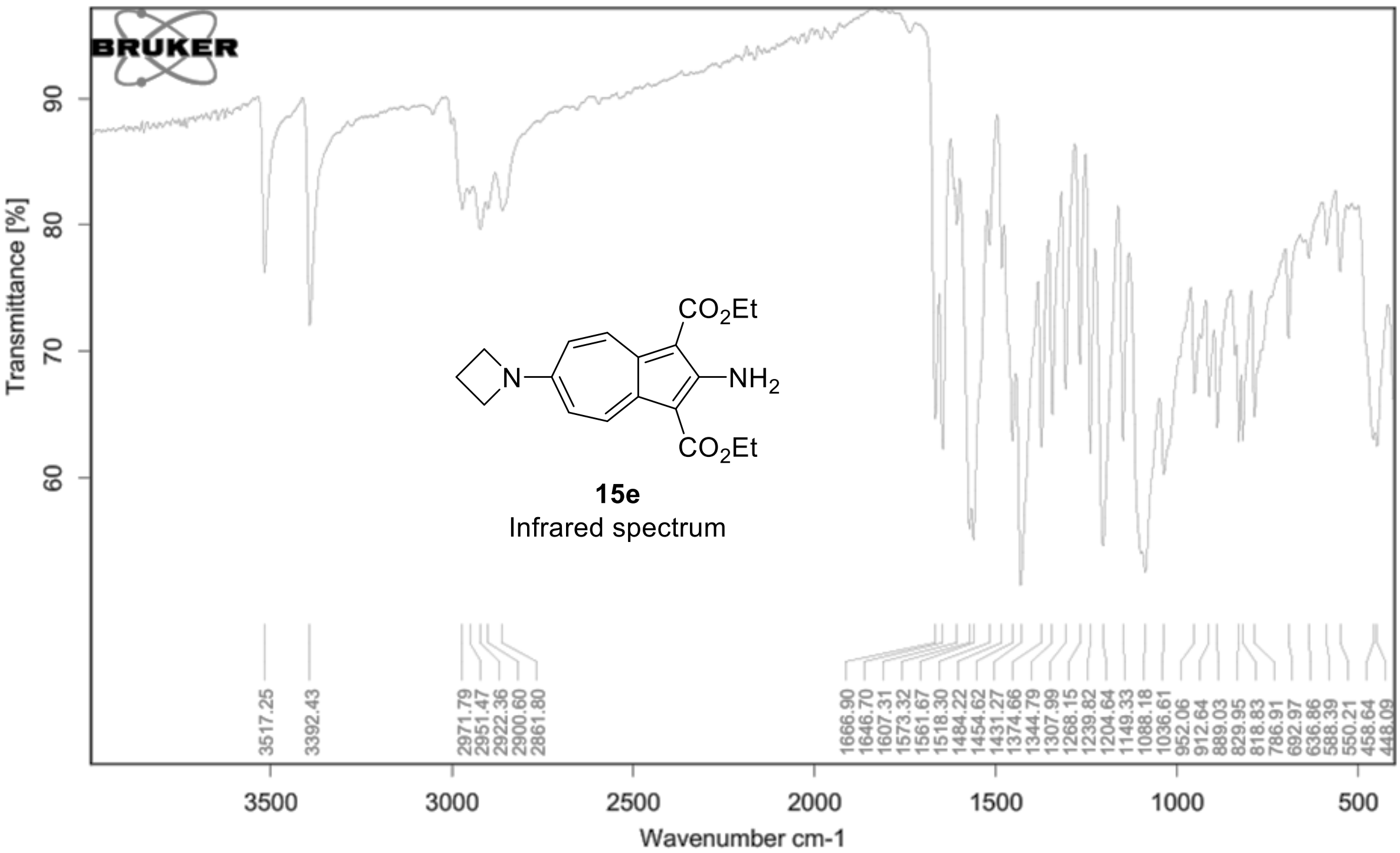

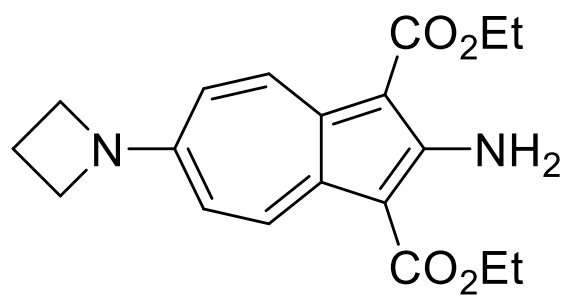

15e
HRMS-ESI$^+$

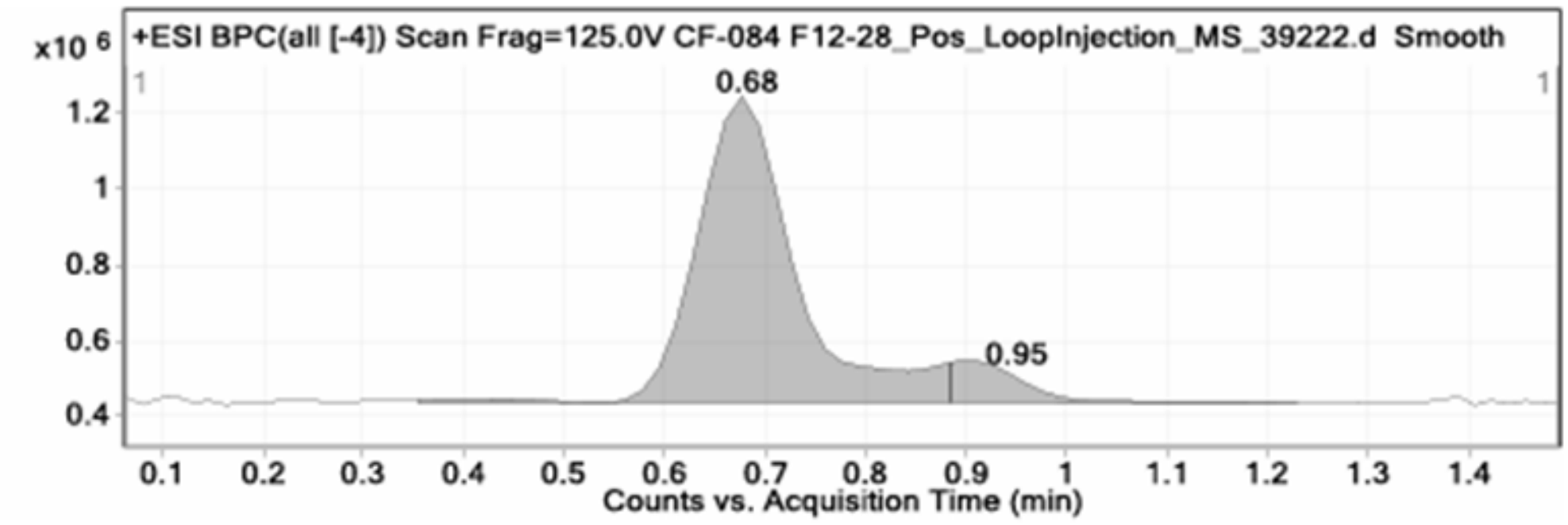

Figure 1: Base peak chromatogram

User Chromatogram Peak List

RT (min)	Area	Area %	Area Sum (%)	Base Peak (m/z)	Width (min)
0.68	5552442	100.00	91.15	371.1952	0.150
0.95	539031	9.71	8.85	922.0081	0.140

Compound Table

Compound Label	RT (min)	Observed mass (m/z)	Neutral observed mass (Da)	Theoretical mass (Da)	Mass error (ppm)	Isotope match score (%)
Cpd 1: C19 H22 N2 O4	0.69	343.1645	342.1574	342.1580	-1.58	98.96

Mass errors of between -5.00 and 5.00 ppm with isotope match scores above 60% are considered confirmation of molecular formulae

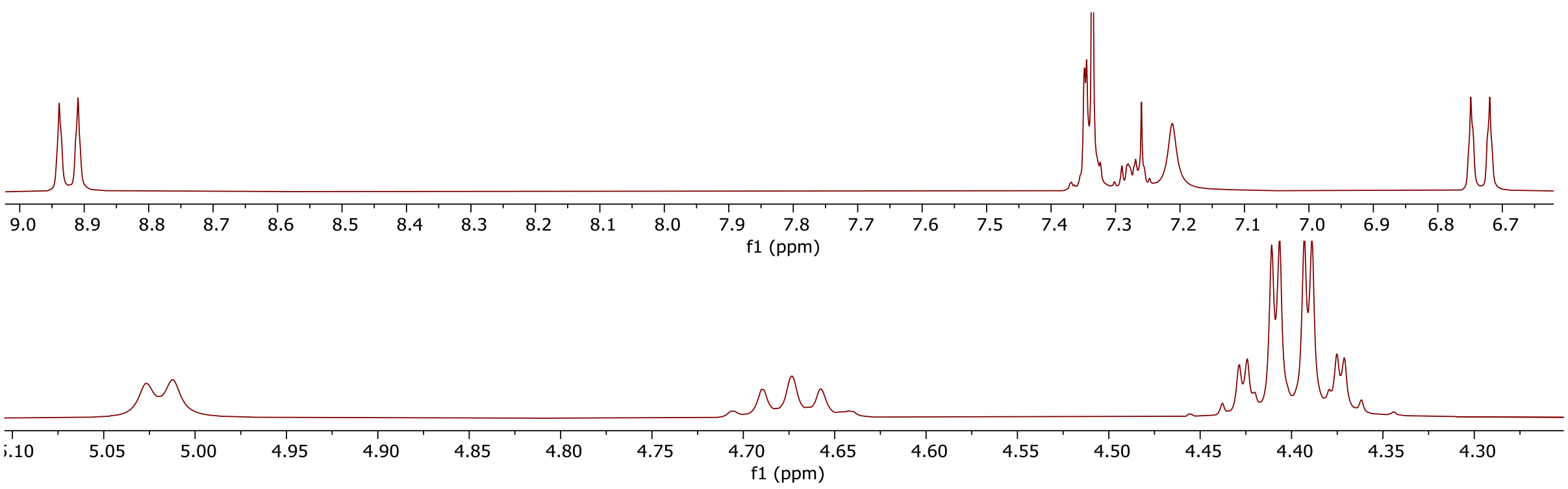

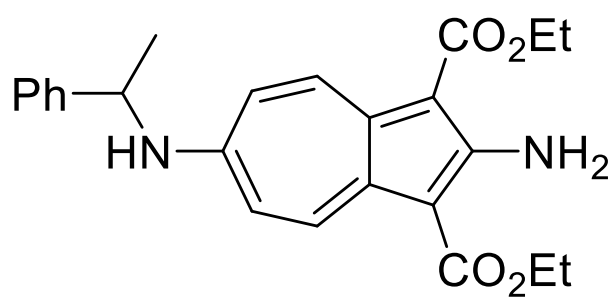

15f

^{1}H-NMR, 400 MHz, $CDCl_3$

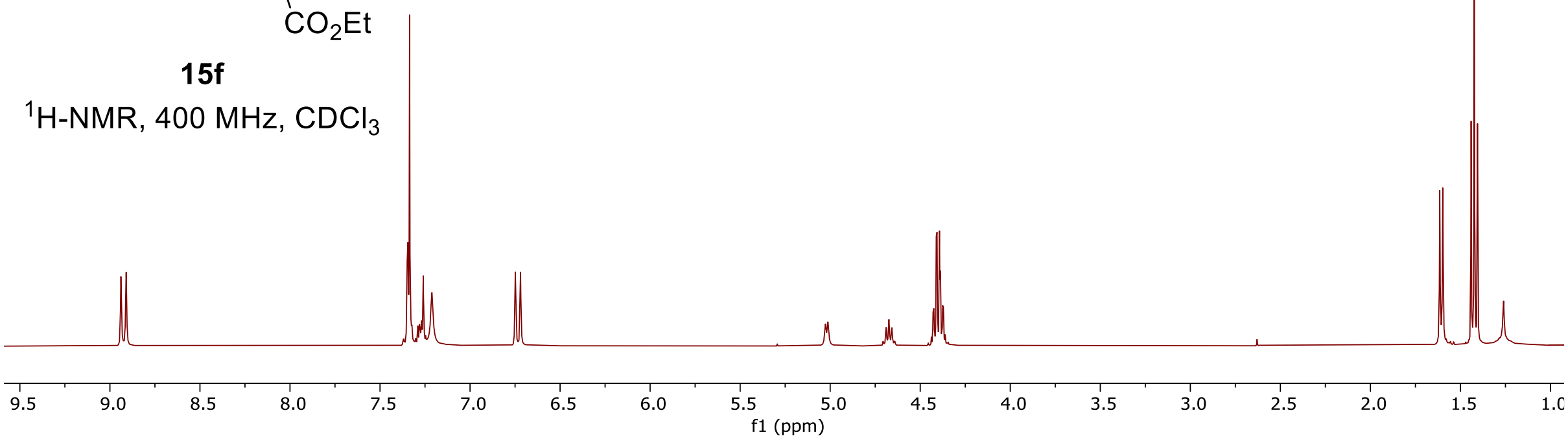

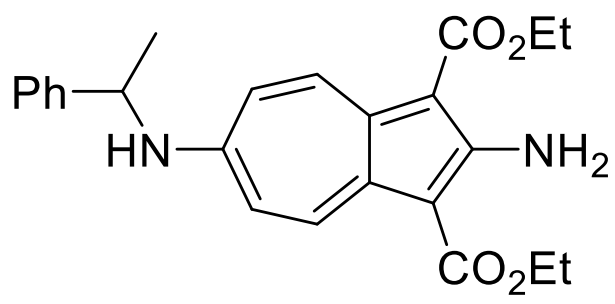

15f

^{13}C-NMR, 101 MHz, $CDCl_3$

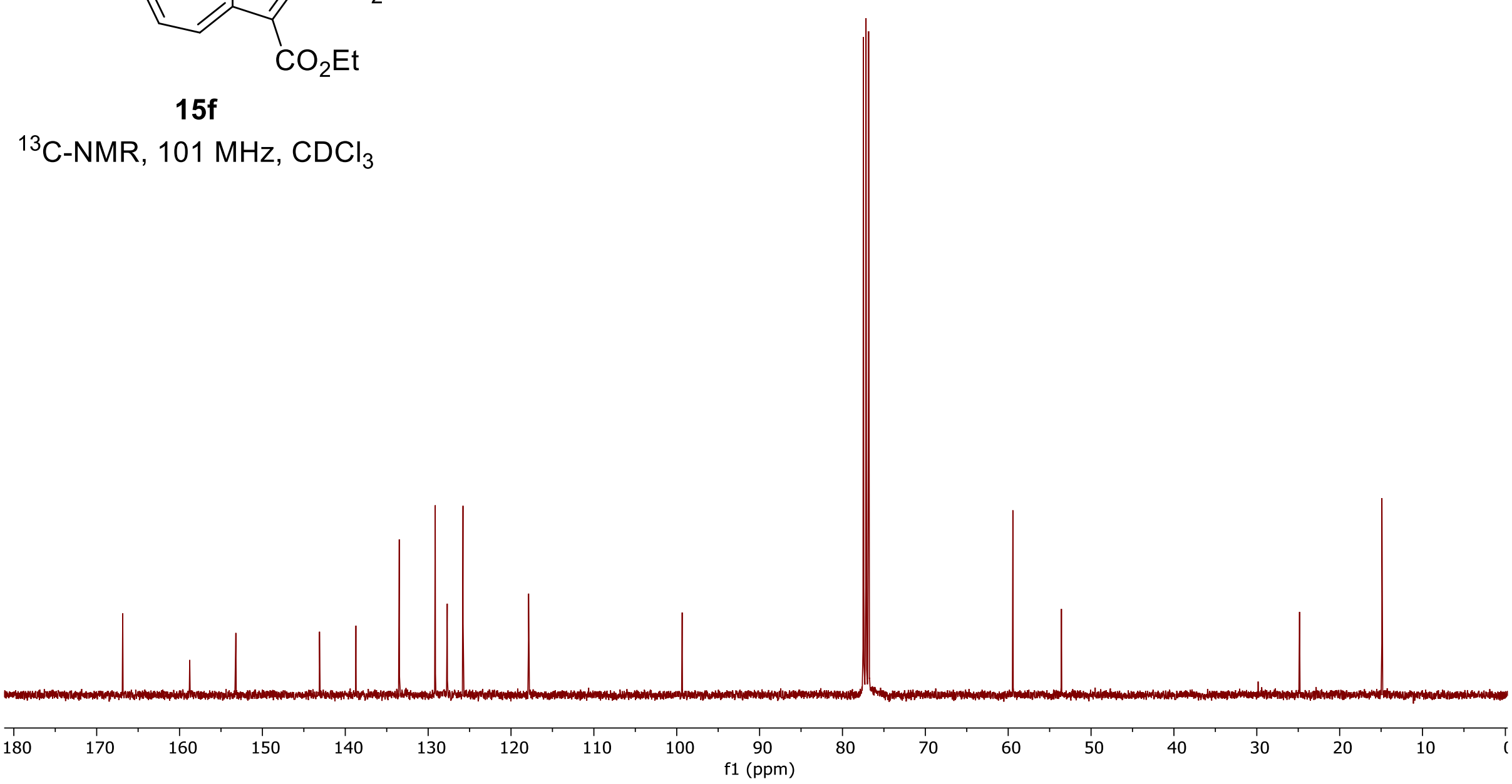

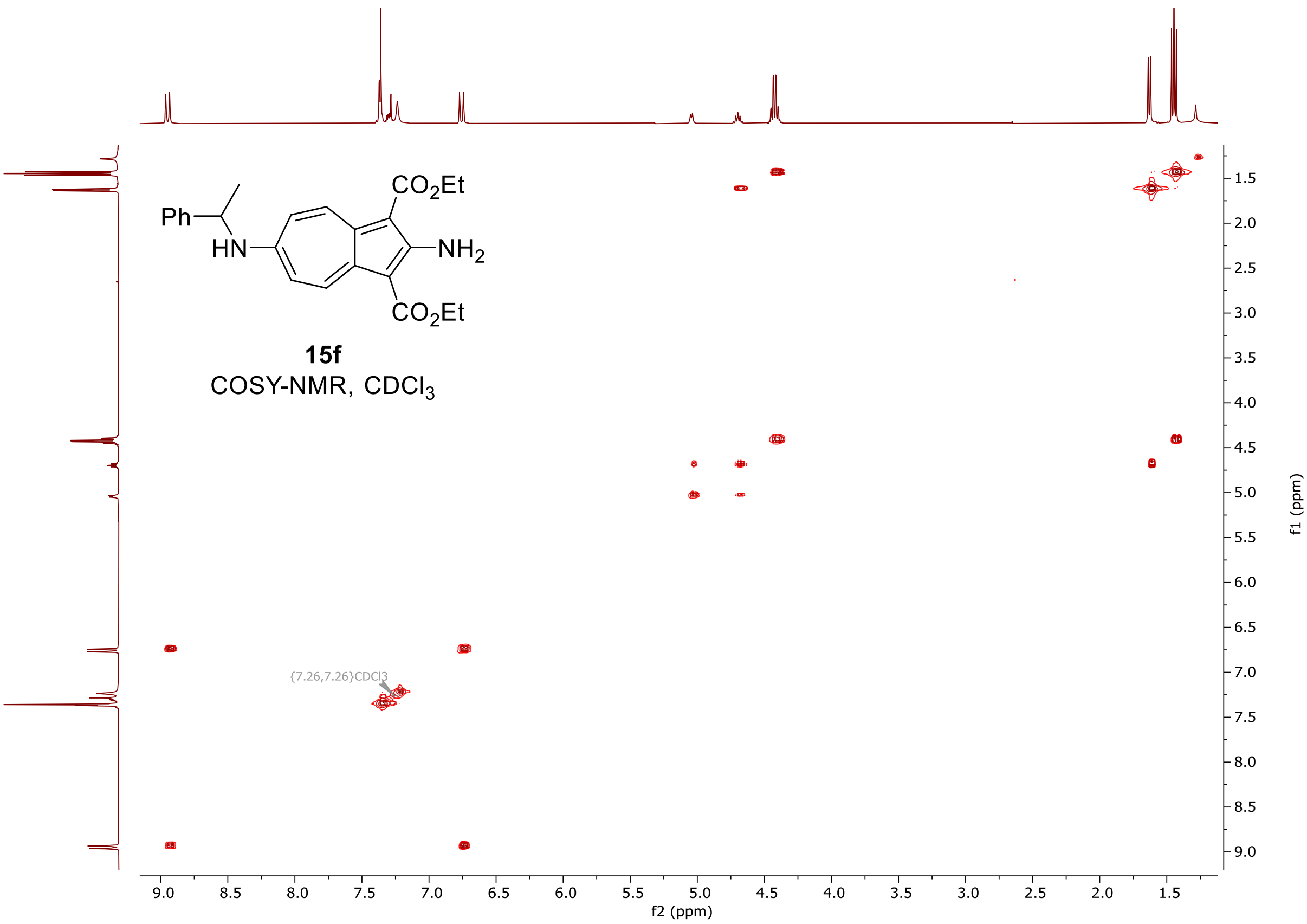

Ph
HN
CO_2Et
NH_2
CO_2Et
15f
COSY-NMR, $CDCl_3$
{7.26,7.26}CDCl3
f2 (ppm)
f1 (ppm)

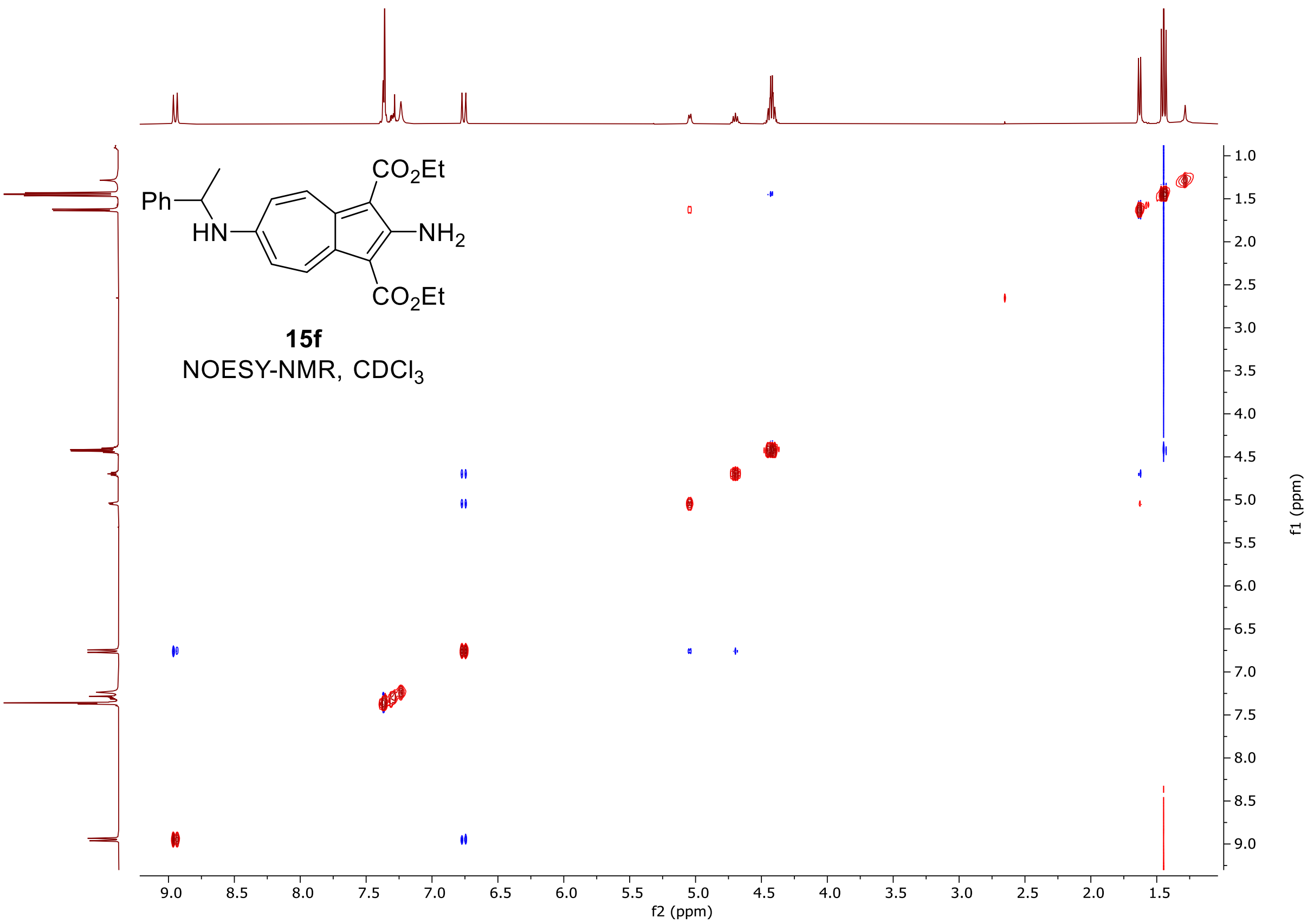

Ph
HN
CO_2Et
NH_2
CO_2Et
15f
NOESY-NMR, $CDCl_3$
f2 (ppm)
f1 (ppm)

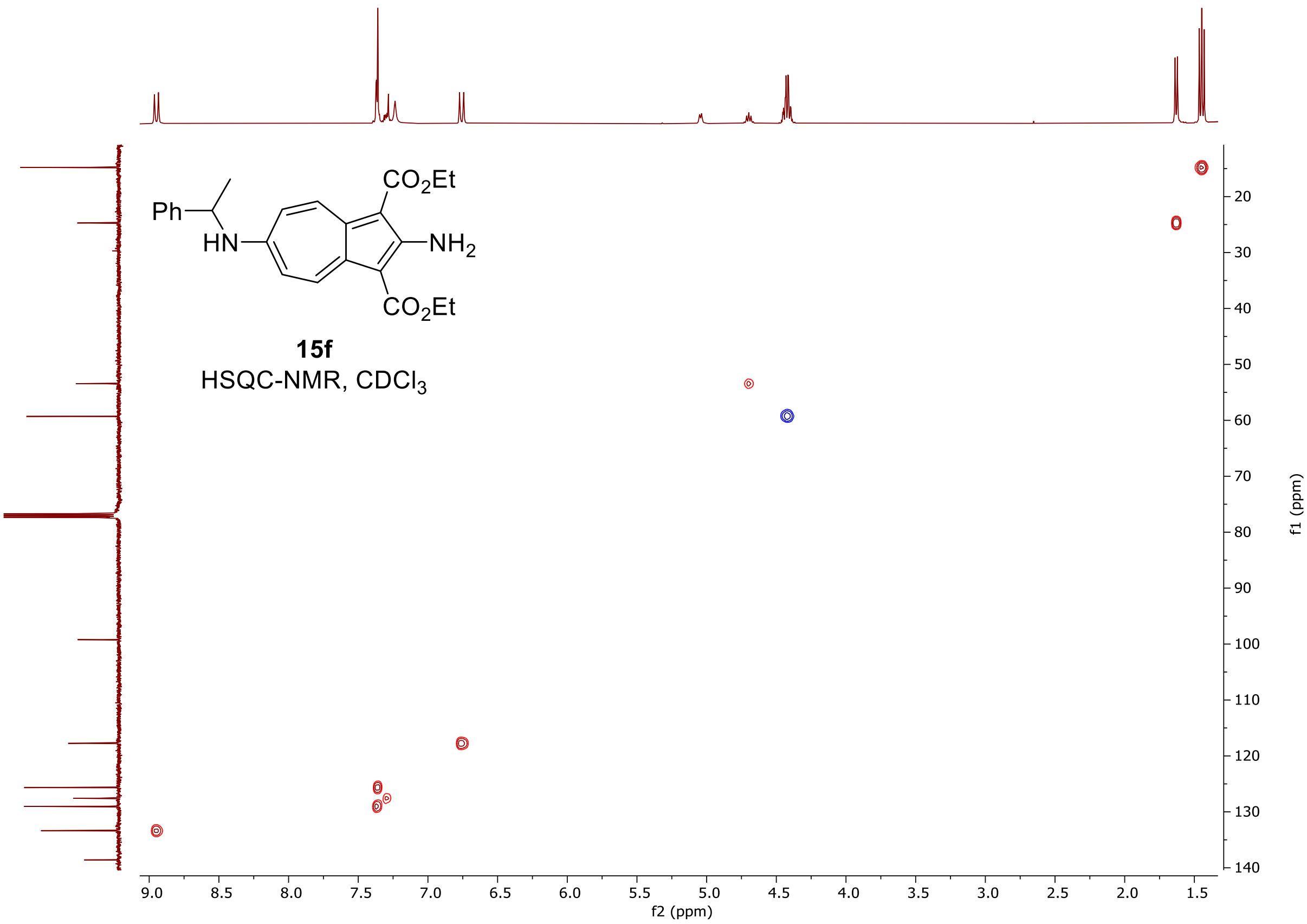

CO2Et
Ph
HN
NH2
CO2Et
15f
HSQC-NMR, CDCl3
f2 (ppm)
f1 (ppm)

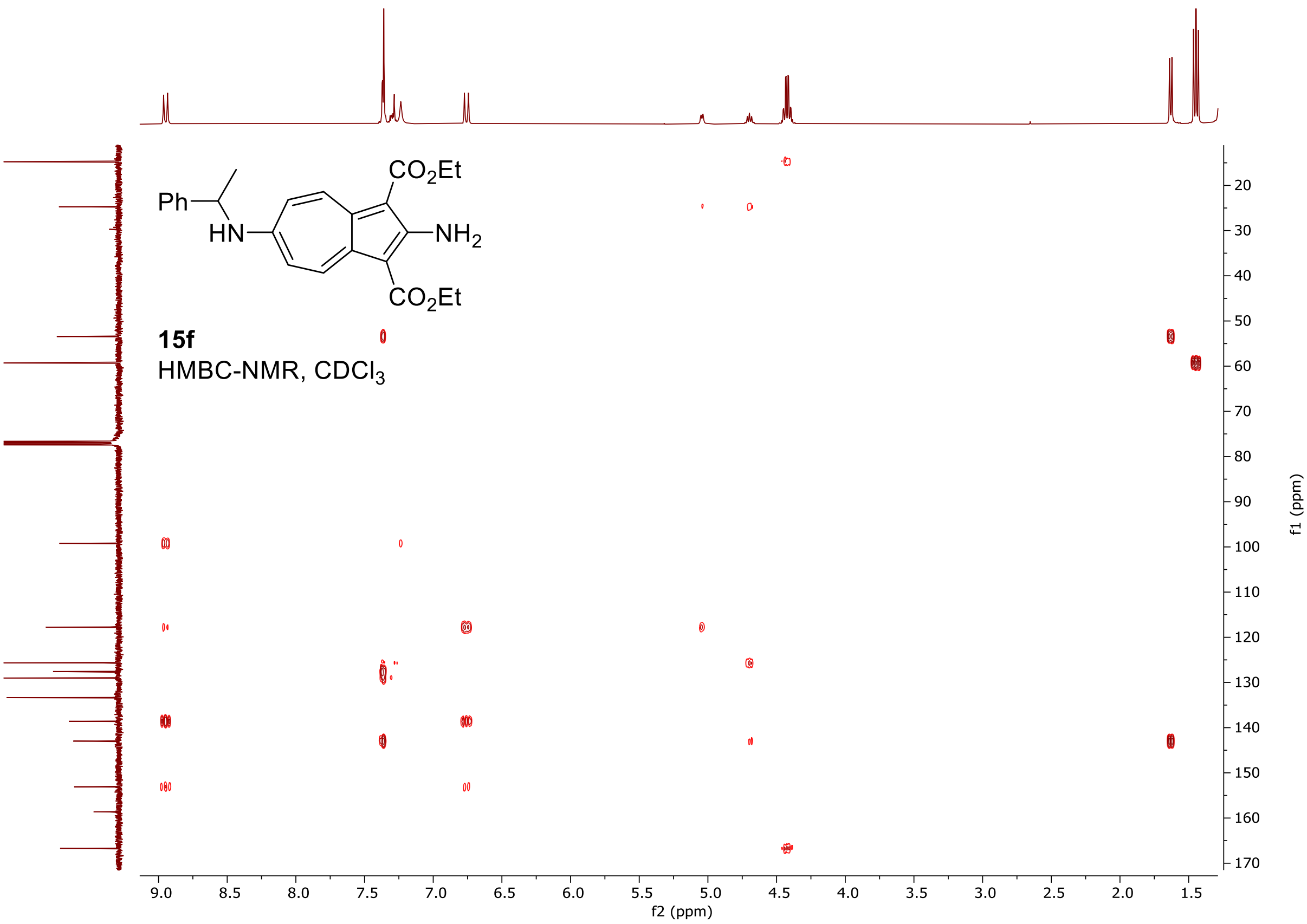

CO2Et
Ph
HN
NH2
CO2Et
15f
HMBC-NMR, CDCl3
f2 (ppm)
f1 (ppm)

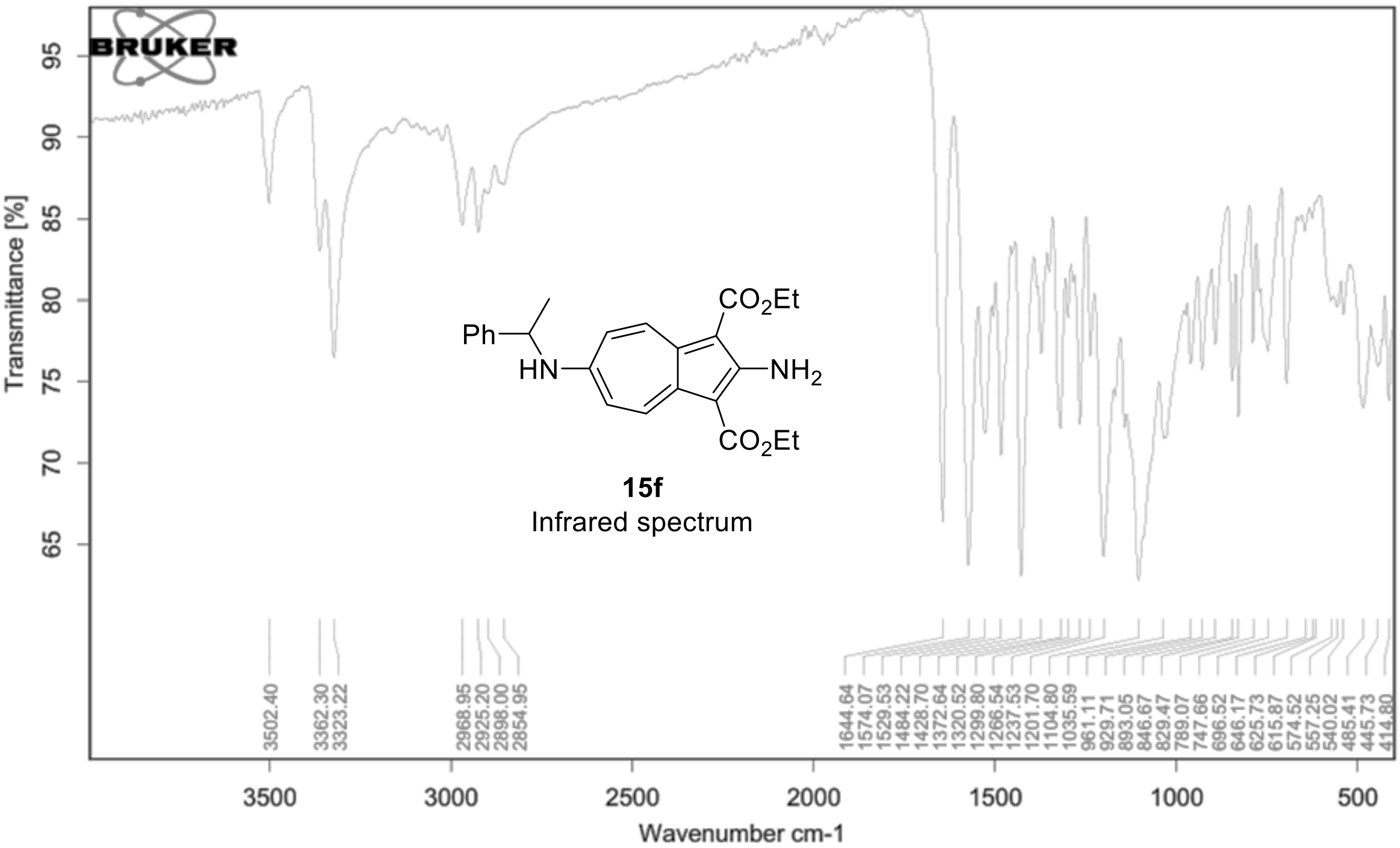

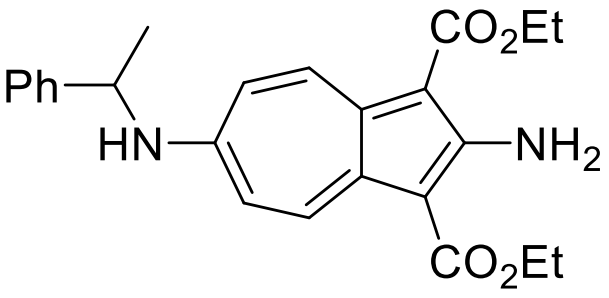

15f
HRMS-ESI$^+$

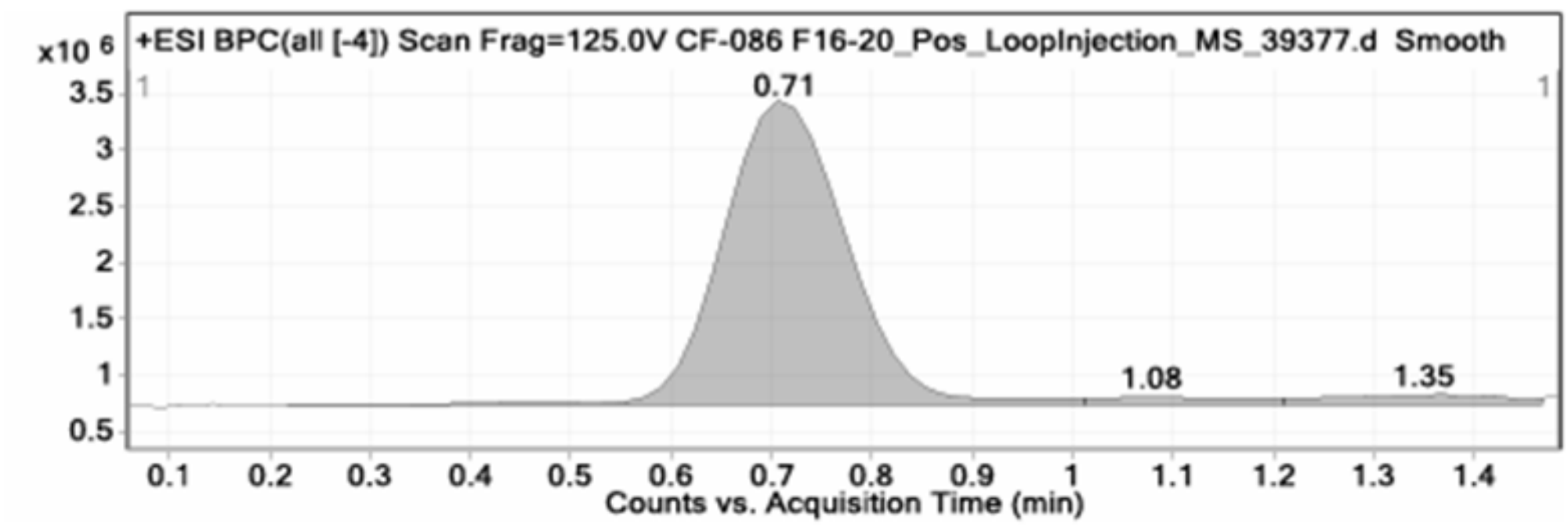

Figure 1: Base peak chromatogram

User Chromatogram Peak List

RT (min)	Area	Area %	Area Sum (%)	Base Peak (m/z)	Width (min)
0.71	23424905	100.00	92.42	407.1923	0.160
1.08	773142	3.30	3.05	922.0081	0.190
1.35	1146994	4.90	4.53	922.0081	0.220

Compound Table

Compound Label	RT (min)	Observed mass (m/z)	Neutral observed mass (Da)	Theoretical mass (Da)	Mass error (ppm)	Isotope match score (%)
Cpd 1: C24 H26 N2 O4	0.71	429.1786	406.1884	406.1893	-2.08	99.79

Mass errors of between -5.00 and 5.00 ppm with isotope match scores above 60% are considered confirmation of molecular formulae

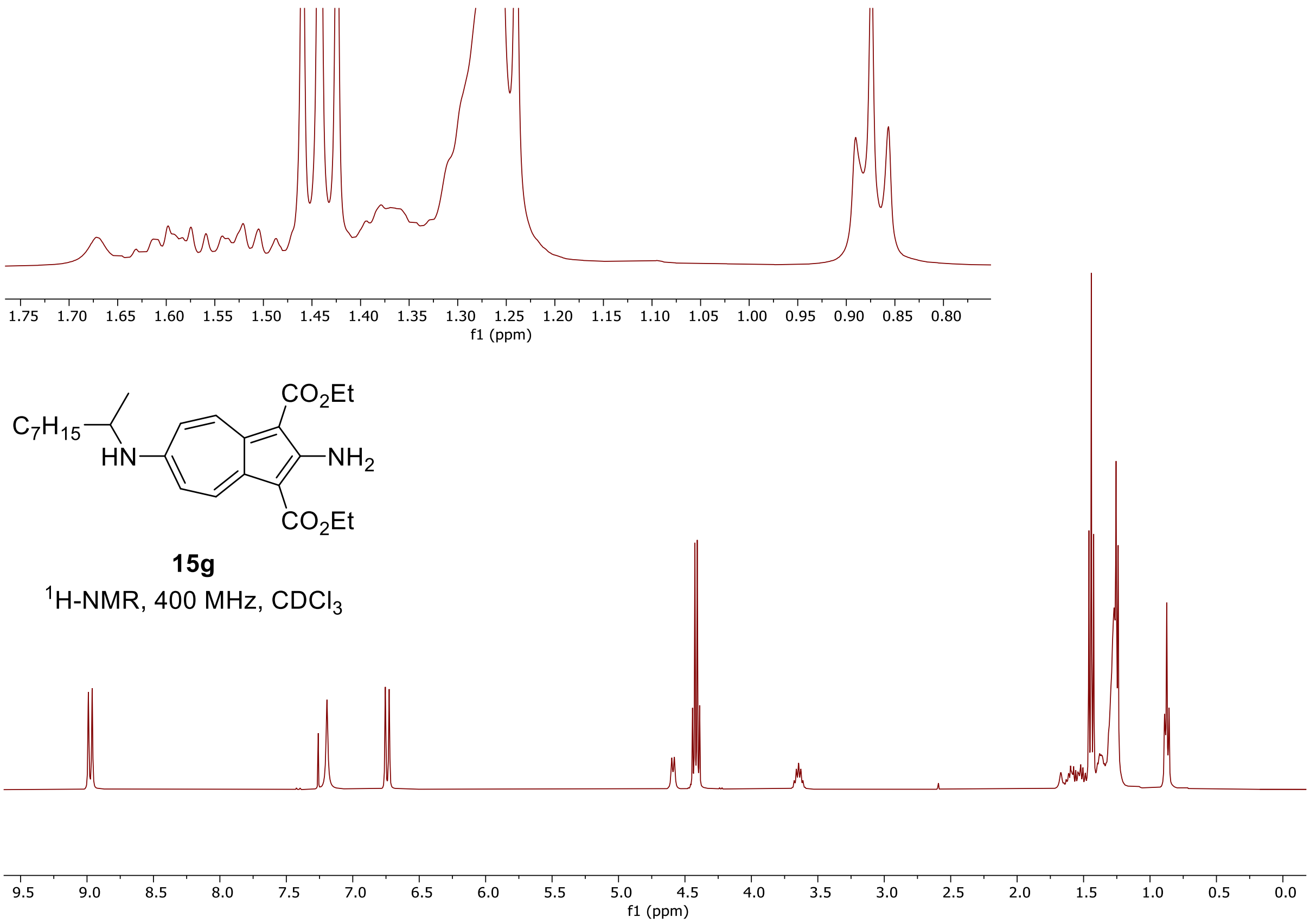

15g

^{1}H-NMR, 400 MHz, $CDCl_3$

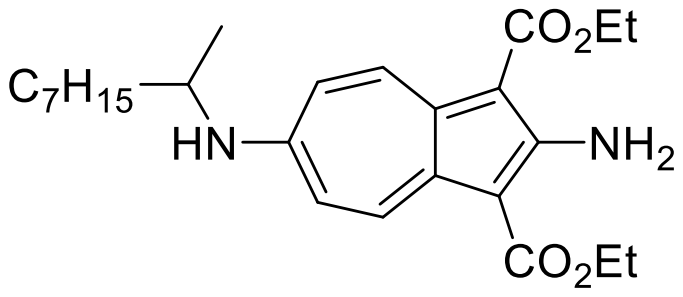

15g

^{13}C-NMR, 101 MHz, $CDCl_3$

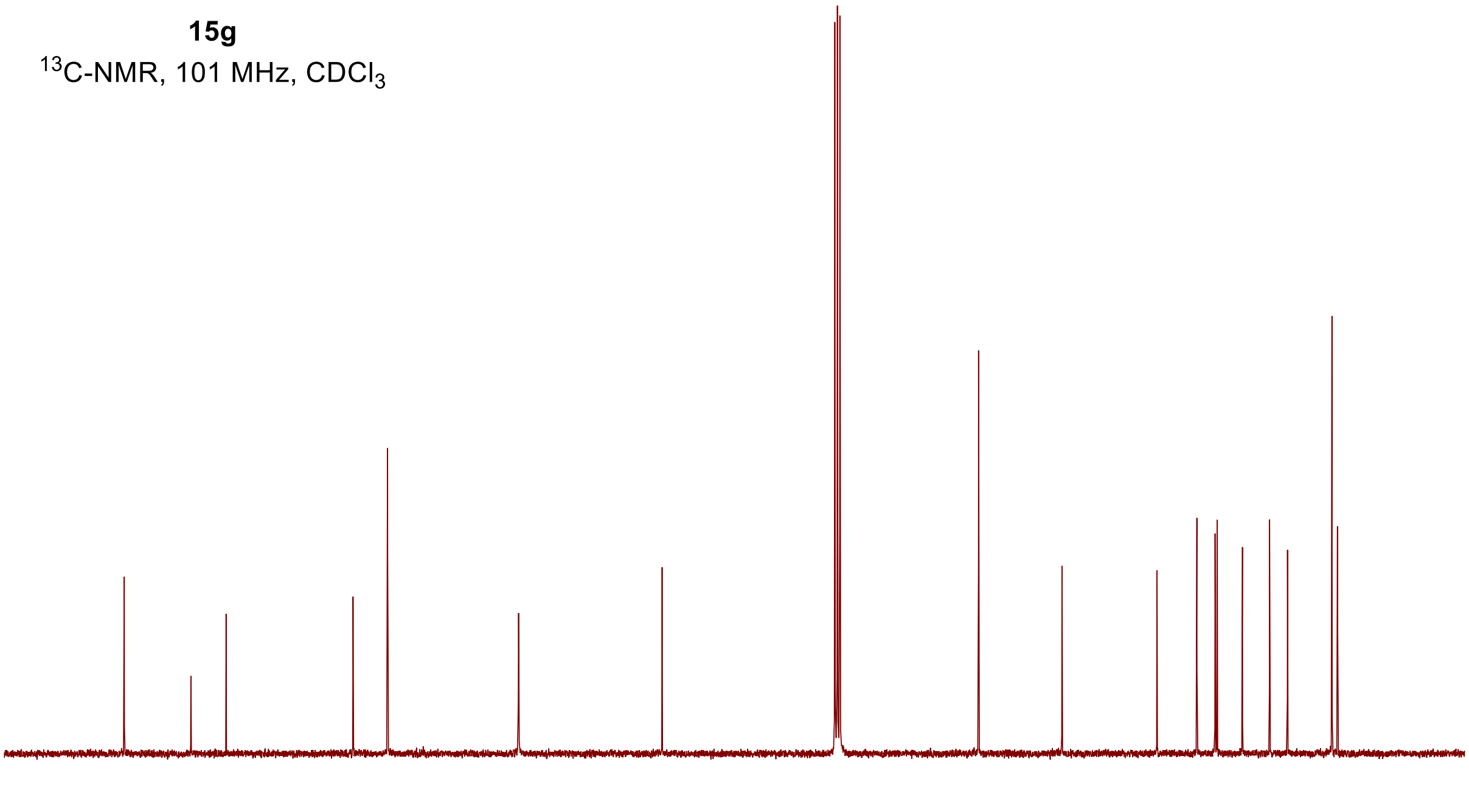

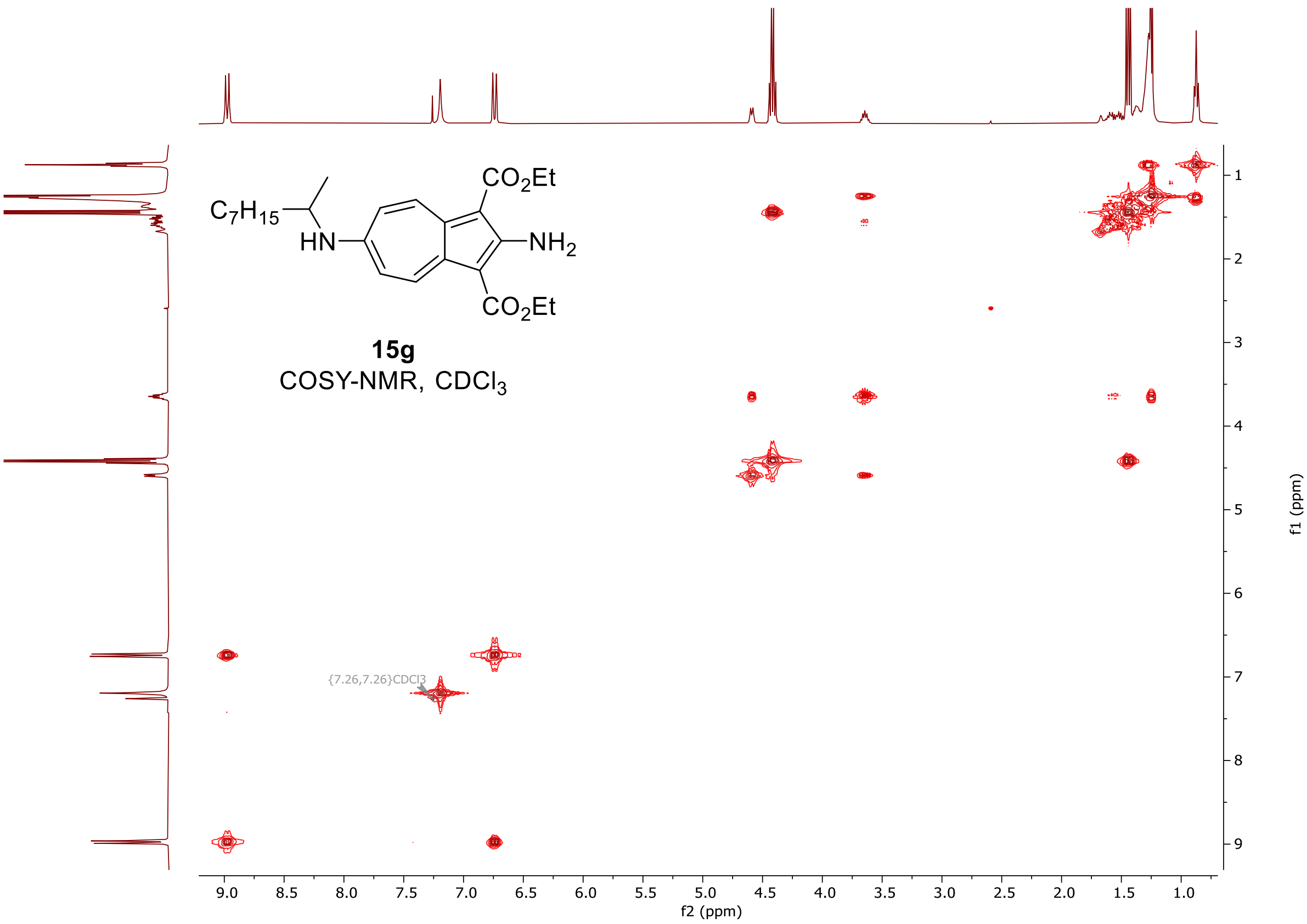

C_7H_{15}
HN
CO_2Et
NH_2
CO_2Et
15g
COSY-NMR, $CDCl_3$
{7.26,7.26}CDCl3
f2 (ppm)
f1 (ppm)

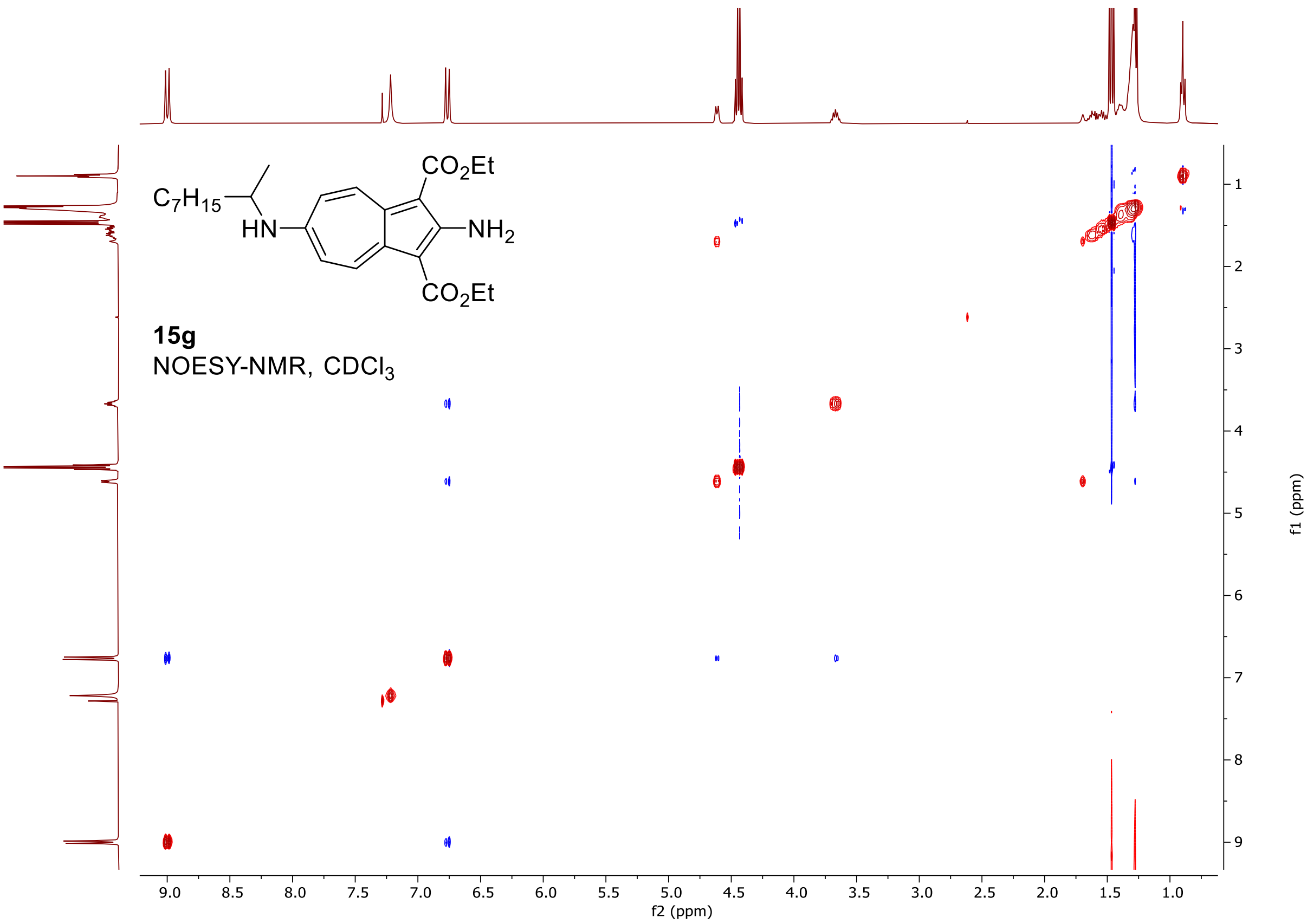

C_7H_{15}
HN
CO_2Et
NH_2
CO_2Et
15g
NOESY-NMR, $CDCl_3$
f2 (ppm)
f1 (ppm)

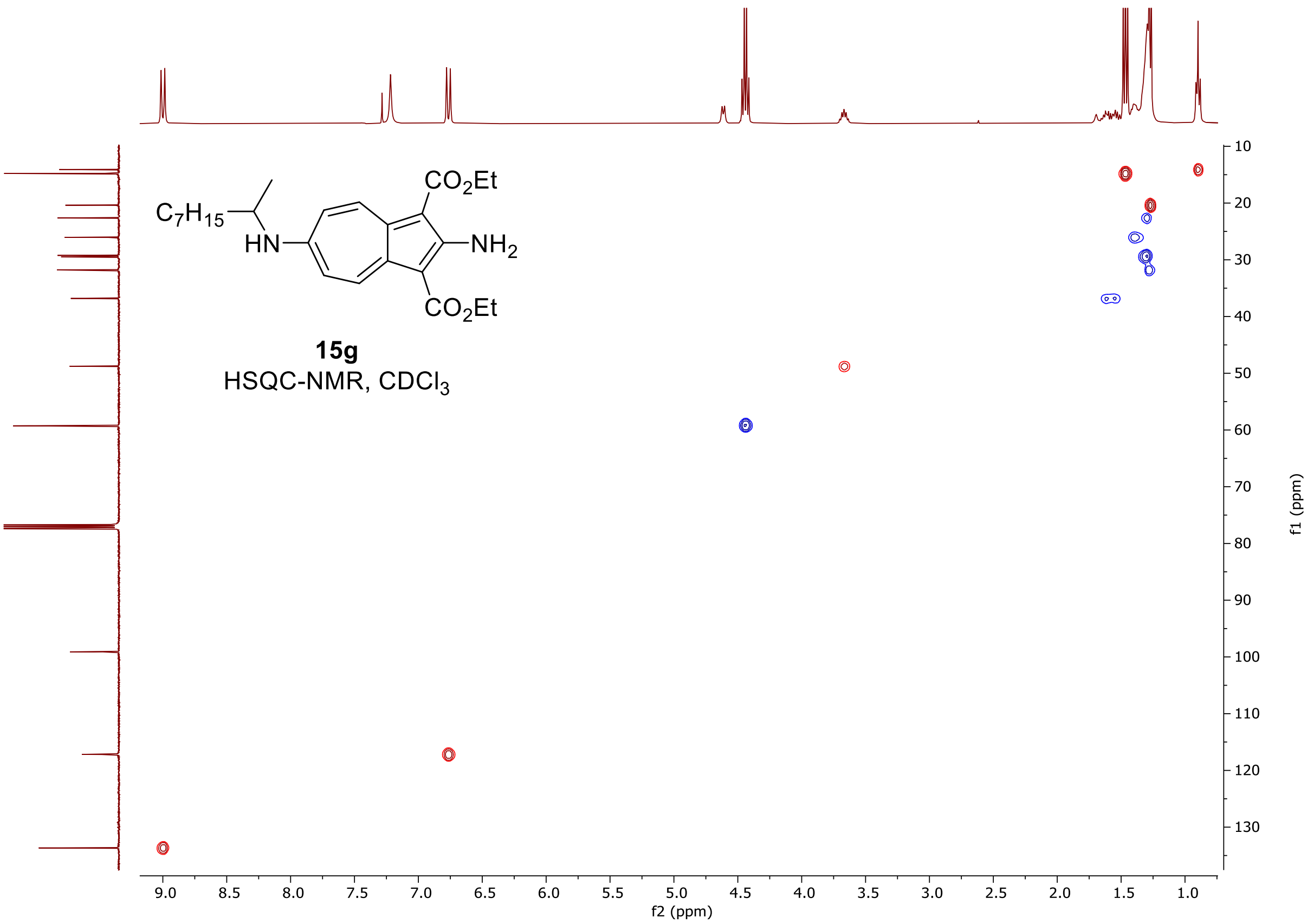

C7H15
HN
CO2Et
NH2
CO2Et
15g
HSQC-NMR, CDCl3
f2 (ppm)
f1 (ppm)

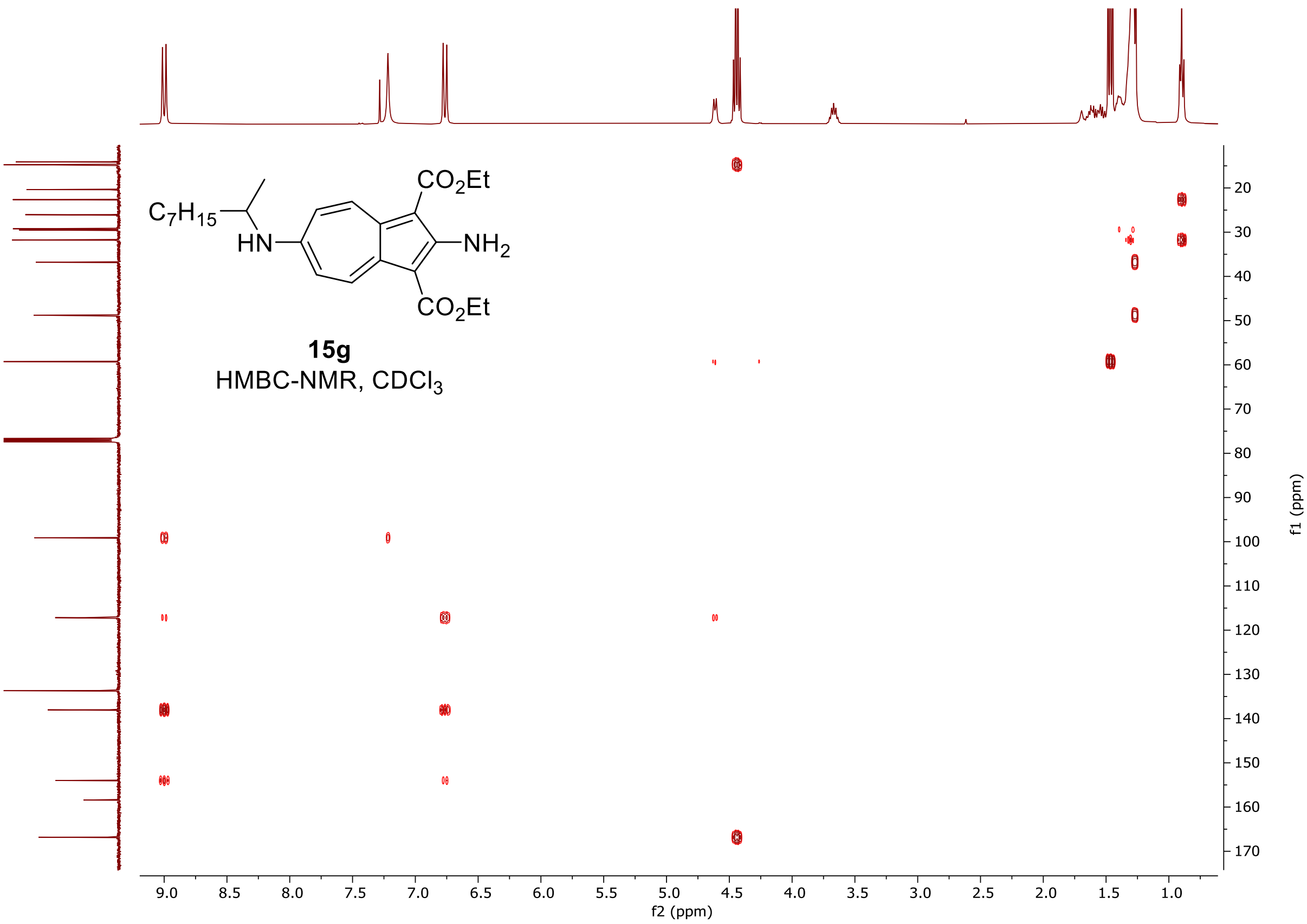

C7H15
HN
CO2Et
NH2
CO2Et
15g
HMBC-NMR, CDCl3
f2 (ppm)
f1 (ppm)

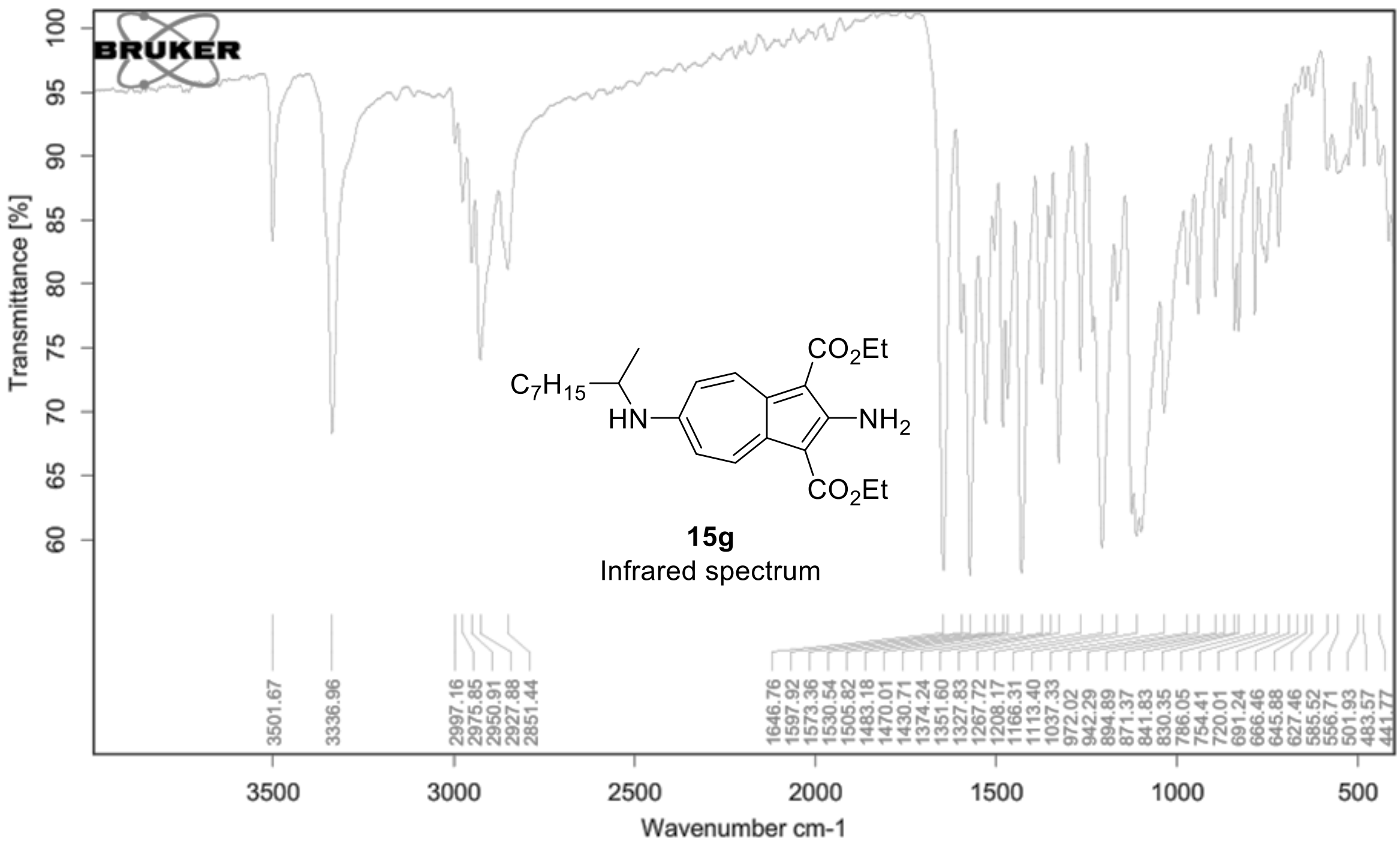

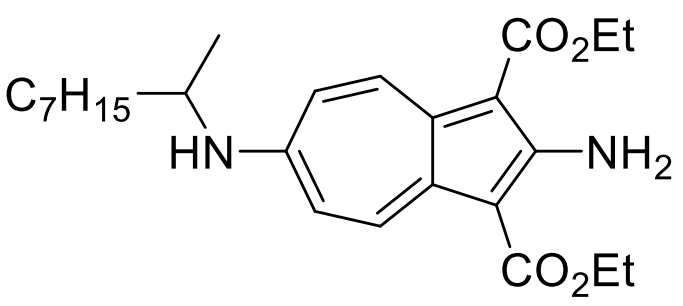

15g
HRMS-ESI$^+$

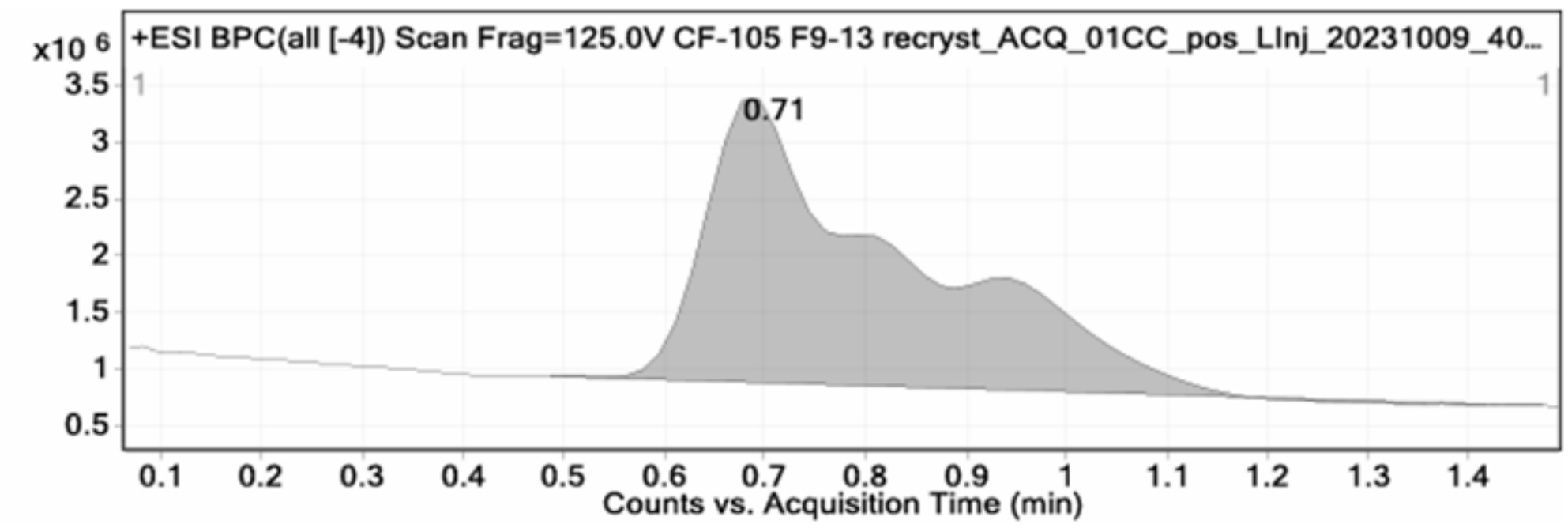

Figure 1: Base peak chromatogram

User Chromatogram Peak List

RT (min)	Area	Area %	Area Sum (%)	Base Peak (m/z)	Width (min)
0.71	34015906	100.00	100.00	429.2775	0.230

Compound Table

Compound Label	RT (min)	Observed mass (m/z)	Neutral observed mass (Da)	Theoretical mass (Da)	Mass error (ppm)	Isotope match score (%)
Cpd 1: C25 H36 N2 O4	0.69	451.2571	428.2673	428.2675	-0.48	99.84

Mass errors of between -5.00 and 5.00 ppm with isotope match scores above 60% are considered confirmation of molecular formulae

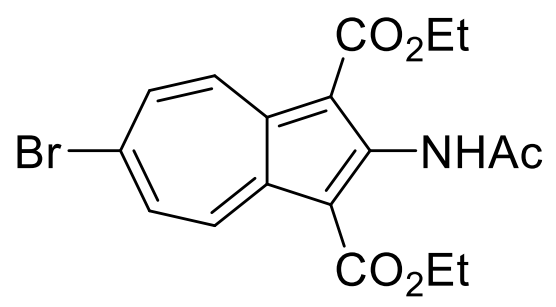

16

^{1}H-NMR, 500 MHz, $CDCl_3$

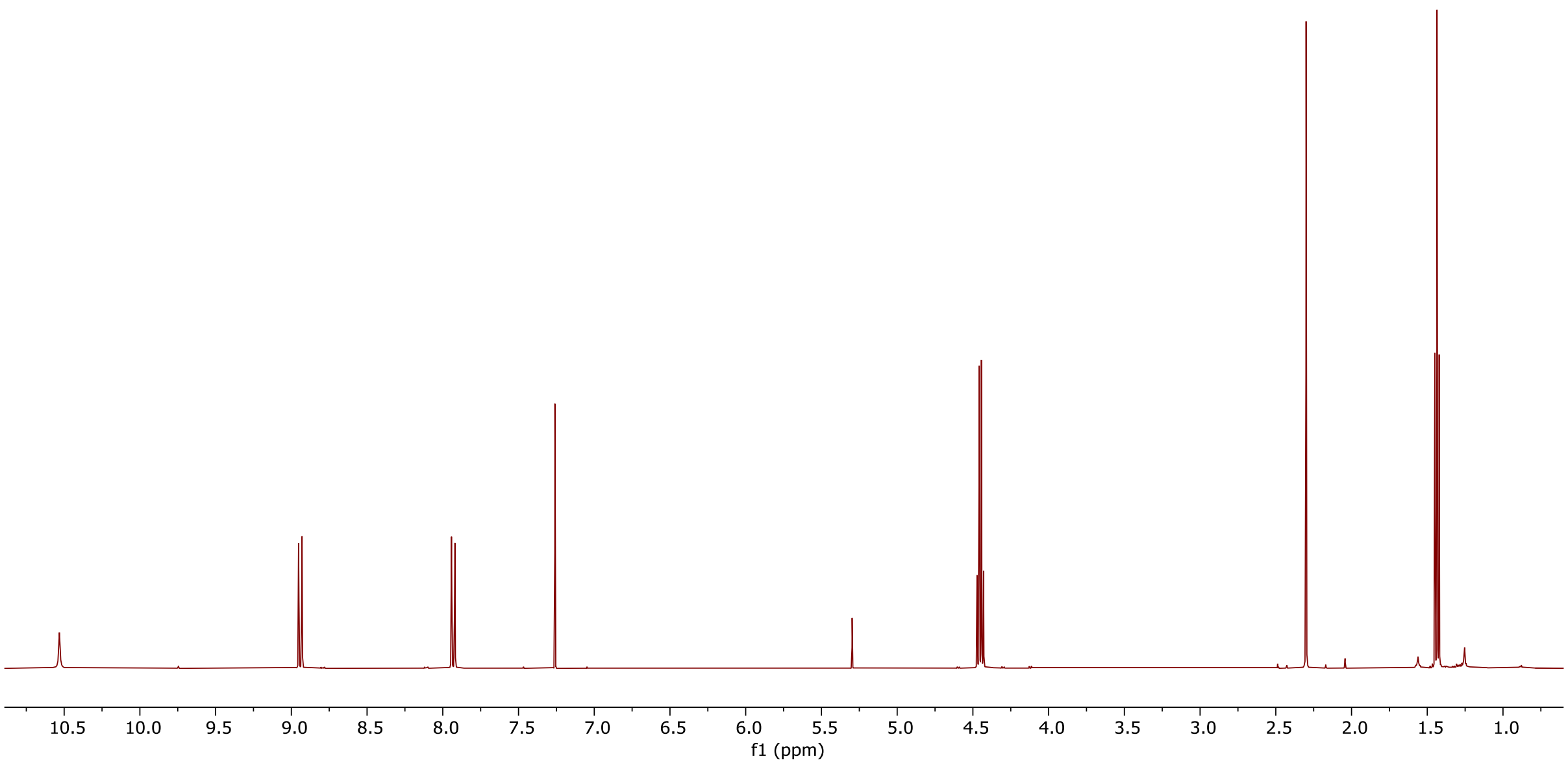

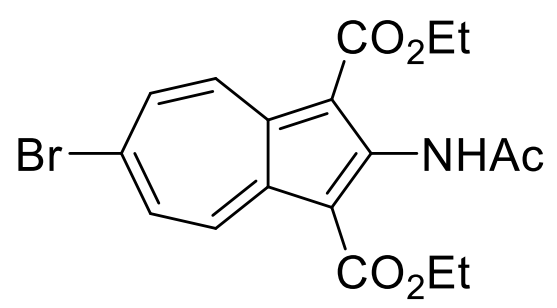

16

^{13}C-NMR, 126 MHz, $CDCl_3$

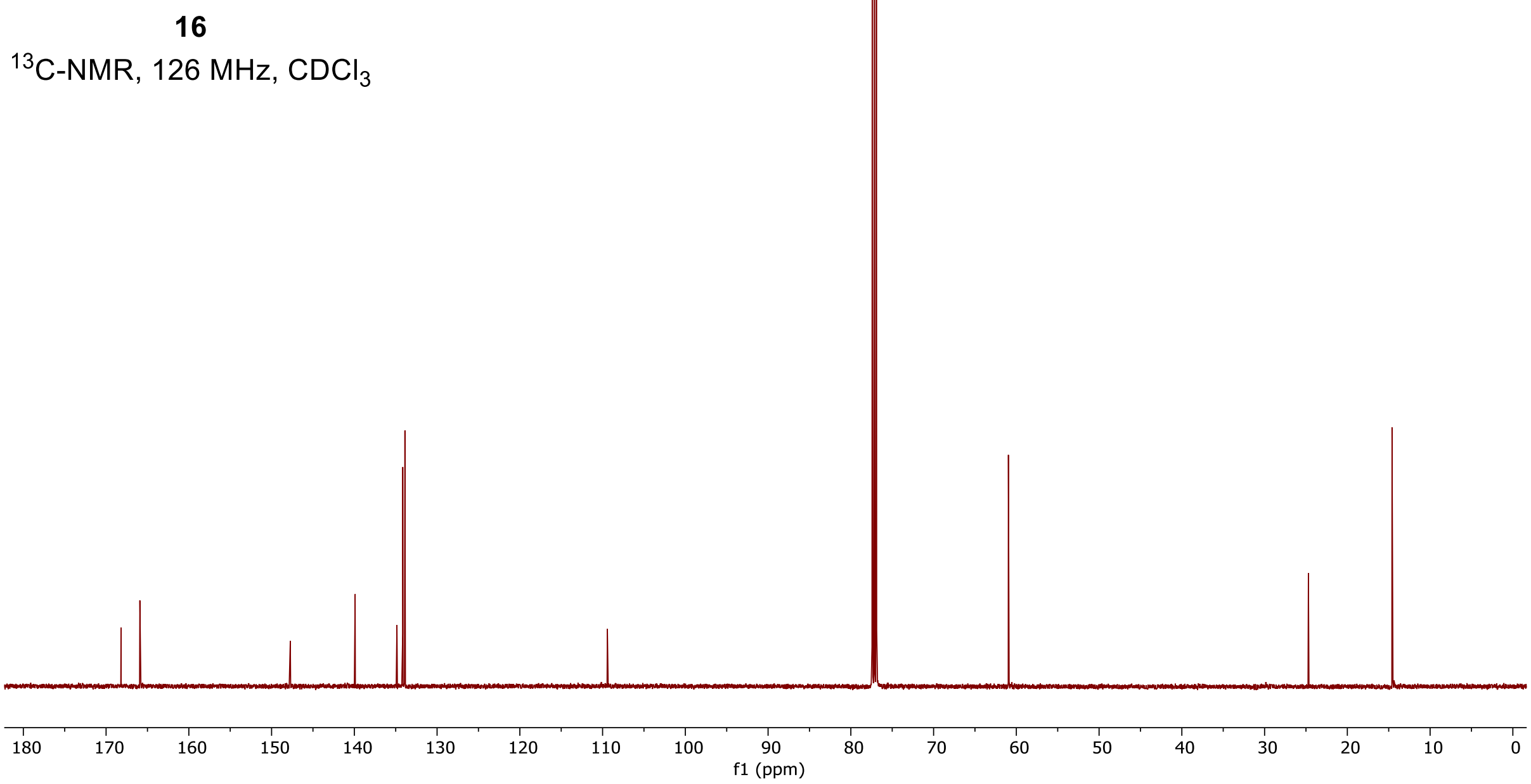

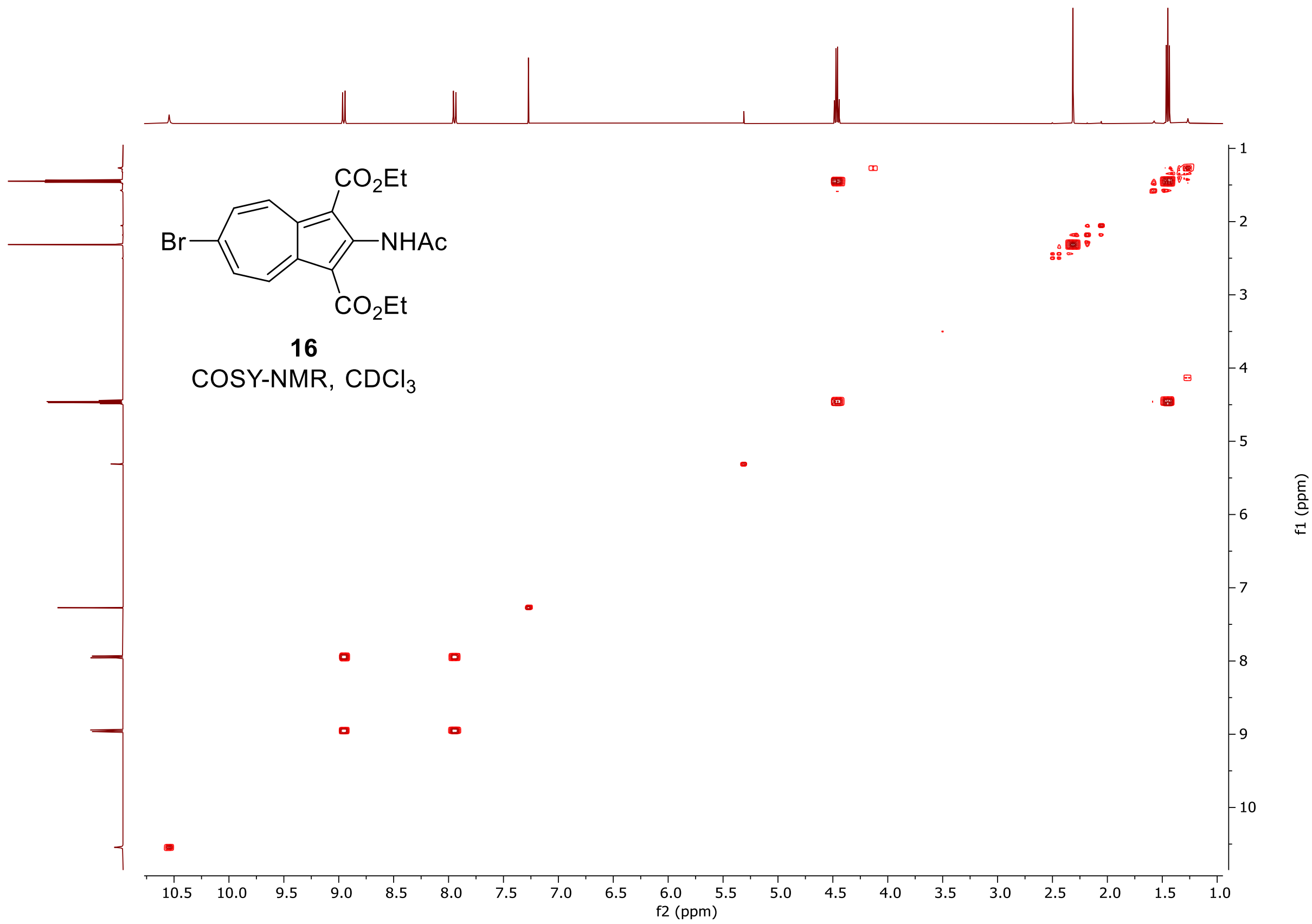

CO2Et
Br
NHAc
CO2Et
16
COSY-NMR, CDCl3
f2 (ppm)
f1 (ppm)

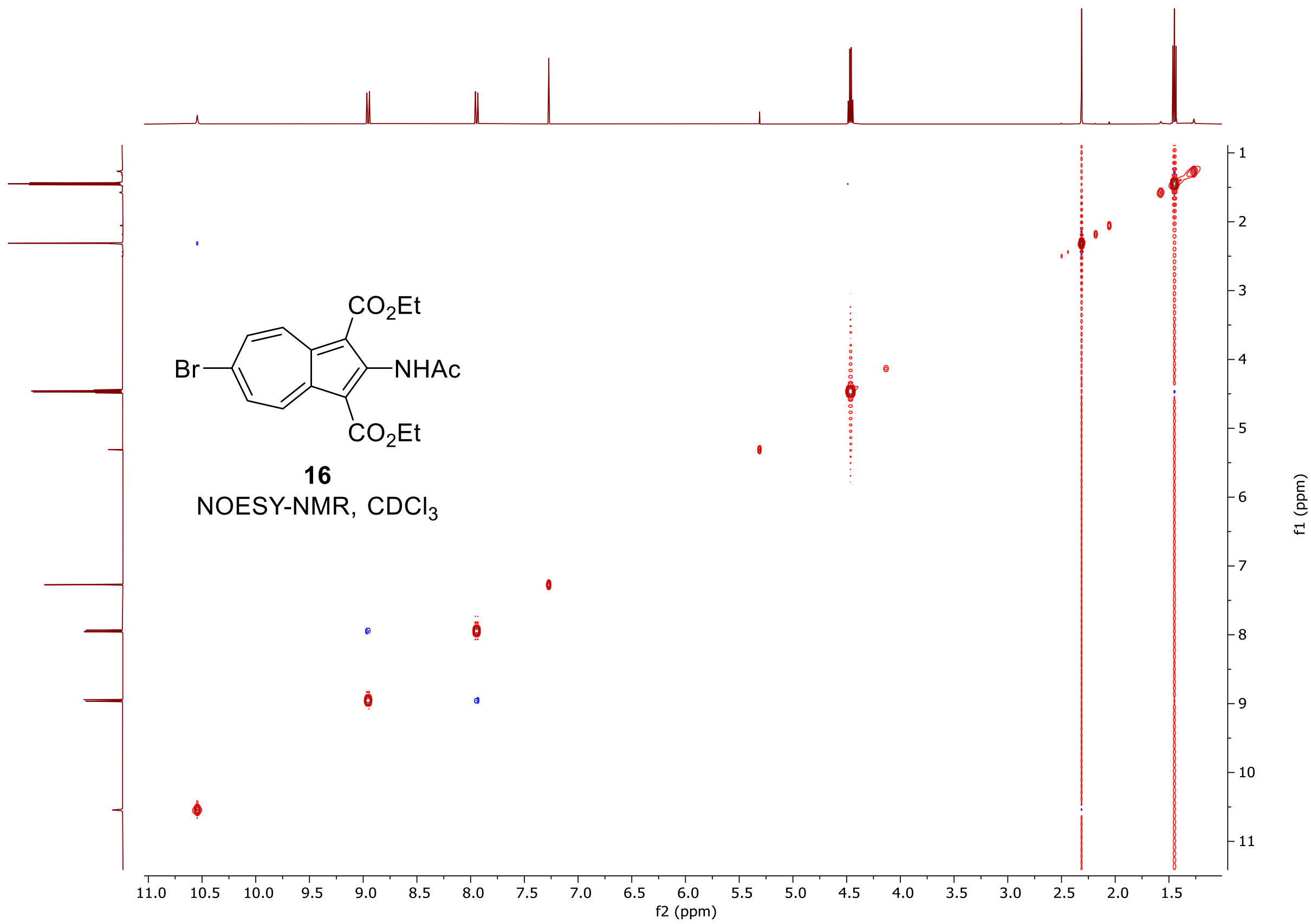

CO2Et
Br
NHAc
CO2Et
16
NOESY-NMR, CDCl3
f2 (ppm)
f1 (ppm)

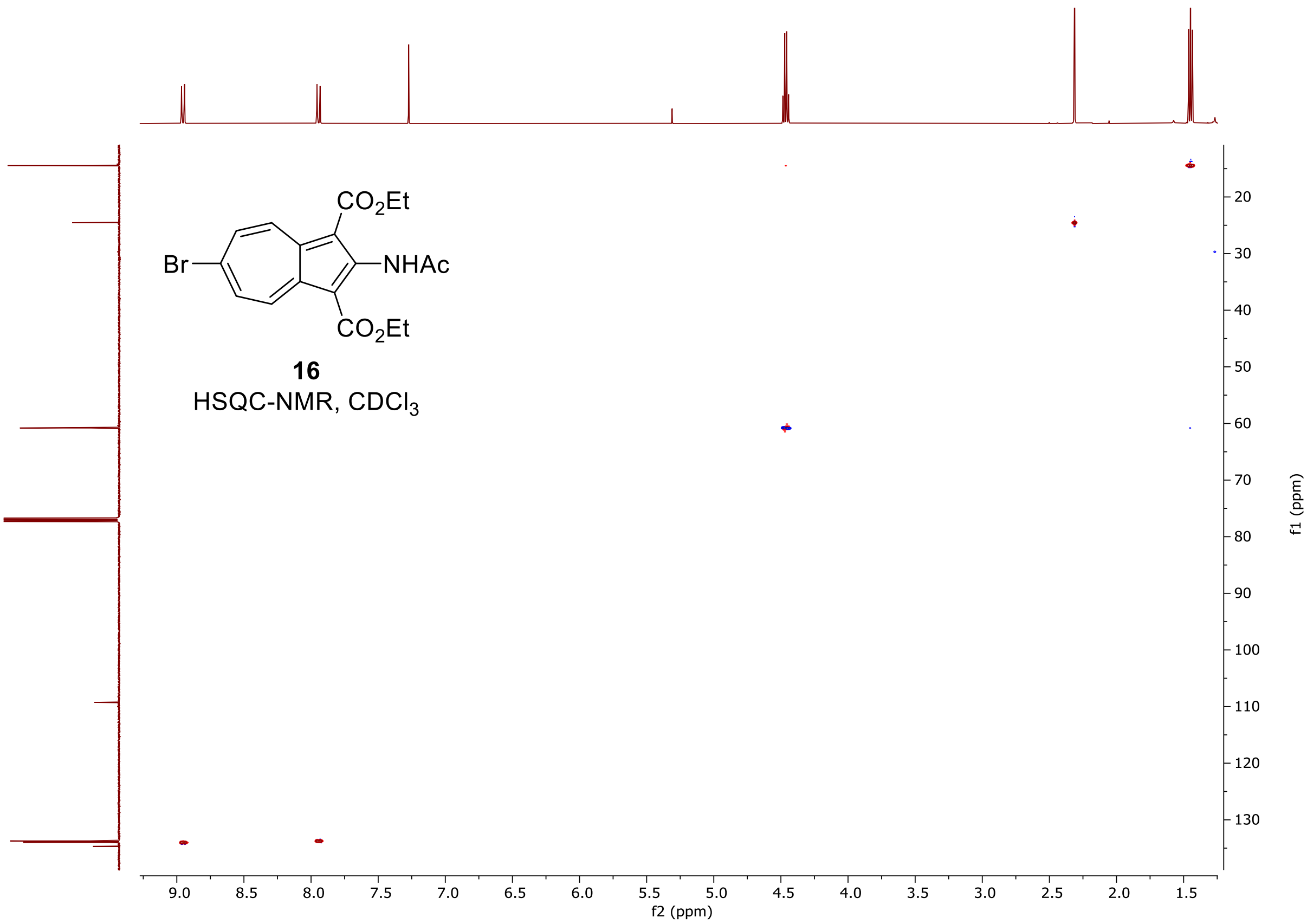
CO2Et
Br
NHAc
CO2Et
16
HSQC-NMR, CDCl3
f1 (ppm)
f2 (ppm)

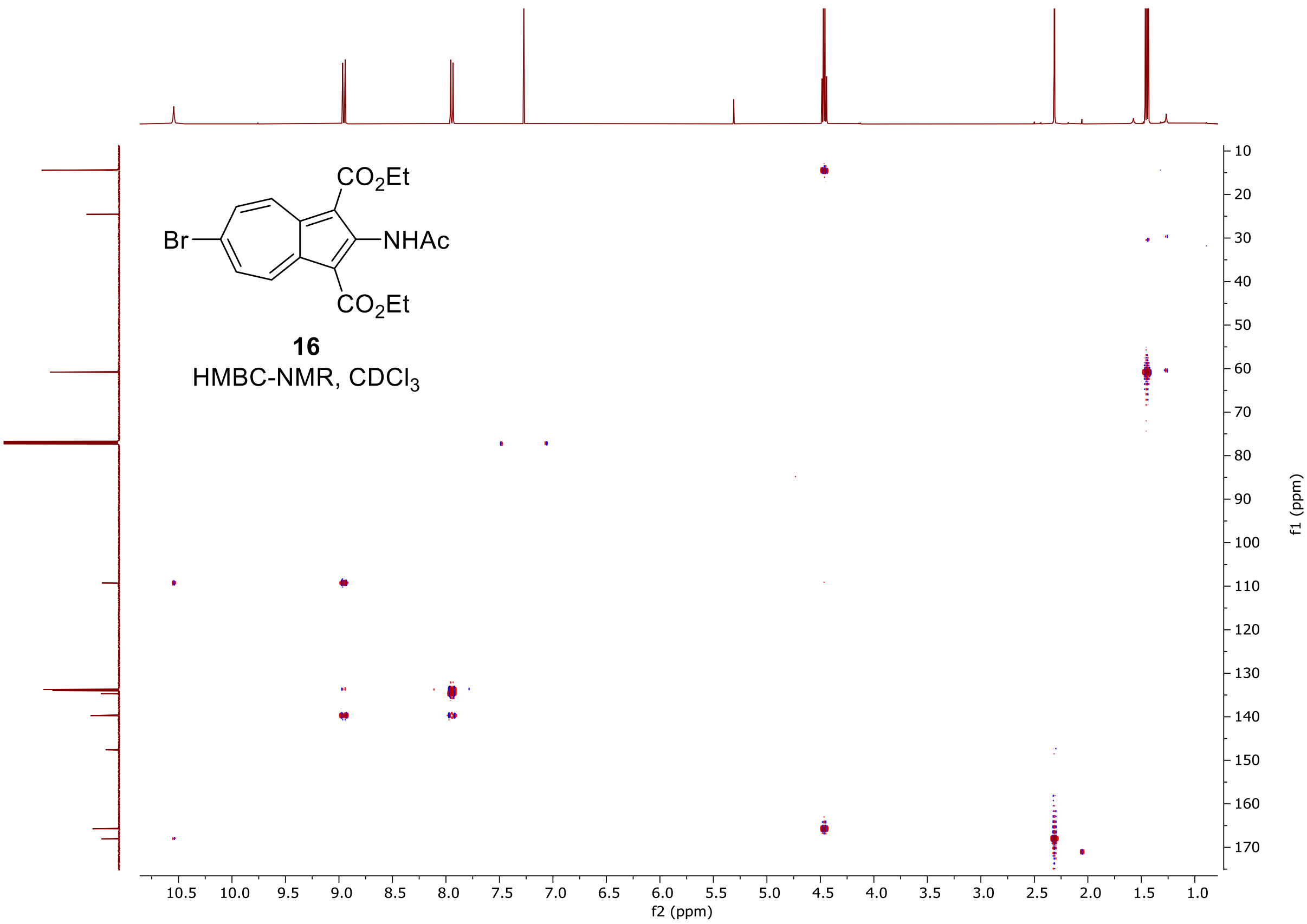
CO2Et
Br
NHAc
CO2Et
16
HMBC-NMR, CDCl3
f1 (ppm)
f2 (ppm)

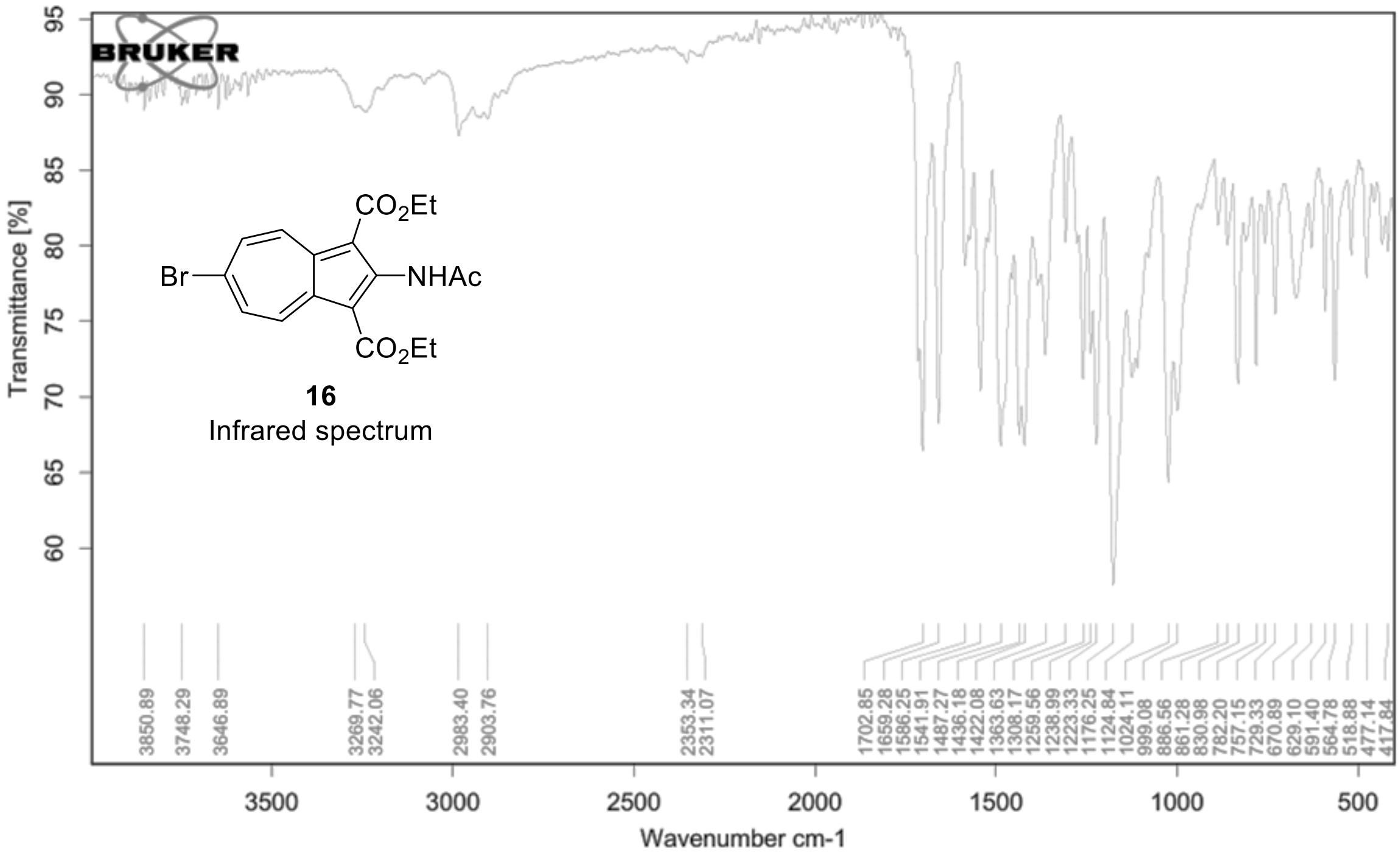

C:\Users\Lab0.25\Documents\IR\220627_1.203 sample sample form 20/09/2023

Page 1 of 1

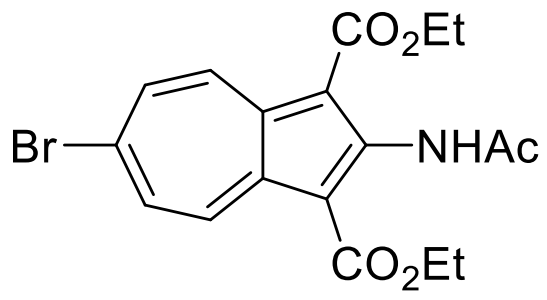

16, HRMS-ESI$^+$

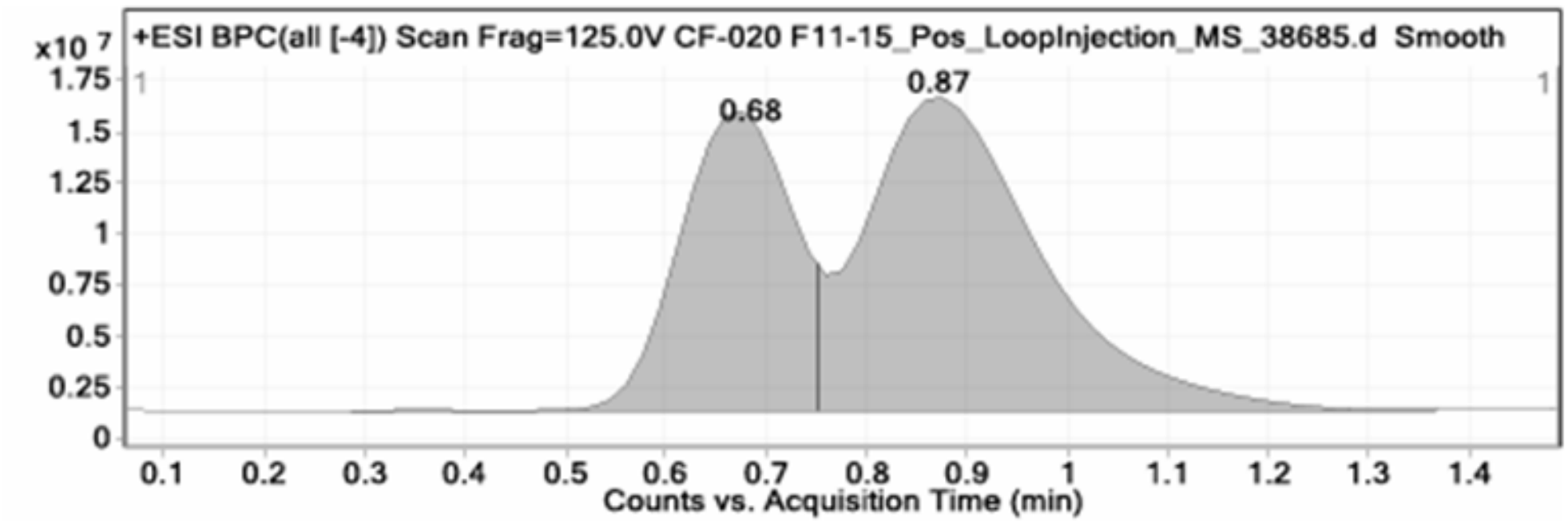

Figure 1: Base peak chromatogram

User Chromatogram Peak List

RT (min)	Area	Area %	Area Sum (%)	Base Peak (m/z)	Width (min)
0.68	112725116	59.60	37.34	839.0632	0.180
0.87	189148463	100.00	62.66	408.0449	0.210

Compound Table

Compound Label	RT (min)	Observed mass (m/z)	Neutral observed mass (Da)	Theoretical mass (Da)	Mass error (ppm)	Isotope match score (%)
Cpd 1: C18 H18 Br N O5	0.88	408.0456	407.0383	407.0368	3.55	98.84

Mass errors of between -5.00 and 5.00 ppm with isotope match scores above 60% are considered confirmation of molecular formulae

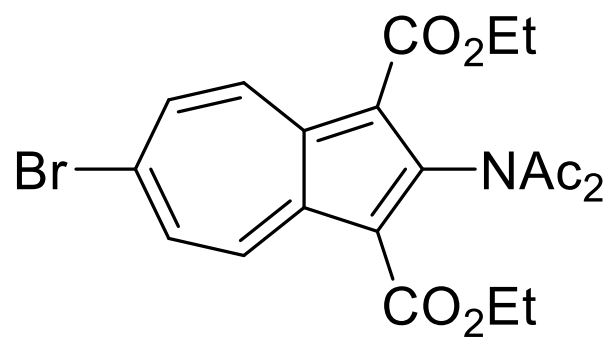

17

^{1}H-NMR, 500 MHz, $CDCl_3$

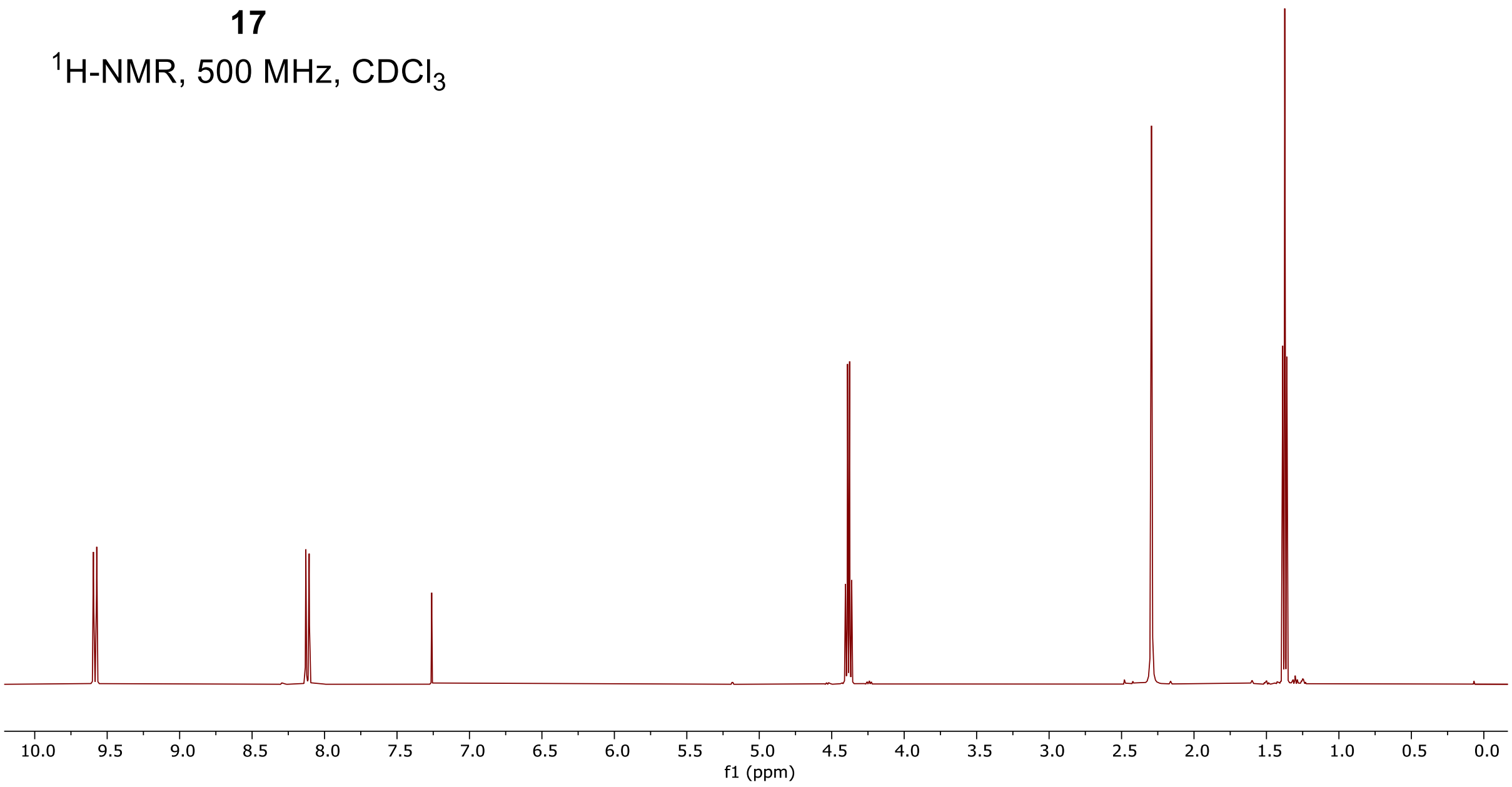

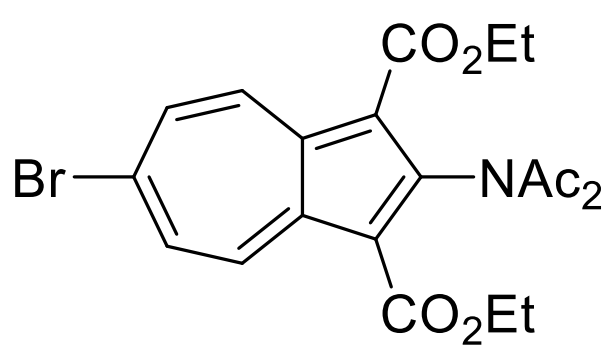

17

^{13}C-NMR, 126 MHz, $CDCl_3$

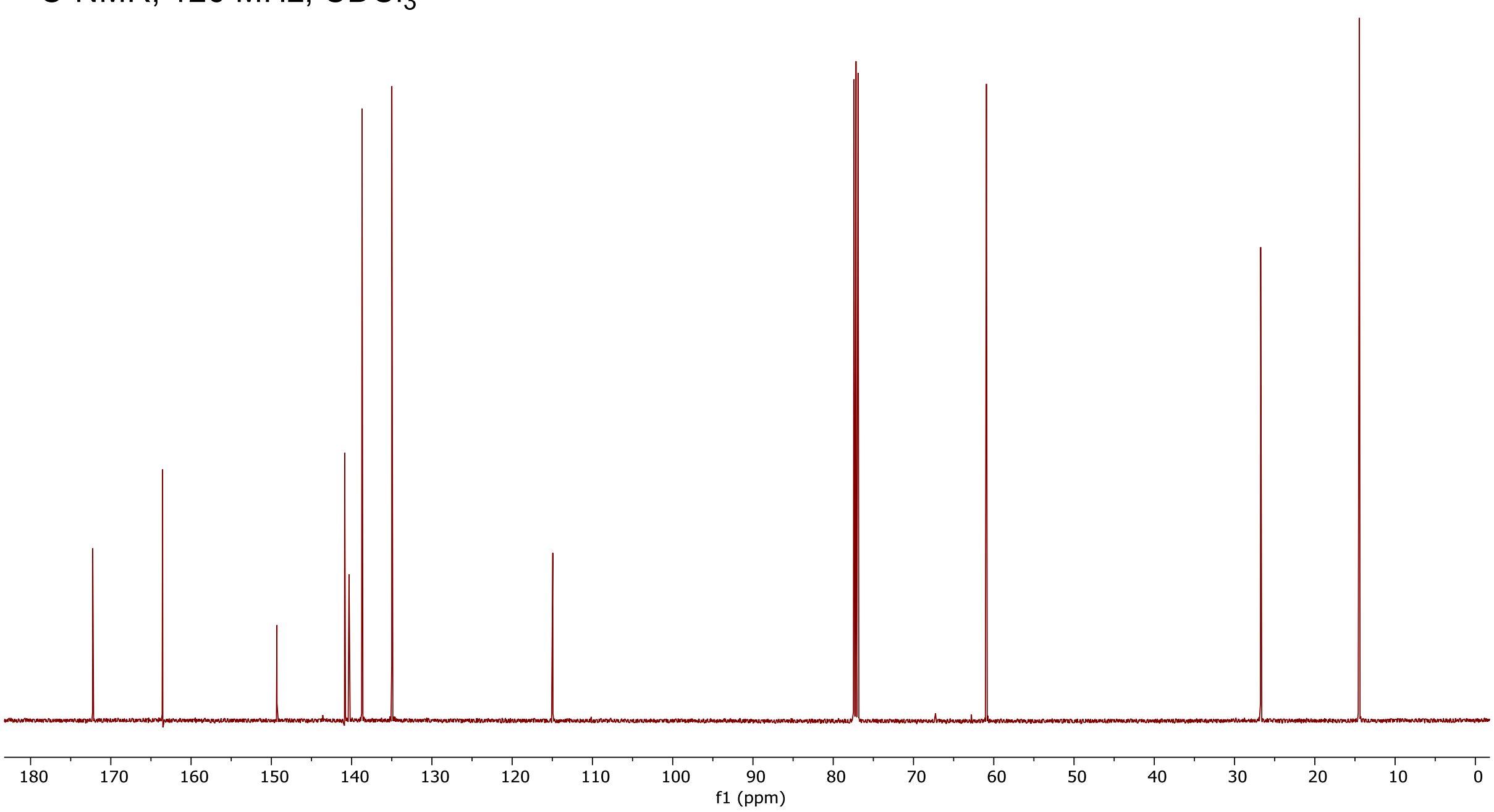

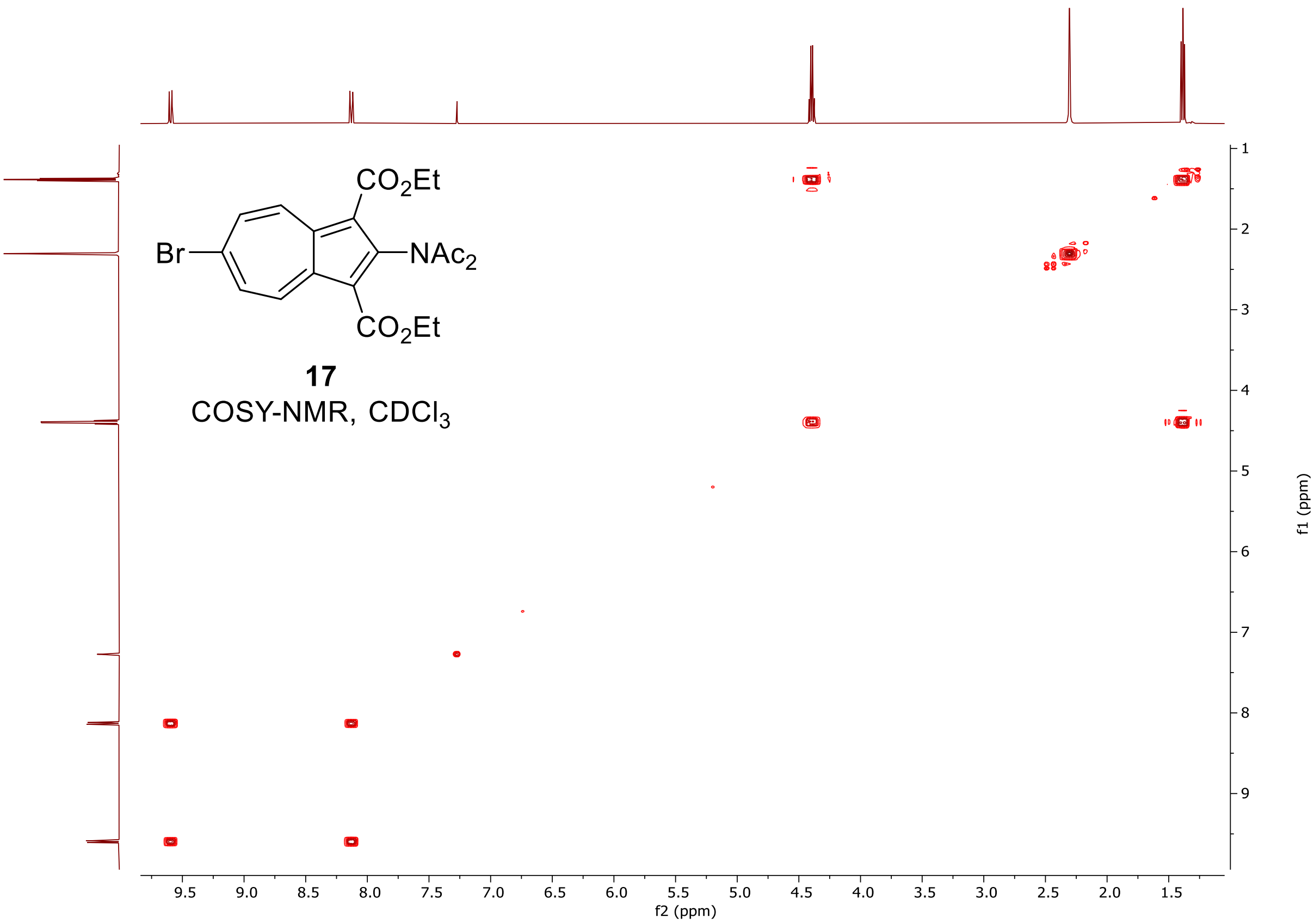
CO2Et
Br
NAc2
CO2Et
17
COSY-NMR, CDCl3
f2 (ppm)
f1 (ppm)

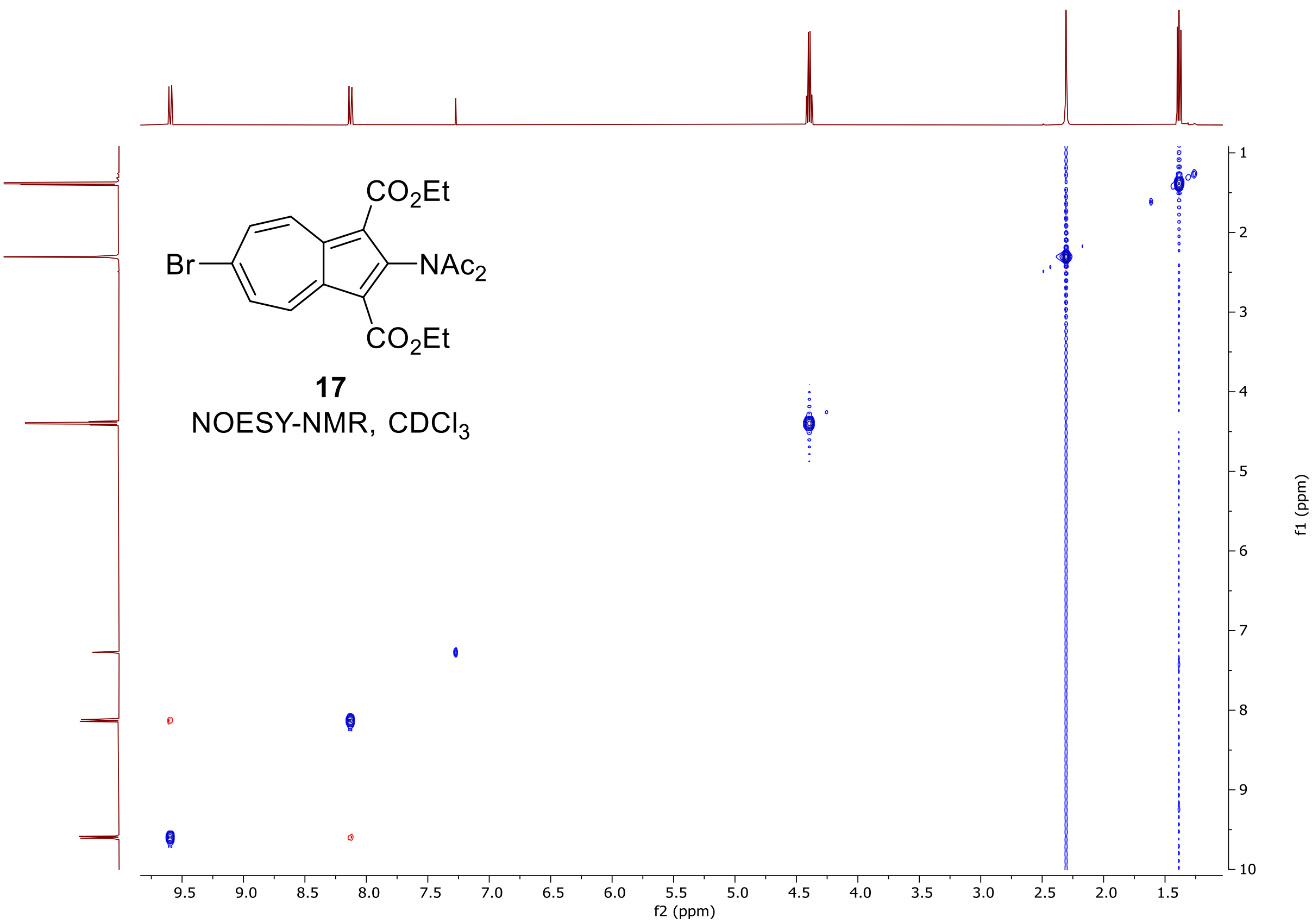
CO2Et
Br
NAc2
CO2Et
17
NOESY-NMR, CDCl3
f2 (ppm)
f1 (ppm)

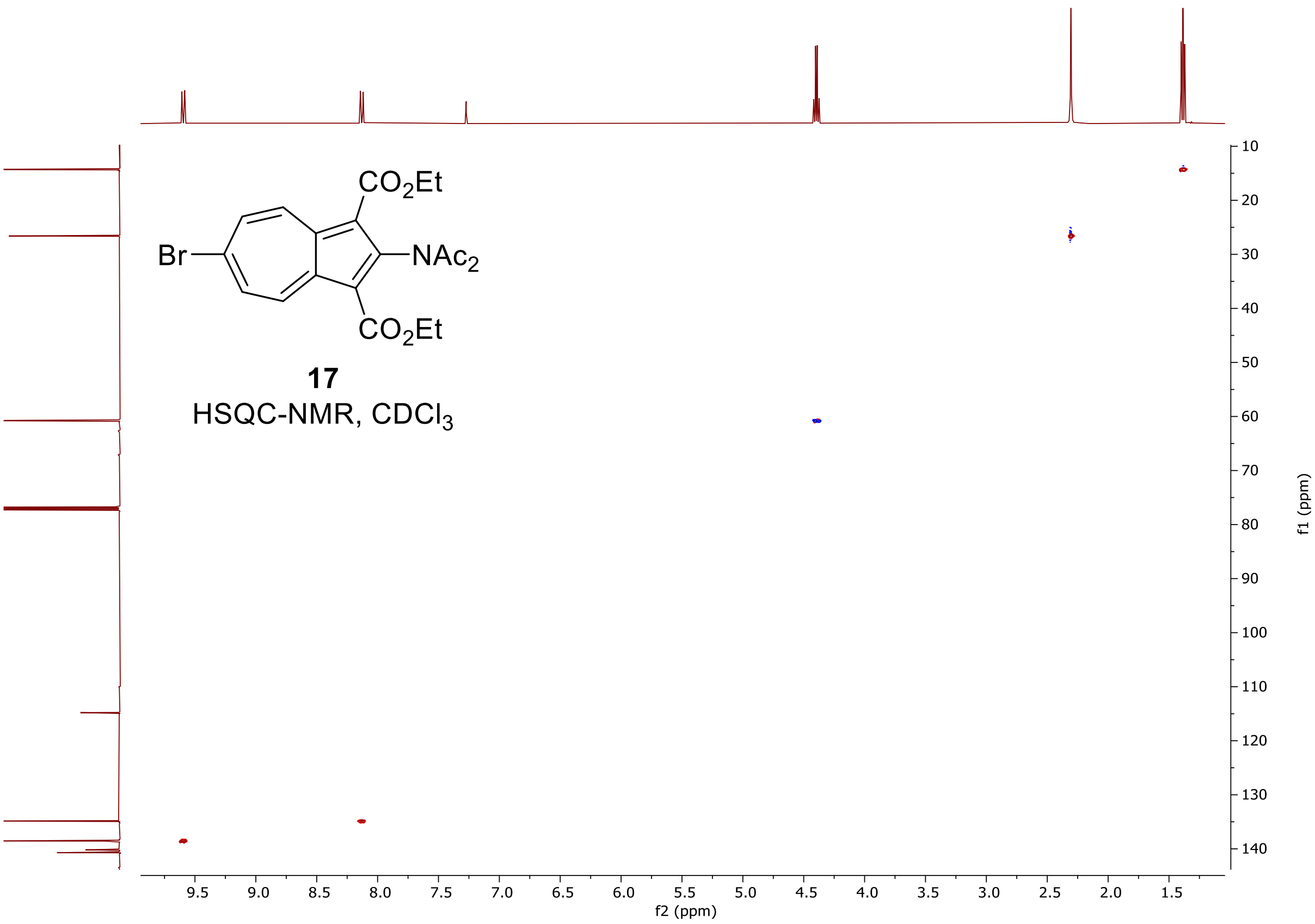
CO2Et
Br
NAc2
CO2Et
17
HSQC-NMR, CDCl3
f2 (ppm)
f1 (ppm)

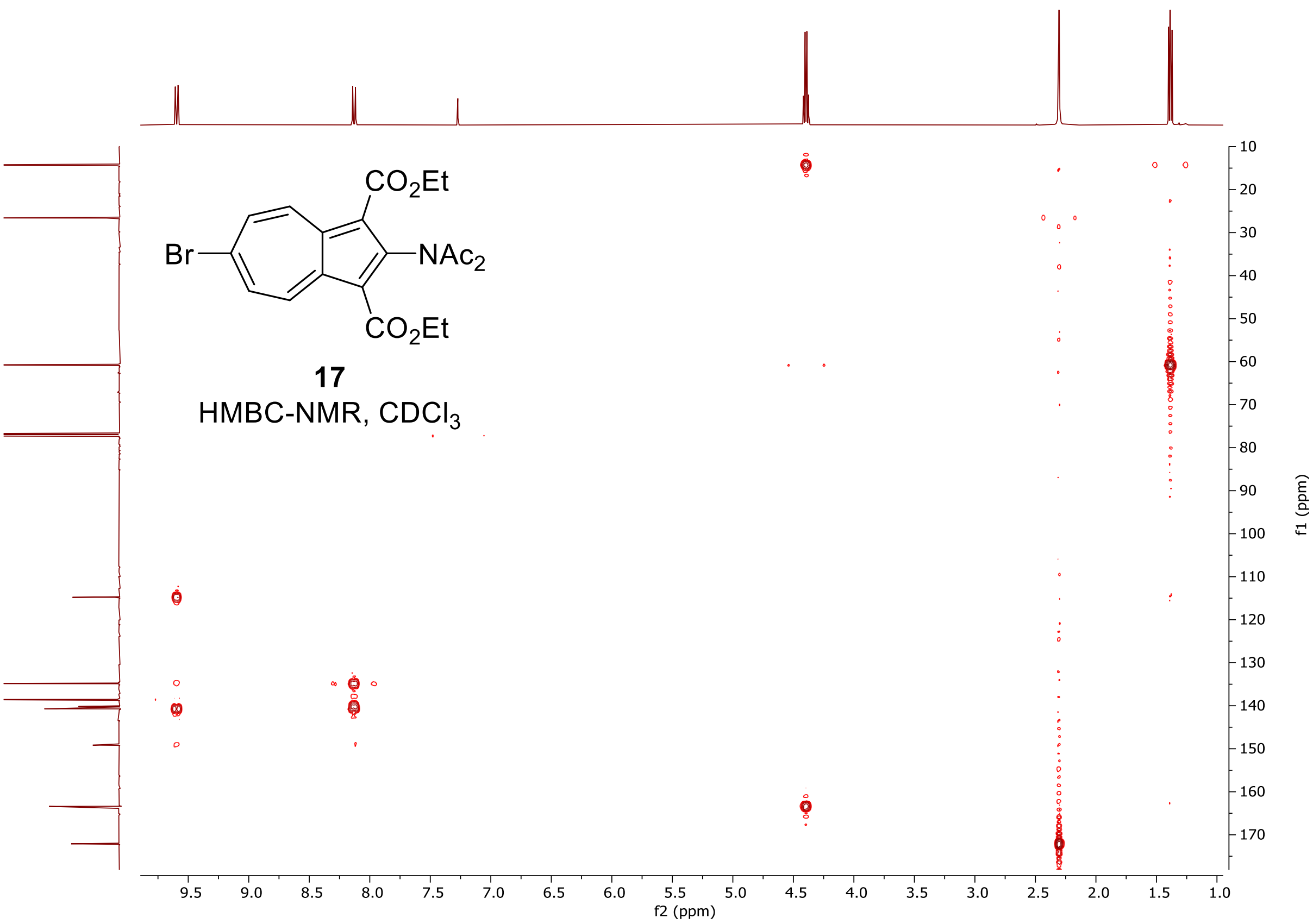
CO2Et
Br
NAc2
CO2Et
17
HMBC-NMR, CDCl3
f2 (ppm)
f1 (ppm)

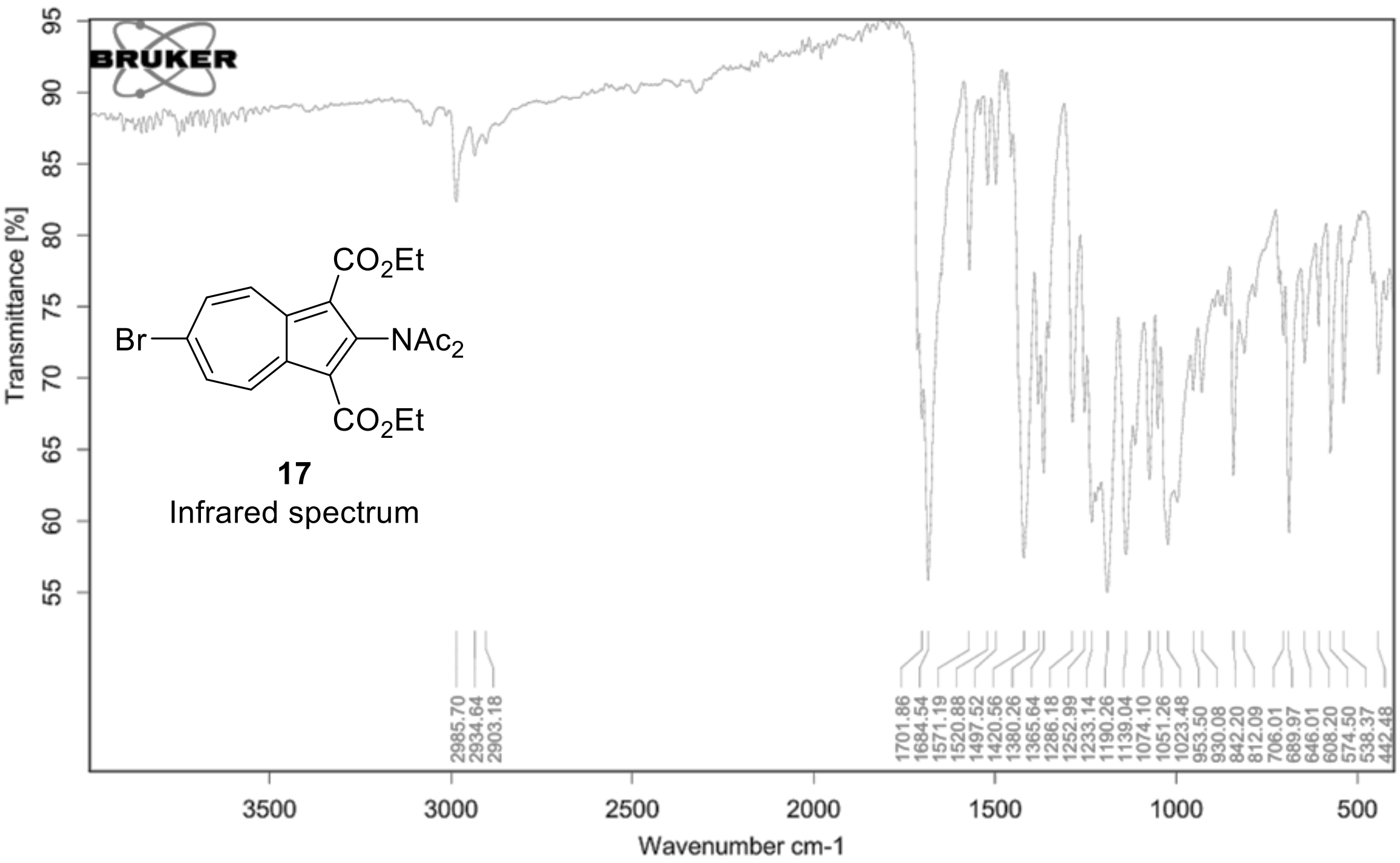

C:\Users\Lab0.25\Documents\IR\220627_1.201	sample	sample form	20/09/2023

Page 1 of 1

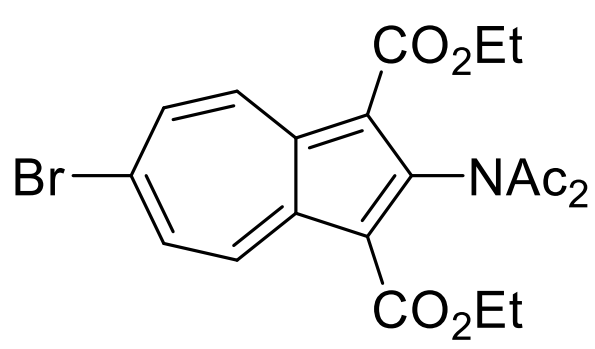

17

HRMS-ESI$^+$

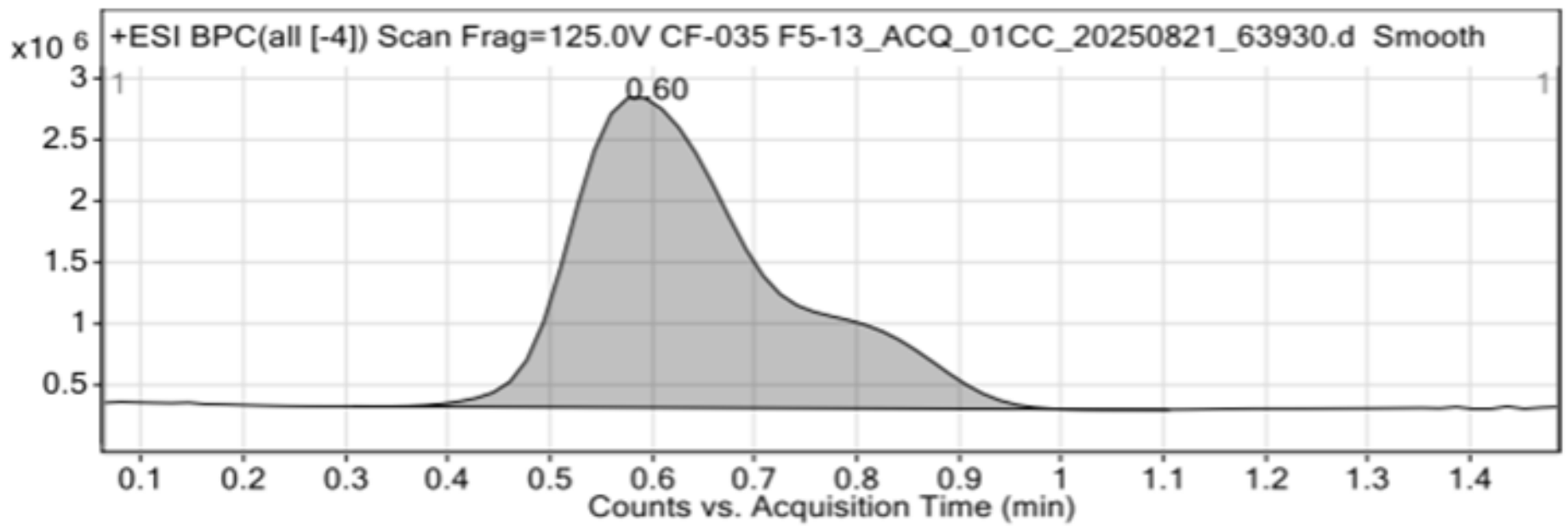

Figure 1: Base peak chromatogram

User Chromatogram Peak List

RT (min)	Area	Area %	Area Sum (%)	Base Peak (m/z)	Width (min)
0.60	33717847	100.00	100.00	474.0369	0.230

Compound Table

Compound Label	RT (min)	Observed mass (m/z)	Neutral observed mass (Da)	Theoretical mass (Da)	Mass error (ppm)	Isotope match score (%)
Cpd 1: C20 H20 Br N O6	0.60	450.0543	449.0470	449.0474	-0.98	99.03

Mass errors of between -5.00 and 5.00 ppm with isotope match scores above 60% are considered confirmation of molecular formulae

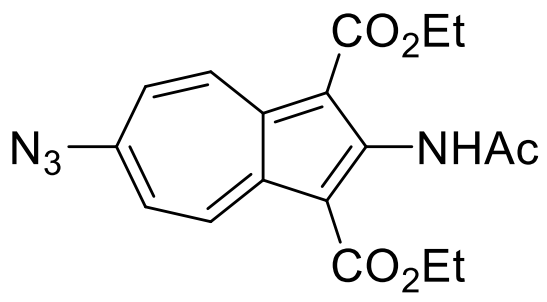

18

^{1}H-NMR, 400 MHz, $CDCl_3$

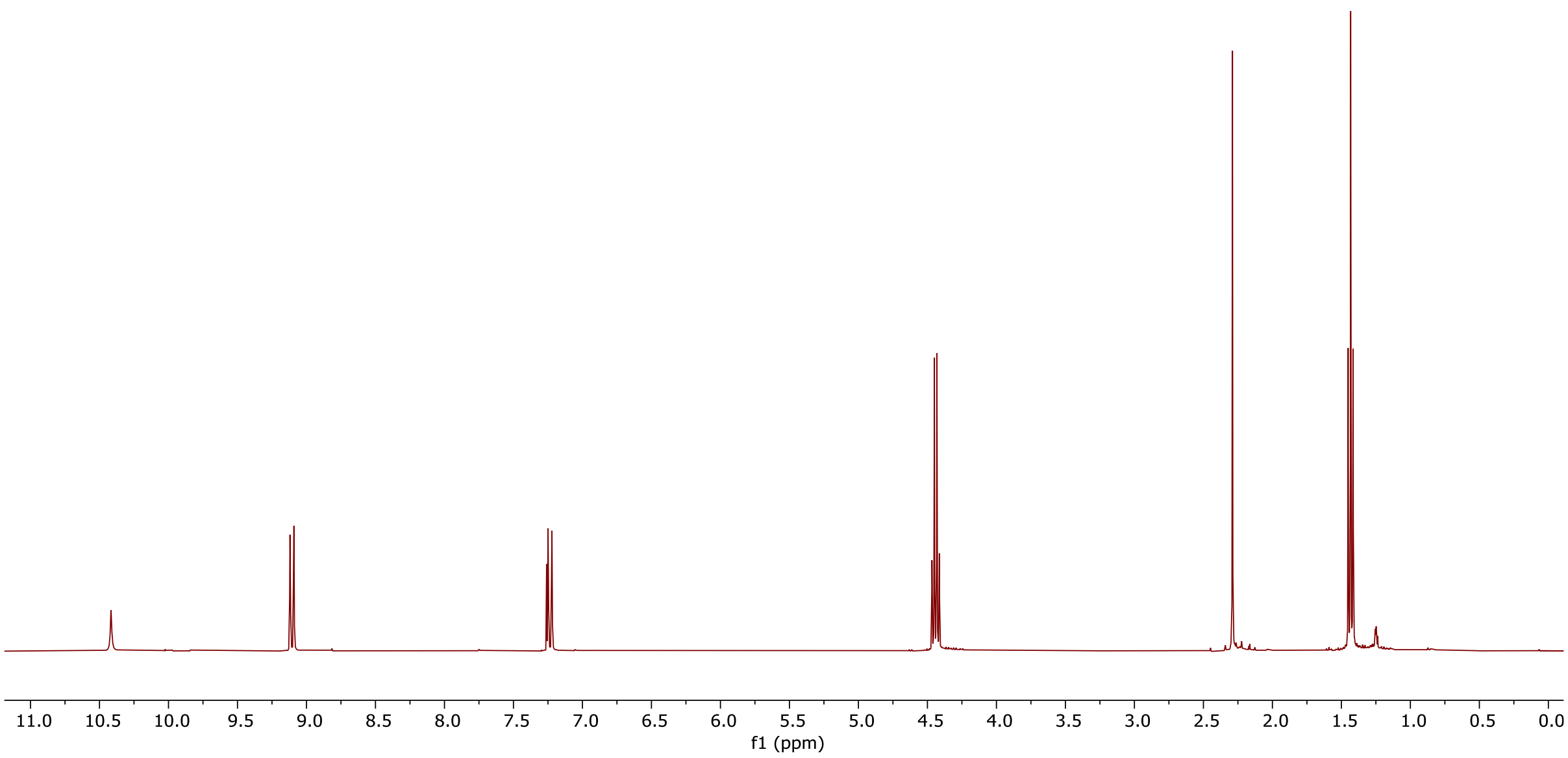

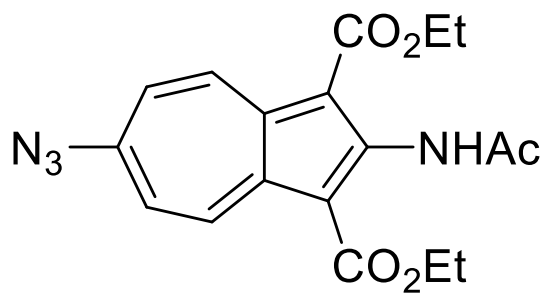

18

^{13}C-NMR, 101 MHz, $CDCl_3$

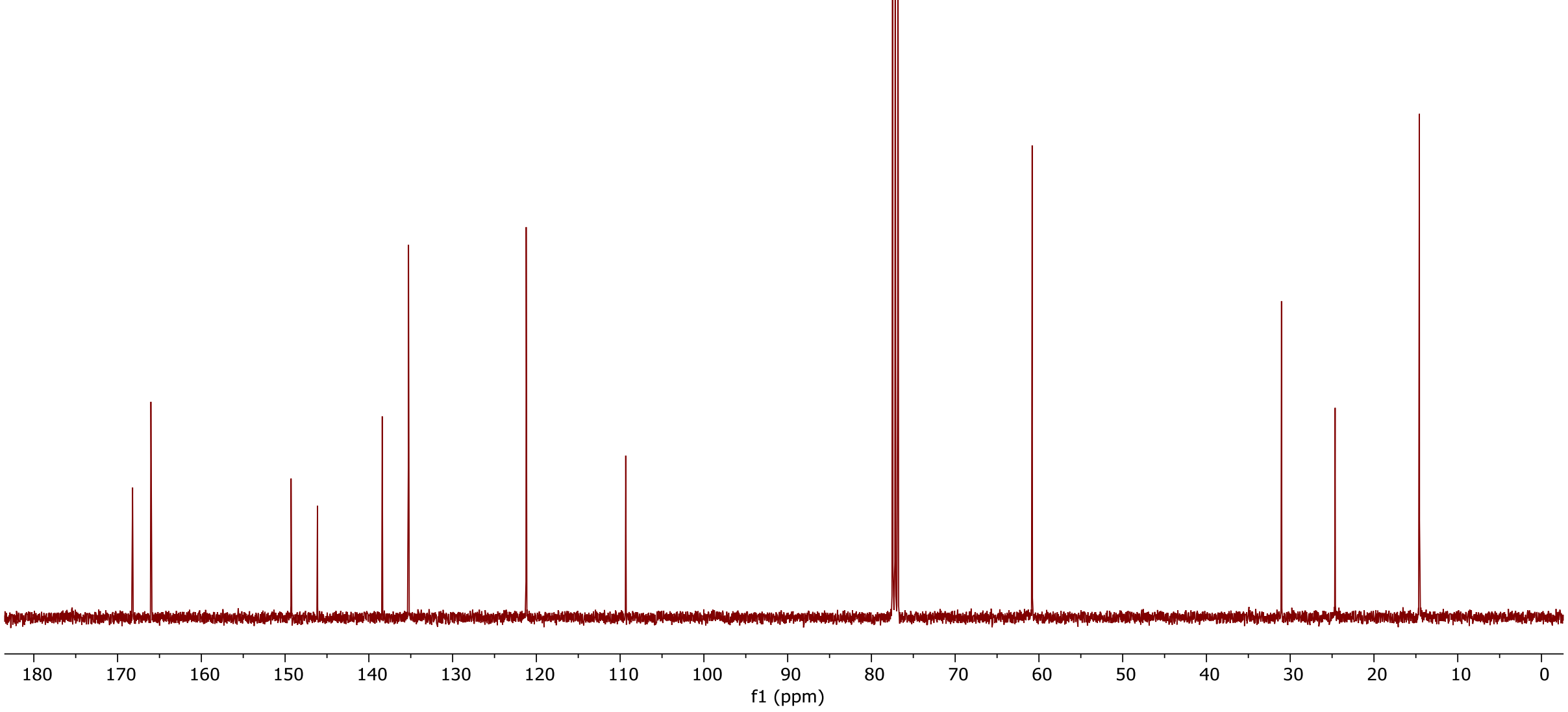

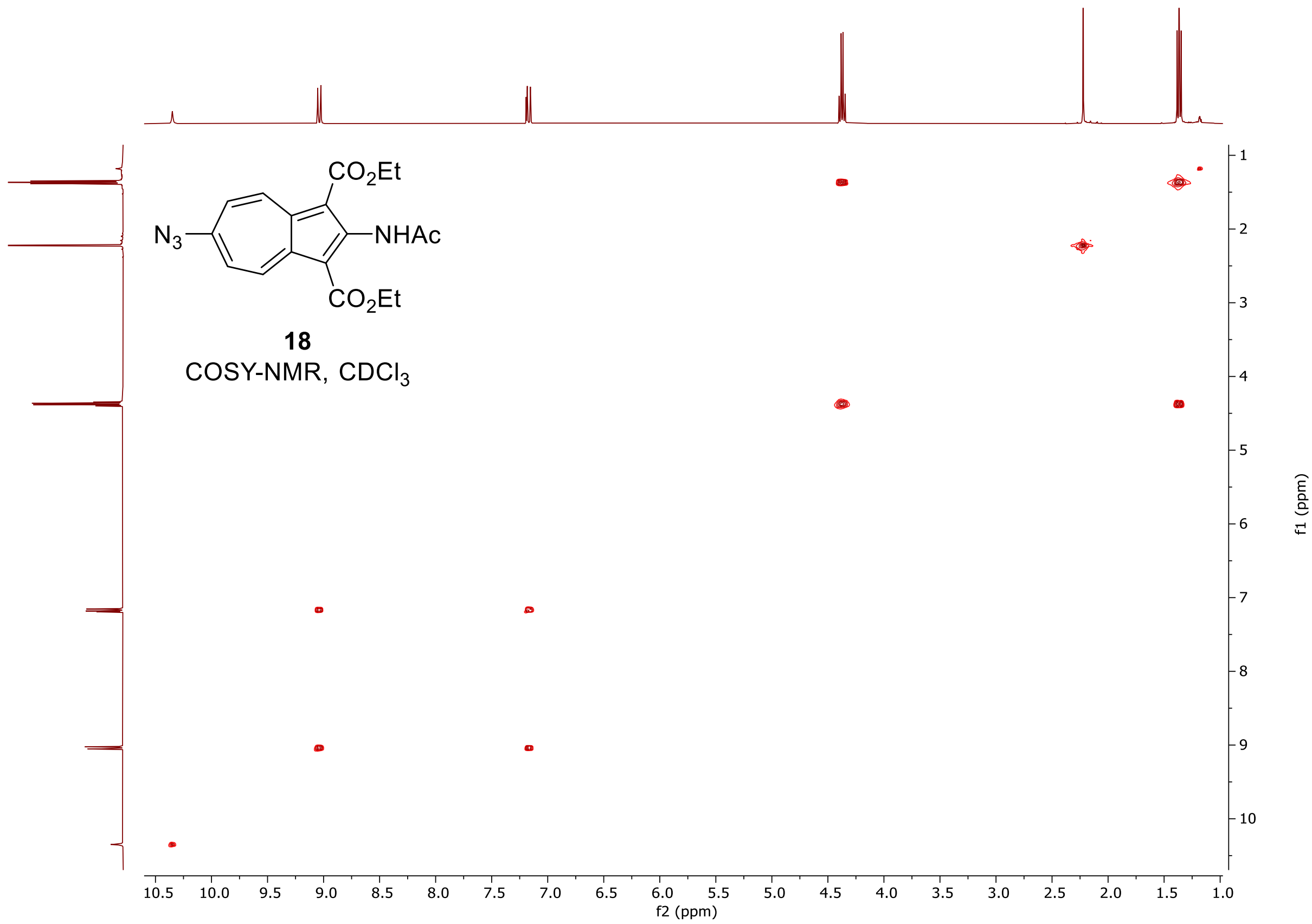
CO2Et
N3
NHAc
CO2Et
18
COSY-NMR, CDCl3
f2 (ppm)
f1 (ppm)

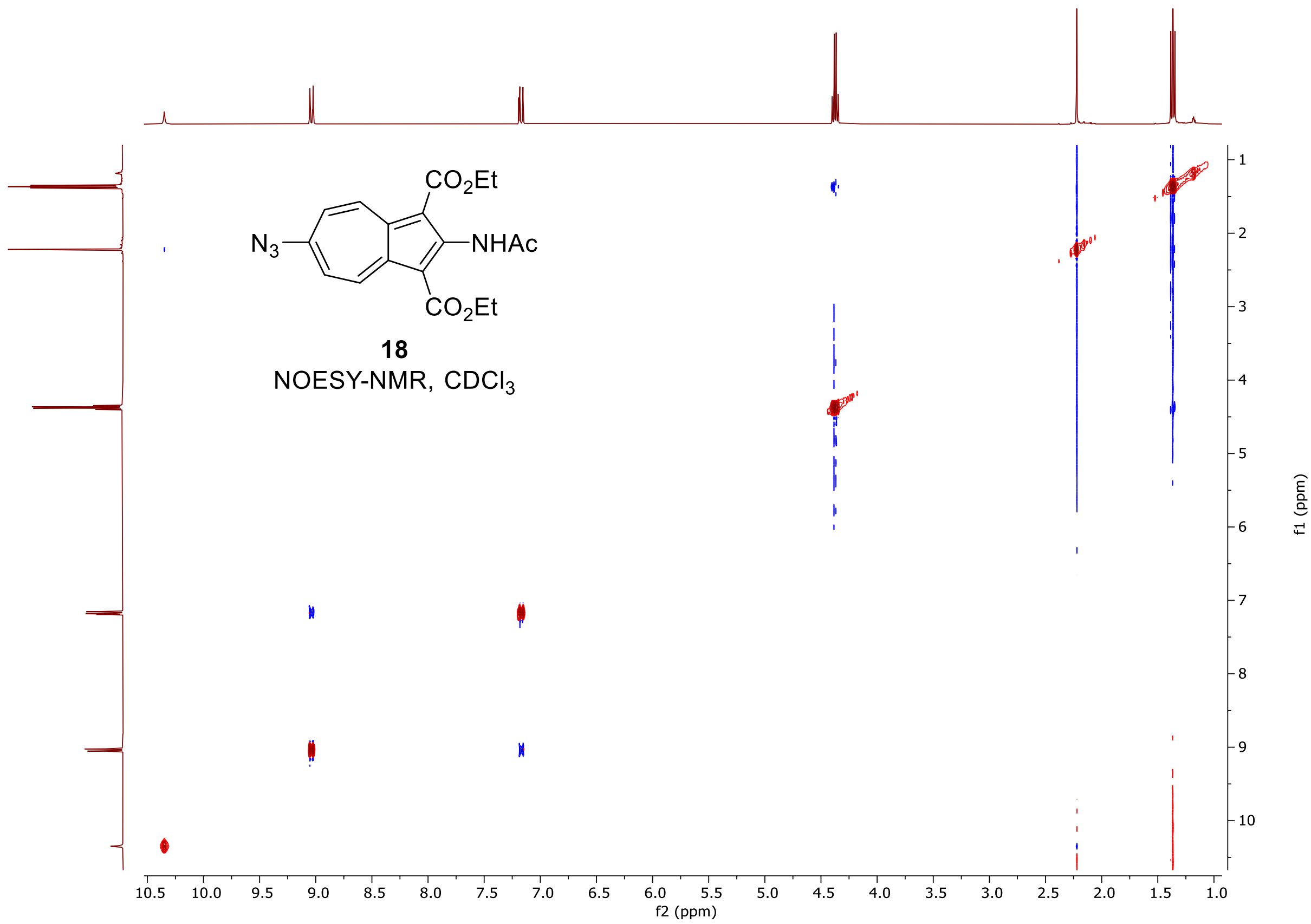
CO2Et
N3
NHAc
CO2Et
18
NOESY-NMR, CDCl3
f2 (ppm)
f1 (ppm)

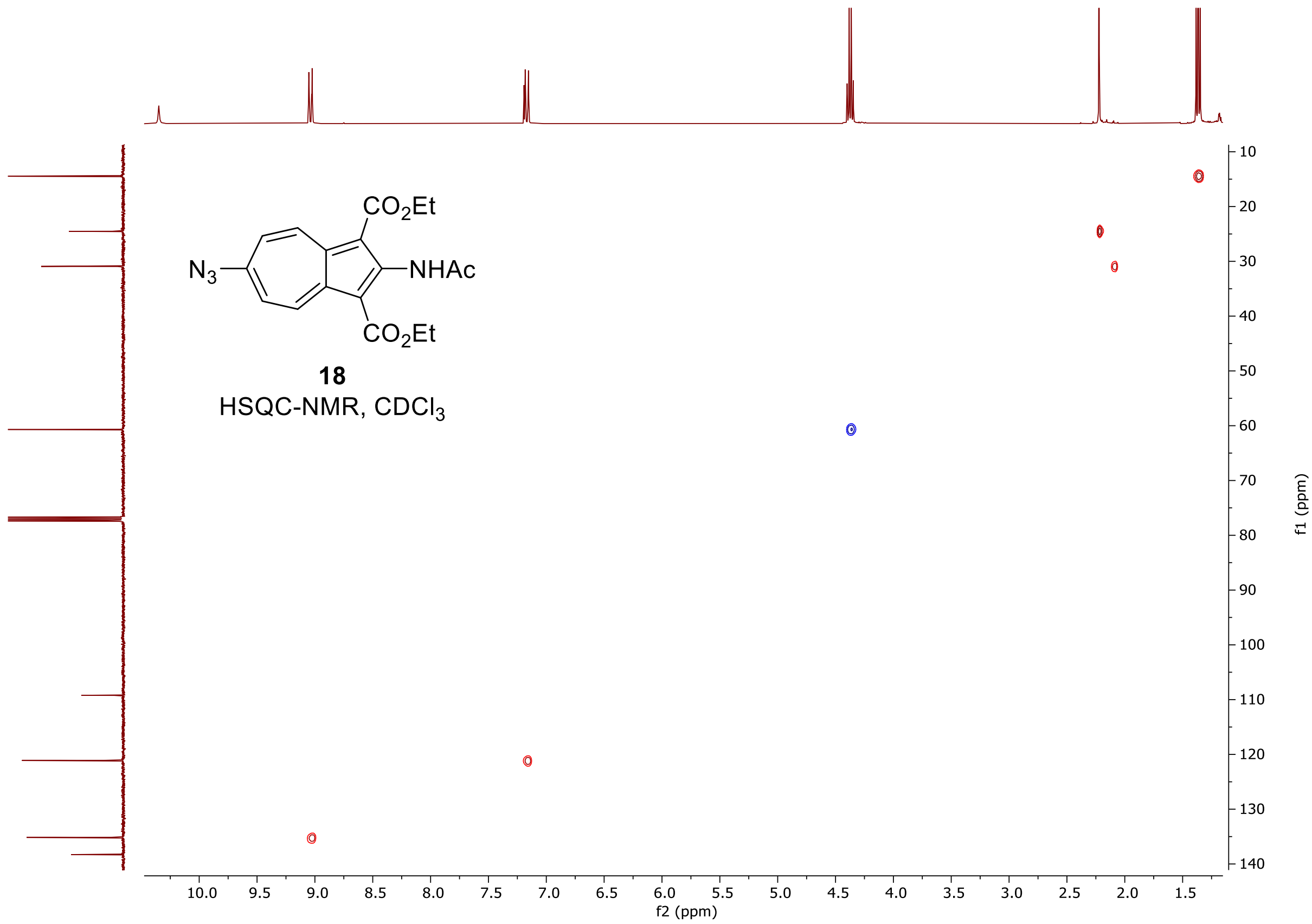

CO2Et
N3
NHAc
CO2Et
18
HSQC-NMR, CDCl3
f1 (ppm)
f2 (ppm)

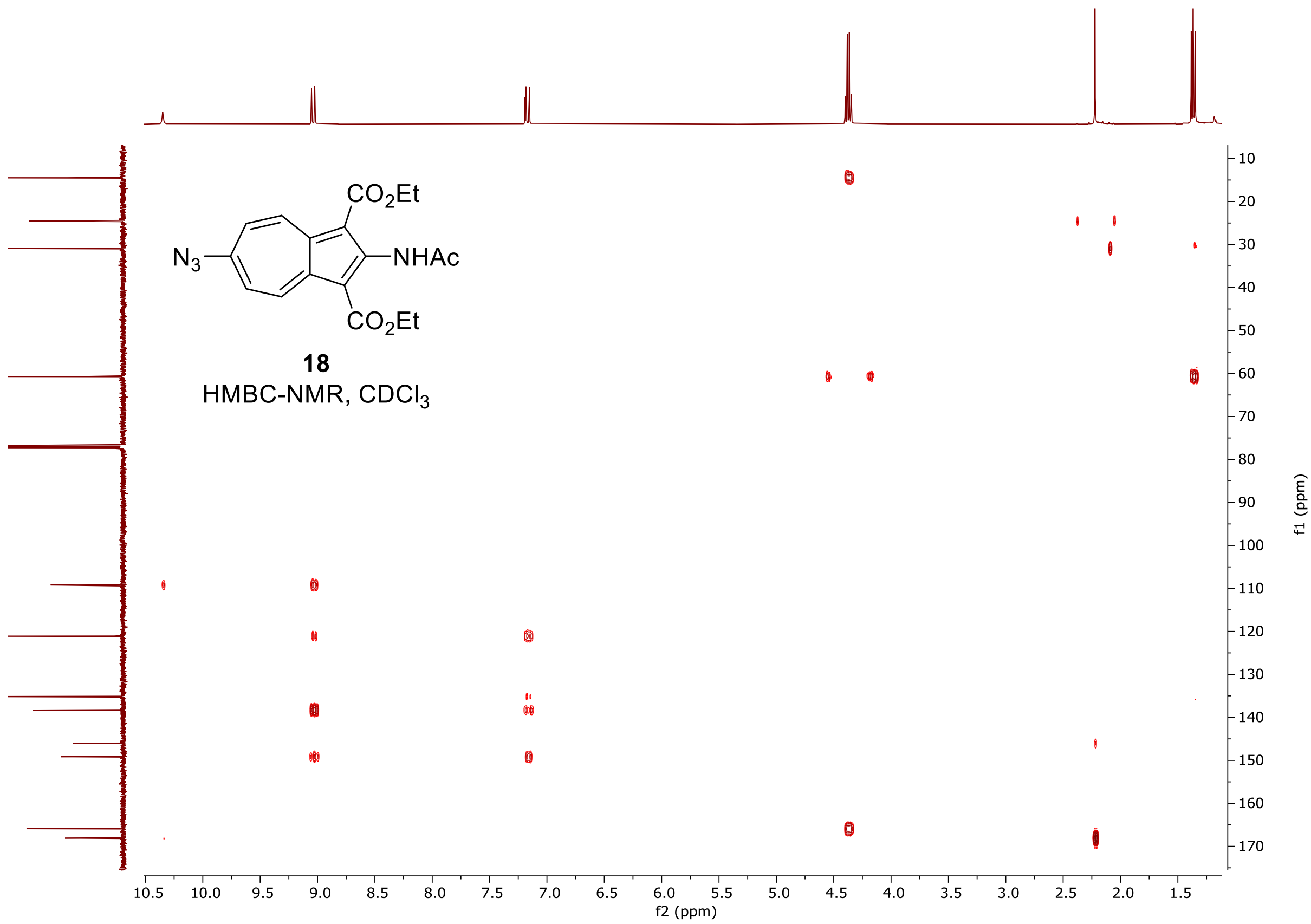

CO2Et
N3
NHAc
CO2Et
18
HMBC-NMR, CDCl3
f1 (ppm)
f2 (ppm)

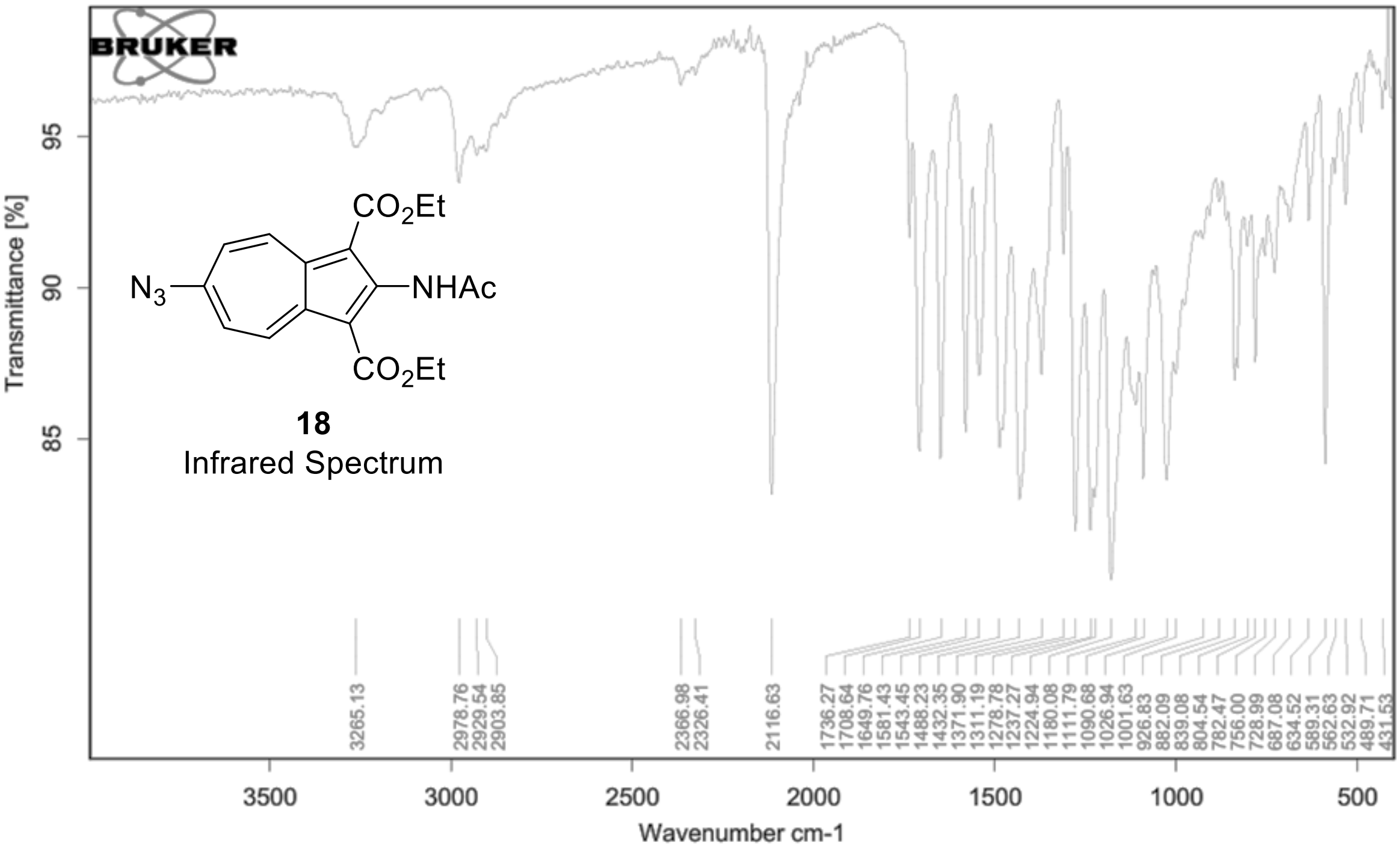

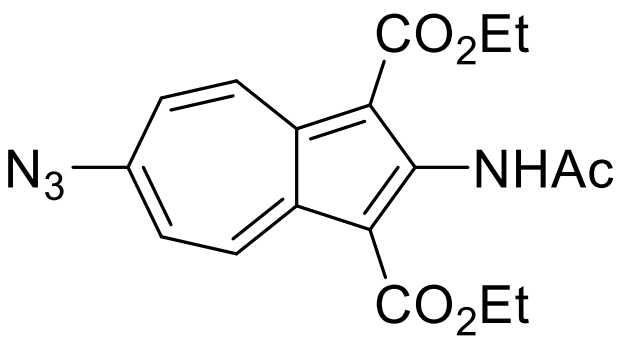

18

HRMS-ESI$^+$

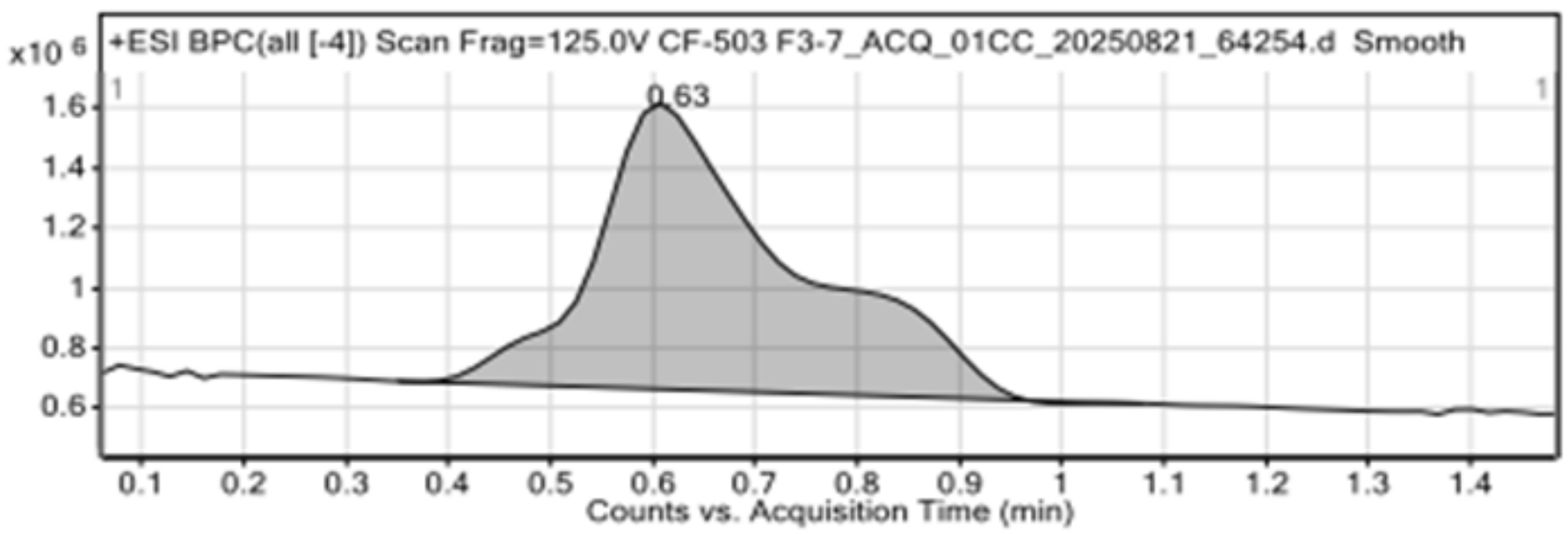

Figure 1: Base peak chromatogram

User Chromatogram Peak List

RT (min)	Area	Area %	Area Sum (%)	Base Peak (m/z)	Width (min)
0.63	12821919	100.00	100.00	763.2388	0.220

Compound Table

Compound Label	RT (min)	Observed mass (m/z)	Neutral observed mass (Da)	Theoretical mass (Da)	Mass error (ppm)	Isotope match score (%)
Cpd 1: C18 H18 N4 O5	0.64	393.1162	370.1271	370.1277	-1.65	99.35

Mass errors of between -5.00 and 5.00 ppm with isotope match scores above 60% are considered confirmation of molecular formulae

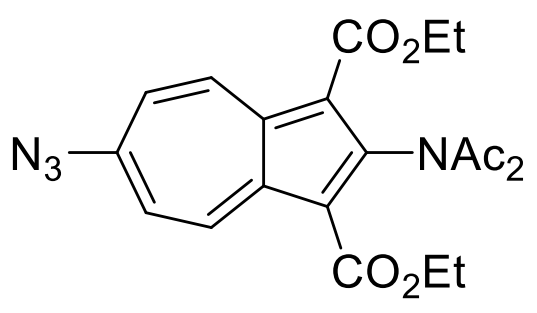

19

^{1}H-NMR, 400 MHz, $CDCl_3$

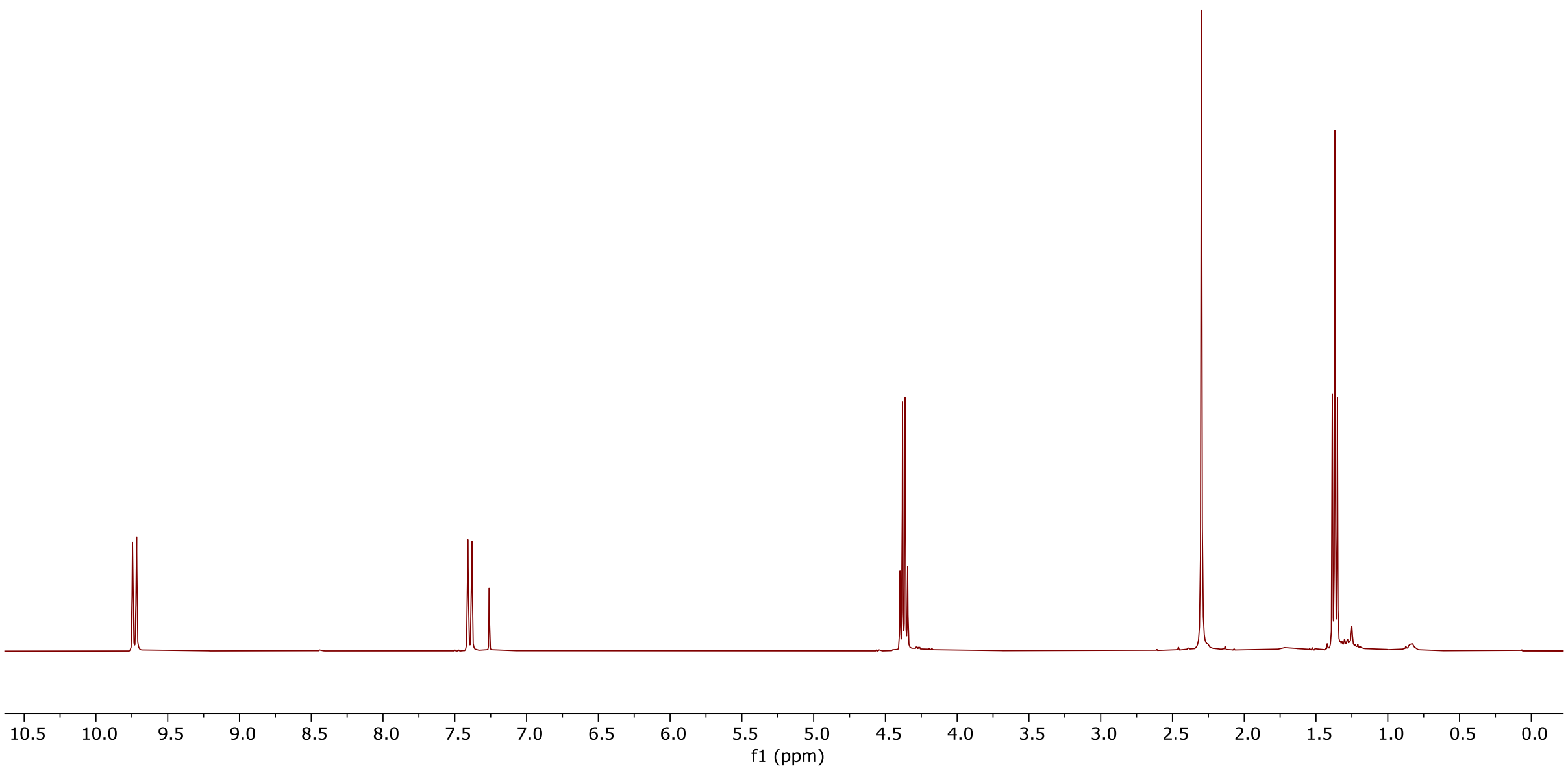

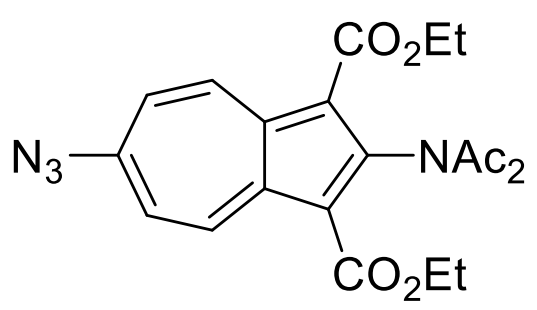

19

^{13}C-NMR, 101 MHz, $CDCl_3$

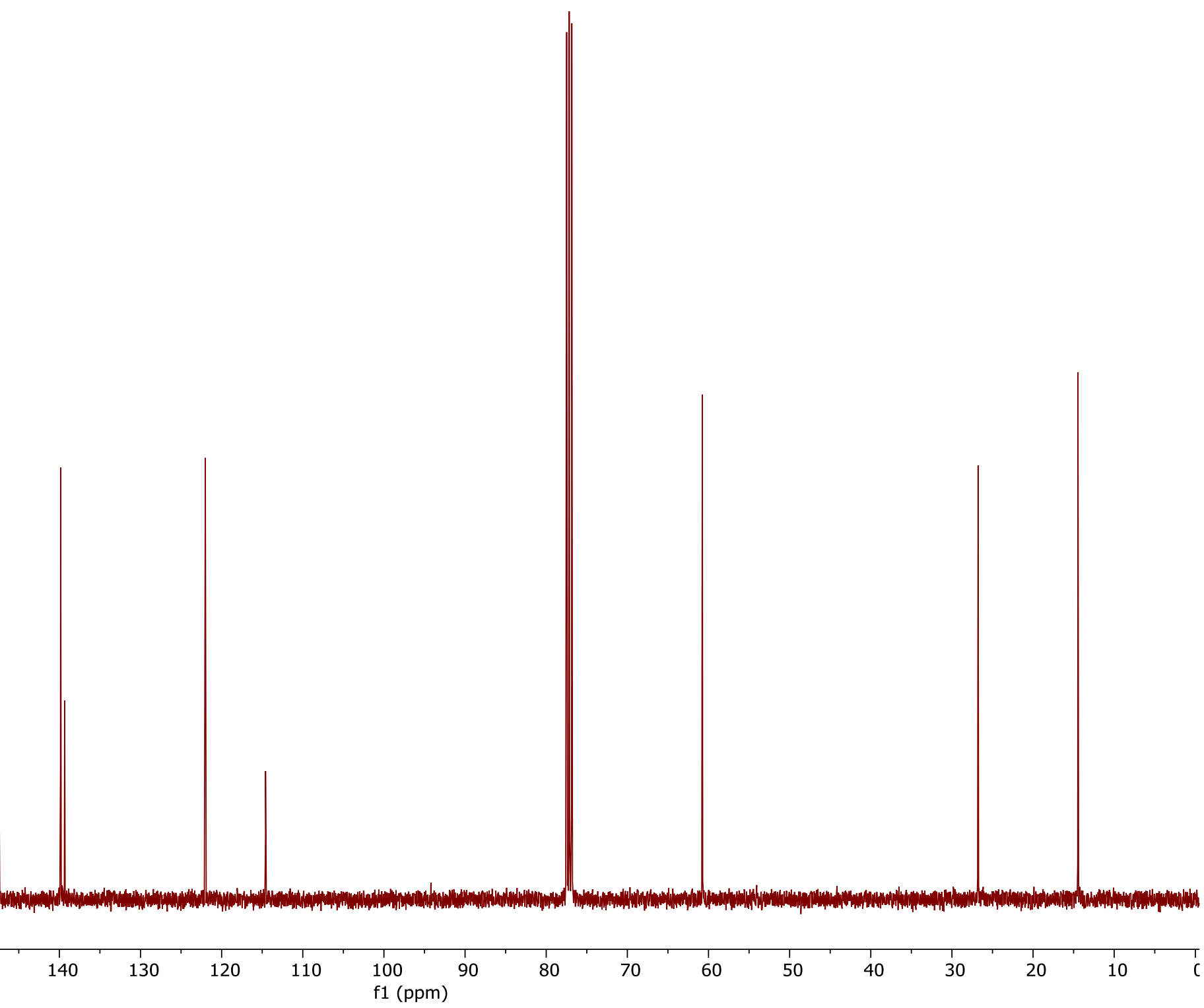

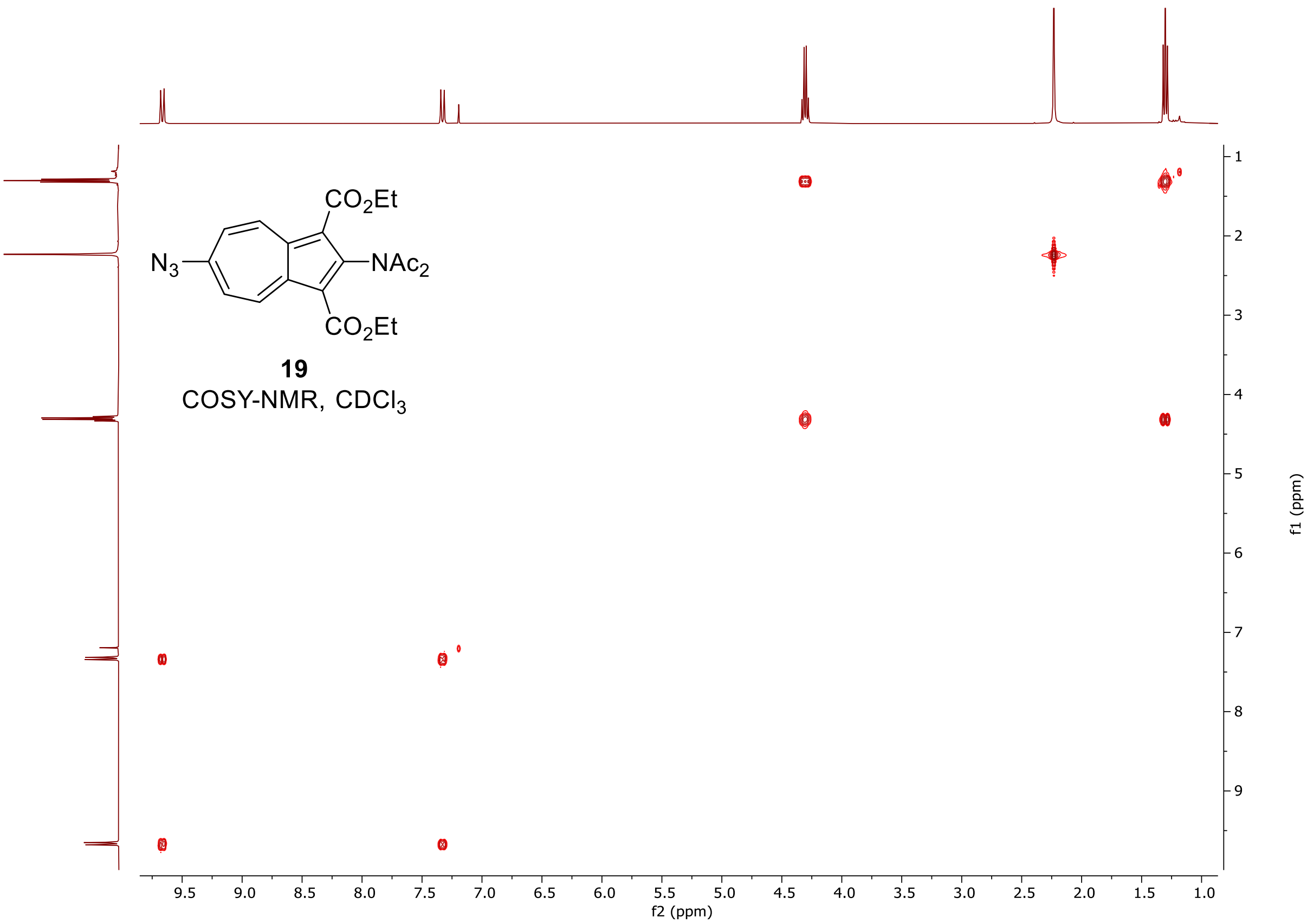
CO2Et
N3
NAc2
CO2Et
19
COSY-NMR, CDCl3
f2 (ppm)
f1 (ppm)

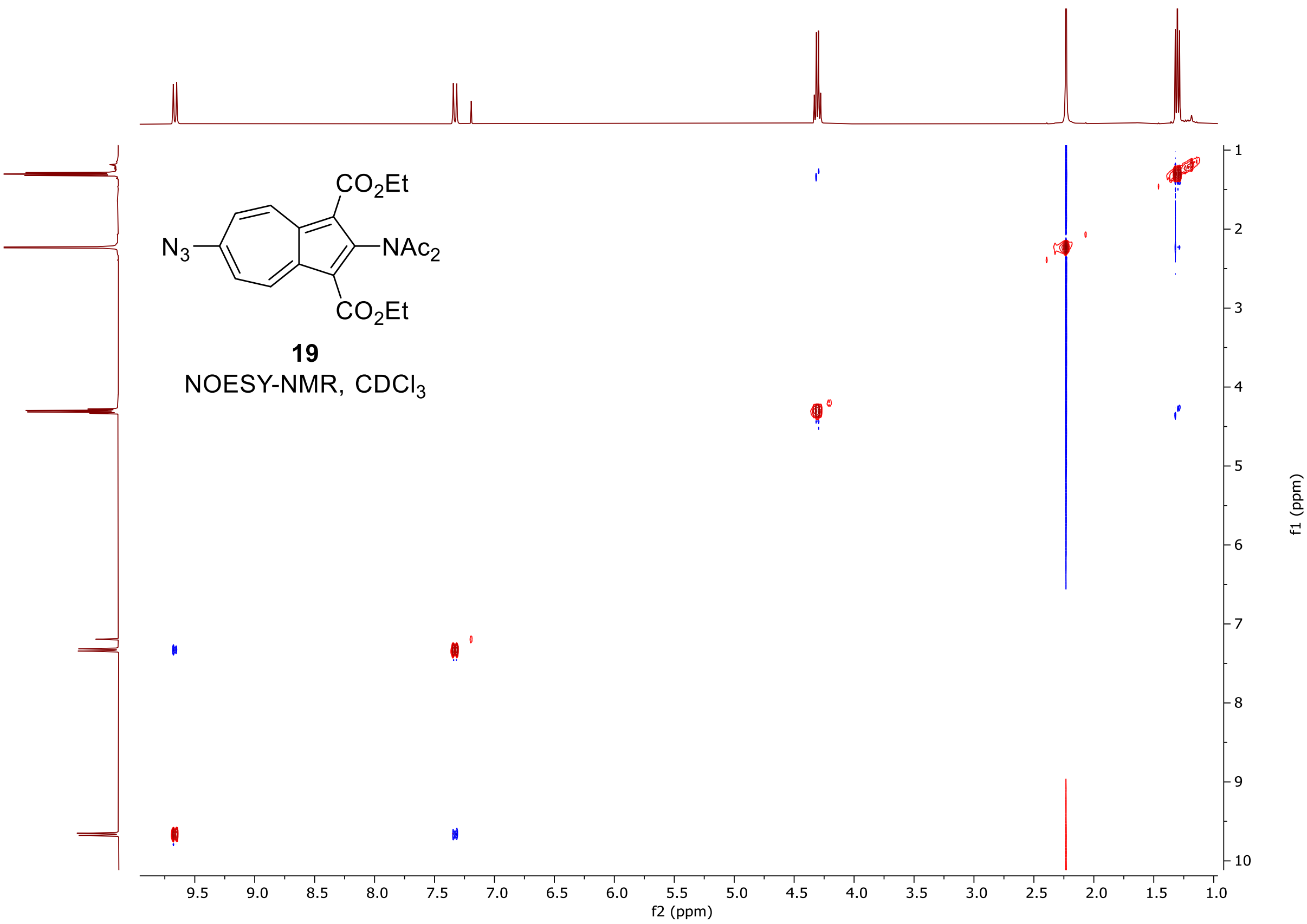
CO2Et
N3
NAc2
CO2Et
19
NOESY-NMR, CDCl3
f2 (ppm)
f1 (ppm)

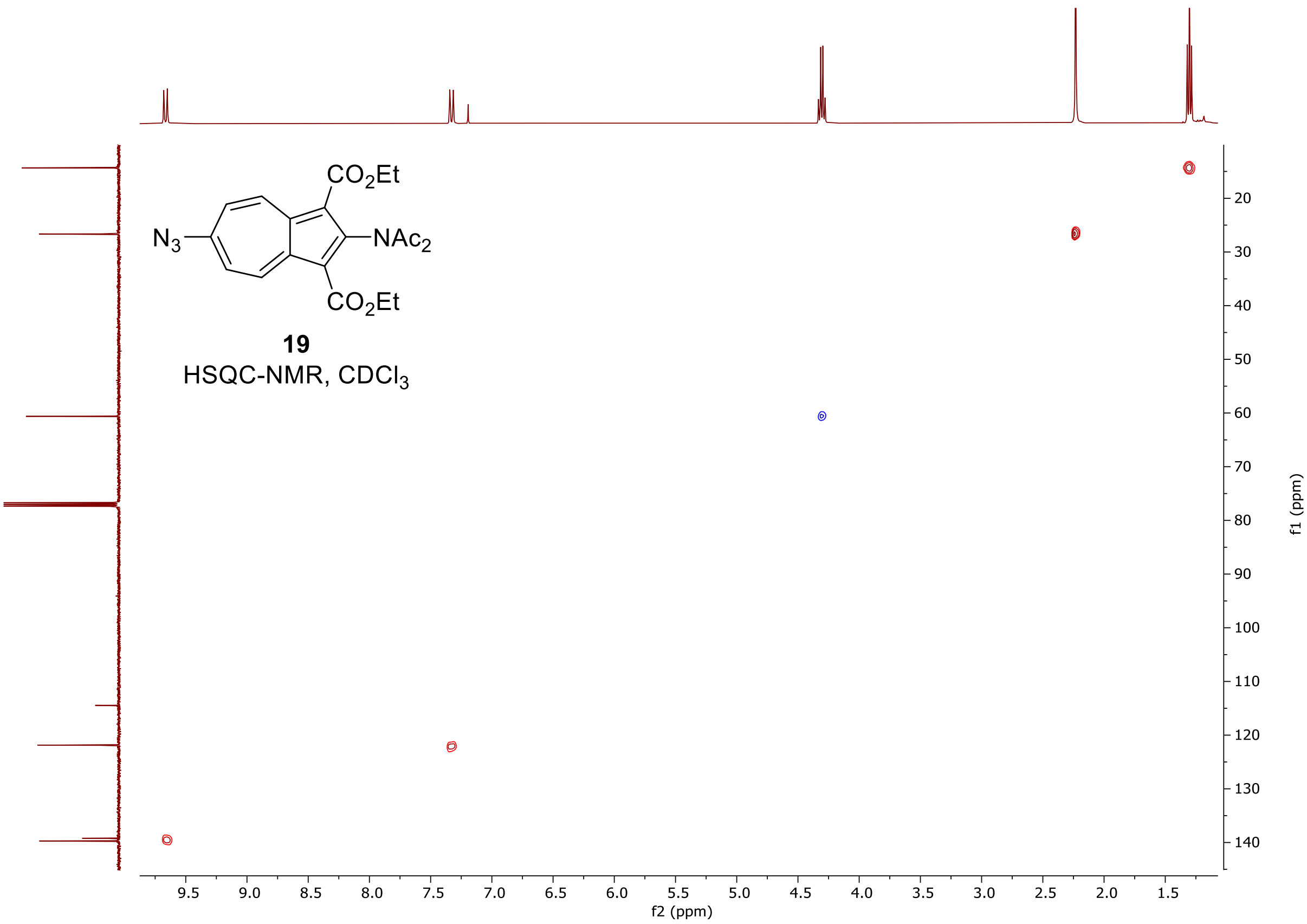

CO2Et
N3
NAc2
CO2Et
19
HSQC-NMR, CDCl3
f2 (ppm)
f1 (ppm)

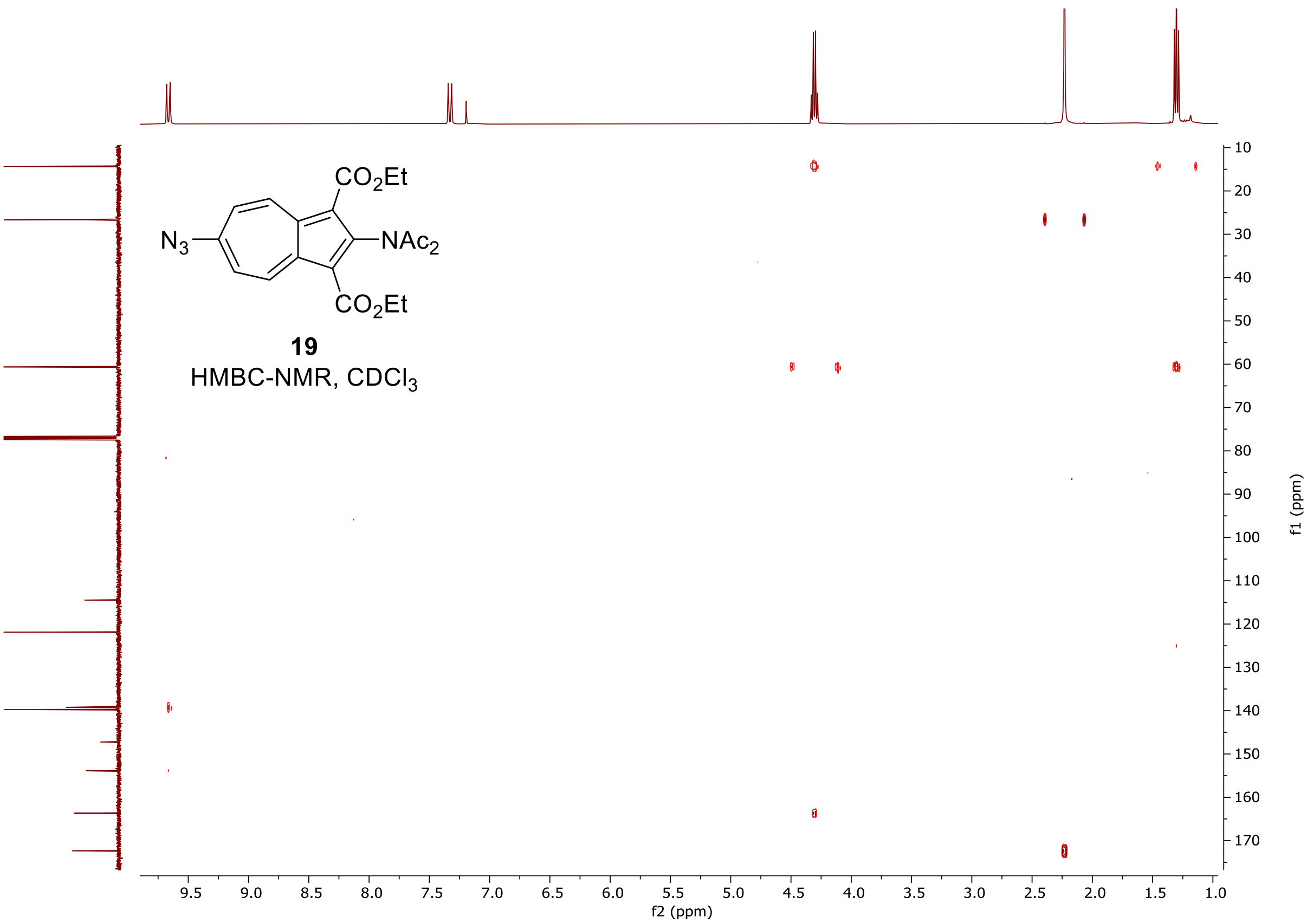

CO2Et
N3
NAc2
CO2Et
19
HMBC-NMR, CDCl3
f2 (ppm)
f1 (ppm)

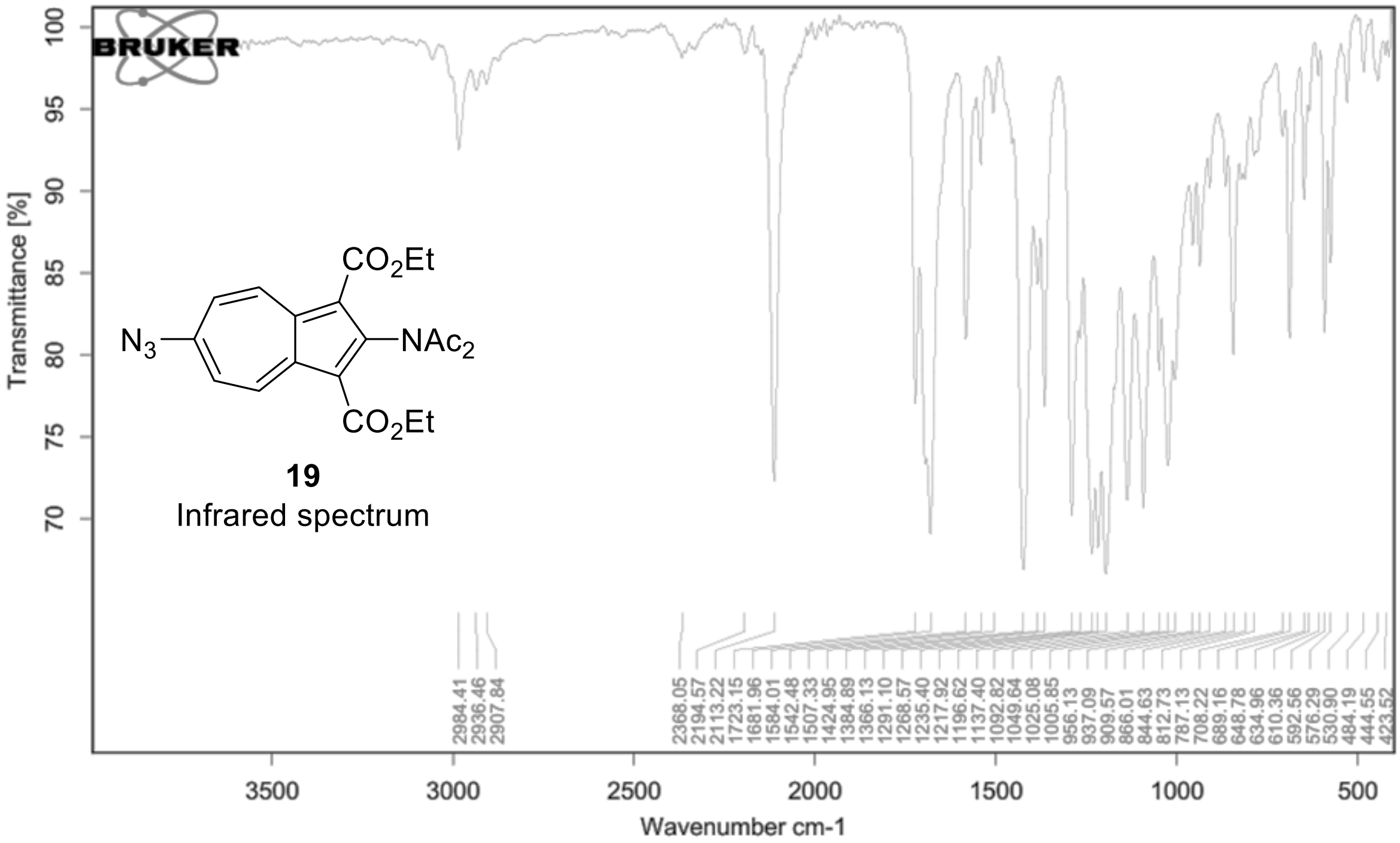

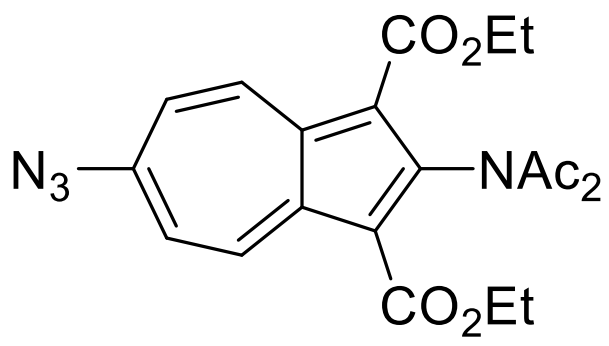

19

HRMS-ESI$^+$

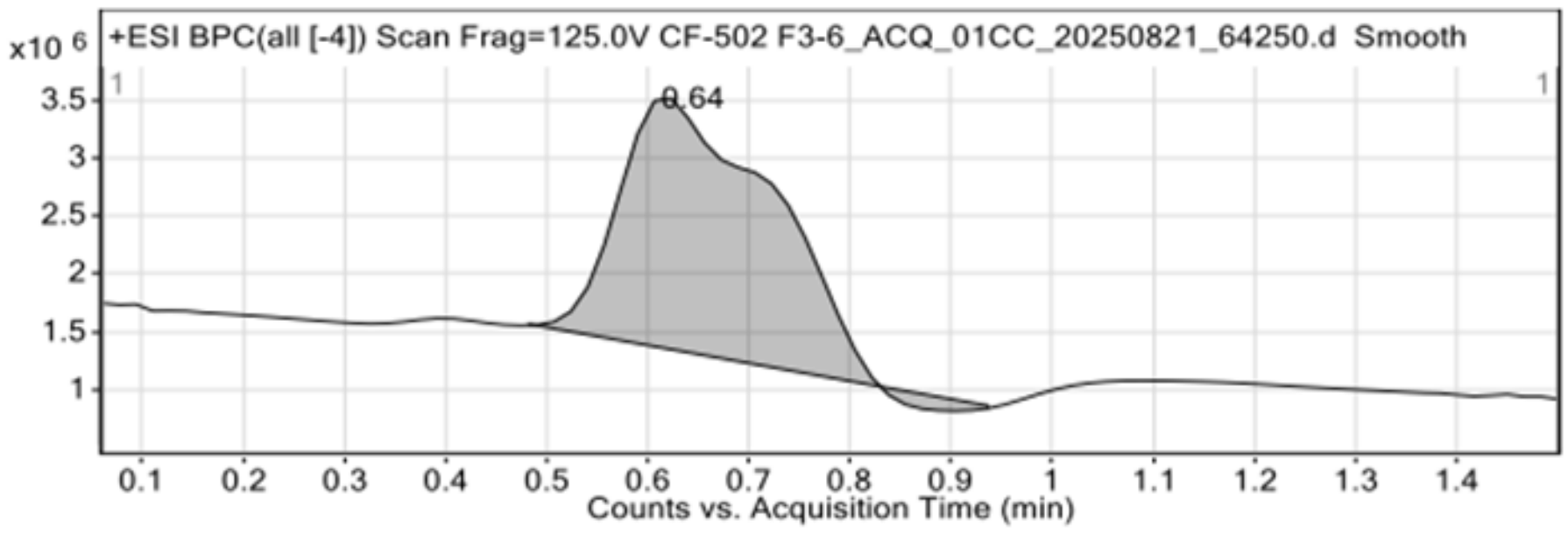

Figure 1: Base peak chromatogram

User Chromatogram Peak List

RT (min)	Area	Area %	Area Sum (%)	Base Peak (m/z)	Width (min)
0.64	22987301	100.00	100.00	435.1268	0.180

Compound Table

Compound Label	RT (min)	Observed mass (m/z)	Neutral observed mass (Da)	Theoretical mass (Da)	Mass error (ppm)	Isotope match score (%)
Cpd 1: C20 H20 N4 O6	0.67	435.1268	412.1376	412.1383	-1.55	98.25

Mass errors of between -5.00 and 5.00 ppm with isotope match scores above 60% are considered confirmation of molecular formulae

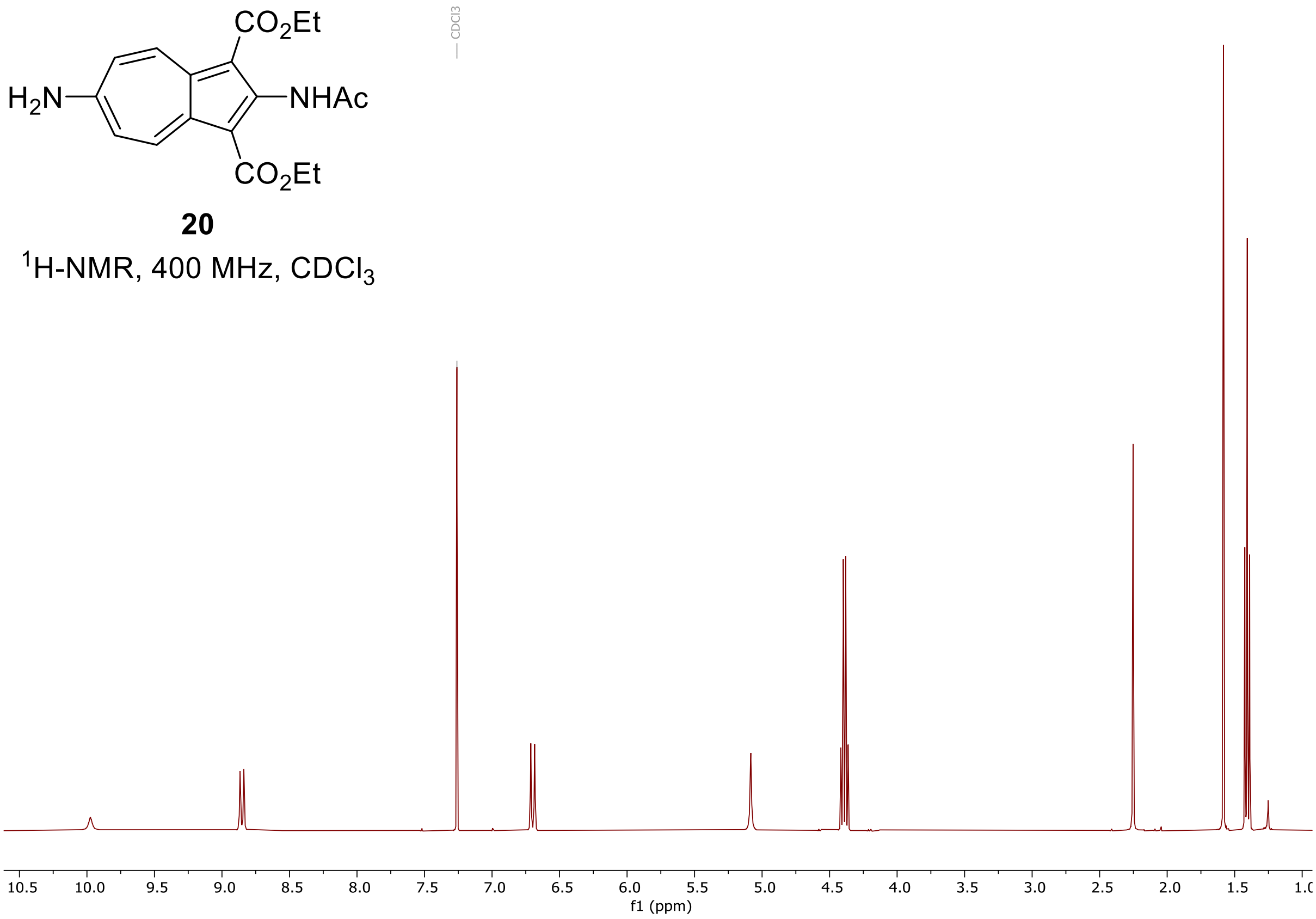

20

^{1}H-NMR, 400 MHz, $CDCl_3$

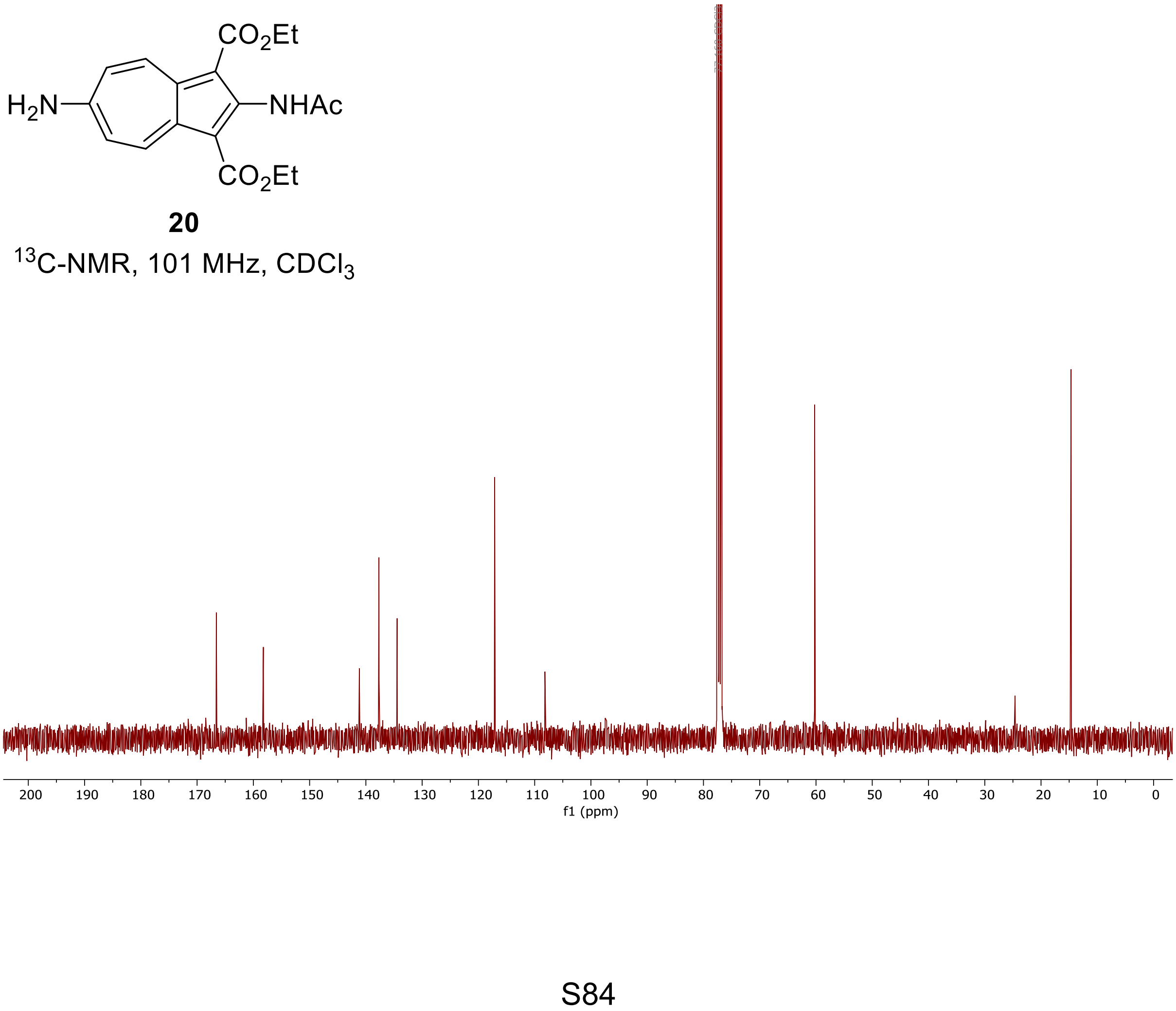

20

^{13}C-NMR, 101 MHz, $CDCl_3$

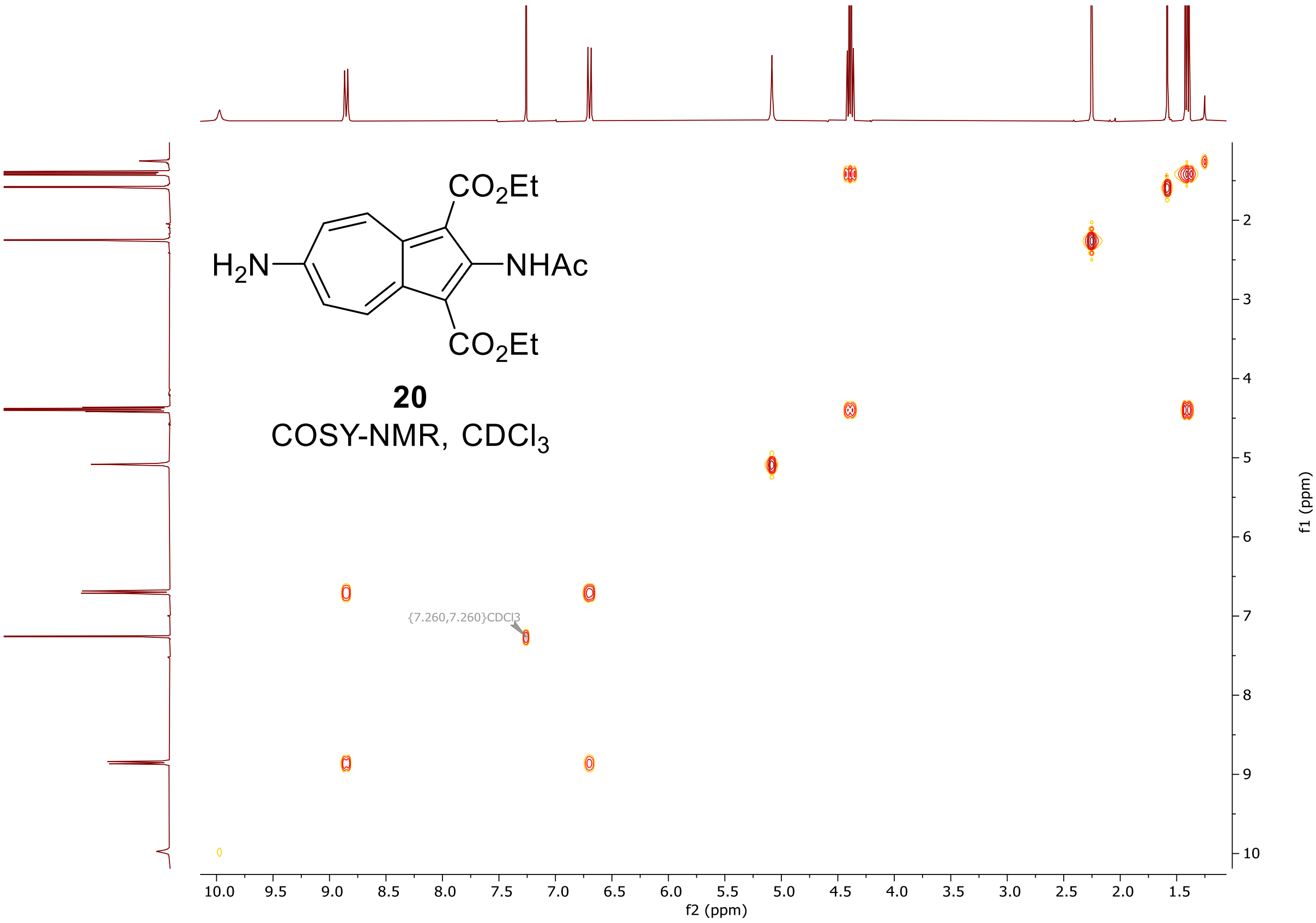
CO2Et
H2N
NHAc
CO2Et
20
COSY-NMR, CDCl3
{7.260,7.260}CDCl3
f2 (ppm)
f1 (ppm)

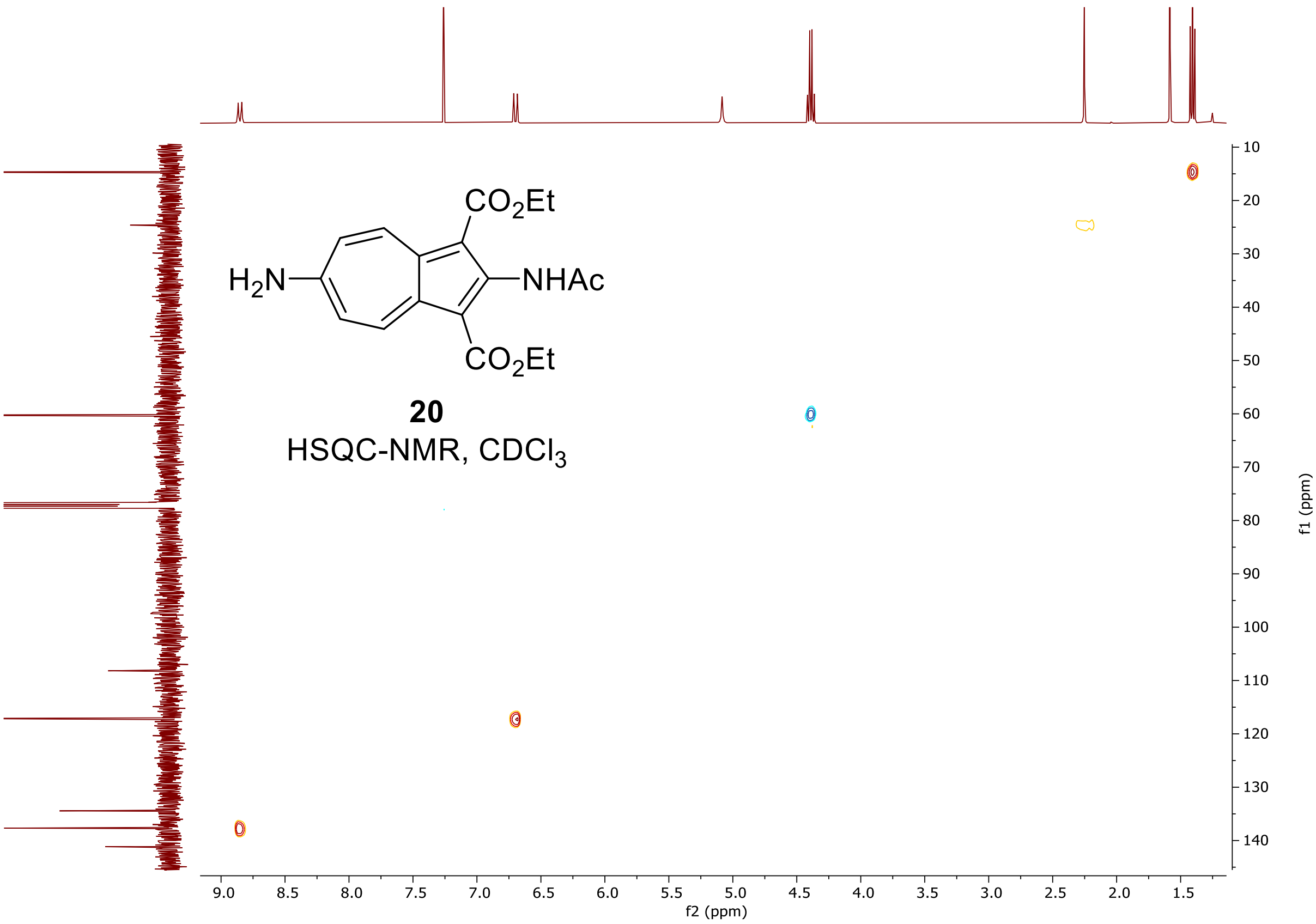
CO2Et
H2N
NHAc
CO2Et
20
HSQC-NMR, CDCl3
f2 (ppm)
f1 (ppm)

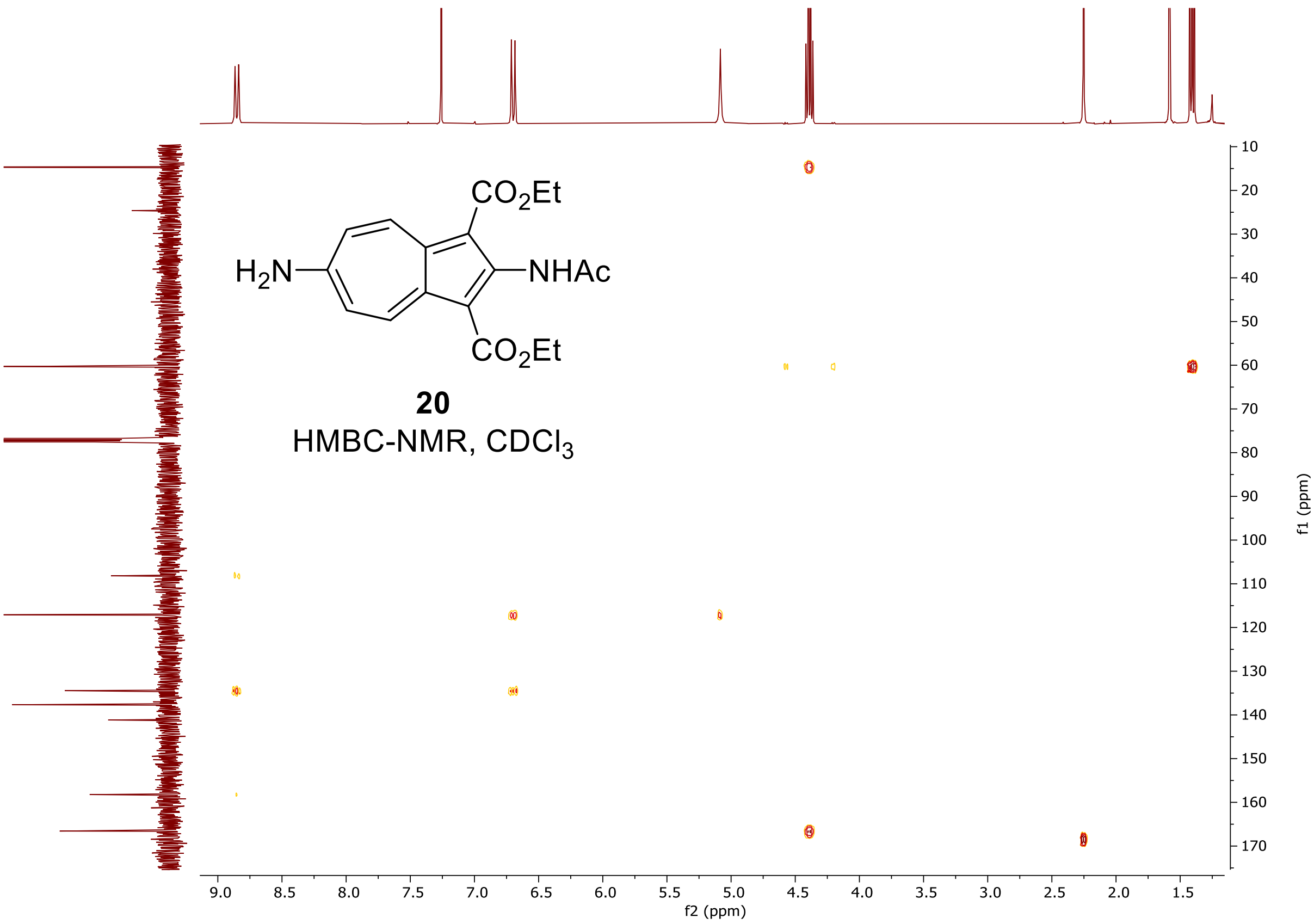
CO2Et
H2N
NHAc
CO2Et
20
HMBC-NMR, CDCl3
f2 (ppm)
f1 (ppm)

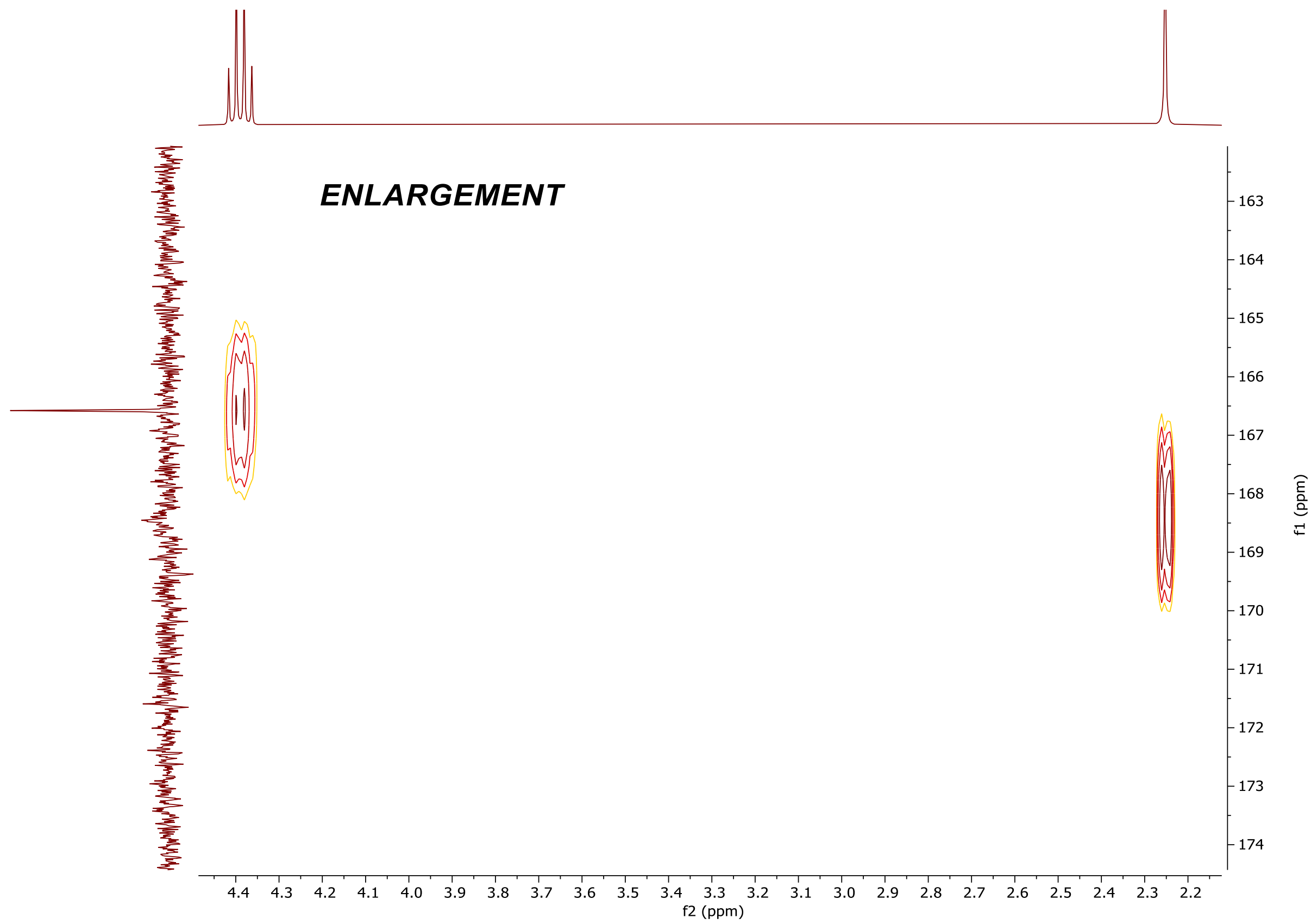
ENLARGEMENT
f2 (ppm)
f1 (ppm)

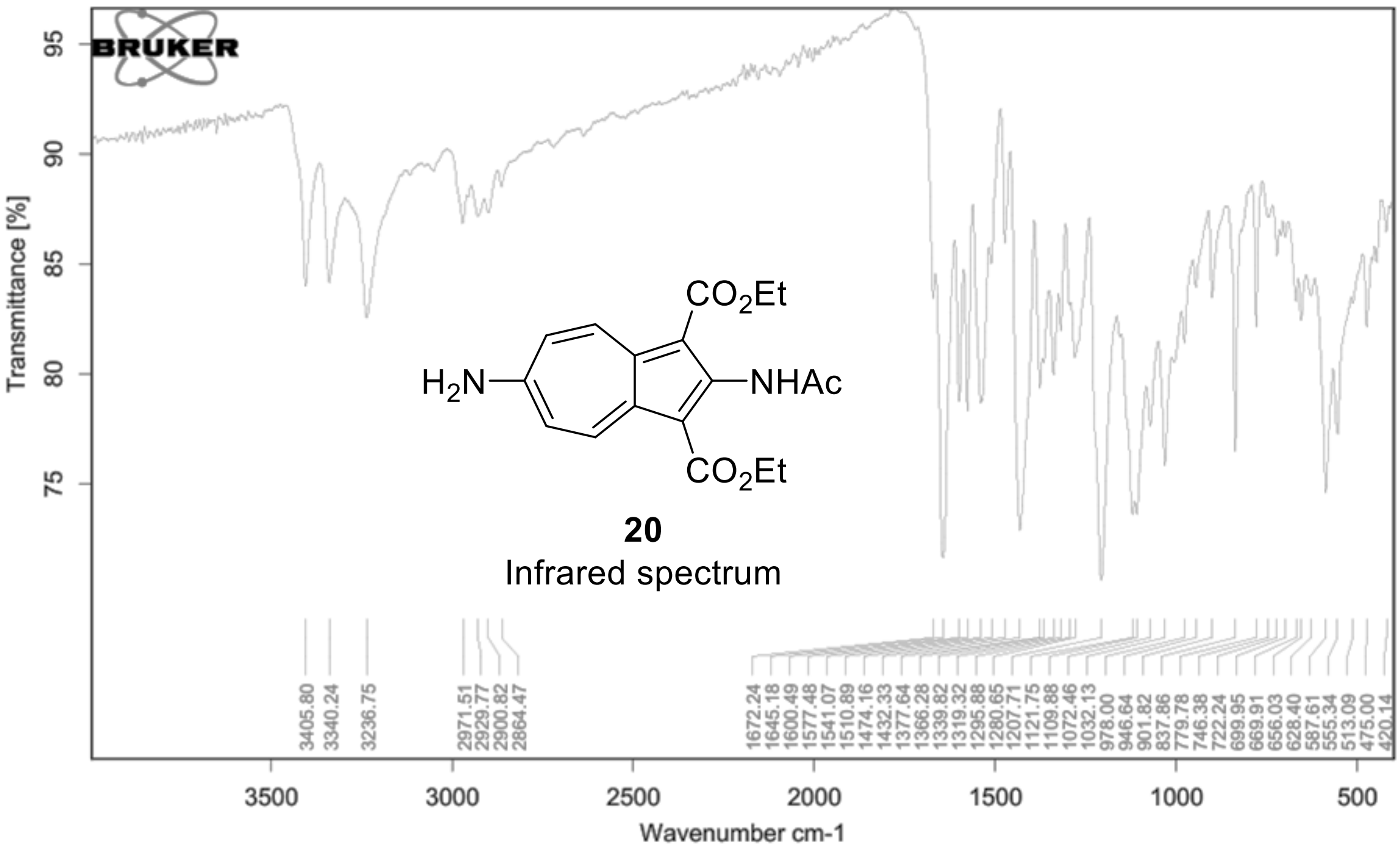

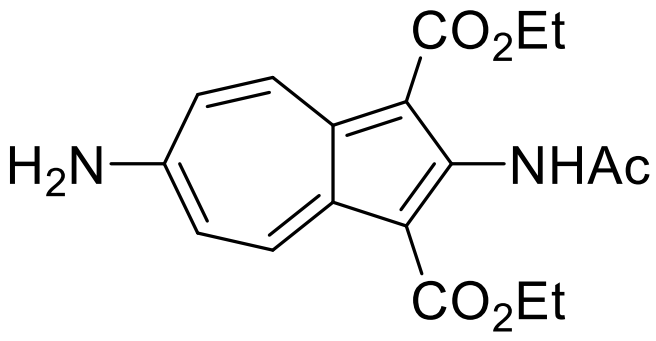

20

HRMS-ESI$^+$

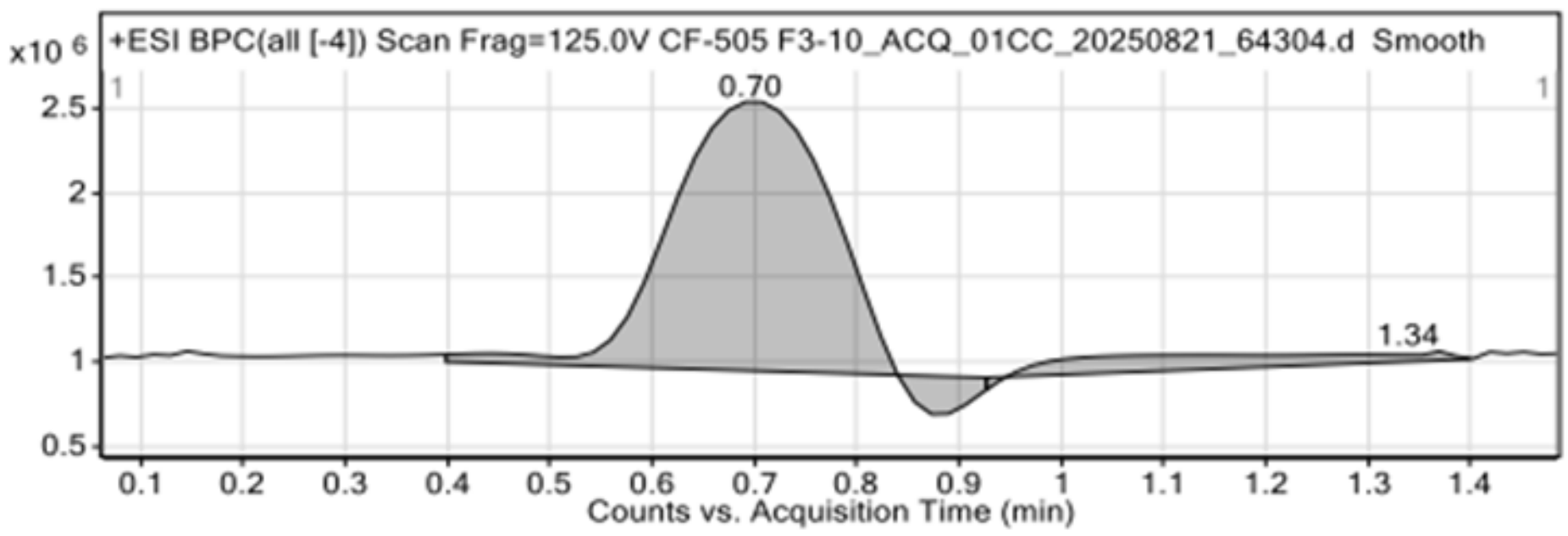

Figure 1: Base peak chromatogram

User Chromatogram Peak List

RT (min)	Area	Area %	Area Sum (%)	Base Peak (m/z)	Width (min)
0.70	16460446	100.00	90.04	418.2266	0.180
1.34	1820700	11.06	9.96	149.9524	0.550

Compound Table

Compound Label	RT (min)	Observed mass (m/z)	Neutral observed mass (Da)	Theoretical mass (Da)	Mass error (ppm)	Isotope match score (%)
Cpd 1: C18 H20 N2 O5	0.68	367.1261	344.1369	344.1372	-0.80	99.10

Mass errors of between -5.00 and 5.00 ppm with isotope match scores above 60% are considered confirmation of molecular formulae

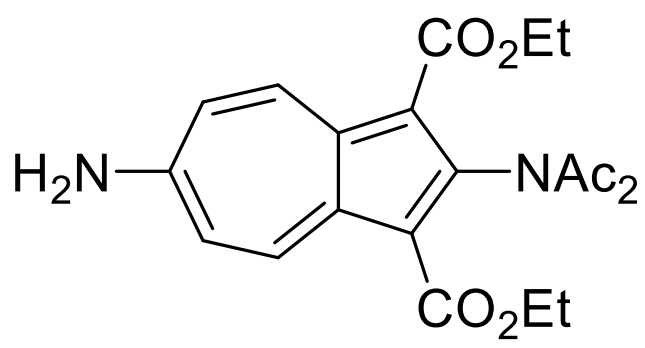

21

^{1}H-NMR, 400 MHz, $CDCl_3$

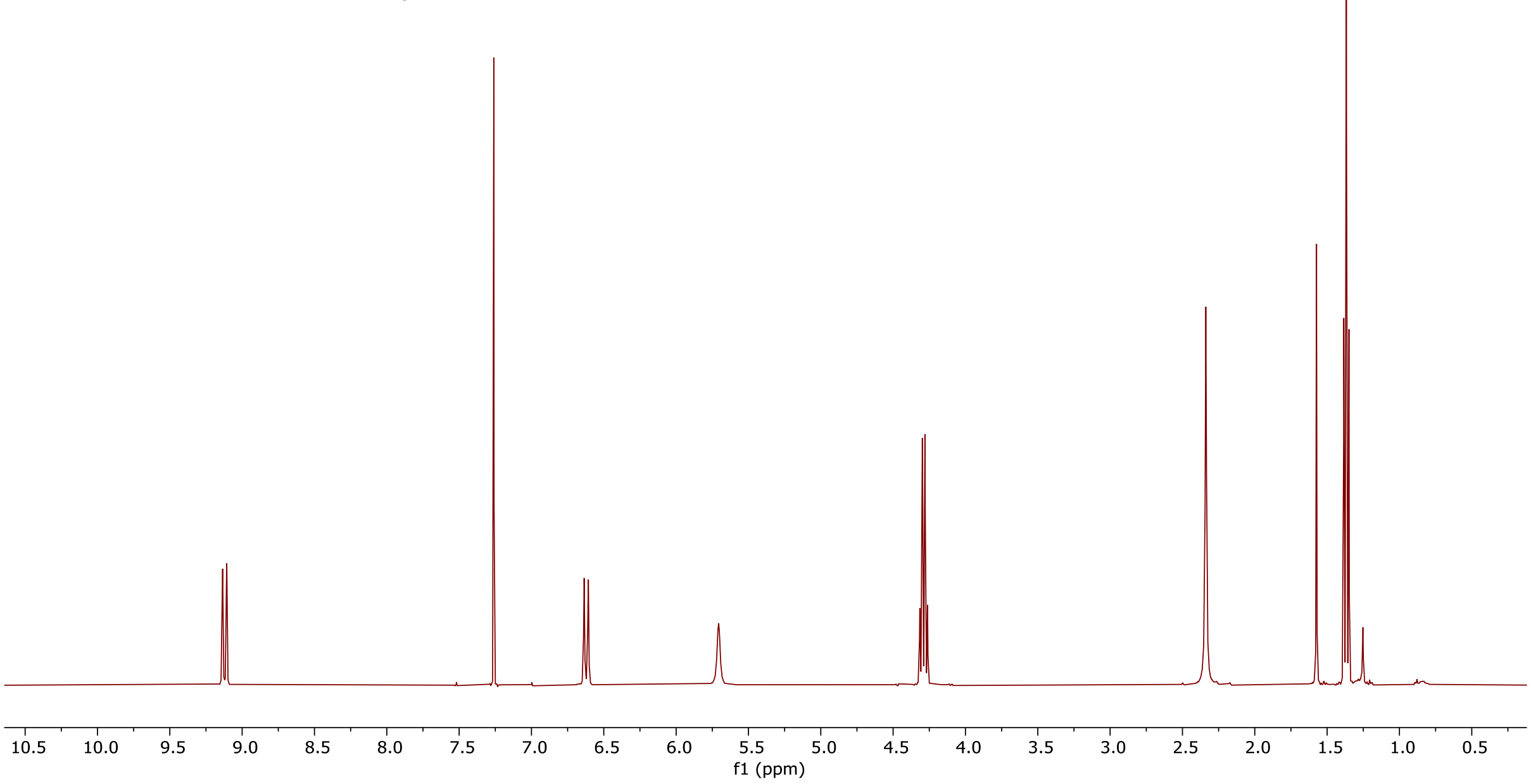

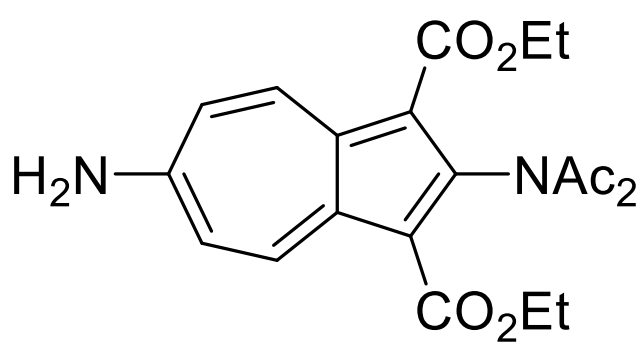

21

^{13}C-NMR, 126 MHz, $CDCl_3$

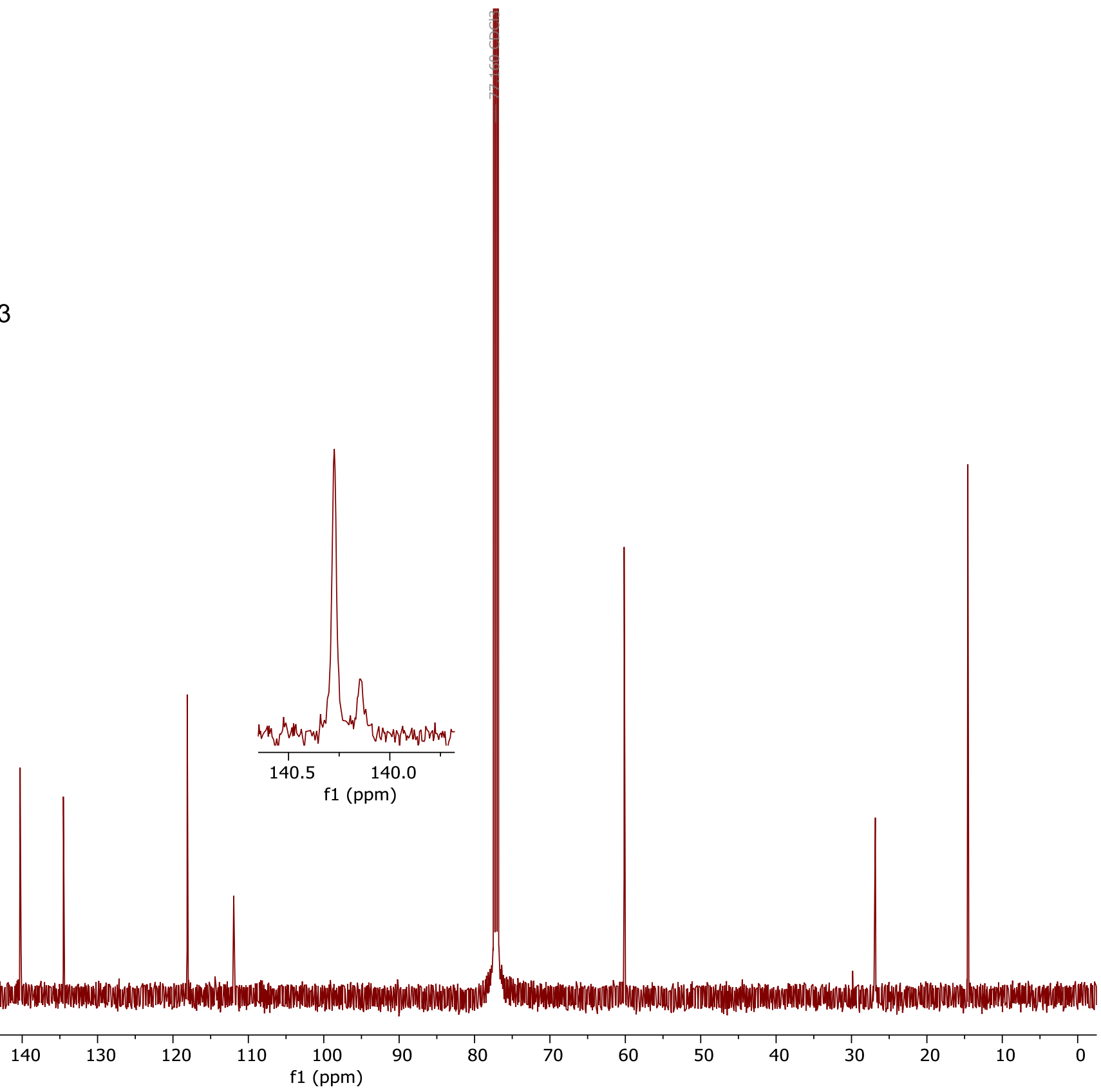

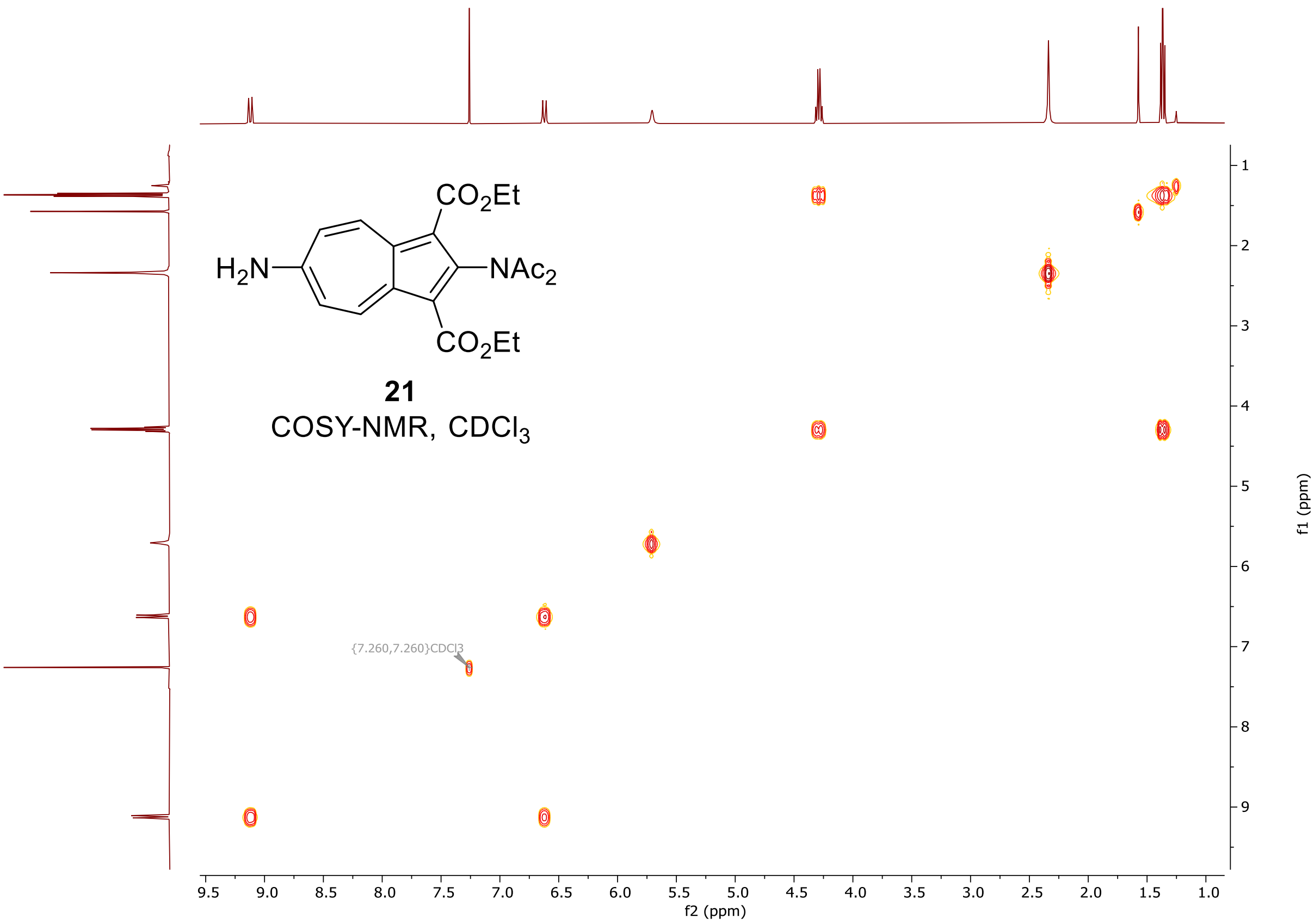

CO2Et
H2N
NAc2
CO2Et
21
COSY-NMR, CDCl3
{7.260,7.260}CDCl3
f2 (ppm)
f1 (ppm)

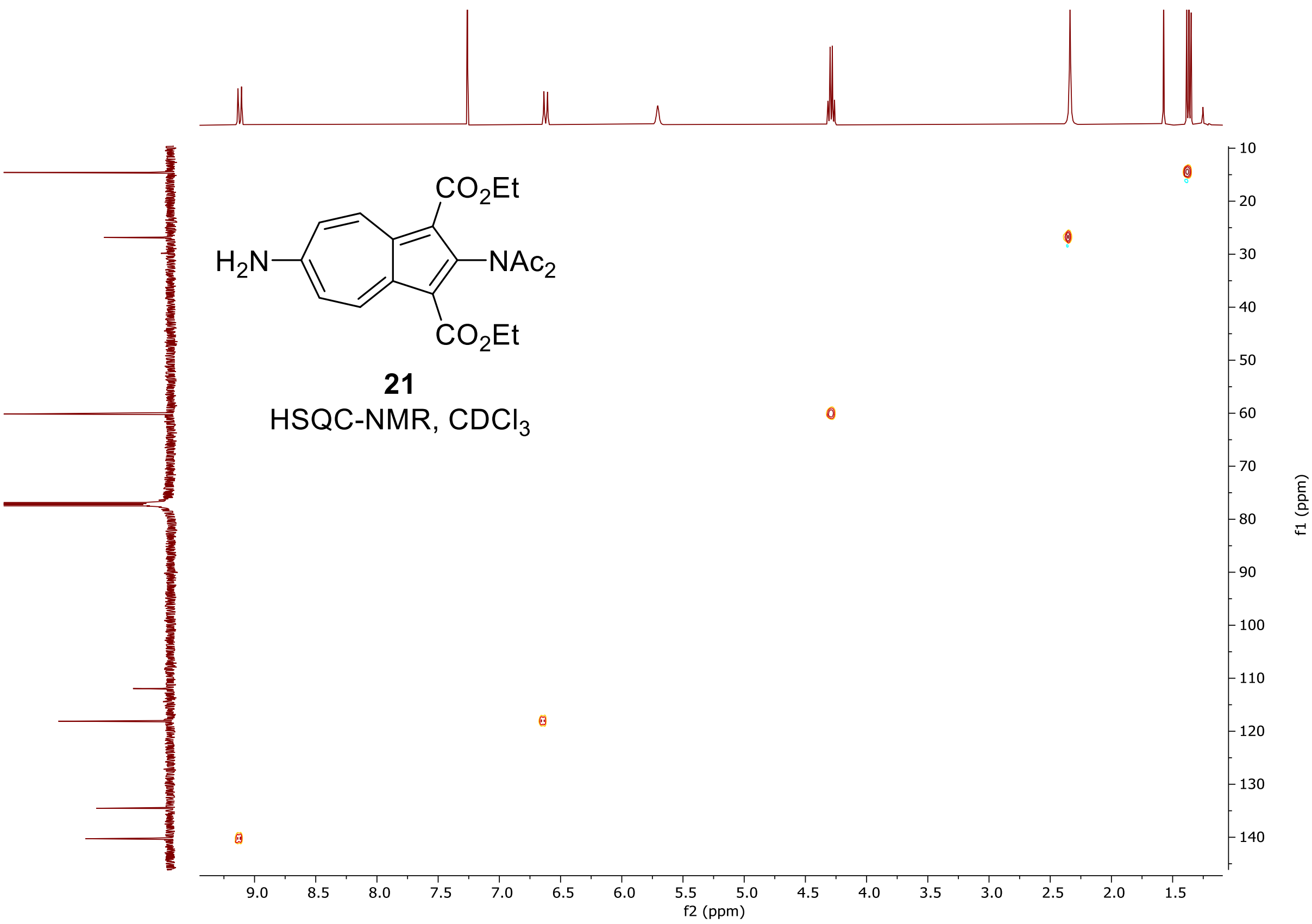

CO2Et
H2N
NAc2
CO2Et
21
HSQC-NMR, CDCl3
f2 (ppm)
f1 (ppm)

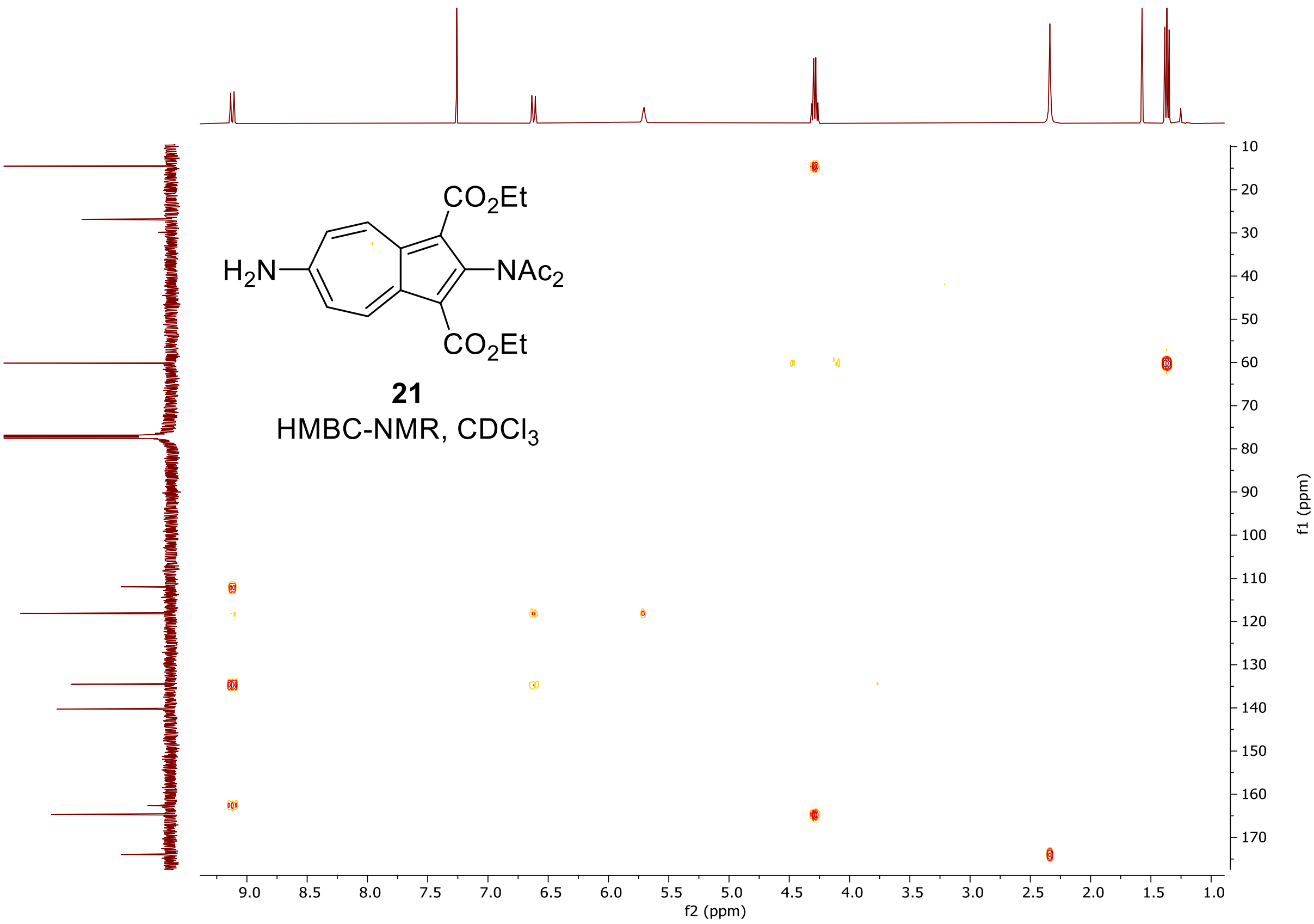
CO2Et
H2N
NAc2
CO2Et
21
HMBC-NMR, CDCl3
f2 (ppm)
f1 (ppm)

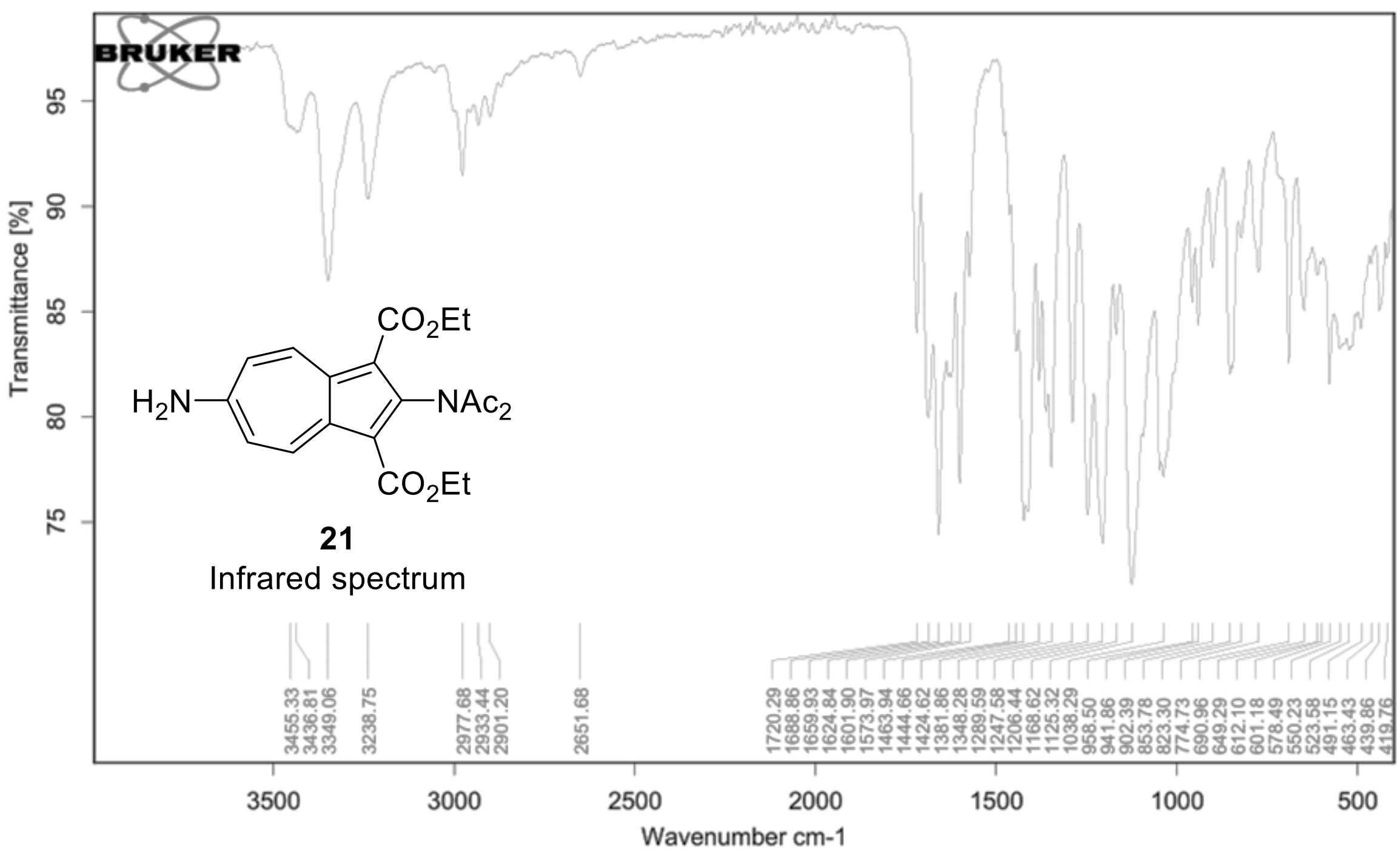
BRUKER
Transmittance [%]
CO2Et
H2N
NAc2
CO2Et
21
Infrared spectrum
3455.33
3436.81
3349.06
3238.75
2977.68
2933.44
2901.20
2651.68
1720.29
1688.86
1659.93
1624.84
1601.90
1573.97
1463.94
1444.66
1424.62
1381.86
1348.28
1289.59
1247.58
1206.44
1168.62
1125.32
1038.29
958.50
941.86
902.39
853.78
823.30
774.73
690.96
649.29
612.10
601.18
578.49
550.23
523.58
491.15
463.43
439.86
419.76
Wavenumber cm-1

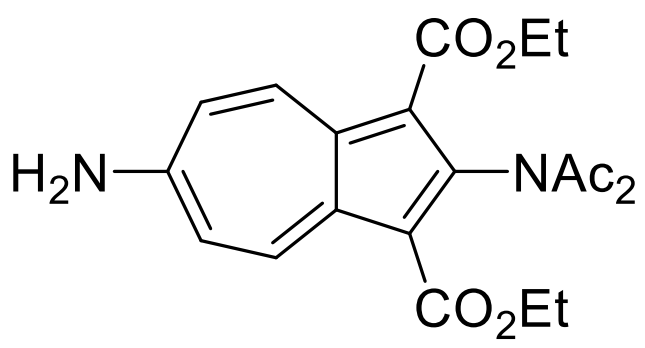

21, HRMS-ESI$^+$

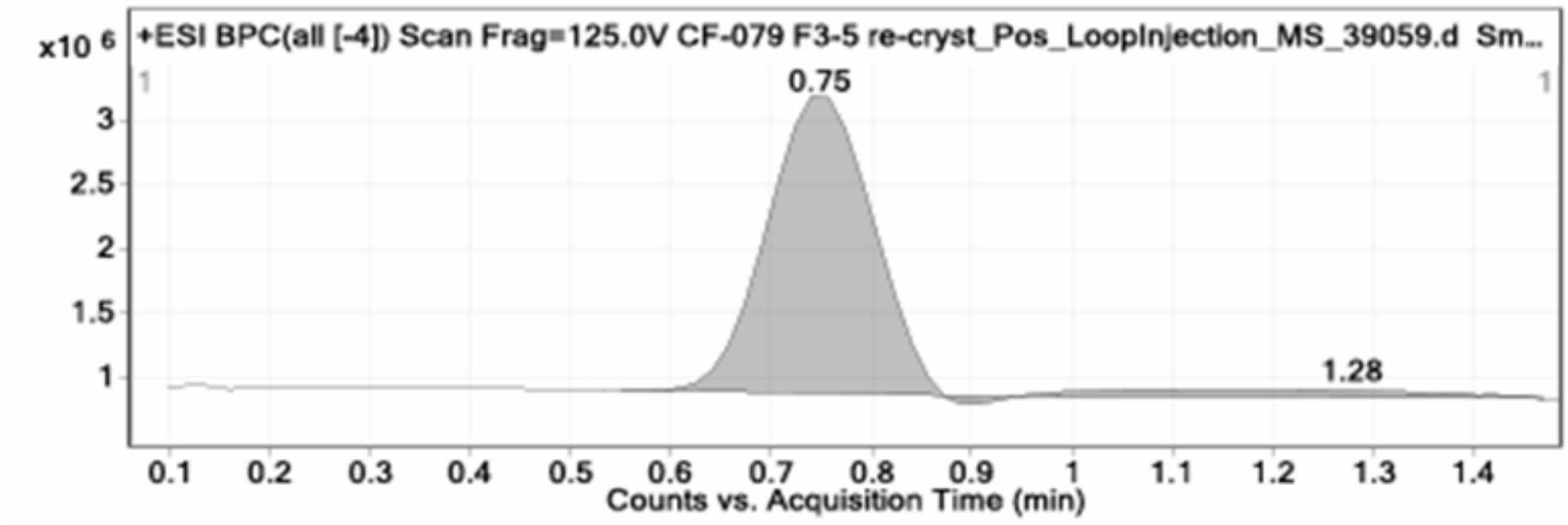

Figure 1: Base peak chromatogram

User Chromatogram Peak List

RT (min)	Area	Area %	Area Sum (%)	Base Peak (m/z)	Width (min)
0.75	16751512	100.00	92.21	795.2824	0.150
1.28	1415472	8.45	7.79	122.0968	0.390

Compound Table

Compound Label	RT (min)	Observed mass (m/z)	Neutral observed mass (Da)	Theoretical mass (Da)	Mass error (ppm)	Isotope match score (%)
Cpd 1: C20 H22 N2 O6	0.76	409.1370	386.1481	386.1478	0.77	87.90

Mass errors of between -5.00 and 5.00 ppm with isotope match scores above 60% are considered confirmation of molecular formulae

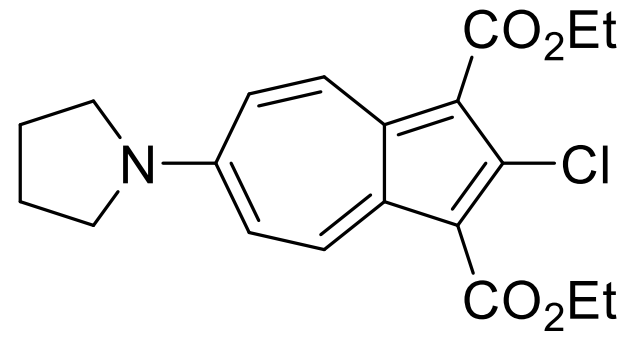

23

^{1}H-NMR, 400 MHz, $CDCl_3$

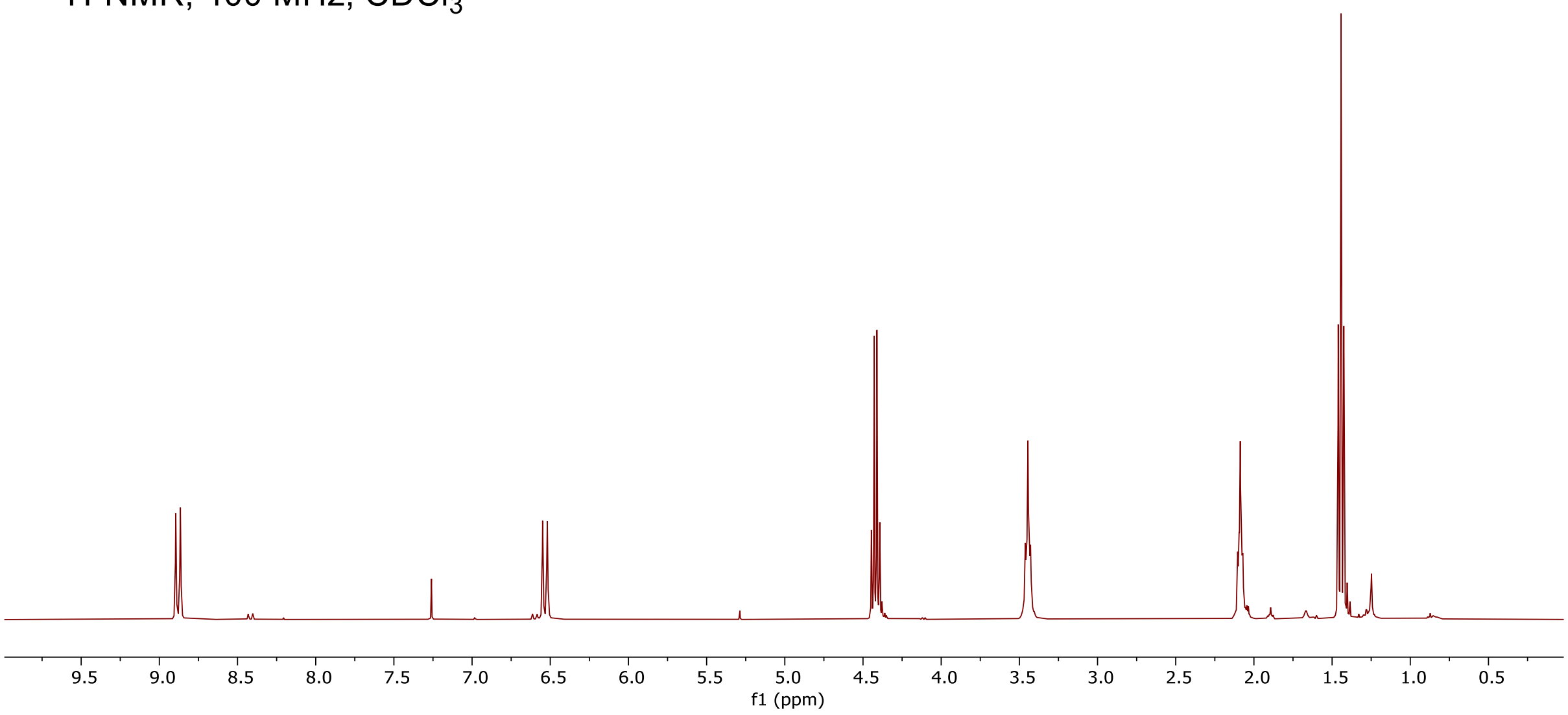

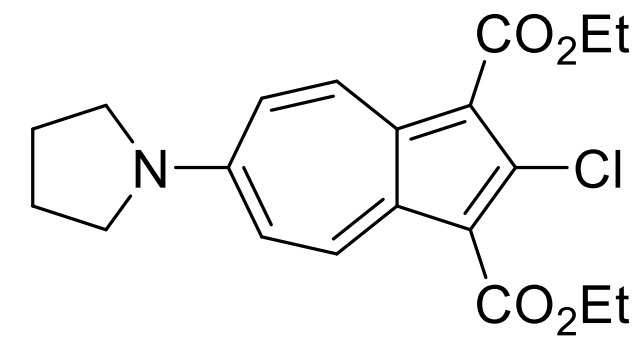

23

^{13}C-NMR, 101 MHz, $CDCl_3$

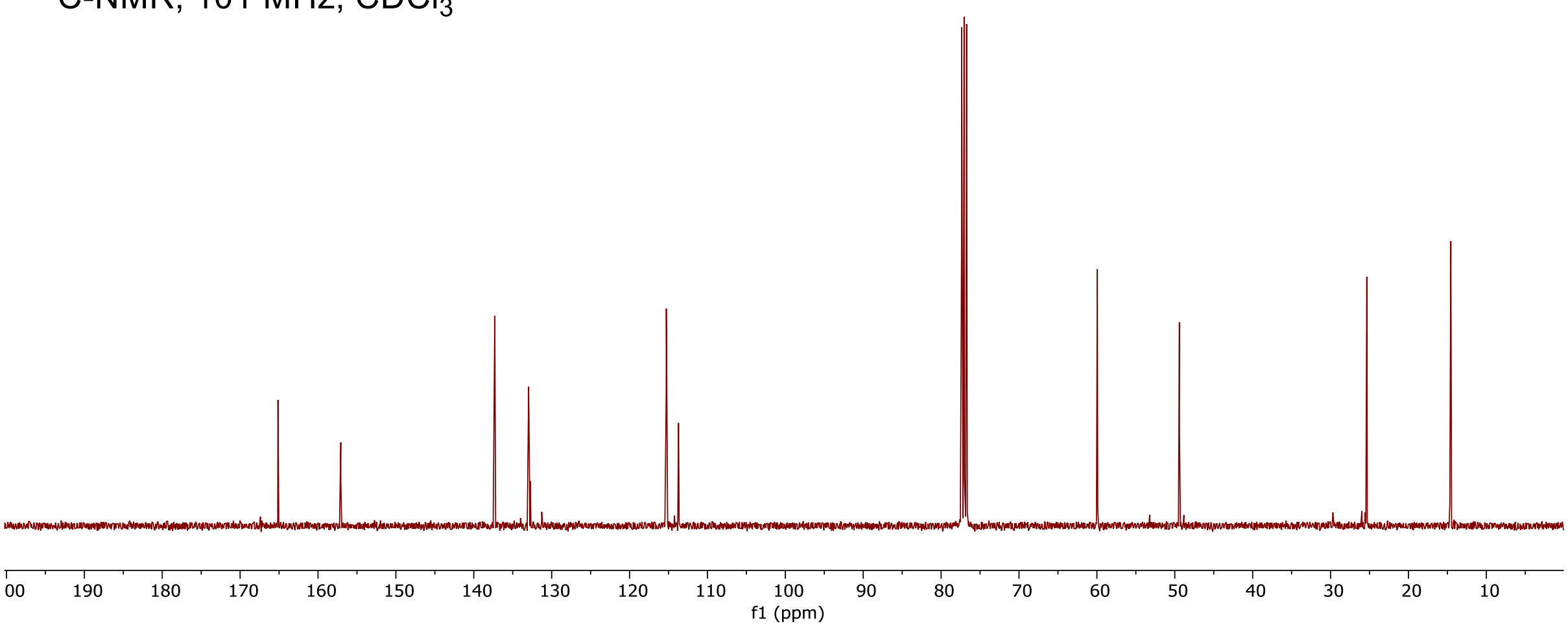

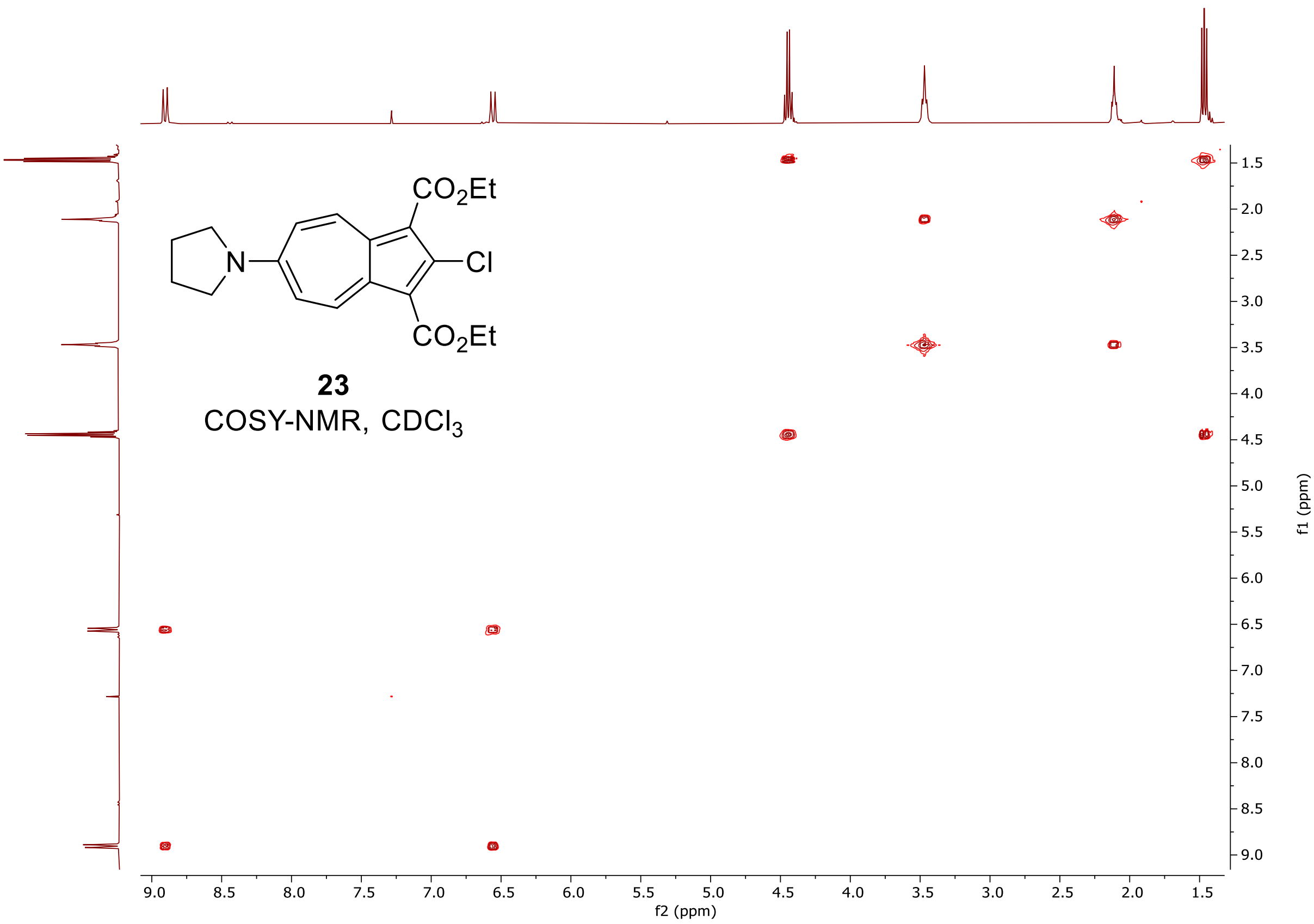
CO2Et
N
Cl
CO2Et
23
COSY-NMR, CDCl3
f2 (ppm)
f1 (ppm)

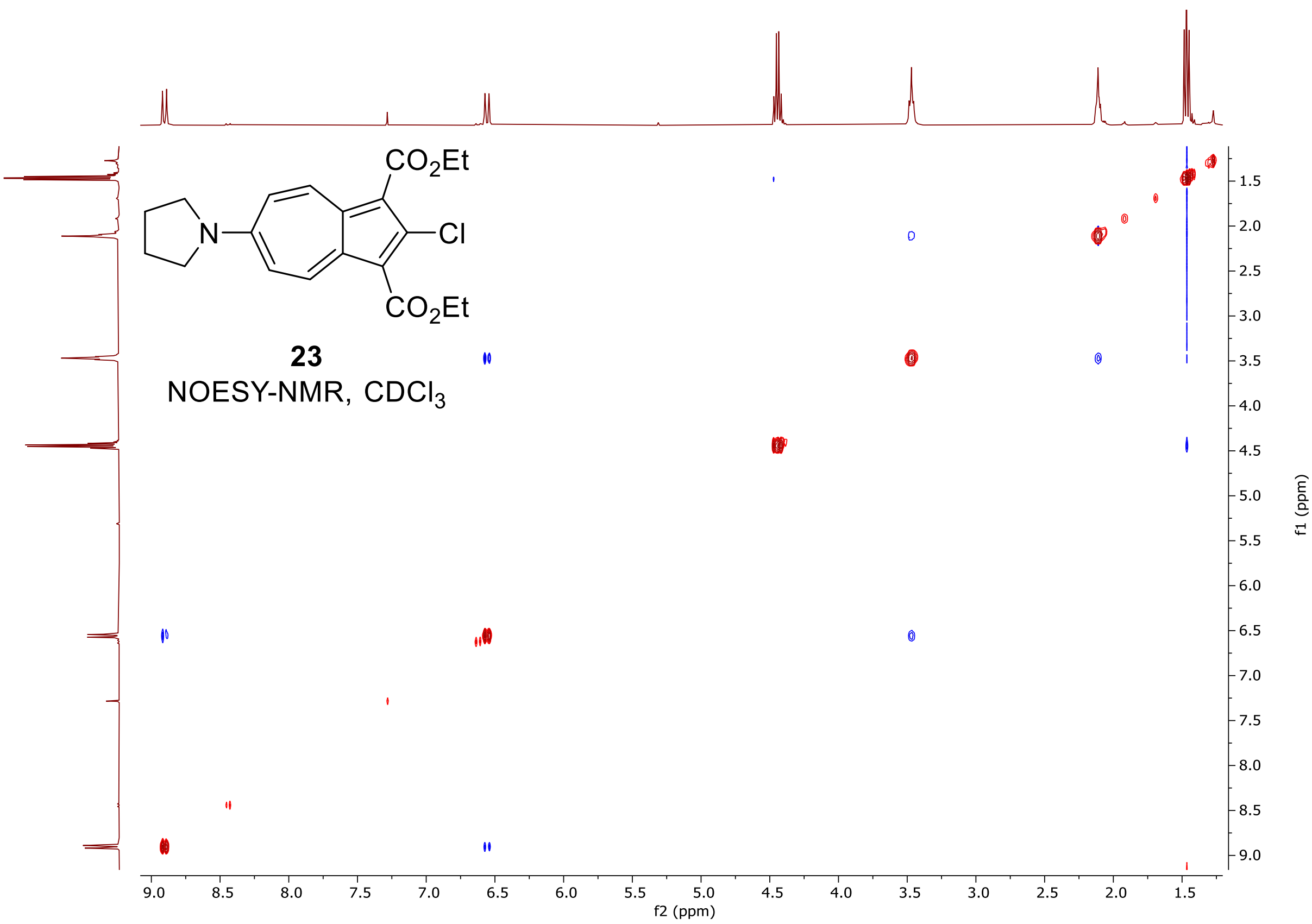
CO2Et
N
Cl
CO2Et
23
NOESY-NMR, CDCl3
f2 (ppm)
f1 (ppm)

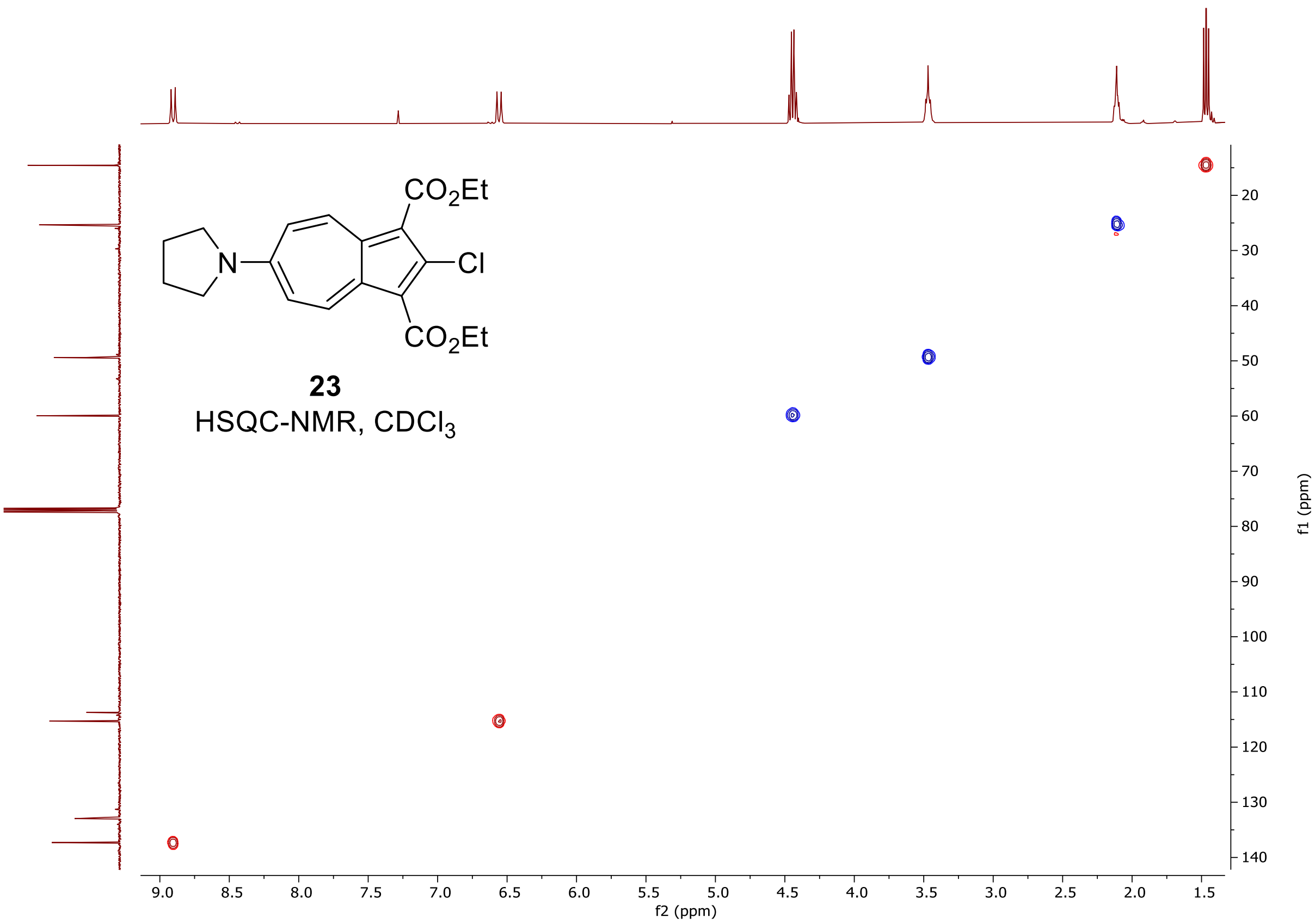
CO2Et
N
Cl
CO2Et
23
HSQC-NMR, CDCl3
f2 (ppm)
f1 (ppm)

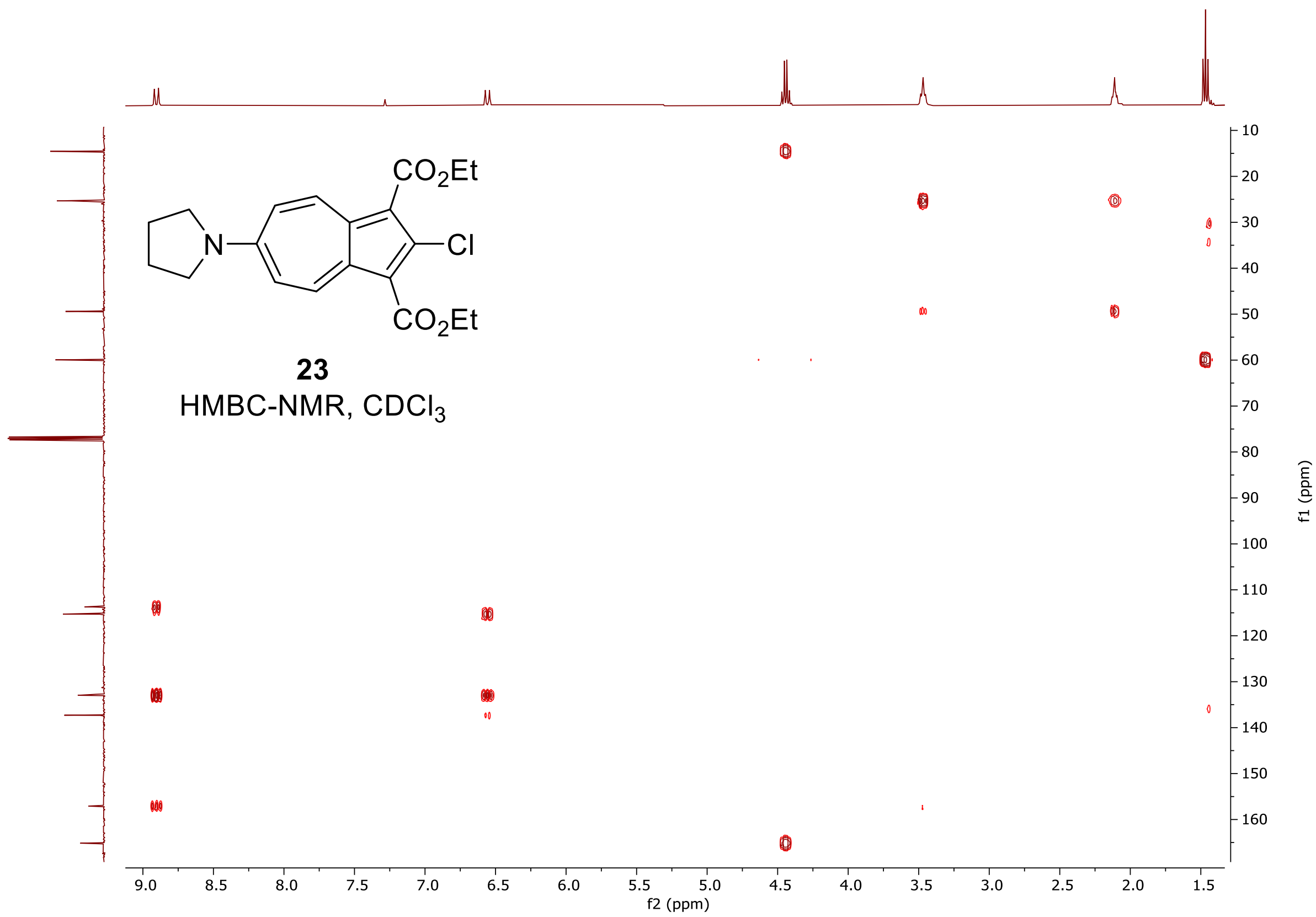
CO2Et
N
Cl
CO2Et
23
HMBC-NMR, CDCl3
f2 (ppm)
f1 (ppm)

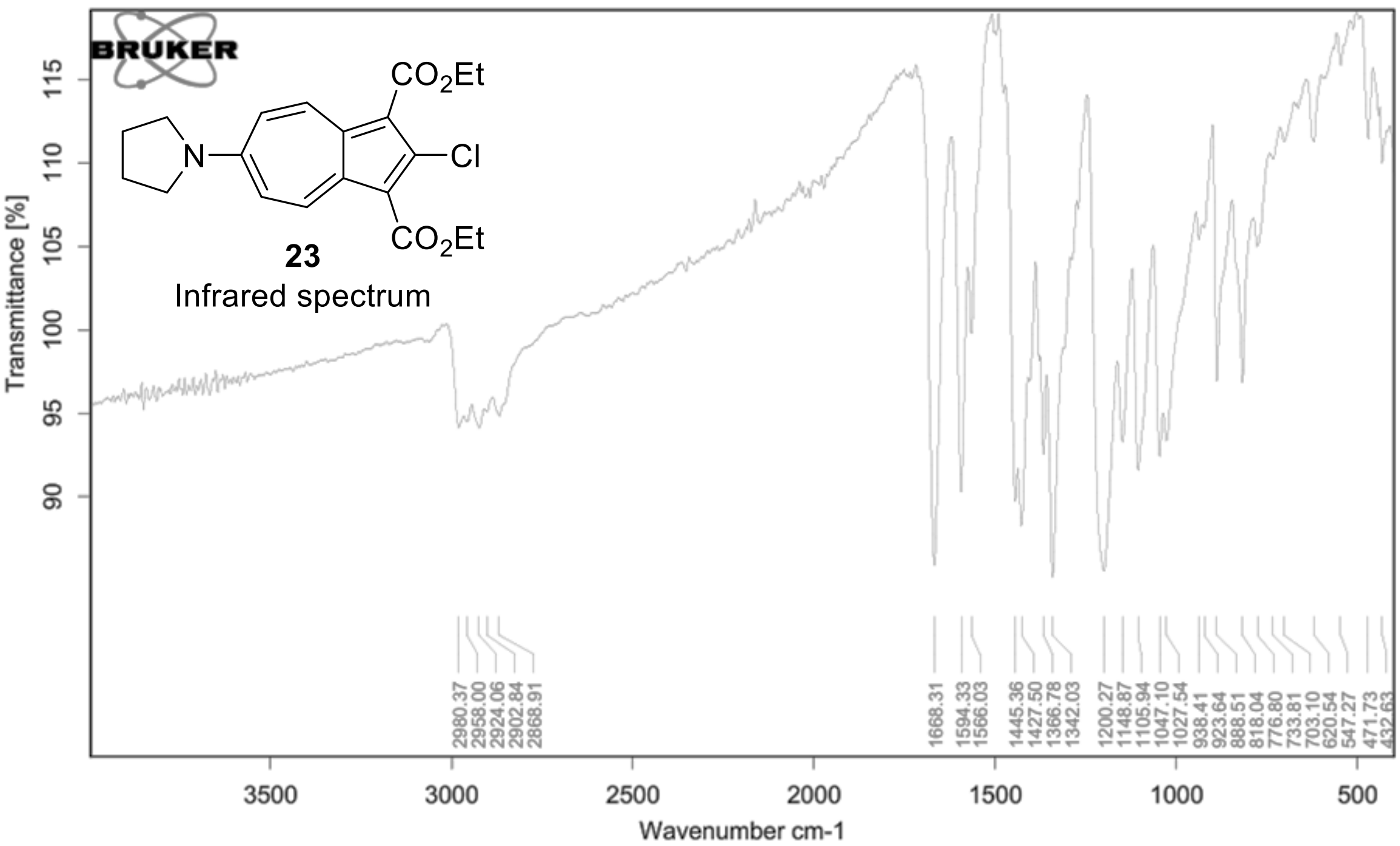

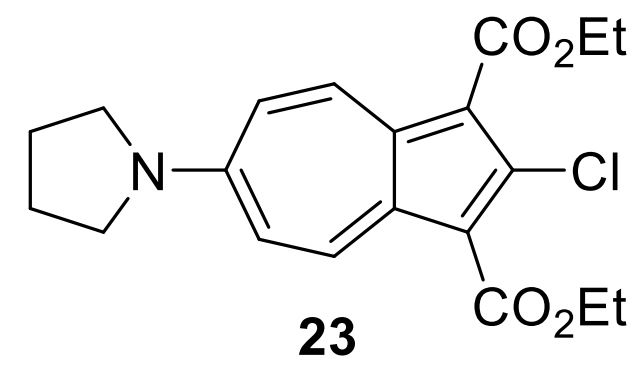

23

HRMS-ESI$^+$

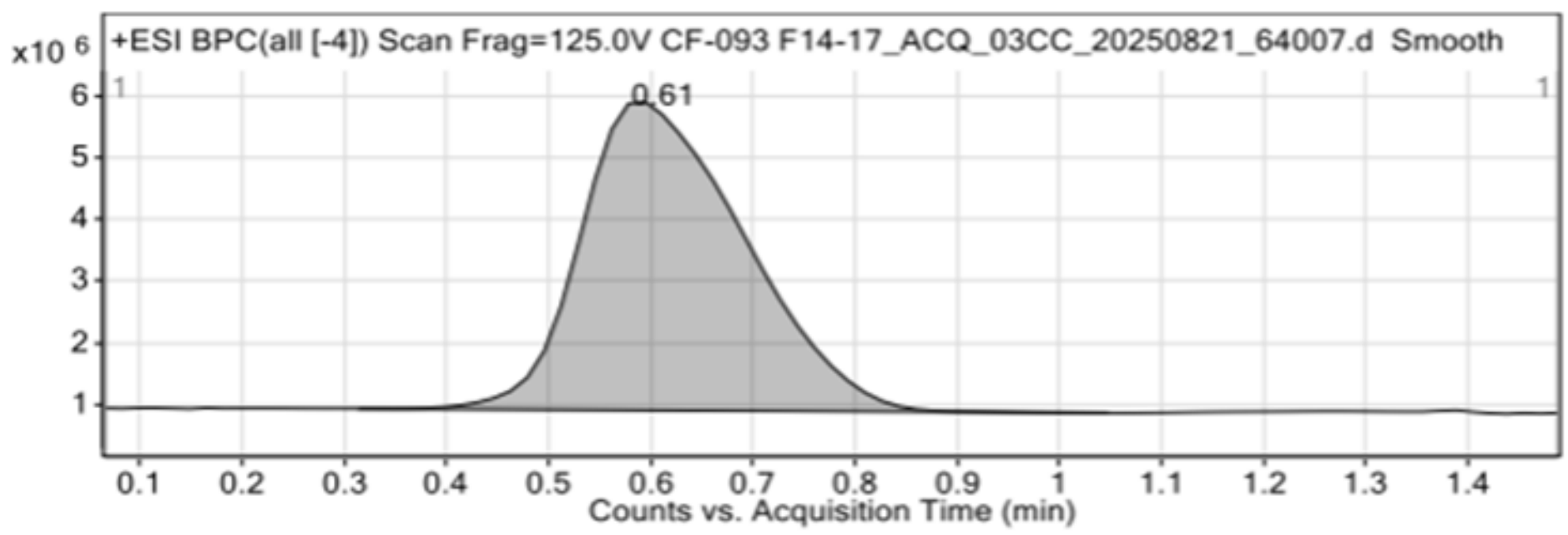

Figure 1: Base peak chromatogram

User Chromatogram Peak List

RT (min)	Area	Area %	Area Sum (%)	Base Peak (m/z)	Width (min)
0.61	55813153	100.00	100.00	376.1294	0.200

Compound Table

Compound Label	RT (min)	Observed mass (m/z)	Neutral observed mass (Da)	Theoretical mass (Da)	Mass error (ppm)	Isotope match score (%)
Cpd 1: C20 H22 Cl N O4	0.62	376.1308	375.1235	375.1237	-0.57	99.19

Mass errors of between -5.00 and 5.00 ppm with isotope match scores above 60% are considered confirmation of molecular formulae

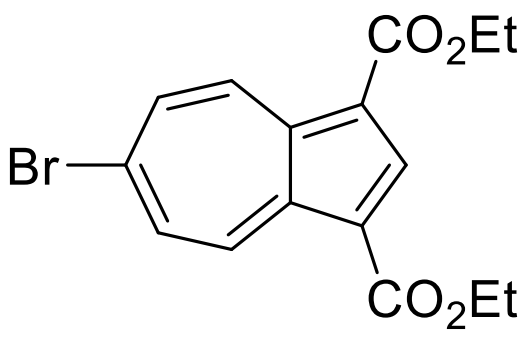

25

^{1}H-NMR, 400 MHz, $CDCl_3$

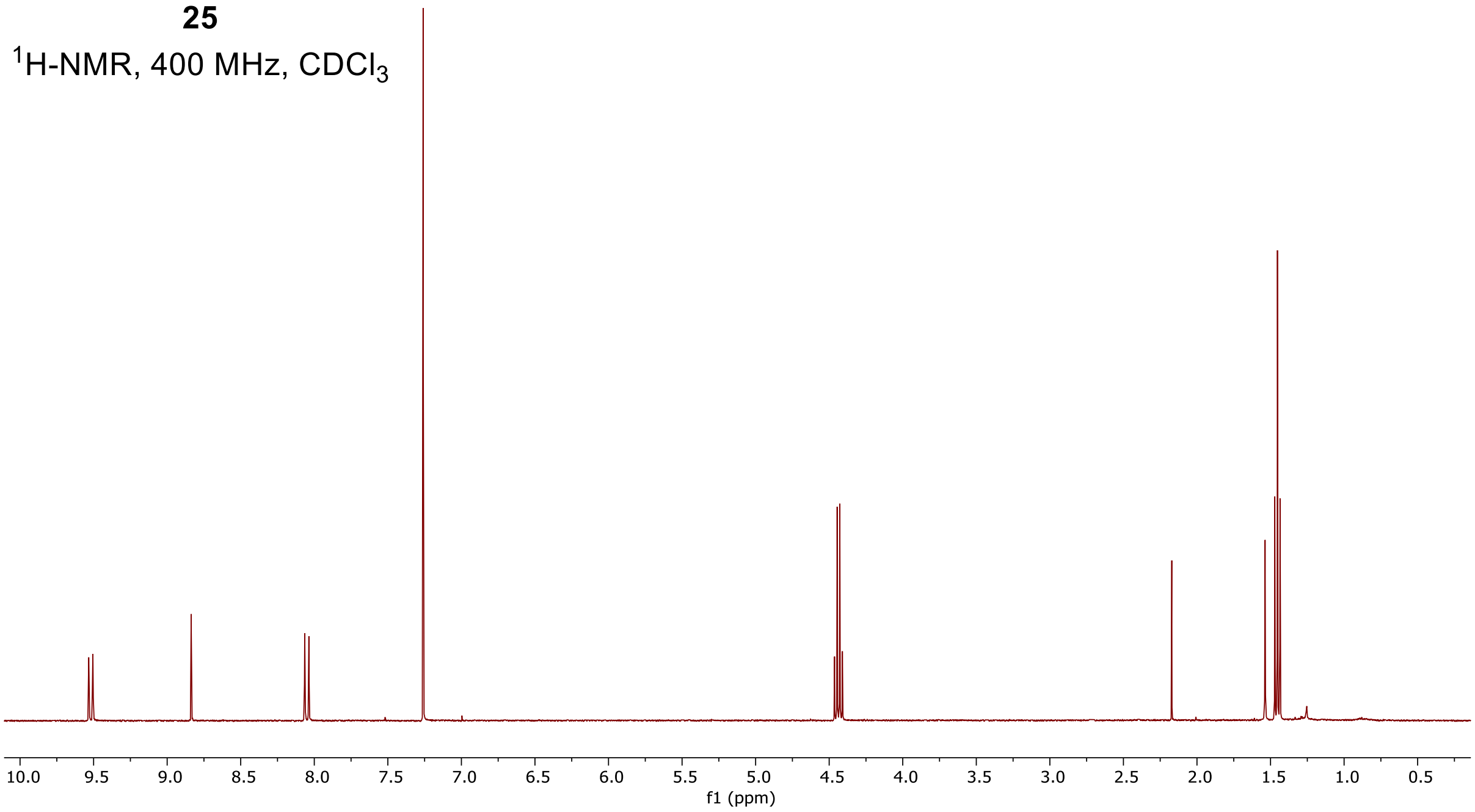

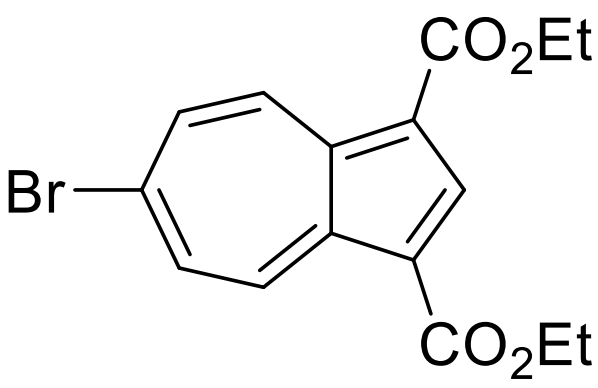

25

^{13}C-NMR, 101 MHz, $CDCl_3$

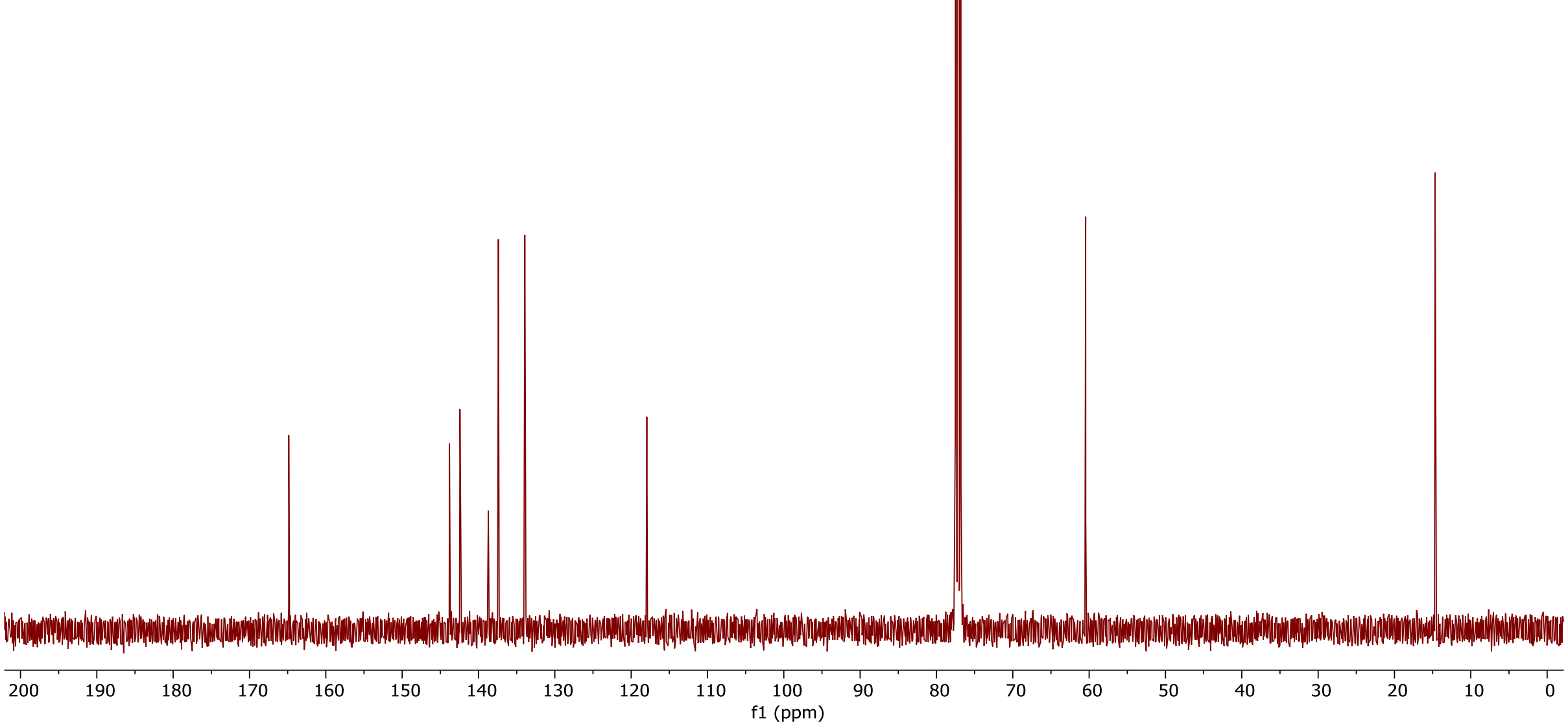

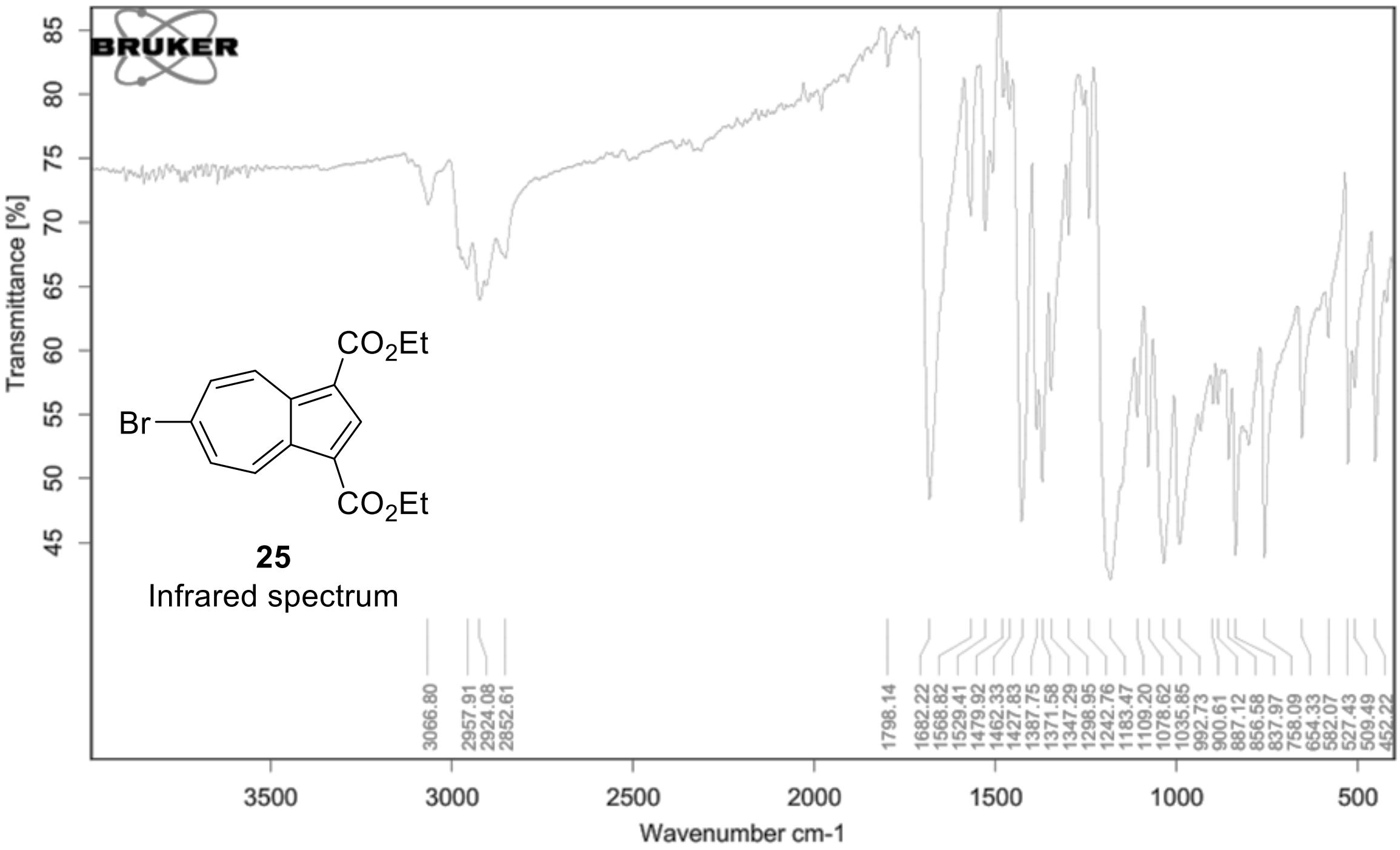

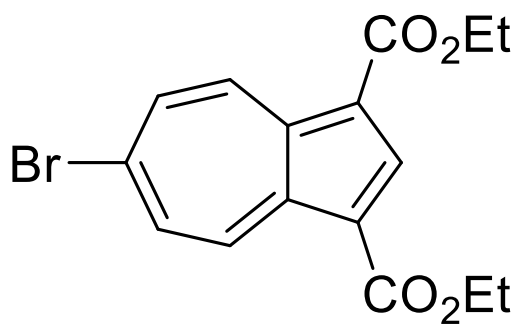

25

Mass spectrum

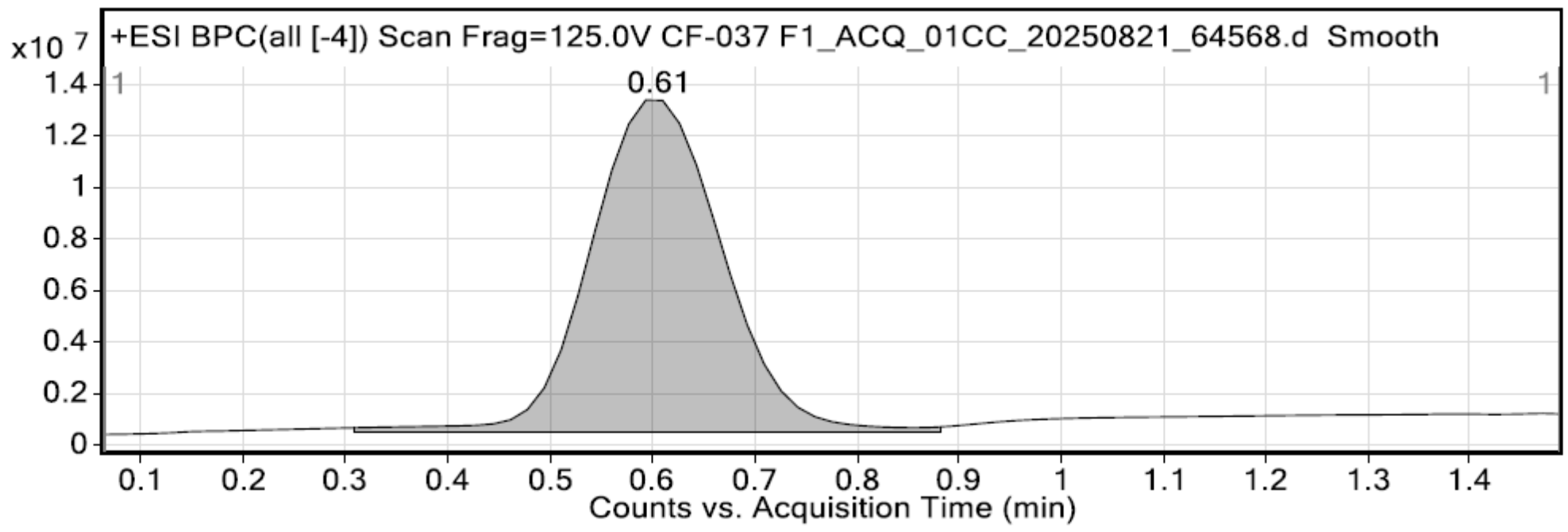

Figure 1: Base peak chromatogram

User Chromatogram Peak List

RT (min)	Area	Area %	Area Sum (%)	Base Peak (m/z)	Width (min)
0.61	116238426	100.00	100.00	122.0821	0.170

Compound Table

Compound Label	RT (min)	Observed mass (m/z)	Neutral observed mass (Da)	Theoretical mass (Da)	Mass error (ppm)	Isotope match score (%)
Cpd 1: C16 H15 Br O4	0.96	351.0224	350.0151	350.0154	-0.81	99.79

Mass errors of between -5.00 and 5.00 ppm with isotope match scores above 60% are considered confirmation of molecular formulae

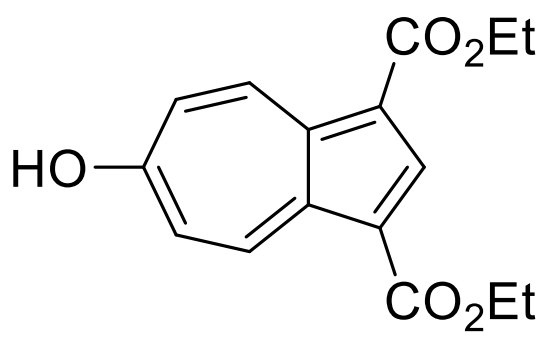

26

^{1}H-NMR, 400 MHz, acetone-d_6

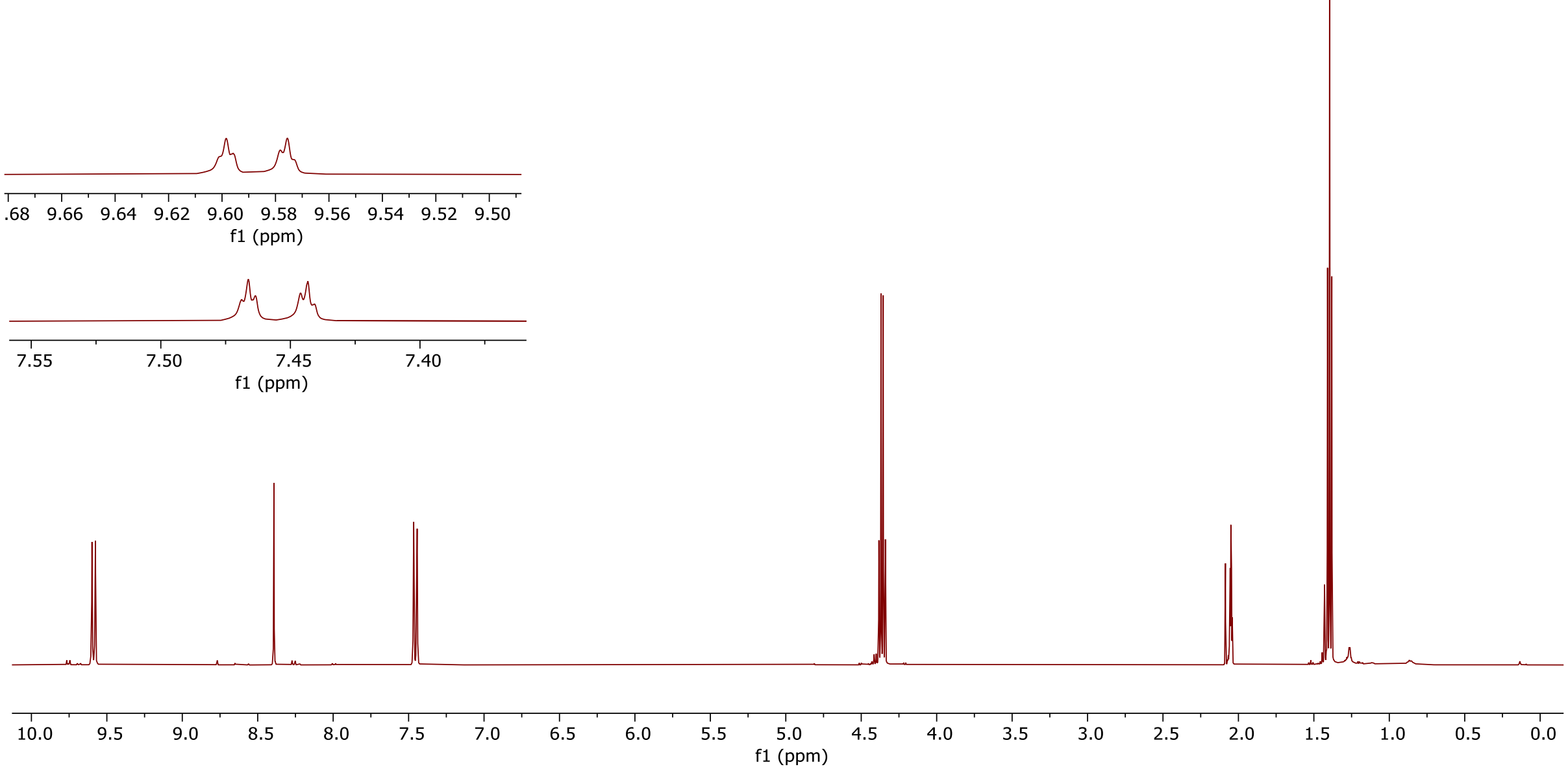

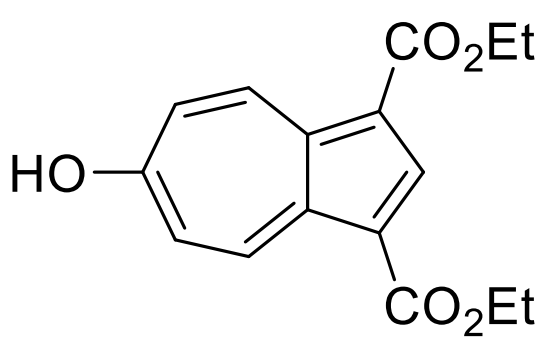

26

^{13}C-NMR, 126 MHz, acetone-d_6

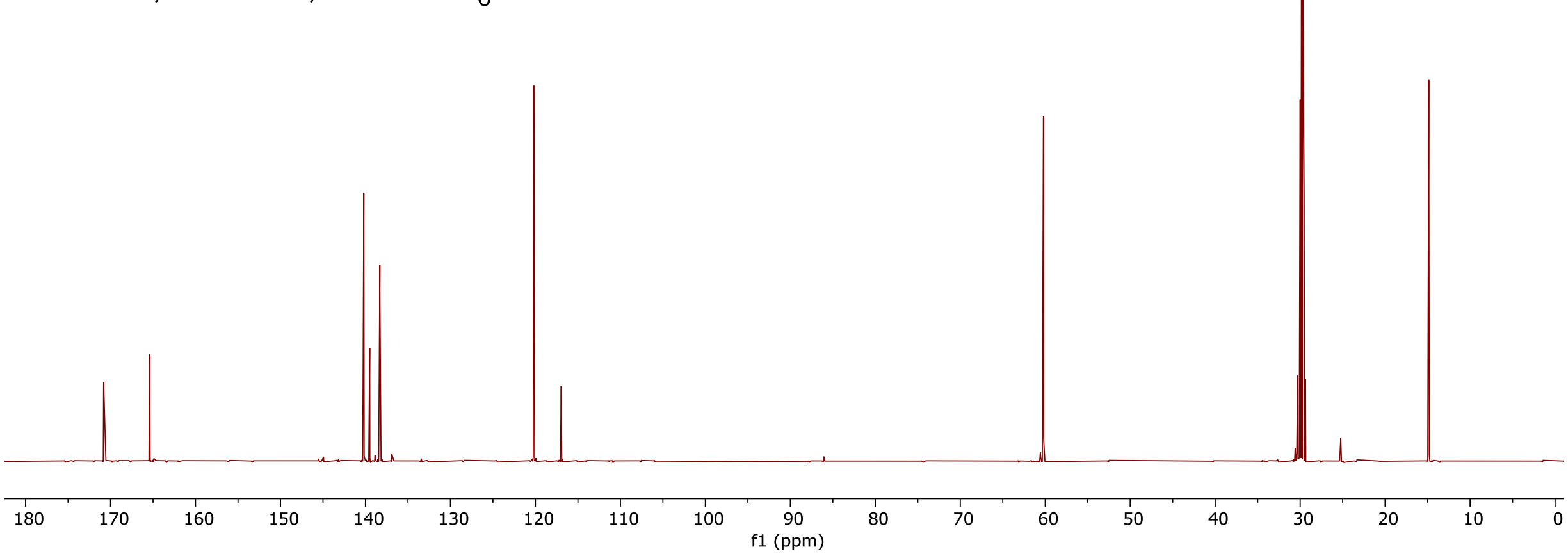

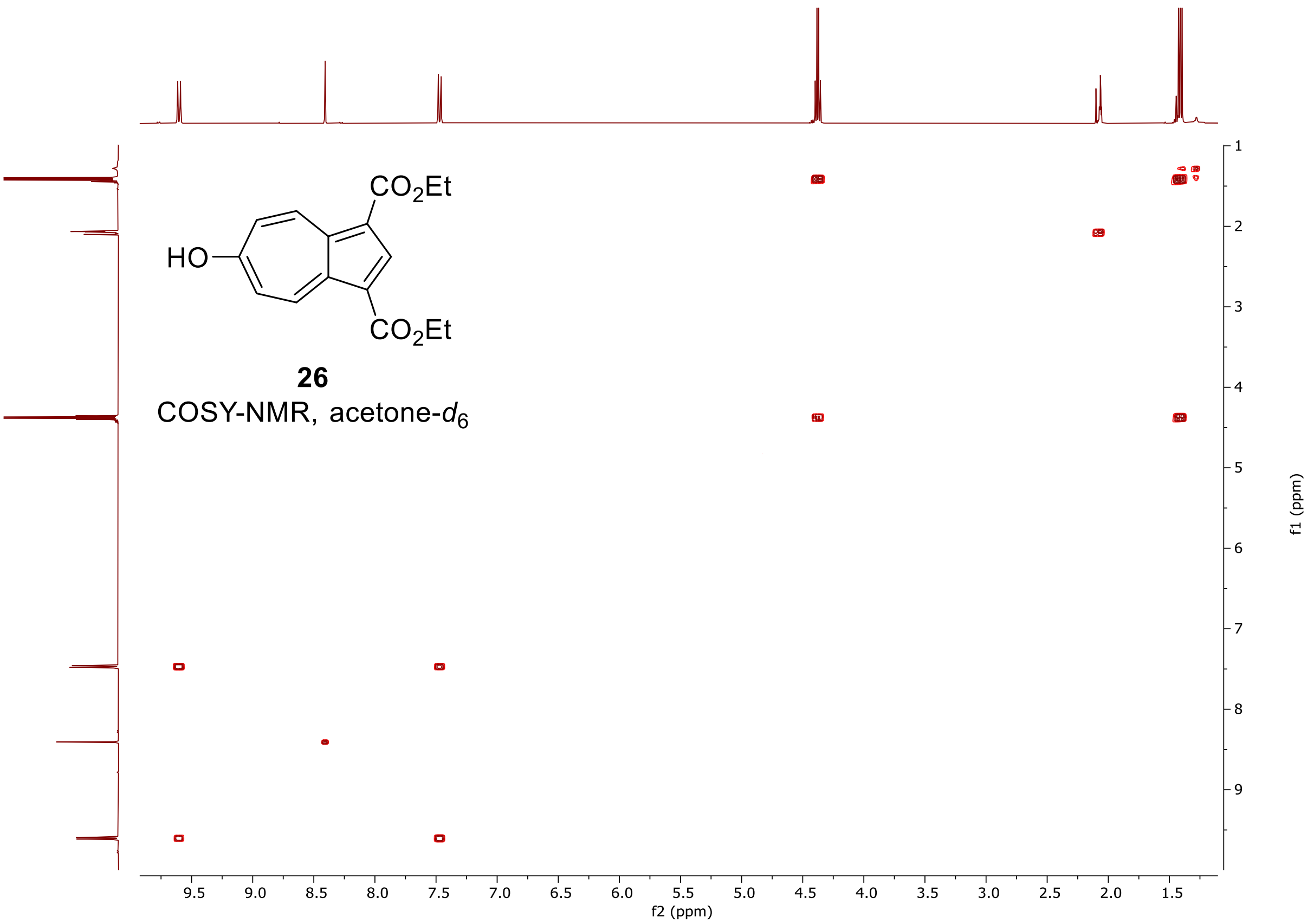
CO2Et
HO
CO2Et
26
COSY-NMR, acetone-d6
f2 (ppm)
f1 (ppm)

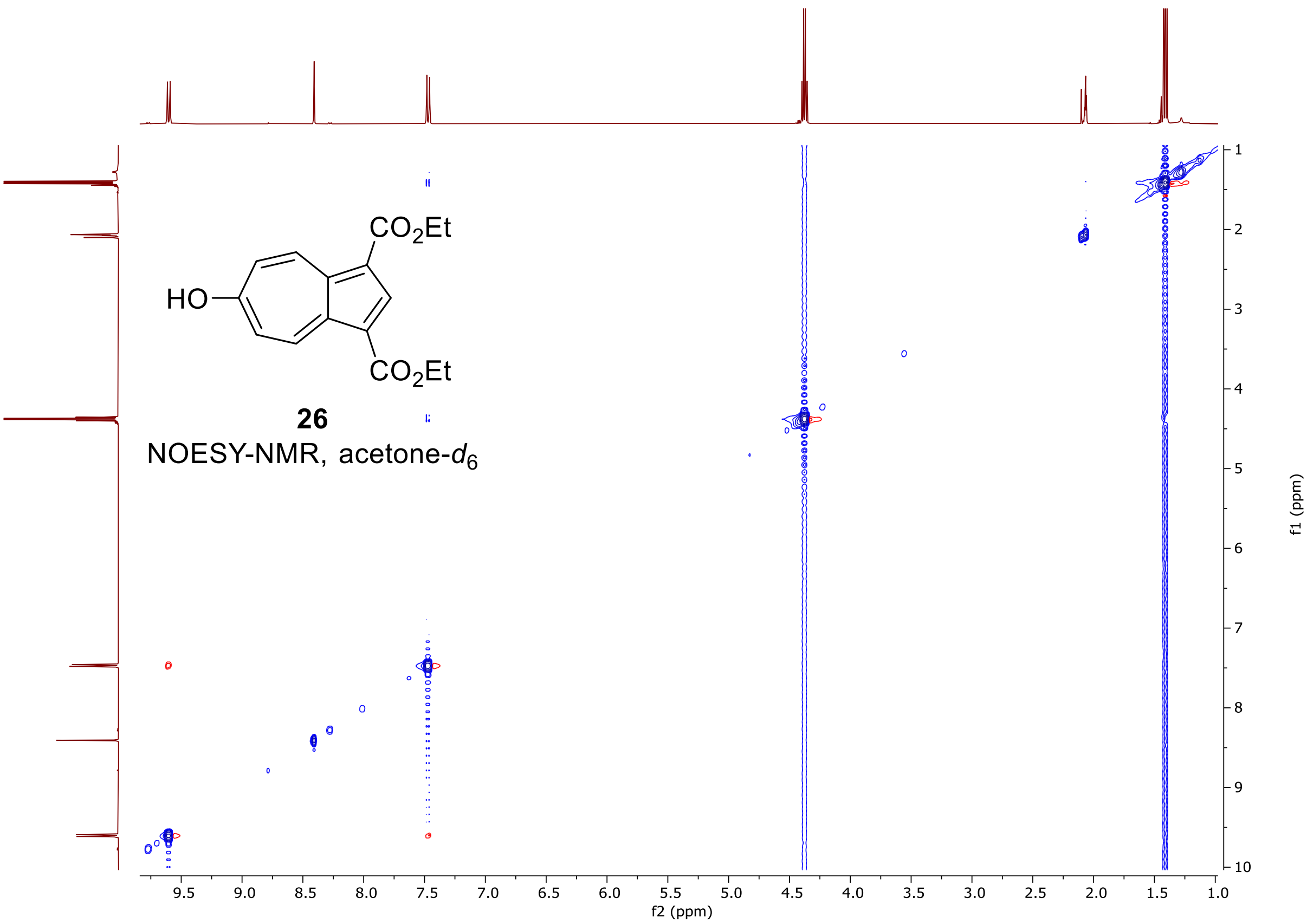
CO2Et
HO
CO2Et
26
NOESY-NMR, acetone-d6
f2 (ppm)
f1 (ppm)

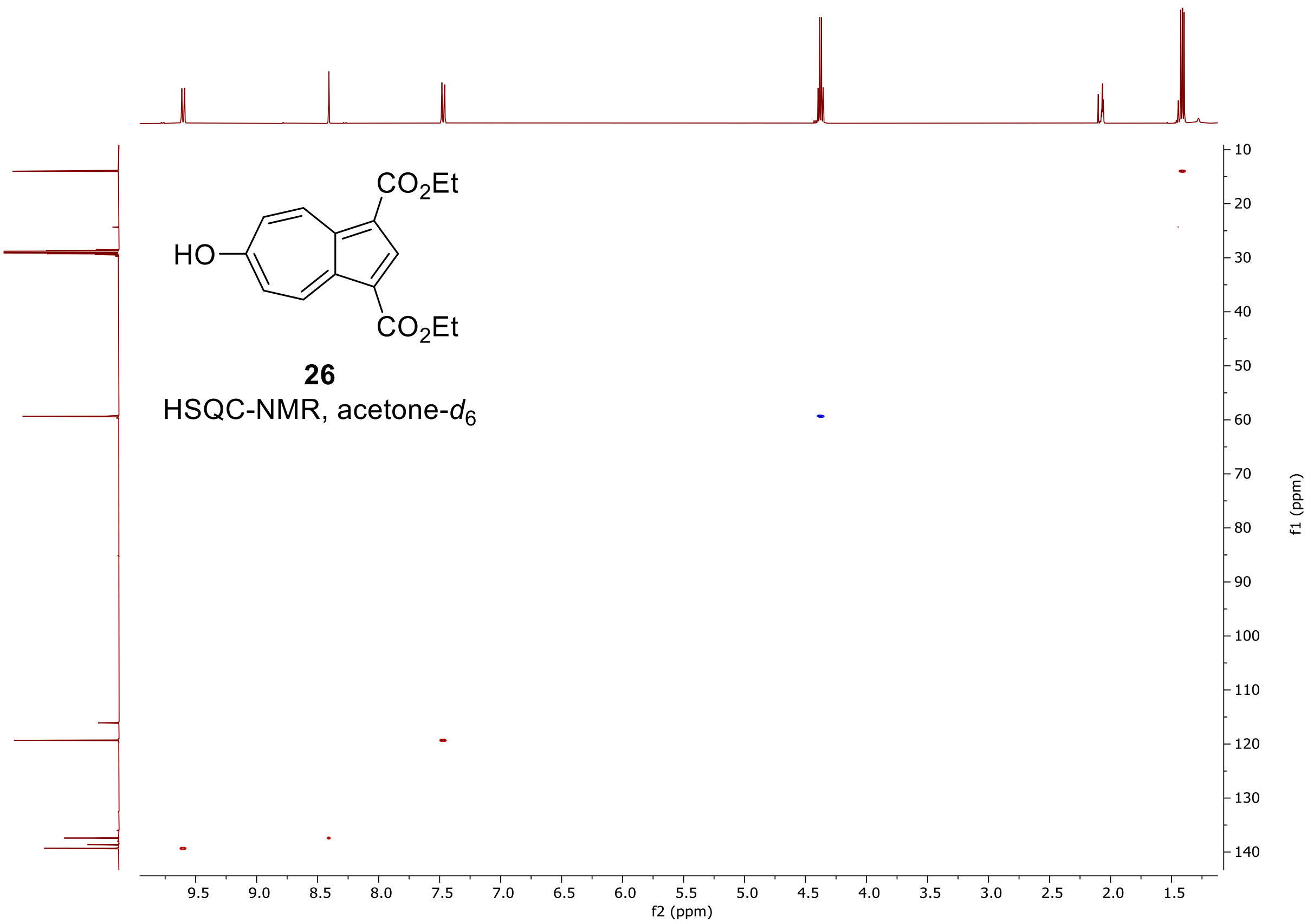
CO2Et
HO
CO2Et
26
HSQC-NMR, acetone-d6
f1 (ppm)
f2 (ppm)

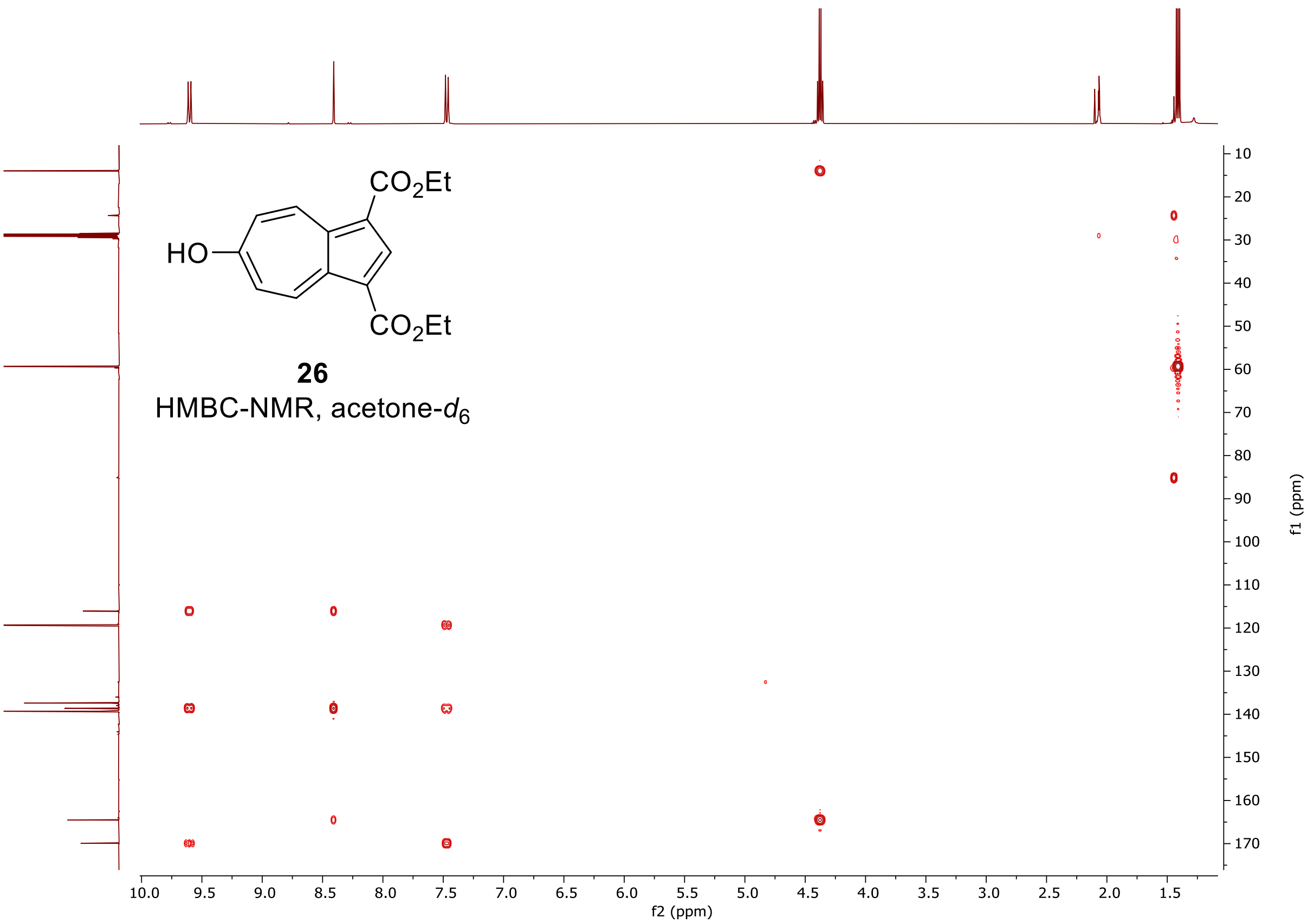
CO2Et
HO
CO2Et
26
HMBC-NMR, acetone-d6
f1 (ppm)
f2 (ppm)

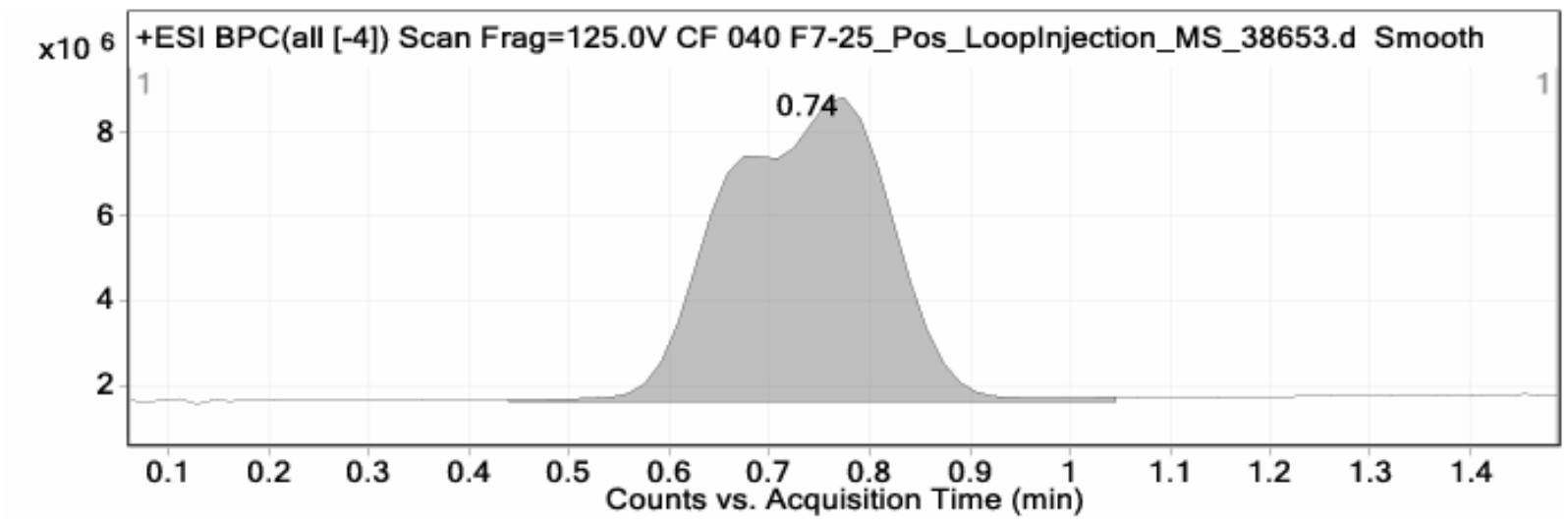

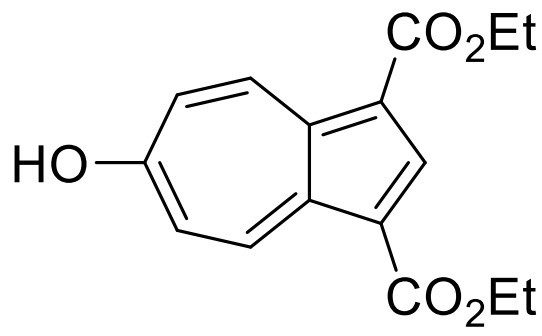

26
HRMS-ESI$^+$

Figure 1: Base peak chromatogram

User Chromatogram Peak List

RT (min)	Area	Area %	Area Sum (%)	Base Peak (m/z)	Width (min)
0.74	84025534	100.00	100.00	289.1060	0.200

Compound Table

Compound Label	RT (min)	Observed mass (m/z)	Neutral observed mass (Da)	Theoretical mass (Da)	Mass error (ppm)	Isotope match score (%)	Error flag
Cpd 1: C22 H27 B O6	0.73	421.1813	397.1980	397.1937	10.82	10.49	m/z tolerance
Cpd 2: C16 H16 O5	0.74	289.1069	288.0997	288.0998	-0.15	98.10	

Mass errors of between -5.00 and 5.00 ppm with isotope match scores above 60% are considered confirmation of molecular formulae

Walkup Analysis Report

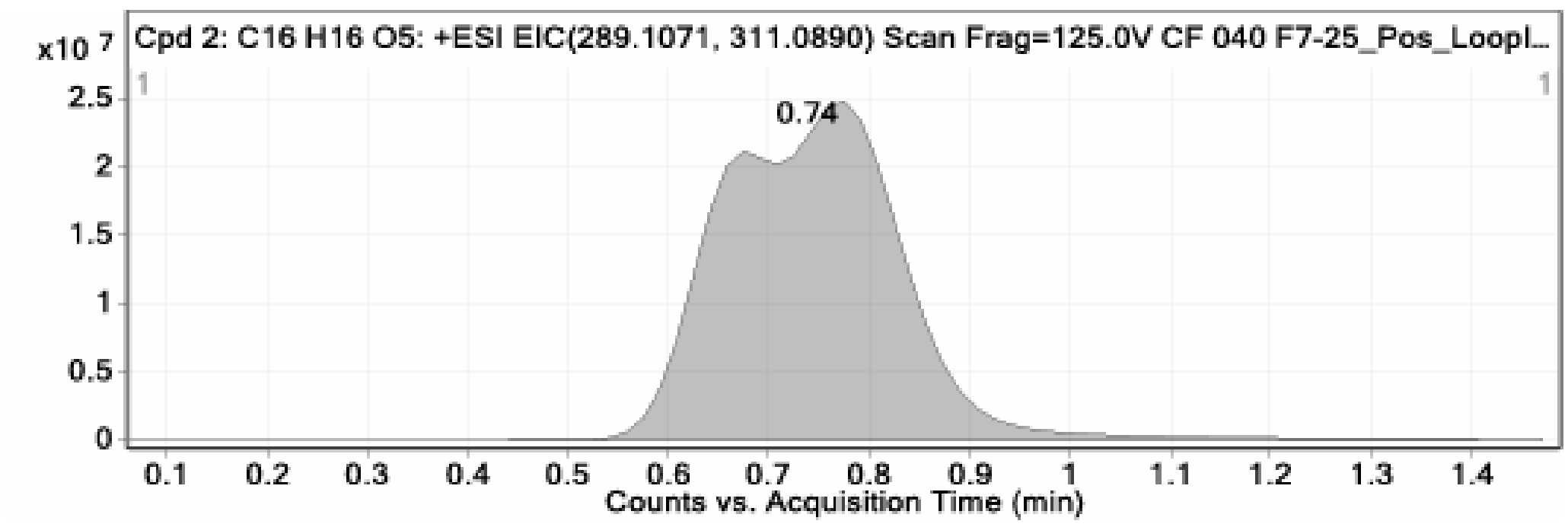

Integration Peak List

Start	RT	End	Height	Area
0.44	0.74	1.47	23199115	316838119

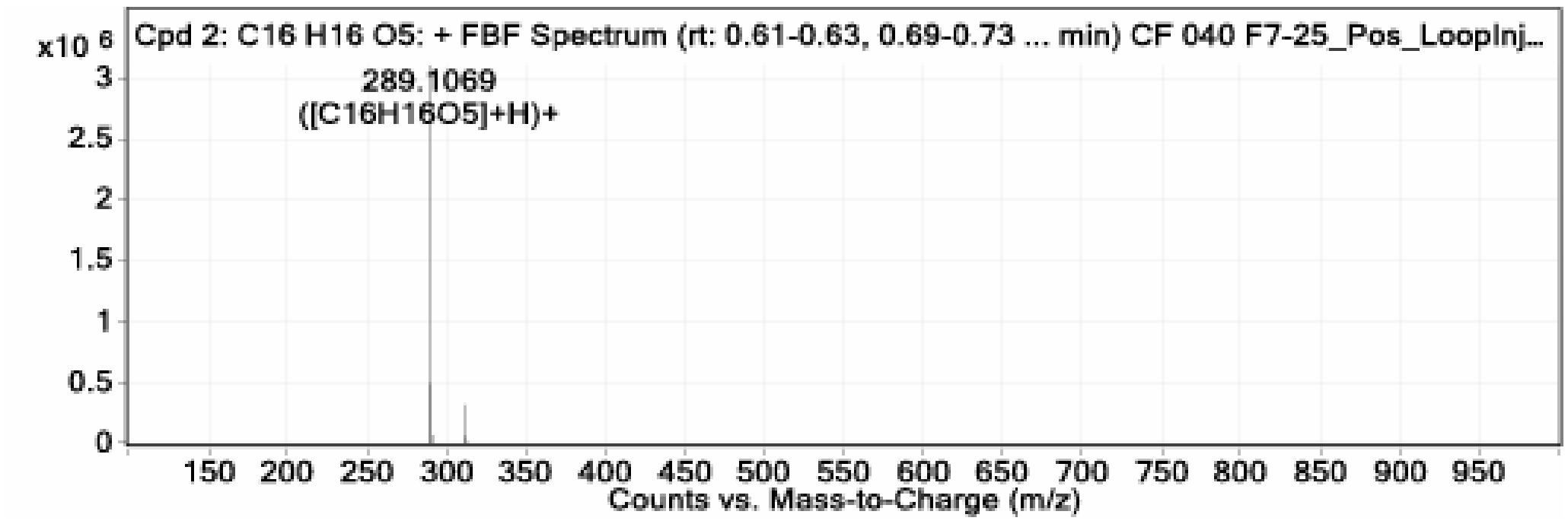

Peak List

m/z	z	Abund	Formula	Ion
289.1069	1	3110931.5	C16H16O5	(M+H)+
290.1106	1	488827.88	C16H16O5	(M+H)+
291.1132	1	67346.34	C16H16O5	(M+H)+
311.0892	1	312293.97	C16H16O5	(M+Na)+
312.0925	1	59007.09	C16H16O5	(M+Na)+
313.0984	1	9217.45	C16H16O5	(M+Na)+

--- End Of Report ---

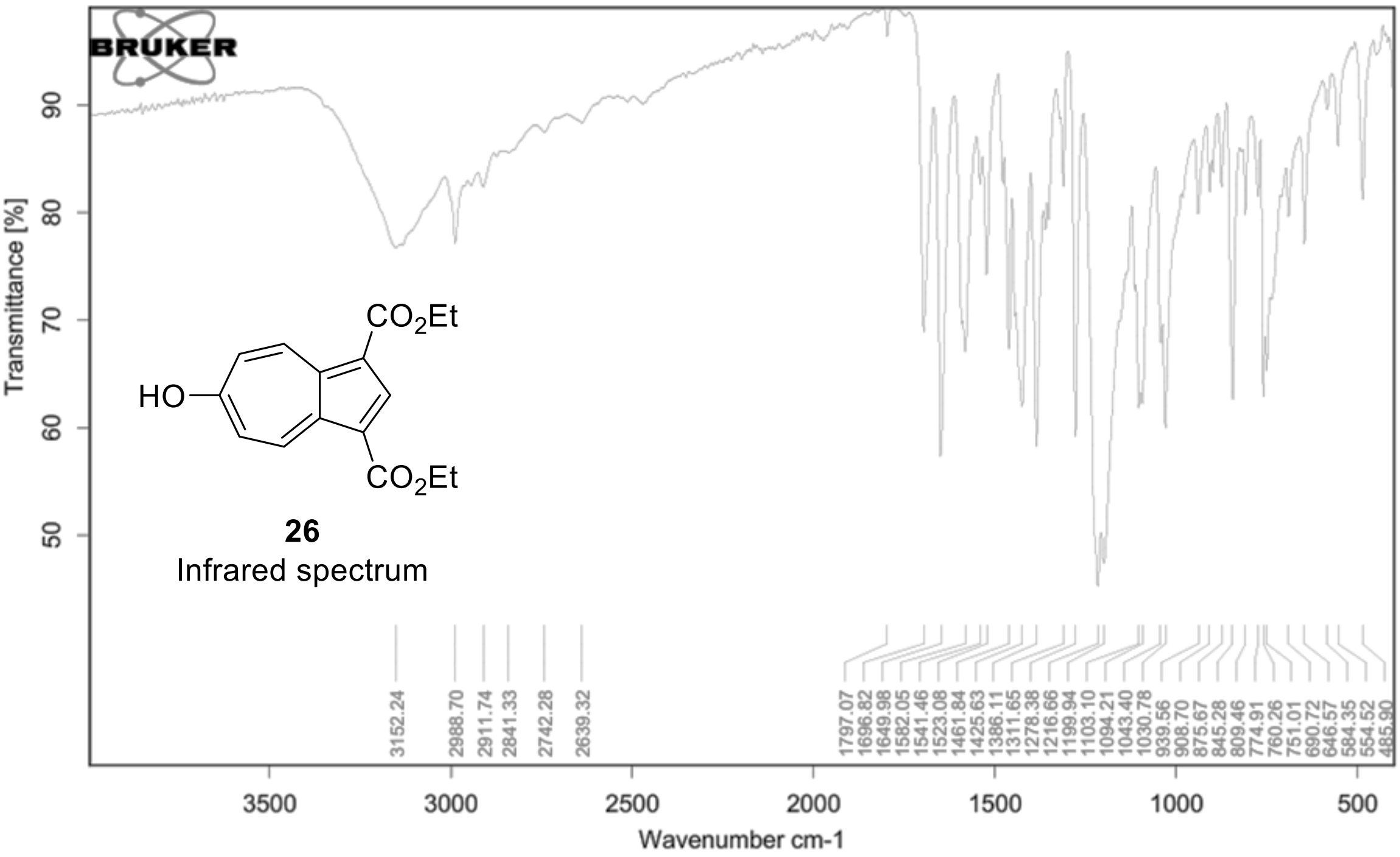
BRUKER
Transmittance [%]
Wavenumber cm-1
3500
3000
2500
2000
1500
1000
500
90
80
70
60
50
HO
CO2Et
CO2Et
26
Infrared spectrum
3152.24
2988.70
2911.74
2841.33
2742.28
2639.32
1797.07
1696.82
1649.98
1582.05
1541.46
1523.08
1461.84
1425.63
1386.11
1311.65
1278.38
1216.66
1199.94
1103.10
1094.21
1043.40
1030.78
939.56
908.70
875.67
845.28
809.46
774.91
760.26
751.01
690.72
646.57
584.35
554.52
485.90

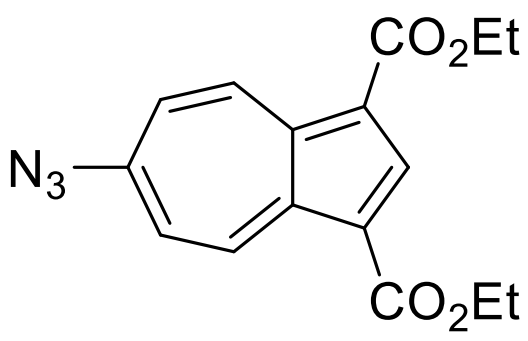

27

^{1}H-NMR, 400 MHz, $CDCl_3$

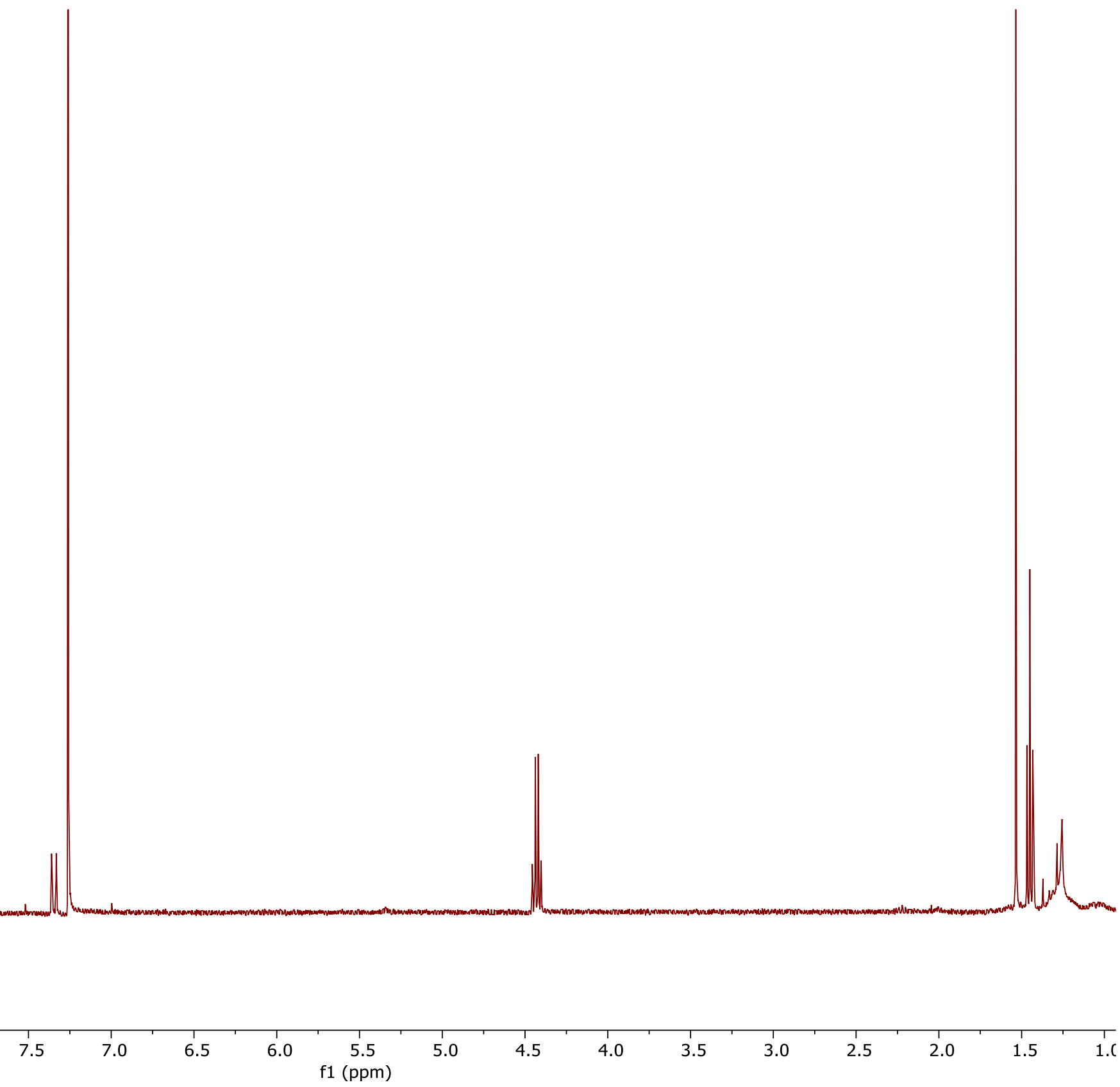

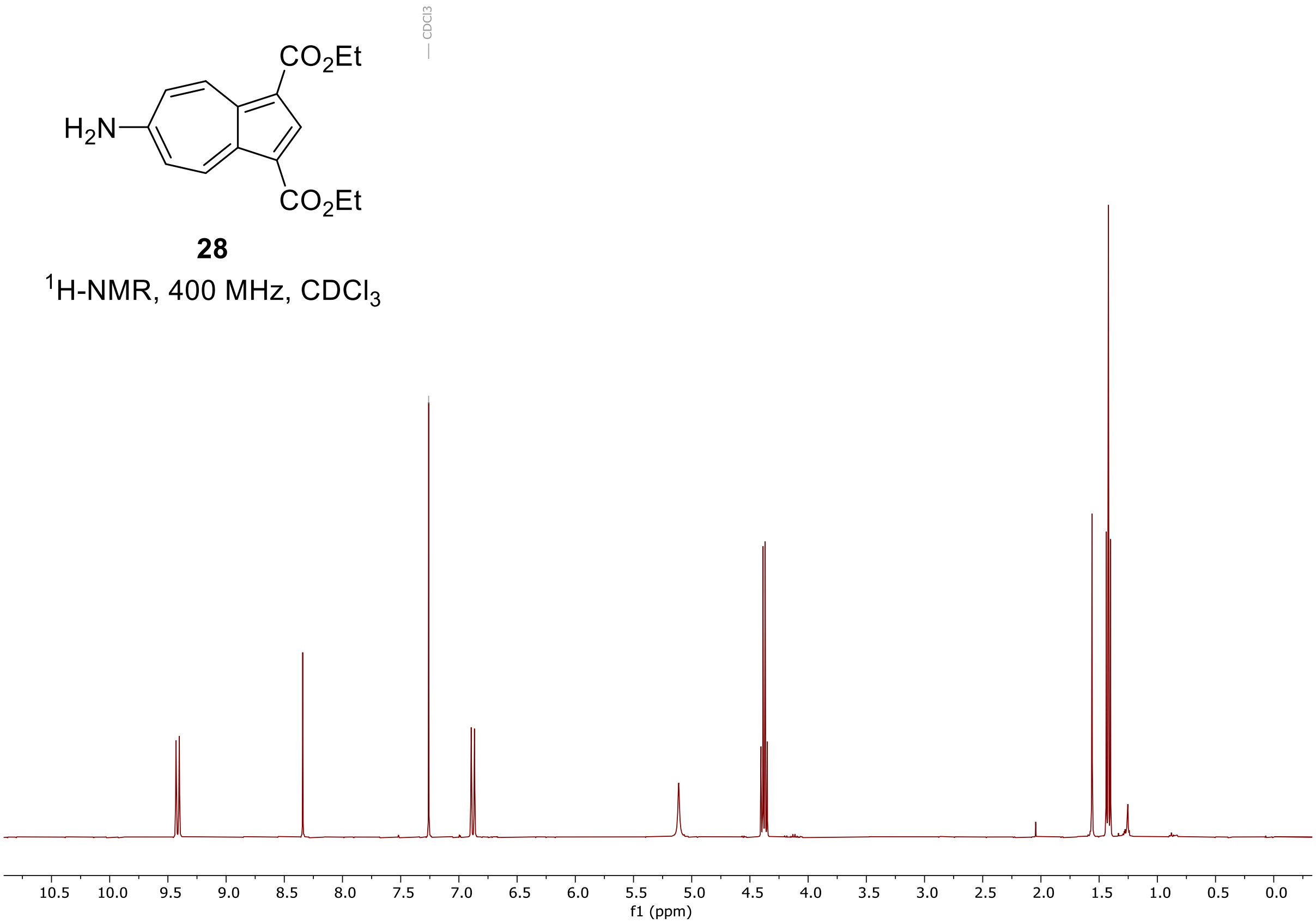

28
^1H-NMR, 400 MHz, $CDCl_3$

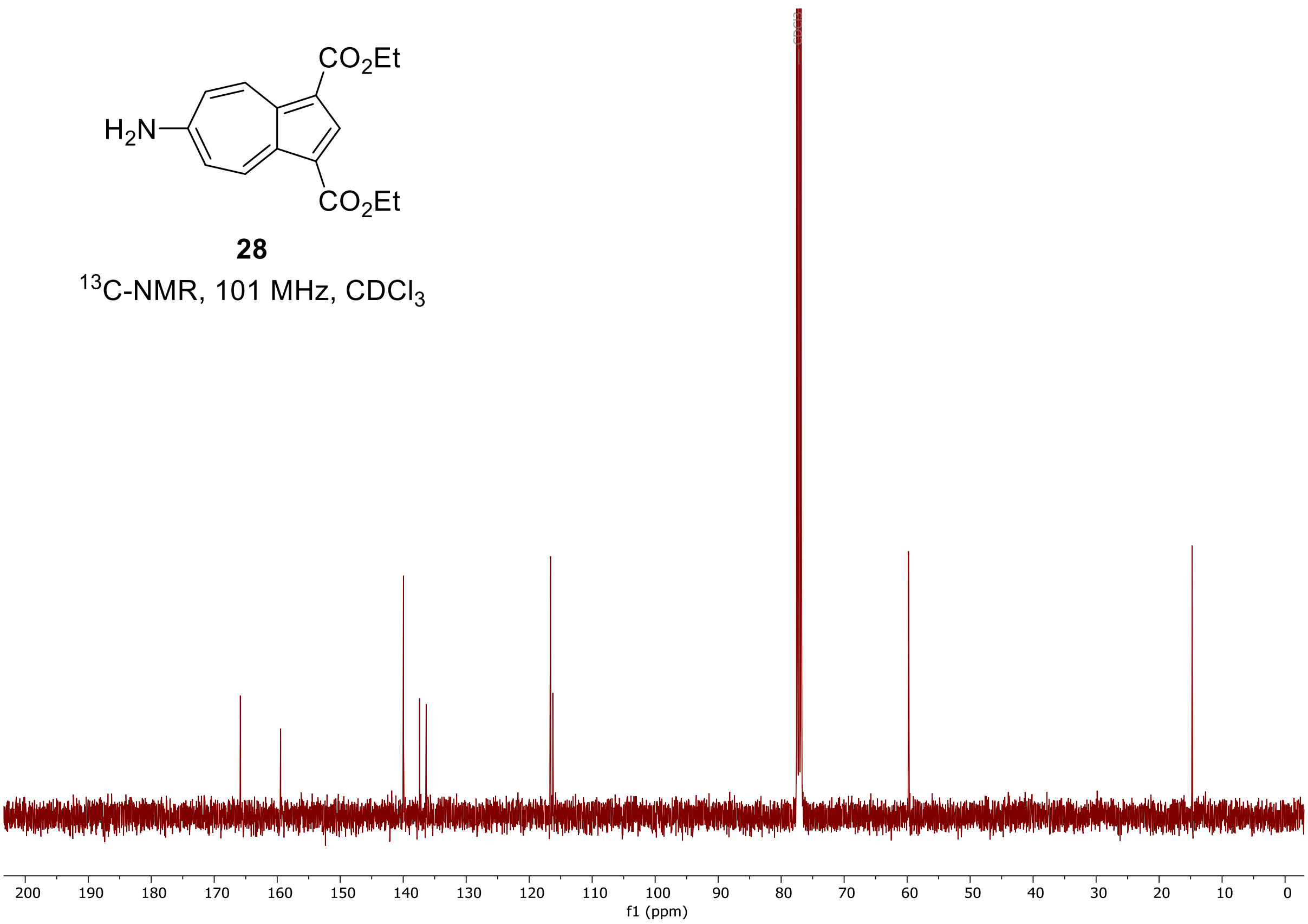

28
^{13}C-NMR, 101 MHz, $CDCl_3$

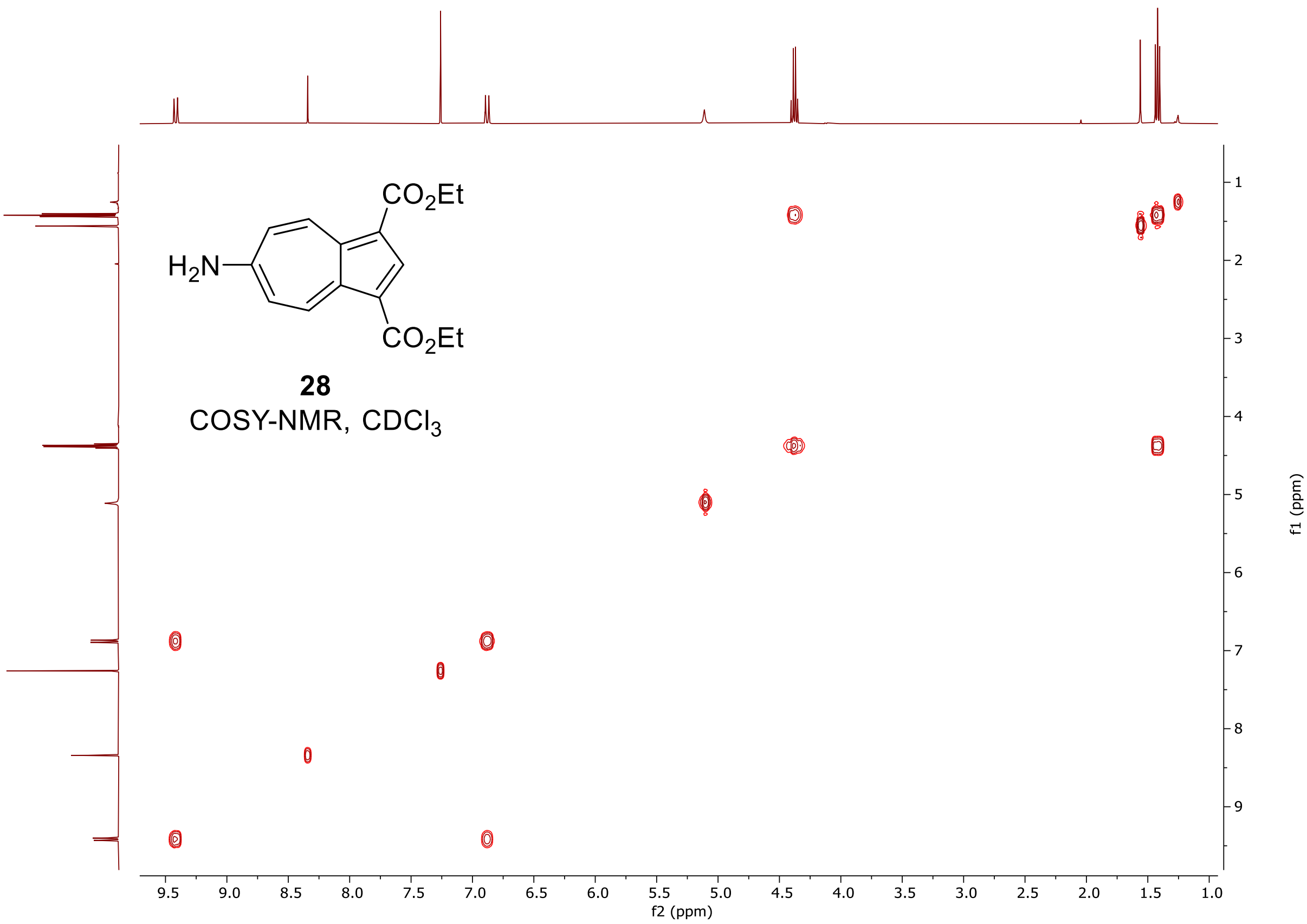
CO2Et
H2N
CO2Et
28
COSY-NMR, CDCl3
f2 (ppm)
f1 (ppm)

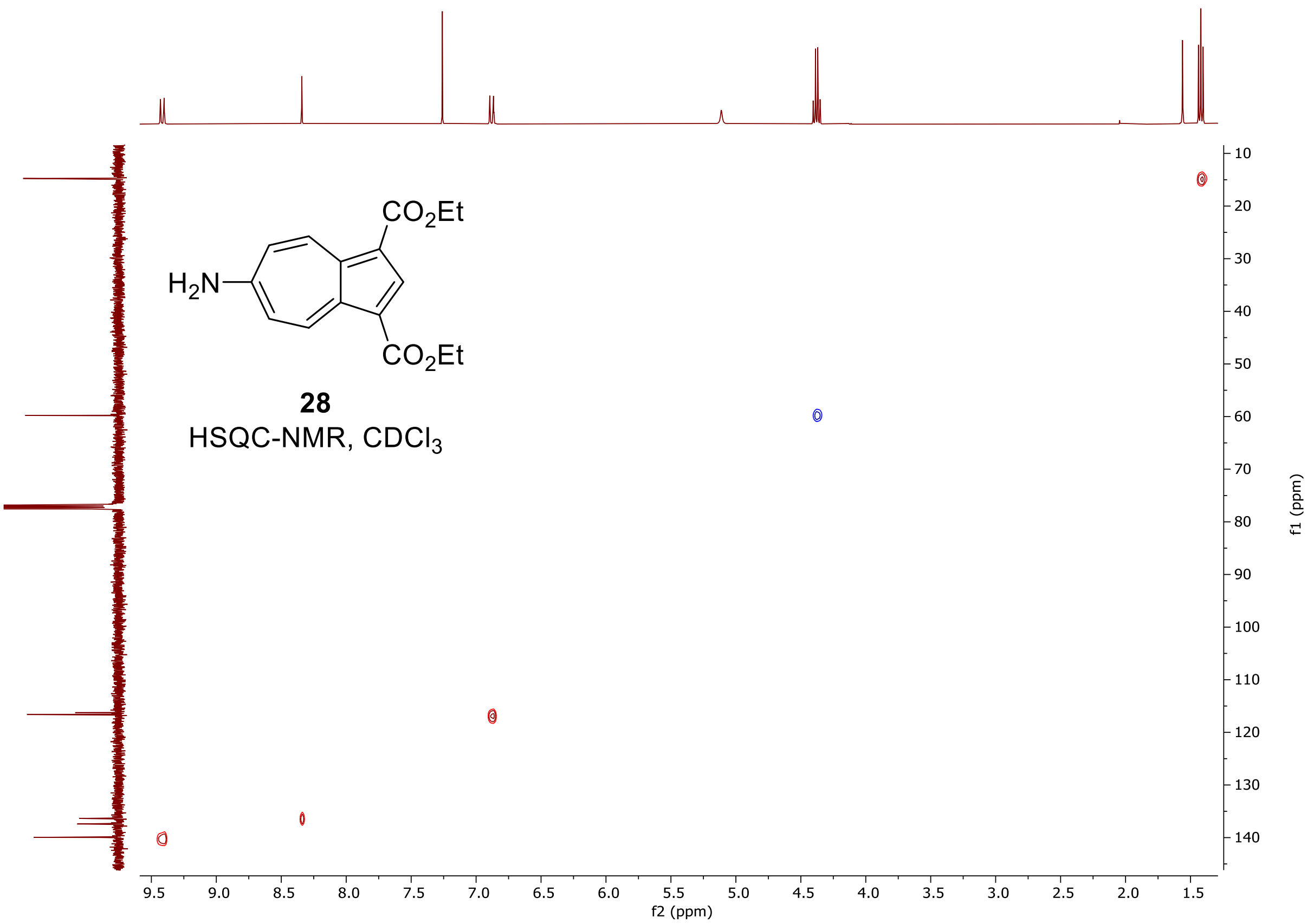
CO2Et
H2N
CO2Et
28
HSQC-NMR, CDCl3
f2 (ppm)
f1 (ppm)

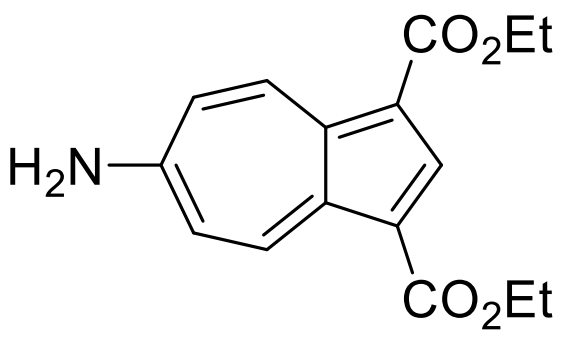

28

HMBC-NMR, $CDCl_3$

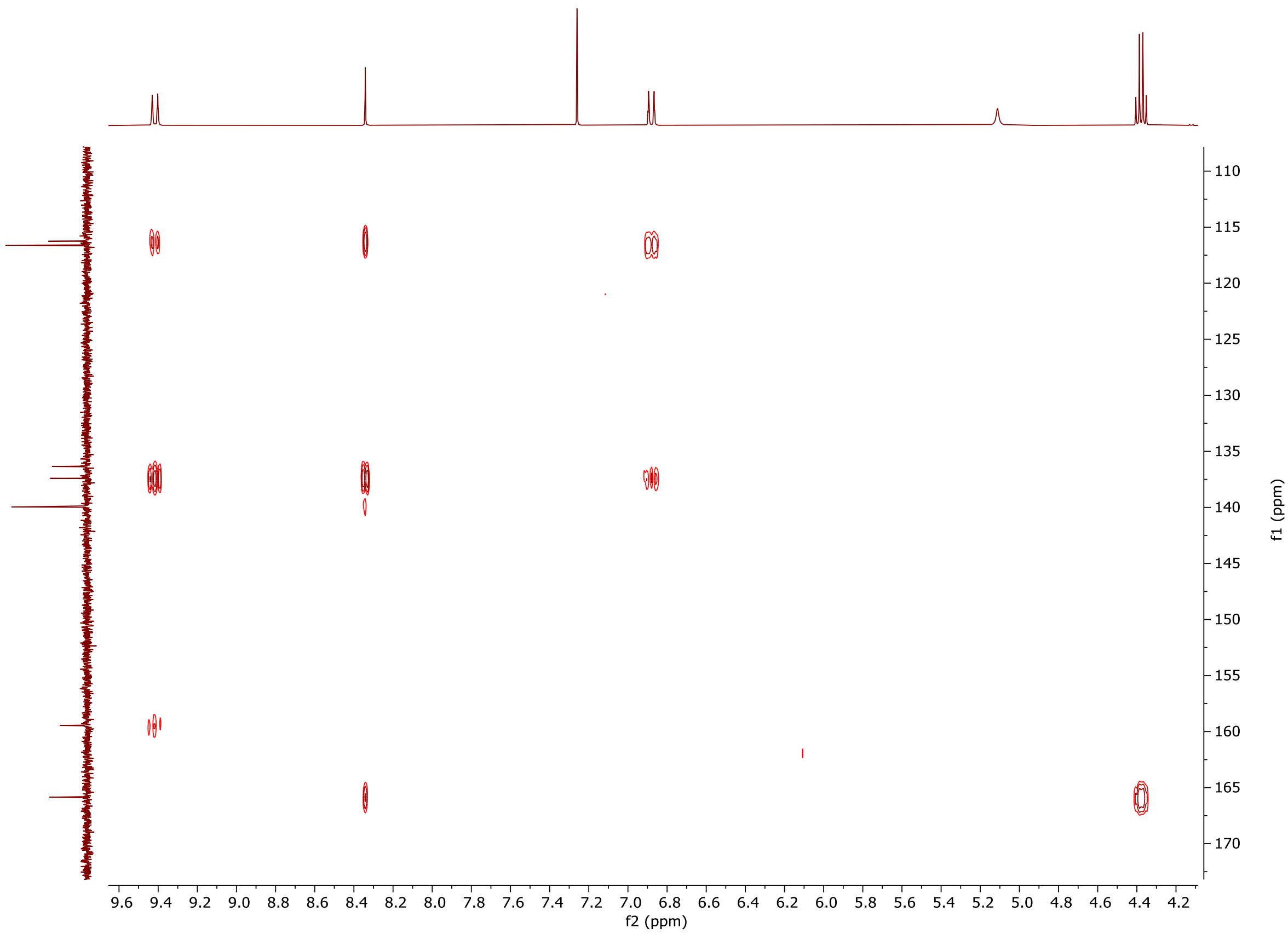

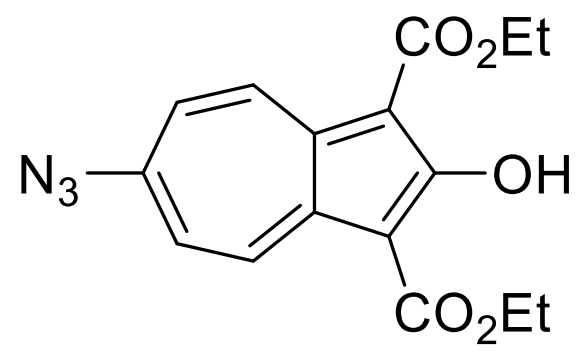

30

^{1}H-NMR, 400 MHz, $CDCl_3$

crude

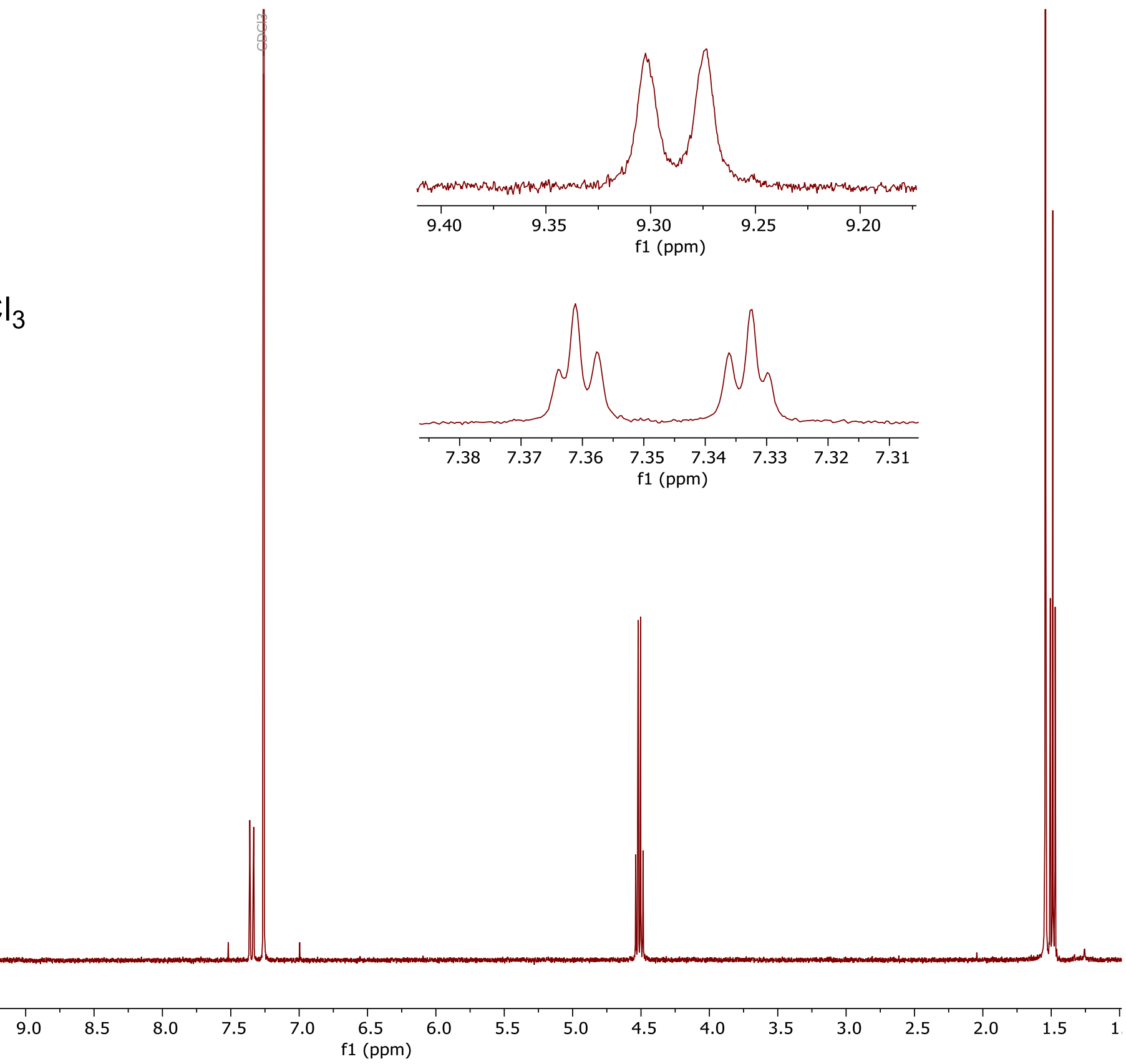

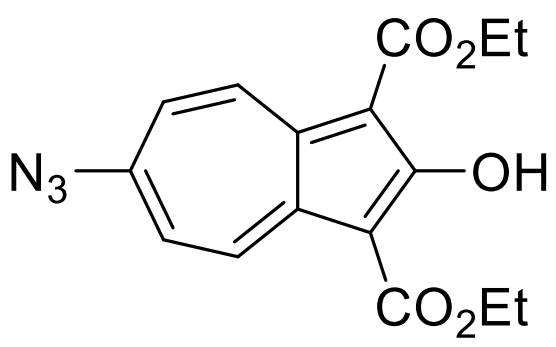

30

Mass spectrum

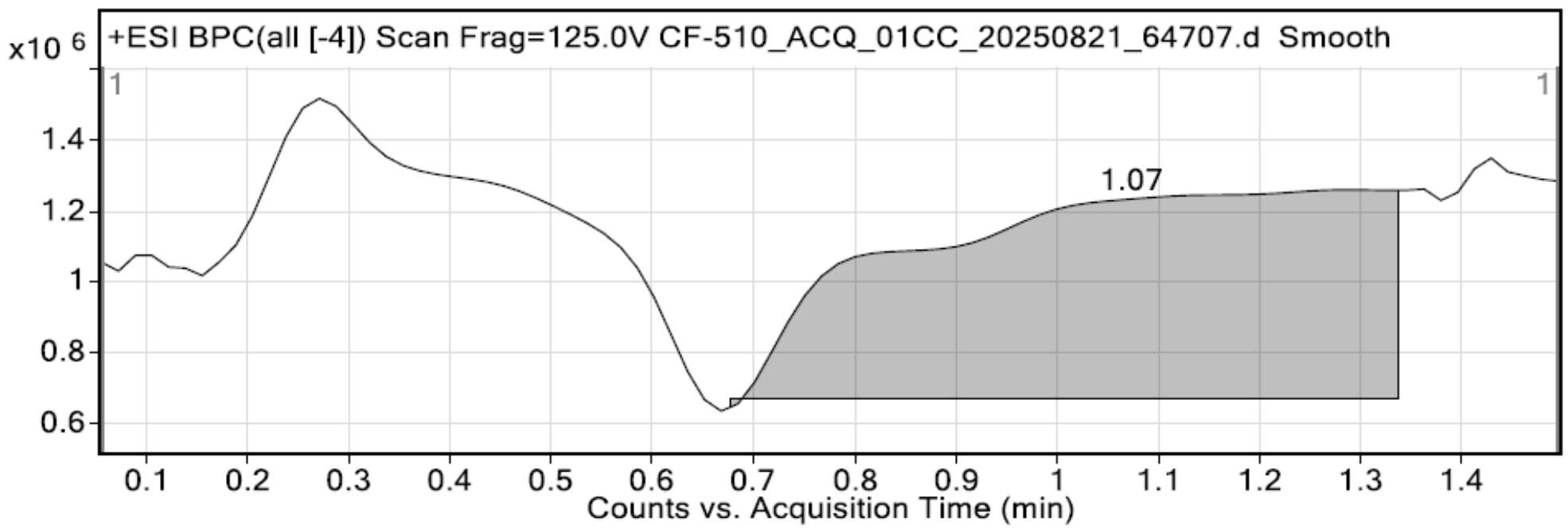

Figure 1: Base peak chromatogram

User Chromatogram Peak List

RT (min)	Area	Area %	Area Sum (%)	Base Peak (m/z)	Width (min)
1.07	18666564	100.00	100.00	149.9519	0.480

Compound Table

Compound Label	RT (min)	Observed mass (m/z)	Neutral observed mass (Da)	Theoretical mass (Da)	Mass error (ppm)	Isotope match score (%)
Cpd 1: C16 H15 N3 O5	0.66	352.0902	329.1009	329.1012	-0.70	97.69

Mass errors of between -5.00 and 5.00 ppm with isotope match scores above 60% are considered confirmation of molecular formulae

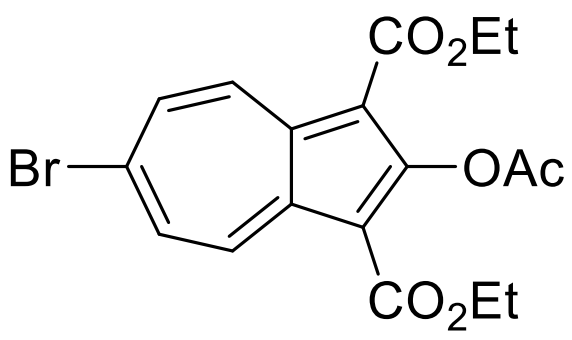

31

^{1}H-NMR, 500 MHz, $CDCl_3$

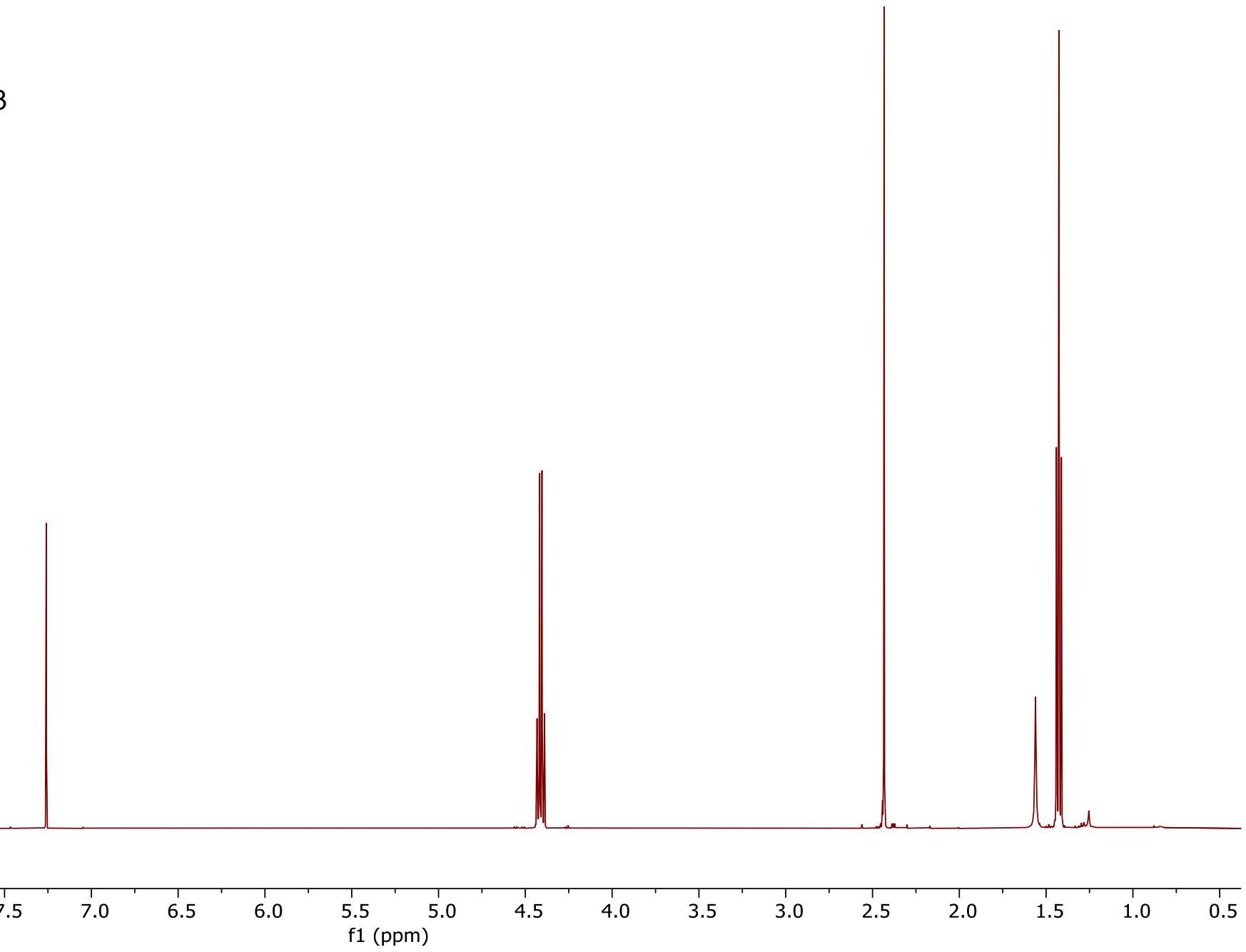

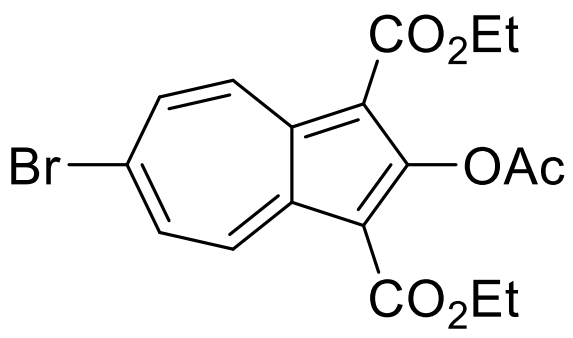

31

^{13}C-NMR, 126 MHz, $CDCl_3$

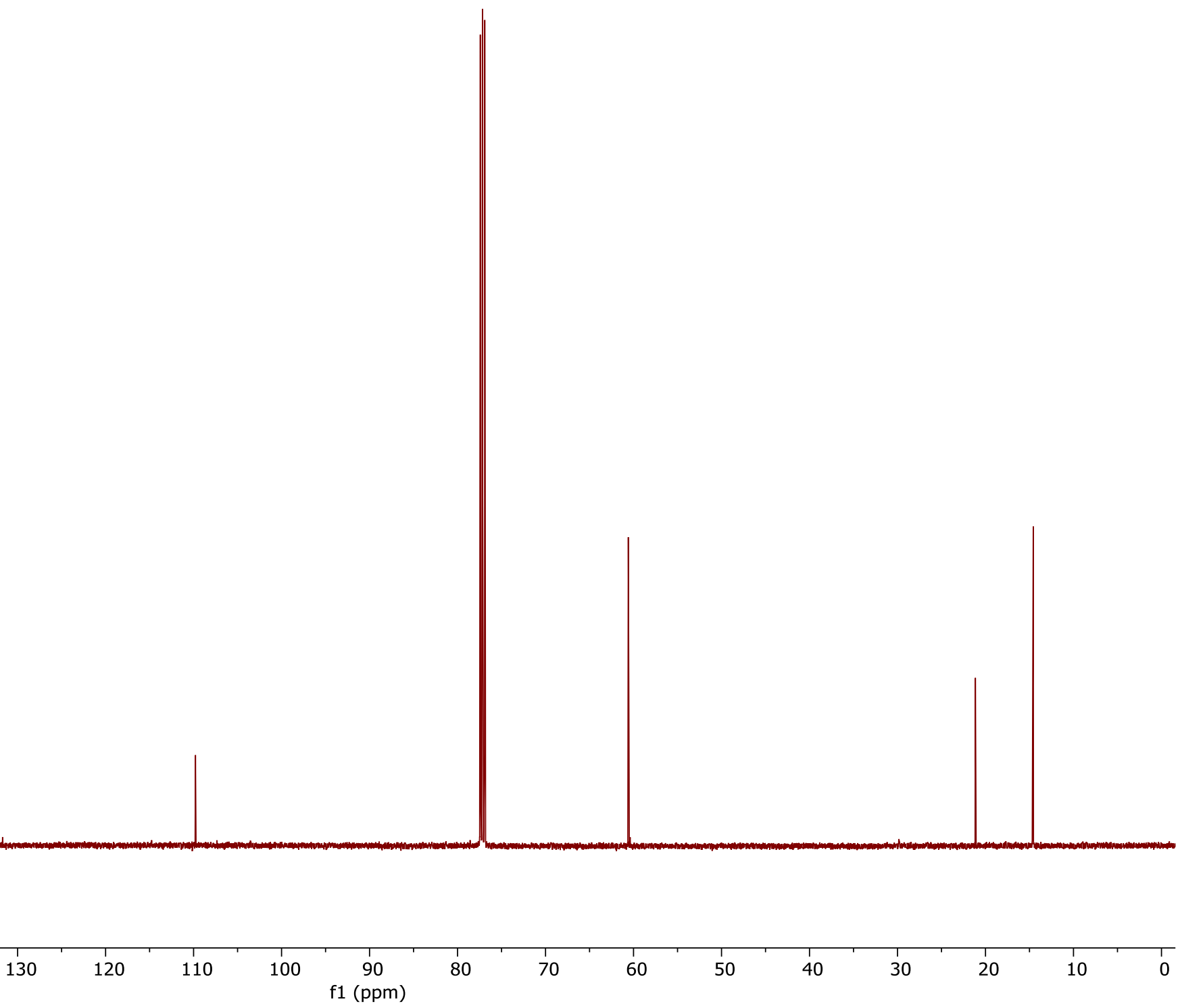

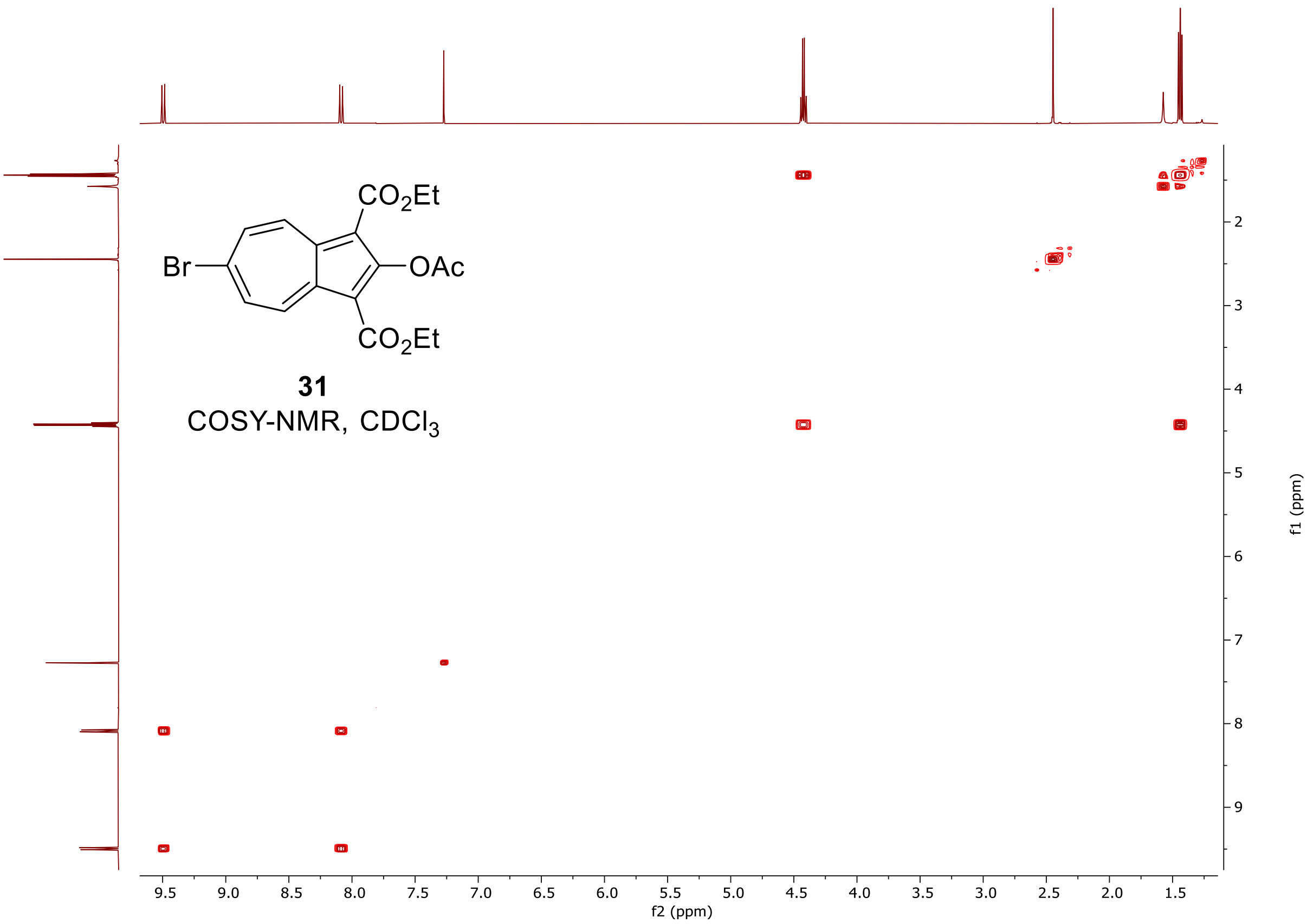
CO2Et
Br
OAc
CO2Et
31
COSY-NMR, CDCl3
f2 (ppm)
f1 (ppm)

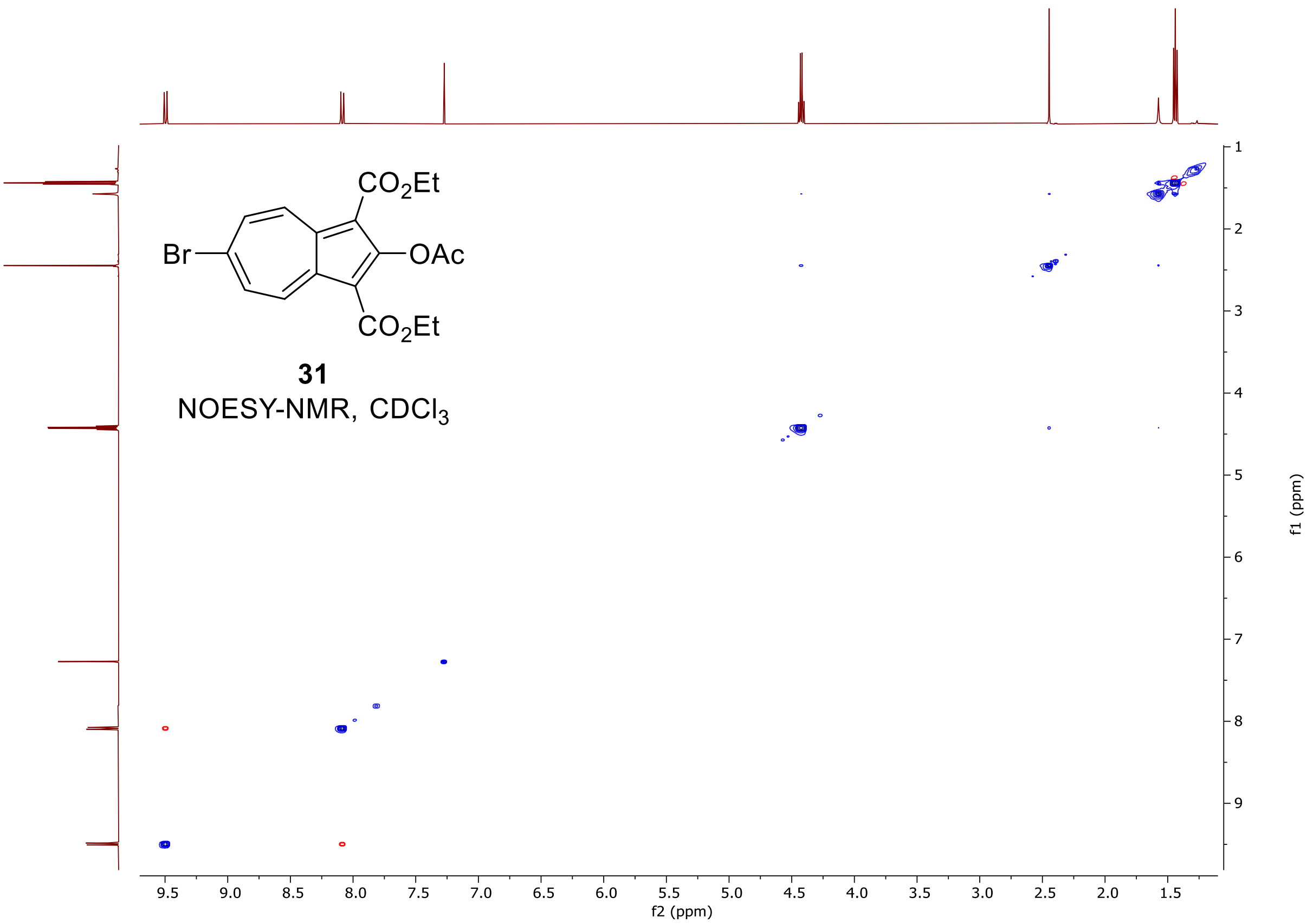
CO2Et
Br
OAc
CO2Et
31
NOESY-NMR, CDCl3
f2 (ppm)
f1 (ppm)

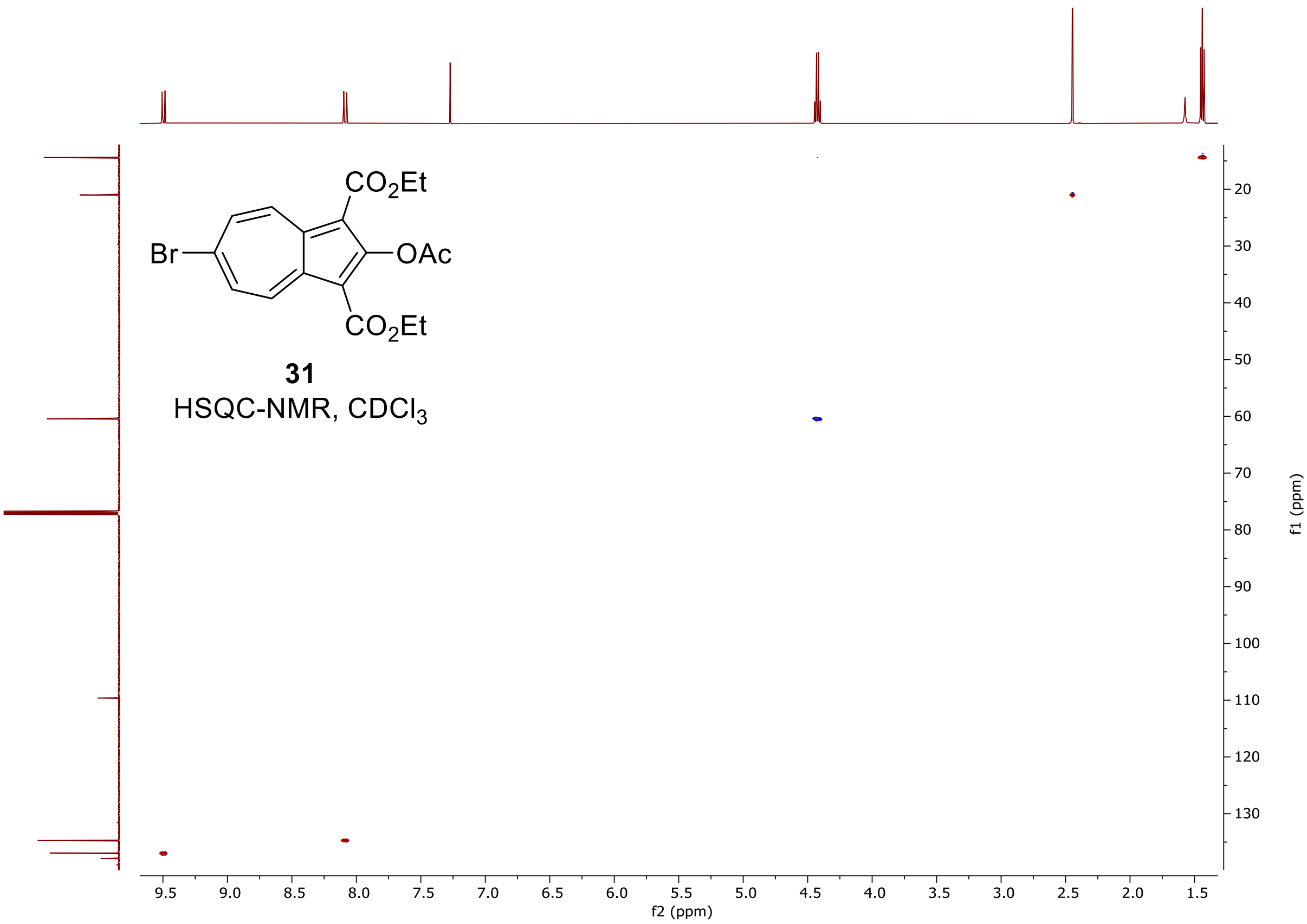
CO2Et
Br
OAc
CO2Et
31
HSQC-NMR, CDCl3
f2 (ppm)
f1 (ppm)

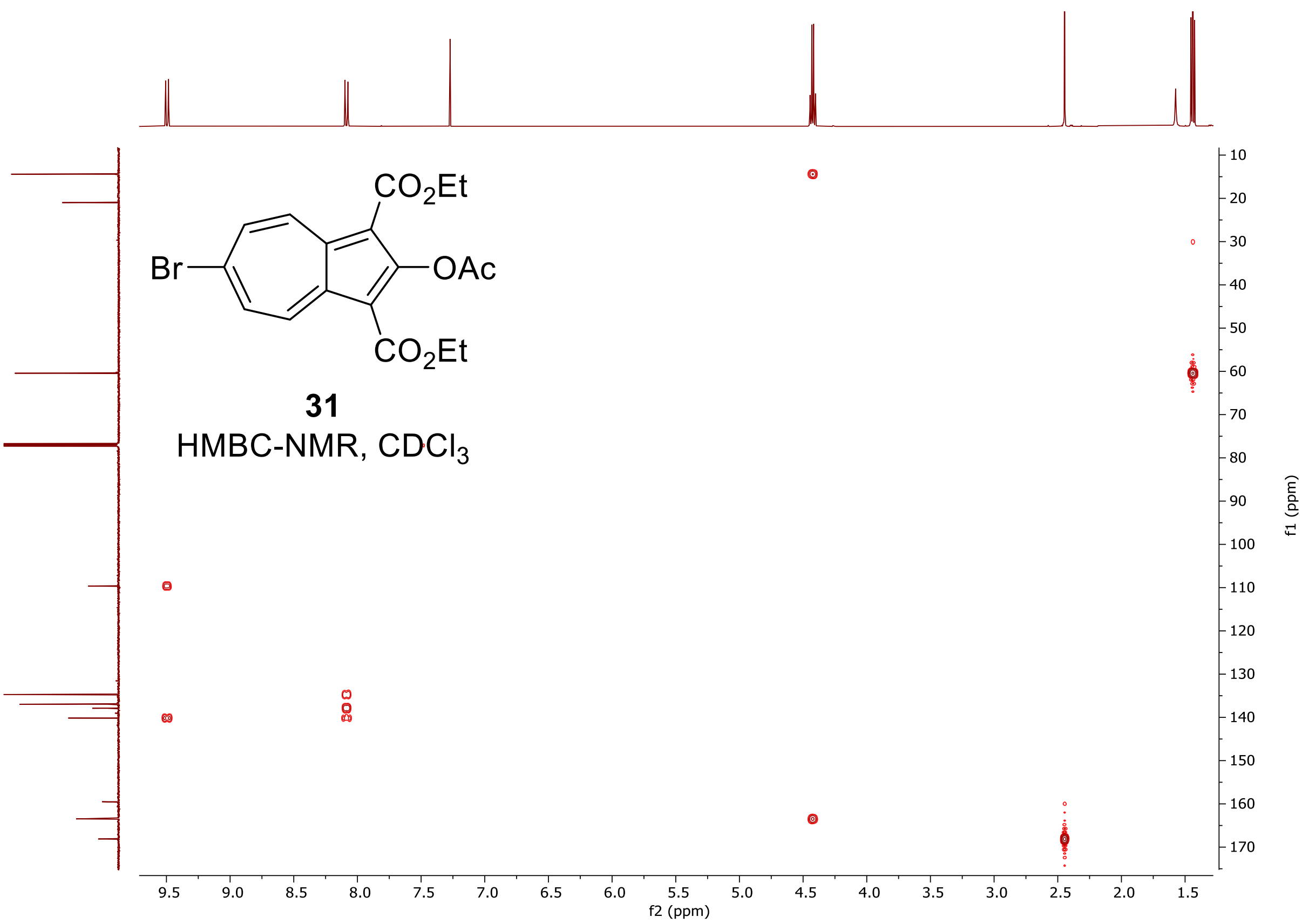
CO2Et
Br
OAc
CO2Et
31
HMBC-NMR, CDCl3
f2 (ppm)
f1 (ppm)

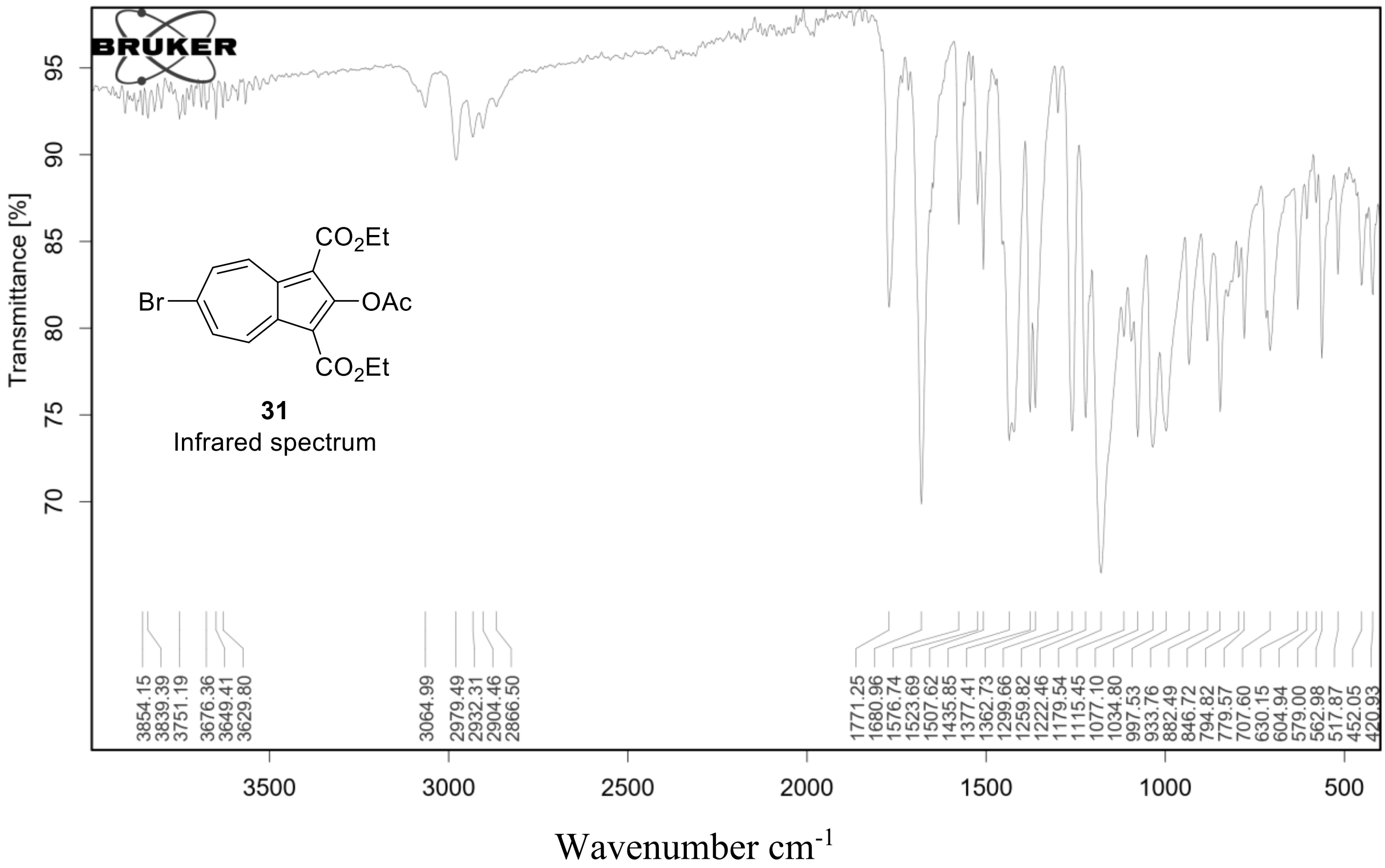

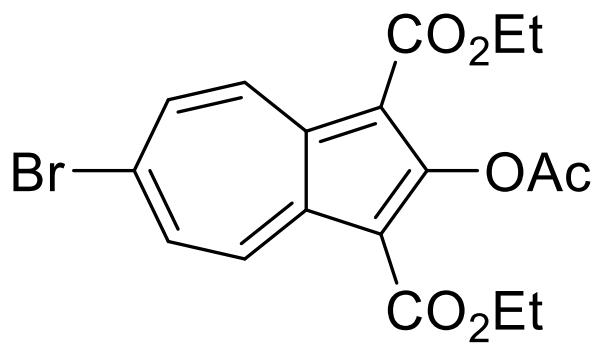

31, HRMS-ESI$^+$

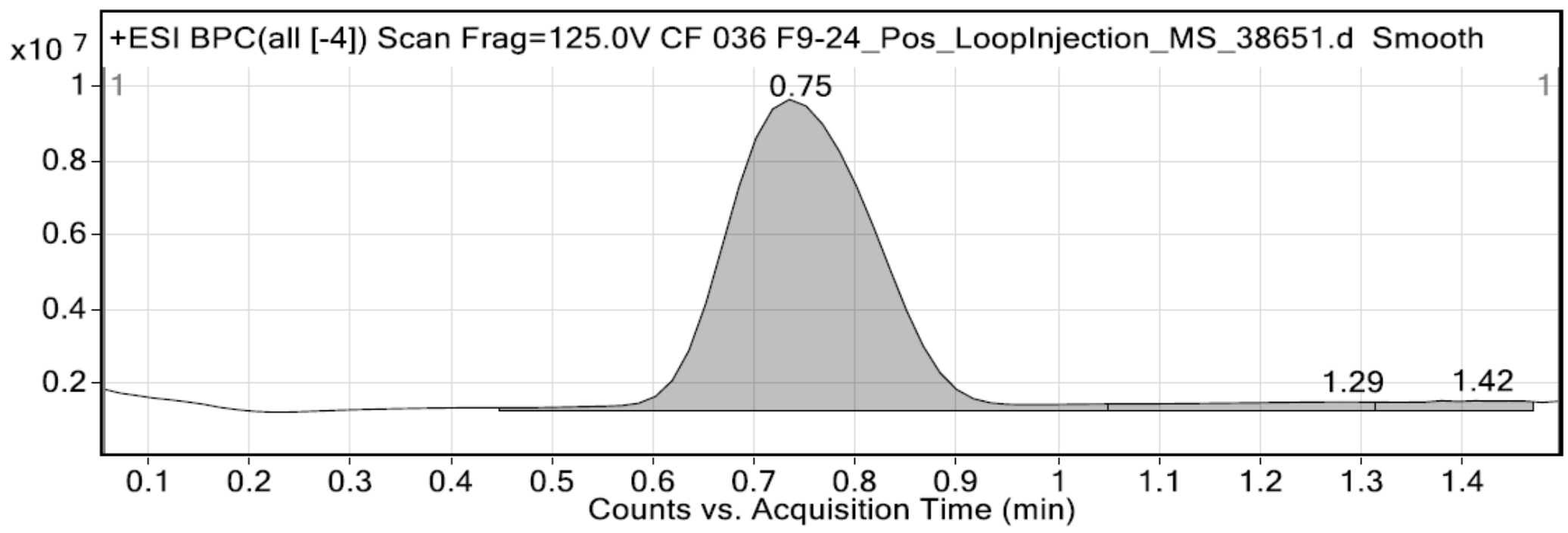

Figure 1: Base peak chromatogram

User Chromatogram Peak List

RT (min)	Area	Area %	Area Sum (%)	Base Peak (m/z)	Width (min)
0.75	86657473	100.00	93.43	433.0063	0.180
1.29	3587009	4.14	3.87	149.9543	0.200
1.42	2502583	2.89	2.70	149.9543	0.150

Compound Table

Compound Label	RT (min)	Observed mass (m/z)	Neutral observed mass (Da)	Theoretical mass (Da)	Mass error (ppm)	Isotope match score (%)
Cpd 1: C18 H17 Br O6	0.75	433.0082	408.0208	408.0209	-0.15	99.22

Mass errors of between -5.00 and 5.00 ppm with isotope match scores above 60% are considered confirmation of molecular formulae

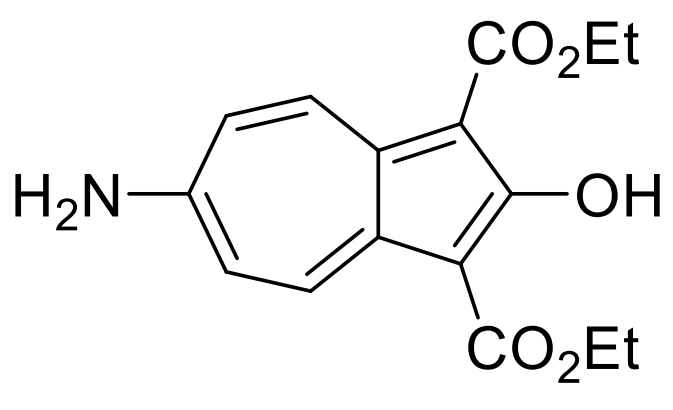

32

^{1}H-NMR, 400 MHz, $CDCl_3$

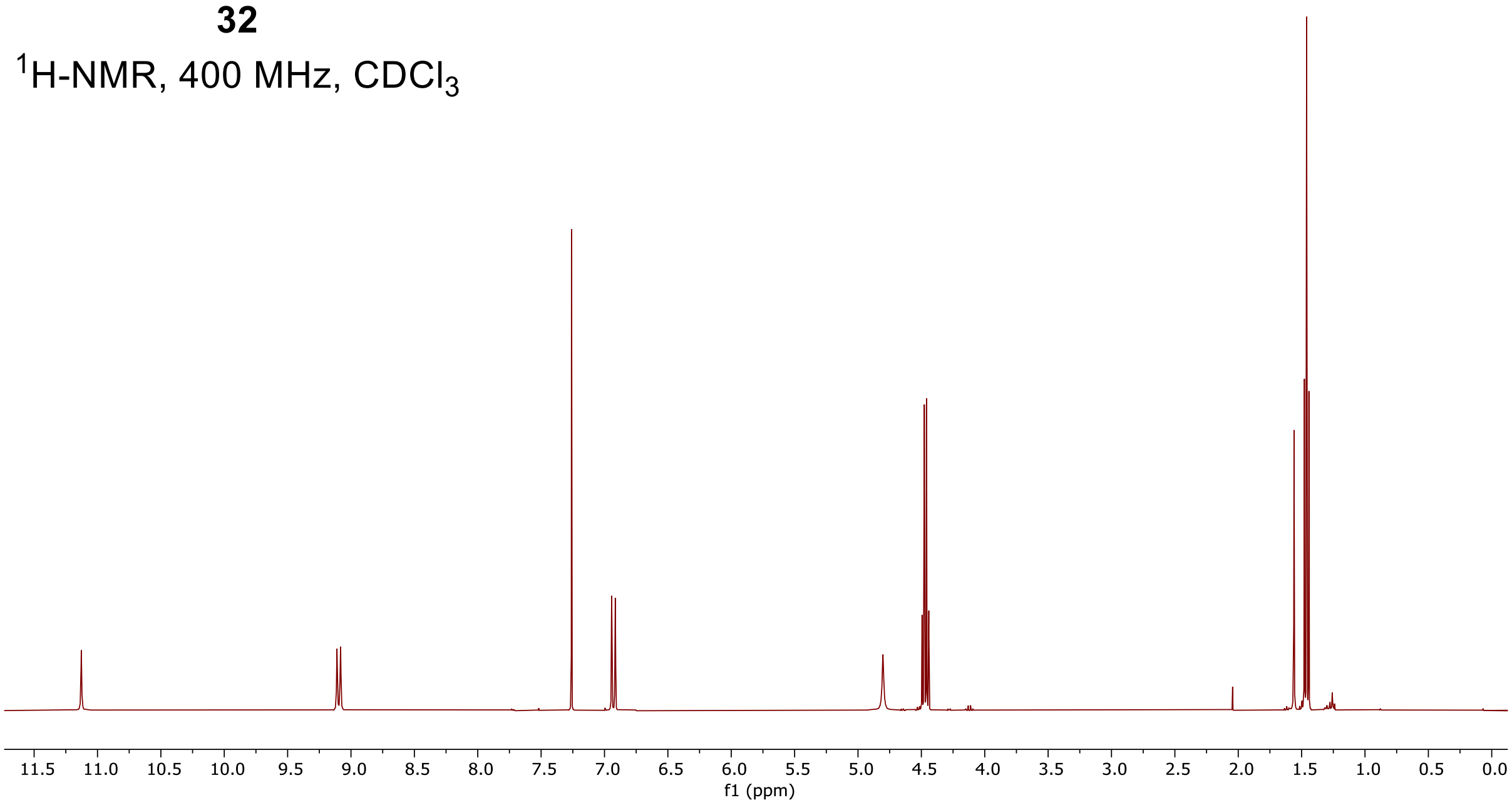

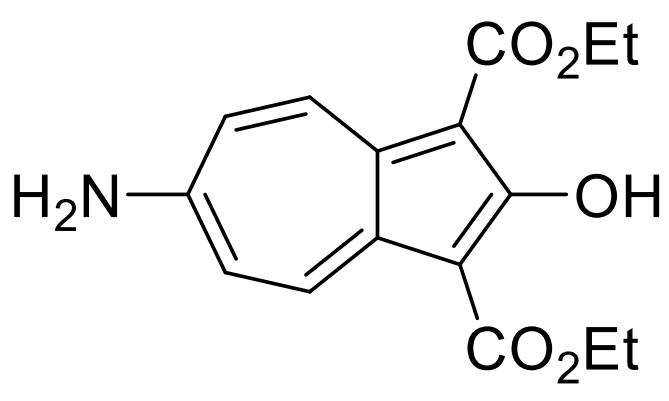

32

^{13}C-NMR, 101 MHz, $CDCl_3$

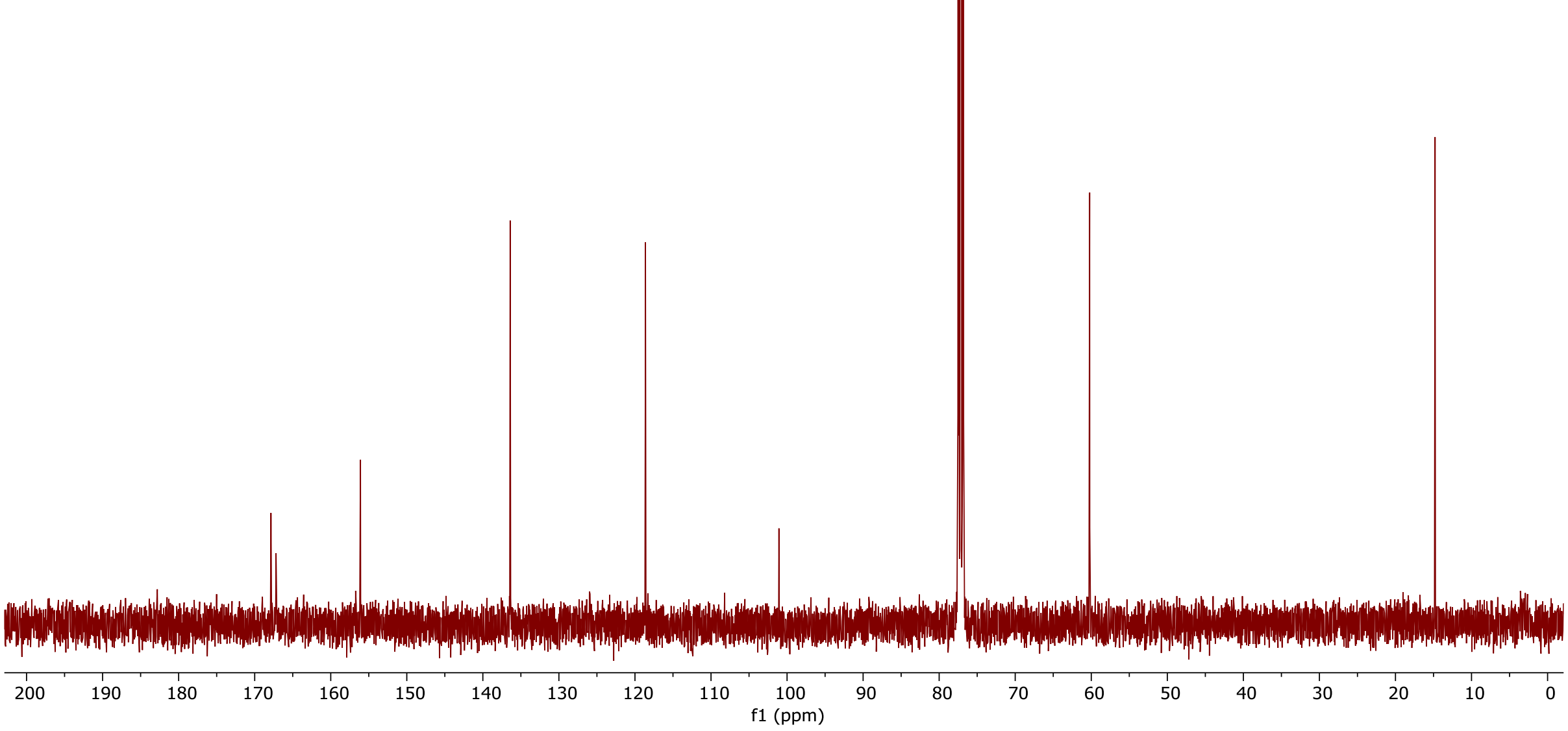

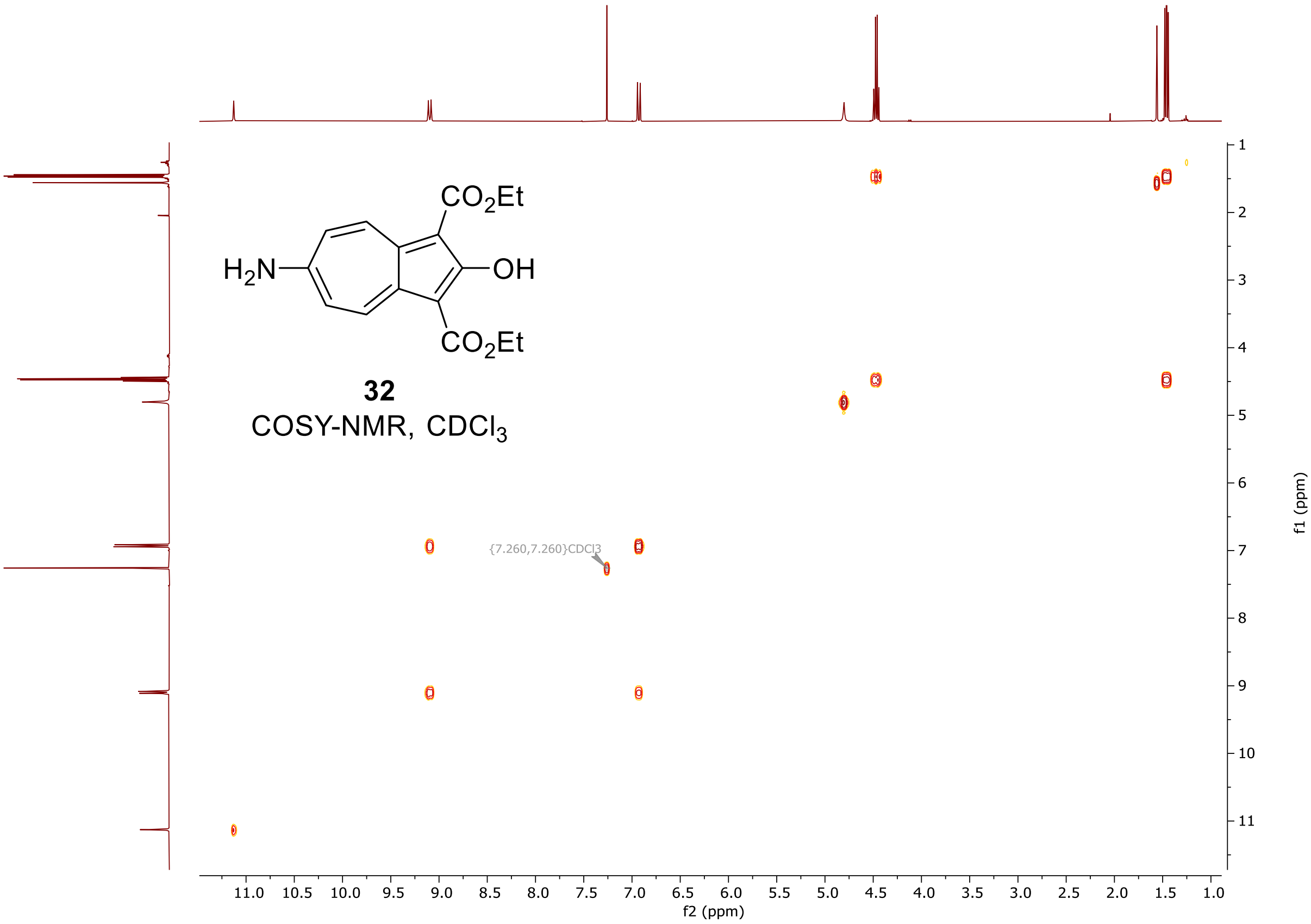
CO2Et
H2N
OH
CO2Et
32
COSY-NMR, CDCl3
{7.260,7.260}CDCl3
f2 (ppm)
f1 (ppm)

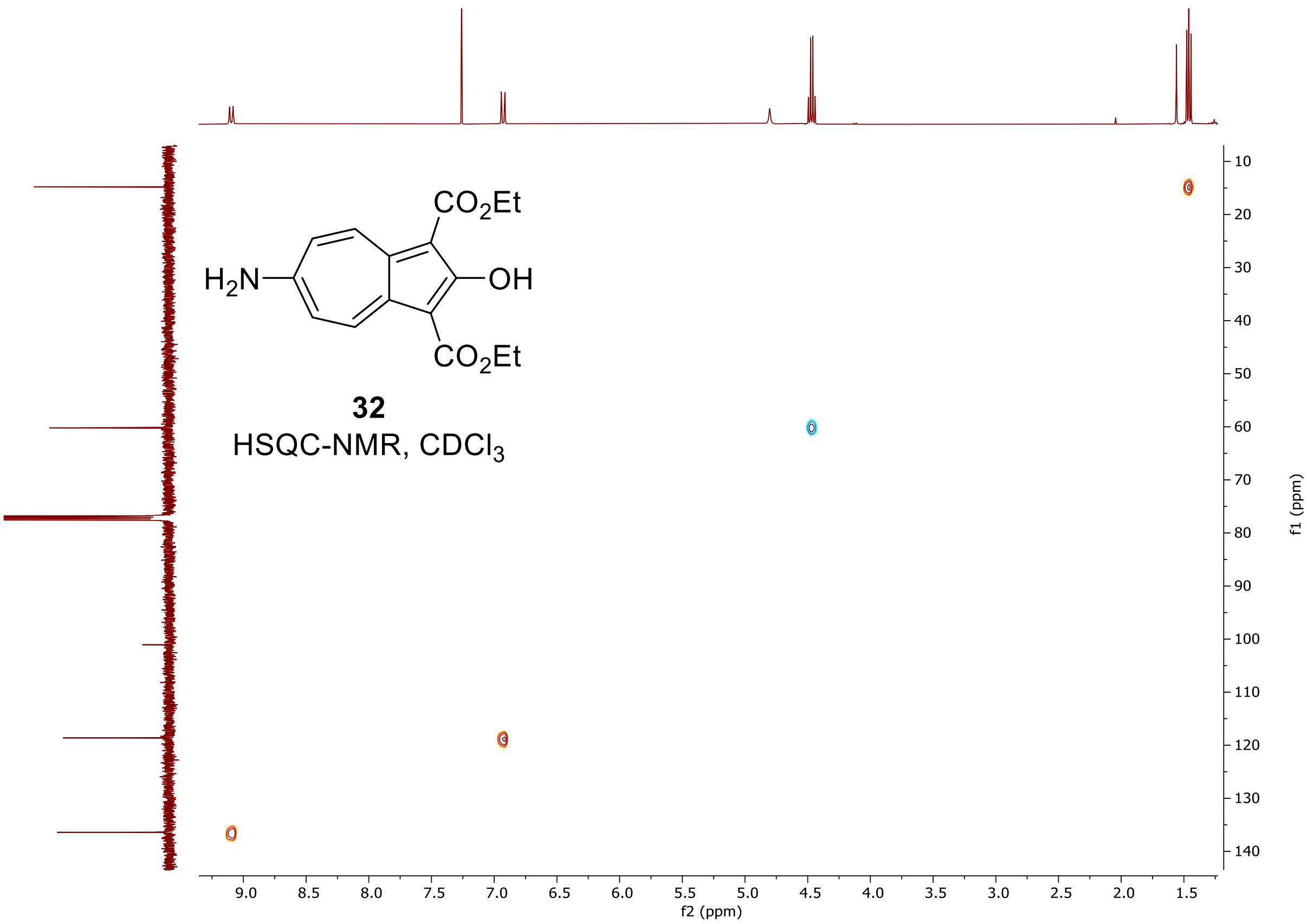
CO2Et
H2N
OH
CO2Et
32
HSQC-NMR, CDCl3
f2 (ppm)
f1 (ppm)

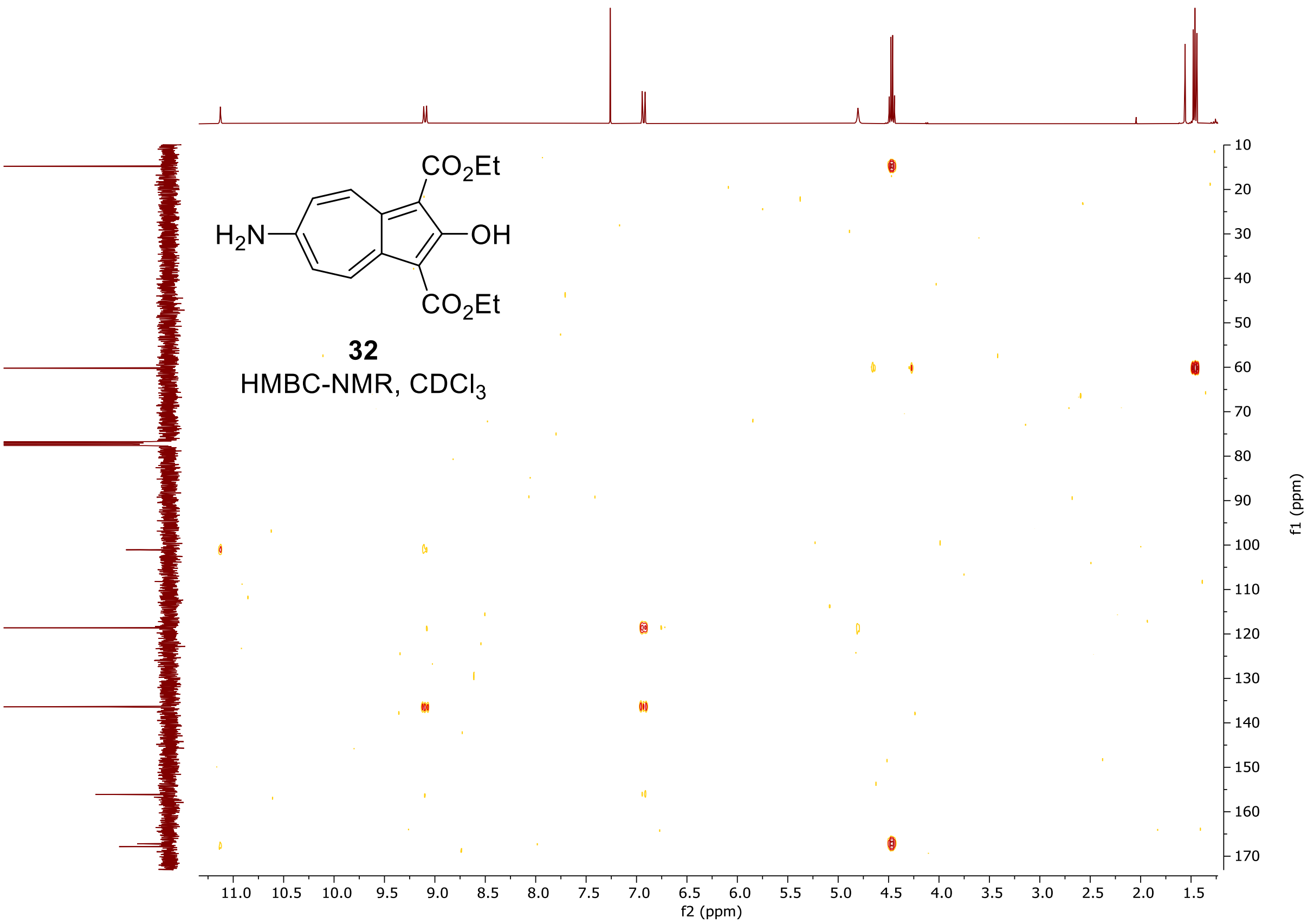
CO2Et
H2N
OH
CO2Et
32
HMBC-NMR, CDCl3
f2 (ppm)
f1 (ppm)

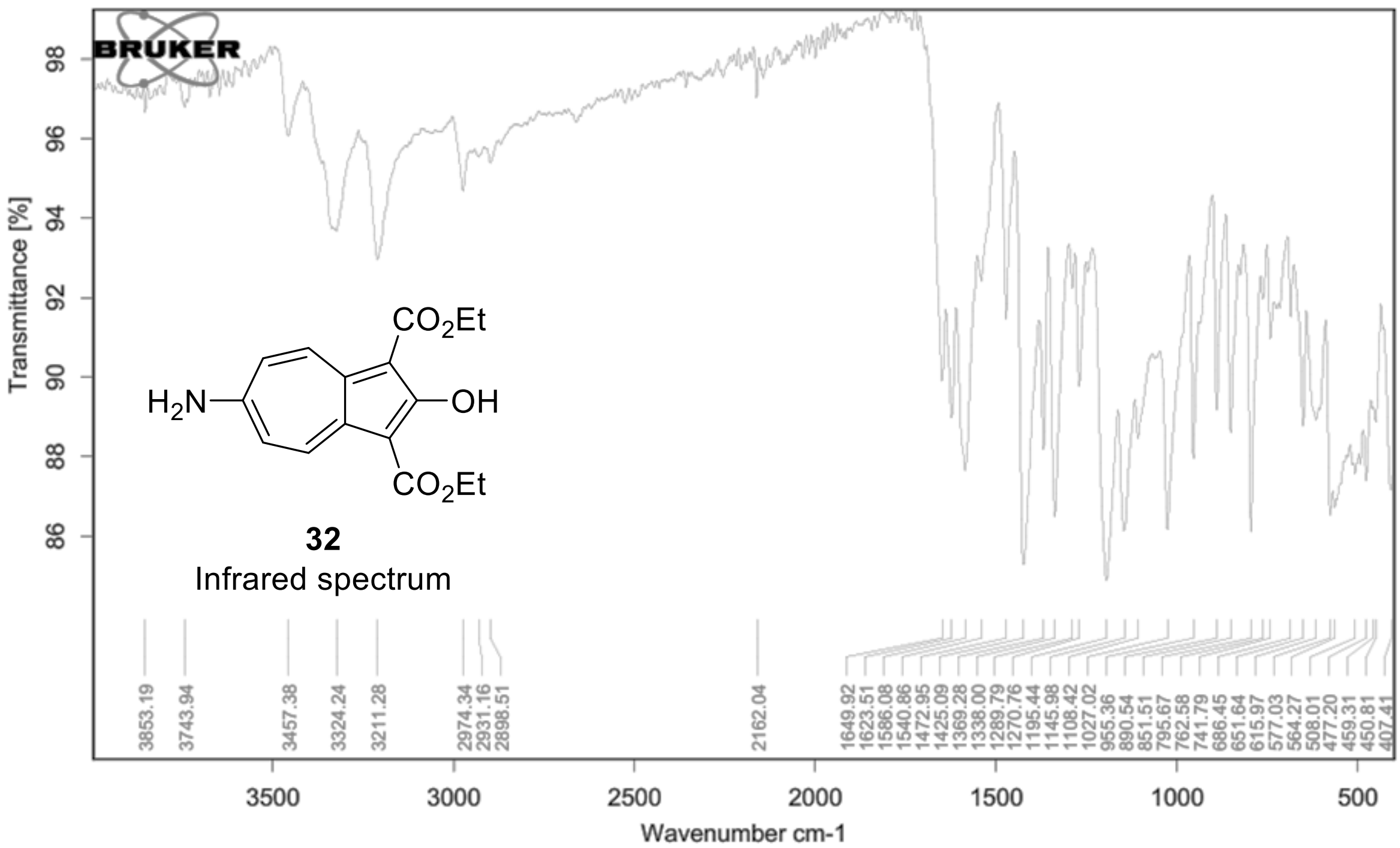
BRUKER
Transmittance [%]
CO2Et
H2N
OH
CO2Et
32
Infrared spectrum
3853.19
3743.94
3457.38
3324.24
3211.28
2974.34
2931.16
2898.51
2162.04
1649.92
1623.51
1586.08
1540.86
1472.95
1425.09
1369.28
1338.00
1289.79
1270.76
1195.44
1145.98
1108.42
1027.02
955.36
890.54
851.51
795.67
762.58
741.79
686.45
651.64
615.97
577.03
564.27
508.01
477.20
459.31
450.81
407.41
Wavenumber cm-1

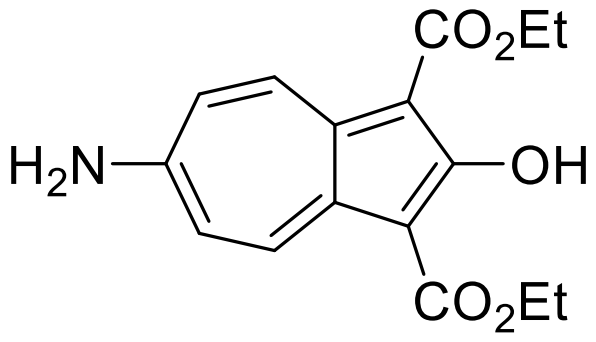

32

HRMS-ESI⁻

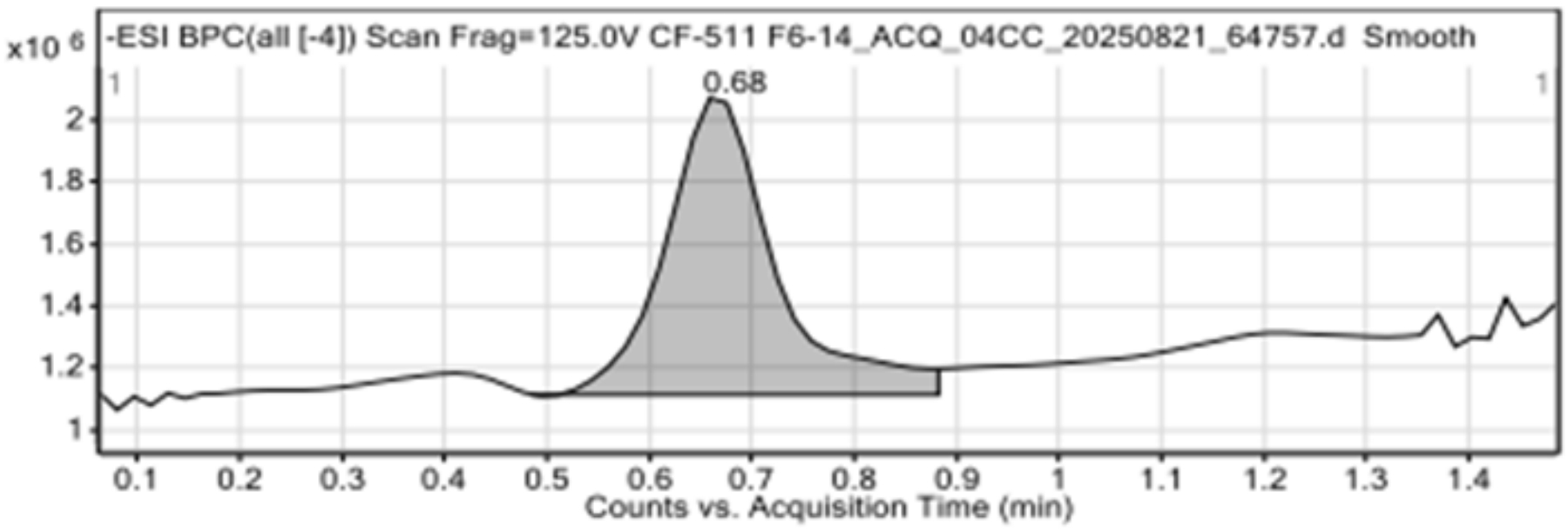

Figure 1: Base peak chromatogram

User Chromatogram Peak List

RT (min)	Area	Area %	Area Sum (%)	Base Peak (m/z)	Width (min)
0.68	7160796	100.00	100.00	1033.9759	0.160

Compound Table

Compound Label	RT (min)	Observed mass (m/z)	Neutral observed mass (Da)	Theoretical mass (Da)	Mass error (ppm)	Isotope match score (%)
Cpd 1: C16 H17 N O5	0.59	302.1032	303.1104	303.1107	-0.84	99.85

Mass errors of between -5.00 and 5.00 ppm with isotope match scores above 60% are considered confirmation of molecular formulae

Crystallographic Data:

Crystal data and structure refinement for **14**

Identification code	s23sel13	
Empirical formula	$C_{16} H_{18} N_2 O_4$	
Formula weight	302.32	
Temperature	150.00(10) K	
Wavelength	1.54184 Å	
Crystal system	Monoclinic	
Space group	$P2_1/c$	
Unit cell dimensions	a = 11.4991(3) Å	α= 90°.
	b = 12.1257(2) Å	β= 104.297(2)°.
	c = 10.9303(2) Å	γ = 90°.
Volume	1476.86(5) Å3	
Z	4	
Density (calculated)	1.360 Mg/m3	
Absorption coefficient	0.816 mm-1	
F(000)	640	
Crystal size	0.290 x 0.180 x 0.020 mm3	
Theta range for data collection	3.967 to 72.879°.	
Index ranges	-14<=h<=11, -14<=k<=14, -9<=l<=13	
Reflections collected	9500	
Independent reflections	2905 [R(int) = 0.0260]	
Completeness to theta = 67.684°	100.0 %	
Absorption correction	Semi-empirical from equivalents	
Max. and min. transmission	1.00000 and 0.81326	
Refinement method	Full-matrix least-squares on F2	
Data / restraints / parameters	2905 / 0 / 217	
Goodness-of-fit on F2	1.047	
Final R indices [I>2sigma(I)]	R1 = 0.0378, wR2 = 0.0965	
R indices (all data)	R1 = 0.0462, wR2 = 0.1011	
Extinction coefficient	n/a	
Largest diff. peak and hole	0.231 and -0.159 e.Å-3	

CCDC 2566798

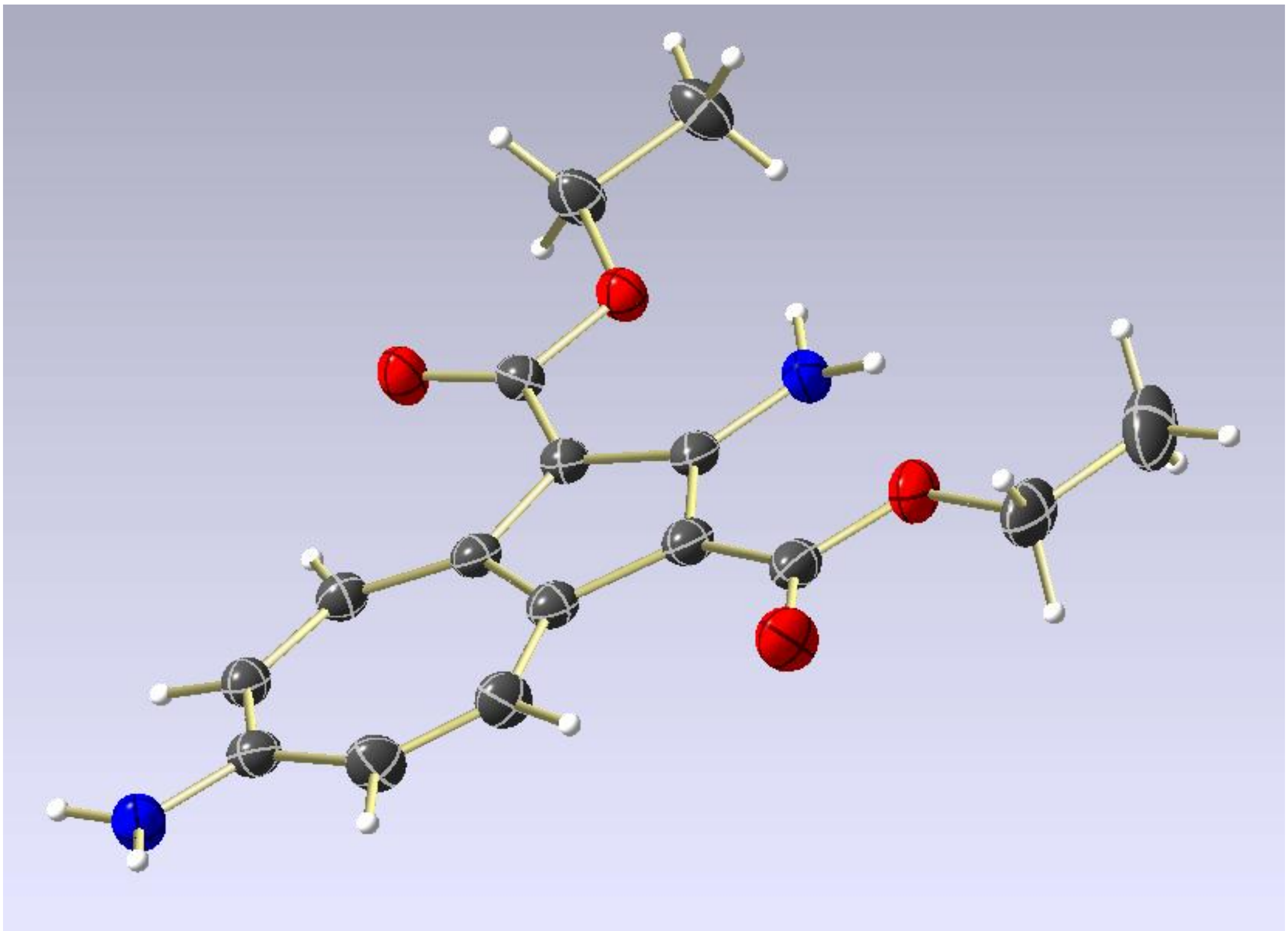

Crystal data and structure refinement for **15a**

Identification code	s23sel26
Empirical formula	$C_{18} H_{22} N_2 O_4$
Formula weight	330.37
Temperature	150(2) K
Wavelength	1.54184 Å
Crystal system	Triclinic
Space group	P-1
Unit cell dimensions	a = 7.4967(7) Å α= 68.534(8)°.
	b = 9.9700(9) Å β= 80.809(7)°.
	c = 12.7164(11) Å γ = 68.232(8)°.
Volume	821.13(14) Å3
Z	2
Density (calculated)	1.336 Mg/m3
Absorption coefficient	0.778 mm-1
F(000)	352
Crystal size	0.060 x 0.050 x 0.045 mm3
Theta range for data collection	3.736 to 72.898°.
Index ranges	-9<=h<=8, -9<=k<=12, -11<=l<=15
Reflections collected	5396
Independent reflections	3169 [R(int) = 0.0327]
Completeness to theta = 67.684°	99.8 %
Refinement method	Full-matrix least-squares on F2
Data / restraints / parameters	3169 / 0 / 229
Goodness-of-fit on F2	1.049
Final R indices [I>2sigma(I)]	R1 = 0.0560, wR2 = 0.1338
R indices (all data)	R1 = 0.0738, wR2 = 0.1470
Extinction coefficient	n/a
Largest diff. peak and hole	0.411 and -0.245 e.Å-3
CCDC 2566799	

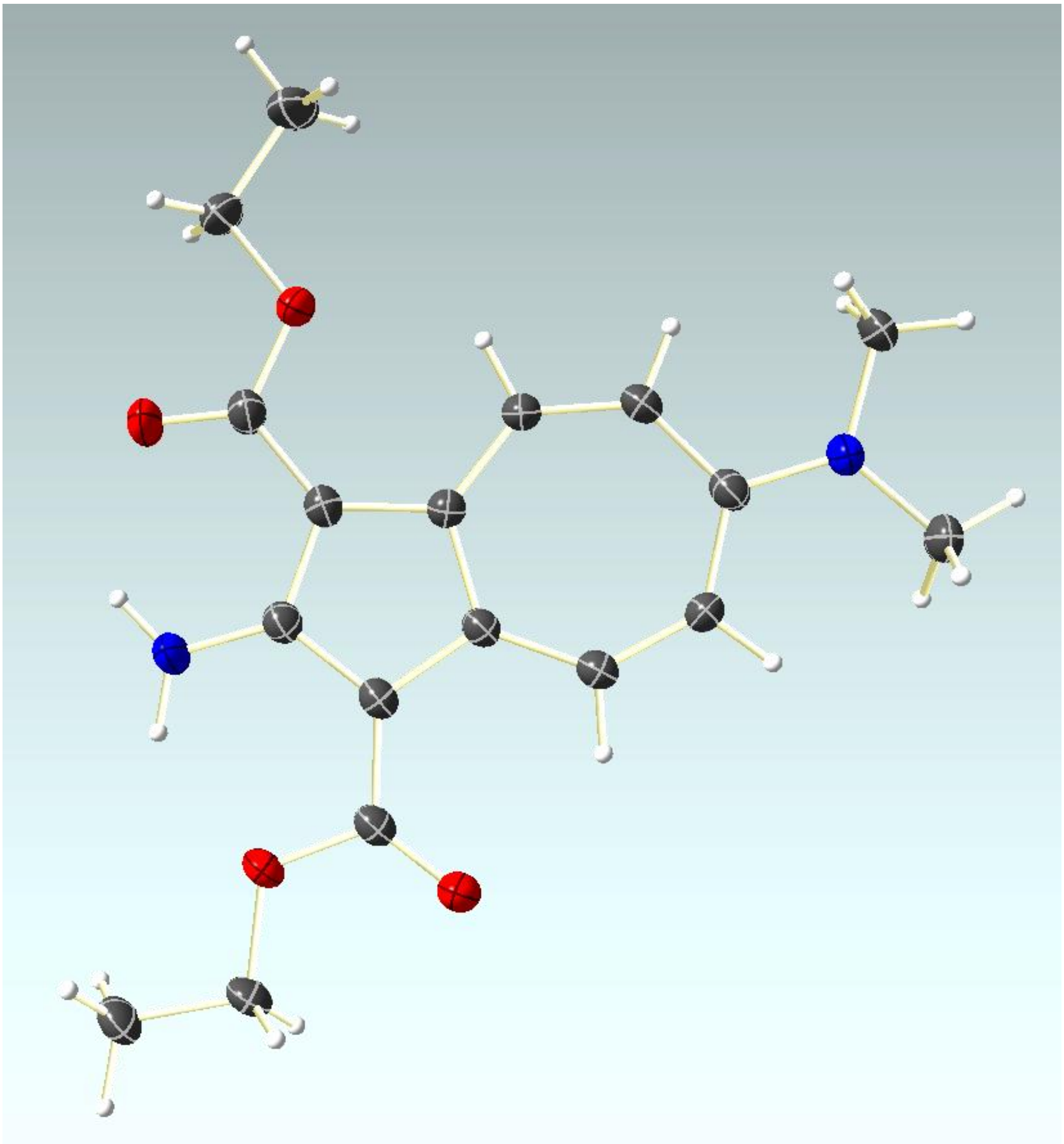

Crystal data and structure refinement for **15b**

Identification code	s23sel21
Empirical formula	$C_{21} H_{26} N_2 O_4$
Formula weight	370.44
Temperature	150.00(10) K
Wavelength	1.54184 Å
Crystal system	Monoclinic
Space group	$P2_1/c$
Unit cell dimensions	a = 11.43232(14) Å α= 90°.
	b = 11.17022(12) Å β= 98.2696(11)°.
	c = 14.50249(15) Å γ = 90°.
Volume	1832.73(4) Å3
Z	4
Density (calculated)	1.343 Mg/m3
Absorption coefficient	0.757 mm-1
F(000)	792
Crystal size	0.400 x 0.350 x 0.100 mm3
Theta range for data collection	3.907 to 72.672°.
Index ranges	-13<=h<=14, -13<=k<=13, -17<=l<=13
Reflections collected	12890
Independent reflections	3610 [R(int) = 0.0356]
Completeness to theta = 67.684°	100.0 %
Absorption correction	Semi-empirical from equivalents
Max. and min. transmission	1.00000 and 0.62768
Refinement method	Full-matrix least-squares on F2
Data / restraints / parameters	3610 / 0 / 254
Goodness-of-fit on F2	1.028
Final R indices [I>2sigma(I)]	R1 = 0.0391, wR2 = 0.0975
R indices (all data)	R1 = 0.0427, wR2 = 0.1006
Extinction coefficient	n/a
Largest diff. peak and hole	0.229 and -0.238 e.Å-3
CCDC 2566800	

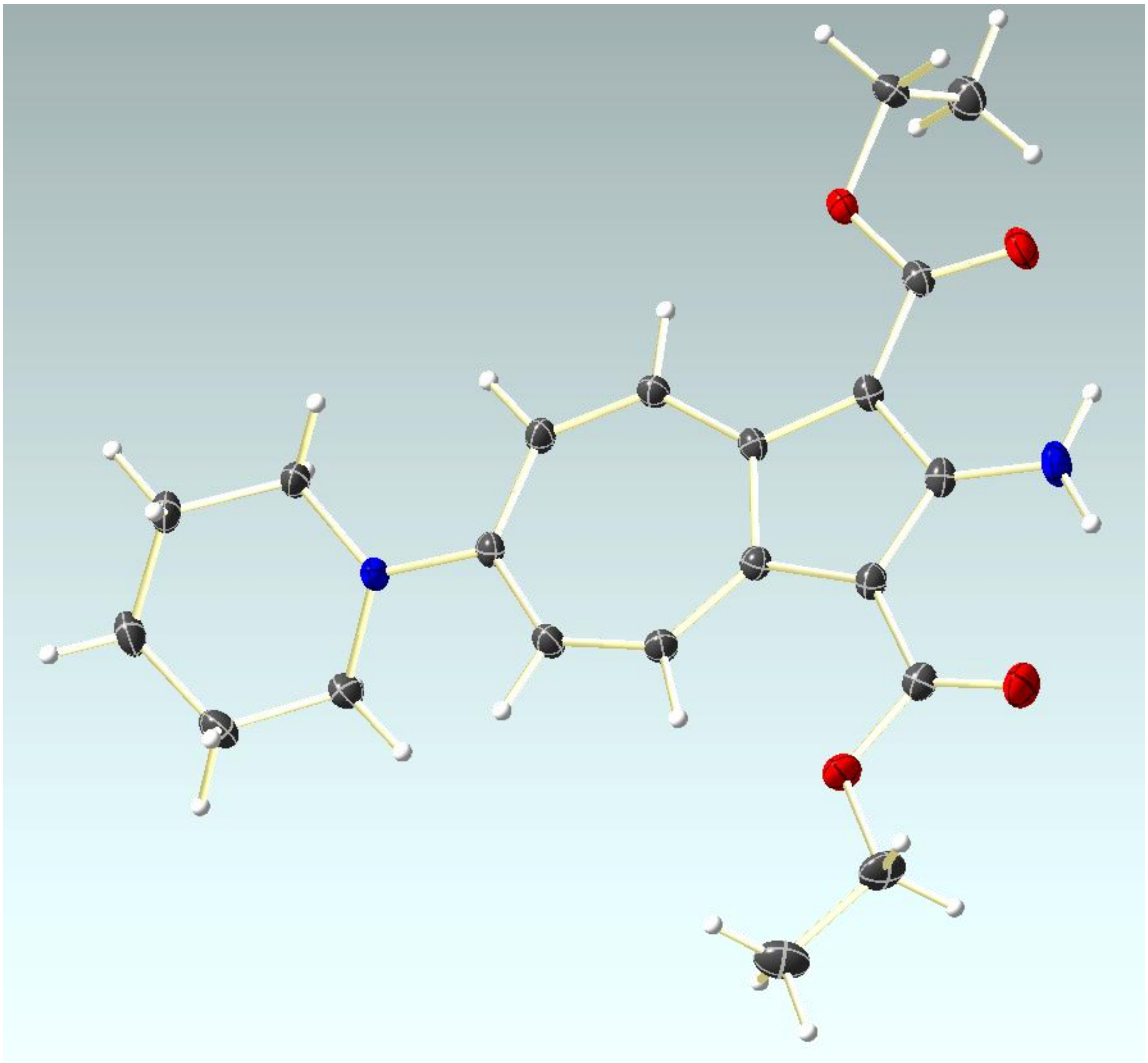

Crystal data and structure refinement for **15c**

Identification code	s23sel17
Empirical formula	$C_{20} H_{24} N_2 O_5$
Formula weight	372.41
Temperature	150.01(10) K
Wavelength	1.54184 Å
Crystal system	Monoclinic
Space group	I2/a
Unit cell dimensions	a = 18.6942(3) Å α= 90°.
	b = 8.53510(10) Å β= 108.848(2)°.
	c = 24.4739(4) Å γ = 90°.
Volume	3695.59(10) Å3
Z	8
Density (calculated)	1.339 Mg/m3
Absorption coefficient	0.797 mm-1
F(000)	1584
Crystal size	0.385 x 0.259 x 0.111 mm3
Theta range for data collection	3.817 to 72.819°.
Index ranges	-23<=h<=19, -10<=k<=8, -29<=l<=30
Reflections collected	13299
Independent reflections	3669 [R(int) = 0.0274]
Completeness to theta = 67.684°	100.0 %
Absorption correction	Semi-empirical from equivalents
Max. and min. transmission	1.00000 and 0.86125
Refinement method	Full-matrix least-squares on F2
Data / restraints / parameters	3669 / 0 / 311
Goodness-of-fit on F2	1.041
Final R indices [I>2sigma(I)]	R1 = 0.0426, wR2 = 0.1113
R indices (all data)	R1 = 0.0456, wR2 = 0.1146
Extinction coefficient	n/a
Largest diff. peak and hole	0.383 and -0.240 e.Å-3

CCDC 2566801

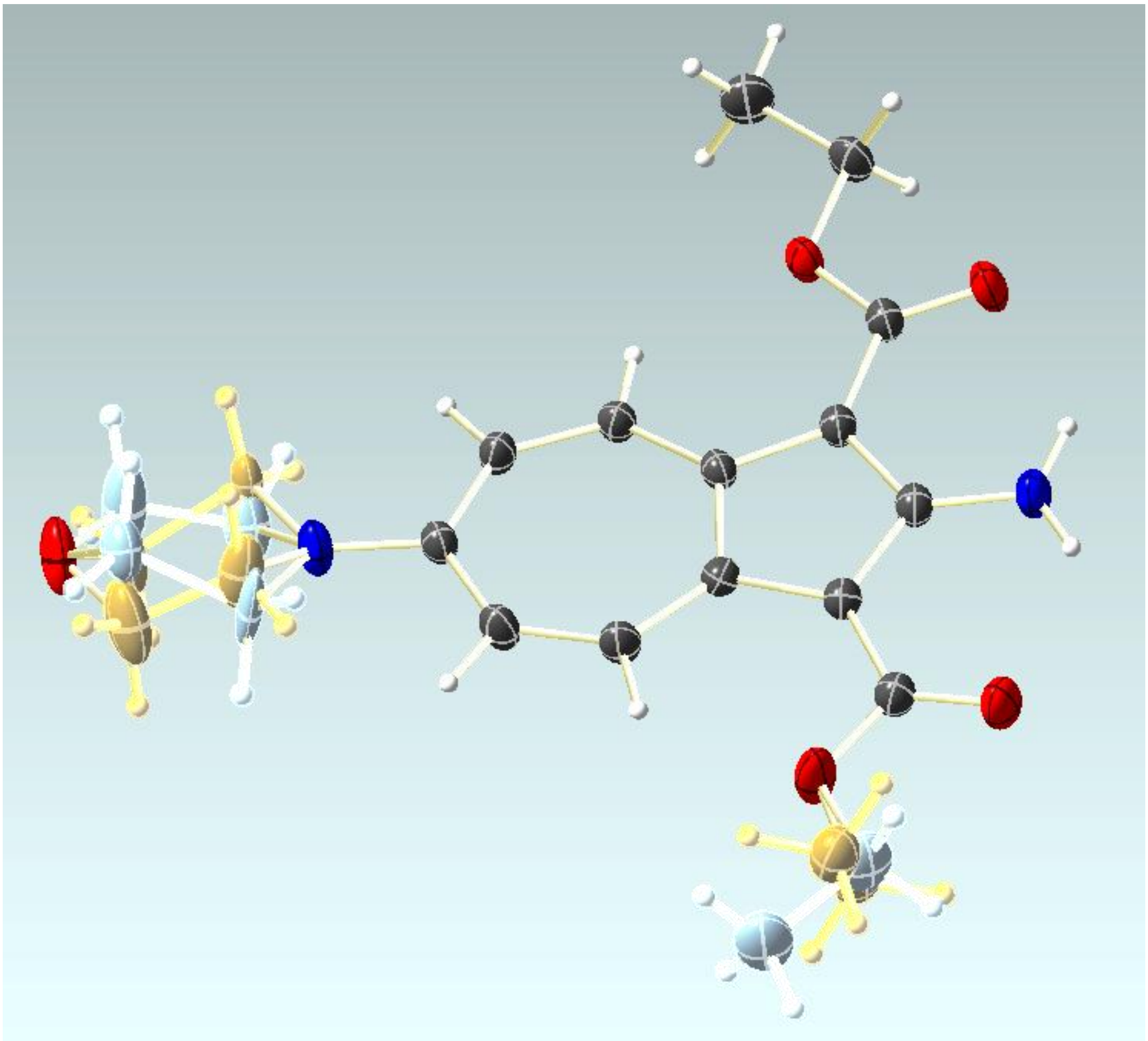

Notes on refinement:

one OEt group and OC_4H_8N are disordered over two sites in the ratio 1:1.

The N-H hydrogen atoms have been located in the difference Fourier map and were refined freely.

Crystal data and structure refinement for **15d**

Identification code	s23sel22	
Empirical formula	$C_{20} H_{24} N_2 O_4$	
Formula weight	356.41	
Temperature	150.00(10) K	
Wavelength	1.54184 Å	
Crystal system	Triclinic	
Space group	P-1	
Unit cell dimensions	a = 8.7225(2) Å	α= 68.646(2)°.
	b = 10.2096(3) Å	β= 78.350(2)°.
	c = 10.7419(3) Å	γ = 82.209(2)°.
Volume	870.56(4) Å3	
Z	2	
Density (calculated)	1.360 Mg/m3	
Absorption coefficient	0.776 mm-1	
F(000)	380	
Crystal size	0.150 x 0.070 x 0.010 mm3	
Theta range for data collection	4.481 to 72.864°.	
Index ranges	-9<=h<=10, -12<=k<=12, -13<=l<=13	
Reflections collected	13441	
Independent reflections	3454 [R(int) = 0.0287]	
Completeness to theta = 67.684°	99.9 %	
Absorption correction	Semi-empirical from equivalents	
Max. and min. transmission	1.00000 and 0.82243	
Refinement method	Full-matrix least-squares on F2	
Data / restraints / parameters	3454 / 0 / 245	
Goodness-of-fit on F2	1.037	
Final R indices [I>2sigma(I)]	R1 = 0.0377, wR2 = 0.0976	
R indices (all data)	R1 = 0.0434, wR2 = 0.1013	
Extinction coefficient	n/a	
Largest diff. peak and hole	0.291 and -0.192 e.Å-3	

CCDC 2566802

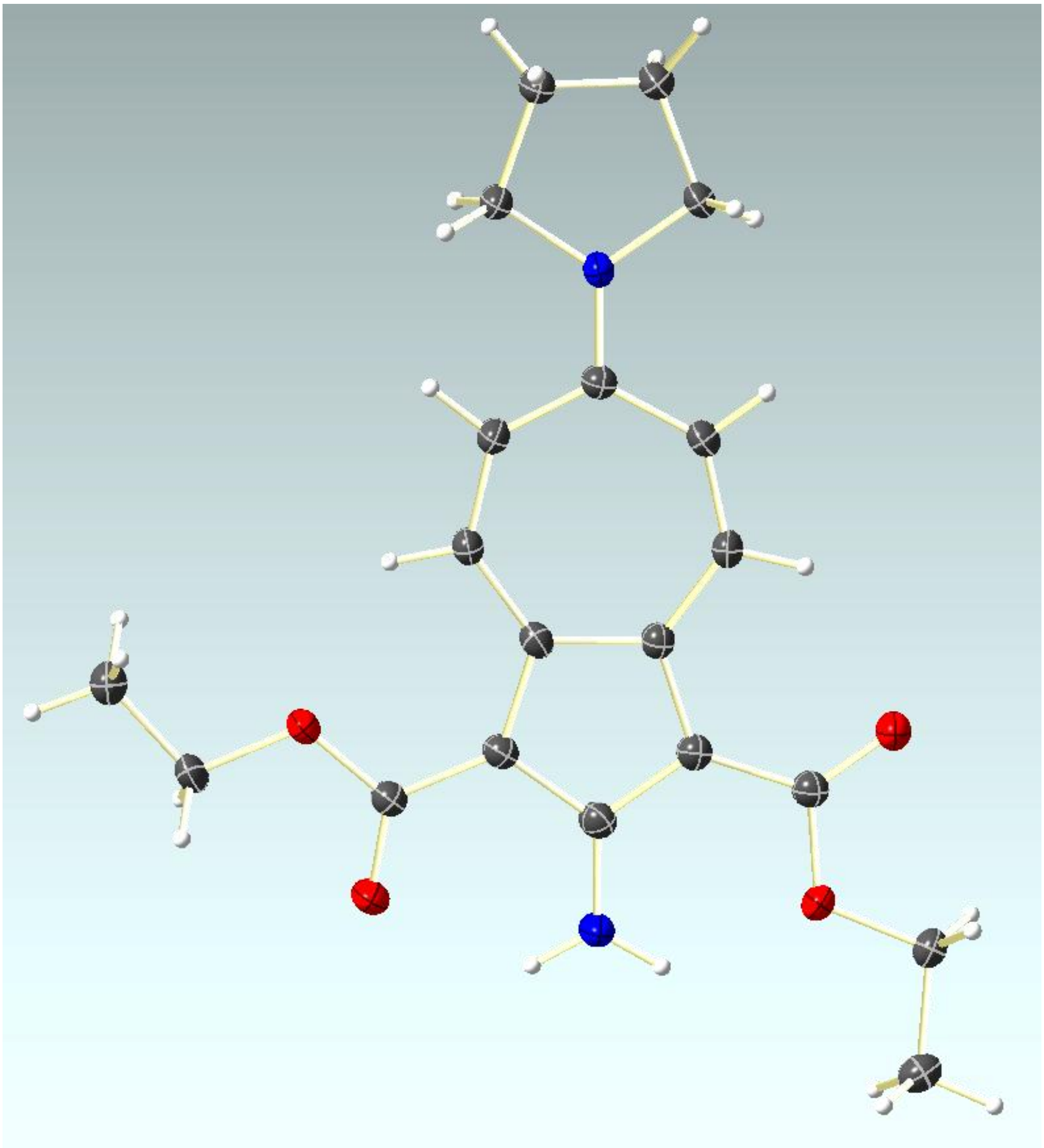

Crystal data and structure refinement for **15e**

Identification code	s23sel18
Empirical formula	$C_{19} H_{22} N_2 O_4$
Formula weight	342.38
Temperature	150.00(10) K
Wavelength	1.54184 Å
Crystal system	Monoclinic
Space group	I2/m
Unit cell dimensions	a = 15.1039(4) Å α= 90°.
	b = 6.8910(2) Å β= 112.290(4)°.
	c = 17.8683(6) Å γ = 90°.
Volume	1720.78(10) Å3
Z	4
Density (calculated)	1.322 Mg/m3
Absorption coefficient	0.763 mm-1
F(000)	728
Crystal size	0.250 x 0.050 x 0.040 mm3
Theta range for data collection	4.857 to 73.121°.
Index ranges	-12<=h<=18, -8<=k<=8, -22<=l<=21
Reflections collected	7934
Independent reflections	1868 [R(int) = 0.0369]
Completeness to theta = 67.684°	99.9 %
Absorption correction	Semi-empirical from equivalents
Max. and min. transmission	1.00000 and 0.62191
Refinement method	Full-matrix least-squares on F2
Data / restraints / parameters	1868 / 0 / 158
Goodness-of-fit on F2	1.035
Final R indices [I>2sigma(I)]	R1 = 0.0400, wR2 = 0.1013
R indices (all data)	R1 = 0.0459, wR2 = 0.1052
Extinction coefficient	n/a
Largest diff. peak and hole	0.306 and -0.196 e.Å-3

CCDC 2566803

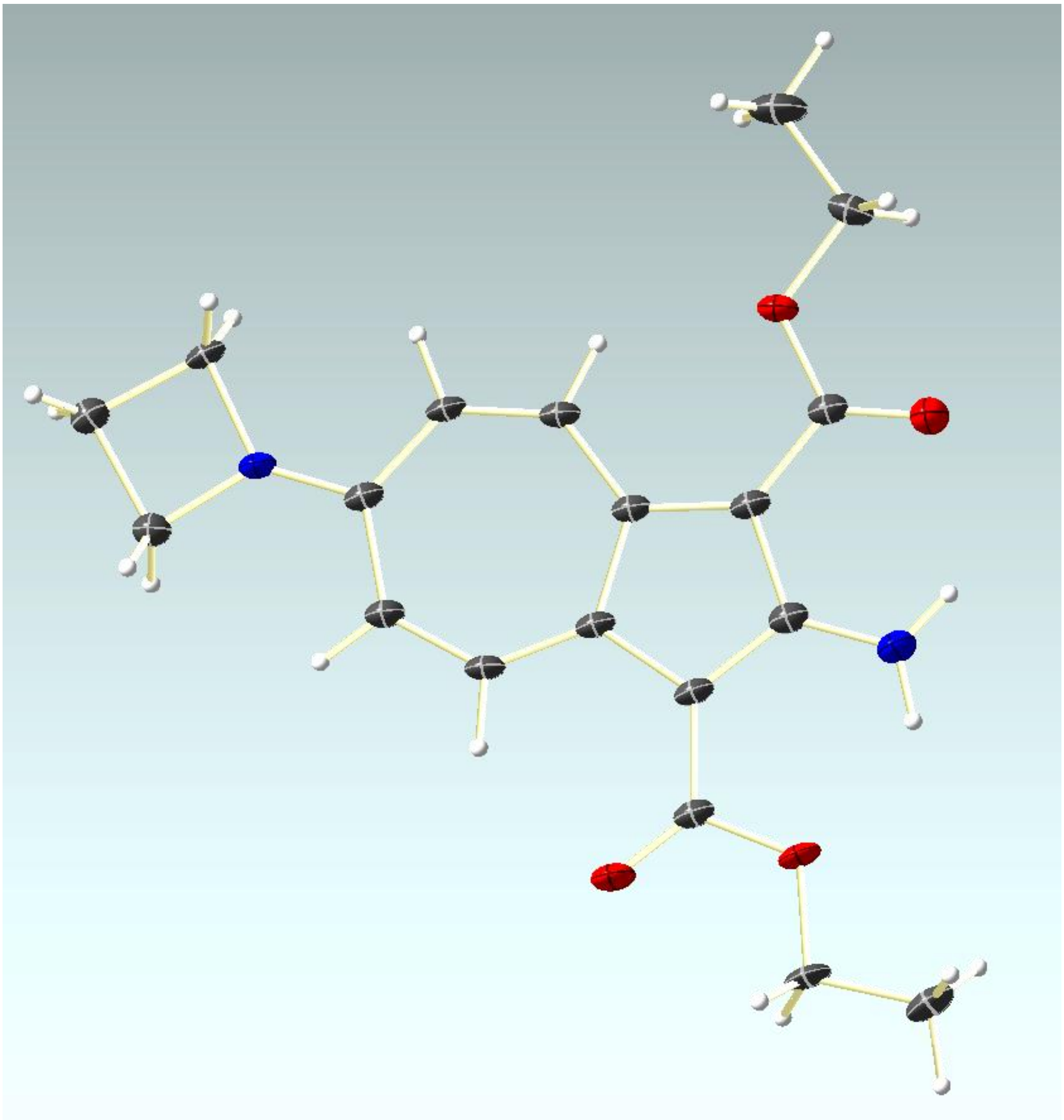

Crystal data and structure refinement for **15g**

Identification code	s23sel25
Empirical formula	$C_{26} H_{30} N_2 O_{4.50}$
Formula weight	442.52
Temperature	150.00(10) K
Wavelength	1.54184 Å
Crystal system	Monoclinic
Space group	I2
Unit cell dimensions	a = 15.7729(6) Å α= 90°.
	b = 5.6340(2) Å β= 92.387(3)°.
	c = 25.6761(10) Å γ = 90°.
Volume	2279.71(15) Å3
Z	4
Density (calculated)	1.289 Mg/m3
Absorption coefficient	0.714 mm-1
F(000)	944
Crystal size	0.505 x 0.052 x 0.028 mm3
Theta range for data collection	5.615 to 73.508°.
Index ranges	-19<=h<=19, -6<=k<=6, -26<=l<=31
Reflections collected	15541
Independent reflections	4443 [R(int) = 0.0675]
Completeness to theta = 67.684°	99.8 %
Absorption correction	Semi-empirical from equivalents
Max. and min. transmission	1.00000 and 0.46635
Refinement method	Full-matrix least-squares on F2
Data / restraints / parameters	4443 / 207 / 375
Goodness-of-fit on F2	1.075
Final R indices [I>2sigma(I)]	R1 = 0.0561, wR2 = 0.1429
R indices (all data)	R1 = 0.0650, wR2 = 0.1509
Absolute structure parameter	0.0(2)
Extinction coefficient	n/a
Largest diff. peak and hole	0.320 and -0.275 e.Å-3

CCDC 2566804

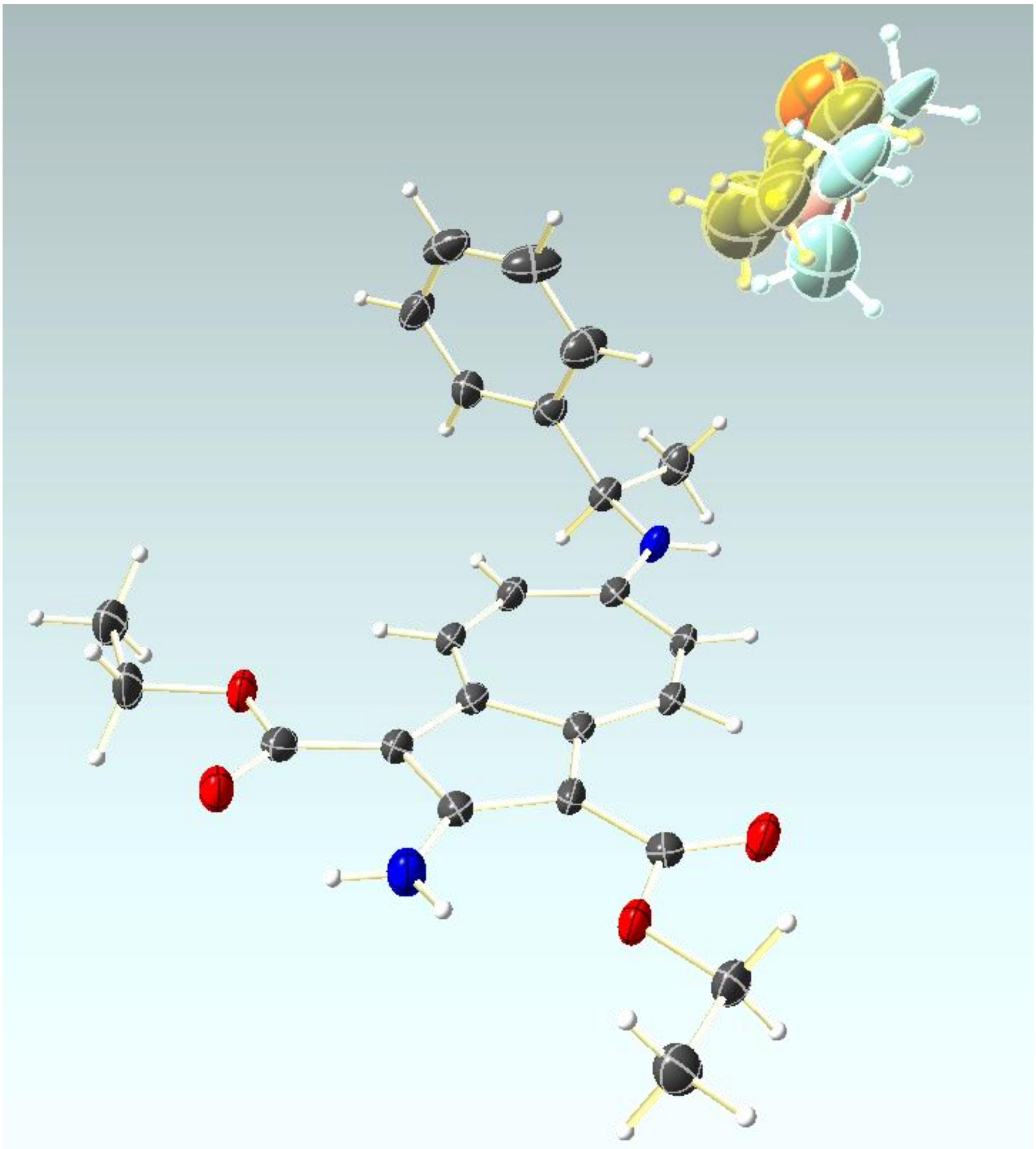

Notes on refinement:

The asymmetric unit contains one solvent molecule of THF which is disordered about a two-fold axis and was refined with 20% and 30% occupation. The N-H hydrogen atoms were located in the difference Fourier map and were freely refined.

Crystal data and structure refinement for **16**

Identification code	s23sel8
Empirical formula	$C_{18} H_{18} Br N O_5$
Formula weight	408.24
Temperature	150.00(10) K
Wavelength	1.54184 Å
Crystal system	Monoclinic
Space group	I2/a
Unit cell dimensions	a = 27.3856(7) Å α= 90°.
	b = 6.8863(3) Å β= 99.389(3)°.
	c = 37.5988(10) Å γ = 90°.
Volume	6995.6(4) Å3
Z	16
Density (calculated)	1.550 Mg/m3
Absorption coefficient	3.459 mm-1
F(000)	3328
Crystal size	0.250 x 0.030 x 0.020 mm3
Theta range for data collection	3.720 to 68.250°.
Index ranges	-32<=h<=32, -8<=k<=7, -44<=l<=45
Reflections collected	48725
Independent reflections	6406 [R(int) = 0.0628]
Completeness to theta = 67.684°	100.0 %
Absorption correction	Semi-empirical from equivalents
Max. and min. transmission	1.00000 and 0.86509
Refinement method	Full-matrix least-squares on F2
Data / restraints / parameters	6406 / 0 / 485
Goodness-of-fit on F2	1.025
Final R indices [I>2sigma(I)]	R1 = 0.0463, wR2 = 0.1112
R indices (all data)	R1 = 0.0690, wR2 = 0.1237
Extinction coefficient	n/a
Largest diff. peak and hole	0.827 and -0.806 e.Å-3
CCDC 2566805	

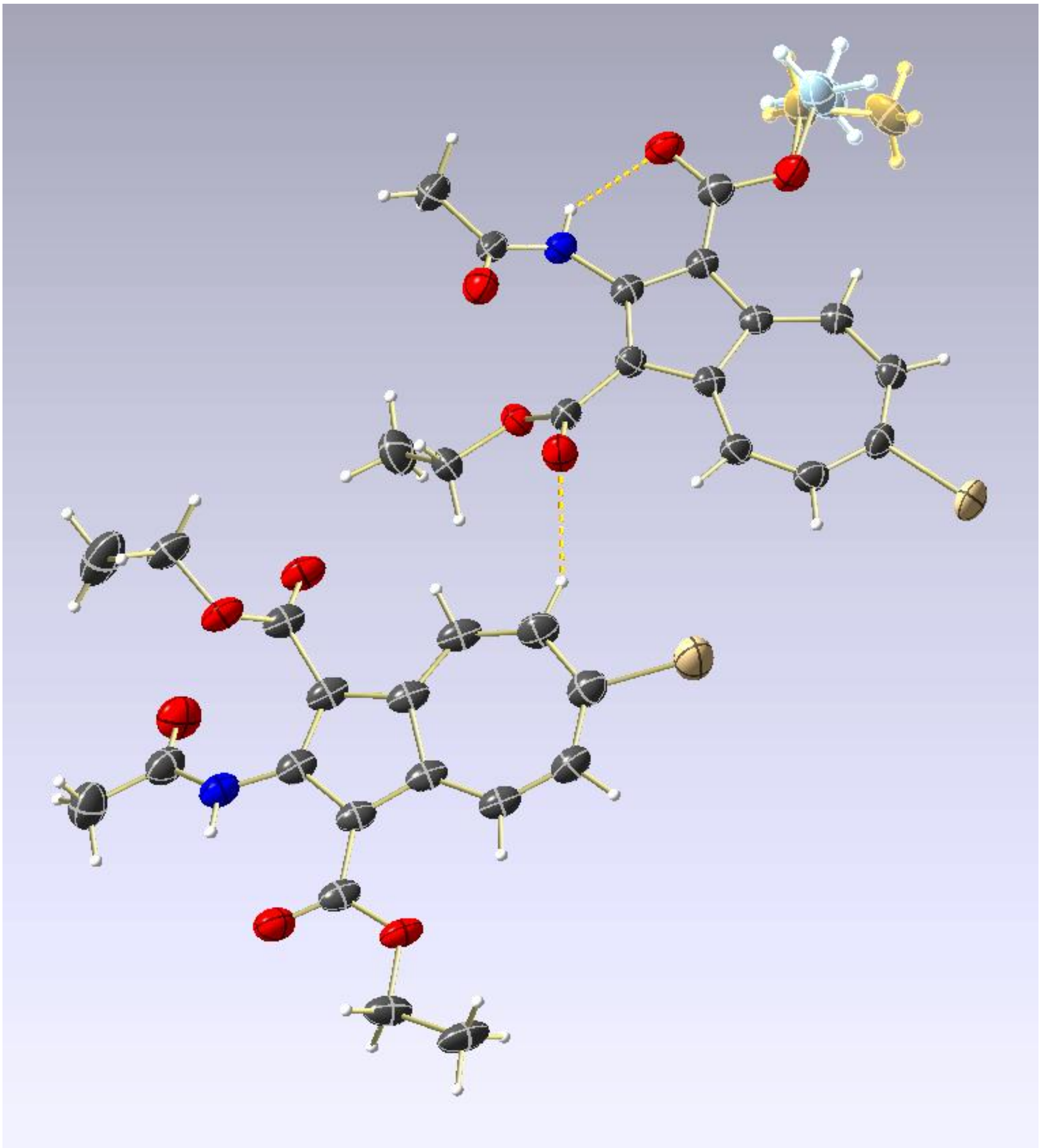

Note on refinement:

One ethyl group is disordered over two sites in the ratio 54:46.

The N-H hydrogen atoms have been located in the difference Fourier map and were refined freely.

Crystal data and structure refinement for **17**

Identification code	e23sel6
Empirical formula	$C_{20} H_{20} Br N O_6$
Formula weight	450.28
Temperature	149.9(6) K
Wavelength	0.71073 Å
Crystal system	Monoclinic
Space group	$P2_1/c$
Unit cell dimensions	a = 7.9753(4) Å α= 90°.
	b = 34.0614(15) Å β= 114.953(7)°.
	c = 7.9189(5) Å γ = 90°.
Volume	1950.4(2) Å3
Z	4
Density (calculated)	1.533 Mg/m3
Absorption coefficient	2.145 mm-1
F(000)	920
Crystal size	0.703 x 0.132 x 0.091 mm3
Theta range for data collection	3.061 to 26.351°.
Index ranges	-9<=h<=9, -42<=k<=42, -9<=l<=9
Reflections collected	27630
Independent reflections	3955 [R(int) = 0.0477]
Completeness to theta = 25.242°	99.9 %
Absorption correction	Semi-empirical from equivalents
Max. and min. transmission	1.00000 and 0.83362
Refinement method	Full-matrix least-squares on F2
Data / restraints / parameters	3955 / 0 / 257
Goodness-of-fit on F2	1.331
Final R indices [I>2sigma(I)]	R1 = 0.0614, wR2 = 0.0971
R indices (all data)	R1 = 0.0701, wR2 = 0.0994
Extinction coefficient	n/a
Largest diff. peak and hole	0.644 and -0.534 e.Å-3

CCDC 2566806

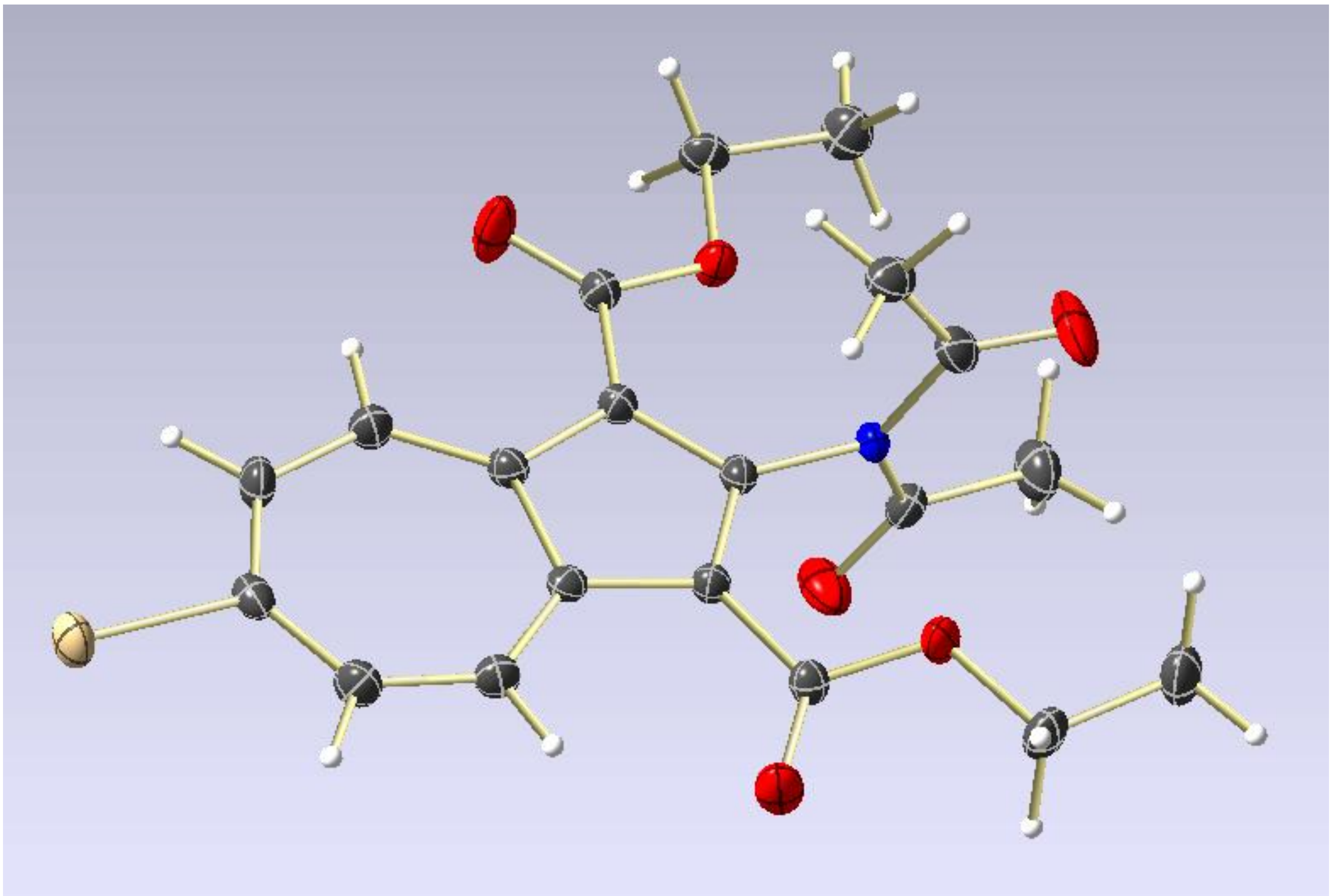

Crystal data and structure refinement for **18**

Identification code	s23sel15	
Empirical formula	$C_{18} H_{18} N_4 O_5$	
Formula weight	370.36	
Temperature	150.00(10) K	
Wavelength	1.54184 Å	
Crystal system	Monoclinic	
Space group	I2/a	
Unit cell dimensions	a = 23.5641(8) Å	α= 90°.
	b = 6.7840(3) Å	β= 94.914(3)°.
	c = 22.5908(7) Å	γ = 90°.
Volume	3598.1(2) Å3	
Z	8	
Density (calculated)	1.367 Mg/m3	
Absorption coefficient	0.854 mm-1	
F(000)	1552	
Crystal size	0.320 x 0.080 x 0.040 mm3	
Theta range for data collection	3.766 to 68.244°.	
Index ranges	-27<=h<=28, -8<=k<=8, -27<=l<=14	
Reflections collected	10969	
Independent reflections	3291 [R(int) = 0.0405]	
Completeness to theta = 67.684°	99.8 %	
Absorption correction	Semi-empirical from equivalents	
Max. and min. transmission	1.00000 and 0.60769	
Refinement method	Full-matrix least-squares on F2	
Data / restraints / parameters	3291 / 0 / 251	
Goodness-of-fit on F2	1.055	
Final R indices [I>2sigma(I)]	R1 = 0.0643, wR2 = 0.1721	
R indices (all data)	R1 = 0.0838, wR2 = 0.1880	
Extinction coefficient	n/a	
Largest diff. peak and hole	0.413 and -0.234 e.Å-3	

CCDC 2566807

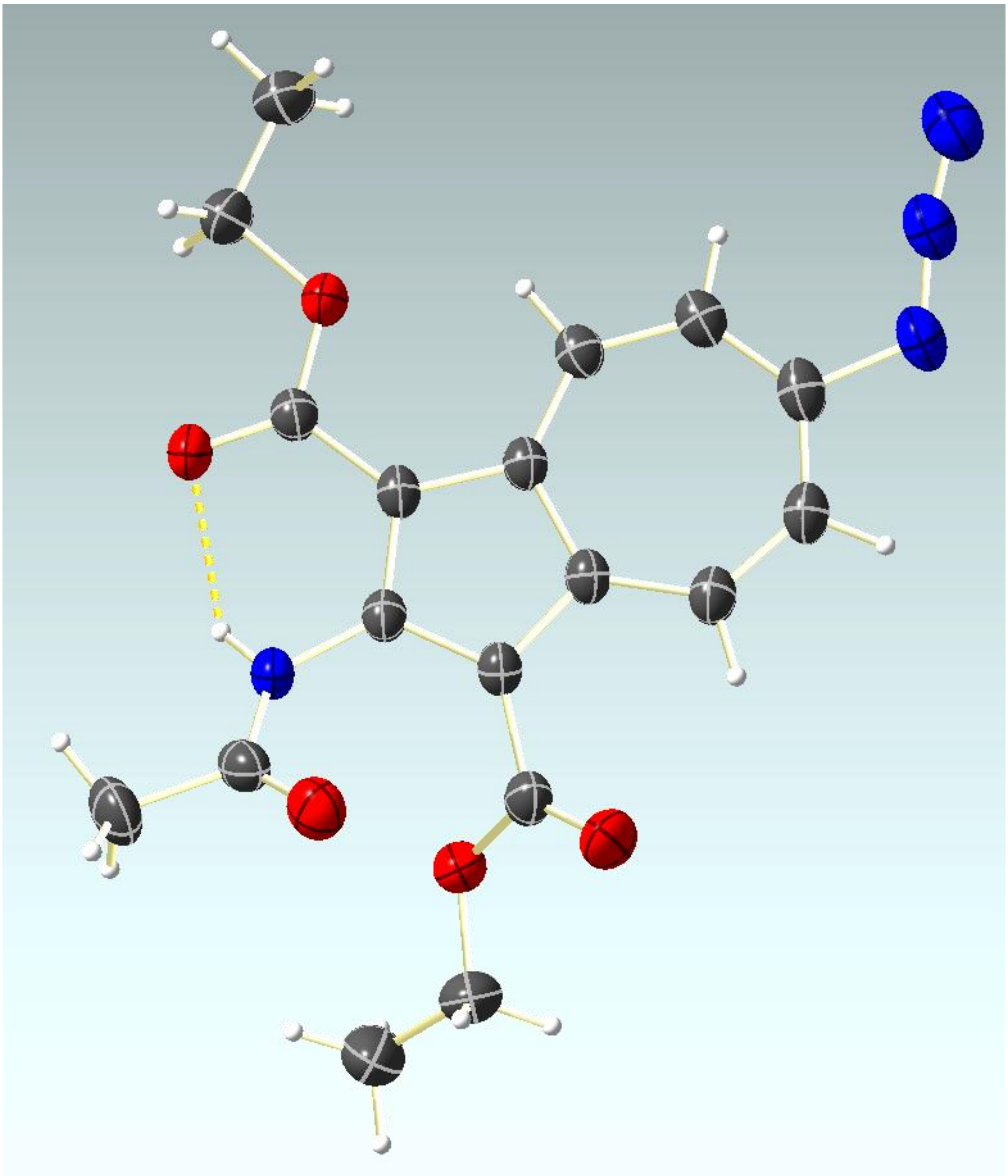

Crystal data and structure refinement for **28**

Identification code	s23sel29
Empirical formula	$C_{16} H_{17} N O_4$
Formula weight	287.30
Temperature	150.00(10) K
Wavelength	1.54184 Å
Crystal system	Monoclinic
Space group	$P2_1/c$
Unit cell dimension	a = 11.7574(2) Å α= 90°.
	b = 12.10517(18) Å β= 111.991(2)°.
	c = 10.6328(2) Å γ = 90°.
Volume	1403.21(5) Å3
Z	4
Density (calculated)	1.360 Mg/m3
Absorption coefficient	0.809 mm-1
F(000)	608
Crystal size	0.807 x 0.055 x 0.027 mm3
Theta range for data collection	4.055 to 72.842°.
Index ranges	-14<=h<=14, -14<=k<=10, -13<=l<=13
Reflections collected	20009
Independent reflections	2784 [R(int) = 0.0346]
Completeness to theta = 67.684°	100.0 %
Absorption correction	Gaussian
Max. and min. transmission	1.000 and 0.710
Refinement method	Full-matrix least-squares on F2
Data / restraints / parameters	2784 / 0 / 200
Goodness-of-fit on F2	1.030
Final R indices [I>2sigma(I)]	R1 = 0.0356, wR2 = 0.0858
R indices (all data)	R1 = 0.0404, wR2 = 0.0884
Extinction coefficient	n/a
Largest diff. peak and hole	0.211 and -0.209 e.Å-3
CCDC 2566808	

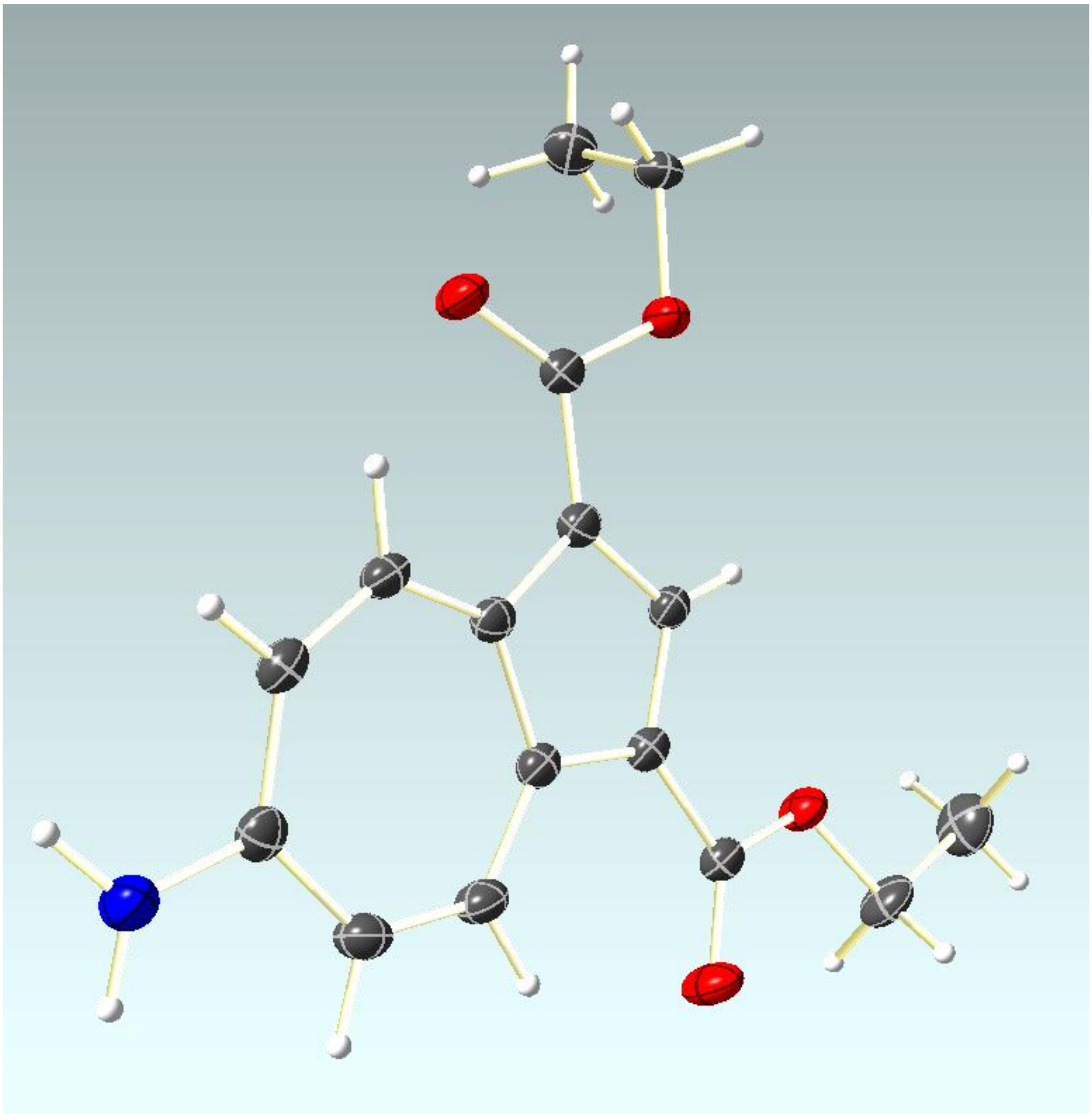

Crystal data and structure refinement for **31**

Identification code	s23sel14	
Empirical formula	$C_{18} H_{17} Br O_6$	
Formula weight	409.23	
Temperature	150.01(10) K	
Wavelength	1.54184 Å	
Crystal system	Triclinic	
Space group	P-1	
Unit cell dimensions	a = 7.2163(3) Å	α= 100.624(2)°.
	b = 8.2996(3) Å	β= 97.624(3)°.
	c = 14.6748(4) Å	γ = 96.217(3)°.
Volume	848.22(5) Å3	
Z	2	
Density (calculated)	1.602 Mg/m3	
Absorption coefficient	3.597 mm-1	
F(000)	416	
Crystal size	0.100 x 0.100 x 0.015 mm3	
Theta range for data collection	5.474 to 73.177°.	
Index ranges	-8<=h<=8, -10<=k<=10, -18<=l<=18	
Reflections collected	6495	
Independent reflections	6495 [R(int) = ?]	
Completeness to theta = 67.684°	99.8 %	
Absorption correction	Semi-empirical from equivalents	
Max. and min. transmission	1.00000 and 0.86352	
Refinement method	Full-matrix least-squares on F2	
Data / restraints / parameters	6495 / 0 / 230	
Goodness-of-fit on F2	1.014	
Final R indices [I>2sigma(I)]	R1 = 0.0451, wR2 = 0.1192	
R indices (all data)	R1 = 0.0518, wR2 = 0.1221	
Extinction coefficient	n/a	
Largest diff. peak and hole	0.825 and -0.683 e.Å-3	

CCDC 2566809

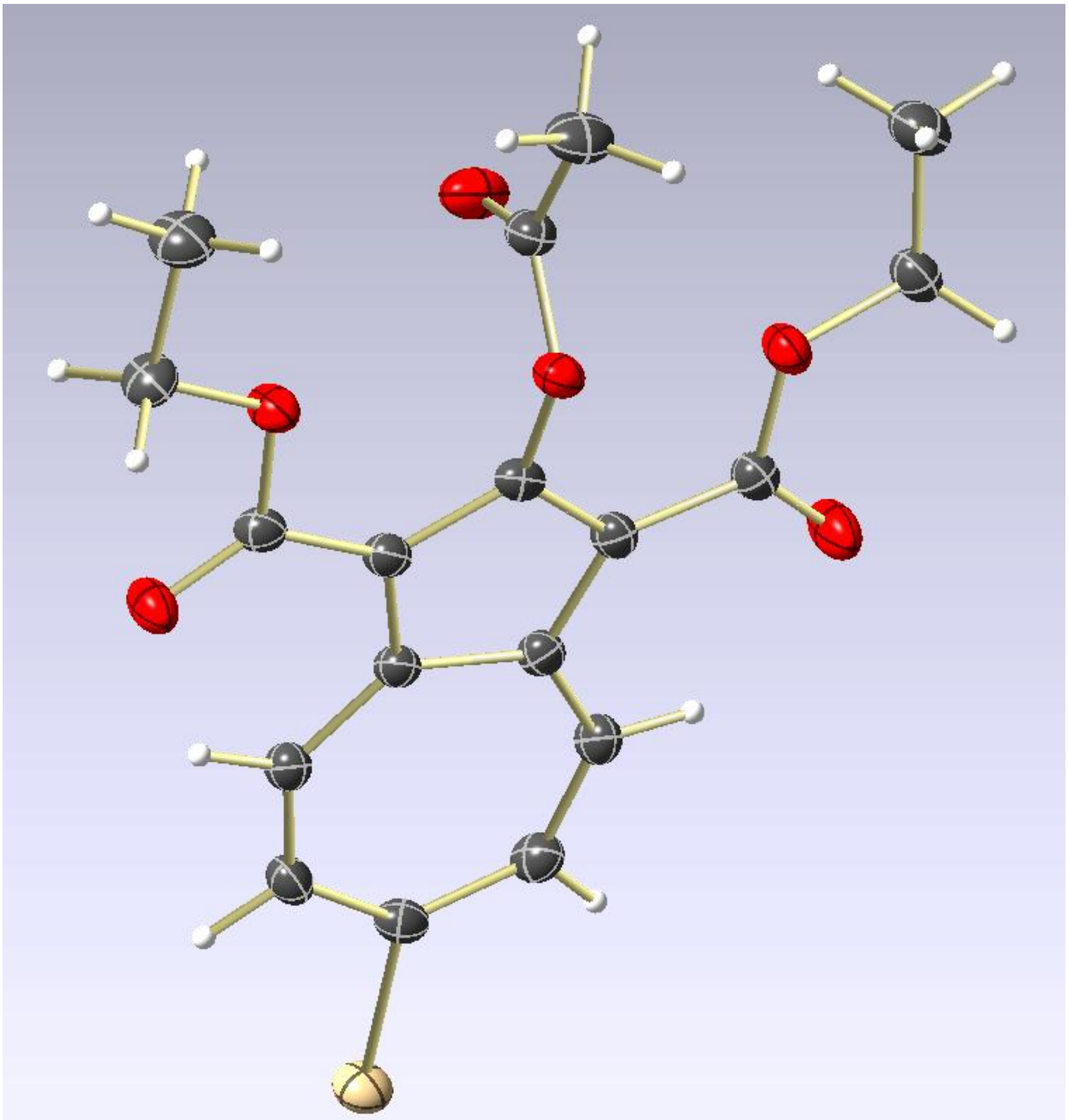

Notes on refinement:

Refined as a 2-component twin. Component 2 rotated by 179.8614°

around [0.79 0.45 -0.41] (reciprocal) or [0.90 0.44 -0.00] (direct)

References

[1] CrysAlisPro CrysAlisPro 1.171.38.41- CrysAlisPro 1.171.43.104a (Rigaku OD, 2015-2023)

[2] G. M. Sheldrick, Crystal structure refinement with SHELXL, *Acta Cryst.*, 2015, **C71**, 3-8.

[3] C. B. Hübschle, G. M. Sheldrick and B. Dittrich, ShelXle: a Qt graphical user interface for SHELXL, *J. Appl. Cryst.*, 2011, **44**, 1281-1284.

[4] C. F. Macrae, P. R. Edgington, P. McCabe, E. Pidcock, G. P. Shields, R. Taylor, M. Towler and J. van der Streek, Mercury: visualization and analysis of crystal structures, *J. Appl. Cryst.*, 2006, **39**, 453-457.

[5] T. Nozoe, S. Seto and S. Matsumura, Synthesis of 2-substituted azulenes by nucleophilic substitution reactions of 2-haloazulene derivatives. *Bull. Chem. Soc. Jpn.* 1962, **35**, 1990-1998.

[6] T. Nozoe, K. Takase and M. Tada, The synthesis of 5- and 6-aminoazulene derivatives. *Bull. Chem. Soc. Jpn.* 1963, **36**, 1006-1009.

[7] T. Shoji, Y. Fujiwara, A. Maruyama, M. Maruyama, S. Ito, M. Yasunami, R. Yokoyama and N. Morita, Synthesis of 2,6-diaminoazulenes by the S_NAr reaction with cyclic amines. *Heterocycles* 2015, **90**, 85-88.

[8] M. Tada, Anionoid substitution reaction of diethyl 2-acetamido-6-bromoazulene-1,3-dicarboxylate. *Bull. Chem. Soc. Jpn.* 1966, **39**, 1954-1961.

[9] K. J. Scheetz, A. D. Spaeth, A. S. Vorushilov, D. R. Powell, V. W. Day and M. V. Barybin, The 2,6-dimercaptoazulene motif: efficient synthesis and completely regioselective metallation of its 6-mercapto terminus. *Chem. Sci.* 2013, **4**, 4267-4272.

[10] K. Ohtsu, R. Hayami, T. Sagawa, S. Tsukada, K. Yamamoto and T. Gunji, Syntheses and properties of linear π-conjugated molecules composed of 1-azaazulene and azulene. *Tetrahedron* 2019, **75**, 130658.

[11] T. Nozoe, K. Takase and M. Tada, Anionoid substitution reaction of diethyl 6-bromoazulene-1,3-dicarboxylate. *Bull. Chem. Soc. Jpn.* 1965, **38**, 247-251.

[12] M. Mąkosza and R. Podraza, Hydroxylation and amination of azulenes by vicarious nucleophilic substitution of hydrogen. *Eur. J. Org. Chem.* 2000, 193-198.

[13] S. Ito, T. Okujima, C. Kabuto and N. Morita, Preparation, characterization, and cycloaddition reaction of the heterocumulenes attached directly to azulenes. An efficient strategy for the preparation of azulene-substituted heterocycles. *Tetrahedron* 2003, **59**, 4651-4659.

[14] M. Mąkosza, P. W. Osiński and S. Ostrowski, On amination and diazotization of azulene and its derivatives. *Polish Journal of Chemistry*, 2001, **75**, 275-281.

[15] T.-C. Huang, T. Morita, B.-B. Lin and Y.-S. Lin, Synthesis and diazotization of diethyl 6-amlno-2-hydroxyazulene-1,3-dicarboxylate and its 2-acetoxyl derivative. *J. Chin. Chem. Soc.*, 1994, **41**, 199-203.

[16] S. Kosuge, T. Morita and K. Takase, The synthesis of 6-methylen-2(6*H*)-azulenone (2,6-azulenoquinone 6-methide) derivatives. *Chem. Lett.* 1975, **4**, 733-736.
